# Haploid selection, sex ratio bias, and transitions between sex-determining systems

**DOI:** 10.1371/journal.pbio.2005609

**Published:** 2018-06-25

**Authors:** Michael Francis Scott, Matthew Miles Osmond, Sarah Perin Otto

**Affiliations:** 1 UCL Genetics Institute, Department of Genetics, Evolution and Environment, University College London, London, United Kingdom; 2 Department of Zoology, University of British Columbia, Vancouver, British Columbia, Canada; University of Zurich, Switzerland

## Abstract

Sex determination is remarkably dynamic; many taxa display shifts in the location of sex-determining loci or the evolution of entirely new sex-determining systems. Predominant theories for why we observe such transitions generally conclude that novel sex-determining systems are favoured by selection if they equalise the sex ratio or increase linkage with a locus that experiences different selection in males versus females. We use population genetic models to extend these theories in two ways: (1) We consider the dynamics of loci very tightly linked to the ancestral sex-determining loci, e.g., within the nonrecombining region of the ancestral sex chromosomes. Variation at such loci can favour the spread of new sex-determining systems in which the heterogametic sex changes (XY to ZW or ZW to XY) and the new sex-determining region is less closely linked (or even unlinked) to the locus under selection. (2) We consider selection upon haploid genotypes either during gametic competition (e.g., pollen competition) or meiosis (i.e., nonmendelian segregation), which can cause the zygotic sex ratio to become biased. Haploid selection can drive transitions between sex-determining systems without requiring selection to act differently in diploid males versus females. With haploid selection, we find that transitions between male and female heterogamety can evolve so that linkage with the sex-determining locus is either strengthened or weakened. Furthermore, we find that sex ratio biases may increase or decrease with the spread of new sex chromosomes, which implies that transitions between sex-determining systems cannot be simply predicted by selection to equalise the sex ratio. In fact, under many conditions, we find that transitions in sex determination are favoured equally strongly in cases in which the sex ratio bias increases or decreases. Overall, our models predict that sex determination systems should be highly dynamic, particularly when haploid selection is present, consistent with the evolutionary lability of this trait in many taxa.

## Author summary

Systems of sex determination are strikingly diverse and labile in many clades. This poses the question: what drives transitions between sex-determining systems? Here, we use models to derive conditions under which new sex-determining systems spread. Prevailing views suggest that new sex-determining systems are favoured when they equalise the sex ratio and/or when they are more closely linked to genes that experience differential selection in males and females. Our models include selection upon haploid genotypes (meiotic drive or gametic competition), which biases the sex ratio and occurs differently in male and female gametes. Surprisingly, we find the two forces (selection to equalise the sex ratio and the benefits of hitchhiking alongside driven alleles that distort the sex ratio) will often be equally strong, and thus neither is sufficient to explain the spread of new sex-determining systems in every case. We also find that new sex-determining alleles can spread despite being less closely linked to selected loci, as long as initial linkage is tight or haploid selection is present. Our models therefore predict that loci in previously unexpected genomic locations and/or experiencing various types of selection (including haploid selection) can now be implicated as drivers of transitions between sex-determining systems.

## Introduction

Animals and angiosperms exhibit extremely diverse sex-determining systems (reviewed in [1–5]). Among species with genetic sex determination (GSD), some taxa have heterogametic males (XY) and homogametic females (XX), including mammals and most dioecious plants [6], whereas other taxa have homogametic males (ZZ) and heterogametic females (ZW), including Lepidoptera and birds. Within several taxa, the chromosome that harbours the master sex-determining locus changes, due either to translocation of the master sex-determining locus or to the evolution of a new master locus. During these transitions, the heterogametic sex can remain the same (hereafter ‘*cis*-GSD transitions’) as in salmonids [7, 8], Diptera [9], and *Oryzias* [10]. Alternatively, species can switch between male and female heterogamety (XY↔ZW, hereafter ‘*trans*-GSD transitions’), as in snakes [11], lizards [12], eight of 26 teleost fish families [13], true fruit flies (tephritids, [9]), amphibians [14], the angiosperm genus *Silene* [15], the angiosperm family Salicaceae [16, 17], and Coleoptera and Hemiptera (plate 2 [3]). Indeed, in some cases, both male and female heterogametic sex-determining systems can be found in the same species, as reported in houseflies [18], midges [19], frogs [20], cichlid fish [21], tilapia [22], sea bass [23], and lab strains of zebrafish [24, 25]. In addition, multiple transitions have occurred between genetic and environmental sex determination (ESD) systems (GSD↔ESD), e.g., in reptiles and fishes [5, 12, 13, 26–29]. In sum, accumulating evidence indicates that transitions between sex-determining systems are common [4].

It has been suggested that sex ratio selection is a particularly dominant force in the evolution of sex determination (e.g., [1]; [3]). Classic ‘fisherian’ sex ratio selection favours a 1:1 zygotic sex ratio when assuming that males and females are equally costly to produce [30, 31]. This follows from the fact that, for an autosomal locus, half of the genetic material is inherited from a male and half from a female [32]. Thus, if the sex ratio is biased, an individual of the rarer sex will, on average, contribute more genetic material to the next generation. Selection therefore typically favours mutants that increase investment in the rarer sex, including new sex determination systems.

The evolution of sex determination is also thought to be strongly influenced by differences in selection between the sexes [3, 33, 34]. For example, loci experiencing sexual antagonism have been shown to favour the spread of new genetic sex-determining alleles that are closely linked [35–37]. Linkage allows a stronger favourable association to build up between a male-beneficial allele and a neo-Y allele, for example. Such associations can favour *cis*-GSD transitions [35], *trans*-GSD transitions [36], and new partially masculinising or partially feminising alleles in a population with ESD [37]. By similar logic, however, existing sexually antagonistic alleles associated with the current sex-determining locus are expected to hinder the spread of a new sex-determining system [35, 36].

One novel feature of the models developed here is that we explicitly consider the maintenance of genetic variation around the ancestral sex-determining locus (e.g., within the nonrecombining region of a sex chromosome). Counterintuitively, when linkage is tight between the sex-determining locus and a selected locus, an allele good for females can be at higher frequency on the ancestral Y than on the ancestral X under a variety of forms of selection. In addition, selection on ancestral X chromosomes in males can prevent the X from becoming optimally specialised for female-beneficial alleles. These factors, in turn, can favour a new ZW sex-determining locus that has weaker linkage with loci under selection, which was not revealed by previous theory [36]. A similar argument applies to ZW↔XY transitions. Thus, we show that selected loci in very tight linkage with the ancestral GSD locus can favour *trans*-GSD transitions, during which linkage associations are actually weakened.

Most significantly, we include haploid selection (gametic competition or meiotic drive) in models describing *cis*-GSD, *trans*-GSD, and GSD-to-ESD transitions. This poses an apparent evolutionary problem. On one hand, haploid selection is typically sex limited in that it usually occurs among gametes produced by one sex only [38–41]. Therefore, one might expect new sex-determining systems to benefit from close linkage with haploid-selected loci, as found for loci that experience diploid sex differences in selection [35–37]. On the other hand, associations between sex-determining loci and haploid-selected loci generate biased zygotic sex ratios, which should generally hinder the spread of new sex-determining systems.

Two previous studies have considered the spread of GSD with sex-limited meiotic drive [42, 43] under a limited number of possible genetic architectures and diploid-selective regimes. Ubeda and colleagues [43] considered ancestral ESD (with no sex ratio bias) and numerically showed that new GSD alleles can spread if they arise in linkage with meiotic drive loci. For example, a masculinising allele spreads in association with an allele that is favoured during male meiosis, causing sex ratios to become male biased. This suggests that the benefits of associating with driving alleles can overwhelm selection to balance the sex ratio. However, Kozielska and colleagues [42] considered an ancestral GSD system that is perfectly linked to a meiotic driver and therefore exhibiting an ancestral sex ratio bias. They found that a new, completely unlinked GSD system can spread if it generates the rarer sex, creating a balanced sex ratio. This suggests that fisherian sex ratio selection can overwhelm the benefits of being associated with driving alleles. It is thus currently unclear when haploid selection favours increased versus decreased linkage between haploid-selected loci and a new sex-determining locus. In addition, because the sex ratio is determined by linkage between haploid-selected loci and the sex-determining locus, it is also unclear when fisherian sex ratio selection is the most important driver of transitions between sex-determining systems.

Here, we analytically find the conditions under which new GSD or ESD systems spread in ancestral GSD systems with any degree of linkage between the loci involved and arbitrary forms of haploid and diploid selection. Doing so, we reconcile and generalise the results of Kozielska and colleagues [42] and Ubeda and colleagues [43] by deriving conditions for the spread of new GSD systems that alter linkage with haploid-selected loci. Our result is qualitatively distinct from those for diploid selection alone [35, 36] and suggests that haploid selection is more likely to promote transitions between sex determination systems. We also show that transitions involving haploid selection cannot be simply explained by invoking sex ratio selection. In particular, under a wide range of conditions, we show that transitions in sex-determining systems are favoured equally strongly in situations in which sex ratio biases increase or decrease (and in situations in which sex ratio biases are ancestrally present or absent). Finally, we show that ESD may not evolve, even if the sex ratio is initially biased by haploid selection, which is not predicted by previous theories for transitions to ESD [1, 31, 32]. Together, our results suggest that both selection to equalise the sex ratio and the benefits of associating with haploid-selected alleles can drive transitions between sex-determining systems, leading to stronger or weaker sex linkage and increased or decreased sex ratio bias.

### Model

**Fig 1:**
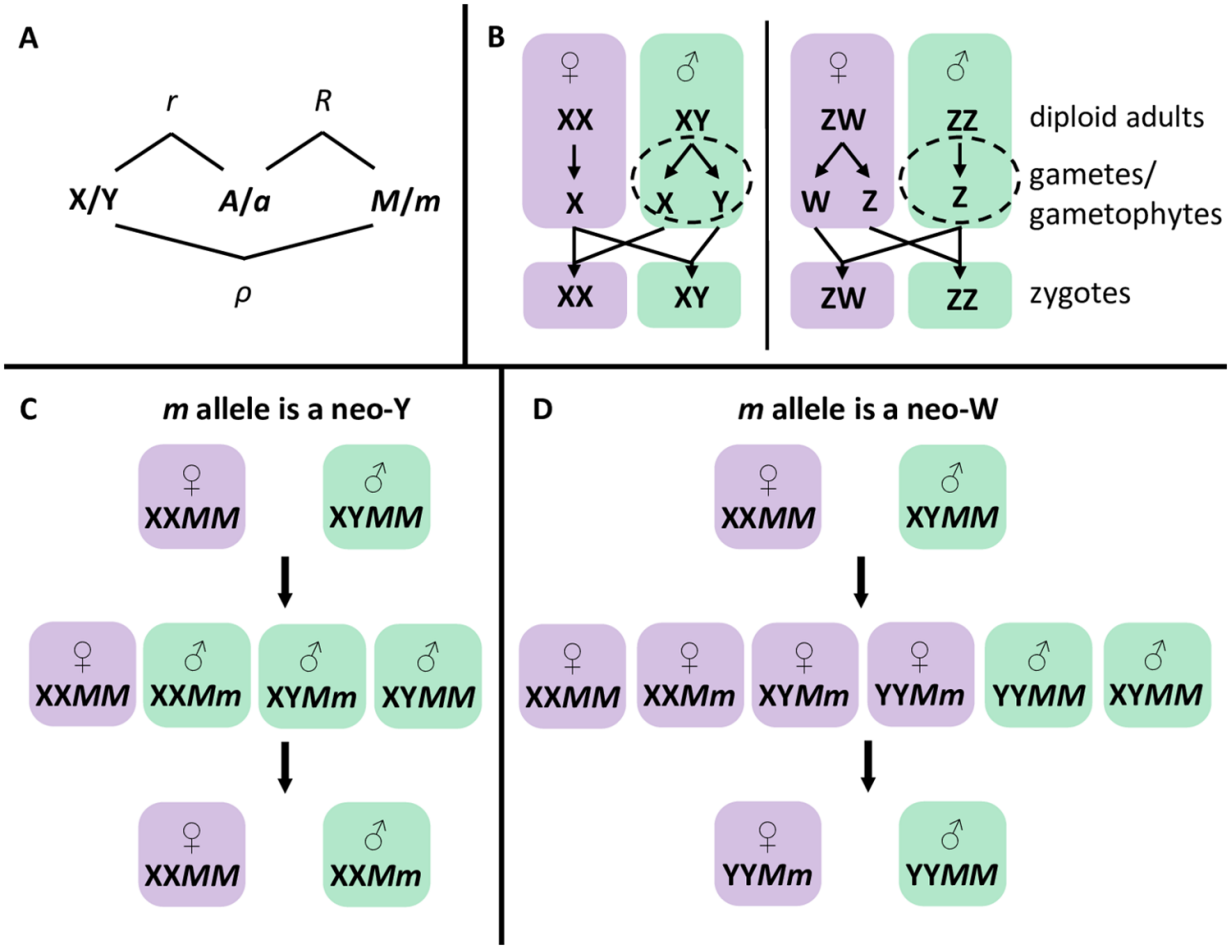
Outline of model features. Panel A: Recombination rate parameters between the ancestral sex-determining locus (**X**, here assumed to have alleles X and Y), a locus under selection (**A**, with alleles *A* and *a*), and a new sex-determining locus (**M**, with alleles *M* and *m*). Panel B: Haploid selection is often sex limited, occurring during haploid production or competition in one sex (shown here in males by dashed circles). If X or Y alleles are linked with alleles that experience haploid selection in males (*r* < 1/2), then zygotic sex ratios can become biased because either X- or Y-bearing male gametes/gametophytes will be more abundant after haploid selection. Similarly, zygotic sex ratio biases can arise if haploid-selected alleles are linked with new sex-determining alleles (*R* < 1/2). However, the zygotic sex ratio is not biased by male haploid selection in ZW sex-determining systems. Panel C: During *cis*-GSD transitions (XY to XY or ZW to ZW), a neo-Y allele (*m*) spreads until all males bear the neo-Y, and the ancestral Y allele is lost. Panel D: During *trans*-GSD transitions (XY to ZW or ZW to XY), a neo-W allele (*m*) spreads until all females bear the neo-W, and the ancestral X allele is lost. Neo-W alleles allow Y-associated alleles into females, which may impede or aid their spread. GSD, genetic sex determination.

We consider transitions between ancestral and novel sex-determining systems using a three-locus model, each locus having two alleles (Fig 1). A full description of our model, including recursion equations, is given in S1 Text. Locus **X** is the ancestral sex-determining region, with alleles X and Y (or Z and W). Locus **A** is a locus under selection, with alleles *A* and *a*. Locus **M** is a novel sex-determining region, at which the null allele (*M*) is initially fixed in the population such that sex of zygotes is determined by the genotype at the ancestral sex-determining region, **X**; XX genotypes become females, and XY become males (or ZW become females, and ZZ become males). To evaluate the evolution of new sex-determining systems, we consider the spread of a novel sex-determining allele (*m*) at the **M** locus.

Here, we assume that the **M** locus is ‘epistatically dominant’ over the **X** locus such that zygotes with at least one *m* allele develop as females with probability *k* and as males with probability 1 − *k*, regardless of the **X** locus genotype. With *k* = 0, the *m* allele is a masculiniser (a neo-Y allele), and with *k* = 1, the *m* allele is a feminiser (a neo-W allele). With intermediate *k*, we can interpret *m* as an ESD allele, such that zygotes develop as females in a proportion (*k*) of the environments they experience. The assumption that derived sex-determining loci are epistatically dominant is motivated by empirical systems in which multiple sex-determining alleles segregate (i.e., X, Y, Z, and W alleles present), such as cichlid fish [21], platyfish (*Xiphophorus maculatus* [44]), houseflies (*Musca domestica* [45]), western clawed frogs (*Xenopus tropicalis* [46]), and *Rana rugosa* [20]. Nevertheless, our supplementary analysis file (S1 File) allows other dominance relationships between loci to be specified (see also [35] supplementary material for a numerical analysis).

We consider two forms of selection upon haploid genotypes, ‘gametic competition’ and ‘meiotic drive’. During gametic competition, we assume that a representative sample of all gametes/gametophytes (hereafter ‘gametes’) compete with others of the same sex for fertilisation, which implies a polygamous mating system. Relative fitnesses in sex 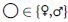 during gametic competition are given by 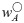 and 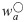 (see Table 1). On the other hand, meiotic drive in our model only affects the segregation of gametes produced by heterozygotes. Specifically, gametes produced by *Aa* heterozygotes of sex 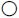 bear allele *A* with probability 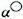. We note that competition between sperm produced by a single male (e.g., in a monogamous mating system) would be appropriately modelled as male meiotic drive, as only the frequency of gametes produced by heterozygotes would be affected. However, we do not consider scenarios in which there is competition among gametes produced by a small number of males/females (e.g., [47]).

**Table 1:** Relative fitness of different genotypes in sex, 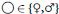.

**Genotype**	**Relative fitness during gametic competition**
*A*	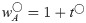
*a*	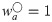
**Genotype**	**Relative fitness during diploid selection**
*AA*	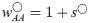
*Aa*	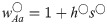
*aa*	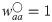
**Genotype**	**Transmission during meiosis in *Aa* heterozygotes**
*A*	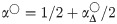
*a*	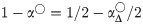

In each generation, we census the genotype frequencies in male and female gametes before gametic competition. After gametic competition, conjugation between male and female gametes occurs at random. The resulting zygotes develop as males or females, depending on their genotypes at the **X** and **M** loci. Diploid males and females then experience viability and/or individual-based fertility selection, with relative fitnesses 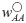, 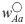, and 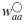. We do not consider fertility selection that depends on the mating partner, e.g., sexual selection with variation in choosiness. The next generation of gametes is produced by meiosis, during which recombination and sex-specific meiotic drive can occur. Recombination (i.e., an odd number of crossovers) occurs between loci **X** and **A** with probability *r*, between loci **A** and **M** with probability *R*, and between loci **X** and **M** with probability *ρ*. Any linear order of the loci can be modelled with appropriate choices of *r*, *R*, and *ρ* (see Fig 1A and S1 Table). Our model is entirely deterministic and hence ignores chance fluctuations in allele frequencies due to genetic drift.

The model outlined above describes both ancestral XY and ZW sex-determining systems. Without loss of generality, we refer to the ancestrally heterogametic sex as male and the ancestrally homogametic sex as female. That is, we primarily describe an ancestral XY sex-determining system, but our model is equally applicable to an ancestral ZW sex-determining system (relabelling the ancestrally heterogametic sex as female and the ancestrally homogametic sex as male and switching the labels of males and females throughout). We use a superscript to specify the ancestral sex-determining system described, e.g., ^(*XY*)^ for ancestral XY sex-determination.

In the ancestral population, it is convenient to follow the frequency of the *A* allele among female gametes (eggs), $p_{X}^{♀}$, and among X-bearing, $p_{X}^{♂}$, or among Y-bearing, $p_{Y}^{♂}$, male gametes (sperm/pollen). We also track the fraction of male gametes that are Y-bearing, *q*_*Y*_, which may deviate from 1/2 because of meiotic drive in males. We consider only equilibrium frequencies of alleles, 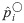, and Y-bearing male gametes, ${\hat{q}}_{Y}$, when determining the invasion of new sex-determining factors. We use *ξ* to measure the sex ratio (fraction male) among zygotes, which is determined by the allele frequencies and haploid selection coefficients (S2 Table).

## Results

We begin by describing the general conditions under which new GSD alleles can spread within a population, without explicitly specifying ancestral allele frequencies. These general conditions then allow us to consider several special cases of interest in subsequent sections, in which equilibrium ancestral allele frequencies are explicitly calculated. Finally, we consider the spread of alleles that cause sex to be determined environmentally.

### Generic invasion by a neo-Y or neo-W

The evolution of a new sex-determining system requires that a rare mutant allele, *m*, at the novel sex-determining locus, **M**, increases in frequency when rare. Specifically, *m* invades when $\lambda_{m}^{\left(XY\right)}>1$, in which $\lambda_{m}^{\left(XY\right)}$ is the leading eigenvalue of the system of eight equations describing *m*-bearing gamete frequencies, Eqs S1.1. This system simplifies substantially for an epistatically dominant neo-Y (*k* = 0) or neo-W (*k* = 1); see S3 Text for details.

Invasion by a neo-Y or a neo-W primarily depends on the ‘haplotypic growth rates’ (denoted by $\Lambda_{mi}^{\left(XY\right)}$) of the neo-sex determination allele *m* on background *i* ∈ {*A*,*a*}, without accounting for loss due to recombination (*R* = 0); see Table 2. If both haplotypic growth rates are greater than 1 ($\Lambda_{mA}^{\left(XY\right)}$, $\Lambda_{ma}^{\left(XY\right)}>1$), then the new sex-determining allele invades regardless of the rate of recombination between the new sex-determining locus and the selected locus (*R*). Conversely, if both haplotypic growth rates are less than 1 ($\Lambda_{mA}^{\left(XY\right)}$, $\Lambda_{ma}^{\left(XY\right)}<1$), then invasion can never occur. Finally, if only one haplotypic growth rate is greater than 1, the new sex-determining allele can always invade when arising at a locus that is tightly linked to the selected locus (*R* ≈ 0). Furthermore, it can be shown that the leading eigenvalue declines with recombination rate, *R*, and invasion requires that *R* is sufficiently small such that

$$\chi_{ma}^{\left(XY\right)}/\left(\Lambda_{ma}^{\left(XY\right)}-1\right)+\chi_{mA}^{\left(XY\right)}/\left(\Lambda_{mA}^{\left(XY\right)}-1\right)<1.$$ (1)

Here, $\chi_{mi}^{\left(XY\right)}>0$ is the rate at which mutant haplotypes on background *i* ∈ {*A*,*a*} recombine onto the other **A** locus background in heterozygotes (which is proportional to *R*; see Table 2). This is a ‘dissociative force’ that breaks down linkage disequilibrium.

**Table 2:** Parameters determining invasion of mutant neo-Y and neo-W alleles into an ancestrally XY system.

*m* is a neo-Y (*k* = 0)
$\Lambda_{Y^{\prime }A}^{\left(XY\right)}={\left(2\zeta \right)}^{-1}\left[{\hat{p}}_{X}^{♀}w_{A}^{♀}w_{A}^{♂}w_{AA}^{♂}+(1-{\hat{p}}_{X}^{♀})w_{a}^{♀}w_{A}^{♂}w_{Aa}^{♂}(1+\alpha_{△}^{♂})\right]/\left({\bar{w}}_{H}^{♀}{\bar{w}}_{H}^{♂}{\bar{w}}_{D}^{♂}\right)$
$\Lambda_{Y^{\prime }a}^{\left(XY\right)}={\left(2\zeta \right)}^{-1}\left[\left(1-{\hat{p}}_{X}^{♀})w_{a}^{♀}w_{a}^{♂}w_{aa}^{♂}+{\hat{p}}_{X}^{♀}w_{A}^{♀}w_{a}^{♂}w_{Aa}^{♂}(1-\alpha_{△}^{♂}\right)\right]/\left({\bar{w}}_{H}^{♀}{\bar{w}}_{H}^{♂}{\bar{w}}_{D}^{♂}\right)$
$\chi_{Y^{\prime }A}^{\left(XY\right)}=R{\left(2\zeta \right)}^{-1}\left[\left(1-{\hat{p}}_{X}^{♀})w_{a}^{♀}w_{A}^{♂}w_{Aa}^{♂}(1+\alpha_{△}^{♂}\right)\right]/\left({\bar{w}}_{H}^{♀}{\bar{w}}_{H}^{♂}{\bar{w}}_{D}^{♂}\right)$
$\chi_{Y^{\prime }a}^{\left(XY\right)}=R{\left(2\zeta \right)}^{-1}\left[{\hat{p}}_{X}^{♀}w_{A}^{♀}w_{a}^{♂}w_{Aa}^{♂}(1-\alpha_{△}^{♂})\right]/\left({\bar{w}}_{H}^{♀}{\bar{w}}_{H}^{♂}{\bar{w}}_{D}^{♂}\right)$
*m* is a neo-W (*k* = 1)
$\Lambda_{W^{\prime }A}^{\left(XY\right)}={\left[2(1-\zeta )\right]}^{-1}\left[{\bar{p}}^{♂}w_{A}^{♂}w_{A}^{♀}w_{AA}^{♀}+(1-{\bar{p}}^{♂})w_{A}^{♂}w_{A}^{♀}w_{Aa}^{♀}(1+\alpha_{\Delta }^{♀})\right]/\left({\bar{w}}_{H}^{♀}{\bar{w}}_{H}^{♂}{\bar{w}}_{D}^{♀}\right)$
$\Lambda_{W^{\prime }a}^{\left(XY\right)}={\left[2(1-\zeta )\right]}^{-1}\left[\left(1-{\bar{p}}^{♂})w_{a}^{♂}w_{a}^{♀}w_{aa}^{♀}+{\bar{p}}^{♂}w_{A}^{♂}w_{a}^{♀}w_{Aa}^{♀}(1-\alpha_{\Delta }^{♀}\right)\right]/\left({\bar{w}}_{H}^{♀}{\bar{w}}_{H}^{♂}{\bar{w}}_{D}^{♀}\right)$
$\chi_{W^{\prime }A}^{\left(XY\right)}=R{\left[2(1-\zeta )\right]}^{-1}\left[\left(1-{\bar{p}}^{♂})w_{a}^{♂}w_{A}^{♀}w_{Aa}^{♀}(1+\alpha_{\Delta }^{♀}\right)\right]/\left({\bar{w}}_{H}^{♀}{\bar{w}}_{H}^{♂}{\bar{w}}_{D}^{♀}\right)$
$\chi_{W^{\prime }a}^{\left(XY\right)}=R{\left[2(1-\zeta )\right]}^{-1}\left[{\bar{p}}^{♂}w_{A}^{♂}w_{a}^{♀}w_{Aa}^{♀}(1-\alpha_{\Delta }^{♀})\right]/\left({\bar{w}}_{H}^{♀}{\bar{w}}_{H}^{♂}{\bar{w}}_{D}^{♀}\right)$

${\bar{p}}^{♂}=(1-{\hat{q}}_{Y}){\hat{p}}_{X}^{♂}+{\hat{q}}_{Y}{\hat{p}}_{Y}^{♂}$ is the average frequency of the *A* allele among X- and Y-bearing male gametes. ${\hat{q}}_{Y}$ is the frequency of Y-bearing male gametes. *ξ* is the zygotic sex ratio (fraction male). 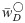 is the mean fitness of diploids of sex 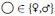. 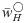 is the mean fitness of haploids from sex 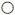 (see S2 Table). *R* is the rate of recombination between the neo-sex determiner and the selected locus. Selection terms (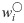, 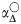) are described in Table 1.

Condition 1 may or may not be satisfied for the full range of locations of the new sex-determining locus, including *R* = 1/2 (e.g., on an autosome), depending on the nature of selection. Interpreting this condition, if we assume that only the *mA* haplotype would increase in frequency when *R* = 0 (i.e., $\Lambda_{ma}^{\left(XY\right)}<1<\Lambda_{mA}^{\left(XY\right)}$), then the first term on the left-hand side of Eq (1) is negative, and invasion requires that the growth rate of *mA* haplotypes ($\Lambda_{mA}^{\left(XY\right)}-1>0$) and the rate at which they are produced by recombination ($\chi_{ma}^{\left(XY\right)}$) are sufficiently large relative to the rate of decline of *ma* haplotypes ($1-\Lambda_{ma}^{\left(XY\right)}>0$) and the rate at which *m* and *A* are dissociated by recombination ($\chi_{mA}^{\left(XY\right)}$).

The haplotypic growth rates and dissociative forces are listed in Table 2 for a neo-Y and neo-W invading an ancestrally XY system. From this table and the arguments above, we draw four main points about the generic invasion of neo-Y and neo-W mutations (without specifying the ancestral equilibrium): (1) Fisherian sex ratio selection will favour the spread of a neo-W and inhibit the spread of a neo-Y if the ancestral zygotic sex ratio is biased towards males (i.e., the first factor of the $\Lambda_{mi}^{\left(XY\right)}$ is greater than 1 for a neo-W and less than 1 for a neo-Y when *ξ* > 1/2). Thus, neo-sex-determining alleles that specify the rarer sex are favoured by fisherian sex ratio selection. (2) In addition, the new sex-determining allele has associations with alleles favoured by either haploid or diploid selection (fitness terms in square brackets). Importantly, invasion by a neo-Y (neo-W) does not directly depend on the fitness of female (male) diploids. This is because a dominant neo-Y (neo-W) is always found in males (females), and therefore the frequency of the neo-Y (neo-W), *m*, only changes in males (females), Fig 1C and 1D. (3) Haploid selection thus plays two roles, generating fisherian selection to equalise the ancestral sex ratio (through *ξ*) and generating selection for the neo-Y/neo-W through associations with haploid-selected loci, which can distort the sex ratio. Each role influences the invasion dynamics of a new sex-determining allele, allowing the sex ratio to become more or less biased during a transition (as previously found in two special cases; [42, 43]). (4) Finally, Table 2 shows that the genetic contexts that arise during *cis*- and *trans*-GSD transitions are qualitatively different. This is because, in an ancestrally XY system, a gamete with the neo-Y always pairs with a female gamete containing an X, Fig 1C. By contrast, a gamete with a neo-W can pair with an X- or Y-bearing male gamete, Fig 1D. Consequently, neo-W-bearing females obtain a different frequency of *A* alleles from mating compared to ancestral (*MM*) females (${\bar{p}}^{♂}$ versus ${\hat{p}}_{X}^{♂}$, respectively). This can inhibit or favour the spread of a neo-W.

In order to explicitly determine the conditions under which a new sex-determining allele spreads, we next calculate the equilibrium frequency of the *A* allele (i.e., ${\hat{p}}_{X}^{♀}$, ${\hat{p}}_{X}^{♂}$, and ${\hat{p}}_{Y}^{♂}$) and Y-bearing male gametes (${\hat{q}}_{Y}$) in the ancestral population. Because only the **A** locus experiences selection directly, any deterministic evolution requires that there be a polymorphism at the **A** locus. Polymorphisms can be maintained by mutation-selection balance or occur transiently during the spread of beneficial alleles. Here, however, we focus on polymorphisms maintained by selection for longer periods. Such polymorphisms can be maintained by heterozygote advantage, sexually antagonistic selection, ploidally antagonistic selection, or a combination [48]. We analytically calculate equilibrium frequencies using two alternative simplifying assumptions: (1) the **A** locus is tightly linked to the nonrecombining region around the ancestral sex-determining locus (*r* ≈ 0), or (2) selection is weak relative to recombination (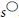, 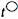, 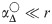). The ancestral equilibria and their stability conditions are given in S2 Text.

### Tight linkage with the ancestral sex-determining locus (*r* ≈ 0)

When there is complete linkage between the ancestral sex-determining locus and the selected locus **A** (*r* = 0), either the *A* allele or the *a* allele must be fixed in gametes containing a Y allele (S2 Text). Because the labelling of alleles is arbitrary, we will assume that the *a* locus is fixed in gametes with a Y (${\hat{p}}_{Y}^{♂}=0$), without loss of generality. If there are two alleles maintained at the **A** locus, the *A* allele can be fixed (${\hat{p}}_{X}^{♀}={\hat{p}}_{X}^{♂}=1$) or segregating at an intermediate frequency ($0<{\hat{p}}_{X}^{♀},{\hat{p}}_{X}^{♂}<1$) in gametes with an X.

We find that a neo-Y allele can never invade an ancestral XY system that already has tight linkage with the locus under selection ($\lambda_{Y^{\prime }}^{\left(XY\right)}\leq 1$ when *r* = 0; for details, see S1 File). In essence, through tight linkage with the **A** locus, the ancestral Y becomes strongly specialised on the allele that has the highest fitness across male haploid and diploid phases. It is thus not possible for a neo-Y to create males that have higher fitness than the ancestral Y, and *cis*-GSD transitions are never favoured.

Neo-W alleles, on the other hand, can invade an ancestral XY system (the full invasion conditions are given in S3 Text; Eqs S3.1 and S3.2). Invasion occurs when neo-W females can have higher fitness than the XX females in the ancestral population. Neo-W invasion is possible under all forms of selection that can maintain a polymorphism (sexually antagonistic selection, overdominance, ploidally antagonistic selection, or some combination, e.g., S2, S3 and S8 Figs). Thus,

**Conclusion 1: Selection on loci in or near the nonrecombining region around the ancestral sex-determining locus (*r* ≈ 0) prevents *cis*-GSD transitions (XY** ↔ **XY, ZW** ↔ **ZW) but can spur *trans*-GSD transitions (XY** ↔ **ZW)**.

To clarify conditions under which *trans*-GSD transitions can occur, we focus here on cases in which there is no haploid selection (and hence no sex ratio bias) and discuss the additional effect of haploid selection in S3 Text. Broadly, it is possible for neo-W females to have higher fitness than XX females for two reasons. Firstly, because the ancestral X experiences selection in both males and females, the X may be unable to specialise strongly on an allele favoured in females. Secondly, an allele can be associated with the Y and yet favoured in females. In turn, a neo-W can spread because (a) it is only found in females and is therefore unleashed from counterselection in males (corresponding to $\Lambda_{W^{\prime }A}^{\left(XY\right)}>1$), and/or (b) it allows Y-associated alleles into females (corresponding to $\Lambda_{W^{\prime }a}^{\left(XY\right)}>1$).

**Fig 2:**
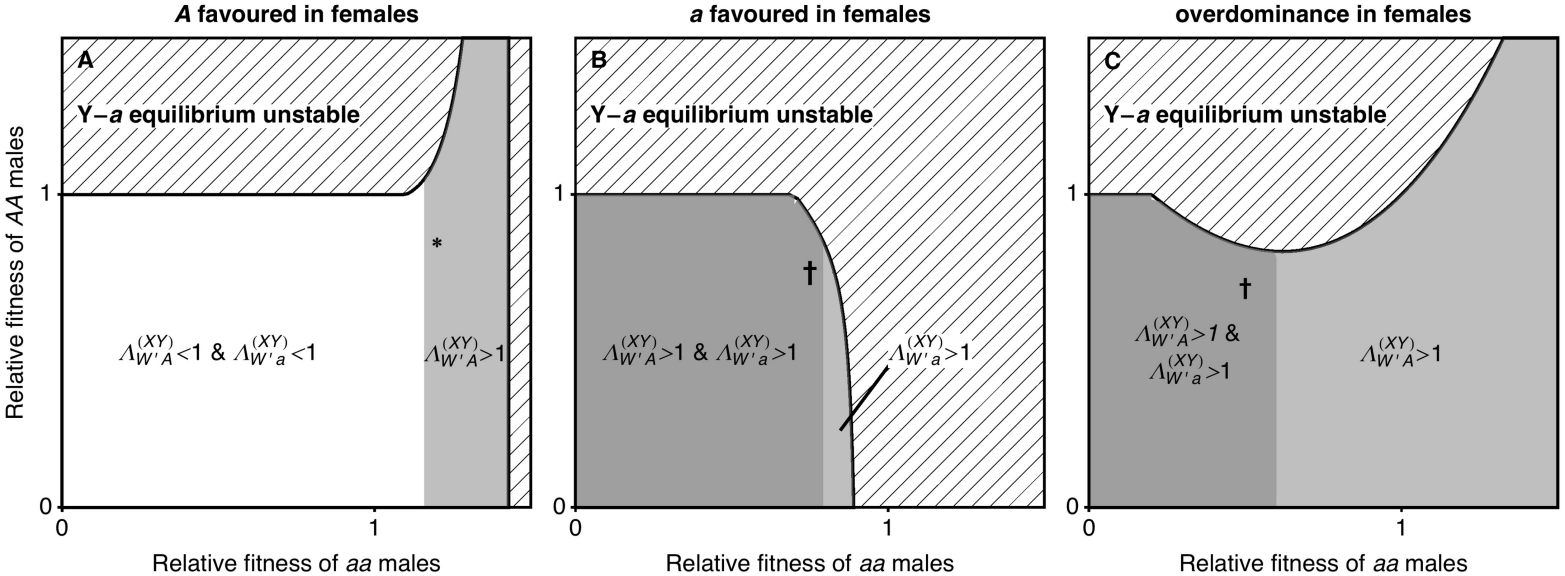
When the ancestral XY locus is tightly linked to a locus under selection (*r* ≈ 0), one or both neo-W haplotypes can spread (no haploid selection). We vary the fitness of male homozygotes relative to heterozygotes (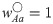) and only consider stable equilibria at which both **A** locus alleles are maintained and the *a* allele is initially fixed on the Y (nonhatched region). Here, selection in females can favour the *A* allele (panel A, $w_{aa}^{♀}=0.85$, $w_{AA}^{♀}=1.05$), favour the *a* allele (panel B, $w_{aa}^{♀}=1.05$, $w_{AA}^{♀}=0.85$), or be overdominant (panel C, $w_{aa}^{♀}=w_{AA}^{♀}=0.6$). If either haplotypic growth rate ($\Lambda_{W^{\prime }A}^{\left(XY\right)}$ or $\Lambda_{W^{\prime }a}^{\left(XY\right)}$) is greater than 1, then a rare neo-W allele can spread for at least some values of *R* > *r* (grey regions). The parameter values marked with an asterisk correspond to the fitnesses used in Fig 3C. S3 Fig shows the dynamics arising with the parameters marked with a dagger.

We first give an example in which neo-W-*A* haplotypes can spread because the neo-W is unleashed from counterselection in males (case [a], in which $\Lambda_{W^{\prime }A}^{\left(XY\right)}>1$). When *A* is female beneficial and *a* is male beneficial, the *A* allele can be fixed (${\hat{p}}_{X}^{♀}={\hat{p}}_{X}^{♂}=1$) or polymorphic ($0<{\hat{p}}_{X}^{♀},{\hat{p}}_{X}^{♂}<1$) on the X. In this case, polymorphism on the ancestral X indicates suboptimal specialisation for females fitness, which occurs because the *A* allele is counterselected in males (requires that $w_{Aa}^{♂}$ be sufficiently small relative to $w_{aa}^{♂}$). Neo-Ws, however, spend no time in males and can build stronger associations with the female-beneficial *A* allele, allowing them to spread (see grey region in Fig 2A).

We next give an example in which neo-W-*a* haplotypes can spread because they bring in female-beneficial alleles associated with the Y (case [b], in which $\Lambda_{W^{\prime }a}^{\left(XY\right)}>1$). When there is overdominance in males, X-*A* Y-*a* males have high fitness, and the *A* allele is favoured by selection on the X background in males. Therefore, the *A* allele can be polymorphic or even fixed on the X background, despite selection favouring the *a* allele in females (e.g., see nonhatched region in Fig 2B and [49, 50]). In such cases, neo-W-*a* haplotypes can spread because they create more *Aa* and *aa* females when pairing with an X-bearing gamete from males and because they bring more of the Y-*a* haplotype into females, in whom it has higher fitness (Fig 1D).

In some cases, both neo-W-*A* and neo-W-*a* haplotypes can spread. For example, when *AA* individuals have low fitness in females, yet the *A* is polymorphic or fixed on the X background due to overdominance in males (Fig 2B and 2C), both neo-W-*A* and neo-W-*a* haplotypes produce fewer unfit *AA* females. This is true for the neo-W-*A* haplotype because it can pair with a Y-*a* haplotype and still be female. Whenever both haplotypic growth rates are greater than 1, invasion by a neo-W is expected regardless of its linkage with the selected locus (i.e., for any *R*); see S1 and S2 Figs for examples. As a consequence, evolution can favour a new sex determination system on a different chromosome (*R* = 1/2), despite the fact that this unlinks the sex-determining locus from the selected locus.

**Fig 3:**
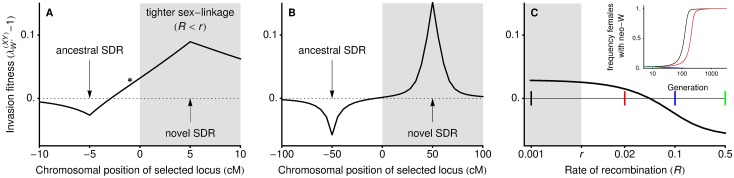
Transitions between XY and ZW systems can occur even when the new sex-determining locus is less tightly linked to a locus under sexually antagonistic selection (no haploid selection). In panel A, linkage is initially tight relative to selection, and a neo-W can invade (above dashed line) even when it is less tightly linked with the selected locus (*r* < *R*; unshaded region around *). In panel B, linkage is loose enough relative to selection that the analytical results assuming weak selection hold, and a neo-W allele can only invade when it arises at a locus more tightly linked with the selected locus (*R* < *r*; shaded region). In panel C, we vary the recombination rate between the neo-W and the selected locus (*R*) for a fixed recombination rate between the ancestral sex-determining locus and the selected locus (*r* = 0.005). Coloured markers show recombination rates for which the temporal dynamics of invasion are plotted in the inset (frequency of females carrying a neo-W), demonstrating that neo-W alleles can reach fixation if they are more (black) or less (red) closely linked to a locus experiencing sexually antagonistic selection. A very loosely linked neo-W does not spread in this case (blue and green lines overlap and go to 0 in inset). Fitness parameters are $w_{AA}^{♀}=1.05$, $w_{aa}^{♂}=1.2$, $w_{aa}^{♀}=w_{AA}^{♂}=0.85$, 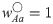. SDR, sex-determining region.

When only one neo-W haplotype has a growth rate greater than 1 (see Fig 2), a neo-W allele can invade as long as Eq (1) is satisfied, which may require that the recombination rate, *R*, is small enough. Nevertheless, because we assume here that *r* is small, these results indicate that a more loosely linked sex-determining region (*r* < *R*) can spread. For example, tightly sex-linked loci that experience sexually antagonistic selection can drive *trans*-GSD transitions in which the new sex-determining locus is less closely linked (*R* > *r*, Fig 3), but the analysis in S1 File indicates that a new unlinked sex-determining allele (*R* = 1/2) cannot invade when selection is purely sexually antagonistic (directional selection in each sex and no haploid selection).

Assuming selection is weak relative to recombination, van Doorn and Kirkpatrick [36] showed that invasion by a neo-W allele occurs under the same conditions as its fixation in females. An equivalent analysis is not possible when recombination rates are low. However, numerical simulations demonstrate that, with tight sex linkage, neo-Y or neo-W alleles do not necessarily reach fixation in males or females, respectively, which can lead to the stable maintenance of a mixed sex-determining system, in which X, Y, and neo-W alleles all segregate (e.g., S9B and S9C Fig).

From the arguments above, we reach

**Conclusion 2: With tight linkage between a selected locus and the ancestral sex-determining locus (***r* ≈ 0**), *trans*-GSD transitions (XY** ↔ **ZW) can be favoured by selection even if they weaken sex-linkage (***r* < *R***), potentially shifting sex determination to a different chromosome (***R* = 1/2**). Such transitions can also lead to the maintenance of multifactorial sex determination systems**.

With haploid selection, Conclusions 1 and 2 continue to apply (S3 Text). The parameters for which neo-W-*A* and neo-W-*a* haplotypes spread under various forms of haploid selection are plotted in S4, S5, S6 and S7 Figs. In particular, we note that adding haploid selection allows shifts in sex determination to a different chromosome (*R* = 1/2) even when selection is sexually antagonistic, with directional selection in each diploid sex, e.g., S3 Fig. Furthermore, haploid selection allows variation to be maintained by ploidally antagonistic selection, under which *trans*-GSD transitions may also be favoured, S8 Fig. Some cases of XY → ZW transitions in which *r* = 0, *R* = 1/2, and selection is ploidally antagonistic (meiotic drive in males opposed by diploid selection) were studied by Kozielska and colleagues [42], who found that sex ratio biases are reduced during these transitions. However, such transitions are not always driven by selection to reduce sex ratio bias. For example, with XY sex determination and haploid selection in females, sex ratios are not ancestrally biased, yet a neo-W can invade (S8 Fig). We further discuss how the spread of neo-sex-determining alleles is influenced by associations with haploid-selected loci in the next section.

### Loose linkage with the ancestral sex-determining region

Here, we assume that selection is weak (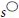, 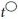, 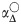 of order *ε*, in which *ε* is some number much less than 1) and thus implicitly assume that all recombination rates (*r*, *R*, and *ρ*) are large relative to selection. To leading order in selection,

$$\lambda_{Y^{\prime }}^{\left(XY\right)}=1+\frac{1}{4}\bar{p}(1-\bar{p})S_{A}{}^{2}\frac{\left(r-R\right)}{rR}+O\left(\varepsilon^{3}\right)$$ (2)

and

$$\lambda_{W^{\prime }}^{\left(XY\right)}=\lambda_{Y^{\prime }}^{\left(XY\right)}+\left[\left(2\alpha_{\Delta }^{♂}-2\alpha_{\Delta }^{♀}+t^{♂}-t^{♀}\right)\left({\hat{p}}_{Y}^{♂}-{\hat{p}}_{X}^{♂}\right)/2\right]+O\left(\varepsilon^{3}\right),$$ (3)

in which $\bar{p}$ is the frequency of *A*, to leading order (Eq S2.3), and $S_{A}=({\bar{s}}^{♂}+\alpha_{\Delta }^{♂}+t^{♂})-({\bar{s}}^{♀}+\alpha_{\Delta }^{♀}+t^{♀})$ describes sex differences in selection for the *A* versus *a* allele across diploid selection, meiosis, and gametic competition. The diploid selection term, 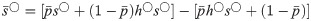, is the difference in fitness between *A* and *a* alleles in diploids of sex 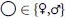. The difference in *A* allele frequency among Y-bearing sperm versus X-bearing sperm is, at equilibrium, ${\hat{p}}_{Y}^{♂}-{\hat{p}}_{X}^{♂}=\bar{p}(1-\bar{p})S_{A}(1-2r)/(2r)$.

Eq (2) demonstrates that, under weak selection, a neo-Y allele will invade an XY system ($\lambda_{Y^{\prime }}^{\left(XY\right)}>1$) if and only if it is more closely linked to the selected locus than the ancestral sex-determining locus (i.e., if *R* < *r*). This echoes our results above, in which a neo-Y could never invade if *r* ≈ 0. It is also consistent with the results of [35], who considered diploid selection only and also found that *cis*-GSD transitions can only occur when the new sex-determining locus is more closely linked to a locus under sexually antagonistic selection.

**Conclusion 3A: New sex-determining alleles causing *cis*-GSD transitions (XY** ↔ **XY or ZW** ↔ **ZW) are favoured if they arise more closely linked with a locus that experiences (haploid and/or diploid) selection than the ancestral sex-determining locus (***R* < *r***)**.

**Fig 4:**
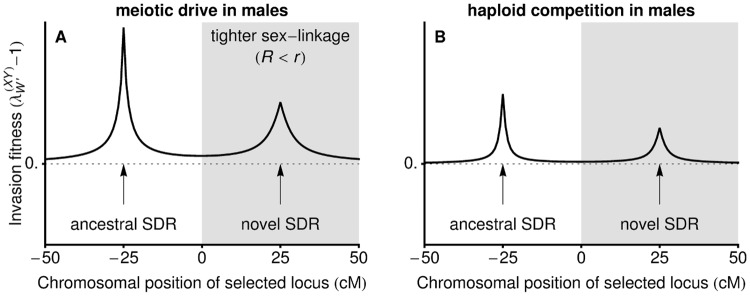
Ploidally antagonistic selection allows a less tightly linked neo-W allele to invade. In panel A, male drive ($\alpha_{\Delta }^{♂}=-1/20$, 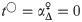) opposes selection in diploids (no sex differences: 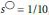, 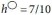). In panel B, gametic competition in males (*t*^♂^ = −1/10, 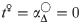) opposes selection in diploids (sex differences: *s*^♂^ = 3/20, *s*^♀^ = 1/20, 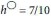). In either case, the new sex-determining allele can invade (above dashed line) regardless of *R*, even when linkage to the selected locus is reduced (white regions). SDR, sex-determining region.

Similarly, in the absence of haploid selection (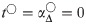), Eq (3) indicates that *trans*-GSD transitions can occur if and only if the new sex-determining locus is more closely linked to a locus under selection, *R* < *r*, as found by [36]. With haploid selection, a neo-W is also usually favoured when it is more closely linked to the selected locus than the ancestral sex-determining region is (*R* < *r*, e.g., Figs 3B and 4); this is true unless the last term in Eq (3) is negative and dominant over the first, which requires relatively restrictive combinations of selection and recombination parameters. For example, with haploid selection, a neo-W will always be favoured if it arises in linkage with a selected locus (*R* < 1/2) that is ancestrally autosomal (*r* = 1/2, leading to ${\hat{p}}_{Y}^{♂}-{\hat{p}}_{X}^{♂}=0$).

**Conclusion 3B: New sex-determining alleles causing *trans*-GSD transitions (XY** ↔ **ZW) are usually favoured if they arise more closely linked with a locus that experiences (haploid and/or diploid) selection than the ancestral sex-determining locus (***R* < *r***)**.

However, with haploid selection and some ancestral sex linkage (*r* < 1/2; allowing allele frequency differences on the X and Y), the term in square brackets in Eq (3) can be positive. This leads to

**Conclusion 3C: With haploid selection, new sex-determining alleles causing *trans*-GSD transitions (XY** ↔ **ZW) can spread even if they arise further from a locus that experiences selection than the ancestral sex-determining locus (***r* < *R***)**.

To clarify the parameter space under which neo-W alleles spread despite looser linkage with the selected locus (*R* > *r*), we focus on cases in which dominance coefficients are equal in the two sexes, *h*^♀^ = *h*^♂^, and haploid selection only occurs in one sex (e.g., during male meiosis only). Table 3 then gives the conditions required for unlinked (*R* = 1/2) neo-W invasion when there is some ancestral sex linkage (*r* < 1/2; e.g., the selected locus is on the ancestral sex chromosome, and the novel sex-determining locus arises on an autosome). These special cases indicate that neo-W invasion occurs for a large fraction of the parameter space, even though the neo-W uncouples the sex-determining locus from a locus under selection. Fig 4 then demonstrates that under these conditions, neo-W alleles can spread when they are more loosely or more closely linked to the locus that experiences haploid selection, i.e., Conclusions 3B and 3C (compare with Fig 3A for diploid sexually antagonistic selection alone).

**Table 3:** Invasion conditions for a neo-W allele at an unlinked locus (*R* = 1/2) into an ancestral XY system with linkage (*r* < 1/2) and a single form of haploid selection.

Scenario	Assumptions	neo-W spreads ($\lambda_{W^{\prime }}^{\left(XY\right)}>1$) if
male drive only	*h*^♂^ = *h*^♀^, $t^{♀}=t^{♂}=\alpha_{\Delta }^{♀}=0$	*s*^♀^ *s*^♂^ > 0
female drive only	*h*^♂^ = *h*^♀^, $t^{♀}=t^{♂}=\alpha_{\Delta }^{♂}=0$	*s*^♀^ *s*^♂^ > 0
male gametic competition only	*h*^♂^ = *h*^♀^, $t^{♀}=\alpha_{\Delta }^{♀}=\alpha_{\Delta }^{♂}=0$	*s*^♀^(*s*^♂^ − *s*^♀^) > 0
female gametic competition only	*h*^♂^ = *h*^♀^, $t^{♂}=\alpha_{\Delta }^{♀}=\alpha_{\Delta }^{♂}=0$	*s*^♂^(*s*^♀^ − *s*^♂^) > 0

**Fig 5:**
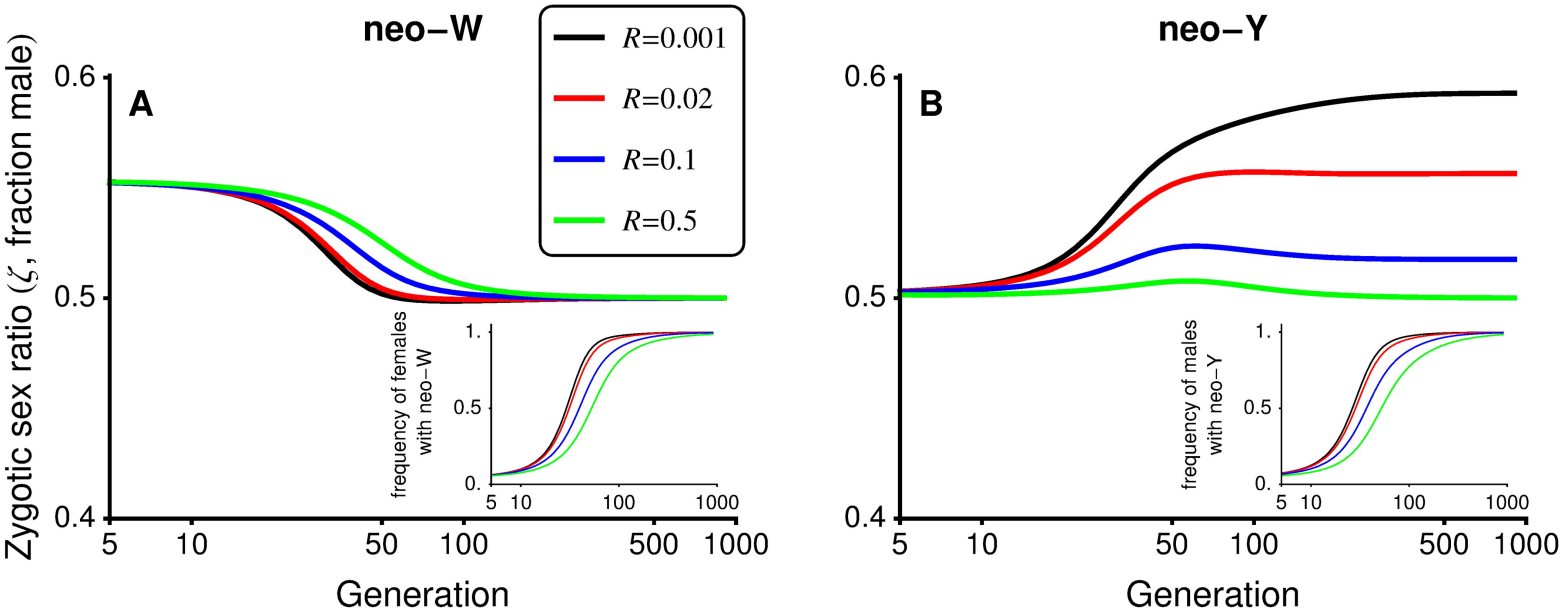
Fisherian sex ratio selection alone is not a good predictor of turnover between sex-determining systems. In this figure, selection is ploidally antagonistic, with haploid selection favouring the *a* allele during male meiosis. In panel A, male meiotic drive in an ancestral XY system causes a male bias (see Fig 1B), allowing a neo-W to invade and replace the ancestral sex-determining system (inset shows the frequency of females carrying a neo-W), which balances the zygotic sex ratio. In panel B, male drive in an ancestral ZW system has no effect on the zygotic sex ratio (50:50 at generation 0), yet a neo-Y can invade and replace the ancestral sex-determining system (inset shows the frequency of males carrying a neo-Y). Parameters: *s*^♀^ = *s*^♂^ = 0.2, *h*^♀^ = *h*^♂^ = 0.7, $t^{♀}=t^{♂}=\alpha_{\Delta }^{♀}=0$, $\alpha_{\Delta }^{♂}=-0.1$, *r* = 0.02.

We can also compare transitions among different GSD systems, as these are associated with different effects on the sex ratio, which can increase, decrease, or remain equal. For example, if there is meiotic drive in males only ($\alpha_{\Delta }^{♂}\neq 0$, $\alpha_{\Delta }^{♀}=0$), without gametic competition (*t*^♀^ = *t*^♂^ = 0), the zygotic sex ratio is initially biased only when the ancestral sex-determining system is XY (Figs 1B and 5A) and not ZW (Figs 1B and 5B). If fisherian sex ratio selection dominated, we would thus expect a difference in the potential for XY-to-ZW and ZW-to-XY transitions. However, invasion by a neo-W allele into an XY system and invasion by a neo-Y allele into a ZW system occur under the same conditions ($\lambda_{Y^{\prime }}^{\left(XY\right)}=\lambda_{W^{\prime }}^{\left(ZW\right)}$ and $\lambda_{W^{\prime }}^{\left(XY\right)}=\lambda_{Y^{\prime }}^{\left(ZW\right)}$, at least to order *ε*^2^), implying that

**Conclusion 4: When selection is weak relative to recombination, *trans*-GSD transitions in the presence of haploid selection are favoured as often and as strongly, whether they erase ancestral sex ratio bias (benefiting from fisherian sex ratio selection) or generate sex ratio bias (benefiting from associations with selected alleles)**.

For example, in Fig 5A, neo-W alleles invade an ancestral XY system in which females are initially rare, equalising the sex ratio (as occurs in [42]). However, Fig 5B shows that a neo-Y can invade the resulting ZW system under the same conditions. When *R* < 1/2, the invading neo-Y becomes associated with the male meiotic drive allele, and the zygotic sex ratio evolves to become male biased (as occurs in [43], beginning from ESD). In this case, the neo-Y spreads because it is often found in males and can, if it carries the driven allele *a*, benefit from haploid selection in males (Fig 5B).

While equalising the sex ratio and benefiting from associations with selected alleles are two primary reasons why haploid selection spurs sex chromosome transitions, more complex situations also arise. For example, with *R* = 1/2 in Fig 5B (green curve), the neo-Y allele spreads despite the fact that it cannot benefit from drive because free recombination moves it randomly between driven and nondriven backgrounds. Nevertheless, the unlinked neo-Y can spread because males bearing it more often carry the nondriven allele *A* and have higher average diploid fitness compared to ZZ males, which bear a high frequency of the driven allele *a* from their mothers.

### Transitions to environmentally determined sex

We next consider the case in which the new sex-determining allele, *m*, causes sex to be determined probabilistically or by heterogeneous environmental conditions (ESD). In particular, we assume individuals carrying allele *m* develop as females with probability *k* ∈ (0,1). In our deterministic model, this means the fraction of females in the subpopulation containing *m* is exactly *k*, even when *m* is rare (i.e., ESD does not introduce any additional variance in sex determination). We also assume that the environmental conditions that determine sex do not differentially affect the fitness of males versus females. Such correlations can favour environmental sex-determining systems by allowing each sex to be produced in the environment in which it has highest fitness; in the absence of these correlations, previous theory would predict that ESD is favoured when it produces more equal sex ratios than the ancestral system (see reviews by [1, 31, 32]).

The characteristic polynomial determining the leading eigenvalue (Eqs S1.1) does not factor for ESD (0 < *k* < 1) as it does for a neo-Y (*k* = 0) or neo-W (*k* = 1) allele. We therefore focus on weak selection here, in which case the leading eigenvalue is

$$\begin{array}{ll} \lambda_{ESD^{\prime }}^{\left(XY\right)}= & 1+\frac{{\left(1-2k\right)}^{2}}{4}\bar{p}(1-\bar{p})S_{A}{}^{2}\frac{r-R}{rR}\\ & +\frac{k({\hat{p}}^{♂}{}_{Y}-{\hat{p}}_{X}^{♂})}{2}\left[k\left(2\alpha_{\Delta }^{♂}-2\alpha_{\Delta }^{♀}+t^{♂}-t^{♀}\right)-2(1-k)S_{A}\right]+O\left(\varepsilon^{3}\right). \end{array}$$ (4)

This reduces to $\lambda_{Y^{\prime }}^{\left(XY\right)}$ when *k* = 0, and $\lambda_{W^{\prime }}^{\left(XY\right)}$ when *k* = 1. Of particular interest are ESD mutations that cause half of their carriers to develop as females and half as males (*k* = 1/2), creating equal sex ratios. The spread of such mutations is determined by

$$\lambda_{ESD^{\prime }}^{\left(XY\right)}=1+\frac{1}{2}\frac{\left(\lambda_{Y^{\prime }|R=1/2}^{\left(XY\right)}-1)+(\lambda_{W^{\prime }|R=1/2}^{\left(XY\right)}-1\right)}{2}+O\left(\varepsilon^{3}\right),$$ (5)

in which $\lambda_{Y^{\prime }|R=1/2}^{\left(XY\right)}$ and $\lambda_{W^{\prime }|R=1/2}^{\left(XY\right)}$ represent $\lambda_{Y^{\prime }}^{\left(XY\right)}$ and $\lambda_{W^{\prime }}^{\left(XY\right)}$ when evaluated at *R* = 1/2 (Eqs 2 and 3). That is, ESD with *k* = 1/2 behaves as if the **M** and **A** loci were unlinked, regardless of the actual value of *R*. This is because sex is randomised each generation in individuals bearing the *m* allele, preventing associations from building up between it and alleles at locus **A**. Eq (5) shows that the ESD mutation gets half of the fitness of a feminising mutation (neo-W) and half of the fitness of a masculinising mutation (neo-Y) but only has an effect one-half of the time (the other half of the time it produces the same sex as the ancestral system would have). As discussed above, $\lambda_{Y^{\prime }|R=1/2}^{\left(XY\right)}$ is necessarily less than or equal to 1 when selection is weak (Conclusion 3A), but $\lambda_{W^{\prime }|R=1/2}^{\left(XY\right)}$ can be greater than 1 if there is haploid selection (see Conclusion 3C). That is, with haploid selection, an allele causing ESD can invade an ancestrally XY system because it generates females that are either rare or have high fitness, in the same manner as a neo-W (likewise, ESD invades a ZW system for the same reasons that a neo-Y can).

Significantly, Eq (5) is the same whether ESD is invading an ancestrally XY or ZW system (because $\lambda_{Y^{\prime }}^{\left(XY\right)}=\lambda_{W^{\prime }}^{\left(ZW\right)}$ and $\lambda_{W^{\prime }}^{\left(XY\right)}=\lambda_{Y^{\prime }}^{\left(ZW\right)}$). Thus, focusing solely on fisherian selection to equalise the sex ratio does not fully explain GSD-to-ESD transitions. For example, when the ancestral sex-determining system is XY, the sex ratio is biased by male haploid selection. When the ancestral sex-determining system is ZW, the sex ratio is not biased. Nevertheless, ESD is equally likely to invade both XY (through $\lambda_{W^{\prime }}^{\left(XY\right)}$) and ZW (through $\lambda_{Y^{\prime }}^{\left(ZW\right)}$) systems, equalising the zygotic sex ratio in the former case but potentially transiently biasing it in the latter. In addition, we note that ESD may not invade, even if the sex ratio is initially biased (e.g., with drive in males only, *r* < 1/2, h^♀^ = *h*^♂^, and *s*^♀^ *s*^♂^ < 0, then $\lambda_{W^{\prime }}^{\left(XY\right)}<1$, see Table 3). We conclude that, as with neo-W and neo-Y mutations,

**Conclusion 5: Transitions from GSD to ESD are not straightforwardly predicted by selection to balance the zygotic sex ratio when haploid selection is present**.

## Discussion

New sex determination systems are typically expected to spread when they equalise the sex ratio and/or when they increase linkage with loci that experience sex differences in selection [33, 34]. In accordance with the latter mechanism, we find that sex differences in selection at the haploid stage can favour *cis*- or *trans*-GSD transitions that tighten sex linkage (Conclusion 3A and 3B). Contrary to this expectation, however, we find that *trans*-GSD transitions can be favoured that loosen linkage with the sex-determining locus, either when linkage is initially tight (Conclusions 1 and 2, Figs 2 and 3) or when there is haploid selection (Conclusion 3C, Figs 4 and 5). Furthermore, we show that the spread of new sex determination systems is not dominated by selection to balance the sex ratio (Conclusions 4 and 5, Fig 5).

On the one hand, sex ratio biases caused by haploid selection can facilitate *trans*-GSD transitions or transitions from GSD to ESD [42]. For instance, alleles favoured by haploid selection in males often become associated with the Y allele, which leads to an ancestral male-biased zygotic sex ratio. This male bias increases the potential for a neo-W or ESD allele to invade (Table 2), equalising the sex ratio (e.g., see Fig 5B; for related examples, see [42]). On the other hand, sex ratio selection can be overwhelmed by additional selective effects, preventing a neo-W or ESD allele from invading, even if it would balance the sex ratio (e.g., when selection also acts in opposite directions in male and female diploids, Table 3). Indeed, transitions between sex-determining systems can generate stronger sex ratio biases (e.g., Fig 5A and step 1 in [43]). In one of our key results, we find that with weak selection, there is no difference in conditions allowing XY-to-ZW and ZW-to-XY transitions (Conclusion 4), even when haploid selection always acts in the same sex (e.g., males). That is, the sex ratio bias created by male haploid selection facilitates the spread of a neo-W allele into an XY system to the same degree that male haploid selection drives the spread of a neo-Y into a ZW system with a 1:1 sex ratio (Fig 5).

Because both fisherian selection to equalise the sex ratio and the benefits of hitchhiking with driven alleles can facilitate transitions among sex chromosome systems, we predict that haploid selection should increase the lability of sex determination systems. Even in animal and plant species that have much larger and more conspicuous diploid phases than haploid phases, many loci have been shown to experience haploid selection through gamete competition and/or meiotic drive [38–41, 51–56], which can generate biased sex ratios [57–64]. In animals, a relatively small proportion of all genes are thought to be expressed and selected during competition in animal sperm [39, 65, 66]. Nevertheless, expression in the gamete is not required for haploid selection if the fitness of a gamete depends on its ability to condense DNA [67]. Furthermore, expression during gamete production often underlies systems of meiotic drive [68–70], which may be a common form of haploid selection in animals [71]. Recent studies have demonstrated that sperm competition, even within a single ejaculate, can alter haploid allele frequencies and increase offspring fitness [72, 73]. In plants, competition among gametophytes may be particularly important. It is estimated that 60%–70% of all genes are expressed in the male gametophyte, and these genes exhibit stronger signatures of selection than randomly chosen genes [74–76]. Furthermore, artificial selection pressures applied to male gametophytes are known to cause a response to selection (e.g., [77–80]).

Linking haploid expression with the evolution of sex-determination, a recent transcriptome analysis in *Rumex* shows that pollen-biased expression (relative to expression in flower buds or leaves) is enhanced among XY-linked genes, compared to autosomal genes or compared to hemizygous genes that are only linked to the X [81]. In addition, Y-linked genes are overexpressed relative to X-linked genes in pollen (but not in flower buds or leaves). This suggests that the spread of neo-Y chromosomes in this clade could have been favoured through linkage with haploid-selected genes rather than those under sexually antagonistic selection.

Frequent turnovers driven by haploid selection may help to explain the relative rarity of heteromorphic sex chromosomes in plants. If haploid selection is strong, but selective differences between male and female diploids are weak, we specifically predict that *trans*-GSD transitions are favoured more strongly than *cis*-GSD transitions, with transitions to ESD intermediate (e.g., with $\left|{\bar{s}}^{♂}-{\bar{s}}^{♀}|<<|\alpha_{\Delta }^{♂}-\alpha_{\Delta }^{♀}+t^{♂}-t^{♀}\right|$, we have $\lambda_{W^{\prime }}^{\left(XY\right)}>\lambda_{Y^{\prime }}^{\left(XY\right)}$; Eq 3). Among the relatively few dioecious clades in which multiple species have well-characterised sex chromosomes [6], *trans*-GSD transitions have been inferred in *Silene otites* [15] and in Salicaceae [16, 17]. Assuming that transitions from dioecy to hermaphroditism (equal parental investment in male and female gametes) are favoured in a similar manner to the ESD examined here (equal probability of zygotes developing as males or females), our results suggest that competition among haploid pollen could drive transitions between dioecy and hermaphroditism, which are frequent in plants [82, 83]. To further examine this link, future theory could also include inbreeding, which is an important consideration during transitions between dioecy and hermaphroditism [84]. Future empirical studies could look for evidence of haploid selection acting on former sex chromosomes in hermaphroditic species (e.g., a study such as [81] on ancestral, rather than derived, sex chromosomes).

New sex-determining alleles have previously been shown to spread when they arise in linkage with loci that experience sex differences in selection because beneficial associations build up between alleles that determine sex and alleles that are favoured in that sex [35–37, 43]. In support of this hypothesis, researchers have identified genes on recently derived sex chromosomes that might be under sexually antagonistic selection [21, 85–87]. However, we show that, if selected loci are tightly linked to the ancestral sex-determining locus, they can drive *trans*-GSD transitions that reduce sex-linkage (Conclusions 1 and 2), thus widening the range of genomic locations where selection could be driving observed *trans*-GSD transitions. In addition, we find that polymorphic sex-determining systems (X, Y, and neo-W alleles all segregating) can be maintained when a selected locus is tightly linked to the ancestral sex-determining system (e.g., S9B and S9C Fig), which is not possible with loose linkage [36]. This pair of conclusions applies in cases with or without haploid selection.

Our tight linkage result—in particular, the prediction that invasion can lead to polymorphic sex determination—is consistent with empirical data from species in which new feminising mutations are found segregating with ancestral XY loci. For example, in the platyfish (*X*. *maculatus*), X,Y, and W alleles segregate at one locus (or two closely linked loci) near potentially sexually antagonistic genes for pigmentation and sexual maturity [44, 88–90]. Furthermore, several rodent species maintain feminising alleles along with the ancestral X and Y sex determination alleles (reviewed in [91]). In nine *Akadon* rodent species, it appears that male-determining *sry* expression is suppressed by an autosomal feminising allele (a neo-W allele), creating XY females [92, 93]. XY females have increased fitness relative to XX females [94]. However, it is not yet clear whether loci linked to the feminising factor or the ancestral Y cause this effect. Most convincingly, in *Mus minutoides*, females can have XX, XX*, or X * Y genotypes [95]. Previous theory would predict that the dominant X* chromosome (potentially an autosome that has fused with the sex chromosome) harbours female-beneficial alleles, driving its spread. However, XX and XX* females have similar fitness, whereas X * Y female fitness is enhanced [96–98]. Although Y-linkage of female-beneficial alleles is counterintuitive, our model suggests that it can be stably maintained when linkage is initially tight between the sex-determining region and the selected locus, subsequently favouring new feminising mutations, which would be a parsimonious explanation for the spread of feminising alleles in this case.

Our models assume that sex-determining alleles do not experience direct selection except via their associations with sex and selected alleles. However, in some cases, there may be significant degeneration around the sex-limited allele (Y or W) in the ancestral sex-determining region because recessive deleterious mutations and/or deletions accumulate in the surrounding nonrecombining regions [99–102]. During *trans*-GSD transitions, but not *cis*-GSD transitions, any recessive deleterious alleles linked to the Y or W are revealed to selection in YY or WW individuals [4]. This phenomenon was studied by van Doorn and Kirkpatrick [36], who found that degeneration can prevent fixation of a neo-W or a neo-Y allele, leading to a mixed sex-determining system in which the ancestral and new sex-determining loci are both segregating. However, they noted that very rare recombination events around the ancestral sex-determining locus can allow the completion of *trans*-GSD transitions. Degeneration around the Y or W could explain why *trans*-GSD transitions are not observed to be much more common than *cis*-GSD transitions despite the fact that our models demonstrate that they are favoured under a wider range of conditions, especially with haploid selection. For example, there are a dozen sex chromosome configurations among dipteran species but only one transition between male and female heterogamety [9], but Y degeneration or absence is also very common among Diptera [9].

In this study, we have only considered new sex-determining alleles of large effect. However, we expect similar selective forces to act on masculinising and feminising alleles of weaker effect. For example, small-effect masculinising and feminising alleles within a threshold model of sex determination can be favoured when linked to loci that experience sexually antagonistic selection [37]. These results echo those for large-effect neo-Y and neo-W alleles [35, 36]. It should be noted, however, that the dynamics of sex-determining alleles with very weak effect will be influenced by genetic drift, which itself has been shown to bias transitions towards epistatically dominant sex-determining systems when there is no direct selection [103].

## Conclusion

We have shown that tight sex linkage and haploid selection can drive previously unexpected transitions between sex-determining systems. In particular, both can select for new sex-determining loci that are more loosely linked to loci under selection (Conclusions 2 and 3C). In addition, haploid selection can cause transitions in GSD analogous to those caused by purely sexually antagonistic selection, eliminating the need for differences in selection between male and female diploids (Conclusion 3A, 3B, and 3C). We conclude that haploid selection should be considered as a pivotal factor driving transitions between sex-determining systems. Further, transitions involving haploid selection can eliminate or generate sex ratio biases; to leading order, selection to balance the sex ratio and the benefits of hitchhiking with haploid-selected alleles, leading to a biased sex ratio, are of equal magnitude (Conclusions 4 and 5). Overall, our results suggest several novel scenarios under which new sex-determining systems are favoured, which could help to explain why the evolution of sex-determining systems is so dynamic.

## Supporting information

S1 File Supplementary *Mathematica* file. This file can be used to rederive our results and generate figures.
(NB) Click here for additional data file.

S2 File Supplementary *Mathematica* file in CDF form. This file can be used to rederive our results and generate figures with a free online viewer (www.wolfram.com/cdf-player/). CDF, computable document format.
(CDF)

S3 File Supplementary *Mathematica* file in PDF form. This file can be used to see how we have derived our results and have generated figures with any PDF viewer. PDF, portable document format.
(PDF)

S1 Table Substitutions for different loci orders, assuming no interference. (PDF)

S2 Table Mean fitnesses and zygotic sex ratio in the resident population (*M* fixed, XY sex determination). (PDF)

S1 Text Recursion equations and complete model description. (PDF)

S2 Text Equilibria and stability conditions when *M* allele is fixed. (PDF)

S3 Text Invasion conditions for the *m* allele. (PDF)

S1 FigWith overdominance, loci near the ancestral sex-determining locus (*r* ≈ 0) can favour neo-W alleles that are less tightly linked (*R* > *r*).In panels A and B, the *a* allele is favoured in females ($w_{aa}^{♀}=1.05$, 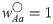, $w_{AA}^{♀}=0.85$), and selection in males is overdominant ($w_{aa}^{♂}=w_{AA}^{♂}=0.75$). In panels C and D, selection in males and females is overdominant ($w_{aa}^{♀}=w_{AA}^{♀}=0.6$, $w_{aa}^{♂}=0.5$, $w_{AA}^{♂}=0.7$, 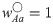). There is no haploid selection 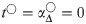. These parameters are marked by daggers in Fig 2B and 2C, which show that neo-W invasion is expected for any *R* ($\Lambda_{W^{\prime }A}^{\left(XY\right)}$, $\Lambda_{W^{\prime }a}^{\left(XY\right)}>1$) if the *a* allele is nearly fixed on the Y (black lines in this figure; not stable for *r* ≫ 0). Equilibria in which the *A* allele is more common among Y-bearing male gametes can also be stable and allow neo-W invasion for these parameters (blue lines).(TIF)

S2 Fig Following invasion by a neo-W allele, there can be a complete transition to a new sex-determining system, maintenance of both ancestral XY and neo-ZW sex-determining systems, or loss of the new sex-determining allele. Here, we plot the frequency of the neo-W allele among female gametes. Panels A, C, and D show cases in which a steady state is reached with the neo-W at a frequency below 0.5, in which case ancestral X and Y alleles also both segregate. In all cases, we assume that the *a* allele is initially more common than the *A* allele on the Y background (Y-*a* is fixed when *r* = 0). When *r* > 0 (panels B and D), Y-*A* haplotypes created by recombination can become more common than Y-*a* haplotypes as the neo-W spreads. In B, this leads to loss of the neo-W, and the system goes to an equilibrium with X-*a* and Y-*A* haplotypes fixed (equilibrium *A*′), such that all females have the high fitness genotype *aa*, and all males are *Aa*. For the parameters in B, neo-W alleles have negative invasion fitness when the Y-*A* haplotype is ancestrally more common than Y-*a* (compare blue to black curves in S1A Fig and S2B Fig near the ancestral sex-determining locus). In contrast, the neo-W is not lost in panel D, as it is favoured regardless of whether Y-*A* or Y-*a* haplotypes predominate (again, compare blue to black curves in S1C and S1D Fig).
(TIF)

S3 FigWhen there is sexually antagonistic selection and haploid selection, a neo-W allele may invade for any *R*.Panel A shows that the invasion fitness of a neo-W is positive, even when *r* < *R* (unshaded region). In panel B, we vary the recombination rate between the neo-W and the selected locus (*R*) for a fixed recombination rate between the ancestral sex-determining locus and the selected locus (*r* = 0.005). Coloured markers show recombination rates for which the temporal dynamics of neo-W invasion are plotted in panel C (black *R* = 0.001, red *R* = 0.02, blue *R* = 0.1, green *R* = 0.5). The diploid selection parameters used in this plot are the same as in Fig 3. There is also meiotic drive in males favouring *a* ($\alpha_{\Delta }^{♂}=-0.08$); this full set of parameters is marked by an asterisk in S4A Fig. When *R* = 0.5 (green curve), the neo-W does not reach fixation, and X, Y, Z, and W alleles are all maintained in the population; see S9C Fig.(TIF)

S4 FigParameters for which neo-W-*A* and neo-W-*a* haplotypes spread when there is male meiotic drive at a locus that is tightly linked to the ancestral XY locus (*r* ≈ 0).This figure is equivalent to Fig 2 but with meiotic drive in males. In panels A–C, meiotic drive in males favours the *a* allele ($\alpha_{\Delta }^{♂}=-0.16$), creating male-biased sex ratios and generally increasing $\Lambda_{W^{\prime }A}^{\left(XY\right)}$ and $\Lambda_{W^{\prime }a}^{\left(XY\right)}$. By contrast, $\Lambda_{W^{\prime }A}^{\left(XY\right)}$ and $\Lambda_{W^{\prime }a}^{\left(XY\right)}$ tend to be reduced when meiotic drive in males favours the *A* allele ($\alpha_{\Delta }^{♂}=0.16$), panels D–F.(TIF)

S5 FigParameters for which neo-W-*A* and neo-W-*a* haplotypes spread when there is male gametic competition at a locus that is tightly linked to the ancestral XY locus (*r* ≈ 0).This figure is equivalent to Fig 2 but with gametic competition in males. The *a* allele is favoured during male gametic competition in panels A–C ($w_{a}^{♂}=1.16$, $w_{A}^{♂}=1$), which creates male-biased sex ratios and increases $\Lambda_{W^{\prime }A}^{\left(XY\right)}$ and $\Lambda_{W^{\prime }a}^{\left(XY\right)}$. By contrast, $\Lambda_{W^{\prime }A}^{\left(XY\right)}$ and $\Lambda_{W^{\prime }a}^{\left(XY\right)}$ tend to be reduced when the *A* allele is favoured during male gametic competition, panels D–F. Compared to the meiotic drive parameters in S4 Fig, the effect of these male gametic competition parameters on the sex ratio is smaller. For example, in S4A–S4C Fig, the ancestral sex ratio is *α*^♂^ = 0.58 at equilibrium (B), and in panels A–C of this plot, the ancestral sex ratio is $w_{a}^{♂}/(w_{A}^{♂}+w_{a}^{♂})=0.537$ at equilibrium (B).(TIF)

S6 FigParameters for which neo-W-*A* and neo-W-*a* haplotypes spread when there is female meiotic drive at a locus that is tightly linked to the ancestral XY locus (*r* ≈ 0).This figure is equivalent to Fig 2 but with meiotic drive in females. The *a* allele is favoured by meiotic drive in females in panels A–C ($\alpha_{\Delta }^{♀}=-0.16$), which increases $\Lambda_{W^{\prime }a}^{\left(XY\right)}$ and decreases $\Lambda_{W^{\prime }A}^{\left(XY\right)}$. Female meiotic drive in favour of the *A* allele (panels D–F, $\alpha_{\Delta }^{♂}=-0.16$) has the opposite effect.(TIF)

S7 FigParameters for which neo-W-*A* and neo-W-*a* haplotypes spread when there is female gametic competition at a locus that is tightly linked to the ancestral XY locus (*r* ≈ 0).This figure is equivalent to Fig 2 but with gametic competition in females. The *a* allele is favoured during female gametic competition in females in panels A–C ($w_{a}^{♀}=1.16$, $w_{A}^{♀}=1$), which increases $\Lambda_{W^{\prime }a}^{\left(XY\right)}$ and decreases $\Lambda_{W^{\prime }A}^{\left(XY\right)}$. The *A* allele is favoured during gametic competition in panels D–F ($w_{a}^{♀}=1$, $w_{A}^{♀}=1.16$), giving the opposite effect on $\Lambda_{W^{\prime }a}^{\left(XY\right)}$ and $\Lambda_{W^{\prime }A}^{\left(XY\right)}$.(TIF)

S8 FigPloidally antagonistic selection can drive the spread of neo-W alleles.A–D show when each of the neo-W haplotypes invades an internally stable equilibrium with *a* fixed on the Y (found by setting *r* = 0). The y-axis shows directional selection in diploids of both sexes, *s*^♀^ = *s*^♂^, and the x-axes show sex-limited drive, 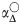, or haploid competition, 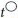. The top-left and bottom-right quadrants therefore imply ploidally antagonistic selection (and these are the only places where neo-W haplotypes can invade). Dominance is equal in both sexes, *h*^♀^ = *h*^♂^ = 3/4. E–F show the temporal dynamics of neo-W frequency in females, with parameters given by the asterisks in the corresponding A–D plot, with *r* = 1/200, for four different *R*. Black *R* = 1/1000, Red *R* = 2/100, Blue *R* = 1/10, Green *R* = 1/2.(TIF)

S9 Fig Pseudofixation of neo-W or maintenance of multiple sex-determining alleles. The curves show the frequencies of the neo-W (red), ancestral Y (blue), and *A* allele (black) among female gametes (solid curves) and among male gametes (dashed curves). In panel A, there is a complete transition from XY sex determination (XX-ZZ females and XY-ZZ males, labeling allele *M* as Z and the new allele *m* as W) to ZW sex determination (YY-ZW females and YY-ZZ males). In panels B and C, a polymorphism is maintained at both the ancestral XY locus and the new ZW locus, such that there are males with genotypes XY-ZZ and YY-ZZ and females with genotypes XX-ZZ, XX-ZW, XY-ZW, and YY-ZW. In panel A, selection is ploidally antagonistic with drive in males (parameters as in the green curve in Fig 5B). In panel B, there is overdominance in both sexes and no haploid selection (parameters as in the green curve in S2C Fig). In panel C, there is sexually antagonistic selection in diploids with drive in males (parameters as in the green curve in S4C Fig). In all cases, the initial equilibrium frequency has *a* near fixation on the Y.
(TIF)

## Abbreviations

ESD: environmental sex determination
GSD: genetic sex determination
