# Supplementary material for: Haploid selection, sex ratio bias, and transitions between sex-determining systems
Source: PLoS Biol. 2018 Jun 25;16(6):e2005609. doi: 10.1371/journal.pbio.2005609 (PMC6042799; doi:10.1371/journal.pbio.2005609)
Supplement: S3 File — This file can be used to see how we have derived our results and have generated figures with any PDF viewer. PDF, portable document format. (PDF) [file pbio.2005609.s003.pdf]

Gametic selection, sex-ratio bias, and transitions between sex-determining systems

Michael F Scott, Matthew M Osmond, Sarah P Otto

Notes:
1) The notation in this notebook differs slightly from the text: here we do not use male and female symbols or sub- and super-scripts.
2) Throughout we mainly refer to an ancestral XY sex-determination system. By consistently flipping male and female labels, this is equivalent to an ancestral ZW system (noting that the heterogametic sex described in this notebook as XY would refer to heterogametic ZW females).

General

Assumptions used to simplify the results:

```
simpcond = Reduce[{0 < MAA, 0 < MAa, 0 < Maa, 0 < FAA, 0 < FAa, 0 < Faa, 0 < wAf,
    0 < waf, 0 < wAm, 0 < wam, 0 < αm < 1, 0 < αf < 1, 0 ≤ Rm ≤ 1/2, 0 ≤ Rf ≤ 1/2}];
```

Recursion equations

Haploid competition

We start with haploid competition in each sex separately (e.g., pollen competition for eggs). We assume that the allele at an autosomal locus **A** determines competitive ability, with relative fitnesses wAf and waf in females and wAm and wam in males (in the text this is, e.g., $w_A{}^{\text{female}}$). Let the frequency of each gamete from each sex before selection be denoted by four letters, the first being the allele at the ancestral SDR (X or Y), the second being the allele at the selected locus (A or a), the third being the allele at the novel SDR (M or m), and the fourth indicating which sex the gamete came from (m or f). E.g., XAMf is the frequency of the X-A-M genotype among eggs (in the text this is $x_1{}^{\text{female}}$). Let the frequencies after haploid selection end in an s, e.g., XAMfs. We then have

```
(*mean fitness of female gametes*)
wbarHapFemale =
  (wAf XAMf + waf XaMf + wAf XAmf + waf Xamf) + (wAf YAMf + waf YaMf + wAf YAmf + waf Yamf);

(*non-mutant eggs*)
XAMfs = wAf XAMf / wbarHapFemale;
XaMfs = waf XaMf / wbarHapFemale;
YAMfs = wAf YAMf / wbarHapFemale;
YaMfs = waf YaMf / wbarHapFemale;

(*mutant eggs*)
XAmfs = wAf XAmf / wbarHapFemale;
Xamfs = waf Xamf / wbarHapFemale;
YAmfs = wAf YAmf / wbarHapFemale;
Yamfs = waf Yamf / wbarHapFemale;

(*mean fitness of male gametes*)
wbarHapMale =
  (wAm XAMm + wam XaMm + wAm XAmm + wam Xamm) + (wAm YAMm + wam YaMm + wAm YAmm + wam Yamm);

(*non-mutant male gametes*)
XAMms = wAm XAMm / wbarHapMale;
XaMms = wam XaMm / wbarHapMale;
YAMms =  wAm YAMm / wbarHapMale;
YaMms =  wam YaMm / wbarHapMale;

(*mutant male gametes*)
XAmms = wAm XAmm / wbarHapMale;
Xamms = wam Xamm / wbarHapMale;
YAmms =  wAm YAmm / wbarHapMale;
Yamms =  wam Yamm / wbarHapMale;
```

Check that frequencies sum to one in each sex after haploid selection:

```
1 == XAMms + XaMms + YAMms + YaMms + XAmms + Xamms + YAmms + Yamms // Factor
(*male gametes*)
1 == XAMfs + XaMfs + XAmfs + Xamfs + YAMfs + YaMfs + YAmfs + Yamfs // Factor (*eggs*)
```

```
True
```

```
True
```

Random mating

Let the probability that a zygote develops into a female be denoted with k followed by four letters: the first two indicating the ancestral SDR genotype, and the second two denoting the novel SDR genotype. For example, kXYMm is the probabiliy that a zygote with XY at the ancestral SDR and Mm at the novel SDR develops into a female. Let the frequency of zygotes be denoted by the two gamete genotypes followed by the sex, e.g., XAMYamfemale is the frequency of zygotes that are female and composed of X-A-M and Y-a-M gametes (it does not matter which came from which parent, i.e., XAM could come from the mother or father). Then, after random mating the frequencies of the diploid genotypes are

```
(*MM Homozygotes*)
(*XM-XM females*)
XAMXAMfemale = kXXMM (XAMfs XAMms);
```

```
XAMXaMfemale = kXXMM (XAMfs XaMms + XaMfs XAMms );
XaMXaMfemale = kXXMM (XaMfs XaMms);
(*XM-XM males*)
XAMXAMmale = (1 - kXXMM) (XAMfs XAMms);
XAMXaMmale = (1 - kXXMM) (XAMfs XaMms + XaMfs XAMms );
XaMXaMmale = (1 - kXXMM) (XaMfs XaMms);

(*XM-YM females*)
XAMYAMfemale = kXYMM (XAMfs YAMms + YAMfs XAMms);
XAMYaMfemale = kXYMM (XAMfs YaMms + YaMfs XAMms);
XaMYAMfemale = kXYMM (XaMfs YAMms + YAMfs XaMms);
XaMYaMfemale = kXYMM (XaMfs YaMms + YaMfs XaMms);
(*XM-YM males*)
XAMYAMmale = (1 - kXYMM) (XAMfs YAMms + YAMfs XAMms);
XAMYaMmale = (1 - kXYMM) (XAMfs YaMms + YaMfs XAMms);
XaMYAMmale = (1 - kXYMM) (XaMfs YAMms + YAMfs XaMms);
XaMYaMmale = (1 - kXYMM) (XaMfs YaMms + YaMfs XaMms);

(*YM-YM females*)
YAMYAMfemale = kYYMM (YAMfs YAMms);
YAMYaMfemale = kYYMM (YAMfs YaMms + YaMfs YAMms);
YaMYaMfemale = kYYMM (YaMfs YaMms);
(*YM-YM males*)
YAMYAMmale = (1 - kYYMM) (YAMfs YAMms);
YAMYaMmale = (1 - kYYMM) (YAMfs YaMms + YaMfs YAMms);
YaMYaMmale = (1 - kYYMM) (YaMfs YaMms);

(*Mm heterozygotes*)
(*XM-Xm females*)
XAmXAMfemale = kXXMm (XAmfs XAMms + XAMfs XAmms);
XAmXaMfemale = kXXMm (XAmfs XaMms + XaMfs XAmms );
XAMXamfemale = kXXMm (XAMfs Xamms + Xamfs XAMms);
XamXaMfemale = kXXMm (Xamfs XaMms + XaMfs Xamms);
(*XM-Xm males*)
XAmXAMmale = (1 - kXXMm) (XAmfs XAMms + XAMfs XAmms);
XAmXaMmale = (1 - kXXMm) (XAmfs XaMms + XaMfs XAmms );
XAMXammale = (1 - kXXMm) (XAMfs Xamms + Xamfs XAMms);
XamXaMmale = (1 - kXXMm) (Xamfs XaMms + XaMfs Xamms);

(*Xm-YM females*)
XAmYAMfemale = kXYMm (XAmfs YAMms + YAMfs XAmms);
XAmYaMfemale = kXYMm (XAmfs YaMms + YaMfs XAmms);
XamYAMfemale = kXYMm (Xamfs YAMms + YAMfs Xamms);
XamYaMfemale = kXYMm (Xamfs YaMms + YaMfs Xamms);
(*Xm-YM males*)
XAmYAMmale = (1 - kXYMm) (XAmfs YAMms + YAMfs XAmms);
XAmYaMmale = (1 - kXYMm) (XAmfs YaMms + YaMfs XAmms);
XamYAMmale = (1 - kXYMm) (Xamfs YAMms + YAMfs Xamms);
XamYaMmale = (1 - kXYMm) (Xamfs YaMms + YaMfs Xamms);

(*XM-Ym females*)
XAMYAmfemale = kXYMm (XAMfs YAmms + YAmfs XAMms);
XAMYamfemale = kXYMm (XAMfs Yamms + Yamfs XAMms );
XaMYAmfemale = kXYMm (XaMfs YAmms + YAmfs XaMms );
XaMYamfemale = kXYMm (XaMfs Yamms + Yamfs XaMms);
```

```
(*XM-Ym females*)
XAMYAmmale = (1 - kXYMm) (XAMfs YAmms + YAmfs XAMms);
XAMYammale = (1 - kXYMm) (XAMfs Yamms + Yamfs XAMms );
XaMYAmmale = (1 - kXYMm) (XaMfs YAmms + YAmfs XaMms );
XaMYammale = (1 - kXYMm) (XaMfs Yamms + Yamfs XaMms);

(*Ym-YM females*)
YAmYAMfemale = kYYMm (YAmfs YAMms + YAMfs YAmms);
YAmYaMfemale = kYYMm (YAmfs YaMms + YaMfs YAmms);
YamYAMfemale = kYYMm (Yamfs YAMms + YAMfs Yamms);
YamYaMfemale = kYYMm (Yamfs YaMms + YaMfs Yamms);
(*Ym-YM males*)
YAmYAMmale = (1 - kYYMm) (YAmfs YAMms + YAMfs YAmms);
YAmYaMmale = (1 - kYYMm) (YAmfs YaMms + YaMfs YAmms);
YamYAMmale = (1 - kYYMm) (Yamfs YAMms + YAMfs Yamms);
YamYaMmale = (1 - kYYMm) (Yamfs YaMms + YaMfs Yamms);

(*mm heterozygotes*)
(*Xm-Xm females*)
XAmXAmfemale = kXXmm (XAmfs XAmms);
XAmXamfemale = kXXmm (XAmfs Xamms + Xamfs XAmms );
XamXamfemale = kXXmm (Xamfs Xamms);
(*Xm-Xm males*)
XAmXAmmale = (1 - kXXmm) (XAmfs XAmms);
XAmXammale = (1 - kXXmm) (XAmfs Xamms + Xamfs XAmms );
XamXammale = (1 - kXXmm) (Xamfs Xamms);

(*Xm-Ym females*)
XAmYAmfemale = kXYmm (XAmfs YAmms + YAmfs XAmms );
XAmYamfemale = kXYmm (XAmfs Yamms + Yamfs XAmms );
XamYAmfemale = kXYmm (Xamfs YAmms + YAmfs Xamms );
XamYamfemale = kXYmm (Xamfs Yamms + Yamfs Xamms);
(*Xm-Ym males*)
XAmYAmmale = (1 - kXYmm) (XAmfs YAmms + YAmfs XAmms );
XAmYammale = (1 - kXYmm) (XAmfs Yamms + Yamfs XAmms );
XamYAmmale = (1 - kXYmm) (Xamfs YAmms + YAmfs Xamms );
XamYammale = (1 - kXYmm) (Xamfs Yamms + Yamfs Xamms);

(*Ym-Ym females*)
YAmYAmfemale = kYYmm (YAmfs YAmms);
YAmYamfemale = kYYmm (YAmfs Yamms + Yamfs YAmms);
YamYamfemale = kYYmm (Yamfs Yamms);
(*Ym-Ym males*)
YAmYAmmale = (1 - kYYmm) (YAmfs YAmms);
YAmYammale = (1 - kYYmm) (YAmfs Yamms + Yamfs YAmms);
YamYammale = (1 - kYYmm) (Yamfs Yamms);
```

Check that they sum to one

```
1 == XAMXAMfemale + XAMXaMfemale + XaMXaMfemale +
    XAMXAMmale + XAMXaMmale + XaMXaMmale +
    XAMYAMmale + XAMYaMmale + XaMYAMmale + XaMYaMmale +
    XAMYAMfemale + XAMYaMfemale + XaMYAMfemale + XaMYaMfemale +
    YAMYAMmale + YAMYaMmale + YaMYaMmale +
    YAMYAMfemale + YAMYaMfemale + YaMYaMfemale +
    XAmXAMfemale + XAmXaMfemale + XAMXamfemale + XamXaMfemale +
    XAmXAMmale + XAmXaMmale + XAMXammale + XamXaMmale +
    XAmYAMfemale + XAmYaMfemale + XamYAMfemale + XamYaMfemale +
    XAmYAMmale + XAmYaMmale + XamYAMmale + XamYaMmale +
    XAMYAmfemale + XAMYamfemale + XaMYAmfemale + XaMYamfemale +
    XAMYAmmale + XAMYammale + XaMYAmmale + XaMYammale +
    YAmYAMfemale + YAmYaMfemale + YamYAMfemale + YamYaMfemale +
    YAmYAMmale + YAmYaMmale + YamYAMmale + YamYaMmale +
    XAmXAmfemale + XAmXamfemale + XamXamfemale +
    XAmXAmmale + XAmXammale + XamXammale +
    XAmYAmfemale + XAmYamfemale + XamYAmfemale + XamYamfemale +
    XAmYAmmale + XAmYammale + XamYAmmale + XamYammale +
    YAmYAmfemale + YAmYamfemale + YamYamfemale +
    YAmYAmmale + YAmYammale + YamYammale // Factor
```

```
True
```

Diploid selection

We next model selection in diploids, which can differ among the sexes. Let FAA, FAa, and Faa be the relative fitnesses of females homozygous for allele A, heterozygous, or homozygous for allele a at the autosomal locus. Similarly we use MAA, MAa, and Maa in males. Frequencies after selection in diploids are indicated by appending an s to the name

```
(*mean male fitness*)
wbarM =
  MAA XAMXAMmale + MAa XAMXaMmale + Maa XaMXaMmale +
   MAA XAMYAMmale + MAa (XAMYaMmale + XaMYAMmale) + Maa XaMYaMmale +
   MAA YAMYAMmale + MAa YAMYaMmale + Maa YaMYaMmale +
   MAA XAmXAMmale + MAa (XAmXaMmale + XAMXammale) + Maa XamXaMmale +
   MAA XAmYAMmale + MAa (XAmYaMmale + XamYAMmale) + Maa XamYaMmale +
   MAA XAMYAmmale + MAa (XAMYammale + XaMYAmmale) + Maa XaMYammale +
   MAA YAmYAMmale + MAa (YAmYaMmale + YamYAMmale) + Maa YamYaMmale +
   MAA XAmXAmmale + MAa XAmXammale + Maa XamXammale +
   MAA XAmYAmmale + MAa (XAmYammale + XamYAmmale) + Maa XamYammale +
   MAA YAmYAmmale + MAa YAmYammale + Maa YamYammale;

XAMXAMmales = MAA XAMXAMmale / wbarM;
XAMXaMmales = MAa XAMXaMmale / wbarM;
XaMXaMmales = Maa XaMXaMmale / wbarM;

XAMYAMmales = MAA XAMYAMmale / wbarM;
XAMYaMmales = MAa XAMYaMmale / wbarM;
XaMYAMmales = MAa XaMYAMmale / wbarM;
XaMYaMmales = Maa XaMYaMmale / wbarM;

YAMYAMmales = MAA YAMYAMmale / wbarM;
YAMYaMmales = MAa YAMYaMmale / wbarM;
```

```
YaMYaMmales = Maa YaMYaMmale / wbarM;

XAmXAMmales = MAA XAmXAMmale / wbarM;
XAmXaMmales = MAa XAmXaMmale / wbarM;
XAMXammales = MAa XAMXammale / wbarM;
XamXaMmales = Maa XamXaMmale / wbarM;

XAmYAMmales = MAA XAmYAMmale / wbarM;
XAmYaMmales = MAa XAmYaMmale / wbarM;
XamYAMmales = MAa XamYAMmale / wbarM;
XamYaMmales = Maa XamYaMmale / wbarM;

XAMYAmmales = MAA XAMYAmmale / wbarM;
XAMYammales = MAa XAMYammale / wbarM;
XaMYAmmales = MAa XaMYAmmale / wbarM;
XaMYammales = Maa XaMYammale / wbarM;

YAmYAMmales = MAA YAmYAMmale / wbarM;
YAmYaMmales = MAa YAmYaMmale / wbarM;
YamYAMmales = MAa YamYAMmale / wbarM;
YamYaMmales = Maa YamYaMmale / wbarM;

XAmXAmmales = MAA XAmXAmmale / wbarM;
XAmXammales = MAa XAmXammale / wbarM;
XamXammales = Maa XamXammale / wbarM;

XAmYAmmales = MAA XAmYAmmale / wbarM;
XAmYammales = MAa XAmYammale / wbarM;
XamYAmmales = MAa XamYAmmale / wbarM;
XamYammales = Maa XamYammale / wbarM;

YAmYAmmales = MAA YAmYAmmale / wbarM;
YAmYammales = MAa YAmYammale / wbarM;
YamYammales = Maa YamYammale / wbarM;
```

Frequency of males after diploid selection should sum to 1

```
1 == XAMXAMmales + XAMXaMmales + XaMXaMmales +
   XAMYAMmales + XAMYaMmales + XaMYAMmales + XaMYaMmales +
   YAMYAMmales + YAMYaMmales + YaMYaMmales +
   XAmXAMmales + XAmXaMmales + XAMXammales + XamXaMmales +
   XAmYAMmales + XAmYaMmales + XamYAMmales + XamYaMmales +
   XAMYAmmales + XAMYammales + XaMYAmmales + XaMYammales +
   YAmYAMmales + YAmYaMmales + YamYAMmales + YamYaMmales +
   XAmXAmmales + XAmXammales + XamXammales +
   XAmYAmmales + XAmYammales + XamYAmmales + XamYammales +
   YAmYAmmales + YAmYammales + YamYammales // Factor
```

```
True
```

```
(*mean female fitness*)
wbarF = FAA XAMXAMfemale + FAa XAMXaMfemale + Faa XaMXaMfemale +
   FAA XAMYAMfemale + FAa (XAMYaMfemale + XaMYAMfemale) + Faa XaMYaMfemale +
   FAA YAMYAMfemale + FAa YAMYaMfemale + Faa YaMYaMfemale +
   FAA XAmXAMfemale + FAa XAmXaMfemale + FAa XAMXamfemale + Faa XamXaMfemale +
   FAA XAmYAMfemale + FAa XAmYaMfemale + FAa XamYAMfemale + Faa XamYaMfemale +
```

```
    FAA XAMYAmfemale + FAa XAMYamfemale + FAa XaMYAmfemale + Faa XaMYamfemale +
    FAA YAmYAMfemale + FAa YAmYaMfemale + FAa YamYAMfemale + Faa YamYaMfemale +
    FAA XAmXAmfemale + FAa XAmXamfemale + Faa XamXamfemale +
    FAA XAmYAmfemale + FAa XAmYamfemale + FAa XamYAmfemale + Faa XamYamfemale +
    FAA YAmYAmfemale + FAa YAmYamfemale + Faa YamYamfemale;

XAMXAMfemales = FAA XAMXAMfemale / wbarF;
XAMXaMfemales = FAa XAMXaMfemale / wbarF;
XaMXaMfemales = Faa XaMXaMfemale / wbarF;

XAMYAMfemales = FAA XAMYAMfemale / wbarF;
XAMYaMfemales = FAa XAMYaMfemale / wbarF;
XaMYAMfemales = FAa XaMYAMfemale / wbarF;
XaMYaMfemales = Faa XaMYaMfemale / wbarF;

YAMYAMfemales = FAA YAMYAMfemale / wbarF;
YAMYaMfemales = FAa YAMYaMfemale / wbarF;
YaMYaMfemales = Faa YaMYaMfemale / wbarF;

XAmXAMfemales = FAA XAmXAMfemale / wbarF;
XAmXaMfemales = FAa XAmXaMfemale / wbarF;
XAMXamfemales = FAa XAMXamfemale / wbarF;
XamXaMfemales = Faa XamXaMfemale / wbarF;

XAmYAMfemales = FAA XAmYAMfemale / wbarF;
XAmYaMfemales = FAa XAmYaMfemale / wbarF;
XamYAMfemales = FAa XamYAMfemale / wbarF;
XamYaMfemales = Faa XamYaMfemale / wbarF;

XAMYAmfemales = FAA XAMYAmfemale / wbarF;
XAMYamfemales = FAa XAMYamfemale / wbarF;
XaMYAmfemales = FAa XaMYAmfemale / wbarF;
XaMYamfemales = Faa XaMYamfemale / wbarF;

YAmYAMfemales = FAA YAmYAMfemale / wbarF;
YAmYaMfemales = FAa YAmYaMfemale / wbarF;
YamYAMfemales = FAa YamYAMfemale / wbarF;
YamYaMfemales = Faa YamYaMfemale / wbarF;

XAmXAmfemales = FAA XAmXAmfemale / wbarF;
XAmXamfemales = FAa XAmXamfemale / wbarF;
XamXamfemales = Faa XamXamfemale / wbarF;

XAmYAmfemales = FAA XAmYAmfemale / wbarF;
XAmYamfemales = FAa XAmYamfemale / wbarF;
XamYAmfemales = FAa XamYAmfemale / wbarF;
XamYamfemales = Faa XamYamfemale / wbarF;

YAmYAmfemales = FAA YAmYAmfemale / wbarF;
YAmYamfemales = FAa YAmYamfemale / wbarF;
YamYamfemales = Faa YamYamfemale / wbarF;
```

Frequency of females after diplod selection should sum to 1

```
1 == XAMXAMfemales + XAMXaMfemales + XaMXaMfemales +
    XAMYAMfemales + XAMYaMfemales + XaMYAMfemales + XaMYaMfemales +
    YAMYAMfemales + YAMYaMfemales + YaMYaMfemales +
    XAmXAMfemales + XAmXaMfemales + XAMXamfemales + XamXaMfemales +
    XAmYAMfemales + XAmYaMfemales + XamYAMfemales + XamYaMfemales +
    XAMYAmfemales + XAMYamfemales + XaMYAmfemales + XaMYamfemales +
    YAmYAMfemales + YAmYaMfemales + YamYAMfemales + YamYaMfemales +
    XAmXAmfemales + XAmXamfemales + XamXamfemales +
    XAmYAmfemales + XAmYamfemales + XamYAmfemales + XamYamfemales +
    YAmYAmfemales + YAmYamfemales + YamYamfemales // Factor
```

```
True
```

Meiosis

Translate zygote frequencies into notation in the main text to give recursion equations:

a) first two letters refer to the ancestral SDR locus genotype (now lower case, e.g., xy)
b) then two numbers ij give A and M locus genotype where 1=AM, 2=aM, 3=Am, 4=am
c) then m or f indicate male or female
d) then s indicates that these are frequencies after selection

e.g., xy41ms is the frequency of zygotes that are male and composed of gametes X-a-m and Y-A-M. When i and j are interchangable (in YY and XX individuals) the lowest number is given first.

```
xx11ms = XAMXAMmales;
xx12ms = XAMXaMmales;
xx22ms = XaMXaMmales;

xx13ms = XAmXAMmales;
xx23ms = XAmXaMmales;
xx14ms = XAMXammales;
xx24ms = XamXaMmales;

xx33ms = XAmXAmmales;
xx34ms = XAmXammales;
xx44ms = XamXammales;

xy11ms = XAMYAMmales;
xy12ms = XAMYaMmales;
xy21ms = XaMYAMmales;
xy22ms = XaMYaMmales;

xy31ms = XAmYAMmales;
xy32ms = XAmYaMmales;
xy41ms = XamYAMmales;
xy42ms = XamYaMmales;

xy13ms = XAMYAmmales;
xy14ms = XAMYammales;
xy23ms = XaMYAmmales;
xy24ms = XaMYammales;

xy33ms = XAmYAmmales;
xy34ms = XAmYammales;
xy43ms = XamYAmmales;
xy44ms = XamYammales;

yy11ms = YAMYAMmales;
yy12ms = YAMYaMmales;
yy22ms = YaMYaMmales;

yy13ms = YAmYAMmales;
yy23ms = YAmYaMmales;
yy14ms = YamYAMmales;
yy24ms = YamYaMmales;

yy33ms = YAmYAmmales;
yy34ms = YAmYammales;
yy44ms = YamYammales;
```

```
xx11fs = XAMXAMfemales;
xx12fs = XAMXaMfemales;
xx22fs = XaMXaMfemales;

xx13fs = XAmXAMfemales;
xx23fs = XAmXaMfemales;
xx14fs = XAMXamfemales;
xx24fs = XamXaMfemales;

xx33fs = XAmXAmfemales;
xx34fs = XAmXamfemales;
xx44fs = XamXamfemales;

xy11fs = XAMYAMfemales;
xy12fs = XAMYaMfemales;
xy21fs = XaMYAMfemales;
xy22fs = XaMYaMfemales;

xy31fs = XAmYAMfemales;
xy32fs = XAmYaMfemales;
xy41fs = XamYAMfemales;
xy42fs = XamYaMfemales;

xy13fs = XAMYAmfemales;
xy14fs = XAMYamfemales;
xy23fs = XaMYAmfemales;
xy24fs = XaMYamfemales;

xy33fs = XAmYAmfemales;
xy34fs = XAmYamfemales;
xy43fs = XamYAmfemales;
xy44fs = XamYamfemales;

yy11fs = YAMYAMfemales;
yy12fs = YAMYaMfemales;
yy22fs = YaMYaMfemales;

yy13fs = YAmYAMfemales;
yy23fs = YAmYaMfemales;
yy14fs = YamYAMfemales;
yy24fs = YamYaMfemales;

yy33fs = YAmYAmfemales;
yy34fs = YAmYamfemales;
yy44fs = YamYamfemales;
```

Let the probability of recombination (an odd number of crossover events) between the ancestral SDR and the selected locus be r. Let the probability of recombination between the selected locus and the novel SDR be R. And let the probability of recombination between the ancestral and novel SDRs be ρ.

Let the probability that a heterozygote at the selected locus passes down an A depend on sex, αf or αm. E.g., when αf=1/2 there is no meiotic drive in females.

The next generation frequencies in female gametes are then

```
(*X bearing ovules*)
nextXAMf = xx11fs + xx13fs / 2 + (xx12fs + xx14fs * (1 - R) + xx23fs * R) αf +
   xy11fs / 2 + (xy12fs (1 - r) + xy21fs (r)) αf +
   (xy13fs (1 - ρ) + xy31fs (ρ)) / 2 +
   xy14fs * αf +
   (xy14fs (- (R + r + ρ)) +
      xy41fs (r + ρ - R) +
      xy23fs (R + r - ρ) +
      xy32fs (R + ρ - r)) αf / 2;

nextXaMf = xx22fs + xx24fs / 2 + (xx12fs + xx23fs * (1 - R) + xx14fs * R) (1 - αf) +
   xy22fs / 2 + (xy12fs (r) + xy21fs (1 - r)) (1 - αf) +
   (xy24fs (1 - ρ) + xy42fs (ρ)) / 2 +
   xy23fs * (1 - αf) +
   (xy14fs (R + r - ρ) +
      xy41fs (R + ρ - r) +
      xy23fs (- (R + r + ρ)) +
      xy32fs (r + ρ - R)) (1 - αf) / 2;

nextXAmf = xx33fs + xx13fs / 2 + (xx23fs * (1 - R) + R * xx14fs + xx34fs) αf +
   xy33fs / 2 + (xy34fs (1 - r) + xy43fs (r)) αf +
   (xy13fs (ρ) + xy31fs (1 - ρ)) / 2 +
   xy32fs * αf +
   (xy14fs (R + ρ - r) +
      xy41fs (R + r - ρ) +
      xy23fs (r + ρ - R) +
      xy32fs (- (R + r + ρ))) αf / 2;

nextXamf = xx44fs + xx24fs / 2 + (xx14fs * (1 - R) + xx23fs * R + xx34fs) (1 - αf) +
   xy44fs / 2 + (xy34fs (r) + xy43fs (1 - r)) (1 - αf) +
   (xy24fs (ρ) + xy42fs (1 - ρ)) / 2 +
   xy41fs * (1 - αf) +
   (xy14fs (r + ρ - R) +
      xy41fs (- (R + r + ρ)) +
      xy23fs (R + ρ - r) +
      xy32fs (R + r - ρ)) (1 - αf) / 2;

(*Y bearing ovules*)
nextYAMf = yy11fs + yy13fs / 2 + (yy12fs + yy14fs * (1 - R) + yy23fs * R) αf +
   xy11fs / 2 + (xy12fs (r) + xy21fs (1 - r)) (αf) +
   (xy13fs (ρ) + xy31fs (1 - ρ)) / 2 +
   xy41fs * αf +
   (xy14fs (r + ρ - R) +
       xy41fs (- (R + r + ρ)) +
      xy23fs (R + ρ - r) +
      xy32fs (R + r - ρ)) αf / 2;

nextYaMf = yy22fs + yy24fs / 2 + (yy12fs + yy23fs * (1 - R) + yy14fs * R) (1 - αf) +
   xy22fs / 2 + (xy12fs (1 - r) + xy21fs (r)) (1 - αf) +
   (xy24fs (ρ) + xy42fs (1 - ρ)) / 2 +
   xy32fs * (1 - αf) +
   (xy14fs (R + ρ - r) +
      xy41fs (R + r - ρ) +
      xy23fs (r + ρ - R) +
      xy32fs (- (R + r + ρ))) (1 - αf) / 2;
```

```
nextYAmf = yy33fs + yy13fs / 2 + (yy23fs * (1 - R) + R * yy14fs + yy34fs) αf +
   xy33fs / 2 + (xy34fs (r) + xy43fs (1 - r)) (αf) +
   (xy13fs (1 - ρ) + xy31fs (ρ)) / 2 +
   xy23fs * αf +
   (xy14fs (R + r - ρ) +
      xy41fs (R + ρ - r) +
      xy23fs (- (R + r + ρ)) +
      xy32fs (r + ρ - R)) αf / 2;

nextYamf = yy44fs + yy24fs / 2 + (yy14fs * (1 - R) + yy23fs * R + yy34fs) (1 - αf) +
   xy44fs / 2 + (xy34fs (1 - r) + xy43fs (r)) (1 - αf) +
   (xy24fs (1 - ρ) + xy42fs (ρ)) / 2 +
   xy14fs * (1 - αf) +
   (xy14fs (- (R + r + ρ)) +
      xy41fs (r + ρ - R) +
      xy23fs (R + r - ρ) +
      xy32fs (R + ρ - r)) (1 - αf) / 2;
```

```
Factor[
 nextXAMf + nextXaMf + nextXAmf + nextXamf + nextYAMf + nextYaMf + nextYAmf + nextYamf]
```

1

And the next generation frequencies in male gametes are

```
(*X bearing pollen*)
nextXAMm = xx11ms + xx13ms / 2 + (xx12ms + xx14ms * (1 - R) + xx23ms * R) αm +
   xy11ms / 2 + (xy12ms (1 - r) + xy21ms (r)) αm +
   (xy13ms (1 - ρ) + xy31ms (ρ)) / 2 +
   xy14ms * αm +
   (xy14ms (- (R + r + ρ)) +
      xy41ms (r + ρ - R) +
      xy23ms (R + r - ρ) +
      xy32ms (R + ρ - r)) αm / 2;

nextXaMm = xx22ms + xx24ms / 2 + (xx12ms + xx23ms * (1 - R) + xx14ms * R) (1 - αm) +
   xy22ms / 2 + (xy12ms (r) + xy21ms (1 - r)) (1 - αm) +
   (xy24ms (1 - ρ) + xy42ms (ρ)) / 2 +
   xy23ms * (1 - αm) +
   (xy14ms (R + r - ρ) +
      xy41ms (R + ρ - r) +
      xy23ms (- (R + r + ρ)) +
      xy32ms (r + ρ - R)) (1 - αm) / 2;

nextXAmm = xx33ms + xx13ms / 2 + (xx23ms * (1 - R) + R * xx14ms + xx34ms) αm +
   xy33ms / 2 + (xy34ms (1 - r) + xy43ms (r)) αm +
   (xy13ms (ρ) + xy31ms (1 - ρ)) / 2 +
   xy32ms * αm +
   (xy14ms (R + ρ - r) +
      xy41ms (R + r - ρ) +
      xy23ms (r + ρ - R) +
      xy32ms (- (R + r + ρ))) αm / 2;

nextXamm = xx44ms + xx24ms / 2 + (xx14ms * (1 - R) + xx23ms * R + xx34ms) (1 - αm) +
   xy44ms / 2 + (xy34ms (r) + xy43ms (1 - r)) (1 - αm) +
```

```
    (xy24ms (ρ) + xy42ms (1 - ρ)) / 2 +
    xy41ms * (1 - αm) +
    (xy14ms (r + ρ - R) +
       xy41ms (- (R + r + ρ)) +
       xy23ms (R + ρ - r) +
       xy32ms (R + r - ρ)) (1 - αm) / 2;

(*Y bearing pollen*)
nextYAMm = yy11ms + yy13ms / 2 + (yy12ms + yy14ms * (1 - R) + yy23ms * R) αm +
    xy11ms / 2 + (xy12ms (r) + xy21ms (1 - r)) (αm) +
    (xy13ms (ρ) + xy31ms (1 - ρ)) / 2 +
    xy41ms * αm +
    (xy14ms (r + ρ - R) +
        xy41ms (- (R + r + ρ)) +
       xy23ms (R + ρ - r) +
       xy32ms (R + r - ρ)) αm / 2;

nextYaMm = yy22ms + yy24ms / 2 + (yy12ms + yy23ms * (1 - R) + yy14ms * R) (1 - αm) +
    xy22ms / 2 + (xy12ms (1 - r) + xy21ms (r)) (1 - αm) +
    (xy24ms (ρ) + xy42ms (1 - ρ)) / 2 +
    xy32ms * (1 - αm) +
    (xy14ms (R + ρ - r) +
       xy41ms (R + r - ρ) +
       xy23ms (r + ρ - R) +
       xy32ms (- (R + r + ρ))) (1 - αm) / 2;

nextYAmm = yy33ms + yy13ms / 2 + (yy23ms * (1 - R) + R * yy14ms + yy34ms) αm +
    xy33ms / 2 + (xy34ms (r) + xy43ms (1 - r)) (αm) +
    (xy13ms (1 - ρ) + xy31ms (ρ)) / 2 +
    xy23ms * αm +
    (xy14ms (R + r - ρ) +
       xy41ms (R + ρ - r) +
       xy23ms (- (R + r + ρ)) +
       xy32ms (r + ρ - R)) αm / 2;

nextYamm = yy44ms + yy24ms / 2 + (yy14ms * (1 - R) + yy23ms * R + yy34ms) (1 - αm) +
    xy44ms / 2 + (xy34ms (1 - r) + xy43ms (r)) (1 - αm) +
    (xy24ms (1 - ρ) + xy42ms (ρ)) / 2 +
    xy14ms * (1 - αm) +
    (xy14ms (- (R + r + ρ)) +
       xy41ms (r + ρ - R) +
       xy23ms (R + r - ρ) +
       xy32ms (R + ρ - r)) (1 - αm) / 2;

Factor[nextXAMm + nextXaMm + nextXAmm +
  nextXamm + nextYAMm + nextYaMm + nextYAmm + nextYamm]
```

1

Let the probability a zygote develops as a female be completely determined by the ancestral SDR when a zygote has no m alleles (XX females and XY and YY males) and be k whenever a zygote has at least on m allele:

```
SUBS = {
    kXXMM → 1,
    kXYMM → 0,
    kYYMM → 0,
    kXXMm → k,
    kXYMm → k,
    kYYMm → k,
    kXXmm → k,
    kXYmm → k,
    kYYmm → k
   };
```

Resident equilibria and stability

General

We first explore the resident equilibria, where the m allele is absent and sex is only determined by the ancestral sex-determining locus. In this case there no Y alleles in gametes from females and no M alleles in gametes from any sex, leaving us with 6 equations.

```
{nextXAMm, nextXaMm, nextXAmm, nextXamm, nextYAMm, nextYaMm, nextYAmm, nextYamm,
          nextXAMf, nextXaMf, nextXAmf, nextXamf, nextYAMf, nextYaMf, nextYAmf,
          nextYamf} /. XAmm → 0 /. Xamm → 0 /. YAmm → 0 /. Yamm → 0 /. XAmf → 0 /.
     Xamf → 0 /. YAmf → 0 /. Yamf → 0 /. YAMf → 0 /. YaMf → 0 /. SUBS // Simplify
```

```
{(MAA wAf wAm XAMf YAMm + 2 MAa (- (-1 + r) wAf wam XAMf YaMm + r waf wAm XaMf YAMm) αm) /
  (2 (Maa waf wam XaMf YaMm + MAa wAf wam XAMf YaMm +
      MAa waf wAm XaMf YAMm + MAA wAf wAm XAMf YAMm)), (Maa waf wam XaMf YaMm -
    2 MAa (r wAf wam XAMf YaMm + waf wAm XaMf YAMm - r waf wAm XaMf YAMm) (-1 + αm)) /
  (2 (Maa waf wam XaMf YaMm + MAa wAf wam XAMf YaMm + MAa waf wAm XaMf YAMm +
      MAA wAf wAm XAMf YAMm)), 0, 0, (MAA wAf wAm XAMf YAMm +
    2 MAa (r wAf wam XAMf YaMm + waf wAm XaMf YAMm - r waf wAm XaMf YAMm) αm) /
  (2 (Maa waf wam XaMf YaMm + MAa wAf wam XAMf YaMm +
      MAa waf wAm XaMf YAMm + MAA wAf wAm XAMf YAMm)),
 (Maa waf wam XaMf YaMm + 2 MAa ((-1 + r) wAf wam XAMf YaMm - r waf wAm XaMf YAMm) (-1 + αm)) /
  (2 (Maa waf wam XaMf YaMm + MAa wAf wam XAMf YaMm +
      MAa waf wAm XaMf YAMm + MAA wAf wAm XAMf YAMm)), 0, 0,
      FAA wAf wAm XAMf XAMm + FAa wAf wam XAMf XaMm αf + FAa waf wAm XaMf XAMm αf
 ─────────────────────────────────────────────────────────────────────────────────────
 Faa waf wam XaMf XaMm + FAa wAf wam XAMf XaMm + FAa waf wAm XaMf XAMm + FAA wAf wAm XAMf XAMm
 ,
      Faa waf wam XaMf XaMm - FAa (wAf wam XAMf XaMm + waf wAm XaMf XAMm) (-1 + αf)
 ─────────────────────────────────────────────────────────────────────────────────────
 Faa waf wam XaMf XaMm + FAa wAf wam XAMf XaMm + FAa waf wAm XaMf XAMm + FAA wAf wAm XAMf XAMm
 , 0, 0, 0, 0, 0, 0}
```

Letting pXf be the frequency of A in non-mutant female gametes, pXm be the frequency of A in in non-mutant male gametes with an X allele, pYm be the frequency of A in in non-mutant female gametes with a Y allele, and q be the frequency of Y alleles in male gametes

```
subequil = {
   XAMf → pXf,
   XaMf → (1 - pXf),
   YAMf → 0,
   YaMf → 0,
   XAMm → pXm (1 - q),
   XaMm → (1 - pXm) (1 - q),
   YAMm → pYm * q,
   YaMm → (1 - pYm) * q,
   XAmf → 0,
   Xamf → 0,
   XAmm → 0,
   Xamm → 0,
   YAmm → 0,
   Yamm → 0,
   YAmf → 0,
   Yamf → 0
  };
```

this system of six equations reduces to four (because the two female gamete frequencies must sum to one, as must the 4 male gamete frequencies).

We now wish to solve for the equilibrium frequencies of the haploid genotypes in gametes of each sex. Equilibrium is achieved when the following four equations are zero

```
differenceEqs = {
      nextXAMf - XAMf,
      nextXAMm - XAMm,
      nextYAMm - YAMm,
      nextYAMm + nextYaMm - (YAMm + YaMm)
     } /. SUBS /. subequil;
```

The first equation is zero when

```
sub1 = Flatten[Solve[differenceEqs[[3]] == 0, pXf]] // Factor
```

$$\{pXf \to (2\ pYm\ waf\ (-Maa\ q\ wam + Maa\ pYm\ q\ wam - MAa\ pYm\ q\ wAm + MAa\ wAm\ \alpha m - MAa\ r\ wAm\ \alpha m)) \Big/ \\ (-2\ Maa\ pYm\ q\ waf\ wam + 2\ Maa\ pYm^2\ q\ waf\ wam + 2\ MAa\ pYm\ q\ wAf\ wam - 2\ MAa\ pYm^2\ q\ wAf\ wam - \\ 2\ MAa\ pYm^2\ q\ waf\ wAm - MAA\ pYm\ wAf\ wAm + 2\ MAA\ pYm^2\ q\ wAf\ wAm - 2\ MAa\ r\ wAf\ wam\ \alpha m + \\ 2\ MAa\ pYm\ r\ wAf\ wam\ \alpha m + 2\ MAa\ pYm\ waf\ wAm\ \alpha m - 2\ MAa\ pYm\ r\ waf\ wAm\ \alpha m)\}$$

The second equation is zero when

```
sub2 = Flatten[Solve[Factor[differenceEqs[[2]] /. sub1] == 0, pXm]] // Factor
```

```
{pXm →
  -(pYm (-Maa MAA pYm q wam wAm + Maa MAA pYm^2 q wam wAm - MAa MAA pYm^2 q wAm^2 - 2 Maa MAa q
          wam^2 αm + 4 Maa MAa pYm q wam^2 αm - 2 Maa MAa pYm^2 q wam^2 αm + 2 Maa MAa q r wam^2 αm -
        4 Maa MAa pYm q r wam^2 αm + 2 Maa MAa pYm^2 q r wam^2 αm - 2 MAa^2 pYm q wam wAm αm +
        2 MAa^2 pYm^2 q wam wAm αm + 4 MAa^2 pYm q r wam wAm αm - 4 MAa^2 pYm^2 q r wam wAm αm +
        MAa MAA pYm wAm^2 αm - 2 MAa MAA pYm r wAm^2 αm + 2 MAa MAA pYm^2 q r wAm^2 αm + 2 MAa^2 wam
          wAm αm^2 - 2 MAa^2 pYm wam wAm αm^2 - 4 MAa^2 r wam wAm αm^2 + 4 MAa^2 pYm r wam wAm αm^2)) /
    ((-1 + q) (-Maa MAA pYm wam wAm + Maa MAA pYm^2 wam wAm - MAa MAA pYm^2 wAm^2 -
        2 Maa MAa r wam^2 αm + 4 Maa MAa pYm r wam^2 αm - 2 Maa MAa pYm^2 r wam^2 αm +
        2 MAa^2 pYm wam wAm αm - 2 MAa^2 pYm^2 wam wAm αm - 4 MAa^2 pYm r wam wAm αm +
        4 MAa^2 pYm^2 r wam wAm αm + 2 MAa MAA pYm^2 wAm^2 αm - 2 MAa MAA pYm^2 r wAm^2 αm))}
```

The fourth equation is zero when

```
sub3 = Flatten[Solve[Factor[differenceEqs[[4]] /. sub1 /. sub2] == 0, q]] // Factor
```

```
{q → -(Maa MAA pYm wam wAm - Maa MAA pYm^2 wam wAm + 2 MAa MAA pYm^2 r wAm^2 +
      2 Maa MAa r wam^2 αm - 4 Maa MAa pYm r wam^2 αm + 2 Maa MAa pYm^2 r wam^2 αm -
      4 MAa^2 pYm wam wAm αm + 4 MAa^2 pYm^2 wam wAm αm + 8 MAa^2 pYm r wam wAm αm -
      8 MAa^2 pYm^2 r wam wAm αm - 2 MAa MAA pYm^2 r wAm^2 αm + 4 MAa^2 pYm wam wAm αm^2 -
      4 MAa^2 pYm^2 wam wAm αm^2 - 8 MAa^2 pYm r wam wAm αm^2 + 8 MAa^2 pYm^2 r wam wAm αm^2) /
    (2 (Maa MAa pYm wam^2 - 2 Maa MAa pYm^2 wam^2 + Maa MAa pYm^3 wam^2 - 2 Maa MAa pYm r wam^2 +
        4 Maa MAa pYm^2 r wam^2 - 2 Maa MAa pYm^3 r wam^2 - Maa MAA pYm wam wAm +
        2 MAa^2 pYm^2 wam wAm + Maa MAA pYm^2 wam wAm - 2 MAa^2 pYm^3 wam wAm -
        4 MAa^2 pYm^2 r wam wAm + 4 MAa^2 pYm^3 r wam wAm - MAa MAA pYm^2 wAm^2 + MAa MAA pYm^3 wAm^2 -
        2 MAa MAA pYm^3 r wAm^2 - 2 Maa MAa pYm wam^2 αm + 4 Maa MAa pYm^2 wam^2 αm -
        2 Maa MAa pYm^3 wam^2 αm - 2 Maa MAa r wam^2 αm + 8 Maa MAa pYm r wam^2 αm -
        10 Maa MAa pYm^2 r wam^2 αm + 4 Maa MAa pYm^3 r wam^2 αm + 2 MAa^2 pYm wam wAm αm -
        6 MAa^2 pYm^2 wam wAm αm + 4 MAa^2 pYm^3 wam wAm αm - 4 MAa^2 pYm r wam wAm αm +
        12 MAa^2 pYm^2 r wam wAm αm - 8 MAa^2 pYm^3 r wam wAm αm + 2 MAa MAA pYm^2 wAm^2 αm -
        2 MAa MAA pYm^3 wAm^2 αm - 2 MAa MAA pYm^2 r wAm^2 αm + 4 MAa MAA pYm^3 r wAm^2 αm))}
```

Leaving us with an equation for pYm only

```
equilsol = Numerator[Factor[differenceEqs[[1]] /. sub1 /. sub2 /. sub3]];
```

Here we use linear stability analysis to examine when the resident equilibrium (i.e., no mutants) derived above is stable.

The internal stability matrix (i.e., the Jacobian for the residents) is

```
eqs = {nextXAMf, nextXAMm / (nextXAMm + nextXaMm),
     nextYAMm / (nextYAMm + nextYaMm), nextYAMm + nextYaMm} /. SUBS /. subequil;
matIntFull = Transpose[{
        D[eqs, pXf],
        D[eqs, pXm],
        D[eqs, pYm],
        D[eqs, freqYm]
       }] /. SUBS /. subequil // Factor;
```

The last column of matIntFull is full of zeros, corresponding to an eigenvalue of zero and indicating that the sex ratio in the system equilibrates immediately:

```
Transpose[matIntFull][[4]]
```

```
{0, 0, 0, 0}
```

As a consequence, we drop the last row and column in matIntFull:

```
matIntFull = Table[matIntFull[[i, j]], {i, 1, 3}, {j, 1, 3}];
```

The characteristic polynomial is

```
charpolyIntFull = Det[matIntFull - IdentityMatrix[3] * λ];
```

We will explore the equilibria for two special cases, tight linkage and weak selection.

Tight linkage

Equilibria

Here we look at the case where there is tight linkage between the ancestral sex-determining locus and the selected locus.

When there is complete linkage, r=0 (ie the selected locus is in the ancestral sex-determining region), the equilibrium frquency of A on Y gametes in males is

```
equilsol /. r → 0 // Factor;
equilsol2 = Solve[% == 0, pYm] // Simplify
```

```
{{pYm → 0}, {pYm → 0}, {pYm → 0}, {pYm → 1}, {pYm → 1}, {pYm → 1},
  {pYm → (wam (Faa Maa waf wam (MAA wAm + 2 MAa wam (-1 + αm)) + 2 FAA MAa wAf wAm αm
         (-Maa wam + 2 MAa wAm αm) - FAa (Maa wam + 2 MAa wAm αm) (Maa waf wam (-1 + αf) +
           MAA wAf wAm αf + 2 MAa (wAf wam αf (-1 + αm) - waf wAm (-1 + αf) αm)))) /
     (Faa waf wam (MAA wAm + 2 MAa wam (-1 + αm)) (Maa wam + 2 MAa wAm (-1 + αm)) +
       FAA wAf wAm (MAA wAm - 2 MAa wam αm) (Maa wam - 2 MAa wAm αm) -
       FAa (Maa wam^2 - wAm (MAA wAm + MAa wam (2 - 4 αm))) (Maa waf wam (-1 + αf) +
          MAA wAf wAm αf + 2 MAa (wAf wam αf (-1 + αm) - waf wAm (-1 + αf) αm)))}}
```

First, consider cases where pYm=0.

With allele a fixed on Y (pYm=0), then {pXf, pXm, q} equal:

```
Solve[(differenceEqs /. r → 0 /. pYm → 0 // Factor) == {0, 0, 0, 0}, {pXf, pXm, q}] //
 Simplify
```

$$\left\{\left\{pXf \to 0,\ pXm \to 0,\ q \to \frac{1}{2}\right\},\ \{pXf \to 1,\ pXm \to 1,\ q \to 1 - \alpha m\},\right.$$

$$\left\{pXf \to \frac{waf\ (Faa\ Maa\ waf\ wam - FAa\ wAf\ \alpha f\ (Maa\ wam + 2\ MAa\ wAm\ \alpha m))}{Faa\ Maa\ waf^2\ wam + wAf\ (2\ FAA\ MAa\ wAf\ wAm\ \alpha m - FAa\ waf\ (Maa\ wam + 2\ MAa\ wAm\ \alpha m))},\right.$$

$$pXm \to -(2\ MAa\ \alpha m\ (-Faa\ Maa\ waf\ wam + FAa\ wAf\ \alpha f\ (Maa\ wam + 2\ MAa\ wAm\ \alpha m)))\ /\ (2\ Maa\ MAa\ (Faa\ waf\ wam + FAA\ wAf\ wAm)\ \alpha m + FAa\ (Maa\ wam + 2\ MAa\ wAm\ \alpha m)\ (Maa\ waf\ (-1 + \alpha f) - 2\ MAa\ wAf\ \alpha f\ \alpha m)),$$

$$q \to (FAa\ (Maa\ waf\ (-1 + \alpha f) + 2\ MAa\ wAf\ \alpha f\ (-1 + \alpha m))\ (Maa\ wam + 2\ MAa\ wAm\ \alpha m) + 2\ Maa\ MAa\ (-Faa\ waf\ wam\ (-1 + \alpha m) + FAA\ wAf\ wAm\ \alpha m))\ /\ (2\ (FAa\ (Maa\ waf\ (-1 + \alpha f) - MAa\ wAf\ \alpha f)\ (Maa\ wam + 2\ MAa\ wAm\ \alpha m) + Maa\ MAa\ (Faa\ waf\ wam + 2\ FAA\ wAf\ wAm\ \alpha m)))\left.\right\}\left.\right\}$$

The last of these has a polymorphism on the X chromosome; we'll call this equilA0 ("0" because this is the leading order term in r...we could take additional order terms to get the linear approximation in r, etc.).

```
equilA0 = {pXf → (pXf /. %[[3]]), pXm → (pXm /. %[[3]]), pYm → 0, q →  (q /. %[[3]])}
```

$$\left\{pXf \to \frac{waf\ (Faa\ Maa\ waf\ wam - FAa\ wAf\ \alpha f\ (Maa\ wam + 2\ MAa\ wAm\ \alpha m))}{Faa\ Maa\ waf^2\ wam + wAf\ (2\ FAA\ MAa\ wAf\ wAm\ \alpha m - FAa\ waf\ (Maa\ wam + 2\ MAa\ wAm\ \alpha m))},\right.$$

$$pXm \to -(2\ MAa\ \alpha m\ (-Faa\ Maa\ waf\ wam + FAa\ wAf\ \alpha f\ (Maa\ wam + 2\ MAa\ wAm\ \alpha m)))\ /\ (2\ Maa\ MAa\ (Faa\ waf\ wam + FAA\ wAf\ wAm)\ \alpha m + FAa\ (Maa\ wam + 2\ MAa\ wAm\ \alpha m)\ (Maa\ waf\ (-1 + \alpha f) - 2\ MAa\ wAf\ \alpha f\ \alpha m)),\ pYm \to 0,$$

$$q \to (FAa\ (Maa\ waf\ (-1 + \alpha f) + 2\ MAa\ wAf\ \alpha f\ (-1 + \alpha m))\ (Maa\ wam + 2\ MAa\ wAm\ \alpha m) + 2\ Maa\ MAa\ (-Faa\ waf\ wam\ (-1 + \alpha m) + FAA\ wAf\ wAm\ \alpha m))\ /\ (2\ (FAa\ (Maa\ waf\ (-1 + \alpha f) - MAa\ wAf\ \alpha f)\ (Maa\ wam + 2\ MAa\ wAm\ \alpha m) + Maa\ MAa\ (Faa\ waf\ wam + 2\ FAA\ wAf\ wAm\ \alpha m)))\left.\right\}$$

The second of these is also polymorphic (Y fixed on a and X fixed on A), which we'll call equilB0.

```
equilB0 = {pXf → (pXf /. %%[[2]]), pXm → (pXm /. %%[[2]]), pYm → 0, q -> (q /. %%[[2]])}
```

$$\{pXf \to 1,\ pXm \to 1,\ pYm \to 0,\ q \to 1 - \alpha m\}$$

Next, consider cases where pYm=1.

With allele A fixed on Y (pYm=0), then {pXf, pXm, q} equal:

```
Solve[(differenceEqs /. r → 0 /. pYm → 1 // Factor) == {0, 0, 0, 0}, {pXf, pXm, q}] //
 Simplify
```

$$\left\{\{pXf \to 0,\ pXm \to 0,\ q \to \alpha m\},\ \left\{pXf \to 1,\ pXm \to 1,\ q \to \frac{1}{2}\right\},\right.$$
$$\{pXf \to (waf\ (FAa\ wAf\ \alpha f\ (MAA\ wAm - 2\ MAa\ wam\ (-1 + \alpha m)) + 2\ Faa\ MAa\ waf\ wam\ (-1 + \alpha m)))\ \Big/ \\ \Big(wAf\ (-FAA\ MAA\ wAf\ wAm + FAa\ waf\ (MAA\ wAm - 2\ MAa\ wam\ (-1 + \alpha m))) + \\ 2\ Faa\ MAa\ waf^2\ wam\ (-1 + \alpha m)\Big),$$
$$pXm \to (MAA\ (FAa\ wAf\ \alpha f\ (MAA\ wAm - 2\ MAa\ wam\ (-1 + \alpha m)) + 2\ Faa\ MAa\ waf\ wam\ (-1 + \alpha m)))\ / \\ (-FAa\ (-MAA\ wAm + 2\ MAa\ wam\ (-1 + \alpha m))\ (MAA\ wAf\ \alpha f + 2\ MAa\ waf\ (-1 + \alpha f)\ (-1 + \alpha m)) + \\ 2\ MAa\ MAA\ (Faa\ waf\ wam + FAA\ wAf\ wAm)\ (-1 + \alpha m)),$$
$$q \to (2\ Faa\ MAa\ MAA\ waf\ wam\ (-1 + \alpha m) - 2\ FAA\ MAa\ MAA\ wAf\ wAm\ \alpha m + \\ FAa\ (-MAA\ wAm + 2\ MAa\ wam\ (-1 + \alpha m))\ (-MAA\ wAf\ \alpha f + 2\ MAa\ waf\ (-1 + \alpha f)\ \alpha m))\ / \\ (2\ (FAa\ (MAa\ waf\ (-1 + \alpha f) - MAA\ wAf\ \alpha f)\ (-MAA\ wAm + 2\ MAa\ wam\ (-1 + \alpha m)) + \\ MAa\ MAA\ (-FAA\ wAf\ wAm + 2\ Faa\ waf\ wam\ (-1 + \alpha m))))\}\}$$

The last of these has a polymorphism on the X chromosome; we'll call this equilAp0 ("p" for prime -- the alternate allele is fixed on the Y).

```
equilAp0 = {pXf → (pXf /. %[[3]]), pXm → (pXm /. %[[3]]), pYm → 1, q → (q /. %[[3]])}
```

$$\{pXf \to (waf\ (FAa\ wAf\ \alpha f\ (MAA\ wAm - 2\ MAa\ wam\ (-1 + \alpha m)) + 2\ Faa\ MAa\ waf\ wam\ (-1 + \alpha m)))\ \Big/ \\ \Big(wAf\ (-FAA\ MAA\ wAf\ wAm + FAa\ waf\ (MAA\ wAm - 2\ MAa\ wam\ (-1 + \alpha m))) + \\ 2\ Faa\ MAa\ waf^2\ wam\ (-1 + \alpha m)\Big),$$
$$pXm \to (MAA\ (FAa\ wAf\ \alpha f\ (MAA\ wAm - 2\ MAa\ wam\ (-1 + \alpha m)) + 2\ Faa\ MAa\ waf\ wam\ (-1 + \alpha m)))\ / \\ (-FAa\ (-MAA\ wAm + 2\ MAa\ wam\ (-1 + \alpha m))\ (MAA\ wAf\ \alpha f + 2\ MAa\ waf\ (-1 + \alpha f)\ (-1 + \alpha m)) + \\ 2\ MAa\ MAA\ (Faa\ waf\ wam + FAA\ wAf\ wAm)\ (-1 + \alpha m)),$$
$$pYm \to 1,\ q \to (2\ Faa\ MAa\ MAA\ waf\ wam\ (-1 + \alpha m) - 2\ FAA\ MAa\ MAA\ wAf\ wAm\ \alpha m + \\ FAa\ (-MAA\ wAm + 2\ MAa\ wam\ (-1 + \alpha m))\ (-MAA\ wAf\ \alpha f + 2\ MAa\ waf\ (-1 + \alpha f)\ \alpha m))\ / \\ (2\ (FAa\ (MAa\ waf\ (-1 + \alpha f) - MAA\ wAf\ \alpha f)\ (-MAA\ wAm + 2\ MAa\ wam\ (-1 + \alpha m)) + \\ MAa\ MAA\ (-FAA\ wAf\ wAm + 2\ Faa\ waf\ wam\ (-1 + \alpha m))))\}$$

The first of these is also polymorphic (Y fixed on A and X fixed on a), which we'll call equilBp0.

```
equilBp0 = {pXf → (pXf /. %%[[1]]), pXm → (pXm /. %%[[1]]), pYm → 1, q → (q /. %%[[1]])}
```

$$\{pXf \to 0,\ pXm \to 0,\ pYm \to 1,\ q \to \alpha m\}$$

Finally, we have the equilibrium with a polymorphism on the Y (which will turn out not to be stable)

```
Factor[{pXf, pXm, pYm, q} /. sub1 /. sub2 /. sub3 /. r → 0] /. Last[equilsol2] //
  Simplify;
equilC0 = {pXf → %[[1]], pXm → %[[2]], pYm → %[[3]], q → %[[4]]}
```

```
{pXf → waf (Maa wam - 2 MAa wAm αm) /
   (Maa waf wam + MAA wAf wAm + 2 MAa (-wAf wam + wAf wam αm - waf wAm αm)),
 pXm → (wam (Maa wam - 2 MAa wAm αm)
     (-Faa waf (MAA wAm + 2 MAa wam (-1 + αm)) + FAa (Maa waf wam (-1 + αf) +
         MAA wAf wAm αf + 2 MAa (wAf wam αf (-1 + αm) - waf wAm (-1 + αf) αm)))) /
   (-(Faa waf wam + FAA wAf wAm) (MAA wAm + 2 MAa wam (-1 + αm)) (Maa wam - 2 MAa wAm αm) +
     FAa (Maa waf wam (-1 + αf) + MAA wAf wAm αf +
        2 MAa (wAf wam αf (-1 + αm) - waf wAm (-1 + αf) αm))
      (Maa wam^2 - wAm (MAA wAm + 2 MAa wam (-1 + 2 αm)))),
 pYm → (wam (Faa Maa waf wam (MAA wAm + 2 MAa wam (-1 + αm)) + 2 FAA MAa wAf wAm αm
        (-Maa wam + 2 MAa wAm αm) - FAa (Maa wam + 2 MAa wAm αm) (Maa waf wam (-1 + αf) +
         MAA wAf wAm αf + 2 MAa (wAf wam αf (-1 + αm) - waf wAm (-1 + αf) αm)))) /
   (Faa waf wam (MAA wAm + 2 MAa wam (-1 + αm)) (Maa wam + 2 MAa wAm (-1 + αm)) +
     FAA wAf wAm (MAA wAm - 2 MAa wam αm) (Maa wam - 2 MAa wAm αm) -
     FAa (Maa wam^2 - wAm (MAA wAm + MAa wam (2 - 4 αm))) (Maa waf wam (-1 + αf) +
        MAA wAf wAm αf + 2 MAa (wAf wam αf (-1 + αm) - waf wAm (-1 + αf) αm))),
 q → (Faa waf wam (MAA wAm + 2 MAa wam (-1 + αm)) (Maa wam + 2 MAa wAm (-1 + αm)) +
     FAA wAf wAm (MAA wAm - 2 MAa wam αm) (Maa wam - 2 MAa wAm αm) -
     FAa (Maa wam^2 - wAm (MAA wAm + MAa wam (2 - 4 αm))) (Maa waf wam (-1 + αf) +
        MAA wAf wAm αf + 2 MAa (wAf wam αf (-1 + αm) - waf wAm (-1 + αf) αm))) /
   (2 (Faa waf wam (Maa wam - MAa wAm) (MAA wAm + 2 MAa wam (-1 + αm)) +
       FAA wAf wAm (MAa wam - MAA wAm) (-Maa wam + 2 MAa wAm αm) -
       FAa (Maa wam^2 - MAA wAm^2) (Maa waf wam (-1 + αf) + MAA wAf wAm αf +
          2 MAa (wAf wam αf (-1 + αm) - waf wAm (-1 + αf) αm))))}
```

We'll call this equilC0.

We can define four terms to convert these leading order equilibria into the form found in the main text

```
trysub = {
   ϕ → (1 + αDf) wAf FAa (wam Maa + (1 + αDm) wAm MAa) / 2 - wam waf Maa Faa,
   ψ → (1 - αDf) waf FAa (wam Maa + (1 + αDm) wAm MAa) / 2 - (1 + αDm) wAm wAf MAa FAA,
   ϕp → (1 - αDf) waf FAa (wAm MAA + (1 - αDm) wam MAa) / 2 - wAm wAf MAA FAA,
   ψp → (1 + αDf) wAf FAa (wAm MAA + (1 - αDm) wam MAa) / 2 - (1 - αDm) wam waf MAa Faa
  };
(q /. equilA0 /. αm → (αDm + 1) / 2 /. αf → (αDf + 1) / 2) - (1 - αDm MAa ϕ / (MAa ϕ + Maa ψ)) / 2 /.
   trysub // Factor // Simplify
(pXf /. equilA0 /. αm → (αDm + 1) / 2 /. αf → (αDf + 1) / 2) -
  (waf ϕ / (waf ϕ + wAf ψ) /. trysub) // Factor
(pXm /. equilA0 /. αm → (αDm + 1) / 2 /. αf → (αDf + 1) / 2) -
  ((1 + αDm) MAa ϕ / ((1 + αDm) MAa ϕ + Maa ψ) /. trysub) // Factor
(q /. equilAp0 /. αm → (αDm + 1) / 2 /. αf → (αDf + 1) / 2) - (1 + αDm MAa ϕp / (MAa ϕp + MAA ψp)) / 2 /.
   trysub // Factor // Simplify
(pXf /. equilAp0 /. αm → (αDm + 1) / 2 /. αf → (αDf + 1) / 2) -
  (1 - wAf ϕp / (wAf ϕp + waf ψp) /. trysub) // Factor
(pXm /. equilAp0 /. αm → (αDm + 1) / 2 /. αf → (αDf + 1) / 2) -
  (1 - (1 - αDm) MAa ϕp / ((1 - αDm) MAa ϕp + MAA ψp) /. trysub) // Factor
```

0

0

0

0

0

0

When there is no haploid selection these reduce to the equilibria in Otto 2014 JEB 27:1431 (where diploid fitness is relative to heterozygotes, and equilB and equilBp are switched)

```
({q, pYm, pXm, pXf} /. equilA0 /. αm → 1 / 2 /. αf → 1 / 2 /. waf → 1 /. wAf → 1 /. wAm → 1 /.
    wam → 1 /. MAa → 1 /. FAa → 1) ==
  {1 / 2, 0, (1 + Maa - 2 Faa Maa) / ((1 + Maa)^2 - 2 (Faa + FAA) Maa), (1 + Maa - 2 Faa Maa) / (2 (1 + Maa - FAA - Faa Maa))} // Simplify
({q, pYm, pXm, pXf} /. equilAp0 /. αm → 1 / 2 /. αf → 1 / 2 /. waf → 1 /. wAf → 1 /. wAm → 1 /.
    wam → 1 /. MAa → 1 /. FAa → 1) ==
  {1 / 2, 1, (MAA + MAA^2 - 2 Faa MAA) / ((1 + MAA)^2 - 2 (Faa + FAA) MAA), (1 + MAA - 2 Faa) / (2 (1 + MAA - Faa - FAA MAA))} // Simplify
({q, pYm, pXm, pXf} /. equilB0 /. αm → 1 / 2 /. αf → 1 / 2 /. waf → 1 /. wAf → 1 /. wAm → 1 /.
   wam → 1) == {1 / 2, 0, 1, 1} // Simplify
({q, pYm, pXm, pXf} /. equilBp0 /. αm → 1 / 2 /. αf → 1 / 2 /. waf → 1 /. wAf → 1 /. wAm → 1 /.
   wam → 1) == {1 / 2, 1, 0, 0} // Simplify
({q, pYm, pXm, pXf} /. equilC0 /. αm → 1 / 2 /. αf → 1 / 2 /. waf → 1 /. wAf → 1 /. wAm → 1 /.
    wam → 1 /. MAa → 1 /. FAa → 1) ==
  {1 / 2, (2 FAA (1 - Maa) - 2 Faa Maa (1 - MAA) - (1 + Maa) (MAA - Maa)) / ((MAA - Maa)^2 + 2 (Faa + FAA) (1 - MAA) (1 - Maa)),
   ((1 - Maa) (2 Faa (1 - MAA) + MAA - Maa)) / ((MAA - Maa)^2 + 2 (Faa + FAA) (1 - MAA) (1 - Maa)), (1 - Maa) / (2 - Maa - MAA)} // Simplify
```

```
True

True

True

True

True
```

Characteristic polynomial

The characteristic polynomial with r = 0 is

```
charpolyIntFullr0 = charpolyIntFull /. r → 0;
```

We consider only equilibria A, B (i.e., with a fixed on Y), and C. The conditions required for equilibria A' and B' can be obtained by interchanging the alleles, *A* and *a*.

Stability of equilA (r ~ 0)

The three allele frequencies at this equilibrium are

```
eq1 = Factor[pXf /. equilA0]
```

$$\frac{\text{waf}\,(\text{Faa Maa waf wam} - \text{FAa Maa wAf wam}\,\alpha f - 2\,\text{FAa MAa wAf wAm}\,\alpha f\,\alpha m)}{\text{Faa Maa waf}^2\,\text{wam} - \text{FAa Maa waf wAf wam} - 2\,\text{FAa MAa waf wAf wAm}\,\alpha m + 2\,\text{FAA MAa wAf}^2\,\text{wAm}\,\alpha m}$$

```
eq2 = Factor[pXm /. equilA0]
```

$$(2\,\text{MAa}\,\alpha m\,(-\text{Faa Maa waf wam}+\text{FAa Maa wAf wam}\,\alpha f+2\,\text{FAa MAa wAf wAm}\,\alpha f\,\alpha m))\Big/\Big(\text{FAa Maa}^2\,\text{waf wam}-\text{FAa Maa}^2\,\text{waf wam}\,\alpha f-2\,\text{Faa Maa MAa waf wam}\,\alpha m+2\,\text{FAa Maa MAa waf wAm}\,\alpha m-2\,\text{FAA Maa MAa wAf wAm}\,\alpha m+2\,\text{FAa Maa MAa wAf wam}\,\alpha f\,\alpha m-2\,\text{FAa Maa MAa waf wAm}\,\alpha f\,\alpha m+4\,\text{FAa MAa}^2\,\text{wAf wAm}\,\alpha f\,\alpha m^2\Big)$$

```
eq3 = Factor[pYm /. equilA0]
```

0

Therefore each of the following terms must be positive for the equilibrium to be valid:

```
{{eq1, 1 - eq1}, {eq2, 1 - eq2}} // Simplify
```

$$\Big\{\Big\{\frac{\text{waf}\,(\text{Faa Maa waf wam}-\text{FAa wAf}\,\alpha f\,(\text{Maa wam}+2\,\text{MAa wAm}\,\alpha m))}{\text{Faa Maa waf}^2\,\text{wam}+\text{wAf}\,(2\,\text{FAA MAa wAf wAm}\,\alpha m-\text{FAa waf}\,(\text{Maa wam}+2\,\text{MAa wAm}\,\alpha m))},$$
$$\frac{\text{wAf}\,(2\,\text{FAA MAa wAf wAm}\,\alpha m+\text{FAa waf}\,(-1+\alpha f)\,(\text{Maa wam}+2\,\text{MAa wAm}\,\alpha m))}{\text{Faa Maa waf}^2\,\text{wam}+\text{wAf}\,(2\,\text{FAA MAa wAf wAm}\,\alpha m-\text{FAa waf}\,(\text{Maa wam}+2\,\text{MAa wAm}\,\alpha m))}\Big\},$$
$$\{-(2\,\text{MAa}\,\alpha m\,(-\text{Faa Maa waf wam}+\text{FAa wAf}\,\alpha f\,(\text{Maa wam}+2\,\text{MAa wAm}\,\alpha m)))\,/\,(2\,\text{Maa MAa}\,(\text{Faa waf wam}+\text{FAA wAf wAm})\,\alpha m+\text{FAa}\,(\text{Maa wam}+2\,\text{MAa wAm}\,\alpha m)\,(\text{Maa waf}\,(-1+\alpha f)-2\,\text{MAa wAf}\,\alpha f\,\alpha m)),$$
$$(\text{Maa}\,(2\,\text{FAA MAa wAf wAm}\,\alpha m+\text{FAa waf}\,(-1+\alpha f)\,(\text{Maa wam}+2\,\text{MAa wAm}\,\alpha m)))\,/\,(2\,\text{Maa MAa}\,(\text{Faa waf wam}+\text{FAA wAf wAm})\,\alpha m+\text{FAa}\,(\text{Maa wam}+2\,\text{MAa wAm}\,\alpha m)\,(\text{Maa waf}\,(-1+\alpha f)-2\,\text{MAa wAf}\,\alpha f\,\alpha m))\}\Big\}$$

Notice that the numerators are common in sign for {eq1,1-eq1} and {eq2,1-eq2}. Thus, either the numerators and denominators must be both positive or both negative, the equilibrium will not be valid otherwise. That is, we must have either:

Condition 1: $\frac{\text{FAa wAf}\,\alpha f\,(\text{Maa wam}+2\,\text{MAa wAm}\,\alpha m)}{\text{Maa waf wam}} > \text{Faa}$ and $\frac{\text{FAa waf}\,(1-\alpha f)\,(\text{Maa wam}+2\,\text{MAa wAm}\,\alpha m)}{2\,\text{MAa wAf wAm}\,\alpha m} > \text{FAA}$

or

Condition 2: $\frac{\text{FAa wAf}\,\alpha f\,(\text{Maa wam}+2\,\text{MAa wAm}\,\alpha m)}{\text{Maa waf wam}} < \text{Faa}$ and $\frac{\text{FAa waf}\,(1-\alpha f)\,(\text{Maa wam}+2\,\text{MAa wAm}\,\alpha m)}{2\,\text{MAa wAf wAm}\,\alpha m} < \text{FAA}$

Reduce[{0 < eq1 < 1, 0 < eq2 < 1, simpcond}, {FAA, Faa}] // Factor

$$\left(0 \le Rf \le \frac{1}{2} \text{ \&\& } MAA > 0 \text{ \&\& } 0 \le Rm \le \frac{1}{2} \text{ \&\& } wAf > 0 \text{ \&\& } 0 < \alpha m < 1 \text{ \&\& } waf > 0 \text{ \&\& } Maa > 0 \text{ \&\& } MAa > 0 \text{ \&\& } 0 < \alpha f < 1 \text{ \&\& } wam > 0 \text{ \&\& } wAm > 0 \text{ \&\& } FAa > 0 \text{ \&\& } 0 < FAA < -\frac{FAa\, waf\, (-1+\alpha f)\, (Maa\, wam + 2\, MAa\, wAm\, \alpha m)}{2\, MAa\, wAf\, wAm\, \alpha m} \text{ \&\& } 0 < Faa < \frac{FAa\, wAf\, \alpha f\, (Maa\, wam + 2\, MAa\, wAm\, \alpha m)}{Maa\, waf\, wam}\right) \text{ || }$$

$$\left(0 \le Rf \le \frac{1}{2} \text{ \&\& } MAA > 0 \text{ \&\& } 0 \le Rm \le \frac{1}{2} \text{ \&\& } wAf > 0 \text{ \&\& } 0 < \alpha m < 1 \text{ \&\& } waf > 0 \text{ \&\& } Maa > 0 \text{ \&\& } MAa > 0 \text{ \&\& } 0 < \alpha f < 1 \text{ \&\& } wam > 0 \text{ \&\& } wAm > 0 \text{ \&\& } FAa > 0 \text{ \&\& } FAA > -\frac{FAa\, waf\, (-1+\alpha f)\, (Maa\, wam + 2\, MAa\, wAm\, \alpha m)}{2\, MAa\, wAf\, wAm\, \alpha m} \text{ \&\& } Faa > \frac{FAa\, wAf\, \alpha f\, (Maa\, wam + 2\, MAa\, wAm\, \alpha m)}{Maa\, waf\, wam}\right)$$

That is, equilA0 is valid if:

$$\textbf{validcondA} = \left(\left(FAA < \frac{FAa\, waf\, (1-\alpha f)\, (Maa\, wam + 2\, MAa\, wAm\, \alpha m)}{2\, MAa\, wAf\, wAm\, \alpha m} \text{ \&\& } Faa < \frac{FAa\, wAf\, \alpha f\, (Maa\, wam + 2\, MAa\, wAm\, \alpha m)}{Maa\, waf\, wam}\right) \text{ || } \left(FAA > \frac{FAa\, waf\, (1-\alpha f)\, (Maa\, wam + 2\, MAa\, wAm\, \alpha m)}{2\, MAa\, wAf\, wAm\, \alpha m} \text{ \&\& } Faa > \frac{FAa\, wAf\, \alpha f\, (Maa\, wam + 2\, MAa\, wAm\, \alpha m)}{Maa\, waf\, wam}\right)\right);$$

Making a set of substitutions that might help simplify factors below:

subA1 = (Solve[eq1 == pXf, MAa] // Flatten)

$$\left\{MAa \to \frac{Maa\, waf\, wam\, (-Faa\, waf + Faa\, pXf\, waf - FAa\, pXf\, wAf + FAa\, wAf\, \alpha f)}{2\, wAf\, wAm\, (FAa\, pXf\, waf - FAA\, pXf\, wAf - FAa\, waf\, \alpha f)\, \alpha m}\right\}$$

subA2 = Solve[(eq2 - pXm /. subA1) == 0, FAa] // Flatten

$$\left\{FAa \to \frac{(-pXf + pXf^2)\, (-Faa\, waf\, wam + Faa\, pXm\, waf\, wam + FAA\, pXm\, wAf\, wAm)}{(-pXf\, wAf\, wam + pXf\, pXm\, wAf\, wam - pXm\, waf\, wAm + pXf\, pXm\, waf\, wAm)\, (pXf - \alpha f)}\right\}$$

subA2b = Solve[(eq2 - pXm /. subA1) == 0, αf] // Flatten

$$\{\alpha f \to (pXf\, (-Faa\, waf\, wam + Faa\, pXf\, waf\, wam + Faa\, pXm\, waf\, wam - Faa\, pXf\, pXm\, waf\, wam - FAa\, pXf\, wAf\, wam + FAa\, pXf\, pXm\, wAf\, wam - FAa\, pXm\, waf\, wAm + FAa\, pXf\, pXm\, waf\, wAm + FAA\, pXm\, wAf\, wAm - FAA\, pXf\, pXm\, wAf\, wAm))\, /\, (FAa\, (-pXf\, wAf\, wam + pXf\, pXm\, wAf\, wam - pXm\, waf\, wAm + pXf\, pXm\, waf\, wAm))\}$$

The equilibrium A is stable if the three eigenvalues of the stability matrix are all less than one in magnitude. We are guaranteed that the eigenvalues are real and that the largest of the eigenvalues is non-negative by the Perron-Frobenius theorem, given that the stability matrix is non-negative.

The characteristic polynomial is

Printed by Wolfram Mathematica Student Edition

```
charpolyIntFullr0 /. equilA0 // Factor
```

$$\big(\big(-\text{Faa Maa MAA waf wam wAm} + \text{FAa Maa MAA wAf wam wAm}\,\alpha f + 2\,\text{FAa Maa MAa waf wam wAm}\,\alpha m - 2\,\text{FAa Maa MAa waf wam wAm}\,\alpha f\,\alpha m + 2\,\text{FAa MAa MAA wAf wAm}^2\,\alpha f\,\alpha m + 4\,\text{FAa MAa}^2\,\text{waf wAm}^2\,\alpha m^2 - 4\,\text{FAA MAa}^2\,\text{wAf wAm}^2\,\alpha m^2 - 4\,\text{FAa MAa}^2\,\text{waf wAm}^2\,\alpha f\,\alpha m^2 - \text{FAa Maa}^2\,\text{waf wam}^2\,\lambda + 2\,\text{Faa Maa MAa waf wam}^2\,\lambda + \text{FAa Maa}^2\,\text{waf wam}^2\,\alpha f\,\lambda - 2\,\text{FAa Maa MAa wAf wam}^2\,\alpha f\,\lambda - 2\,\text{Faa Maa MAa waf wam}^2\,\alpha m\,\lambda - 2\,\text{FAa Maa MAa waf wam wAm}\,\alpha m\,\lambda + 2\,\text{FAA Maa MAa wAf wam wAm}\,\alpha m\,\lambda + 2\,\text{FAa Maa MAa wAf wam}^2\,\alpha f\,\alpha m\,\lambda + 2\,\text{FAa Maa MAa waf wam wAm}\,\alpha f\,\alpha m\,\lambda - 4\,\text{FAa MAa}^2\,\text{wAf wam wAm}\,\alpha f\,\alpha m\,\lambda + 4\,\text{FAa MAa}^2\,\text{wAf wam wAm}\,\alpha f\,\alpha m^2\,\lambda\big)$$

$$\big(-2\,\text{Faa FAa Maa MAa waf}^2\,\text{wam wAm}\,\alpha m + 2\,\text{Faa FAA Maa MAa waf wAf wam wAm}\,\alpha m + 2\,\text{Faa FAa Maa MAa waf}^2\,\text{wam wAm}\,\alpha f\,\alpha m - 2\,\text{FAa FAA Maa MAa wAf}^2\,\text{wam wAm}\,\alpha f\,\alpha m - \text{Faa FAa Maa}^2\,\text{waf}^2\,\text{wam}^2\,\lambda + \text{Faa FAa Maa}^2\,\text{waf}^2\,\text{wam}^2\,\alpha f\,\lambda + 2\,\text{Faa FAA Maa MAa waf wAf wam wAm}\,\alpha m\,\lambda - 4\,\text{FAa FAA MAa}^2\,\text{wAf}^2\,\text{wAm}^2\,\alpha f\,\alpha m^2\,\lambda + \text{FAa}^2\,\text{Maa}^2\,\text{waf wAf wam}^2\,\alpha f\,\lambda^2 - \text{FAa}^2\,\text{Maa}^2\,\text{waf wAf wam}^2\,\alpha f^2\,\lambda^2 - 2\,\text{Faa FAA Maa MAa waf wAf wam wAm}\,\alpha m\,\lambda^2 + 4\,\text{FAa}^2\,\text{Maa MAa waf wAf wam wAm}\,\alpha f\,\alpha m\,\lambda^2 - 4\,\text{FAa}^2\,\text{Maa MAa waf wAf wam wAm}\,\alpha f^2\,\alpha m\,\lambda^2 + 4\,\text{FAa}^2\,\text{MAa}^2\,\text{waf wAf wAm}^2\,\alpha f\,\alpha m^2\,\lambda^2 - 4\,\text{FAa}^2\,\text{MAa}^2\,\text{waf wAf wAm}^2\,\alpha f^2\,\alpha m^2\,\lambda^2\big)\big) \Big/$$

$$\Big(\text{waf wAf wam}\,\big(-\text{FAa Maa}^2\,\text{waf wam} + 2\,\text{Faa Maa MAa waf wam} + \text{FAa Maa}^2\,\text{waf wam}\,\alpha f - 2\,\text{FAa Maa MAa wAf wam}\,\alpha f - 2\,\text{Faa Maa MAa waf wam}\,\alpha m - 2\,\text{FAa Maa MAa waf wAm}\,\alpha m + 2\,\text{FAA Maa MAa wAf wAm}\,\alpha m + 2\,\text{FAa Maa MAa wAf wam}\,\alpha f\,\alpha m + 2\,\text{FAa Maa MAa waf wAm}\,\alpha f\,\alpha m - 4\,\text{FAa MAa}^2\,\text{wAf wAm}\,\alpha f\,\alpha m + 4\,\text{FAa MAa}^2\,\text{wAf wAm}\,\alpha f\,\alpha m^2\big)$$

$$\big(-\text{FAa}^2\,\text{Maa}^2\,\text{wam}^2\,\alpha f + \text{FAa}^2\,\text{Maa}^2\,\text{wam}^2\,\alpha f^2 + 2\,\text{Faa FAA Maa MAa wam wAm}\,\alpha m - 4\,\text{FAa}^2\,\text{Maa MAa wam wAm}\,\alpha f\,\alpha m + 4\,\text{FAa}^2\,\text{Maa MAa wam wAm}\,\alpha f^2\,\alpha m - 4\,\text{FAa}^2\,\text{MAa}^2\,\text{wAm}^2\,\alpha f\,\alpha m^2 + 4\,\text{FAa}^2\,\text{MAa}^2\,\text{wAm}^2\,\alpha f^2\,\alpha m^2\big)\Big)$$

The first eigenvalue is

```
Numerator[Factor[charpolyIntFull /. r → 0 /. equilA0]][[1]] // Simplify;
eigenA1 = λ /. (Solve[% == 0, λ] // Simplify // Flatten)
```

$$\big(\text{wAm}\,\big(\text{Faa Maa MAA waf wam} + 4\,\text{FAA MAa}^2\,\text{wAf wAm}\,\alpha m^2 + \text{FAa}\,(\text{Maa wam} + 2\,\text{MAa wAm}\,\alpha m)\,(-\text{MAA wAf}\,\alpha f + 2\,\text{MAa waf}\,(-1+\alpha f)\,\alpha m)\big)\big) \Big/ \big(\text{wam}\,(\text{FAa}\,(\text{Maa waf}\,(-1+\alpha f) + 2\,\text{MAa wAf}\,\alpha f\,(-1+\alpha m))\,(\text{Maa wam} + 2\,\text{MAa wAm}\,\alpha m) + 2\,\text{Maa MAa}\,(-\text{Faa waf wam}\,(-1+\alpha m) + \text{FAA wAf wAm}\,\alpha m))\big)$$

This first eigenvalue is consistent with stability if the following is positive:

```
Collect[1 - eigenA1 /. subA1 /. subA2, MAA, Factor]
```

$$\frac{\text{MAA pXf}\,(-1+\text{pXm})\,\text{wAf wAm}\,\alpha m}{\text{Maa}\,(-1+\text{pXf})\,\text{waf wam}\,(-\text{pXm} - \alpha m + 2\,\text{pXm}\,\alpha m)} - \frac{\text{pXf pXm wAf wam} + \text{pXf wAf wam}\,\alpha m - 2\,\text{pXf pXm wAf wam}\,\alpha m - \text{pXm waf wAm}\,\alpha m + \text{pXf pXm waf wAm}\,\alpha m}{\text{pXf wAf wam}\,(-\text{pXm} - \alpha m + 2\,\text{pXm}\,\alpha m)}$$

which we write more clearly as:

$$-\frac{\text{MAA}\,(1-\text{pXm})\,\text{pXf}\,\text{wAf}\,\text{wAm}\,\alpha\text{m}}{\text{Maa}\,(1-\text{pXf})\,\text{waf}\,\text{wam}\,(\text{pXm}\,(1-\alpha\text{m})+\alpha\text{m}\,(1-\text{pXm}))}+\frac{\text{pXf}\,\text{wAf}\,\text{wam}\,(\text{pXm}\,(1-\alpha\text{m})+\alpha\text{m}\,(1-\text{pXm}))-\text{pXm}\,(1-\text{pXf})\,\text{waf}\,\text{wAm}\,\alpha\text{m}}{\text{pXf}\,\text{wAf}\,\text{wam}\,(\text{pXm}\,(1-\alpha\text{m})+\alpha\text{m}\,(1-\text{pXm}))};$$

```
% - %% // FullSimplify
```

0

The first term is never positive, so we require that MAA be sufficiently small, $\text{MAA}<\frac{\text{Maa}\,(1-\text{pXf})\,\text{waf}\,(\text{pXf}\,\text{wAf}\,\text{wam}\,(\text{pXm}\,(1-\alpha\text{m})+\alpha\text{m}\,(1-\text{pXm}))-\text{pXm}\,(1-\text{pXf})\,\text{waf}\,\text{wAm}\,\alpha\text{m})}{(1-\text{pXm})\,\text{pXf}^2\,\text{wAf}^2\,\text{wAm}\,\alpha\text{m}}$, which itself requires that the second term is positive (i.e., we must have pXf wAf wam (pX1(1-αm)+αm(1-pX1))-pX1 (1-pXf) waf wAm αm>0 for there to be any parameter set in which MAA can be positive and less than this quantity):

```
Solve[%% == 0, MAA] // Simplify
```

$$\{\{\text{MAA}\to(\text{Maa}\,(-1+\text{pXf})\,\text{waf}\,(-\text{pXm}\,\text{waf}\,\text{wAm}\,\alpha\text{m}+\text{pXf}\,(\text{wAf}\,\text{wam}\,\alpha\text{m}+\text{pXm}\,(\text{wAf}\,\text{wam}-2\,\text{wAf}\,\text{wam}\,\alpha\text{m}+\text{waf}\,\text{wAm}\,\alpha\text{m}))))\,/\,(\text{pXf}^2\,(-1+\text{pXm})\,\text{wAf}^2\,\text{wAm}\,\alpha\text{m})\}\}$$

The last two eigenvalues are more readily interpreted by analysing the quadratic portion of the characteristic polynomial (with leading $+\lambda^2$ term):

```
Table[matIntFull[[i, j]], {i, 1, 2}, {j, 1, 2}] /. r → 0 /. equilA0 // Factor;
charpolyA = Det[% - λ IdentityMatrix[2]] // Factor;
```

Stability requires that the slope at λ=1 is positive and that the intercept at λ=1 is positive, otherwise one or both of these two eigenvalues will be greater than one:

```
slopeA = D[charpolyA, λ] /. λ → 1 /. subA1 /. subA2 // Factor
```

$$(-\text{Faa}\,\text{pXf}^2\,\text{waf}\,\text{wAf}\,\text{wam}^2+2\,\text{Faa}\,\text{pXf}^2\,\text{pXm}\,\text{waf}\,\text{wAf}\,\text{wam}^2-\text{Faa}\,\text{pXf}^2\,\text{pXm}^2\,\text{waf}\,\text{wAf}\,\text{wam}^2+2\,\text{FAA}\,\text{pXf}^2\,\text{pXm}\,\text{wAf}^2\,\text{wam}\,\text{wAm}-2\,\text{FAA}\,\text{pXf}^2\,\text{pXm}^2\,\text{wAf}^2\,\text{wam}\,\text{wAm}+\text{FAA}\,\text{pXf}\,\text{pXm}^2\,\text{waf}\,\text{wAf}\,\text{wAm}^2-\text{FAA}\,\text{pXf}^2\,\text{pXm}^2\,\text{waf}\,\text{wAf}\,\text{wAm}^2-\text{Faa}\,\text{pXf}\,\text{waf}\,\text{wAf}\,\text{wam}^2\,\alpha\text{f}+2\,\text{Faa}\,\text{pXf}^2\,\text{waf}\,\text{wAf}\,\text{wam}^2\,\alpha\text{f}+2\,\text{Faa}\,\text{pXf}\,\text{pXm}\,\text{waf}\,\text{wAf}\,\text{wam}^2\,\alpha\text{f}-4\,\text{Faa}\,\text{pXf}^2\,\text{pXm}\,\text{waf}\,\text{wAf}\,\text{wam}^2\,\alpha\text{f}-\text{Faa}\,\text{pXf}\,\text{pXm}^2\,\text{waf}\,\text{wAf}\,\text{wam}^2\,\alpha\text{f}+2\,\text{Faa}\,\text{pXf}^2\,\text{pXm}^2\,\text{waf}\,\text{wAf}\,\text{wam}^2\,\alpha\text{f}-2\,\text{Faa}\,\text{pXm}\,\text{waf}^2\,\text{wam}\,\text{wAm}\,\alpha\text{f}+4\,\text{Faa}\,\text{pXf}\,\text{pXm}\,\text{waf}^2\,\text{wam}\,\text{wAm}\,\alpha\text{f}-2\,\text{Faa}\,\text{pXf}^2\,\text{pXm}\,\text{waf}^2\,\text{wam}\,\text{wAm}\,\alpha\text{f}+2\,\text{Faa}\,\text{pXm}^2\,\text{waf}^2\,\text{wam}\,\text{wAm}\,\alpha\text{f}-4\,\text{Faa}\,\text{pXf}\,\text{pXm}^2\,\text{waf}^2\,\text{wam}\,\text{wAm}\,\alpha\text{f}+2\,\text{Faa}\,\text{pXf}^2\,\text{pXm}^2\,\text{waf}^2\,\text{wam}\,\text{wAm}\,\alpha\text{f}-2\,\text{FAA}\,\text{pXf}^2\,\text{pXm}\,\text{wAf}^2\,\text{wam}\,\text{wAm}\,\alpha\text{f}+2\,\text{FAA}\,\text{pXf}^2\,\text{pXm}^2\,\text{wAf}^2\,\text{wam}\,\text{wAm}\,\alpha\text{f}+\text{FAA}\,\text{pXm}^2\,\text{waf}\,\text{wAf}\,\text{wAm}^2\,\alpha\text{f}-3\,\text{FAA}\,\text{pXf}\,\text{pXm}^2\,\text{waf}\,\text{wAf}\,\text{wAm}^2\,\alpha\text{f}+2\,\text{FAA}\,\text{pXf}^2\,\text{pXm}^2\,\text{waf}\,\text{wAf}\,\text{wAm}^2\,\alpha\text{f})\,/\,((-\text{pXf}\,\text{wAf}\,\text{wam}+\text{pXf}\,\text{pXm}\,\text{wAf}\,\text{wam}-\text{pXm}\,\text{waf}\,\text{wAm}+\text{pXf}\,\text{pXm}\,\text{waf}\,\text{wAm})\,(-\text{FAA}\,\text{pXf}\,\text{pXm}\,\text{wAf}\,\text{wAm}+\text{Faa}\,\text{waf}\,\text{wam}\,\alpha\text{f}-\text{Faa}\,\text{pXf}\,\text{waf}\,\text{wam}\,\alpha\text{f}-\text{Faa}\,\text{pXm}\,\text{waf}\,\text{wam}\,\alpha\text{f}+\text{Faa}\,\text{pXf}\,\text{pXm}\,\text{waf}\,\text{wam}\,\alpha\text{f}+\text{FAA}\,\text{pXf}\,\text{pXm}\,\text{wAf}\,\text{wAm}\,\alpha\text{f}))$$

```
slopeA2 = D[charpolyA, λ] /. λ → 1 /. subA1 /. subA2b // Factor
```

$$\left(\text{Faa pXf waf wAf wam}^2 - 2\,\text{Faa pXf}^2\,\text{waf wAf wam}^2 - 2\,\text{Faa pXf pXm waf wAf wam}^2 + 4\,\text{Faa pXf}^2\,\text{pXm waf wAf wam}^2 + \text{Faa pXf pXm}^2\,\text{waf wAf wam}^2 - 2\,\text{Faa pXf}^2\,\text{pXm}^2\,\text{waf wAf wam}^2 + 2\,\text{FAa pXf}^2\,\text{wAf}^2\,\text{wam}^2 - 4\,\text{FAa pXf}^2\,\text{pXm wAf}^2\,\text{wam}^2 + 2\,\text{FAa pXf}^2\,\text{pXm}^2\,\text{wAf}^2\,\text{wam}^2 + 2\,\text{Faa pXm waf}^2\,\text{wam wAm} - 4\,\text{Faa pXf pXm waf}^2\,\text{wam wAm} + 2\,\text{Faa pXf}^2\,\text{pXm waf}^2\,\text{wam wAm} - 2\,\text{Faa pXm}^2\,\text{waf}^2\,\text{wam wAm} + 4\,\text{Faa pXf pXm}^2\,\text{waf}^2\,\text{wam wAm} - 2\,\text{Faa pXf}^2\,\text{pXm}^2\,\text{waf}^2\,\text{wam wAm} + 4\,\text{FAa pXf pXm waf wAf wam wAm} - 4\,\text{FAa pXf}^2\,\text{pXm waf wAf wam wAm} - 4\,\text{FAa pXf pXm}^2\,\text{waf wAf wam wAm} + 4\,\text{FAa pXf}^2\,\text{pXm}^2\,\text{waf wAf wam wAm} + 2\,\text{FAA pXf}^2\,\text{pXm wAf}^2\,\text{wam wAm} - 2\,\text{FAA pXf}^2\,\text{pXm}^2\,\text{wAf}^2\,\text{wam wAm} + 2\,\text{FAa pXm}^2\,\text{waf}^2\,\text{wAm}^2 - 4\,\text{FAa pXf pXm}^2\,\text{waf}^2\,\text{wAm}^2 + 2\,\text{FAa pXf}^2\,\text{pXm}^2\,\text{waf}^2\,\text{wAm}^2 - \text{FAA pXm}^2\,\text{waf wAf wAm}^2 + 3\,\text{FAA pXf pXm}^2\,\text{waf wAf wAm}^2 - 2\,\text{FAA pXf}^2\,\text{pXm}^2\,\text{waf wAf wAm}^2\right) \Big/ \left(\left(-\text{pXf wAf wam} + \text{pXf pXm wAf wam} - \text{pXm waf wAm} + \text{pXf pXm waf wAm}\right)\left(-\text{Faa waf wam} + \text{Faa pXf waf wam} + \text{Faa pXm waf wam} - \text{Faa pXf pXm waf wam} - \text{FAa pXf wAf wam} + \text{FAa pXf pXm wAf wam} - \text{FAa pXm waf wAm} + \text{FAa pXf pXm waf wAm} - \text{FAA pXf pXm wAf wAm}\right)\right)$$

```
interceptA = charpolyA /. λ → 1 /. subA1 /. subA2 // Factor
```

$$(\text{Faa pXf waf wam} - \text{Faa pXf pXm waf wam} - \text{Faa pXf waf wam}\,\alpha f + \text{Faa pXf pXm waf wam}\,\alpha f - \text{FAA pXm wAf wAm}\,\alpha f + \text{FAA pXf pXm wAf wAm}\,\alpha f) / (-\text{FAA pXf pXm wAf wAm} + \text{Faa waf wam}\,\alpha f - \text{Faa pXf waf wam}\,\alpha f - \text{Faa pXm waf wam}\,\alpha f + \text{Faa pXf pXm waf wam}\,\alpha f + \text{FAA pXf pXm wAf wAm}\,\alpha f)$$

The intercept is easier to interpret using subA2b:

```
interceptA2 = charpolyA /. λ → 1 /. subA1 /. subA2b // Factor
```

$$(\text{Faa pXf waf wam} - \text{Faa pXf pXm waf wam} - \text{FAa pXf wAf wam} + \text{FAa pXf pXm wAf wam} - \text{FAa pXm waf wAm} + \text{FAa pXf pXm waf wAm} + \text{FAA pXm wAf wAm} - \text{FAA pXf pXm wAf wAm}) / (-\text{Faa waf wam} + \text{Faa pXf waf wam} + \text{Faa pXm waf wam} - \text{Faa pXf pXm waf wam} - \text{FAa pXf wAf wam} + \text{FAa pXf pXm wAf wam} - \text{FAa pXm waf wAm} + \text{FAa pXf pXm waf wAm} - \text{FAA pXf pXm wAf wAm})$$

Using Collect, this can be written as:

```
(-Faa pXf (1 - pXm) waf wam - FAA (1 - pXf) pXm wAf wAm +
    FAa (pXf (1 - pXm) wAf wam + pXm (1 - pXf) waf wAm)) / (Faa (1 - pXf) (1 - pXm) waf wam +
    FAA pXf pXm wAf wAm + FAa (pXf (1 - pXm) wAf wam + pXm (1 - pXf) waf wAm));
Factor[interceptA2 /
  %]
```

1

The denominator is always positive, so we require the numerator to be positive for stability, that is, FAa must be large enough for the intercept at λ=1 to be positive,

$$\text{FAa} > \frac{\text{Faa pXf }(1-\text{pXm})\text{ waf wam}+\text{FAA }(1-\text{pXf})\text{ pXm wAf wAm}}{\text{pXf }(1-\text{pXm})\text{ wAf wam}+\text{pXm }(1-\text{pXf})\text{ waf wAm}}.$$

Note that the fraction can be interpretted further in light of the validity conditions:

validcondA

$$\left(\text{FAA} < \frac{\text{FAa waf } (1 - \alpha f)\ (\text{Maa wam} + 2\ \text{MAa wAm}\ \alpha m)}{2\ \text{MAa wAf wAm}\ \alpha m}\ \&\&\ \text{Faa} < \frac{\text{FAa wAf}\ \alpha f\ (\text{Maa wam} + 2\ \text{MAa wAm}\ \alpha m)}{\text{Maa waf wam}}\right)\ ||$$
$$\left(\text{FAA} > \frac{\text{FAa waf } (1 - \alpha f)\ (\text{Maa wam} + 2\ \text{MAa wAm}\ \alpha m)}{2\ \text{MAa wAf wAm}\ \alpha m}\ \&\&\ \text{Faa} > \frac{\text{FAa wAf}\ \alpha f\ (\text{Maa wam} + 2\ \text{MAa wAm}\ \alpha m)}{\text{Maa waf wam}}\right)$$

Under the first of these conditions (equivalent to overdominance),
$\frac{\text{Faa pXf } (1-\text{pXm})\text{ waf wam}+\text{FAA } (1-\text{pXf})\text{ pXm wAf wAm}}{\text{pXf } (1-\text{pXm})\text{ wAf wam}+\text{pXm } (1-\text{pXf})\text{ waf wAm}}$ will be less than

$$\left(\frac{\text{FAa wAf}\ \alpha f\ (\text{Maa wam} + 2\ \text{MAa wAm}\ \alpha m)}{\text{Maa waf wam}}\ \text{pXf}\ (1 - \text{pXm})\ \text{waf wam} + \frac{\text{FAa waf}\ (1 - \alpha f)\ (\text{Maa wam} + 2\ \text{MAa wAm}\ \alpha m)}{2\ \text{MAa wAf wAm}\ \alpha m}\ (1 - \text{pXf})\ \text{pXm wAf wAm}\right) \Big/$$
$$(\text{pXf}\ (1 - \text{pXm})\ \text{wAf wam} + \text{pXm}\ (1 - \text{pXf})\ \text{waf wAm})\ /.\ \text{subA1}\ /.\ \text{subA2b}\ //\ \text{Factor}$$

FAa

which makes it guaranteed that $\text{FAa} > \frac{\text{Faa pXf } (1-\text{pXm})\text{ waf wam}+\text{FAA } (1-\text{pXf})\text{ pXm wAf wAm}}{\text{pXf } (1-\text{pXm})\text{ wAf wam}+\text{pXm } (1-\text{pXf})\text{ waf wAm}}$ (i.e., the validity conditions in the overdominance-like case ensure intercept condition for stability is satisfied).

Under the second of these conditions (equivalent to underdominance), however,
$\frac{\text{Faa pXf } (1-\text{pXm})\text{ waf wam}+\text{FAA } (1-\text{pXf})\text{ pXm wAf wAm}}{\text{pXf } (1-\text{pXm})\text{ wAf wam}+\text{pXm } (1-\text{pXf})\text{ waf wAm}}$ will be greater than FAa by the same calculations, making it impossible to satisfy the intercept condition for stability.

We conclude that only under the first validity conditions (equivalent to overdominance) is stability and validity of the equilibrium possible. Furthermore, under the first validity conditions, we are guaranteed that the intercept is positive at λ=1.

To complete the proof, we show below that the slope at λ=1 provides no further conditions on stability:

slopeA2 // Factor

$$\big(\text{Faa pXf waf wAf wam}^2 - 2\ \text{Faa pXf}^2\ \text{waf wAf wam}^2 - 2\ \text{Faa pXf pXm waf wAf wam}^2 +$$
$$4\ \text{Faa pXf}^2\ \text{pXm waf wAf wam}^2 + \text{Faa pXf pXm}^2\ \text{waf wAf wam}^2 - 2\ \text{Faa pXf}^2\ \text{pXm}^2\ \text{waf wAf wam}^2 +$$
$$2\ \text{FAa pXf}^2\ \text{wAf}^2\ \text{wam}^2 - 4\ \text{FAa pXf}^2\ \text{pXm wAf}^2\ \text{wam}^2 + 2\ \text{FAa pXf}^2\ \text{pXm}^2\ \text{wAf}^2\ \text{wam}^2 +$$
$$2\ \text{Faa pXm waf}^2\ \text{wam wAm} - 4\ \text{Faa pXf pXm waf}^2\ \text{wam wAm} + 2\ \text{Faa pXf}^2\ \text{pXm waf}^2\ \text{wam wAm} -$$
$$2\ \text{Faa pXm}^2\ \text{waf}^2\ \text{wam wAm} + 4\ \text{Faa pXf pXm}^2\ \text{waf}^2\ \text{wam wAm} - 2\ \text{Faa pXf}^2\ \text{pXm}^2\ \text{waf}^2\ \text{wam wAm} +$$
$$4\ \text{FAa pXf pXm waf wAf wam wAm} - 4\ \text{FAa pXf}^2\ \text{pXm waf wAf wam wAm} -$$
$$4\ \text{FAa pXf pXm}^2\ \text{waf wAf wam wAm} + 4\ \text{FAa pXf}^2\ \text{pXm}^2\ \text{waf wAf wam wAm} +$$
$$2\ \text{FAA pXf}^2\ \text{pXm wAf}^2\ \text{wam wAm} - 2\ \text{FAA pXf}^2\ \text{pXm}^2\ \text{wAf}^2\ \text{wam wAm} + 2\ \text{FAa pXm}^2\ \text{waf}^2\ \text{wAm}^2 -$$
$$4\ \text{FAa pXf pXm}^2\ \text{waf}^2\ \text{wAm}^2 + 2\ \text{FAa pXf}^2\ \text{pXm}^2\ \text{waf}^2\ \text{wAm}^2 - \text{FAA pXm}^2\ \text{waf wAf wAm}^2 +$$
$$3\ \text{FAA pXf pXm}^2\ \text{waf wAf wAm}^2 - 2\ \text{FAA pXf}^2\ \text{pXm}^2\ \text{waf wAf wAm}^2\big) \Big/$$
$$((-\text{pXf wAf wam} + \text{pXf pXm wAf wam} - \text{pXm waf wAm} + \text{pXf pXm waf wAm})$$
$$(-\text{Faa waf wam} + \text{Faa pXf waf wam} + \text{Faa pXm waf wam} - \text{Faa pXf pXm waf wam} - \text{FAa pXf wAf wam} +$$
$$\text{FAa pXf pXm wAf wam} - \text{FAa pXm waf wAm} + \text{FAa pXf pXm waf wAm} - \text{FAA pXf pXm wAf wAm}))$$

The proof we use involves rewriting the above in terms of the intercept, shown above to be positive:

```
subtry = Solve[int == interceptA2, FAa] // Flatten
```

```
{FAa → (Faa int waf wam + Faa pXf waf wam - Faa int pXf waf wam -
      Faa int pXm waf wam - Faa pXf pXm waf wam + Faa int pXf pXm waf wam +
      FAA pXm wAf wAm - FAA pXf pXm wAf wAm + FAA int pXf pXm wAf wAm) /
    ((-1 + int) (-pXf wAf wam + pXf pXm wAf wam - pXm waf wAm + pXf pXm waf wAm))}
```

Multiplying slopeA2 by a positive quantity to get rid of the denominator:

```
slopeA2 * ((pXf (1 - pXm) wAf wam + pXm (1 - pXf) waf wAm) (Faa waf wam (1 - pXf) (1 - pXm) +
      FAA wAf wAm pXf pXm + FAa ( wAf wam pXf (1 - pXm) + waf wAm (1 - pXf) pXm))) // Factor
```

$$
\begin{aligned}
&\mathrm{Faa\ pXf\ waf\ wAf\ wam}^2 - 2\ \mathrm{Faa\ pXf}^2\ \mathrm{waf\ wAf\ wam}^2 - 2\ \mathrm{Faa\ pXf\ pXm\ waf\ wAf\ wam}^2 + \\
&4\ \mathrm{Faa\ pXf}^2\ \mathrm{pXm\ waf\ wAf\ wam}^2 + \mathrm{Faa\ pXf\ pXm}^2\ \mathrm{waf\ wAf\ wam}^2 - 2\ \mathrm{Faa\ pXf}^2\ \mathrm{pXm}^2\ \mathrm{waf\ wAf\ wam}^2 + \\
&2\ \mathrm{FAa\ pXf}^2\ \mathrm{wAf}^2\ \mathrm{wam}^2 - 4\ \mathrm{FAa\ pXf}^2\ \mathrm{pXm\ wAf}^2\ \mathrm{wam}^2 + 2\ \mathrm{FAa\ pXf}^2\ \mathrm{pXm}^2\ \mathrm{wAf}^2\ \mathrm{wam}^2 + \\
&2\ \mathrm{Faa\ pXm\ waf}^2\ \mathrm{wam\ wAm} - 4\ \mathrm{Faa\ pXf\ pXm\ waf}^2\ \mathrm{wam\ wAm} + 2\ \mathrm{Faa\ pXf}^2\ \mathrm{pXm\ waf}^2\ \mathrm{wam\ wAm} - \\
&2\ \mathrm{Faa\ pXm}^2\ \mathrm{waf}^2\ \mathrm{wam\ wAm} + 4\ \mathrm{Faa\ pXf\ pXm}^2\ \mathrm{waf}^2\ \mathrm{wam\ wAm} - 2\ \mathrm{Faa\ pXf}^2\ \mathrm{pXm}^2\ \mathrm{waf}^2\ \mathrm{wam\ wAm} + \\
&4\ \mathrm{FAa\ pXf\ pXm\ waf\ wAf\ wam\ wAm} - 4\ \mathrm{FAa\ pXf}^2\ \mathrm{pXm\ waf\ wAf\ wam\ wAm} - \\
&4\ \mathrm{FAa\ pXf\ pXm}^2\ \mathrm{waf\ wAf\ wam\ wAm} + 4\ \mathrm{FAa\ pXf}^2\ \mathrm{pXm}^2\ \mathrm{waf\ wAf\ wam\ wAm} + \\
&2\ \mathrm{FAA\ pXf}^2\ \mathrm{pXm\ wAf}^2\ \mathrm{wam\ wAm} - 2\ \mathrm{FAA\ pXf}^2\ \mathrm{pXm}^2\ \mathrm{wAf}^2\ \mathrm{wam\ wAm} + \\
&2\ \mathrm{FAa\ pXm}^2\ \mathrm{waf}^2\ \mathrm{wAm}^2 - 4\ \mathrm{FAa\ pXf\ pXm}^2\ \mathrm{waf}^2\ \mathrm{wAm}^2 + 2\ \mathrm{FAa\ pXf}^2\ \mathrm{pXm}^2\ \mathrm{waf}^2\ \mathrm{wAm}^2 - \\
&\mathrm{FAA\ pXm}^2\ \mathrm{waf\ wAf\ wAm}^2 + 3\ \mathrm{FAA\ pXf\ pXm}^2\ \mathrm{waf\ wAf\ wAm}^2 - 2\ \mathrm{FAA\ pXf}^2\ \mathrm{pXm}^2\ \mathrm{waf\ wAf\ wAm}^2
\end{aligned}
$$

```
Collect[Factor[% (1 - int) /. subtry], {int, Faa, FAa, FAA}, Factor]
```

$$
\begin{aligned}
&-\mathrm{FAA\ pXm\ wAf\ wAm}\ (-2\ \mathrm{pXf\ wAf\ wam} + 2\ \mathrm{pXf\ pXm\ wAf\ wam} - \mathrm{pXm\ waf\ wAm} + \mathrm{pXf\ pXm\ waf\ wAm}) + \\
&\mathrm{Faa}\ (-1 + \mathrm{pXm})\ \mathrm{waf\ wam}\ (-\mathrm{pXf\ wAf\ wam} + \mathrm{pXf\ pXm\ wAf\ wam} - 2\ \mathrm{pXm\ waf\ wAm} + 2\ \mathrm{pXf\ pXm\ waf\ wAm}) + \\
&\mathrm{int}\left(\mathrm{Faa\ pXf}\ (-1 + \mathrm{pXm})^2\ \mathrm{waf\ wAf\ wam}^2 - \mathrm{FAA}\ (-1 + \mathrm{pXf})\ \mathrm{pXm}^2\ \mathrm{waf\ wAf\ wAm}^2\right)
\end{aligned}
$$

Below, we show that (1-int) is also positive (that is, the intercept at λ=1 is never greater than +1), the remaining quantity can be written as the following:

```
Faa waf wam (1 - pXm) (pXf (1 - pXm) wAf wam + 2 pXm (1 - pXf) waf wAm) +
   FAA wAf wAm pXm  (2 pXf (1 - pXm) wAf wam + pXm (1 - pXf) waf wAm) +
   int (Faa pXf (1 - pXm)^2 waf wAf wam^2 + FAA (1 - pXf) pXm^2 waf wAf wAm^2);
%% - % // Factor
```

0

If the intercept is negative, we already know that the system is unstable (above proof). If the intercept is positive, then the slope will also be positive because all of the terms in the above are positive and so is (1-int), shown next:

```
Factor[1 - interceptA2]
```

```
-(-Faa waf wam + Faa pXm waf wam - FAA pXm wAf wAm) /
  (Faa waf wam - Faa pXf waf wam - Faa pXm waf wam + Faa pXf pXm waf wam + FAa pXf wAf wam -
    FAa pXf pXm wAf wam + FAa pXm waf wAm - FAa pXf pXm waf wAm + FAA pXf pXm wAf wAm)
```

Using Collect, this can be written as the strictly positive quantity:

```
(Faa waf wam (1 - pXm) + FAA wAf wAm pXm) / (Faa waf wam (1 - pXf) (1 - pXm) +
     FAA wAf wAm pXf pXm + FAa ( wAf wam pXf (1 - pXm) + waf wAm (1 - pXf) pXm));
Factor[(1 - interceptA2) - %]
```

0

Hence the slope at λ=1 is positive, assuming that the intercept at λ=1 is positive, and adds no further constraint on the stability conditions.

Putting together the constraints on all of the eigenvalues, as well as the validity conditions, the conditions required for validity and stability are:

```
stabcondA = Simplify[Reduce[{MAA <
    Maa (1 - pXf) waf (pXf wAf wam (pXm (1 - αm) + αm (1 - pXm)) - pXm (1 - pXf) waf wAm αm)
    / ((1 - pXm) pXf^2 wAf^2 wAm αm),
   validcondA[[1]], simpcond, 0 < pXf < 1, 0 < pXm < 1}]]
```

$$0 \le Rf \le \frac{1}{2} \,\&\&\, 0 \le Rm \le \frac{1}{2} \,\&\&\, 0 < \alpha m < 1 \,\&\&\, 0 < pXm < 1 \,\&\&\, 0 < pXf < 1 \,\&\&\, wAm > 0 \,\&\&\, wam > 0 \,\&\&\, wAf > 0 \,\&\&\, 0 < waf < -\frac{pXf\, wAf\, wam\, (pXm + \alpha m - 2\, pXm\, \alpha m)}{(-1 + pXf)\, pXm\, wAm\, \alpha m} \,\&\&\, MAA > 0 \,\&\&\, Maa > \left(MAA\, pXf^2\, (-1 + pXm)\, wAf^2\, wAm\, \alpha m\right) / \left((-1 + pXf)\, waf\, (-pXm\, waf\, wAm\, \alpha m + pXf\, (wAf\, wam\, \alpha m + pXm\, (wAf\, wam - 2\, wAf\, wam\, \alpha m + waf\, wAm\, \alpha m)))\right) \,\&\&\, 0 < \alpha f < 1 \,\&\&\, MAa > 0 \,\&\&\, FAA > 0 \,\&\&\, FAa > -\frac{2\, FAA\, MAa\, wAf\, wAm\, \alpha m}{waf\, (-1 + \alpha f)\, (Maa\, wam + 2\, MAa\, wAm\, \alpha m)} \,\&\&\, 0 < Faa < \frac{FAa\, wAf\, \alpha f\, (Maa\, wam + 2\, MAa\, wAm\, \alpha m)}{Maa\, waf\, wam}$$

We can regain the validity and stability conditions from Otto (2014):

Condition for validity and stability: $\frac{1+Maa}{2\,Maa}$ > Faa and $\frac{1+Maa}{2}$> FAA and $1 - \frac{(1-Maa)\left(\frac{1+Maa}{2}-FAA\right)}{Maa\left(\frac{1+Maa}{2\,Maa}-Faa\right)}$ > MAA.

by reducing the model to that one (wAf=waf=wAm=wam=1, αm=αf=1/2, FAa=MAa=1):

```
validcondA[[1]] /.
 {wAf → 1, waf → 1, wAm → 1, wam → 1, αf → 1 / 2, αm → 1 / 2, FAa → 1, MAa → 1}
```

$$FAA < \frac{1 + Maa}{2} \,\&\&\, Faa < \frac{1 + Maa}{2\, Maa}$$

```
(MAA -
    Maa (1 - pXf) waf (pXf wAf wam (pXm (1 - αm) + αm (1 - pXm)) - pXm (1 - pXf) waf wAm αm)
    / ((1 - pXm) pXf^2 wAf^2 wAm αm))
  / (MAa ((MAa - Maa)/Maa ((1/2 (Maa/MAa + 1) - FAA/FAa) / (1/2 (MAa/Maa + 1) - Faa/FAa)) + (MAA - MAa)/MAa)) /. equilA0 /.
  {wAf → 1, waf → 1, wAm → 1, wam → 1, αf → 1 / 2, αm → 1 / 2} // Factor
```

1

We cannot have underdominance in marginal fitness in females (including ovule selection):

```
Reduce[{stabcondA, FAA wAf > FAa waf, Faa waf > FAa wAf}]
```

False

With haploid selection we need

```
-(Maa (1 - pXf) waf (pXf wAf wam (pXm (1 - αm) + αm (1 - pXm)) - pXm (1 - pXf) waf wAm αm))/((1 - pXm) pXf^2 wAf^2 wAm αm) + MAA <
    0 /. equilA0 // Simplify;
% /. Faa → (Faa /. Solve[ϕ == αf wAf FAa (wam Maa + 2 αm wAm MAa) - wam waf Maa Faa, Faa]) //
  Simplify;
% /. FAA → (FAA /. Solve[ψ == (1 - αf) waf FAa (wam Maa + 2 αm wAm MAa) - 2 αm wAm wAf MAa FAA,
      FAA]) // Simplify
```

$\{\{-\text{wAm}\,\phi\,(\text{MAA wAm}\,\phi - \text{Maa wam}\,\psi + 2\,\text{MAa}\,(\text{wam}\,(-1+\alpha\text{m})\,\phi + \text{wAm}\,\alpha\text{m}\,\psi))\}\} > 0$

Note that ϕ>0 whenever the equilibrium is valid and stable

```
Reduce[{αf wAf FAa (wam Maa + 2 αm wAm MAa) - wam waf Maa Faa > 0, simpcond}, Faa] // Factor
```

$\text{MAA} > 0\ \&\&\ 0 \le \text{Rf} \le \frac{1}{2}\ \&\&\ \text{FAA} > 0\ \&\&\ 0 \le \text{Rm} \le \frac{1}{2}\ \&\&\ 0 < \alpha\text{m} < 1\ \&\&$
$0 < \alpha\text{f} < 1\ \&\&\ \text{wAm} > 0\ \&\&\ \text{wam} > 0\ \&\&\ \text{Maa} > 0\ \&\&\ \text{MAa} > 0\ \&\&\ \text{wAf} > 0\ \&\&$
$\text{waf} > 0\ \&\&\ \text{FAa} > 0\ \&\&\ 0 < \text{Faa} < \frac{\text{FAa wAf}\,\alpha\text{f}\,(\text{Maa wam} + 2\,\text{MAa wAm}\,\alpha\text{m})}{\text{Maa waf wam}}$

```
validcondA[[1, 2]]
```

$\text{Faa} < \frac{\text{FAa wAf}\,\alpha\text{f}\,(\text{Maa wam} + 2\,\text{MAa wAm}\,\alpha\text{m})}{\text{Maa waf wam}}$

(as is ψ)

```
Reduce[{(1 - αf) waf FAa (wam Maa + 2 αm wAm MAa) - 2 αm wAm wAf MAa FAA > 0, simpcond},
  FAA] // Factor
```

$\text{MAA} > 0\ \&\&\ 0 \le \text{Rf} \le \frac{1}{2}\ \&\&\ \text{Faa} > 0\ \&\&\ 0 \le \text{Rm} \le \frac{1}{2}\ \&\&\ 0 < \alpha\text{m} < 1\ \&\&$
$0 < \alpha\text{f} < 1\ \&\&\ \text{wAm} > 0\ \&\&\ \text{wam} > 0\ \&\&\ \text{Maa} > 0\ \&\&\ \text{MAa} > 0\ \&\&\ \text{wAf} > 0\ \&\&$
$\text{waf} > 0\ \&\&\ \text{FAa} > 0\ \&\&\ 0 < \text{FAA} < -\frac{\text{FAa waf}\,(-1+\alpha\text{f})\,(\text{Maa wam} + 2\,\text{MAa wAm}\,\alpha\text{m})}{2\,\text{MAa wAf wAm}\,\alpha\text{m}}$

```
validcondA[[1, 1]]
```

$\text{FAA} < \frac{\text{FAa waf}\,(1-\alpha\text{f})\,(\text{Maa wam} + 2\,\text{MAa wAm}\,\alpha\text{m})}{2\,\text{MAa wAf wAm}\,\alpha\text{m}}$

We therefore need only

```
(MAA wAm ϕ - Maa wam ψ + 2 MAa (wam (-1 + αm) ϕ + wAm αm ψ)) < 0;
Collect[%, {wAm, wam}]
```

$\text{wam}\,(2\,\text{MAa}\,(-1+\alpha\text{m})\,\phi - \text{Maa}\,\psi) + \text{wAm}\,(\text{MAA}\,\phi + 2\,\text{MAa}\,\alpha\text{m}\,\psi) < 0$

which is the same as $\gamma_{(A)} > 0$ as stated in the main text.

This is the same as ensuring the marginal fitness of Y-a is greater than the marginal fitness of Y-A, as stated in the main text,

```
wam (pXf (1 - αDm) wAf MAa + (1 - pXf) waf Maa)
----------------------------------------------- > 1 /. equilA0 /. αDm → 2 αm - 1 /.
wAm (pXf wAf MAA + (1 - pXf) waf (1 + αDm) MAa)
    αDf → 2 αf - 1 // Simplify;
0 > wAm (pXf wAf MAA + (1 - pXf) waf (1 + αDm) MAa) -
        wam (pXf (1 - αDm) wAf MAa + (1 - pXf) waf Maa) /.
      equilA0 /. αDm → 2 αm - 1 /. αDf → 2 αf - 1 // Simplify;
% /. Faa → (Faa /. Solve[ϕ == αf wAf FAa (wam Maa + 2 αm wAm MAa) - wam waf Maa Faa, Faa]) //
   Simplify;
% /. FAA → (FAA /. Solve[ψ == (1 - αf) waf FAa (wam Maa + 2 αm wAm MAa) - 2 αm wAm wAf MAa FAA,
     FAA]) // Simplify
```

{{waf wAf (waf ϕ + wAf ψ) (MAA wAm ϕ − Maa wam ψ + 2 MAa (wam (−1 + αm) ϕ + wAm αm ψ))}} < 0

Stability of equilB (r ~ 0)

The eigenvalues are

```
eigenB = Solve[0 == charpolyIntFullr0 /. equilB0, λ] // Simplify
```

$$\left\{\left\{\lambda \to \frac{\text{MAA wAm}}{2\ \text{MAa wam} - 2\ \text{MAa wam}\ \alpha\text{m}}\right\}, \left\{\lambda \to -\frac{1}{2\ \text{FAA MAa wAf wAm}\ \alpha\text{m}}\left(\text{FAa MAa waf wAm}\ (-1+\alpha\text{f})\ \alpha\text{m} + \sqrt{\text{FAa MAa waf wAm}\ (-1+\alpha\text{f})\ \alpha\text{m}\ (-2\ \text{FAA Maa wAf wam} + \text{FAa MAa waf wAm}\ (-1+\alpha\text{f})\ \alpha\text{m})}\right)\right\},$$
$$\left\{\lambda \to \frac{1}{2\ \text{FAA MAa wAf wAm}\ \alpha\text{m}}\left(-\text{FAa MAa waf wAm}\ (-1+\alpha\text{f})\ \alpha\text{m} + \sqrt{\text{FAa MAa waf wAm}\ (-1+\alpha\text{f})\ \alpha\text{m}\ (-2\ \text{FAA Maa wAf wam} + \text{FAa MAa waf wAm}\ (-1+\alpha\text{f})\ \alpha\text{m})}\right)\right\}\right\}$$

as can be seen from the Jacobian

```
matIntFull /. r → 0 /. equilB0 // Factor;
% // MatrixForm
```

$$\begin{pmatrix} -\frac{\text{FAa waf}\ (-1+\alpha\text{f})}{\text{FAA wAf}} & -\frac{\text{FAa wam}\ (-1+\alpha\text{f})}{\text{FAA wAm}} & 0 \\ \frac{\text{Maa waf}}{2\ \text{MAa wAf}\ \alpha\text{m}} & 0 & 0 \\ 0 & 0 & -\frac{\text{MAA wAm}}{2\ \text{MAa wam}\ (-1+\alpha\text{m})} \end{pmatrix}$$

I.e., one eigenvalue is $\frac{\text{MAA wAm}}{2\ \text{MAa wam}\ (1-\alpha\text{m})}$ and the other two are the roots of a 2x2.

Equilibrium B is locally stable when all eigenvalues are less than one in magnitude. We are guaranteed that the eigenvalues are real and that the largest of the eigenvalues is non-negative by the Perron-Frobenius theorem, given that the stability matrix is non-negative.

The last two eigenvalues are more readily interpreted by analysing the quadratic portion of the characteristic polynomial (with leading $+\lambda^2$ term):

```
charpolyB =
 Collect[Det[Table[matIntFull[[i, j]] /. r → 0 /. equilB0, {i, 1, 2}, {j, 1, 2}] -
    λ * IdentityMatrix[2]], λ, Factor]
```

$$\frac{\text{FAa Maa waf wam}\ (-1+\alpha\text{f})}{2\ \text{FAA MAa wAf wAm}\ \alpha\text{m}} + \frac{\text{FAa waf}\ (-1+\alpha\text{f})\ \lambda}{\text{FAA wAf}} + \lambda^2$$

Stability requires that the slope at λ=1 is positive and that the intercept at λ=1 is positive, otherwise one

or both of these two eigenvalues will be greater than one:

```
slopeB = D[charpolyB, λ] /. λ → 1 // Factor
```

$$\frac{-\text{FAa waf} + 2\,\text{FAA wAf} + \text{FAa waf}\,\alpha\text{f}}{\text{FAA wAf}}$$

```
interceptB = charpolyB /. λ → 1 // Factor
```

$$\frac{1}{2\,\text{FAA MAa wAf wAm}\,\alpha\text{m}}(-\text{FAa Maa waf wam} + \text{FAa Maa waf wam}\,\alpha\text{f} - 2\,\text{FAa MAa waf wAm}\,\alpha\text{m} + 2\,\text{FAA MAa wAf wAm}\,\alpha\text{m} + 2\,\text{FAa MAa waf wAm}\,\alpha\text{f}\,\alpha\text{m})$$

slopeB is strictly greater than interceptB, so both will be positive if interceptB is positive:

```
slopeB - interceptB // Factor
```

$$\frac{\text{FAa Maa waf wam} - \text{FAa Maa waf wam}\,\alpha\text{f} + 2\,\text{FAA MAa wAf wAm}\,\alpha\text{m}}{2\,\text{FAA MAa wAf wAm}\,\alpha\text{m}}$$

Thus, for equilibrium B to be stable, we must have $\frac{\text{MAA wAm}}{2\,\text{MAa wam}\,(1-\alpha\text{m})} < 1$ and $\text{FAA} > \frac{\text{FAa waf}\,(1-\alpha\text{f})\,(\text{Maa wam}+2\,\text{MAa wAm}\,\alpha\text{m})}{2\,\text{MAa wAf wAm}\,\alpha\text{m}}$:

```
stabcondB =
  Reduce[{MAA wAm / (2 MAa wam (1 - αm)) < 1, interceptB > 0, simpcond}, {MAa, Maa, FAA}] // Factor
```

$$0 \le \text{Rf} \le \frac{1}{2}\ \&\&\ \text{Faa} > 0\ \&\&\ 0 \le \text{Rm} \le \frac{1}{2}\ \&\&\ 0 < \alpha\text{m} < 1\ \&\&\ 0 < \alpha\text{f} < 1\ \&\&\ \text{wAm} > 0\ \&\&$$
$$\text{wam} > 0\ \&\&\ \text{wAf} > 0\ \&\&\ \text{waf} > 0\ \&\&\ \text{MAA} > 0\ \&\&\ \text{FAa} > 0\ \&\&\ \text{MAa} > -\frac{\text{MAA wAm}}{2\,\text{wam}\,(-1+\alpha\text{m})}\ \&\&$$
$$\text{Maa} > 0\ \&\&\ \text{FAA} > -\frac{\text{FAa waf}\,(-1+\alpha\text{f})\,(\text{Maa wam} + 2\,\text{MAa wAm}\,\alpha\text{m})}{2\,\text{MAa wAf wAm}\,\alpha\text{m}}$$

That the first eigenvalue is less than one is the same as saying $\gamma_{(B)} > 0$, or that the marginal fitness of Y-a is greater than the marginal fitness of Y-A, as stated in the main text

```
wam (pXf (1 - αDm) wAf MAa + (1 - pXf) waf Maa) / (wAm (pXf wAf MAA + (1 - pXf) waf (1 + αDm) MAa)) > 1 /. equilB0 /. αDm → 2 αm - 1 /.
   αDf → 2 αf - 1 // Simplify;
0 > wAm (pXf wAf MAA + (1 - pXf) waf (1 + αDm) MAa) -
      wam (pXf (1 - αDm) wAf MAa + (1 - pXf) waf Maa) /.
    equilB0 /. αDm → 2 αm - 1 /. αDf → 2 αf - 1 // Simplify;
% /. Faa → (Faa /. Solve[ϕ == αf wAf FAa (wam Maa + 2 αm wAm MAa) - wam waf Maa Faa, Faa]) //
  Simplify;
% /. FAA → (FAA /. Solve[ψ == (1 - αf) waf FAa (wam Maa + 2 αm wAm MAa) - 2 αm wAm wAf MAa FAA,
     FAA]) // Simplify
```

$$\text{wAf}\,(\text{MAA wAm} + 2\,\text{MAa wam}\,(-1+\alpha\text{m})) < 0$$

Also notice that ψ must be negative for equilB to be valid and stable

```
Reduce[{(1 - αf) waf FAa (wam Maa + 2 αm wAm MAa) - 2 αm wAm wAf MAa FAA < 0, simpcond},
  FAA] // Factor
```

$$MAA > 0 \,\&\&\, 0 \le Rf \le \frac{1}{2} \,\&\&\, Faa > 0 \,\&\&\, 0 \le Rm \le \frac{1}{2} \,\&\&\, 0 < \alpha m < 1 \,\&\&\, 0 < \alpha f < 1 \,\&\&\, wAm > 0 \,\&\&\, wam > 0 \,\&\&\, Maa > 0 \,\&\&\, MAa > 0 \,\&\&\, wAf > 0 \,\&\&\, waf > 0 \,\&\&\, FAa > 0 \,\&\&\, FAA > -\frac{FAa\ waf\ (-1+\alpha f)\ (Maa\ wam + 2\ MAa\ wAm\ \alpha m)}{2\ MAa\ wAf\ wAm\ \alpha m}$$

```
Reduce[{interceptB > 0, simpcond}, FAA] // Factor
```

$$MAA > 0 \,\&\&\, 0 \le Rf \le \frac{1}{2} \,\&\&\, Faa > 0 \,\&\&\, 0 \le Rm \le \frac{1}{2} \,\&\&\, 0 < \alpha m < 1 \,\&\&\, 0 < \alpha f < 1 \,\&\&\, wAm > 0 \,\&\&\, wam > 0 \,\&\&\, Maa > 0 \,\&\&\, MAa > 0 \,\&\&\, wAf > 0 \,\&\&\, waf > 0 \,\&\&\, FAa > 0 \,\&\&\, FAA > -\frac{FAa\ waf\ (-1+\alpha f)\ (Maa\ wam + 2\ MAa\ wAm\ \alpha m)}{2\ MAa\ wAf\ wAm\ \alpha m}$$

Stability of equilC (r ~ 0)

The three allele frequencies at this equilibrium are

```
eq1 = pXf /. equilC0 // Simplify
```

$$\frac{waf\ (Maa\ wam - 2\ MAa\ wAm\ \alpha m)}{Maa\ waf\ wam + MAA\ wAf\ wAm + 2\ MAa\ (-wAf\ wam + wAf\ wam\ \alpha m - waf\ wAm\ \alpha m)}$$

```
eq2 = pXm /. equilC0 // Simplify
```

$$(wam\ (Maa\ wam - 2\ MAa\ wAm\ \alpha m)\ (-Faa\ waf\ (MAA\ wAm + 2\ MAa\ wam\ (-1+\alpha m)) + FAa\ (Maa\ waf\ wam\ (-1+\alpha f) + MAA\ wAf\ wAm\ \alpha f + 2\ MAa\ (wAf\ wam\ \alpha f\ (-1+\alpha m) - waf\ wAm\ (-1+\alpha f)\ \alpha m)))) \Big/ \Big(-(Faa\ waf\ wam + FAA\ wAf\ wAm)\ (MAA\ wAm + 2\ MAa\ wam\ (-1+\alpha m))\ (Maa\ wam - 2\ MAa\ wAm\ \alpha m) + FAa\ (Maa\ waf\ wam\ (-1+\alpha f) + MAA\ wAf\ wAm\ \alpha f + 2\ MAa\ (wAf\ wam\ \alpha f\ (-1+\alpha m) - waf\ wAm\ (-1+\alpha f)\ \alpha m))\ \big(Maa\ wam^2 - wAm\ (MAA\ wAm + 2\ MAa\ wam\ (-1+2\ \alpha m))\big)\Big)$$

```
eq3 = pYm /. equilC0 // Simplify
```

$$(wam\ (Faa\ Maa\ waf\ wam\ (MAA\ wAm + 2\ MAa\ wam\ (-1+\alpha m)) + 2\ FAA\ MAa\ wAf\ wAm\ \alpha m\ (-Maa\ wam + 2\ MAa\ wAm\ \alpha m) - FAa\ (Maa\ wam + 2\ MAa\ wAm\ \alpha m)\ (Maa\ waf\ wam\ (-1+\alpha f) + MAA\ wAf\ wAm\ \alpha f + 2\ MAa\ (wAf\ wam\ \alpha f\ (-1+\alpha m) - waf\ wAm\ (-1+\alpha f)\ \alpha m)))) \Big/ \Big(Faa\ waf\ wam\ (MAA\ wAm + 2\ MAa\ wam\ (-1+\alpha m))\ (Maa\ wam + 2\ MAa\ wAm\ (-1+\alpha m)) + FAA\ wAf\ wAm\ (MAA\ wAm - 2\ MAa\ wam\ \alpha m)\ (Maa\ wam - 2\ MAa\ wAm\ \alpha m) - FAa\ \big(Maa\ wam^2 - wAm\ (MAA\ wAm + Maa\ wam\ (2-4\ \alpha m))\big)\ (Maa\ waf\ wam\ (-1+\alpha f) + MAA\ wAf\ wAm\ \alpha f + 2\ MAa\ (wAf\ wam\ \alpha f\ (-1+\alpha m) - waf\ wAm\ (-1+\alpha f)\ \alpha m))\Big)$$

For pXf to lie between 0 and 1 requires that:

$$pXf = \frac{waf\ (Maa\ wam - 2\ MAa\ wAm\ \alpha m)}{Maa\ waf\ wam + MAA\ wAf\ wAm + 2\ MAa\ (-wAf\ wam + wAf\ wam\ \alpha m - waf\ wAm\ \alpha m)} > 0$$

$$1 - pXf = \frac{wAf\ (MAA\ wAm + 2\ MAa\ wam\ (-1+\alpha m))}{Maa\ waf\ wam + MAA\ wAf\ wAm + 2\ MAa\ (-wAf\ wam + wAf\ wam\ \alpha m - waf\ wAm\ \alpha m)} > 0$$

so that either the numerators and denominators are both positive (without haploid selection, requires overdominance in males) or both negative (without haploid selection, requires underdominance in males). Thus the equilibrium will not be valid if $\frac{\text{MAA wAm}}{2\,\text{wam}\,(1-\alpha\text{m})} > \text{MAa} > \frac{\text{Maa wam}}{2\,\text{wAm}\,\alpha\text{m}}$ or if $\frac{\text{MAA wAm}}{2\,\text{wam}\,(1-\alpha\text{m})} < \text{MAa} < \frac{\text{Maa wam}}{2\,\text{wAm}\,\alpha\text{m}}$.

Evaluating the characteristic polynomial at $\lambda = 1$ (if this is negative, we know the leading eigenvalue is greater than one because the leading term of the characteristic polynomial is λ^3 and so it must eventually cross the horizontal axis at some $\lambda>1$):

```
stab = Factor[Det[IdentityMatrix[3] - matIntFull] /. r → 0 /. equilC0]
```

$$\begin{aligned}
&\Big(\big(-2\,\text{MAa wam} + \text{MAA wAm} + 2\,\text{MAa wam}\,\alpha\text{m}\big)\,\big(\text{Maa wam} - 2\,\text{MAa wAm}\,\alpha\text{m}\big)\\
&\quad\big(2\,\text{FAa Maa MAa waf wam}^2 - 4\,\text{Faa MAa}^2\,\text{waf wam}^2 + \text{FAa Maa MAA waf wam wAm} +\\
&\qquad 2\,\text{Faa MAa MAA waf wam wAm} - \text{FAA Maa MAA wAf wam wAm} - 2\,\text{FAa Maa MAa waf wam}^2\,\alpha\text{f} +\\
&\qquad 4\,\text{FAa MAa}^2\,\text{wAf wam}^2\,\alpha\text{f} - \text{FAa Maa MAA waf wam wAm}\,\alpha\text{f} - \text{FAa MAA}^2\,\text{wAf wAm}^2\,\alpha\text{f} -\\
&\qquad 2\,\text{FAa Maa MAa waf wam}^2\,\alpha\text{m} + 8\,\text{Faa MAa}^2\,\text{waf wam}^2\,\alpha\text{m} - 4\,\text{FAa MAa}^2\,\text{waf wam wAm}\,\alpha\text{m} -\\
&\qquad 2\,\text{Faa MAa MAA waf wam wAm}\,\alpha\text{m} - 2\,\text{FAa MAa MAA waf wAm}^2\,\alpha\text{m} + 2\,\text{FAA MAa MAA wAf wAm}^2\,\alpha\text{m} +\\
&\qquad 2\,\text{FAa Maa MAa waf wam}^2\,\alpha\text{f}\,\alpha\text{m} - 8\,\text{FAa MAa}^2\,\text{wAf wam}^2\,\alpha\text{f}\,\alpha\text{m} + 4\,\text{FAa MAa}^2\,\text{waf wam wAm}\,\alpha\text{f}\,\alpha\text{m} +\\
&\qquad 2\,\text{FAa MAa MAA waf wAm}^2\,\alpha\text{f}\,\alpha\text{m} - 4\,\text{Faa MAa}^2\,\text{waf wam}^2\,\alpha\text{m}^2 + 4\,\text{FAa MAa}^2\,\text{waf wam wAm}\,\alpha\text{m}^2 +\\
&\qquad 4\,\text{FAa MAa}^2\,\text{wAf wam}^2\,\alpha\text{f}\,\alpha\text{m}^2 - 4\,\text{FAa MAa}^2\,\text{waf wam wAm}\,\alpha\text{f}\,\alpha\text{m}^2\big)\\
&\quad\big(-\text{FAa Maa}^2\,\text{waf wam}^2 + 2\,\text{Faa Maa MAa waf wam}^2 - \text{Faa Maa MAA waf wam wAm} +\\
&\qquad \text{FAa Maa}^2\,\text{waf wam}^2\,\alpha\text{f} - 2\,\text{FAa Maa MAa wAf wam}^2\,\alpha\text{f} + \text{FAa Maa MAA wAf wam wAm}\,\alpha\text{f} -\\
&\qquad 2\,\text{Faa Maa MAa waf wam}^2\,\alpha\text{m} + 2\,\text{FAA Maa MAa wAf wam wAm}\,\alpha\text{m} + 2\,\text{FAa Maa MAa wAf wam}^2\,\alpha\text{f}\,\alpha\text{m} -\\
&\qquad 4\,\text{FAa MAa}^2\,\text{wAf wam wAm}\,\alpha\text{f}\,\alpha\text{m} + 2\,\text{FAa MAa MAA wAf wAm}^2\,\alpha\text{f}\,\alpha\text{m} + 4\,\text{FAa MAa}^2\,\text{waf wAm}^2\,\alpha\text{m}^2 -\\
&\qquad 4\,\text{FAA MAa}^2\,\text{wAf wAm}^2\,\alpha\text{m}^2 + 4\,\text{FAa MAa}^2\,\text{wAf wam wAm}\,\alpha\text{f}\,\alpha\text{m}^2 - 4\,\text{FAa MAa}^2\,\text{waf wAm}^2\,\alpha\text{f}\,\alpha\text{m}^2\big)\Big)\Big/\\
&\Big(\text{waf wAf wam}^2\,\text{wAm}^2\,\big(\text{Maa MAA} - 4\,\text{MAa}^2\,\alpha\text{m} + 4\,\text{MAa}^2\,\alpha\text{m}^2\big)^2\\
&\quad\big(-\text{FAa FAA Maa}^2\,\text{wam}^2 + 2\,\text{Faa FAA Maa MAa wam}^2 - \text{Faa FAA Maa MAA wam wAm} +\\
&\qquad \text{FAa FAA Maa}^2\,\text{wam}^2\,\alpha\text{f} - 4\,\text{Faa FAa MAa}^2\,\text{wam}^2\,\alpha\text{f} + 4\,\text{Faa FAa MAa MAA wam wAm}\,\alpha\text{f} -\\
&\qquad \text{Faa FAa MAA}^2\,\text{wAm}^2\,\alpha\text{f} - 2\,\text{Faa FAA Maa MAa wam}^2\,\alpha\text{m} + 4\,\text{FAa FAA Maa MAa wam wAm}\,\alpha\text{m} -\\
&\qquad 4\,\text{Faa FAA MAa}^2\,\text{wam wAm}\,\alpha\text{m} + 2\,\text{Faa FAA MAa MAA wAm}^2\,\alpha\text{m} +\\
&\qquad 8\,\text{Faa FAa MAa}^2\,\text{wam}^2\,\alpha\text{f}\,\alpha\text{m} - 4\,\text{FAa FAA Maa MAa wam wAm}\,\alpha\text{f}\,\alpha\text{m} -\\
&\qquad 4\,\text{Faa FAa MAa MAA wam wAm}\,\alpha\text{f}\,\alpha\text{m} + 4\,\text{Faa FAA MAa}^2\,\text{wam wAm}\,\alpha\text{m}^2 -\\
&\qquad 4\,\text{FAa FAA MAa}^2\,\text{wAm}^2\,\alpha\text{m}^2 - 4\,\text{Faa FAa MAa}^2\,\text{wam}^2\,\alpha\text{f}\,\alpha\text{m}^2 + 4\,\text{FAa FAA MAa}^2\,\text{wAm}^2\,\alpha\text{f}\,\alpha\text{m}^2\big)\Big)
\end{aligned}$$

Making a set of substitutions that might help simplify factors below:

```
subC1 = (Solve[eq1 == pXf, MAa] // Flatten)
```

$$\left\{\text{MAa} \to \frac{-\text{Maa waf wam} + \text{Maa pXf waf wam} + \text{MAA pXf wAf wAm}}{2\,(\text{pXf wAf wam} - \text{pXf wAf wam}\,\alpha\text{m} - \text{waf wAm}\,\alpha\text{m} + \text{pXf waf wAm}\,\alpha\text{m})}\right\}$$

```
subC2 = Solve[(eq2 - pXm /. subC1) == 0, FAA] // Flatten
```

$$\Big\{FAA \to \frac{1}{(-pXf + pXf^2)\ pXm\ wAf\ wAm} \big(-Faa\ pXf\ waf\ wam + Faa\ pXf^2\ waf\ wam + Faa\ pXf\ pXm\ waf\ wam - Faa\ pXf^2\ pXm\ waf\ wam - FAa\ pXf^2\ wAf\ wam + FAa\ pXf^2\ pXm\ wAf\ wam - FAa\ pXf\ pXm\ waf\ wAm + FAa\ pXf^2\ pXm\ waf\ wAm + FAa\ pXf\ wAf\ wam\ \alpha f - FAa\ pXf\ pXm\ wAf\ wam\ \alpha f + FAa\ pXm\ waf\ wAm\ \alpha f - FAa\ pXf\ pXm\ waf\ wAm\ \alpha f\big)\Big\}$$

```
subC3 = Solve[(eq3 - pYm /. subC1 /. subC2 // Factor) == 0, MAA] // Flatten
```

$$\{MAA \to (Maa\ (-1 + pXf)\ waf\ wam\ (-pXf\ pXm\ wAf\ wam + pXf\ pXm\ pYm\ wAf\ wam + pXm\ pYm\ waf\ wAm - pXf\ pXm\ pYm\ waf\ wAm - pXf\ wAf\ wam\ \alpha m + 2\ pXf\ pXm\ wAf\ wam\ \alpha m + pXf\ pYm\ wAf\ wam\ \alpha m - 2\ pXf\ pXm\ pYm\ wAf\ wam\ \alpha m + pXm\ waf\ wAm\ \alpha m - pXf\ pXm\ waf\ wAm\ \alpha m - 2\ pXm\ pYm\ waf\ wAm\ \alpha m + 2\ pXf\ pXm\ pYm\ waf\ wAm\ \alpha m)) / (pXf\ wAf\ wAm\ (pXf\ pYm\ wAf\ wam - pXf\ pXm\ pYm\ wAf\ wam - pXm\ pYm\ waf\ wAm + pXf\ pXm\ pYm\ waf\ wAm + pXf\ wAf\ wam\ \alpha m - pXf\ pXm\ wAf\ wam\ \alpha m - 2\ pXf\ pYm\ wAf\ wam\ \alpha m + 2\ pXf\ pXm\ pYm\ wAf\ wam\ \alpha m - pYm\ waf\ wAm\ \alpha m + pXf\ pYm\ waf\ wAm\ \alpha m + 2\ pXm\ pYm\ waf\ wAm\ \alpha m - 2\ pXf\ pXm\ pYm\ waf\ wAm\ \alpha m))\}$$

With these substitutions, the sign of stab reduces to:

```
stab /. subC1 /. subC2 /. subC3 // Factor;
Collect[Factor[% / ((1 - pYm) pYm wam (pXf (1 - pXm) wAf wam - pXm (1 - pXf) waf wAm)^2 /
    (pXm (pXf (1 - pYm) wAf wam - pYm (1 - pXf) waf wAm)^2
      (Faa (1 - pXf) (1 - pXm) waf wam + FAa (pXf (1 - pXm) wAf wam + pXm (1 - pXf) waf wAm )
        (1 - αf))))], {Faa, FAa, FAA}, Factor]
```

$$Faa\ (-1 + pXf)\ waf - FAa\ wAf\ (pXf - \alpha f)$$

If (pXf-αf) is positive, then stab is strictly negative and the leading eigenvalue must be greater than one.

The case where (pXf-αf) is negative is better handled by repeating the above but with a different subC2 (replacing for Faa instead):

```
subC2b = Solve[(eq2 - pXm /. subC1) == 0, Faa] // Flatten
```

$$\Big\{Faa \to \frac{1}{(-pXf + pXf^2)\ (-1 + pXm)\ waf\ wam} \big(-FAa\ pXf^2\ wAf\ wam + FAa\ pXf^2\ pXm\ wAf\ wam - FAa\ pXf\ pXm\ waf\ wAm + FAa\ pXf^2\ pXm\ waf\ wAm + FAA\ pXf\ pXm\ wAf\ wAm - FAA\ pXf^2\ pXm\ wAf\ wAm + FAa\ pXf\ wAf\ wam\ \alpha f - FAa\ pXf\ pXm\ wAf\ wam\ \alpha f + FAa\ pXm\ waf\ wAm\ \alpha f - FAa\ pXf\ pXm\ waf\ wAm\ \alpha f\big)\Big\}$$

```
subC3b = Solve[(eq3 - pYm /. subC1 /. subC2b // Factor) == 0, MAA] // Flatten
```

```
{MAA →
  (Maa (-1 + pXf) waf wam (-pXf pXm wAf wam + pXf pXm pYm wAf wam + pXm pYm waf wAm - pXf pXm
          pYm waf wAm - pXf wAf wam αm + 2 pXf pXm wAf wam αm + pXf pYm wAf wam αm -
        2 pXf pXm pYm wAf wam αm + pXm waf wAm αm - pXf pXm waf wAm αm -
        2 pXm pYm waf wAm αm + 2 pXf pXm pYm waf wAm αm)) /
   (pXf wAf wAm (pXf pYm wAf wam - pXf pXm pYm wAf wam - pXm pYm waf wAm +
        pXf pXm pYm waf wAm + pXf wAf wam αm - pXf pXm wAf wam αm -
        2 pXf pYm wAf wam αm + 2 pXf pXm pYm wAf wam αm - pYm waf wAm αm +
        pXf pYm waf wAm αm + 2 pXm pYm waf wAm αm - 2 pXf pXm pYm waf wAm αm))}
```

```
stab /. subC1 /. subC2b /. subC3b // Factor;
Collect[
 Factor[% / ((1 - pYm) pYm wAm (pXf (1 - pXm) wAf wam - pXm (1 - pXf) waf wAm)^2 / ((1 - pXm)
        (pXf (1 - pYm) wAf wam - pYm (1 - pXf) waf wAm)^2 (FAA pXf pXm wAf wAm + FAa (pXf
              (1 - pXm) wAf wam + pXm (1 - pXf) waf wAm ) αf)))], {Faa, FAa, FAA}, Factor]
```

```
-FAA pXf wAf + FAa waf (pXf - αf)
```

Hence, if (pXf-αf) is negative, stab is also negative, and therefore the leading eigenvalue must be greater than one.

We conclude that in no case can equilC0 represent a stable equilibrium.

Weak selection

Assumptions

In this section we will assume weak selection in both haploids and diploids

```
weaksel = {
   MAA → 1 + sAm ϵ,
   MAa → 1 + hAm sAm ϵ,
   Maa → 1,
   FAA → 1 + sAf ϵ,
   FAa → 1 + hAf sAf ϵ,
   Faa → 1,
   wAf → 1 + tf ϵ,
   waf → 1,
   wAm → 1 + tm ϵ,
   wam → 1,
   αm → 1 / 2 + α1m ϵ,
   αf → 1 / 2 + α1f ϵ
  };
```

All selection terms are of order ϵ. We will solve for genotype frequencies using a Taylor Series. First, we write the genotype frequencies as a series of terms, each proportional to increasing orders of ϵ (only necessary to go to ϵ^2 here), which we will then solve for.

```
frequenciessub = {
    pXf → pXf0 + pXf1 ∈ + pXf2 * ∈^2,
    pXm → pXm0 + pXm1 ∈ + pXm2 * ∈^2,
    pYm → pYm0 + pYm1 ∈ + pYm2 * ∈^2,
    q → q + q1 ∈ + q2 * ∈^2
  };
```

Stability

Instead of examining when the non-trivial equilibrium (A polymorphic) is stable we will look for when the trivial equilibria (loss or fixation of A) are unstable.

When A is fixed the characteristic polynomial with no selection gives eigenvalues

```
charpolyInt1 = charpolyIntFull /. pXm → 1 /. pXf → 1 /. pYm → 1 /. q → 1 / 2;
Normal[Series[charpolyInt1 /. weaksel, {∈, 0, 0}]] // Factor;
Solve[% == 0, λ] // Factor // Flatten
```

$$\left\{\lambda \to 1,\ \lambda \to \frac{1}{4}\left(1 - 2\,r - \sqrt{9 - 20\,r + 4\,r^2}\right),\ \lambda \to \frac{1}{4}\left(1 - 2\,r + \sqrt{9 - 20\,r + 4\,r^2}\right)\right\}$$

and λ=1 is the leading eigenvalue.

With weak selection first order term in the eigenvalues is

```
Normal[Series[charpolyInt1 /. weaksel /. λ → 1 + δλ1 * ∈, {∈, 0, 1}]] /. ∈ → 1 // Factor;
δλ1fix = Solve[% == 0, δλ1] // Flatten
```

$$\left\{\delta\lambda 1 \to \frac{1}{2}\left(-sAf + hAf\ sAf - sAm + hAm\ sAm - tf - tm - 2\,\alpha 1f - 2\,\alpha 1m\right)\right\}$$

And so the fixation of A is unstable when this expression is positive.

When A is lost the characteristic polynomial with no selection gives eigenvalues

```
charpolyInt0 = charpolyIntFull /. pXm → 0 /. pXf → 0 /. pYm → 0 /. q → 1 / 2;
Normal[Series[charpolyInt0 /. weaksel, {∈, 0, 0}]] // Factor;
Solve[% == 0, λ] // Flatten
```

$$\left\{\lambda \to 1,\ \lambda \to \frac{1}{4}\left(1 - 2\,r - \sqrt{9 - 20\,r + 4\,r^2}\right),\ \lambda \to \frac{1}{4}\left(1 - 2\,r + \sqrt{9 - 20\,r + 4\,r^2}\right)\right\}$$

With weak selection first order term in the eigenvalues is

```
Normal[Series[charpolyInt0 /. weaksel /. λ → 1 + δλ1 * ∈, {∈, 0, 1}]] /. ∈ → 1 // Factor;
δλ1loss = Solve[% == 0, δλ1] // Flatten
```

$$\left\{\delta\lambda 1 \to \frac{1}{2}\left(hAf\ sAf + hAm\ sAm + tf + tm + 2\,\alpha 1f + 2\,\alpha 1m\right)\right\}$$

And so the loss of A is unstable when this expression is positive.

Combining the two results, the protected polymorphism (non-trivial equilibrium) requires that

```
stabconds = {(δλ1 /. δλ1fix) > 0, (δλ1 /. δλ1loss) > 0};
% /. α1f → αDf / 2 /. α1m → αDm / 2
```

$$\left\{\frac{1}{2}\left(-\text{sAf}+\text{hAf sAf}-\text{sAm}+\text{hAm sAm}-\text{tf}-\text{tm}-\alpha\text{Df}-\alpha\text{Dm}\right)>0,\ \frac{1}{2}\left(\text{hAf sAf}+\text{hAm sAm}+\text{tf}+\text{tm}+\alpha\text{Df}+\alpha\text{Dm}\right)>0\right\}$$

Equilibria

Without selection the recursions for the change in the frequencies are

```
differenceEqs0 = Normal[Series[differenceEqs /. weaksel /. frequenciessub, {ϵ, 0, 0}]]
```

$$\left\{\frac{1}{2}\left(-\text{pXf0}+\text{pXm0}\right),\ \frac{1}{2}\left(\text{pXf0}-2\,\text{pXm0}+2\,\text{pXm0}\,q-\text{pXf0}\,r+\text{pYm0}\,r\right),\ \frac{1}{2}\left(\text{pYm0}-2\,\text{pYm0}\,q+\text{pXf0}\,r-\text{pYm0}\,r\right),\ \frac{1}{2}\left(1-2\,q\right)\right\}$$

And at equilibrium we have

```
sol0 = Solve[differenceEqs0 == 0, {pYm0, pXm0, q}]
```

$$\left\{\left\{\text{pYm0}\to\text{pXf0},\ \text{pXm0}\to\text{pXf0},\ q\to\frac{1}{2}\right\}\right\}$$

That is, to leading order (O(1)), there is no difference in A allele frequency between gamete types (pYm0=pXm0=pXf0) and 50% of male gametes are Y-bearing (q=1/2). We call the leading order frequency of A alleles pA.

At the next order we have

```
differenceEqs1 =
  Collect[Normal[Series[differenceEqs /. weaksel /. frequenciessub /. sol0,
      {ϵ, 0, 1}]], ϵ, Factor] /. pXf0 → pA // Flatten
```

$$\left\{\frac{1}{2}\left(-\text{pXf1}+\text{pXm1}+2\,\text{hAf pA sAf}+2\,\text{pA}^2\,\text{sAf}-6\,\text{hAf pA}^2\,\text{sAf}-2\,\text{pA}^3\,\text{sAf}+4\,\text{hAf pA}^3\,\text{sAf}+\text{pA tf}-\text{pA}^2\,\text{tf}+\text{pA tm}-\text{pA}^2\,\text{tm}+4\,\text{pA}\,\alpha\text{1f}-4\,\text{pA}^2\,\alpha\text{1f}\right)\epsilon,\right.$$

$$\frac{1}{2}\left(\text{pXf1}-\text{pXm1}+2\,\text{pA q1}-\text{pXf1 r}+\text{pYm1 r}+\text{hAm pA sAm}+\text{pA}^2\,\text{sAm}-3\,\text{hAm pA}^2\,\text{sAm}-\text{pA}^3\,\text{sAm}+2\,\text{hAm pA}^3\,\text{sAm}+\text{pA tf}-\text{pA}^2\,\text{tf}-\text{pA r tf}+\text{pA}^2\,\text{r tf}+\text{pA r tm}-\text{pA}^2\,\text{r tm}+2\,\text{pA}\,\alpha\text{1m}-2\,\text{pA}^2\,\alpha\text{1m}\right)\epsilon,$$

$$\left.\frac{1}{2}\left(-2\,\text{pA q1}+\text{pXf1 r}-\text{pYm1 r}+\text{hAm pA sAm}+\text{pA}^2\,\text{sAm}-3\,\text{hAm pA}^2\,\text{sAm}-\text{pA}^3\,\text{sAm}+2\,\text{hAm pA}^3\,\text{sAm}+\text{pA r tf}-\text{pA}^2\,\text{r tf}+\text{pA tm}-\text{pA}^2\,\text{tm}-\text{pA r tm}+\text{pA}^2\,\text{r tm}+2\,\text{pA}\,\alpha\text{1m}-2\,\text{pA}^2\,\alpha\text{1m}\right)\epsilon,\ -\text{q1}\,\epsilon\right\}$$

And we can solve for the first order difference in the frequency of A on Xs in males vs females, dpAXmXf , and on Ys in males vs Xs in females, dpAYmXf

```
realsol = Solve[differenceEqs1 == 0 /. pXm1 → dpAXmXf + pXf1 /. pYm1 → dpAYmXf + pXf1,
    {dpAXmXf, dpAYmXf, pA, q1}] // Factor
```

$$\Big\{\{dpAXmXf \to 0, dpAYmXf \to 0, pA \to 0, q1 \to 0\}, \{dpAXmXf \to 0, dpAYmXf \to 0, pA \to 1, q1 \to 0\},$$

$$\Big\{dpAXmXf \to \frac{1}{(-sAf + 2\,hAf\,sAf - sAm + 2\,hAm\,sAm)^3} (-sAf + hAf\,sAf - sAm + hAm\,sAm - tf - tm - 2\,\alpha1f - 2\,\alpha1m) (hAf\,sAf + hAm\,sAm + tf + tm + 2\,\alpha1f + 2\,\alpha1m) (2\,hAf\,sAf\,sAm - 2\,hAm\,sAf\,sAm - sAf\,tf + 2\,hAf\,sAf\,tf + sAm\,tf - 2\,hAm\,sAm\,tf - sAf\,tm + 2\,hAf\,sAf\,tm + sAm\,tm - 2\,hAm\,sAm\,tm + 4\,sAm\,\alpha1f - 8\,hAm\,sAm\,\alpha1f - 4\,sAf\,\alpha1m + 8\,hAf\,sAf\,\alpha1m),$$

$$dpAYmXf \to \frac{1}{r\,(-sAf + 2\,hAf\,sAf - sAm + 2\,hAm\,sAm)^3} (-sAf + hAf\,sAf - sAm + hAm\,sAm - tf - tm - 2\,\alpha1f - 2\,\alpha1m) (hAf\,sAf + hAm\,sAm + tf + tm + 2\,\alpha1f + 2\,\alpha1m) (hAf\,sAf\,sAm - hAm\,sAf\,sAm - r\,sAf\,tf + 2\,hAf\,r\,sAf\,tf + sAm\,tf - 2\,hAm\,sAm\,tf - r\,sAm\,tf + 2\,hAm\,r\,sAm\,tf - sAf\,tm + 2\,hAf\,sAf\,tm + r\,sAf\,tm - 2\,hAf\,r\,sAf\,tm + r\,sAm\,tm - 2\,hAm\,r\,sAm\,tm + 2\,sAm\,\alpha1f - 4\,hAm\,sAm\,\alpha1f - 2\,sAf\,\alpha1m + 4\,hAf\,sAf\,\alpha1m),$$

$$pA \to \frac{hAf\,sAf + hAm\,sAm + tf + tm + 2\,\alpha1f + 2\,\alpha1m}{-sAf + 2\,hAf\,sAf - sAm + 2\,hAm\,sAm}, q1 \to 0\Big\}\Big\}$$

Alternatively we could look at the difference in the frequency of A on Xs in males vs females, dpAXmXf , and on Ys in males vs Xs in males, dpAYmXm

```
realsol2 = Solve[differenceEqs1 == 0 /. pXf1 → dpAXfXm + pXm1 /. pYm1 → dpAYmXm + pXm1,
    {dpAXfXm, dpAYmXm, pA, q1}] // Factor;
```

Given that there is a polymorphs at the selected locus, the zeroth order frequency of A on all backgrounds, pA, can be written

```
pA /. realsol[[3]] /. α1f → αDf / 2 /. α1m → αDm / 2
```

$$\frac{hAf\,sAf + hAm\,sAm + tf + tm + \alpha Df + \alpha Dm}{-sAf + 2\,hAf\,sAf - sAm + 2\,hAm\,sAm}$$

The first order differences can be re-written in a more readable form using the following terms

```
trysub3 = {Df → (pA sAf + (1 - pA) hAf sAf) - (pA hAf sAf + (1 - pA)),
    Dm → (pA sAm + (1 - pA) hAm sAm) - (pA hAm sAm + (1 - pA)),
    VA → pA (1 - pA), αDm → 2 α1m, αDf → 2 α1f};
```

The difference in frequency of A between Xs in males and Xs in females is then

```
VA (Dm - Df + αDm - αDf) /. trysub3;
(% == dpAXmXf) /. realsol[[3]] // Simplify
```

```
True
```

The difference in frequency of A between Ys in males and Xs in females is then

```
VA (Dm - Df + αDm - αDf + (1 - 2 r) (tm - tf)) / (2 r) /. trysub3;
(% == dpAYmXf) /. realsol[[3]] // Simplify
```

```
True
```

And the difference in frequency of A between Ys in males and Xs in males is then

```
VA (Dm - Df + αDm - αDf + tm - tf) (1 - 2 r) / (2 r) /. trysub3;
(% == dpAYmXm) /. realsol2[[3]] // Simplify
```

```
True
```

To get the frequency of Y bearing male gametes we need to go to the next order of selection (takes a minute)

```
differenceEqs2freqY =
  Collect[Normal[Series[differenceEqs[[4]] /. weaksel /. frequenciessub /. sol0 /.
          pXf0 → pA /. pXm1 → (dpAXmXf + pXf1) /. pYm1 → dpAYmXf + pXf1 /.
        realsol[[3]], {ϵ, 0, 2}]], ϵ, Factor] // Flatten;
```

And we can solve for the leading order Y bias in males

```
freqYm2sol = Solve[differenceEqs2freqY == 0, {q2}] // Flatten // Simplify;
```

which is simply α1m times the difference in frequency of A between Y-bearing and X-bearing male gametes

```
α1m dpAYmXm  == q2 /. freqYm2sol /. realsol2[[3]] // Factor
```

```
True
```

Finally, van Doorn and Kirkpatrick 2007 (SOM, page 6) give the first order frequency of pA as

$$\text{vDK07} = \frac{\text{hAf sAf} + \text{hAm sAm}}{(-1 + 2\,\text{hAf})\,\text{sAf} + (-1 + 2\,\text{hAm})\,\text{sAm}};$$

And when there is no haploid selection or drive our result reduces to theirs

```
(pA /. realsol[[3]] /. tf → 0 /. tm → 0 /. α1f → 0 /. α1m → 0) == vDK07 // FullSimplify
```

```
True
```

Invasion

General

Characteristic polynomial

The Jacobian for the mutants is

```
eqsmut = {nextXAmf, nextXamf, nextYAmf,
    nextYamf, nextXAmm, nextXamm, nextYAmm, nextYamm} /. SUBS;
matExtFull = Transpose[{D[eqsmut, XAmf], D[eqsmut, Xamf], D[eqsmut, YAmf],
      D[eqsmut, Yamf], D[eqsmut, XAmm], D[eqsmut, Xamm],
      D[eqsmut, YAmm], D[eqsmut, Yamm]}] /. subequil // Factor;
```

Giving characteristic polynomial

```
charpolyExt = Det[matExtFull - IdentityMatrix[8] * λ];
```

Note that for a neo-W invading an XY system, the Jacobian is not in block triangular form:

```
PossibleZeroQ[matExtFull /. k → 1] /. True → 0 /. False → 1 // MatrixForm
```

$$\begin{pmatrix} 1&1&1&1&1&1&1&1\\ 1&1&1&1&1&1&1&1\\ 1&1&1&1&0&0&1&1\\ 1&1&1&1&0&0&1&1\\ 0&0&0&0&0&0&0&0\\ 0&0&0&0&0&0&0&0\\ 0&0&0&0&0&0&0&0\\ 0&0&0&0&0&0&0&0 \end{pmatrix}$$

But it is for a neo-Y

```
PossibleZeroQ[matExtFull /. k → 0] /. True → 0 /. False → 1 // MatrixForm
```

$$\begin{pmatrix} 0&0&0&0&0&0&0&0\\ 0&0&0&0&0&0&0&0\\ 0&0&0&0&0&0&0&0\\ 0&0&0&0&0&0&0&0\\ 1&1&1&1&1&1&1&1\\ 1&1&1&1&1&1&1&1\\ 1&1&1&1&0&0&1&1\\ 1&1&1&1&0&0&1&1 \end{pmatrix}$$

The relevant quadratic

Let us instead track the sum of the mutant-selected allele haplotypes across X and Y backgrounds in each sex, XAm+YAm (call this S), as well as the difference, Xam-Yam (call this D):

```
subsumdif = Flatten[
  Solve[{SAmf == YAmf + XAmf, Samf == Yamf + Xamf, DAmf == XAmf - YAmf, Damf == Xamf - Yamf,
    SAmm == YAmm + XAmm, Samm == Yamm + Xamm, DAmm == XAmm - YAmm, Damm == Xamm - Yamm},
   {XAmf, Xamf, YAmf, Yamf, XAmm, Xamm, YAmm, Yamm}]]
```

$$\left\{XAmf \to \frac{DAmf + SAmf}{2},\ Xamf \to \frac{Damf + Samf}{2},\ YAmf \to -\frac{DAmf}{2} + \frac{SAmf}{2},\ Yamf \to -\frac{Damf}{2} + \frac{Samf}{2},\right.$$
$$\left. XAmm \to \frac{DAmm + SAmm}{2},\ Xamm \to \frac{Damm + Samm}{2},\ YAmm \to -\frac{DAmm}{2} + \frac{SAmm}{2},\ Yamm \to -\frac{Damm}{2} + \frac{Samm}{2}\right\}$$

This is just a translation of variables with no loss of information.

Calculating the Jacobian matrix for this new set of variables:

```
eqsmutNEW =
  {nextXAmf + nextYAmf, nextXamf + nextYamf, nextXAmf - nextYAmf, nextXamf - nextYamf,
    nextXAmm + nextYAmm, nextXamm + nextYamm, nextXAmm - nextYAmm,
    nextXamm - nextYamm} /. SUBS /. subsumdif;
matExtFullNEW = Transpose[{D[eqsmutNEW, SAmf], D[eqsmutNEW, Samf],
       D[eqsmutNEW, DAmf], D[eqsmutNEW, Damf], D[eqsmutNEW, SAmm],
       D[eqsmutNEW, Samm], D[eqsmutNEW, DAmm], D[eqsmutNEW, Damm]}] /. subequil /.
    {SAmf → 0, Samf → 0, DAmf → 0, Damf → 0, SAmm → 0, Samm → 0,
     DAmm → 0, Damm → 0} // Factor;
```

The advantage of this new variable system is that it now has a block (2x2) by (2x2) by (4x4) form for a neo-W into a neo-Y system (a “1” in the following means there is a potential non-zero entry):

```
PossibleZeroQ[matExtFullNEW /. k → 1] /. True → 0 /. False → 1 // MatrixForm
```

$$\begin{pmatrix} 1 & 1 & 0 & 0 & 1 & 1 & 0 & 0 \\ 1 & 1 & 0 & 0 & 1 & 1 & 0 & 0 \\ 1 & 1 & 1 & 1 & 1 & 1 & 1 & 1 \\ 1 & 1 & 1 & 1 & 1 & 1 & 1 & 1 \\ 0 & 0 & 0 & 0 & 0 & 0 & 0 & 0 \\ 0 & 0 & 0 & 0 & 0 & 0 & 0 & 0 \\ 0 & 0 & 0 & 0 & 0 & 0 & 0 & 0 \\ 0 & 0 & 0 & 0 & 0 & 0 & 0 & 0 \end{pmatrix}$$

The 2x2 in the top left describes the dynamics of the sum total frequencies of the neo-W-A and neo-W-a in females, summed over the old-X and old-Y backgrounds. The 2x2 below and right of it describes the dynamics of the difference between old-X and old-Y backgrounds in the frequencies of the neo-W-A and neo-W-a types in females.

The same thing goes for a neo-Y invading an XY system.

```
PossibleZeroQ[matExtFullNEW /. k → 0] /. True → 0 /. False → 1 // MatrixForm
```

$$\begin{pmatrix} 0 & 0 & 0 & 0 & 0 & 0 & 0 & 0 \\ 0 & 0 & 0 & 0 & 0 & 0 & 0 & 0 \\ 0 & 0 & 0 & 0 & 0 & 0 & 0 & 0 \\ 0 & 0 & 0 & 0 & 0 & 0 & 0 & 0 \\ 1 & 1 & 0 & 0 & 1 & 1 & 0 & 0 \\ 1 & 1 & 0 & 0 & 1 & 1 & 0 & 0 \\ 1 & 1 & 1 & 1 & 1 & 1 & 1 & 1 \\ 1 & 1 & 1 & 1 & 1 & 1 & 1 & 1 \end{pmatrix}$$

Now the bottom right 2x2 describes the dynamics of the difference between old-X and old-Y backgrounds in the frequencies of the neo-Y-A and neo-Y-a types in males. The 2x2 above and to the left it describes the sum total frequencies of the neo-Y-A and neo-Y-a in males, summed over the old-X and old-Y backgrounds.

Now, the difference cannot grow faster than the sum because the new mutant type is assumed rare and cannot become negative in frequency. So at the very worst, the difference might increase until one of the old-X or old-Y types becomes exceedingly rare, but at that point the difference and the sum would have to grow at the same rate. Consequently, the behaviour of the system is determined by the behaviour of the sum. We can therefore look only at the eigenvalues emerging from the 2x2 Jacobian describing the changes in the summed frequencies over X and Y backgrounds of the m-A and m-a haplotypes in the sex that m makes.

The relevant part of the characteristic polynomial for a neo-W is thus

```
{nextXAmf + nextYAmf, nextXamf + nextYamf} /. SUBS /. subsumdif;
Transpose[{D[%, SAmf], D[%, Samf]}] /. subequil /. {SAmf → 0, Samf → 0,
    DAmf → 0, Damf → 0, SAmm → 0, Samm → 0, DAmm → 0, Damm → 0} // Factor;
charpolyα1 = Collect[Det[% - IdentityMatrix[2] * λ /. k → 1], λ, Simplify]
```

```
- (waf wAf (Faa (1 + pXm (-1 + q) - pYm q) wam
        (FAA (pXm (-1 + q) - pYm q) wAm + 2 FAa (1 + pXm (-1 + q) - pYm q) (-1 + R) wam αf) +
      2 FAa (pXm (-1 + q) - pYm q) wAm (-1 + αf) (-FAA (pXm (-1 + q) - pYm q) (-1 + R) wAm +
          2 FAa (1 + pXm (-1 + q) - pYm q) (-1 + 2 R) wam αf))) /
   (4 (-1 + q)^2 (Faa (-1 + pXf) (-1 + pXm) waf wam + FAA pXf pXm wAf wAm +
         FAa (pXm waf wAm - pXf ((-1 + pXm) wAf wam + pXm waf wAm)))^2) +
  ((Faa (1 + pXm (-1 + q) - pYm q) waf wam + FAA (pXm - pXm q + pYm q) wAf wAm -
       2 FAa (-1 + R) (wAf wam αf + pXm (-1 + q) (waf wAm (-1 + αf) + wAf wam αf) -
          pYm q (waf wAm (-1 + αf) + wAf wam αf))) λ) /
   (2 (-1 + q) (Faa (-1 + pXf) (-1 + pXm) waf wam + FAA pXf pXm wAf wAm +
       FAa (pXm waf wAm - pXf ((-1 + pXm) wAf wam + pXm waf wAm)))) + λ^2
```

And the relevant part of the characteristic polynomial for a neo-W is thus

```
{nextXAmm + nextYAmm, nextXamm + nextYamm} /. SUBS /. subsumdif;
Transpose[{D[%, SAmm], D[%, Samm]}] /. subequil /. {SAmf → 0, Samf → 0,
    DAmf → 0, Damf → 0, SAmm → 0, Samm → 0, DAmm → 0, Damm → 0} // Factor;
charpolyα0 = Collect[Det[% - IdentityMatrix[2] * λ /. k → 0], λ, Simplify]
```

```
(wam wAm (-Maa (-1 + pXf) waf (MAA pXf wAf + 2 MAa (-1 + pXf) (-1 + R) waf αm) + 2 MAa pXf wAf
        (-1 + αm) (MAA pXf (-1 + R) wAf + 2 MAa (-1 + pXf + 2 R - 2 pXf R) waf αm))) /
   (4 q^2 (Maa (-1 + pXf) (-1 + pYm) waf wam + MAA pXf pYm wAf wAm +
        MAa (pYm waf wAm - pXf ((-1 + pYm) wAf wam + pYm waf wAm)))^2) +
  ((Maa (-1 + pXf) waf wam - MAA pXf wAf wAm - 2 MAa (-1 + R)
        (-waf wAm αm + pXf (wAf wam (-1 + αm) + waf wAm αm))) λ) /
   (2 q (Maa (-1 + pXf) (-1 + pYm) waf wam + MAA pXf pYm wAf wAm +
       MAa (pYm waf wAm - pXf ((-1 + pYm) wAf wam + pYm waf wAm)))) + λ^2
```

We confirmed that the alternative quadratic (from the difference instead of the sum) never determined invasion (details available upon request).

Mean fitness

Among the zygotes, the frequency of diploid females is (1-ζ):

```
freqFemale = XAMXAMfemale + XAMXaMfemale + XaMXaMfemale +
    XAMYAMfemale + (XAMYaMfemale + XaMYAMfemale) + XaMYaMfemale +
    YAMYAMfemale + YAMYaMfemale + YaMYaMfemale +
    XAmXAMfemale + XAmXaMfemale + XAMXamfemale + XamXaMfemale +
    XAmYAMfemale + XAmYaMfemale + XamYAMfemale + XamYaMfemale +
    XAMYAmfemale + XAMYamfemale + XaMYAmfemale + XaMYamfemale +
    YAmYAMfemale + YAmYaMfemale + YamYAMfemale + YamYaMfemale +
    XAmXAmfemale + XAmXamfemale + XamXamfemale +
    XAmYAmfemale + XAmYamfemale + XamYAmfemale + XamYamfemale +
    YAmYAmfemale + YAmYamfemale + YamYamfemale /. SUBS /. subequil // Factor
```

$$\frac{(-1+q)\,(-\text{wam}+\text{pXm wam}-\text{pXm wAm})}{\text{wam}-\text{pXm wam}+\text{pXm q wam}-\text{pYm q wam}+\text{pXm wAm}-\text{pXm q wAm}+\text{pYm q wAm}}$$

The frequency of diploid male zygotes is then ζ, as given in Table S.2:

```
freqMale = XAMXAMmale + XAMXaMmale + XaMXaMmale +
    XAMYAMmale + (XAMYaMmale + XaMYAMmale) + XaMYaMmale +
    YAMYAMmale + YAMYaMmale + YaMYaMmale +
    XAmXAMmale + XAmXaMmale + XAMXammale + XamXaMmale +
    XAmYAMmale + XAmYaMmale + XamYAMmale + XamYaMmale +
    XAMYAmmale + XAMYammale + XaMYAmmale + XaMYammale +
    YAmYAMmale + YAmYaMmale + YamYAMmale + YamYaMmale +
    XAmXAmmale + XAmXammale + XamXammale +
    XAmYAmmale + XAmYammale + XamYAmmale + XamYammale +
    YAmYAmmale + YAmYammale + YamYammale /. SUBS /. subequil // Factor
```

$$\frac{q\ (-wam + pYm\ wam - pYm\ wAm)}{-wam + pXm\ wam - pXm\ q\ wam + pYm\ q\ wam - pXm\ wAm + pXm\ q\ wAm - pYm\ q\ wAm}$$

As a check the frequency of females and males sum to one:

```
freqFemale + freqMale // Factor
```

1

The mean fitness of a diploid female can be written by considering the chance that the female inherited "A" from the mother and either "A" or "a" from the father (first row) or "a" from the mother and either "A" or "a" from the father (second row), conditioning on the fact that an X must have been inherited from the dad (i.e., dividing by the frequency of female zygotes):

$$wbarDipFemale = pXf\left(FAA\frac{wAf}{wbarHapFemale}\left(\frac{pXm\ (1-q)\ \frac{wAm}{wbarHapMale}}{freqFemale}\right) + FAa\frac{wAf}{wbarHapFemale}\left(\frac{(1-pXm)\ (1-q)\ \frac{wam}{wbarHapMale}}{freqFemale}\right)\right) + (1-pXf)\left(FAa\frac{waf}{wbarHapFemale}\left(\frac{pXm\ (1-q)\ \frac{wAm}{wbarHapMale}}{freqFemale}\right) + Faa\frac{waf}{wbarHapFemale}\left(\frac{(1-pXm)\ (1-q)\ \frac{wam}{wbarHapMale}}{freqFemale}\right)\right)\ /.\ SUBS\ /.\ subequil\ //\ Simplify$$

```
(Faa (-1 + pXf) (-1 + pXm) waf wam + FAA pXf pXm wAf wAm +
    FAa (pXm waf wAm - pXf ((-1 + pXm) wAf wam + pXm waf wAm))) /
  (((-1 + pXf) waf - pXf wAf) ((-1 + pXm) wam - pXm wAm))
```

Similarly, the mean fitness of a diploid male can be written as:

```
wbarDipMale =
  pXf (MAA wAf/wbarHapFemale ((pYm q wAm/wbarHapMale)/freqMale) + MAa wAf/wbarHapFemale (((1 - pYm) q wam/wbarHapMale)/freqMale)) +
   (1 - pXf) (MAa waf/wbarHapFemale ((pYm q wAm/wbarHapMale)/freqMale) +
      Maa waf/wbarHapFemale (((1 - pYm) q wam/wbarHapMale)/freqMale)) /. SUBS /. subequil // Simplify
```

```
(Maa (-1 + pXf) (-1 + pYm) waf wam + MAA pXf pYm wAf wAm +
   MAa (pYm waf wAm - pXf ((-1 + pYm) wAf wam + pYm waf wAm))) /
 (((-1 + pXf) waf - pXf wAf) ((-1 + pYm) wam - pYm wAm))
```

Note that if we define mean haploid fitnesses of X and Y bearing male gametes

```
wbarHapMaleX = (1 - q) ((1 - pXm) wam + pXm wAm);
wbarHapMaleY = q ((1 - pYm) wam + pYm wAm);
```

then

```
freqFemale wbarHapMale == wbarHapMaleX /. SUBS /. subequil // Simplify
```

```
True
```

```
freqMale wbarHapMale == wbarHapMaleY /. SUBS /. subequil // Simplify
```

```
True
```

The following replaces terms used to stand in for the mean fitnesses with their formulae:

```
reverse =
  {meandipM -> wbarDipMale, meandipF -> wbarDipFemale, ξ -> freqMale, meanhapM ->
      wbarHapMale, meanhapF -> wbarHapFemale} /. SUBS /. subequil // Simplify;
```

The following removes haploid selection:

```
nohap = {wam → 1, wAm → 1, waf → 1, wAf → 1, αm → 1 / 2, αf → 1 / 2};
```

neo-Y characteristic polynomial

As shown above, the relevant part of the characteristic polynomial for a masculinizing mutant is

```
charpolyα0
```

```
(wam wAm (-Maa (-1 + pXf) waf (MAA pXf wAf + 2 MAa (-1 + pXf) (-1 + R) waf αm) + 2 MAa pXf wAf
        (-1 + αm) (MAA pXf (-1 + R) wAf + 2 MAa (-1 + pXf + 2 R - 2 pXf R) waf αm))) /
  (4 q^2 (Maa (-1 + pXf) (-1 + pYm) waf wam + MAA pXf pYm wAf wAm +
        MAa (pYm waf wAm - pXf ((-1 + pYm) wAf wam + pYm waf wAm)))^2) +
 ((Maa (-1 + pXf) waf wam - MAA pXf wAf wAm - 2 MAa (-1 + R)
        (-waf wAm αm + pXf (wAf wam (-1 + αm) + waf wAm αm))) λ) /
  (2 q (Maa (-1 + pXf) (-1 + pYm) waf wam + MAA pXf pYm wAf wAm +
       MAa (pYm waf wAm - pXf ((-1 + pYm) wAf wam + pYm waf wAm)))) + λ^2
```

The coefficients of the relevant quadratic, $\lambda^2+b\lambda+c=0$, are then:

```
acoef0 = Coefficient[charpolyα0, λ^2];
bcoef0 = Coefficient[charpolyα0, λ] / acoef0;
ccoef0 = (charpolyα0 - acoef0 λ^2 - acoef0 bcoef0 λ) / acoef0;
```

The b coefficient is

```
bcoef0 * (wbarDipMale freqMale wbarHapMale wbarHapFemale / (meandipM ξ meanhapM meanhapF)) /. SUBS /. subequil //
 Simplify
```

$$(\text{Maa}(-1+\text{pXf})\,\text{waf}\,\text{wam} - \text{MAA}\,\text{pXf}\,\text{wAf}\,\text{wAm} - 2\,\text{MAa}(-1+R)(-\text{waf}\,\text{wAm}\,\alpha\text{m} + \text{pXf}(\text{wAf}\,\text{wam}(-1+\alpha\text{m}) + \text{waf}\,\text{wAm}\,\alpha\text{m})))\,/\,(2\,\text{meandipM}\,\text{meanhapF}\,\text{meanhapM}\,\xi)$$

The c coefficient is

```
ccoef0 * (wbarDipMale freqMale wbarHapMale wbarHapFemale / (meandipM ξ meanhapM meanhapF))^2 /. SUBS /. subequil //
 Simplify
```

$$-(\text{wam}\,\text{wAm}(\text{Maa}(-1+\text{pXf})\,\text{waf}(\text{MAA}\,\text{pXf}\,\text{wAf} + 2\,\text{MAa}(-1+\text{pXf})(-1+R)\,\text{waf}\,\alpha\text{m}) + 2\,\text{MAa}\,\text{pXf}\,\text{wAf}(-1+\alpha\text{m})(-\text{MAA}\,\text{pXf}(-1+R)\,\text{wAf} + 2\,\text{MAa}(-1+\text{pXf})(-1+2R)\,\text{waf}\,\alpha\text{m})))\,/\,(4\,\text{meandipM}^2\,\text{meanhapF}^2\,\text{meanhapM}^2\,\xi^2)$$

With no recombination the eigenvalues are

```
noreceigens0 = (λ /. Solve[0 == charpolyα0 /. R → 0, λ]) *
    (wbarDipMale freqMale wbarHapMale wbarHapFemale / (meandipM ξ meanhapM meanhapF)) /.
   SUBS /. subequil // Factor // Simplify
```

$$\left\{-\frac{\text{wam}(\text{Maa}(-1+\text{pXf})\,\text{waf} + 2\,\text{MAa}\,\text{pXf}\,\text{wAf}(-1+\alpha\text{m}))}{2\,\text{meandipM}\,\text{meanhapF}\,\text{meanhapM}\,\xi},\ \frac{\text{wAm}(\text{MAA}\,\text{pXf}\,\text{wAf} - 2\,\text{MAa}(-1+\text{pXf})\,\text{waf}\,\alpha\text{m})}{2\,\text{meandipM}\,\text{meanhapF}\,\text{meanhapM}\,\xi}\right\}$$

Define the no recombination eigenvalues

```
λmA0 = noreceigens0[[2]]
λma0 = noreceigens0[[1]]
```

$$\frac{\text{wAm}(\text{MAA}\,\text{pXf}\,\text{wAf} - 2\,\text{MAa}(-1+\text{pXf})\,\text{waf}\,\alpha\text{m})}{2\,\text{meandipM}\,\text{meanhapF}\,\text{meanhapM}\,\xi}$$

$$-\frac{\text{wam}(\text{Maa}(-1+\text{pXf})\,\text{waf} + 2\,\text{MAa}\,\text{pXf}\,\text{wAf}(-1+\alpha\text{m}))}{2\,\text{meandipM}\,\text{meanhapF}\,\text{meanhapM}\,\xi}$$

and the rate at which recombination occurs

```
χmA0 = R MAa Coefficient[noreceigens0[[2]], MAa]
χma0 = R MAa Coefficient[noreceigens0[[1]], MAa]
```

$$-\frac{\text{MAa}\,(-1+\text{pXf})\,\text{R}\,\text{waf}\,\text{wAm}\,\alpha\text{m}}{\text{meandipM}\,\text{meanhapF}\,\text{meanhapM}\,\xi}$$

$$-\frac{\text{MAa}\,\text{pXf}\,\text{R}\,\text{wAf}\,\text{wam}\,(-1+\alpha\text{m})}{\text{meandipM}\,\text{meanhapF}\,\text{meanhapM}\,\xi}$$

The b coefficient can then be written as

```
-(λmA0 + λma0) + (χmA0 + χma0);

% - bcoef0 * (wbarDipMale freqMale wbarHapMale wbarHapFemale / (meandipM ξ meanhapM meanhapF)) /. SUBS /. subequil //
 Simplify
```

0

and the c coefficient can be written as

```
(λmA0 - χmA0) (λma0 - χma0) - (χmA0 χma0);

% - ccoef0 * (wbarDipMale freqMale wbarHapMale wbarHapFemale / (meandipM ξ meanhapM meanhapF))^2 /. SUBS /. subequil //
 Simplify
```

0

neo-W characteristic polynomial

As shown above, the relevant part of the characteristic polynomial for a feminizing mutant is

```
charpolyα1
```

$$\begin{aligned}&-\Big(\text{waf}\,\text{wAf}\,(\text{Faa}\,(1+\text{pXm}\,(-1+q)-\text{pYm}\,q)\,\text{wam}\\&\qquad(\text{FAA}\,(\text{pXm}\,(-1+q)-\text{pYm}\,q)\,\text{wAm}+2\,\text{FAa}\,(1+\text{pXm}\,(-1+q)-\text{pYm}\,q)\,(-1+R)\,\text{wam}\,\alpha\text{f})+\\&\qquad 2\,\text{FAa}\,(\text{pXm}\,(-1+q)-\text{pYm}\,q)\,\text{wAm}\,(-1+\alpha\text{f})\,(-\text{FAA}\,(\text{pXm}\,(-1+q)-\text{pYm}\,q)\,(-1+R)\,\text{wAm}+\\&\qquad\quad 2\,\text{FAa}\,(1+\text{pXm}\,(-1+q)-\text{pYm}\,q)\,(-1+2\,R)\,\text{wam}\,\alpha\text{f}))\Big)\Big/\\&\quad\Big(4\,(-1+q)^2\,(\text{Faa}\,(-1+\text{pXf})\,(-1+\text{pXm})\,\text{waf}\,\text{wam}+\text{FAA}\,\text{pXf}\,\text{pXm}\,\text{wAf}\,\text{wAm}+\\&\qquad\text{FAa}\,(\text{pXm}\,\text{waf}\,\text{wAm}-\text{pXf}\,((-1+\text{pXm})\,\text{wAf}\,\text{wam}+\text{pXm}\,\text{waf}\,\text{wAm})))^2\Big)+\\&\quad((\text{Faa}\,(1+\text{pXm}\,(-1+q)-\text{pYm}\,q)\,\text{waf}\,\text{wam}+\text{FAA}\,(\text{pXm}-\text{pXm}\,q+\text{pYm}\,q)\,\text{wAf}\,\text{wAm}-\\&\qquad 2\,\text{FAa}\,(-1+R)\,(\text{wAf}\,\text{wam}\,\alpha\text{f}+\text{pXm}\,(-1+q)\,(\text{waf}\,\text{wAm}\,(-1+\alpha\text{f})+\text{wAf}\,\text{wam}\,\alpha\text{f})-\\&\qquad\quad\text{pYm}\,q\,(\text{waf}\,\text{wAm}\,(-1+\alpha\text{f})+\text{wAf}\,\text{wam}\,\alpha\text{f})))\,\lambda)\,/\\&\quad(2\,(-1+q)\,(\text{Faa}\,(-1+\text{pXf})\,(-1+\text{pXm})\,\text{waf}\,\text{wam}+\text{FAA}\,\text{pXf}\,\text{pXm}\,\text{wAf}\,\text{wAm}+\\&\qquad\text{FAa}\,(\text{pXm}\,\text{waf}\,\text{wAm}-\text{pXf}\,((-1+\text{pXm})\,\text{wAf}\,\text{wam}+\text{pXm}\,\text{waf}\,\text{wAm}))))+\lambda^2\end{aligned}$$

```
acoef1 = Coefficient[charpolyα1, λ^2];
bcoef1 = Coefficient[charpolyα1, λ] / acoef1;
ccoef1 = (charpolyα1 - acoef1 λ^2 - acoef1 bcoef1 λ) / acoef1;
```

The b coefficient is

```
bcoef1 * (wbarDipFemale freqFemale wbarHapMale wbarHapFemale / (meandipF (1 - ζ) meanhapM meanhapF)) /. SUBS /. subequil //
 Simplify
```

$$\frac{1}{2\,\text{meandipF meanhapF meanhapM}\,(-1+\zeta)}(\text{Faa}\,(1+\text{pXm}\,(-1+q)-\text{pYm}\,q)\,\text{waf wam}+\text{FAA}\,(\text{pXm}-\text{pXm}\,q+\text{pYm}\,q)\,\text{wAf wAm}-2\,\text{FAa}\,(-1+R)\,(\text{wAf wam}\,\alpha f+\text{pXm}\,(-1+q)\,(\text{waf wAm}\,(-1+\alpha f)+\text{wAf wam}\,\alpha f)-\text{pYm}\,q\,(\text{waf wAm}\,(-1+\alpha f)+\text{wAf wam}\,\alpha f)))$$

The c coefficient is

```
ccoef1 * (wbarDipFemale freqFemale wbarHapMale wbarHapFemale / (meandipF (1 - ζ) meanhapM meanhapF))^2 /. SUBS /. subequil //
 Simplify
```

$$-(\text{waf wAf}\,(\text{Faa}\,(1+\text{pXm}\,(-1+q)-\text{pYm}\,q)\,\text{wam}\,(\text{FAA}\,(\text{pXm}\,(-1+q)-\text{pYm}\,q)\,\text{wAm}+2\,\text{FAa}\,(1+\text{pXm}\,(-1+q)-\text{pYm}\,q)\,(-1+R)\,\text{wam}\,\alpha f)+2\,\text{FAa}\,(\text{pXm}\,(-1+q)-\text{pYm}\,q)\,\text{wAm}\,(-1+\alpha f)\,(-\text{FAA}\,(\text{pXm}\,(-1+q)-\text{pYm}\,q)\,(-1+R)\,\text{wAm}+2\,\text{FAa}\,(1+\text{pXm}\,(-1+q)-\text{pYm}\,q)\,(-1+2\,R)\,\text{wam}\,\alpha f)))\Big/\left(4\,\text{meandipF}^2\,\text{meanhapF}^2\,\text{meanhapM}^2\,(-1+\zeta)^2\right)$$

The no recombination eigenvalues are (this takes a couple minutes)

```
noreceigens1 = (λ /. Solve[0 == charpolyα1 /. R → 0, λ]) *
    (wbarDipFemale freqFemale wbarHapMale wbarHapFemale / (meandipF (1 - ζ) meanhapM meanhapF)) /. SUBS /.
   subequil /. pXm → (pAveM - q pYm) / (1 - q) // Factor // Simplify
```

$$\left\{\frac{\text{FAA pAveM wAf wAm}+2\,\text{FAa wAf wam}\,\alpha f-2\,\text{FAa pAveM wAf wam}\,\alpha f}{2\,\text{meandipF meanhapF meanhapM}-2\,\text{meandipF meanhapF meanhapM}\,\zeta},\ \frac{\text{waf}\,(\text{Faa}\,(-1+\text{pAveM})\,\text{wam}+2\,\text{FAa pAveM wAm}\,(-1+\alpha f))}{2\,\text{meandipF meanhapF meanhapM}\,(-1+\zeta)}\right\}$$

Define the no recombination eigenvalues

```
λmA1 = noreceigens1[[1]]
λma1 = noreceigens1[[2]]
```

$$\frac{\text{FAA pAveM wAf wAm}+2\,\text{FAa wAf wam}\,\alpha f-2\,\text{FAa pAveM wAf wam}\,\alpha f}{2\,\text{meandipF meanhapF meanhapM}-2\,\text{meandipF meanhapF meanhapM}\,\zeta}$$

$$\frac{\text{waf}\,(\text{Faa}\,(-1+\text{pAveM})\,\text{wam}+2\,\text{FAa pAveM wAm}\,(-1+\alpha f))}{2\,\text{meandipF meanhapF meanhapM}\,(-1+\zeta)}$$

and the rate at which recombination occurs

```
χmA1 = R FAa Coefficient[noreceigens1[[1]], FAa]
χma1 = R FAa Coefficient[noreceigens1[[2]], FAa]
```

$$\frac{\text{FAa R } (2\text{ wAf wam }\alpha\text{f} - 2\text{ pAveM wAf wam }\alpha\text{f})}{2\text{ meandipF meanhapF meanhapM} - 2\text{ meandipF meanhapF meanhapM }\xi}$$

$$\frac{\text{FAa pAveM R waf wAm } (-1 + \alpha\text{f})}{\text{meandipF meanhapF meanhapM } (-1 + \xi)}$$

The b coefficient can then be written as

```
-(λmA1 + λma1) + (χmA1 + χma1);
```

```
% - bcoef1 * (wbarDipFemale freqFemale wbarHapMale wbarHapFemale / (meandipF (1 - ξ) meanhapM meanhapF)) /. SUBS /.
    subequil /. pXm → (pAveM - q pYm)/(1 - q) // Simplify
```

0

and the c coefficient can be written as

```
(λmA1 - χmA1) (λma1 - χma1) - (χmA1 χma1) // Simplify;
```

```
% - ccoef1 * (wbarDipFemale freqFemale wbarHapMale wbarHapFemale / (meandipF (1 - ξ) meanhapM meanhapF))^2 /. SUBS /.
    subequil /. pXm → (pAveM - q pYm)/(1 - q) // Simplify
```

0

Invasion conditions

```
Clear[λmA, λma]
```

To recap, with either a neo-Y or a neo-W, invasion is determined by the solution to the quadratic $f(\lambda) = \lambda^2 + b\,\lambda\, + c = 0$, where

b = - (λmA - χmA) - (λma - χma)

c = (λmA - χmA) (λma - χma) - χmA χma.

When R=0, both χmA = χma = 0, and the two solutions for λ are λmA and λma. We call these the “haplotype-only growth rates”, as they indicate how rapidly the haplotypes would spread if recombination never altered the background of the neo-SD.

In this section, we show for R>0 that:

* **Lemma 1:** invasion always becomes more difficult as R rises.
* **Lemma 2:** invasion is always prohibited if both λmA<1 and λma<1.
* **Lemma 3:** invasion is guaranteed for any R if both λmA>1 and λma>1. In particular, invasion of a neo-SD on a different chromosome (R = 1/2) will occur in this case.
* **Lemma 4:** invasion becomes more difficult as R rises if only one of λmA and λma is greater than one, requiring that equation (1) in the paper be satisfied.

Preliminaries: We are guaranteed to have both roots of f(λ) be greater than one if the slope at λ = 1 is negative, i.e., if $f'(1) = 2 + b < 0$, or equivalently when $-\frac{b}{2} = \frac{(\lambda mA - \chi mA) + (\lambda ma - \chi ma)}{2} > 1$. This is because the Perron-Frobenius theorem for a stability matrix without negative entries guarantees that the leading eigenvalue is positive and real and we know f(λ) is concave up. Thus, if the quadratic has a negative slope at λ=1 then it will cross zero and cross back at some λ>1. In words, the neo-SD is guaranteed to spread when the haplotype-only growth rate minus its loss due to recombination averaged for the mA and ma haplotypes is greater than 1. We will see that this condition is a bit stronger than we really need, because it does not consider the fact that recombination also creates mutant haplotypes.

When - $\frac{b}{2}$ < 1, then we will still have one λ>1 when f(1) = 1 + b + c < 0. Again, the leading eigenvalue must be real and postitive and f(λ) is concave up, so if it is negative at λ=1 it must cross zero at some λ>1. This condition can be rearranged to give equation (1)

$$(\lambda mA-1)\,(\lambda ma-1) < \chi ma\,(\lambda mA-1)+\chi mA\,(\lambda ma-1)$$

```
(λmA - 1) (λma - 1) - (χma (λmA - 1) + χmA (λma - 1));

% - (1 + b + c) /. b -> - ((λmA - χmA) + (λma - χma)) /.
   c -> (λmA - χmA) (λma - χma) - χmA χma // Factor
```

0

Furthermore, the leading eigenvalue, λ_L, is bounded between λmA and λma, given that the quadratic has opposite signs when evaluated at these two values:

```
λ^2 + b λ + c /. b -> - ((λmA - χmA) + (λma - χma)) /. c -> (λmA - χmA) (λma - χma) - χmA χma;
% /. λ → λmA // Factor
% /. λ → λma // Factor
```

$(-\lambda ma + \lambda mA)\ \chi mA$

$-(\lambda ma - \lambda mA)\ \chi mA$

when R = 0, the χ terms are zero and the larger of λma and λmA is the leading eigenvalue.

Proof of Lemma 1: We next prove that the leading eigenvalue always declines as R increases. First we note that both χma and χmA are proportional to R and we make this explicit in the quadratic f(λ):

```
λ[R]^2 + b λ[R] + c /. b -> - ((λmA - χmA) + (λma - χma)) /.
    c -> (λmA - χmA) (λma - χma) - χmA χma /. χmA → f_A * R /. χma → f_a * R
```

$-R^2\, f_a\, f_A + (\lambda ma - R\, f_a)\ (\lambda mA - R\, f_A) + (-\lambda ma - \lambda mA + R\, f_a + R\, f_A)\ \lambda[R] + \lambda[R]^2$

Solving for $\frac{d\lambda}{dR}$ and returning to the χ terms:

```
Solve[D[%, R] == 0, λ'[R] ] /. f_A -> χmA / R /. f_a -> χma / R /. λ[R] -> λ // Flatten //
  Simplify
```

$$\left\{\lambda'[R] \to \frac{\lambda mA\ \chi ma + \lambda ma\ \chi mA - \lambda\ (\chi ma + \chi mA)}{R\ (2\ \lambda - \lambda ma - \lambda mA + \chi ma + \chi mA)}\right\}$$

This can be written as:

$$-\ \frac{\chi ma+\chi mA}{R\ \mathrm{slope}}\left(\lambda_L - \frac{\lambda mA\ \chi ma+\lambda ma\ \chi mA}{\chi ma+\chi mA}\right)$$

where "slope" = $(2\lambda_L + b)$ is the slope of the quadratic, $\frac{df(\lambda)}{d\lambda}$, evaluated at the leading eigenvalue λ_L, which must be positive (because f(λ) is concave up and we're asking about the right-most root). Hence the first fraction is positive.

Furthermore, the second fraction must be positive, since the leading eigenvalue is strictly above $\frac{\lambda mA\ \chi ma + \lambda ma\ \chi mA}{\chi ma + \chi mA}$. This is because $f\left(\frac{\lambda mA\ \chi ma + \lambda ma\ \chi mA}{\chi ma + \chi mA}\right)$ is strictly negative and so the leading eigenvalue must occur at a higher value of λ:

```
λ² + b λ + c /. b -> - ((λmA - χmA) + (λma - χma)) /. c -> (λmA - χmA) (λma - χma) - χmA χma;
% /. λ → (λmA χma + λma χmA)/(χma + χmA) // Factor
```

$$-\frac{(\lambda ma - \lambda mA)^2\ \chi ma\ \chi mA}{(\chi ma + \chi mA)^2}$$

We conclude that the leading eigenvalue must fall as R increases.

Corollary of Lemma 1: The non-leading eigenvalue will also decline as R increase, because "slope" is then negative and the term, $\left(\lambda_N - \frac{\lambda mA\ \chi ma + \lambda ma\ \chi mA}{\chi ma + \chi mA}\right)$, will also be negative (because the non-leading eigenvalue must fall below $\frac{\lambda mA\ \chi ma + \lambda ma\ \chi mA}{\chi ma + \chi mA}$ is the quadratic is negative at that point).

Proof of Lemma 2: When both the haplotype-only growth rates (λmA and λma) are less than 1, then $-\frac{b}{2} = \frac{(\lambda mA - \chi mA) + (\lambda ma - \chi ma)}{2}$ must be less than one (recall that the χ terms are positive) and 1 + b + c cannot be negative because (λmA - 1) (λma - 1) < χma (λmA - 1) + χmA (λma - 1) is false (left hand side is positive while right hand side is negative). Thus, the neo-SD never invades when neither haplotype can grow in the absence of recombination (R=0).

Check: Can invasion occur?

```
Simplify[Reduce[
   {(λmA - χmA + λma - χma)/2 > 1(* Slope condition met: f'(1)< 0 *)
     || χma (λmA - 1) + χmA (λma - 1) > (λmA - 1) (λma - 1)
     (* or intercept condition met: f(1)>0*) }],
  {λmA < 1, λma < 1, 0 < χmA, 0 < χma}]
```

```
False
```

Proof of Lemma 3: When both the haplotype-only growth rates (λmA and λma) are greater than 1, we consider two sub-cases:

Case 1: Both haplotype-only growth rates are so large that the average of (λmA - χmA) and (λma - χma) is also greater than one. Then we are guaranteed that both roots of f(λ) are greater than one regardless of R since $-\frac{b}{2} = \frac{(\lambda mA - \chi mA) + (\lambda ma - \chi ma)}{2} > 1$.

Case 2: If, however, $-\frac{b}{2} < 1$, then invasion will still occur if both λmA and λma are greater than one. This is because the intercept can be written as:

$-\left[(\lambda mA-1)\ (2+b)+(\lambda mA-1)^2+(\lambda ma-\lambda mA)\ \chi mA\right]$

and equivalently as:

$-\left[(\lambda ma-1)\ (2+b)+(\lambda ma-1)^2+(\lambda mA-\lambda ma)\ \chi ma\right]$

Thus, if λma ≥ λmA, we choose the first way to write the intercept, which is clearly negative (all terms in square brackets are positive given that both λmA and λma are greater than one and $-\frac{b}{2}<1$). If if λmA ≥ λma, we choose the second way to write the intercept, which is also clearly negative. Either way, invasion is guaranteed regardless of R.

```
- ((λmA - 1) (2 + b) + (λmA - 1)^2 + (λma - λmA) χmA);
% - (1 + b + c) /. b -> - ((λmA - χmA) + (λma - χma)) /.
  c -> (λmA - χmA) (λma - χma) - χmA χma // Factor
```

0

```
- ((λma - 1) (2 + b) + (λma - 1)^2 + (λmA - λma) χma);
% - (1 + b + c) /. b -> - ((λmA - χmA) + (λma - χma)) /.
  c -> (λmA - χmA) (λma - χma) - χmA χma // Factor
```

0

Check: Can invasion occur?

```
Simplify[Reduce[
  {(λmA - χmA + λma - χma)/2 > 1(* Slope condition met: f'(1)< 0 *)
    || χma (λmA - 1) + χmA (λma - 1) > (λmA - 1) (λma - 1)
    (* or intercept condition met: f(1)>0*) }],
 {λmA > 1, λma > 1, 0 < χmA, 0 < χma}]
```

True

Proof of Lemma 4: When only one of the haplotype-only growth rates (λmA and λma) is greater than 1, we first note that stability is determined solely by the intercept of f(λ) at λ = 1, because the slope at λ = 1 is immaterial. This is because Lemma 1 and its corollary imply that both roots of f(λ) decline as R rises. Thus, if only one root is greater than 1 at R = 0 and if both roots decline with R, then we can never have both roots greater than one. With only one root possibly greater than 1, this root will be greater than one if and only if f(1) is negative.

Thus, when only one of λmA and λma is greater than one, invasion will occur according to the intercept condition:

$$(\lambda mA-1)\ (\lambda ma-1) < \chi ma\ (\lambda mA-1)+\chi mA\ (\lambda ma-1)$$

[dividing both sides by (λmA-1) (λma-1) and switching the inequality because this is negative]:

$$1 > \frac{\chi\text{ma}}{\lambda\text{ma}-1} + \frac{\chi\text{mA}}{\lambda\text{mA}-1}$$

which is equation (1). One of the terms on the right is positive and one negative. Because the χ are both positive and proportional to R, the condition is always satisfied when R = 0 (right-hand side is zero).

In words, we need the amount of recombination off the growing background, weighted by the growth rate of the growing haplotype, to be small enough relative to the amount of recombination off the shrinking haplotype, weighted by its rate of decline.

Check: Can invasion occur if equation (1) is satisfied?

```
Simplify[Reduce[
  {(λmA - χmA + λma - χma)/2 > 1(* Slope condition met: f'(1)< 0 *)
    || χma (λmA - 1) + χmA (λma - 1) > (λmA - 1) (λma - 1)
    (* or intercept condition met: f(1)>0*) }],
  {λmA > 1, λma < 1, 0 < χmA, 0 < χma, 1 > χma/(λma - 1) + χmA/(λmA - 1)}]
```

True

Tight linkage

neo-Y equilA (r ~ 0)

From the above conditions, invasion requires that at least one of the no recombination eigenvalues is greater than one

```
{λmA0, λma0} /. reverse /. equilA0 // Simplify
```

$$\{(\text{wAm}\,(\text{Faa Maa MAA waf wam} + 4\,\text{FAA MAa}^2\,\text{wAf wAm}\,\alpha\text{m}^2 + \text{FAa}\,(\text{Maa wam} + 2\,\text{MAa wAm}\,\alpha\text{m})\,(-\text{MAA wAf}\,\alpha\text{f} + 2\,\text{MAa waf}\,(-1+\alpha\text{f})\,\alpha\text{m})))\,/\,(\text{wam}\,(\text{FAa}\,(\text{Maa waf}\,(-1+\alpha\text{f}) + 2\,\text{MAa wAf}\,\alpha\text{f}\,(-1+\alpha\text{m}))\,(\text{Maa wam} + 2\,\text{MAa wAm}\,\alpha\text{m}) + 2\,\text{Maa MAa}\,(-\text{Faa waf wam}\,(-1+\alpha\text{m}) + \text{FAA wAf wAm}\,\alpha\text{m}))),\ 1\}$$

The first of these can be written as (1-factor) where factor is positive under the stability condition, demonstrating that a neo-Y cannot invade a system that already has tight linkage:

$$\left(1 - \frac{\left(\frac{\text{Maa}\,(1-\text{pXf})\,\text{waf}\,(\text{pXf wAf wam}\,(\text{pXm}\,(1-\alpha\text{m})+\alpha\text{m}\,(1-\text{pXm}))-\text{pXm}\,(1-\text{pXf})\,\text{waf wAm}\,\alpha\text{m})}{(1-\text{pXm})\,\text{pXf}^2\,\text{wAf}^2\,\text{wAm}\,\alpha\text{m}} - \text{MAA}\right)\text{wAm wAf}\,\alpha\text{m pXf}\,(1-\text{pXm})}{\text{Maa}\,(1-\text{pXf})\,\text{waf wam}\,(\text{pXm}\,(1-\alpha\text{m})+\alpha\text{m}\,(1-\text{pXm}))}\right);$$

```
(%%[[1]]) - % /. subA1 /. subA2 // Factor
```

0

Confirming that invasion is not possible:

```
Reduce[
 {((λmA0 > 1 || λma0 > 1) /. reverse /. equilA0 // Simplify) && (stabcondA /. equilA0)}]
False
```

neo-Y equilB (r ~ 0)

From the above conditions, invasion requires that at least one of the no recombination eigenvalues is greater than one

```
{λmA0, λma0} /. reverse /. equilB0 // Simplify
```

$$\left\{\frac{\text{MAA wAm}}{2\ \text{MAa wam} - 2\ \text{MAa wam}\ \alpha m},\ 1\right\}$$

This is equilibrium is internally stable only if $\frac{\text{MAA wAm}}{2\ \text{MAa wam}\ (1-\alpha m)} < 1$, so a neo-Y can be neutral at best and will not invade.

Confirming:

This is not possible given the stability conditions for equilB

```
Reduce[{((λmA0 > 1 || λma0 > 1) /. reverse /. equilB0 // Simplify) && stabcondB}]
False
```

neo-W equilA (r ~ 0)

The four terms that define the characteristic polynomial are

```
{λmA1, λma1, χmA1, χma1} /. reverse /. pAveM → q pYm + (1 - q) pXm /. equilA0 // Simplify
```

$$\Big\{\Big(\text{Faa FAA Maa MAa waf wam wAm}\ \alpha m + 2\ \text{FAa}^2\ \text{wam}\ \alpha f\ (\text{Maa waf}\ (-1+\alpha f) + \text{MAa wAf}\ \alpha f\ (-1+\alpha m))\ (\text{Maa wam} + 2\ \text{MAa wAm}\ \alpha m) + \text{FAa MAa}\ \alpha f\ \big(-2\ \text{Faa Maa waf wam}^2\ (-1+\alpha m) + \text{FAA wAf wAm}\ \alpha m\ (3\ \text{Maa wam} - 2\ \text{MAa wAm}\ \alpha m)\big)\Big) \Big/ \Big(\text{waf}\ \big(2\ \text{Faa FAA Maa MAa wam wAm}\ \alpha m + \text{FAa}^2\ (-1+\alpha f)\ \alpha f\ (\text{Maa wam} + 2\ \text{MAa wAm}\ \alpha m)^2\big)\Big),$$

$$\Big(-\text{Faa}^2\ \text{Maa MAa waf wam}^2\ (-1+\alpha m) + 2\ \text{FAa}^2\ \text{MAa wAf wAm}\ (-1+\alpha f)\ \alpha f\ \alpha m\ (\text{Maa wam} + 2\ \text{MAa wAm}\ \alpha m) + \text{Faa wam}\ \big(2\ \text{FAA Maa MAa wAf wAm}\ \alpha m + \text{FAa}\ \big(\text{Maa}^2\ \text{waf wam}\ (-1+\alpha f) + \text{Maa MAa wAf wam}\ \alpha f\ (-1+\alpha m) + 2\ \text{MAa}^2\ \text{wAf wAm}\ \alpha f\ (-1+\alpha m)\ \alpha m\big)\big)\Big) \Big/ \Big(\text{wAf}\ \big(2\ \text{Faa FAA Maa MAa wam wAm}\ \alpha m + \text{FAa}^2\ (-1+\alpha f)\ \alpha f\ (\text{Maa wam} + 2\ \text{MAa wAm}\ \alpha m)^2\big)\Big),$$

$$\big(2\ \text{FAa R wam}\ \alpha f\ (\text{FAa}\ (\text{Maa waf}\ (-1+\alpha f) + \text{MAa wAf}\ \alpha f\ (-1+\alpha m))\ (\text{Maa wam} + 2\ \text{MAa wAm}\ \alpha m) + \text{Maa MAa}\ (-\text{Faa waf wam}\ (-1+\alpha m) + 2\ \text{FAA wAf wAm}\ \alpha m))\big) \Big/ \Big(\text{waf}\ \big(2\ \text{Faa FAA Maa MAa wam wAm}\ \alpha m + \text{FAa}^2\ (-1+\alpha f)\ \alpha f\ (\text{Maa wam} + 2\ \text{MAa wAm}\ \alpha m)^2\big)\Big),$$

$$\frac{2\ \text{FAa MAa R wAm}\ (-1+\alpha f)\ \alpha m\ (-\text{Faa Maa waf wam} + \text{FAa wAf}\ \alpha f\ (\text{Maa wam} + 2\ \text{MAa wAm}\ \alpha m))}{\text{wAf}\ \big(2\ \text{Faa FAA Maa MAa wam wAm}\ \alpha m + \text{FAa}^2\ (-1+\alpha f)\ \alpha f\ (\text{Maa wam} + 2\ \text{MAa wAm}\ \alpha m)^2\big)}\Big\}$$

As shown in the equilibria section above, we can write equilA as

```
equilA0simp = {q → (1 - αDm MAa ϕ / (MAa ϕ + Maa ψ)) / 2,
   pXf → waf ϕ / (waf ϕ + wAf ψ), pXm → (1 + αDm) MAa ϕ / ((1 + αDm) MAa ϕ + Maa ψ), pYm → 0};
```

Define 3 new terms a,b,c

```
trysub2 = {(waf ϕ + wAf ψ) → a, (MAa (-1 + αDm) ϕ - 2 Maa ψ) → -c,
   FAA MAa wAm (1 + αDm) ϕ^2 + ψ (FAa (Maa wam + MAa wAm (1 + αDm)) ϕ + Faa Maa wam ψ) → b};
trysub2back = {a → (waf ϕ + wAf ψ), c → - (MAa (-1 + αDm) ϕ - 2 Maa ψ),
   b → FAA MAa wAm (1 + αDm) ϕ^2 + ψ (FAa (Maa wam + MAa wAm (1 + αDm)) ϕ + Faa Maa wam ψ)};
```

The four terms in the characteristic polynomial can then be written

```
equilA0terms =
 Simplify[{λmA1, λma1, χmA1, χma1} /. αm → (αDm + 1) / 2 /. αf → (αDf + 1) / 2 /. reverse /.
    pAveM → q pYm + (1 - q) pXm /. equilA0simp] /. trysub2
```

$$\left\{\frac{a\,(c\,\text{FAa wam}\,(1+\alpha\text{Df})+\text{FAA MAa wAm}\,(1+\alpha\text{Dm})\,\phi)}{2\,b\,\text{waf}},\right.$$
$$-\frac{a\,(-c\,\text{Faa wam}+\text{FAa MAa wAm}\,(-1+\alpha\text{Df})\,(1+\alpha\text{Dm})\,\phi)}{2\,b\,\text{wAf}},$$
$$\left.\frac{a\,c\,\text{FAa R wam}\,(1+\alpha\text{Df})}{2\,b\,\text{waf}},\ -\frac{a\,\text{FAa MAa R wAm}\,(-1+\alpha\text{Df})\,(1+\alpha\text{Dm})\,\phi}{2\,b\,\text{wAf}}\right\}$$

where

```
(freqMale == wam (c - Maa ψ) / (wam c + wAm MAa (1 + αDm) ϕ)) /. equilA0simp /. trysub2back // Simplify
(freqFemale == (wam Maa ψ + wAm MAa (1 + αDm) ϕ) / (wam c + wAm MAa (1 + αDm) ϕ)) /. equilA0simp /. trysub2back // Simplify
(1 / (2 freqFemale) == (wam c + wAm MAa (1 + αDm) ϕ) / (2 (wam Maa ψ + wAm MAa (1 + αDm) ϕ))) /. equilA0simp /. trysub2back //
 Simplify
```

```
True
```

```
True
```

```
True
```

In the absence of haploid selection the four terms are

```
equilA0termsNoHap =
 equilA0terms /. trysub2back(*/.trysub*) /. αDm → 2 αm - 1 /. αDf → 2 αf - 1 /. waf → 1 /.
       wAf → 1 /. wam → 1 /. wAm → 1 /. αm → 1 / 2 /. αf → 1 / 2 // Simplify
```

$$\left\{\frac{(\phi+\psi)\,(\text{FAa MAa}\,\phi+\text{FAA MAa}\,\phi+2\,\text{FAa Maa}\,\psi)}{2\,\left(\text{FAA MAa}\,\phi^2+\psi\,(\text{FAa}\,(\text{Maa}+\text{MAa})\,\phi+\text{Faa Maa}\,\psi)\right)},\right.$$
$$\frac{(\phi+\psi)\,(\text{Faa MAa}\,\phi+\text{FAa MAa}\,\phi+2\,\text{Faa Maa}\,\psi)}{2\,\left(\text{FAA MAa}\,\phi^2+\psi\,(\text{FAa}\,(\text{Maa}+\text{MAa})\,\phi+\text{Faa Maa}\,\psi)\right)},$$
$$\frac{\text{FAa R}\,(\phi+\psi)\,(\text{MAa}\,\phi+2\,\text{Maa}\,\psi)}{2\,\left(\text{FAA MAa}\,\phi^2+\psi\,(\text{FAa}\,(\text{Maa}+\text{MAa})\,\phi+\text{Faa Maa}\,\psi)\right)},$$
$$\left.\frac{\text{FAa MAa R}\,\phi\,(\phi+\psi)}{2\,\left(\text{FAA MAa}\,\phi^2+\psi\,(\text{FAa}\,(\text{Maa}+\text{MAa})\,\phi+\text{Faa Maa}\,\psi)\right)}\right\}$$

Interpretation:

A neo-W carrying the A allele would have mean representation in the pool of female gametes (accounting for meiotic drive) of:

$$\left(\text{FAA}\,\frac{\text{wAf}}{\text{wbarHapFemale}}\,\text{pXm}\,(1-\text{q})\,\frac{\text{wAm}}{\text{wbarHapMale}}+\frac{\alpha\text{f}}{1/2}\,\text{FAa}\,\frac{\text{wAf}}{\text{wbarHapFemale}}\,(1-\text{pXm})\,(1-\text{q})\,\frac{\text{wam}}{\text{wbarHapMale}}+\text{FAA}\,\frac{\text{wAf}}{\text{wbarHapFemale}}\,\text{pYm q}\,\frac{\text{wAm}}{\text{wbarHapMale}}+\frac{\alpha\text{f}}{1/2}\,\text{FAa}\,\frac{\text{wAf}}{\text{wbarHapFemale}}\,(1-\text{pYm})\,\text{q}\,\frac{\text{wam}}{\text{wbarHapMale}}\right)\text{ /. SUBS /. subequil // Factor}$$

$$(\text{wAf}\,(-\text{FAA pXm wAm}+\text{FAA pXm q wAm}-\text{FAA pYm q wAm}-2\,\text{FAa wam}\,\alpha\text{f}+2\,\text{FAa pXm wam}\,\alpha\text{f}-2\,\text{FAa pXm q wam}\,\alpha\text{f}+2\,\text{FAa pYm q wam}\,\alpha\text{f}))\,/\,((\text{waf}-\text{pXf waf}+\text{pXf wAf})\,(-\text{wam}+\text{pXm wam}-\text{pXm q wam}+\text{pYm q wam}-\text{pXm wAm}+\text{pXm q wAm}-\text{pYm q wAm}))$$

[Note that this is summed over all gametes donated by males because the W is assumed dominant and always produces females.]

This divided by the mean fitness of the wildtype M bearing females (meanF) divided by 2*freqfemale gives the leading eigenvalue in this case:

$$\frac{(\%\,/\,\text{wbarDipFemale})}{2\,\text{freqfemale}}\text{ /. SUBS /. subequil // Factor;}$$

```
% - λmA1 /. freqfemale → 1 - ξ /. SUBS /. reverse /.
   pAveM → q pYm + (1 - q) pXm /. equilA0 // Factor
```

0

The 2 freqfemale in the denominator describes a sex ratio advantage of being in the rarer sex.

A neo-W carrying the a allele would have mean representation in the pool of female gametes (accounting for meiotic drive) of:

$$\left(\frac{1-\alpha f}{1/2}\,\text{FAa}\,\frac{\text{waf}}{\text{wbarHapFemale}}\left(\text{pXm}\,(1-q)\,\frac{\text{wAm}}{\text{wbarHapMale}}\right)+\text{Faa}\,\frac{\text{waf}}{\text{wbarHapFemale}}\left((1-\text{pXm})\,(1-q)\,\frac{\text{wam}}{\text{wbarHapMale}}\right)+\frac{1-\alpha f}{1/2}\,\text{FAa}\,\frac{\text{waf}}{\text{wbarHapFemale}}\left(\text{pYm}\,q\,\frac{\text{wAm}}{\text{wbarHapMale}}\right)+\text{Faa}\,\frac{\text{waf}}{\text{wbarHapFemale}}\left((1-\text{pYm})\,q\,\frac{\text{wam}}{\text{wbarHapMale}}\right)\right)\text{ /. SUBS /. subequil // Factor}$$

```
- (waf (Faa wam - Faa pXm wam + Faa pXm q wam - Faa pYm q wam + 2 FAa pXm wAm - 2 FAa pXm q wAm +
      2 FAa pYm q wAm - 2 FAa pXm wAm αf + 2 FAa pXm q wAm αf - 2 FAa pYm q wAm αf)) /
  ((-waf + pXf waf - pXf wAf) (wam - pXm wam + pXm q wam - pYm q wam +
      pXm wAm - pXm q wAm + pYm q wAm))
```

This divided by the mean fitness of the wildtype M bearing females (meanF) divided by 2*freqfemale gives the leading eigenvalue in this case:

$$\frac{(\%\,/\,\text{wbarDipFemale})}{2\ \text{freqfemale}}\ \text{// Factor;}$$

```
% - λmal /. freqfemale → 1 - ξ /. SUBS /. reverse /. pAveM → q pYm + (1 - q) pXm /. equilA0 //
  Factor
```

0

It is difficult for the neo-W to spread with only sexually antagonistic selection because the neo-W often makes daughters with the Y-a haplotype, increasing the flow of “a” alleles (that are fixed on the Y and in that sense male-beneficial) into the daughters. But if $\frac{\text{fitness[W-a]/meandipF}}{2\ \text{freqfemale}} > 1$ or if $\frac{\text{fitness[W-A]/meandipF}}{2\ \text{freqfemale}} > 1$ then invasion is possible.

R near 0:

Consequently, when R = 0 (the χ terms absent), the neo-W-A haplotype spreads when the following is greater than 0

```
Collect[Numerator[equilA0termsNoHap[[1]]] - Denominator[equilA0termsNoHap[[1]]],
  {Maa, MAa}, Simplify]
```

$$(\text{FAa} - \text{FAA})\ \text{MAa}\ \phi\ (\phi - \psi) + 2\ (-\text{Faa} + \text{FAa})\ \text{Maa}\ \psi^2$$

where ϕ>0 and ψ>0 for equilA to be valid and stable and ϕ-ψ in the absence of haploid selection is

```
ϕ - ψ /. trysub /. αDm → 2 αm - 1 /. αDf → 2 αf - 1 /. waf → 1 /. wAf → 1 /. wam → 1 /. wAm → 1 /.
   αm → 1 / 2 /. αf → 1 / 2
```

$$-\text{Faa Maa} + \text{FAA MAa}$$

Thus the neo-W-A haplotype can spread from equilA when sufficiently tightly linked under purely sexually antagonistic selection, e.g.,

```
Reduce[
 {stabcondA, equilA0termsNoHap[[1]] > 1, Faa < FAa < FAA, MAA < MAa < Maa} /. trysub /.
         equilA0 /. αDm → 2 αm - 1 /. αDf → 2 αf - 1 /. waf → 1 /.
      wAf → 1 /. wam → 1 /. wAm → 1 /. αm → 1 / 2 /. αf → 1 / 2]
```

$$0 \le Rf \le \frac{1}{2} \,\&\&\, 0 \le Rm \le \frac{1}{2} \,\&\&\, MAa > 0 \,\&\&\, Maa > MAa \,\&\&\, FAA > 0 \,\&\&\, \left(\left(\frac{2\,FAA\,MAa}{Maa + MAa} < FAa < \frac{3\,FAA\,Maa\,MAa - FAA\,MAa^2}{Maa^2 + Maa\,MAa} \,\&\&\, \frac{FAa^2\,Maa^2 + FAa^2\,Maa\,MAa - 3\,FAa\,FAA\,Maa\,MAa + FAa\,FAA\,MAa^2}{2\,FAa\,Maa\,MAa - 2\,FAA\,Maa\,MAa} < Faa < \frac{FAa\,Maa + FAa\,MAa}{2\,Maa} \,\&\&\, 0 < MAA < MAa\right) \,||\, \left(\frac{3\,FAA\,Maa\,MAa - FAA\,MAa^2}{Maa^2 + Maa\,MAa} \le FAa < FAA \,\&\&\, 0 < Faa < \frac{FAa\,Maa + FAa\,MAa}{2\,Maa} \,\&\&\, 0 < MAA < MAa\right)\right)$$

for example

```
Reduce[{stabcondA, validcondA, equilA0termsNoHap[[1]] > 1, Faa < FAa < FAA,
                    MAA < MAa < Maa} /. trysub /. equilA0 /. αDm → 2 αm - 1 /.
               αDf → 2 αf - 1 /. waf → 1 /. wAf → 1 /. wam → 1 /. wAm → 1 /.
          αm → 1 / 2 /. αf → 1 / 2 /. Faa → 9 / 10 /. FAa → 1 /.
     FAA → 11 / 10 /. MAA → 9 / 10 /. MAa → 1 /. Rf → 0 /. Rm → 0] // N
```

$1.21362 < Maa < 1.25$

Similarly, the neo-W-a haplotype thus spreads when the following is greater than 0

```
Collect[Numerator[equilA0termsNoHap[[2]]] -
  Denominator[equilA0termsNoHap[[2]]], {ϕ}, Simplify]
```

$(Faa + FAa - 2\,FAA)\,MAa\,\phi^2 + (Faa - FAa)\,(2\,Maa + MAa)\,\phi\,\psi$

but this is not possible with purely sexually antagonistic selection

```
Reduce[
 {stabcondA, equilA0termsNoHap[[2]] > 1, Faa < FAa < FAA, MAA < MAa < Maa} /. trysub /.
         equilA0 /. αDm → 2 αm - 1 /. αDf → 2 αf - 1 /. waf → 1 /.
      wAf → 1 /. wam → 1 /. wAm → 1 /. αm → 1 / 2 /. αf → 1 / 2]
```

False

Other forms of selection allow the neo-W to invade an internally stable equilibrium, even without haploid selection:

```
Simplify[
 Reduce[{stabcondA, ((λmA1 > 1) || (λma1 > 1))} /. reverse /. pAveM → q pYm + (1 - q) pXm /.
      equilA0
     /. nohap /. Rm → 0 /. Rf → 0]]
```

$$\text{FAa} > 0 \ \&\&\ \text{MAa} > 0 \ \&\&\ 0 < \text{MAA} \ \&\& \\ \text{MAA} < \frac{2\,(\text{Faa Maa} + \text{FAA}\,(\text{Maa} - \text{MAa}))\,\text{MAa} - \text{FAa Maa}\,(\text{Maa} + \text{MAa})}{2\,\text{Faa Maa} - \text{FAa}\,(\text{Maa} + \text{MAa})} \ \&\&\ \Bigg(\Bigg(0 < \text{Faa} \ \&\&\ 0 < \text{FAA} \ \&\& \\ \Bigg(\Bigg(\text{Maa} == \text{MAa} \ \&\&\ \text{FAa} + \frac{\text{FAa Maa}}{\text{MAa}} > 2\,\text{FAA} \ \&\&\ 2\,\text{Faa} < \text{FAa} + \frac{\text{FAa Maa}}{\text{MAa}}\Bigg) \ ||\ \Bigg(\text{Maa} > \text{MAa} \ \&\& \\ \text{Faa} < \frac{\text{FAa}\,(\text{Maa} + \text{MAa})}{2\,\text{Maa}} \ \&\&\ \text{FAA} + \frac{\text{FAa Maa}\,(-2\,\text{Faa MAa} + \text{FAa}\,(\text{Maa} + \text{MAa}))}{\text{MAa}\,(2\,\text{Faa Maa} + \text{FAa}\,(-3\,\text{Maa} + \text{MAa}))} < 0\Bigg)\Bigg)\Bigg) \ || \\ \Bigg(\text{Maa} < \text{MAa} \ \&\&\ \Bigg(\Bigg(\text{Faa} > 0 \ \&\&\ \text{FAA} > 0 \ \&\&\ \text{FAa} + \frac{\text{FAa Maa}}{\text{MAa}} > 2\,\text{FAA} \ \&\&\ 0 < \text{Maa} \ \&\& \\ 2\,\text{Faa} \le \text{FAa} + \frac{\text{FAa Maa}}{\text{MAa}}\Bigg) \ ||\ \Big(\text{Maa} > 0 \ \&\&\ 2\,\text{Faa Maa} < \text{FAa}\,(\text{Maa} + \text{MAa}) \ \&\& \\ 2\,\text{FAA MAa} < \text{FAa}\,(\text{Maa} + \text{MAa}) \ \&\&\ 2\,(\text{Faa} + \text{FAA})\,\text{Maa MAa} < \\ 2\,\text{FAA MAa}^2 + \text{FAa Maa}\,(\text{Maa} + \text{MAa}) \ \&\&\ \text{FAa}\,(\text{Maa} + \text{MAa}) < 2\,\text{Faa MAa}\Big)\Bigg)\Bigg)\Bigg)$$

such as heterozygote advantage in males with directional selection in females:

```
Simplify[Reduce[{stabcondA, λmA1 > 1, (((0 < FAA < FAa < Faa) && (Maa < MAa > MAA > 0)))}
        /. reverse /. pAveM → q pYm + (1 - q) pXm /. equilA0 /. nohap /. Rm → 0 /. Rf → 0]]
Simplify[Reduce[{stabcondA, λma1 > 1, (((0 < FAA < FAa < Faa) && (Maa < MAa > MAA > 0)))}
        /. reverse /. pAveM → q pYm + (1 - q) pXm /. equilA0 /. nohap /. Rm → 0 /. Rf → 0]]
```

$$\text{MAa} > 0 \ \&\&\ 0 < \text{Maa} < \text{MAa} \ \&\&\ \text{FAA} > 0 \ \&\&\ \frac{2\,\text{FAA MAa}}{\text{Maa} + \text{MAa}} < \text{FAa} < \frac{\text{FAA MAa}}{\text{Maa}} \ \&\& \\ \text{FAa} < \text{Faa} < \frac{\text{FAa}\,(\text{FAA MAa}\,(-3\,\text{Maa} + \text{MAa}) + \text{FAa Maa}\,(\text{Maa} + \text{MAa}))}{2\,(\text{FAa} - \text{FAA})\,\text{Maa MAa}} \ \&\& \\ 0 < \text{MAA} < \frac{2\,(\text{Faa Maa} + \text{FAA}\,(\text{Maa} - \text{MAa}))\,\text{MAa} - \text{FAa Maa}\,(\text{Maa} + \text{MAa})}{2\,\text{Faa Maa} - \text{FAa}\,(\text{Maa} + \text{MAa})}$$

$$\text{MAa} > 0 \ \&\&\ \text{Maa} > 0 \ \&\&\ \text{FAa} > 0 \ \&\&\ \text{Faa} > 0 \ \&\&\ \text{MAA} > 0 \ \&\& \\ \text{Maa} < \text{MAa} \ \&\&\ \text{FAa} < \text{Faa} \ \&\&\ 2\,\text{Faa Maa} < \text{FAa}\,(\text{Maa} + \text{MAa}) \ \&\& \\ 2\,(\text{Faa} + \text{FAA})\,\text{Maa MAa} < 2\,\text{FAA MAa}^2 + \text{FAa Maa}\,(\text{Maa} + \text{MAa}) \ \&\&\ 2\,\text{FAA MAa} < \text{FAa}\,(\text{Maa} + \text{MAa}) \ \&\& \\ 2\,(\text{FAA}\,(\text{Maa} - \text{MAa})\,\text{MAa} + \text{Faa Maa}\,(\text{MAa} - \text{MAA})) < \text{FAa}\,(\text{Maa} + \text{MAa})\,(\text{Maa} - \text{MAA})$$

e.g., in the blue region of figure 2d in Otto 2014 JEB (p 1436)

```
Reduce[{%} /. Faa → 1.05 /. FAa → 1 /. FAA → 0.6 /. MAa → 1 /. MAA → 0.5 /. Maa → 0.5] // N
```

```
True
```

Underdominance in females with directional selection in males does not work (it does for equilB, see next section):

```
Simplify[Reduce[{stabcondA, λmA1 > 1,
     (((FAA > FAa < Faa > 0) && ((0 < Maa < MAa < MAA) || (Maa > MAa > MAA > 0))))}
    /. reverse /. pAveM → q pYm + (1 - q) pXm /. equilA0 /. nohap /. Rm → 0 /. Rf → 0]]
Simplify[Reduce[{stabcondA, λma1 > 1,
     (((FAA > FAa < Faa > 0) && ((0 < Maa < MAa < MAA) || (Maa > MAa > MAA > 0))))}
    /. reverse /. pAveM → q pYm + (1 - q) pXm /. equilA0 /. nohap /. Rm → 0 /. Rf → 0]]
```

False

False

Heterozygote advantage in females with directional selection in males also works (only λmA1 can be greater than 1):

```
Simplify[Reduce[{stabcondA, λmA1 > 1,
     (((FAA < FAa > Faa > 0) && ((0 < Maa < MAa < MAA) || (Maa > MAa > MAA > 0))))}
    /. reverse /. pAveM → q pYm + (1 - q) pXm /. equilA0 /. nohap /. Rm → 0 /. Rf → 0]]
Simplify[Reduce[{stabcondA, λma1 > 1,
     (((FAA < FAa > Faa > 0) && ((0 < Maa < MAa < MAA) || (Maa > MAa > MAA > 0))))}
    /. reverse /. pAveM → q pYm + (1 - q) pXm /. equilA0 /. nohap /. Rm → 0 /. Rf → 0]]
```

$$\text{MAa} > 0 \;\&\&\; \text{Maa} > \text{MAa} \;\&\&\; \text{FAA} > 0 \;\&\&\; \text{FAa} > \text{FAA} \;\&\&\; \text{Faa} > 0 \;\&\&\; 2\,\text{Faa} < \text{FAa} + \frac{\text{FAa MAa}}{\text{Maa}} \;\&\&\; \text{MAA} > 0 \;\&\&\; \text{MAa} > \text{MAA}$$

False

Haploid selection increases the types of selection in the diploid phase that are consistent with neo-W invasion, including sexual antagonism in diploids:

```
(*Including meiotic drive in males*)
Simplify[
 Reduce[{stabcondA, ((λmA1 > 1) || (λma1 > 1)), (((FAA > FAa > Faa > 0) && (Maa > MAa >
          MAA > 0)) || ((0 < FAA < FAa < Faa) && (0 < Maa < MAa < MAA)))} /.
       reverse /. pAveM → q pYm + (1 - q) pXm /. equilA0 /. wam → 1 /. wAm → 1 /.
    waf → 1 /. wAf → 1 /. αf → 1 / 2 /. αm → 4 / 10 /. Rm → 0 /. Rf → 0]]
```

$$\text{FAa} > 0 \;\&\&\; \text{MAa} > 0 \;\&\&\; \Bigg(\Bigg(0 < \text{Faa} \;\&\&\; 0 < \text{MAA} \;\&\&\; \text{Faa} < \frac{1}{10}\,\text{FAa}\left(5 + \frac{4\,\text{MAa}}{\text{Maa}}\right) \;\&\&\; \text{FAa} < \text{FAA} \;\&\&\; \text{MAA} < \text{MAa} \;\&\&\; \Bigg(\left(\text{FAa}\left(4 + \frac{5\,\text{Maa}}{\text{MAa}}\right) > 8\,\text{FAA} \;\&\&\; \text{MAa} < \text{Maa} \;\&\&\; 5\,\text{Maa} \le 8\,\text{MAa}\right) \;||\; \Bigg(5\,\text{Maa} > 8\,\text{MAa} \;\&\&\; \text{FAA} + \frac{\text{FAa}\left(-60\,\text{Faa Maa MAa} + \text{FAa}\left(25\,\text{Maa}^2 + 30\,\text{Maa MAa} + 8\,\text{MAa}^2\right)\right)}{4\,\text{MAa}\,(10\,\text{Faa Maa} - 15\,\text{FAa Maa} + 4\,\text{FAa MAa})} < 0\Bigg)\Bigg)\Bigg) \;||\; \Big(\text{Faa} > 0 \;\&\&\; \text{Maa} > 0 \;\&\&\; \text{MAA} > 0 \;\&\&\; \text{FAa} < \text{Faa} \;\&\&\; 5\,\text{Maa} < 4\,\text{MAa} \;\&\&\; 10\,\text{Faa Maa} < 5\,\text{FAa Maa} + 4\,\text{FAa MAa} \;\&\&\; \text{MAa} < \text{MAA} \;\&\&\; 8\,\text{FAA MAa} < 5\,\text{FAa Maa} + 4\,\text{FAa MAa} \;\&\&\; 2\,\text{MAa}\,(5\,\text{Faa Maa} + 20\,\text{FAA Maa} + 6\,\text{FAa MAa}) < 32\,\text{FAA MAa}^2 + 5\,\text{FAa Maa}\,(5\,\text{Maa} + \text{MAa}) \;\&\&\; 8\,\text{FAA}\,(5\,\text{Maa} - 4\,\text{MAa})\,\text{MAa} + 10\,\text{Faa Maa}\,(6\,\text{MAa} - 5\,\text{MAA}) < \text{FAa}\,(5\,\text{Maa} + 4\,\text{MAa})\,(5\,\text{Maa} + 2\,\text{MAa} - 5\,\text{MAA})\Big)\Bigg)$$

```
(*Including meiotic drive in females*)
Simplify[
 Reduce[{stabcondA, ((λmA1 > 1) || (λma1 > 1)), (((FAA > FAa > Faa > 0) && (Maa > MAa >
                    MAA > 0)) || ((0 < FAA < FAa < Faa) && (0 < Maa < MAa < MAA)))} /.
              reverse /. pAveM → q pYm + (1 - q) pXm /. equilA0 /. wam → 1 /. wAm → 1 /.
        waf → 1 /. wAf → 1 /. αf → 1 / 2 /. αm → 4 / 10 /. Rm → 0 /. Rf → 0]]
```

$$FAa > 0 \;\&\&\; MAa > 0 \;\&\& \left(\left(0 < Faa \;\&\&\; 0 < MAA \;\&\&\; Faa < \frac{1}{10} FAa \left(5 + \frac{4\,MAa}{Maa}\right) \;\&\&\; FAa < FAA \;\&\&\; MAA < MAa \;\&\& \left(\left(FAa\left(4 + \frac{5\,Maa}{MAa}\right) > 8\,FAA \;\&\&\; MAa < Maa \;\&\&\; 5\,Maa \le 8\,MAa\right) \;||\; \left(5\,Maa > 8\,MAa \;\&\&\; FAA + \frac{FAa\left(-60\,Faa\,Maa\,MAa + FAa\left(25\,Maa^2 + 30\,Maa\,MAa + 8\,MAa^2\right)\right)}{4\,MAa\,(10\,Faa\,Maa - 15\,FAa\,Maa + 4\,FAa\,MAa)} < 0\right)\right)\right) \;||\; \left(Faa > 0 \;\&\&\; Maa > 0 \;\&\&\; MAA > 0 \;\&\&\; FAa < Faa \;\&\&\; 5\,Maa < 4\,MAa \;\&\&\; 10\,Faa\,Maa < 5\,FAa\,Maa + 4\,FAa\,MAa \;\&\&\; MAa < MAA \;\&\&\; 8\,FAA\,MAa < 5\,FAa\,Maa + 4\,FAa\,MAa \;\&\&\; 2\,MAa\,(5\,Faa\,Maa + 20\,FAA\,Maa + 6\,FAa\,MAa) < 32\,FAA\,MAa^2 + 5\,FAa\,Maa\,(5\,Maa + MAa) \;\&\&\; 8\,FAA\,(5\,Maa - 4\,MAa)\,MAa + 10\,Faa\,Maa\,(6\,MAa - 5\,MAA) < FAa\,(5\,Maa + 4\,MAa)\,(5\,Maa + 2\,MAa - 5\,MAA)\right)\right)$$

and directional selection in diploids (ploidally antagonistic selection):

```
(*Including meiotic drive in males*)
Simplify[
 Reduce[{stabcondA, ((λmA1 > 1) || (λma1 > 1)), (((0 < FAA < FAa < Faa) && (Maa > MAa >
                    MAA > 0)) || ((FAA > FAa > Faa > 0) && (0 < Maa < MAa < MAA)))} /.
              reverse /. pAveM → q pYm + (1 - q) pXm /. equilA0
           /. wam → 1 /. wAm → 1 /. waf → 1 /. wAf → 1 /. αf → 1 / 2 /. αm → 4 / 10 /. Rm → 0 /.
   Rf → 0]]
```

$$MAa > 0 \;\&\&\; \frac{4\,MAa}{5} < Maa < MAa \;\&\&\; FAa > 0 \;\&\&\; 0 < Faa < \frac{1}{10} FAa\left(5 + \frac{4\,MAa}{Maa}\right) \;\&\&\; FAa < FAA \;\&\&\; FAa\left(4 + \frac{5\,Maa}{MAa}\right) > 8\,FAA \;\&\&\; MAa < MAA < \frac{4\,MAa\,(-15\,Faa\,Maa - 10\,FAA\,Maa + 8\,FAA\,MAa) + FAa\left(25\,Maa^2 + 30\,Maa\,MAa + 8\,MAa^2\right)}{-50\,Faa\,Maa + 5\,FAa\,(5\,Maa + 4\,MAa)}$$

```
(*Including meiotic drive in females*)
Simplify[
 Reduce[{stabcondA, ((λmA1 > 1) || (λma1 > 1)), (((0 < FAA < FAa < Faa) && (Maa > MAa >
                   MAA > 0)) || ((FAA > FAa > Faa > 0) && (0 < Maa < MAa < MAA)))} /.
            reverse /. pAveM → q pYm + (1 - q) pXm /. equilA0
         /. wam → 1 /. wAm → 1 /. waf → 1 /. wAf → 1 /. αf → 6 / 10 /. αm → 1 / 2 /. Rm → 0 /.
   Rf → 0]]
```

$$MAa > 0 \,\&\&\, MAa < Maa < \frac{3\,MAa}{2} \,\&\&\, FAa > 0 \,\&\&\, FAa < Faa < \frac{3\,FAa\,(Maa + MAa)}{5\,Maa} \,\&\&\, 0 < FAA < \frac{2\,FAa\,(Maa + MAa)}{5\,MAa} \,\&\&\, 0 < MAA < MAa$$

R near 1/2:

Because sexually antagonistic selection can allow invasion at R = 0, with λmA>1 (but λma<1), we have to check condition (1) to determine if an unlinked neo-W can invade. At the end of this section, we prove:

Lemma 5: An unlinked neo-W (R=1/2) cannot invade a system with sexually antagonistic selection if selection is directional in each sex and haploid selection is absent.

Unlinked neo-Ws can invade in several other cases, however. We know that if both λmA and λma are greater than one, then invasion will occur for all R (as the leading eigenvalue is bounded between these two values, see “Invasion Conditions”). Scanning the above requirements, we can thus conclude that it is possible for unlinked neo-W chromosomes to invade with:

* heterozygote advantage in males with directional selection in females (no haploid selection)

Recalling that the eigenvalues must decrease with increasing R (see “Invasion Conditions”), we know that sexually antagonistic selection alone (with opposing directional selection in the two sexes) will not allow the spread of a neo-W at equilibrium B under any linkage conditions because both λmA and λma are less than one.

We also know that if both λmA and λma are greater than one, then invasion will occur for all R (as the leading eigenvalue is bounded between these two values, see “Invasion Conditions”). Scanning the above requirements, we can thus conclude that it is possible for unlinked neo-W chromosomes to invade with:

* heterozygote advantage in males with directional selection in females if haploid selection is absent.

Indeed, overdominance in males is required for both λmA>1 and λma>1 in the absence of haploid selection:

```
(*Other forms of selection in males*)Simplify[
 Reduce[{stabcondA, ((λmA1 > 1) && (λma1 > 1)), (((Maa < MAa < MAA) || (Maa > MAa < MAA) ||
            (Maa > MAa > MAA)))} /. reverse /. pAveM → q pYm + (1 - q) pXm /. equilA0
     /. nohap /. Rm → 0 /. Rf → 0]]
(*Overdominance in males*)
Simplify[Reduce[{stabcondA, ((λmA1 > 1) && (λma1 > 1)), (Maa < MAa > MAA)} /. reverse /.
       pAveM → q pYm + (1 - q) pXm /. equilA0
     /. nohap /. Rm → 0 /. Rf → 0]]
```

False

$$FAa > 0 \&\& MAa > 0 \&\& 0 < Maa < MAa \&\& 0 < MAA < \frac{2\,(Faa\,Maa + FAA\,(Maa - MAa))\,MAa - FAa\,Maa\,(Maa + MAa)}{2\,Faa\,Maa - FAa\,(Maa + MAa)} \&\& \left(\left(FAA > 0 \&\& FAa + \frac{FAa\,Maa}{MAa} > 2\,FAA \&\& Faa > \frac{FAa\,Maa}{MAa} \&\& 2\,Faa \le FAa + \frac{FAa\,Maa}{MAa}\right) \,||\, \left(2\,Faa > FAa + \frac{FAa\,Maa}{MAa} \&\& 2\,Faa < FAa + \frac{FAa\,MAa}{Maa} \&\& -\frac{FAa\,Maa\,(-2\,Faa\,MAa + FAa\,(Maa + MAa))}{MAa\,(2\,Faa\,Maa + FAa\,(-3\,Maa + MAa))} < FAA < \frac{FAa\,(Maa + MAa)}{2\,MAa}\right)\right)$$

Various forms of haploid selection are also consistent with both λmA and λma being greater than one (ensuring unlinked neo-W can invade). For example, meiotic drive for the Y linked allele in males skews the sex ratio towards males, allowing a neo-W chromosome to invade (which balances the sex ratio). Meiotic drive in females can also work, if balanced by the right type of diploid selection.

Haploid selection coupled for example with sexual antagonism in diploids:

```
(*Including meiotic drive in males*)
Simplify[
 Reduce[{stabcondA, ((λmA1 > 1) && (λma1 > 1)), (((FAA > FAa > Faa > 0) && (Maa > MAa >
                MAA > 0)) || ((0 < FAA < FAa < Faa) && (0 < Maa < MAa < MAA)))} /.
            reverse /. pAveM → q pYm + (1 - q) pXm /. equilA0
         /. wam → 1 /. wAm → 1 /. waf → 1 /. wAf → 1 /. αm → 4 / 10 /. αf → 1 / 2 /.
    Rm → 1 / 2 /. Rf → 1 / 2]]
```

$$FAa > 0 \&\& MAa > 0 \&\& \left(\left(Maa > MAa \&\& FAa\left(4 + \frac{5\,Maa}{MAa}\right) > 8\,FAA \&\& 3\,MAa + \sqrt{57}\,\sqrt{MAa^2} > 10\,Maa \&\& 0 < MAA \&\& Faa < \frac{1}{10}\,FAa\left(5 + \frac{4\,MAa}{Maa}\right) \&\& FAa < FAA \&\& \frac{5\,FAa\,Maa}{6\,MAa} < Faa \&\& MAA < MAa\right) \,||\, \left(Maa > 0 \&\& MAA > 0 \&\& FAa < Faa \&\& 5\,Maa < 4\,MAa \&\& 10\,Faa\,Maa < 5\,FAa\,Maa + 4\,FAa\,MAa \&\& MAa < MAA \&\& 8\,FAA\,MAa < 5\,FAa\,Maa + 4\,FAa\,MAa \&\& 2\,MAa\,(5\,Faa\,Maa + 20\,FAA\,Maa + 6\,FAa\,MAa) < 32\,FAA\,MAa^2 + 5\,FAa\,Maa\,(5\,Maa + MAa) \&\& 8\,FAA\,(5\,Maa - 4\,MAa)\,MAa + 10\,Faa\,Maa\,(6\,MAa - 5\,MAA) < FAa\,(5\,Maa + 4\,MAa)\,(5\,Maa + 2\,MAa - 5\,MAA)\right)\right)$$

but not if there is only meiotic drive in females
[NOT SHOWN GENERALLY, ALSO TRIED αf→1/10, 4/10, 49/100, 51/100, 6/10, 9/10]:

```
(*Including meiotic drive in females*)
Simplify[
 Reduce[{stabcondA, ((λmA1 > 1) && (λma1 > 1)), (((FAA > FAa > Faa > 0) && (Maa > MAa >
                   MAA > 0)) || ((0 < FAA < FAa < Faa) && (0 < Maa < MAa < MAA)))} /.
             reverse /. pAveM → q pYm + (1 - q) pXm /. equilA0
          /. wam → 1 /. wAm → 1 /. waf → 1 /. wAf → 1 /. αm → 1 / 2 /. αf → 4 / 10 /. Rm → 0 /.
   Rf → 0]]
```

False

Haploid selection (in either sex) also favors the spread of unlinking neo-W with directional selection in diploids (ploidally antagonistic selection):

```
(*Including meiotic drive in males*)
Simplify[
 Reduce[{stabcondA, ((λmA1 > 1) && (λma1 > 1)), (((0 < FAA < FAa < Faa) && (Maa > MAa >
                   MAA > 0)) || ((FAA > FAa > Faa > 0) && (0 < Maa < MAa < MAA)))} /.
             reverse /. pAveM → q pYm + (1 - q) pXm /. equilA0
          /. wam → 1 /. wAm → 1 /. waf → 1 /. wAf → 1 /. αm → 4 / 10 /. αf → 1 / 2 /. Rm → 0 /.
   Rf → 0]]
```

$$MAa > 0 \;\&\&\; Maa > 0 \;\&\&\; FAa > 0 \;\&\&\; FAA > 0 \;\&\&\; MAA > 0 \;\&\&\; 4\,MAa < 5\,Maa \;\&\&\; Maa < MAa \;\&\&\; 5\,FAa\,Maa < 6\,Faa\,MAa \;\&\&\; 10\,Faa\,Maa < 5\,FAa\,Maa + 4\,FAa\,MAa \;\&\&\; FAa < FAA \;\&\&\; 8\,FAA\,MAa < 5\,FAa\,Maa + 4\,FAa\,MAa \;\&\&\; MAa < MAA \;\&\&\; 8\,FAA\,(5\,Maa - 4\,MAa)\,MAa + 10\,Faa\,Maa\,(6\,MAa - 5\,MAA) < FAa\,(5\,Maa + 4\,MAa)\,(5\,Maa + 2\,MAa - 5\,MAA)$$

```
(*Including meiotic drive in females*)
Simplify[
 Reduce[{stabcondA, ((λmA1 > 1) && (λma1 > 1)), (((0 < FAA < FAa < Faa) && (Maa > MAa >
                   MAA > 0)) || ((FAA > FAa > Faa > 0) && (0 < Maa < MAa < MAA)))} /.
             reverse /. pAveM → q pYm + (1 - q) pXm /. equilA0
          /. wam → 1 /. wAm → 1 /. waf → 1 /. wAf → 1 /. αm → 1 / 2 /. αf → 6 / 10 /. Rm → 0 /.
   Rf → 0]]
```

$$FAa > 0 \;\&\&\; MAa > 0 \;\&\&\; 0 < FAA < \frac{2\,FAa\,(Maa + MAa)}{5\,MAa} \;\&\&\; 0 < MAA < MAa \;\&\&\; Faa < \frac{3\,FAa\,(Maa + MAa)}{5\,Maa} \;\&\&\; \left((FAa < Faa \;\&\&\; MAa < Maa \;\&\&\; 4\,Maa \le 5\,MAa) \;||\; \left(4\,Maa > 5\,MAa \;\&\&\; 3\,MAa + \sqrt{57}\,\sqrt{MAa^2} > 8\,Maa \;\&\&\; \frac{4\,FAa\,Maa}{5\,MAa} < Faa\right)\right)$$

Proof of Lemma 5: An unlinked neo-W (R=1/2) cannot invade a system with sexually antagonistic selection if selection is directional in each sex and haploid selection is absent.

We now return explicitly to the case where only one of to the case where only one of λmA or λma is greater than one. In this case (see the “Invasion Condition” section), we showed that a neo-W will invade if and only if the intercept of the primary quadratic is negative:

```
(1 + b + c) /. b -> - ((λmA - χmA) + (λma - χma)) /.
   c -> (λmA - χmA) (λma - χma) - χmA χma // Factor
```

$$1 - \lambda ma - \lambda mA + \lambda ma\,\lambda mA + \chi ma - \lambda mA\,\chi ma + \chi mA - \lambda ma\,\chi mA$$

Without haploid selection, this becomes:

```
interceptA = (1 + b + c) /. b -> - ((λmA1 - χmA1) + (λma1 - χma1)) /.
        c -> (λmA1 - χmA1) (λma1 - χma1) - χmA1 χma1 /. reverse /.
      pAveM → q pYm + (1 - q) pXm /. equilA0 /. nohap /. R → 1 / 2 // Factor
```

$$-\left((Faa\,FAa - 2\,Faa\,FAA + FAa\,FAA)\,MAa\,(-2\,Faa\,Maa + FAa\,Maa + FAa\,MAa)\left(-2\,FAa\,Maa^2 + 2\,Faa\,Maa\,MAa - FAa\,Maa\,MAa + FAa\,MAa^2\right)\right) \Big/ \left(2\left(FAa^2\,Maa^2 + 2\,FAa^2\,Maa\,MAa - 4\,Faa\,FAA\,Maa\,MAa + FAa^2\,MAa^2\right)^2\right)$$

In the following, we will use the equilibrium's validity and stability conditions to define the following two positive terms (see stabcondA without haploid selection):

$$pos1 = \frac{FAa\,(Maa + MAa)}{2\,MAa} - FAA;$$

$$pos2 = \frac{FAa\,(Maa + MAa)}{2\,Maa} - Faa;$$

```
Simplify[
 Reduce[{stabcondA, ((0 < FAA < FAa < Faa) && (0 < Maa < MAa < MAA))} /. equilA0 /. nohap],
 {Rf == 1 / 2, Rm == 1 / 2}]
Simplify[Reduce[{stabcondA, ((FAA > FAa > Faa > 0) && (Maa > MAa > MAA > 0))} /.
    equilA0 /. nohap], {Rf == 1 / 2, Rm == 1 / 2}]
```

False

$$MAa > 0 \;\&\&\; Maa > MAa \;\&\&\; FAA > 0 \;\&\&\; FAa > \frac{2\,FAA\,MAa}{Maa + MAa} \;\&\&\; FAa < FAA \;\&\&\; Faa > 0 \;\&\&\; 2\,Faa < FAa + \frac{FAa\,MAa}{Maa} \;\&\&\; MAA > 0 \;\&\&\; MAa > MAA$$

With pure sexually antagonistic selection with directional selection in each sex, we must also have (FAa Maa-Faa MAa) and (Maa-MAa) positive at this equilibrium (which requires that "a" is favored in males and "A" in females).

Consequently, the intercept can be written as a strictly positive quantity when R = 1/2, and invasion of an unlinked neo-W is not possible with pure sexually antagonistic selection:

$$\left(\left(2\,Maa\,MAa\,pos2\,(2\,Maa\,(FAa\,Maa - Faa\,MAa) + FAa\,(Maa - MAa)\,MAa)\left(2\,\frac{MAa}{(Maa + MAa)}\,Faa\,pos1 + 2\,\frac{Maa}{(Maa + MAa)}\,FAA\,pos2\right)\right) \Big/ \left(2\left(FAa^2\,Maa^2 + 2\,FAa^2\,Maa\,MAa - 4\,Faa\,FAA\,Maa\,MAa + FAa^2\,MAa^2\right)^2\right)\right);$$

```
% / interceptA // Factor
```

1

Corollary of proof: An unlinked neo-W (R=1/2) can only invade a system at equilibrium A if selection is overdominant in males when haploid selection is absent [Not true for equilibrium B, see below]

In the absence of haploid selection, overdominance in males is required for invasion of neo-W with R=1/2 in the case where only λmA>1 (directional selection or underdominance does not work):

```
(*Other forms of selection in males*)
Simplify[Reduce[{stabcondA, (2 Maa (FAa Maa - Faa MAa) + FAa (Maa - MAa) MAa) < 0,
      λmA1 > 1, ((Maa < MAa < MAA) || (Maa > MAa < MAA) || (Maa > MAa > MAA))}
    /. reverse /. pAveM → q pYm + (1 - q) pXm /. equilA0 /. nohap /. Rm → 0 /. Rf → 0]]

(*Overdominance in males*)
Simplify[Reduce[{stabcondA, (2 Maa (FAa Maa - Faa MAa) + FAa (Maa - MAa) MAa) < 0,
      λmA1 > 1, (Maa < MAa > MAA)}
    /. reverse /. pAveM → q pYm + (1 - q) pXm /. equilA0 /. nohap /. Rm → 0 /. Rf → 0]]
```

False

$$FAa > 0 \,\&\&\, MAa > 0 \,\&\&\, 0 < MAA < \frac{2\,(Faa\,Maa + FAA\,(Maa - MAa))\,MAa - FAa\,Maa\,(Maa + MAa)}{2\,Faa\,Maa - FAa\,(Maa + MAa)} \,\&\&$$
$$\Bigg(\bigg(0 < FAA < \frac{FAa\,(Maa + MAa)}{2\,MAa} \,\&\&\, 2\,Faa \le FAa + \frac{FAa\,Maa}{MAa} \,\&\&$$
$$\bigg(\Big(2\,Maa > MAa \,\&\&\, 2\,Faa + \frac{FAa\,MAa}{Maa} > FAa + \frac{2\,FAa\,Maa}{MAa} \,\&\&\, Maa < MAa\Big) \,||$$
$$(Faa > 0 \,\&\&\, Maa > 0 \,\&\&\, 2\,Maa \le MAa)\bigg)\bigg) \,||$$
$$\bigg(-\frac{FAa\,Maa\,(-2\,Faa\,MAa + FAa\,(Maa + MAa))}{MAa\,(2\,Faa\,Maa + FAa\,(-3\,Maa + MAa))} < FAA < \frac{FAa\,(Maa + MAa)}{2\,MAa} \,\&\&$$
$$2\,Faa < FAa + \frac{FAa\,MAa}{Maa} \,\&\&\, (Maa < MAa \,||\, 2\,Maa \le MAa) \,\&\&$$
$$2\,Faa > FAa + \frac{FAa\,Maa}{MAa} \,\&\&\, (Maa > 0 \,||\, 2\,Maa > MAa)\bigg)\Bigg)$$

neo-W equilB (r ~ 0)

The four terms that define the characteristic polynomial are

```
{λmA1, λma1, χmA1, χma1} /. reverse /. pAveM → q pYm + (1 - q) pXm /. equilB0 // Factor
```

$$\left\{-\frac{-2\,FAa\,wam\,\alpha f - FAA\,wAm\,\alpha m + 2\,FAa\,wam\,\alpha f\,\alpha m}{2\,FAA\,wAm\,\alpha m},\right.$$
$$-\frac{waf\,(-Faa\,wam + Faa\,wam\,\alpha m - 2\,FAa\,wAm\,\alpha m + 2\,FAa\,wAm\,\alpha f\,\alpha m)}{2\,FAA\,wAf\,wAm\,\alpha m},$$
$$\left.-\frac{FAa\,R\,wam\,\alpha f\,(-1 + \alpha m)}{FAA\,wAm\,\alpha m},\ -\frac{FAa\,R\,waf\,(-1 + \alpha f)}{FAA\,wAf}\right\}$$

```
charpolyextB =
  λ^2 + b λ + c /. b -> -((λmA - χmA) + (λma - χma)) /. c -> (λmA - χmA) (λma - χma) -
       χmA χma /. {λmA -> λmA1, χmA -> χmA1, λma -> λma1, χma -> χma1} /.
    reverse /. pAveM → q pYm + (1 - q) pXm /. equilB0 // Factor;
```

In the following, we will use the following to help interpret the results:

DEFINING A FEW TERMS TO INTERPRET THE ABOVE:

With A fixed in females and a in males (equilB0), the mean fitness in haploids is:

```
wbarHapFemale /. SUBS /. subequil /. equilB0 // Factor
```

wAf

```
wbarHapMale /. SUBS /. subequil /. equilB0 // Factor
```

$\mathrm{wam} - \mathrm{wam}\,\alpha\mathrm{m} + \mathrm{wAm}\,\alpha\mathrm{m}$

The mean fitness in females times the frequency of females (i.e., the normalizing term in the recursions) is:

```
wbarF /. SUBS /. subequil /. equilB0 // Factor
```

$$\frac{\mathrm{FAA}\ \mathrm{wAm}\ \alpha\mathrm{m}}{\mathrm{wam} - \mathrm{wam}\ \alpha\mathrm{m} + \mathrm{wAm}\ \alpha\mathrm{m}}$$

The frequency of diploid females is:

```
freqFemale /. SUBS /. subequil /. equilB0 // Factor
```

$$\frac{\mathrm{wAm}\ \alpha\mathrm{m}}{\mathrm{wam} - \mathrm{wam}\ \alpha\mathrm{m} + \mathrm{wAm}\ \alpha\mathrm{m}}$$

The mean fitness of diploid females:

```
wbarDipFemale /. equilB0 // Factor
```

FAA

R near 0:

If the neo-W is also closely linked (R near 0 but potentially R>r), the quadratic factors to give the two eigenvalues, λmA1 and λma

```
charpolyextB /. R → 0 /. equilB0 // Factor
```

$$\frac{1}{4\ \mathrm{FAA}^2\ \mathrm{wAf}\ \mathrm{wAm}^2\ \alpha\mathrm{m}^2}(-2\ \mathrm{FAa}\ \mathrm{wam}\ \alpha\mathrm{f} - \mathrm{FAA}\ \mathrm{wAm}\ \alpha\mathrm{m} + 2\ \mathrm{FAa}\ \mathrm{wam}\ \alpha\mathrm{f}\ \alpha\mathrm{m} + 2\ \mathrm{FAA}\ \mathrm{wAm}\ \alpha\mathrm{m}\ \lambda)$$
$$(-\mathrm{Faa}\ \mathrm{waf}\ \mathrm{wam} + \mathrm{Faa}\ \mathrm{waf}\ \mathrm{wam}\ \alpha\mathrm{m} - 2\ \mathrm{FAa}\ \mathrm{waf}\ \mathrm{wAm}\ \alpha\mathrm{m} + 2\ \mathrm{FAa}\ \mathrm{waf}\ \mathrm{wAm}\ \alpha\mathrm{f}\ \alpha\mathrm{m} + 2\ \mathrm{FAA}\ \mathrm{wAf}\ \mathrm{wAm}\ \alpha\mathrm{m}\ \lambda)$$

```
λ /. Solve[% == 0, λ] // Factor
```

$$\left\{\frac{2\ \mathrm{FAa}\ \mathrm{wam}\ \alpha\mathrm{f} + \mathrm{FAA}\ \mathrm{wAm}\ \alpha\mathrm{m} - 2\ \mathrm{FAa}\ \mathrm{wam}\ \alpha\mathrm{f}\ \alpha\mathrm{m}}{2\ \mathrm{FAA}\ \mathrm{wAm}\ \alpha\mathrm{m}},\right.$$
$$\left.-\frac{\mathrm{waf}\ (-\mathrm{Faa}\ \mathrm{wam} + \mathrm{Faa}\ \mathrm{wam}\ \alpha\mathrm{m} - 2\ \mathrm{FAa}\ \mathrm{wAm}\ \alpha\mathrm{m} + 2\ \mathrm{FAa}\ \mathrm{wAm}\ \alpha\mathrm{f}\ \alpha\mathrm{m})}{2\ \mathrm{FAA}\ \mathrm{wAf}\ \mathrm{wAm}\ \alpha\mathrm{m}}\right\}$$

Interpretation of the λmA and λma terms:

A neo-W carrying the A allele would have mean representation in the pool of female gametes (accounting for meiotic drive) of:
[Note that this is summed over all gametes donated by males because the W is assumed dominant and always produces females.]

$$\left(FAA \frac{wAf}{wbarHapFemale} pXm (1-q) \frac{wAm}{wbarHapMale} + \frac{\alpha f}{1/2} FAa \frac{wAf}{wbarHapFemale} (1-pXm)(1-q) \frac{wam}{wbarHapMale} + FAA \frac{wAf}{wbarHapFemale} pYm\, q \frac{wAm}{wbarHapMale} + \frac{\alpha f}{1/2} FAa \frac{wAf}{wbarHapFemale} (1-pYm)\, q \frac{wam}{wbarHapMale}\right) \text{ /. SUBS /. subequil /. equilB0 // Factor}$$

$$\frac{-2\, FAa\, wam\, \alpha f - FAA\, wAm\, \alpha m + 2\, FAa\, wam\, \alpha f\, \alpha m}{-wam + wam\, \alpha m - wAm\, \alpha m}$$

This divided by the mean fitness of the wildtype M bearing females (wbarDipFemale=FAA) divided by 2*freqfemale gives the leading eigenvalue in this case:

$$\frac{(\% / wbarDipFemale)}{2\, freqfemale} \text{ /. SUBS /. subequil /. equilB0 // Factor;}$$

```
% - λmA1 /. freqfemale → 1 - ξ /. reverse /. pAveM → q pYm + (1 - q) pXm /. equilB0 // Factor
```

0

The 2 freqfemale in the denominator describes a sex ratio advantage of being in the rarer sex. If the combination of meiotic drive and pollen selection in males causes females to be rare, then the neo-W will gain an advantage.

A neo-W carrying the a allele would have mean representation in the pool of female gametes (accounting for meiotic drive) of:

$$\left(\frac{1-\alpha f}{1/2} FAa \frac{waf}{wbarHapFemale} pXm (1-q) \frac{wAm}{wbarHapMale} + Faa \frac{waf}{wbarHapFemale} (1-pXm)(1-q) \frac{wam}{wbarHapMale} + \frac{1-\alpha f}{1/2} FAa \frac{waf}{wbarHapFemale} pYm\, q \frac{wAm}{wbarHapMale} + Faa \frac{waf}{wbarHapFemale} (1-pYm)\, q \frac{wam}{wbarHapMale}\right) \text{ /. SUBS /. subequil /. equilB0 // Factor}$$

$$\frac{waf\, (-Faa\, wam + Faa\, wam\, \alpha m - 2\, FAa\, wAm\, \alpha m + 2\, FAa\, wAm\, \alpha f\, \alpha m)}{wAf\, (-wam + wam\, \alpha m - wAm\, \alpha m)}$$

This divided by the mean fitness of the wildtype M bearing females (wbarDipFemale=FAA) divided by 2*freqfemale gives the leading eigenvalue in this case:

$$\frac{(\% / wbarDipFemale)}{2\, freqfemale} \text{ /. SUBS /. subequil /. equilB0 // Factor;}$$

```
% - λma1 /. freqfemale → 1 - ξ /. reverse /. pAveM → q pYm + (1 - q) pXm /. equilB0 // Factor
```

0

A neo-W arriving into the system can invade under some conditions:

In the absence of haploid selection, invasion occurs if female fitness is higher with the “a” allele, despite that allele being fixed in males:

```
{λmA1, λma1} /. reverse /. pAveM → q pYm + (1 - q) pXm /. equilB0 /. nohap // Simplify
```

$$\left\{\frac{\text{FAa}+\text{FAA}}{2\ \text{FAA}}, \frac{\text{Faa}+\text{FAa}}{2\ \text{FAA}}\right\}$$

This is impossible with purely sexually antagonistic selection, where “a” is directionally favored in males (Y-a is fixed) and “A” is directionally favored in females. Essentially, the X is already as specialized as possible for the female beneficial allele (X-A is fixed), and the neo-W often makes daughters with the Y-a haplotype, increasing the flow of “a” alleles into the daughters, which reduces fitness of those daughters.

If selection doesn’t uniformly favor “A” in females, however, the neo-W can spread at this equilibium if FAa>FAA (from λmA) or if $\frac{\text{Faa}}{2}+\frac{\text{FAa}}{2}>\text{FAA}$ (from λma). If FAa>FAA, the implication is that “a” is favored in females despite “A” being fixed on the X, which must require that “X-A” is sufficiently favored in males to drive the frequency of “X-A” to one (specifically, from the stability conditions, we must have $\frac{\text{MAa}}{(\text{Maa}+\text{MAa})/2}>\frac{\text{FAa}}{\text{FAA}}$).

Even if FAa<FAA, the neo-W can spread alongside the “a” allele if there is sufficiently strong underdominance in females with Faa>FAa, such that $\frac{\text{Faa}}{2}+\frac{\text{FAa}}{2}>\text{FAA}$. In this case, “a” is not favored in females near the equilibrium where females are AA (comparing Aa to AA genotypes) and yet the neo-W with “a” can spread because it produces female aa individuals at a high rate by capturing Y-a haplotypes and Faa is high enough.

Confirming that sexually antagonistic selection alone does not work:

```
Simplify[Reduce[
  {stabcondB, ((λmA1 > 1) || (λma1 > 1)), (((FAA > FAa > Faa > 0) && (Maa > MAa > MAA >
            0)) || ((0 < FAA < FAa < Faa) && (0 < Maa < MAa < MAA)))} /. reverse /.
      pAveM → q pYm + (1 - q) pXm /. equilB0 /. nohap /. Rm → 0 /. Rf → 0]]
```

```
False
```

Other forms of selection allow the neo-W to invade an internally stable equilibrium, even without haploid selection:

```
Simplify[
 Reduce[{stabcondB, ((λmA1 > 1) || (λma1 > 1))} /. reverse /. pAveM → q pYm + (1 - q) pXm /.
     equilB0 /. nohap /. Rm → 0 /. Rf → 0]]
```

$$\text{FAA}>0\ \&\&\ \text{MAa}>\text{MAA}\ \&\&\ \text{MAA}>0\ \&\&\ \text{Maa}>0\ \&\&\ \frac{2\ \text{FAA}\ \text{MAa}}{\text{FAa}}>\text{Maa}+\text{MAa}\ \&\&$$
$$((\text{Faa}+\text{FAa}>2\ \text{FAA}\ \&\&\ 0<\text{FAa}\le\text{FAA})\ ||\ (\text{Faa}>0\ \&\&\ \text{FAA}<\text{FAa}<2\ \text{FAA}))$$

such as heterozygote advantage in males with directional selection in females:

```
Simplify[Reduce[{stabcondB, λmA1 > 1, (((0 < FAA < FAa < Faa) && (Maa < MAa > MAA > 0)))}
    /. reverse /. pAveM → q pYm + (1 - q) pXm /. equilB0 /. nohap /. Rm → 0 /. Rf → 0]]
Simplify[Reduce[{stabcondB, λma1 > 1, (((0 < FAA < FAa < Faa) && (Maa < MAa > MAA > 0)))}
    /. reverse /. pAveM → q pYm + (1 - q) pXm /. equilB0 /. nohap /. Rm → 0 /. Rf → 0]]
```

MAa > 0 && 0 < Maa < MAa && FAA > 0 && FAA < FAa < $\frac{2\ \text{FAA MAa}}{\text{Maa} + \text{MAa}}$ && 0 < MAA < MAa && Faa > FAa

FAA > 0 && FAA < FAa < 2 FAA && MAa > 0 &&
Maa > 0 && $\frac{2\ \text{FAA MAa}}{\text{FAa}}$ > Maa + MAa && Faa > FAa && 0 < MAA < MAa

or underdominance in females with directional selection in males:

```
Simplify[Reduce[{stabcondB, λmA1 > 1,
      (((FAA > FAa < Faa > 0) && ((0 < Maa < MAa < MAA) || (Maa > MAa > MAA > 0))))}
    /. reverse /. pAveM → q pYm + (1 - q) pXm /. equilB0 /. nohap /. Rm → 0 /. Rf → 0]]
Simplify[Reduce[{stabcondB, λma1 > 1,
      (((FAA > FAa < Faa > 0) && ((0 < Maa < MAa < MAA) || (Maa > MAa > MAA > 0))))}
    /. reverse /. pAveM → q pYm + (1 - q) pXm /. equilB0 /. nohap /. Rm → 0 /. Rf → 0]]
```

False

FAA > 0 && 0 < FAa < FAA && MAa > 0 && Maa > MAa &&
$\frac{2\ \text{FAA MAa}}{\text{FAa}}$ > Maa + MAa && Faa + FAa > 2 FAA && 0 < MAA < MAa

but not heterozygote advantage in females with directional selection in males (which works for equilibrium A considered above):

```
Simplify[Reduce[{stabcondB, λmA1 > 1,
      (((FAA < FAa > Faa > 0) && ((0 < Maa < MAa < MAA) || (Maa > MAa > MAA > 0))))}
    /. reverse /. pAveM → q pYm + (1 - q) pXm /. equilB0 /. nohap /. Rm → 0 /. Rf → 0]]
Simplify[Reduce[{stabcondB, λma1 > 1,
      (((FAA < FAa > Faa > 0) && ((0 < Maa < MAa < MAA) || (Maa > MAa > MAA > 0))))}
    /. reverse /. pAveM → q pYm + (1 - q) pXm /. equilB0 /. nohap /. Rm → 0 /. Rf → 0]]
```

False

False

Haploid selection also increases the types of selection in the diploid phase that are consistent with neo-W invasion, including sexual antagonism in diploids:

```
(*Including meiotic drive in males*)
Simplify[
 Reduce[{stabcondB, ((λmA1 > 1) || (λma1 > 1)), (((FAA > FAa > Faa > 0) && (Maa > MAa >
                      MAA > 0)) || ((0 < FAA < FAa < Faa) && (0 < Maa < MAa < MAA)))} /.
          reverse /. pAveM → q pYm + (1 - q) pXm /. equilB0
       /. wam → 1 /. wAm → 1 /. waf → 1 /. wAf → 1 /. αf → 1 / 2 /. Rm → 0 /. Rf → 0]]
```

$$FAA > 0 \;\&\&\; MAA > 0 \;\&\&\; Maa < 2\left(-1+\frac{2\,FAA}{FAa}\right) MAa\,\alpha m \;\&\& \left(\left(Faa > FAa \;\&\&\; 0 < Maa \;\&\&\; 0 < \alpha m \;\&\&\; FAa < 2\,FAA \;\&\&\; FAA < FAa \;\&\&\; MAa < MAA \;\&\&\; \frac{MAA}{2-2\,\alpha m} < MAa \;\&\&\; 2\,\alpha m < 1\right) \;||\; \left(Faa > 0 \;\&\&\; FAa > 0 \;\&\&\; Faa < FAa \;\&\&\; FAa < FAA \;\&\&\; MAa < Maa \;\&\&\; MAA < MAa \;\&\&\; (FAa + FAA)\,\alpha m < FAa \;\&\&\; FAa + 2\,FAa\,\alpha m < 4\,FAA\,\alpha m\right)\right)$$

```
(*Including meiotic drive in females*)
Simplify[
 Reduce[{stabcondB, ((λmA1 > 1) || (λma1 > 1)), (((FAA > FAa > Faa > 0) && (Maa > MAa >
                      MAA > 0)) || ((0 < FAA < FAa < Faa) && (0 < Maa < MAa < MAA)))} /.
          reverse /. pAveM → q pYm + (1 - q) pXm /. equilB0
       /. wam → 1 /. wAm → 1 /. waf → 1 /. wAf → 1 /. αm → 1 / 2 /. Rm → 0 /. Rf → 0]]
```

$$FAA > 0 \;\&\&\; FAa > 0 \;\&\&\; MAa > 0 \;\&\&\; Maa > 0 \;\&\&\; Faa > 0 \;\&\&\; MAA > 0 \;\&\&\; FAA < 2\,FAa \;\&\&\; FAa < FAA \;\&\&\; FAA < 2\,FAa\,\alpha f \;\&\&\; \alpha f < 1 \;\&\&\; MAa < Maa \;\&\&\; FAA\,MAa + FAa\,(Maa + MAa)\,(-1+\alpha f) > 0 \;\&\&\; Faa < FAa \;\&\&\; MAA < MAa$$

and even directional selection in diploids (ploidally antagonistic selection):

```
(*Including meiotic drive in males*)
Simplify[
 Reduce[{stabcondB, ((λmA1 > 1) || (λma1 > 1)), (((0 < FAA < FAa < Faa) && (Maa > MAa >
                      MAA > 0)) || ((FAA > FAa > Faa > 0) && (0 < Maa < MAa < MAA)))} /.
          reverse /. pAveM → q pYm + (1 - q) pXm /. equilB0
       /. wam → 1 /. wAm → 1 /. waf → 1 /. wAf → 1 /. αf → 1 / 2 /. Rm → 0 /. Rf → 0]]
```

$$FAA > 0 \;\&\&\; MAA > 0 \;\&\& \left(\left(0 < Faa \;\&\&\; 0 < FAa \;\&\&\; 0 < Maa \;\&\&\; Faa < FAa \;\&\&\; FAa < FAA \;\&\&\; MAa < MAA \;\&\&\; \frac{MAA}{2-2\,\alpha m} < MAa \;\&\& \left(\left(0 < \alpha m \;\&\&\; Maa < 2\left(-1+\frac{2\,FAA}{FAa}\right) MAa\,\alpha m \;\&\&\; \frac{FAa}{2\,FAa-4\,FAA} + \alpha m \le 0\right) \;||\; \left(\frac{FAa}{2\,FAa-4\,FAA} + \alpha m > 0 \;\&\&\; Maa < MAa \;\&\&\; \alpha m < \frac{FAa}{FAa+FAA}\right)\right)\right) \;||\; \left(Faa > \frac{(FAa - 2\,FAA)\,\alpha m}{-1+\alpha m} \;\&\&\; MAa > \frac{MAA}{2-2\,\alpha m} \;\&\&\; 3\,FAa < 4\,FAA \;\&\&\; \frac{FAa}{2\,FAa-4\,FAA} + \alpha m > 0 \;\&\&\; FAA < FAa \;\&\&\; Maa < 2\left(-1+\frac{2\,FAA}{FAa}\right) MAa\,\alpha m \;\&\&\; MAa < Maa \;\&\&\; \alpha m < 1\right)\right)$$

```
(*Including meiotic drive in females*)
Simplify[
 Reduce[{stabcondB, ((λmA1 > 1) || (λma1 > 1)), (((0 < FAA < FAa < Faa) && (Maa > MAa >
                    MAA > 0)) || ((FAA > FAa > Faa > 0) && (0 < Maa < MAa < MAA)))} /.
            reverse /. pAveM → q pYm + (1 - q) pXm /. equilB0
          /. wam → 1 /. wAm → 1 /. waf → 1 /. wAf → 1 /. αm → 1 / 2 /. Rm → 0 /. Rf → 0]]
```

$$FAA > 0 \&\& FAa > FAA \&\& \frac{FAA}{FAa} + 2\,\alpha f > 2 \&\& \alpha f < 1 \&\& MAa > 0 \&\&$$
$$MAa < Maa < -\frac{MAa\,(FAA + FAa\,(-1 + \alpha f))}{FAa\,(-1 + \alpha f)} \&\& Faa > FAa \&\& 0 < MAA < MAa$$

R near 1/2:

Recalling that the eigenvalues must decrease with increasing R (see “Invasion Conditions”), we know that sexually antagonistic selection alone (with opposing directional selection in the two sexes) will not allow the spread of a neo-W at equilibrium B under any linkage conditions because both λmA and λma are less than one.

We also know that if both λmA and λma are greater than one, then invasion will occur for all R (as the leading eigenvalue is bounded between these two values, see “Invasion Conditions”). Scanning the above requirements, we can thus conclude that it is possible for unlinked neo-W chromosomes to invade with:

 * heterozygote advantage in males with directional selection in females

Indeed, in the absence of haploid selection, overdominance in males is required for both λmA>1 and λma>1:

```
(*Other forms of selection in males*)Simplify[
 Reduce[{stabcondB, ((λmA1 > 1) && (λma1 > 1)), (((Maa < MAa < MAA) || (Maa > MAa < MAA) ||
          (Maa > MAa > MAA)))} /. reverse /. pAveM → q pYm + (1 - q) pXm /. equilB0
     /. nohap /. Rm → 0 /. Rf → 0]]
(*Overdominance in males*)
Simplify[Reduce[{stabcondB, ((λmA1 > 1) && (λma1 > 1)), (Maa < MAa > MAA)} /. reverse /.
       pAveM → q pYm + (1 - q) pXm /. equilB0
     /. nohap /. Rm → 0 /. Rf → 0]]
```

False

$$FAA > 0 \&\& FAA < FAa < 2\,FAA \&\& MAa > 0 \&\& Maa > 0 \&\&$$
$$\frac{2\,FAA\,MAa}{FAa} > Maa + MAa \&\& 0 < MAA < MAa \&\& Faa + FAa > 2\,FAA$$

Various forms of haploid selection are also consistent with both λmA and λma being greater than one (ensuring unlinked neo-W can invade). For example, meiotic drive for the Y linked allele in males skews the sex ratio towards males, allowing a neo-W chromosome to invade (which balances the sex ratio). Meiotic drive in females can also work, if balanced by the right type of diploid selection.

Haploid selection coupled for example with sexual antagonism in diploids:

```
(*Including meiotic drive in males*)
Simplify[
 Reduce[{stabcondB, ((λmA1 > 1) && (λma1 > 1)), (((FAA > FAa > Faa > 0) && (Maa > MAa >
                    MAA > 0)) || ((0 < FAA < FAa < Faa) && (0 < Maa < MAa < MAA)))} /.
          reverse /. pAveM → q pYm + (1 - q) pXm /. equilB0
        /. wam → 1 /. wAm → 1 /. waf → 1 /. wAf → 1 /. αf → 1 / 2 /. Rm → 1 / 2 /. Rf → 1 / 2]]
```

$$FAA > 0 \,\&\&\, MAA > 0 \,\&\&\, Maa < 2\left(-1+\frac{2\,FAA}{FAa}\right) MAa\,\alpha m \,\&\& \left(\left(MAa > MAA \,\&\&\, 0 < FAa \,\&\&\, Faa < FAa \,\&\&\, FAa < FAA \,\&\&\, \frac{FAa}{2\,FAa-4\,FAA}+\alpha m > 0 \,\&\&\, MAa < Maa \,\&\&\, 2\,FAA^2\,\alpha m < FAa\,FAA \,\&\&\, \frac{(FAa-2\,FAA)\,\alpha m}{-1+\alpha m} < Faa\right) \,||\, \left(Faa > FAa \,\&\&\, 0 < Maa \,\&\&\, 0 < \alpha m \,\&\&\, FAa < 2\,FAA \,\&\&\, FAA < FAa \,\&\&\, MAa < MAA \,\&\&\, \frac{MAA}{2-2\,\alpha m} < MAa \,\&\&\, 2\,\alpha m < 1\right)\right)$$

but not if there is only meiotic drive in females:

```
(*Including meiotic drive in females*)
Simplify[
 Reduce[{stabcondB, ((λmA1 > 1) && (λma1 > 1)), (((FAA > FAa > Faa > 0) && (Maa > MAa >
                    MAA > 0)) || ((0 < FAA < FAa < Faa) && (0 < Maa < MAa < MAA)))} /.
          reverse /. pAveM → q pYm + (1 - q) pXm /. equilB0
        /. wam → 1 /. wAm → 1 /. waf → 1 /. wAf → 1 /. αm → 1 / 2 /. Rm → 1 / 2 /. Rf → 1 / 2]]
```

```
False
```

Haploid selection (in either sex) also favors the spread of unlinking neo-W with directional selection in diploids (ploidally antagonistic selection):

```
(*Including meiotic drive in males*)
Simplify[
 Reduce[{stabcondB, ((λmA1 > 1) && (λma1 > 1)), (((0 < FAA < FAa < Faa) && (Maa > MAa >
                    MAA > 0)) || ((FAA > FAa > Faa > 0) && (0 < Maa < MAa < MAA)))} /.
          reverse /. pAveM → q pYm + (1 - q) pXm /. equilB0
        /. wam → 1 /. wAm → 1 /. waf → 1 /. wAf → 1 /. αf → 1 / 2 /. Rm → 1 / 2 /. Rf → 1 / 2]]
```

$$FAA > 0 \,\&\&\, MAA > 0 \,\&\&\, 0 < FAa < FAA \,\&\&\, \frac{MAA}{2-2\,\alpha m} < MAa < MAA \,\&\&\, \frac{(FAa-2\,FAA)\,\alpha m}{-1+\alpha m} < Faa < FAa \,\&\&\, \left(\left(Maa > 0 \,\&\&\, Maa < MAa \,\&\&\, \frac{FAa}{2\,FAa-4\,FAA}+\alpha m > 0 \,\&\&\, \frac{FAa}{FAA} > 2\,\alpha m\right) \,||\, \left(0 < Maa < 2\left(-1+\frac{2\,FAA}{FAa}\right) MAa\,\alpha m \,\&\&\, 0 < \alpha m \le -\frac{FAa}{2\,FAa-4\,FAA}\right)\right)$$

```
(*Including meiotic drive in females*)
Simplify[
 Reduce[{stabcondB, ((λmA1 > 1) && (λma1 > 1)), (((0 < FAA < FAa < Faa) && (Maa > MAa >
                    MAA > 0)) || ((FAA > FAa > Faa > 0) && (0 < Maa < MAa < MAA)))} /.
           reverse /. pAveM → q pYm + (1 - q) pXm /. equilB0
        /. wam → 1 /. wAm → 1 /. waf → 1 /. wAf → 1 /. αm → 1 / 2 /. Rm → 1 / 2 /. Rf → 1 / 2]]
```

FAA > 0 && MAa > 0 && 0 < MAA &&
FAa (FAA MAa + FAa (Maa + MAa) (-1 + αf)) (-1 + αf) < 0 && MAa < Maa && MAA < MAa &&
$\left(\left(2\,\text{FAa} < 3\,\text{FAA}\ \&\&\ \text{FAA} < \text{FAa}\ \&\&\ \left(\left(\text{Faa} > 2\,(\text{FAA} + \text{FAa}\,(-1 + \alpha f))\ \&\&\ \frac{\text{FAA}}{\text{FAa}} + \alpha f > \frac{3}{2}\ \&\&\ \alpha f < 1\right) \,||\, \left(\text{Faa} > \text{FAa}\ \&\&\ \frac{\text{FAA}}{\text{FAa}} + 2\,\alpha f > 2\ \&\&\ \frac{\text{FAA}}{\text{FAa}} + \alpha f \le \frac{3}{2}\right)\right)\right) \,||\, \left(\text{FAa} < 2\,\text{FAA}\ \&\&\ 3\,\text{FAA} \le 2\,\text{FAa}\ \&\&\ \left(\left(\text{Faa} > 2\,(\text{FAA} + \text{FAa}\,(-1 + \alpha f))\ \&\&\ \frac{\text{FAA}}{\text{FAa}} + \alpha f \ge \frac{3}{2}\ \&\&\ \alpha f < 1\right) \,||\, \left(\text{Faa} > \text{FAa}\ \&\&\ \frac{\text{FAA}}{\text{FAa}} + 2\,\alpha f > 2\ \&\&\ \frac{\text{FAA}}{\text{FAa}} + \alpha f < \frac{3}{2}\right)\right)\right) \,||\, \left(\text{FAa} \ge 2\,\text{FAA}\ \&\&\ \alpha f < 1\ \&\&\ \text{Faa} > \text{FAa}\ \&\&\ \frac{\text{FAA}}{\text{FAa}} + 2\,\alpha f > 2\right)\right)$

Even if only one of λmA or λma is greater than one, invasion is still possible with R = 1/2, as explored in the Figures. We show below that this can allow the invasion of unlinked neo-W alleles without haploid selection with underdominance in females or overdominance in males:

Lemma 6: Without haploid selection, an unlinked neo-W (R=1/2) can invade only if there is underdominance in females or overdominance in males.

An example where a tightly linked XY can be invaded by an unlinked neo-W with ploidally antagonistic selection:

```
tryFAA = 1.1;
tryFAa = 1;
tryFaa = 0.9;
tryMAA = 1.1;
tryMAa = 1;
tryMaa = 0.9;
trywAf = 1;
trywaf = 1;
trywam = 1;
tryαf = 0.5;
tryαm = 0.5;
tryR = 1 / 2;
subpar = {FAA → tryFAA, FAa → tryFAa, Faa → tryFaa,
   MAA → tryMAA, MAa → tryMAa, Maa → tryMaa, wAf -> trywAf, waf -> trywaf,
   wam -> trywam, αf -> tryαf, αm -> tryαm, R -> tryR, Rm -> tryR, Rf -> tryR};
Reduce[{λ == 1.1, charpolyextB == 0, simpcond} /. subpar]
```

(wAm == 0.258014 || wAm == 0.688166) && λ == 1.1

An example where a tightly linked XY can be invaded by an unlinked neo-W with overdominant selection in males (no haploid selection):

```
tryFAa = 1;
tryFaa = 1.1;
tryMAA = 0.8;
tryMAa = 1;
tryMaa = 0.8;
trywAf = 1;
trywaf = 1;
trywAm = 1;
trywam = 1;
tryαf = 0.5;
tryαm = 0.5;
tryR = 1 / 2;
subpar = {FAa → tryFAa, Faa → tryFaa, MAA → tryMAA,
   MAa → tryMAa, Maa → tryMaa, wAf -> trywAf, waf -> trywaf, wAm -> trywAm,
   wam -> trywam, αf -> tryαf, αm -> tryαm, R -> tryR, Rm -> tryR, Rf -> tryR};
Reduce[{λ == 1.1, charpolyextB == 0, simpcond, FAA < 1, Faa > 1} /. subpar]
```

```
(FAA == 0.227273 || FAA == 0.916667) && λ == 1.1
```

An example where a tightly linked XY can be invaded by an unlinked neo-W with sexually antagonistic selection and haploid selection:

```
tryFAA = 1.1;
tryFAa = 1;
tryFaa = 0.98;
tryMAA = 0.8;
tryMAa = 1;
tryMaa = 1.01;
trywAf = 1;
trywaf = 1;
trywam = 1;
tryαf = 0.5;
tryαm = 0.5;
tryR = 1 / 2;
subpar = {FAA → tryFAA, FAa → tryFAa, Faa → tryFaa,
   MAA → tryMAA, MAa → tryMAa, Maa → tryMaa, wAf -> trywAf, waf -> trywaf,
   wam -> trywam, αf -> tryαf, αm -> tryαm, R -> tryR, Rm -> tryR, Rf -> tryR};
Reduce[{λ == 1.1, charpolyextB == 0, interceptB < 0, simpcond} /. subpar]
```

```
(wAm == 0.268933 || wAm == 0.718914) && λ == 1.1
```

Proof of Lemma 6: Without haploid selection, an unlinked neo-W (R=1/2) can invade only if there is underdominance in females or overdominance in males.

We showed above that for both λmA and λma to be greater than one without haploid selection requires overdominance in males. This allows the invasion of unlinked neo-W chromosomes.

Here we consider when an unlinked neo-W can spread with only one of λmA or λma greater than one. In this case (see the “Invasion Condition” section), we showed that a neo-W will invade if and only if the intercept of the primary quadratic is negative:

```
(1 + b + c) /. b -> - ((λmA - χmA) + (λma - χma)) /.
  c -> (λmA - χmA) (λma - χma) - χmA χma // Factor
```

$1 - \lambda ma - \lambda mA + \lambda ma\,\lambda mA + \chi ma - \lambda mA\,\chi ma + \chi mA - \lambda ma\,\chi mA$

Without haploid selection, this becomes:

```
interceptB = (1 + b + c) /. b -> - ((λmA1 - χmA1) + (λma1 - χma1)) /.
      c -> (λmA1 - χmA1) (λma1 - χma1) - χmA1 χma1 /. reverse /.
    pAveM → q pYm + (1 - q) pXm /. equilB0 /. nohap /. R → 1 / 2 // Factor
```

$$\frac{\text{Faa FAa} - 2\,\text{Faa FAA} - 3\,\text{FAa FAA} + 4\,\text{FAA}^2}{8\,\text{FAA}^2}$$

For invasion, the intercept must be negative, requiring that FAa be larger than:

```
Solve[interceptB == 0, FAa] // Flatten
```

$$\left\{\text{FAa} \to \frac{2\,(\text{Faa} - 2\,\text{FAA})\,\text{FAA}}{\text{Faa} - 3\,\text{FAA}}\right\}$$

The invasion of an unlinked neo-W into a stable system at equilibrium B is thus possible without over-dominance in males, but only if there is underdominance in females:

```
(*Underdominance in females without overdominance in males*)Simplify[
 Reduce[{stabcondB, (Faa FAa - 2 Faa FAA - 3 FAa FAA + 4 FAA^2)/(8 FAA^2) < 0, ((λmA1 > 1) || (λma1 > 1)),
     ((Faa > FAa < FAA) && ((Maa < MAa < MAA) || (Maa > MAa < MAA) ||
         (Maa > MAa > MAA)))} /. reverse /. pAveM → q pYm + (1 - q) pXm /. equilB0
    /. nohap /. Rm → 0 /. Rf → 0]]

(*Overdominance in females without overdominance in males*)
Simplify[Reduce[{stabcondB, (Faa FAa - 2 Faa FAA - 3 FAa FAA + 4 FAA^2)/(8 FAA^2) < 0,
     ((λmA1 > 1) || (λma1 > 1)), ((Faa < FAa > FAA) &&
       ((Maa < MAa < MAA) || (Maa > MAa < MAA) || (Maa > MAa > MAA)))} /. reverse /.
    pAveM → q pYm + (1 - q) pXm /. equilB0 /. nohap /. Rm → 0 /. Rf → 0]]

(*Directional selection in females without overdominance in males*)
Simplify[Reduce[{stabcondB, (Faa FAa - 2 Faa FAA - 3 FAa FAA + 4 FAA^2)/(8 FAA^2) < 0,
     ((λmA1 > 1) || (λma1 > 1)), (((Faa < FAa < FAA) || (Faa > FAa > FAA)) &&
       ((Maa < MAa < MAA) || (Maa > MAa < MAA) || (Maa > MAa > MAA)))} /. reverse /.
    pAveM → q pYm + (1 - q) pXm /. equilB0 /. nohap /. Rm → 0 /. Rf → 0]]
```

$$\text{FAA} > 0 \;\&\&\; 0 < \text{FAa} < \text{FAA} \;\&\&\; \text{Faa} > \frac{(3\,\text{FAa} - 4\,\text{FAA})\,\text{FAA}}{\text{FAa} - 2\,\text{FAA}} \;\&\&$$
$$\text{MAa} > 0 \;\&\&\; \text{Maa} > \text{MAa} \;\&\&\; \frac{2\,\text{FAA}\,\text{MAa}}{\text{FAa}} > \text{Maa} + \text{MAa} \;\&\&\; 0 < \text{MAA} < \text{MAa}$$

```
False
```

```
False
```

With overdominance in males, any form of selection in females can generate conditions allowing the spread of a neo-W:

```
(*Underdominance in females with overdominance in males*)Simplify[
 Reduce[{stabcondB, (Faa FAa - 2 Faa FAA - 3 FAa FAA + 4 FAA^2)/(8 FAA^2) < 0, ((λmA1 > 1) || (λma1 > 1)),
     ((Faa > FAa < FAA) && (Maa < MAa > MAA))} /. reverse /.
   pAveM → q pYm + (1 - q) pXm /. equilB0 /. nohap /. Rm → 0 /. Rf → 0]]

(*Overdominance in females with overdominance in males*)Simplify[
 Reduce[{stabcondB, (Faa FAa - 2 Faa FAA - 3 FAa FAA + 4 FAA^2)/(8 FAA^2) < 0, ((λmA1 > 1) || (λma1 > 1)),
     ((Faa < FAa > FAA) && (Maa < MAa > MAA))} /. reverse /.
   pAveM → q pYm + (1 - q) pXm /. equilB0 /. nohap /. Rm → 0 /. Rf → 0]]

(*Directional selection in females with overdominance in males*)Simplify[
 Reduce[{stabcondB, (Faa FAa - 2 Faa FAA - 3 FAa FAA + 4 FAA^2)/(8 FAA^2) < 0, ((λmA1 > 1) || (λma1 > 1)),
     (((Faa < FAa < FAA) || (Faa > FAa > FAA)) && (Maa < MAa > MAA))} /. reverse /.
   pAveM → q pYm + (1 - q) pXm /. equilB0 /. nohap /. Rm → 0 /. Rf → 0]]
```

$FAA > 0 \&\& FAa > 0 \&\& MAa > 0 \&\& Maa > 0 \&\& MAA > 0 \&\& FAa < FAA \&\&$
$Faa\,FAa + 4\,FAA^2 < 2\,Faa\,FAA + 3\,FAa\,FAA \&\& Maa < MAa \&\& MAA < MAa$

$FAA > 0 \&\& MAa > 0 \&\& Maa > 0 \&\& \frac{2\,FAA\,MAa}{FAa} > Maa + MAa \&\&$
$0 < MAA < MAa \&\& Faa < FAa \&\& \Big((0 < Faa \&\& FAa < 2\,FAA \&\& 4\,FAA \le 3\,FAa) \mid\mid$
$\Big(3\,FAa < 4\,FAA \&\& FAA < FAa \&\& \frac{(3\,FAa - 4\,FAA)\,FAA}{FAa - 2\,FAA} < Faa\Big)\Big)$

$FAA > 0 \&\& FAA < FAa < 2\,FAA \&\& Faa > FAa \&\&$
$MAa > 0 \&\& Maa > 0 \&\& \frac{2\,FAA\,MAa}{FAa} > Maa + MAa \&\& 0 < MAA < MAa$

Weak selection

(This section runs slow, so the inputs have been commented out and the main product pasted as “lambda2diffsExplicit”)

Solving for the eigenvalues with no selection gives

```
(*Normal[Series[charpolyExt/.weaksel/.q→1/2+q1*ϵ,{ϵ,0,0}]]//Factor;
Solve[%==0,λ]//Flatten*)
```

So on the zeroth order the leading eigenvalue is λ=1.

On the first order the eiganvalue is

```
(*Normal[Series[charpolyExt/.weaksel/.λ→1+δλ*ϵ/.frequenciessub/.sol0/.pXf0→pA,
    {ϵ,0,1}]]//Factor;
Solve[%==0,δλ];
Collect[(δλ/.%)/((1-pA) pA /VA),α1,Simplify]*)
```

which at equilibrium is zero (because at first order there is no Y bias in male gametes, q1=0)

```
(*%/.realsol[[3]]*)
```

On second order we have

```
(*Normal[
  Series[charpolyExt/.weaksel/.λ→1+δλ2*ϵ^2/.frequenciessub/.q1→0/.sol0/.pXf0→pA/.
     pXm1→dpAXmXf+pXf1/.pYm1→dpAYmXf+pXf1,{ϵ,0,2}]]//Factor;
Solve[%==0,δλ2];
lambda2diffs=Collect[δλ2/.%,{dpAXmXf,dpAYmXf},Simplify]*)
```

```
lambda2diffs =
```

$$\Big\{-\frac{1}{2}\,\text{dpAXmXf}\;k\;(2\,\text{hAf}\,(-1+k)\,(-1+2\,\text{pA})\,\text{sAf}-2\,\text{hAm}\,\text{sAm}+2\,\text{hAm}\,k\,\text{sAm}-2\,(-1+k)\,\text{pA}\,(\text{sAf}-\text{sAm}+2\,\text{hAm}\,\text{sAm})-k\,\text{tf}+k\,\text{tm})+\text{dpAYmXf}\Big(\text{hAf}\,k^2\,(1-2\,\text{pA})\,\text{sAf}+\frac{1}{2}\big(2\,\text{hAm}\,(-1+2\,\text{pA})\,\text{sAm}+k^2\,(-2\,\text{hAm}\,\text{sAm}+2\,\text{pA}\,(\text{sAf}-\text{sAm}+2\,\text{hAm}\,\text{sAm})+\text{tf}-\text{tm})-2\,(\text{pA}\,\text{sAm}+\text{tm})+4\,k\,(\text{pA}\,\text{sAm}+\text{hAm}\,(\text{sAm}-2\,\text{pA}\,\text{sAm})+\text{tm})\big)\Big)+\frac{1}{R}\Big(2\,(-1+2\,k)\,\text{q2}\,R-(-1+\text{pA})\,\text{pA}\,\Big(\text{hAf}^2\,k^2\,(1-2\,\text{pA})^2\,\text{sAf}^2+\text{hAm}^2\,\text{sAm}^2+2\,\text{hAm}\,\text{pA}\,\text{sAm}^2-4\,\text{hAm}^2\,\text{pA}\,\text{sAm}^2+\text{pA}^2\,\text{sAm}^2-4\,\text{hAm}\,\text{pA}^2\,\text{sAm}^2+4\,\text{hAm}^2\,\text{pA}^2\,\text{sAm}^2+\text{hAm}\,R\,\text{sAm}\,\text{tf}+\text{pA}\,R\,\text{sAm}\,\text{tf}-2\,\text{hAm}\,\text{pA}\,R\,\text{sAm}\,\text{tf}+2\,\text{hAm}\,\text{sAm}\,\text{tm}+2\,\text{pA}\,\text{sAm}\,\text{tm}-4\,\text{hAm}\,\text{pA}\,\text{sAm}\,\text{tm}-\text{hAm}\,R\,\text{sAm}\,\text{tm}-\text{pA}\,R\,\text{sAm}\,\text{tm}+2\,\text{hAm}\,\text{pA}\,R\,\text{sAm}\,\text{tm}+R\,\text{tf}\,\text{tm}+\text{tm}^2-R\,\text{tm}^2+4\,\text{hAm}\,\text{sAm}\,\alpha\text{1m}+4\,\text{pA}\,\text{sAm}\,\alpha\text{1m}-8\,\text{hAm}\,\text{pA}\,\text{sAm}\,\alpha\text{1m}-4\,\text{hAm}\,R\,\text{sAm}\,\alpha\text{1m}-4\,\text{pA}\,R\,\text{sAm}\,\alpha\text{1m}+8\,\text{hAm}\,\text{pA}\,R\,\text{sAm}\,\alpha\text{1m}+4\,\text{tm}\,\alpha\text{1m}-4\,R\,\text{tm}\,\alpha\text{1m}+4\,\alpha\text{1m}^2-8\,R\,\alpha\text{1m}^2+k^2\,\Big(\text{hAm}^2\,\text{sAm}^2+\text{pA}^2\,(\text{sAf}+(-1+2\,\text{hAm})\,\text{sAm})^2+\text{tf}^2-2\,R\,\text{tf}^2-2\,\text{tf}\,\text{tm}+4\,R\,\text{tf}\,\text{tm}+\text{tm}^2-2\,R\,\text{tm}^2+4\,\text{tf}\,\alpha\text{1f}-6\,R\,\text{tf}\,\alpha\text{1f}-4\,\text{tm}\,\alpha\text{1f}+6\,R\,\text{tm}\,\alpha\text{1f}+4\,\alpha\text{1f}^2-8\,R\,\alpha\text{1f}^2+\text{hAm}\,\text{sAm}\,((-2+3\,R)\,\text{tf}+(2-3\,R)\,\text{tm}+4\,(-1+R)\,(\alpha\text{1f}-\alpha\text{1m}))-4\,\text{tf}\,\alpha\text{1m}+6\,R\,\text{tf}\,\alpha\text{1m}+4\,\text{tm}\,\alpha\text{1m}-6\,R\,\text{tm}\,\alpha\text{1m}-8\,\alpha\text{1f}\,\alpha\text{1m}+16\,R\,\alpha\text{1f}\,\alpha\text{1m}+4\,\alpha\text{1m}^2-8\,R\,\alpha\text{1m}^2-\text{pA}\,(\text{sAf}+(-1+2\,\text{hAm})\,\text{sAm})\,(2\,\text{hAm}\,\text{sAm}+(-2+3\,R)\,\text{tf}+2\,\text{tm}-3\,R\,\text{tm}-4\,\alpha\text{1f}+4\,R\,\alpha\text{1f}+4\,\alpha\text{1m}-4\,R\,\alpha\text{1m})\Big)-\text{hAf}\,k\,(-1+2\,\text{pA})\,\text{sAf}\,(2\,(\text{pA}\,\text{sAm}+\text{hAm}\,(\text{sAm}-2\,\text{pA}\,\text{sAm})+R\,\text{tf}+\text{tm}-R\,\text{tm}+2\,\alpha\text{1m}-2\,R\,\alpha\text{1m})+k\,(-2\,\text{hAm}\,\text{sAm}+2\,\text{pA}\,(\text{sAf}-\text{sAm}+2\,\text{hAm}\,\text{sAm})+2\,\text{tf}-3\,R\,\text{tf}-2\,\text{tm}+3\,R\,\text{tm}+4\,\alpha\text{1f}-4\,R\,\alpha\text{1f}-4\,\alpha\text{1m}+4\,R\,\alpha\text{1m}))+k\,\Big(2\,\text{pA}^2\,(\text{sAf}-\text{sAm})\,\text{sAm}-2\,\text{hAm}^2\,(1-2\,\text{pA})^2\,\text{sAm}^2+R\,\text{tf}^2+2\,\text{tf}\,\text{tm}-4\,R\,\text{tf}\,\text{tm}-2\,\text{tm}^2+3\,R\,\text{tm}^2+2\,R\,\text{tf}\,\alpha\text{1f}+4\,\text{tm}\,\alpha\text{1f}-6\,R\,\text{tm}\,\alpha\text{1f}+4\,\text{tf}\,\alpha\text{1m}-6\,R\,\text{tf}\,\alpha\text{1m}-8\,\text{tm}\,\alpha\text{1m}+10\,R\,\text{tm}\,\alpha\text{1m}+8\,\alpha\text{1f}\,\alpha\text{1m}-16\,R\,\alpha\text{1f}\,\alpha\text{1m}-8\,\alpha\text{1m}^2+16\,R\,\alpha\text{1m}^2+2\,\text{pA}\,(R\,(\text{sAf}\,(\text{tf}-\text{tm}-2\,\alpha\text{1m})-2\,\text{sAm}\,(\text{tf}-\text{tm}+\alpha\text{1f}-2\,\alpha\text{1m}))+\text{sAm}\,(\text{tf}-2\,\text{tm}+2\,\alpha\text{1f}-4\,\alpha\text{1m})+\text{sAf}\,(\text{tm}+2\,\alpha\text{1m}))-2\,\text{hAm}\,(-1+2\,\text{pA})\,\text{sAm}\,(\text{pA}\,(\text{sAf}-2\,\text{sAm})+\text{tf}-2\,R\,\text{tf}+2\,(-1+R)\,(\text{tm}-\alpha\text{1f}+2\,\alpha\text{1m}))\Big)\Big)\Big)\Big\};$$

Then we can put in all the differences

```
lambda2diffsExplicit = lambda2diffs /. realsol[[3]] /. freqYm2sol // Simplify
```

```
{- 1/(2 r R (sAf - 2 hAf sAf + sAm - 2 hAm sAm)^4)
   (hAf^3 sAf^3 (sAm + 2 tm + 4 α1m) - hAf^2 sAf^2 (- (- 1 + 2 hAm) sAm
          (sAm - tf + 2 tm - 2 α1f + 4 α1m) + sAf (sAm + hAm sAm + 3 tm + 6 α1m)) -
      hAf sAf (2 sAm^2 tf + sAm tf^2 + sAm^2 tm + 4 sAm tf tm + 2 tf^2 tm + 3 sAm tm^2 +
         4 tf tm^2 + 2 tm^3 + 4 sAm^2 α1f + 4 sAm tf α1f + 8 sAm tm α1f + 8 tf tm α1f + 8 tm^2 α1f +
         4 sAm α1f^2 + 8 tm α1f^2 + 2 sAm^2 α1m + 8 sAm tf α1m + 4 tf^2 α1m + 12 sAm tm α1m +
         16 tf tm α1m + 12 tm^2 α1m + 16 sAm α1f α1m + 16 tf α1f α1m + 32 tm α1f α1m +
         16 α1f^2 α1m + 12 sAm α1m^2 + 16 tf α1m^2 + 24 tm α1m^2 + 32 α1f α1m^2 + 16 α1m^3 -
         sAf^2 (tm + 2 α1m) - hAm^2 sAm^2 (- 2 sAf + sAm - 4 tf + 2 tm - 8 α1f + 4 α1m) + hAm sAm
          (- sAf^2 - 2 sAf (tf - 2 tm + 2 α1f - 4 α1m) + sAm (sAm - 4 tf + 2 tm - 8 α1f + 4 α1m)) +
         2 sAf (sAm (tf + 2 α1f) + (tm + 2 α1m) (tf + tm + 2 (α1f + α1m)))) -
      (hAm sAm + tf + tm + 2 α1f + 2 α1m) (hAm^2 sAm^2 (sAf + 2 tf + 4 α1f) -
         sAf^2 (tm + 2 α1m) + sAm (tf + 2 α1f) (sAm + tf + tm + 2 α1f + 2 α1m) +
         sAf (sAm (tf - tm + 2 α1f - 2 α1m) - (tm + 2 α1m) (tf + tm + 2 (α1f + α1m))) -
         hAm sAm (sAf^2 + sAf (sAm + 3 tf + 6 α1f) +
            (tf + 2 α1f) (3 sAm + 2 (tf + tm + 2 (α1f + α1m))))))
   (- k^2 R sAf tf + 2 k^2 r R sAf tf - 2 r sAm tf + 8 k r sAm tf - 8 k^2 r sAm tf +
      2 R sAm tf - 4 k R sAm tf + 3 k^2 R sAm tf - 8 k r R sAm tf +
      10 k^2 r R sAm tf + 2 r sAf tm - 8 k r sAf tm + 8 k^2 r sAf tm -
      2 R sAf tm + 4 k R sAf tm - 3 k^2 R sAf tm + 8 k r R sAf tm -
      10 k^2 r R sAf tm + k^2 R sAm tm - 2 k^2 r R sAm tm - 4 k^2 R sAf α1f +
      8 k^2 r R sAf α1f - 4 r sAm α1f + 16 k r sAm α1f - 16 k^2 r sAm α1f +
      4 R sAm α1f - 8 k R sAm α1f + 4 k^2 R sAm α1f - 16 k r R sAm α1f +
      24 k^2 r R sAm α1f + 4 r sAf α1m - 16 k r sAf α1m + 16 k^2 r sAf α1m -
      4 R sAf α1m + 8 k R sAf α1m - 4 k^2 R sAf α1m + 16 k r R sAf α1m -
      24 k^2 r R sAf α1m + 4 k^2 R sAm α1m - 8 k^2 r R sAm α1m +
      2 hAf sAf (R ((1 - 2 k + 2 k^2) sAm + 2 (tm + 2 α1m) -
            4 k (tm + 2 α1m) + k^2 (tf + 3 tm + 4 (α1f + α1m))) +
         r ((- 1 - 4 k (- 1 + R) + 4 k^2 (- 1 + R)) sAm - 2 (tm + 2 α1m + 4 k (- 1 + R) (tm + 2 α1m) +
               k^2 (R (tf - 5 tm + 4 α1f - 12 α1m) + 4 (tm + 2 α1m))))) - 2 hAm sAm
       (R ((1 - 2 k + 2 k^2) sAf + (2 - 4 k + 3 k^2) tf + k^2 tm + 4 α1f - 8 k α1f + 4 k^2 α1f + 4 k^2 α1m) +
         r ((- 1 - 4 k (- 1 + R) + 4 k^2 (- 1 + R)) sAf + 2 ((- 1 - 4 k (- 1 + R) + k^2 (- 4 + 5 R))
                tf - 2 α1f - 8 k (- 1 + R) α1f - k^2 (8 α1f + R (tm - 12 α1f + 4 α1m))))))}
```

For any k we can write this as

```
(1 - 2 k)^2/4 VA SA^2 (r - R)/(r R) + (k dpAYmXm)/2 (k (2 αDm - 2 αDf + tm - tf) - 2 (1 - k) SA) /.
   SA → (Dm + αDm + tm) - (Df + αDf + tf) /. trysub3 /. realsol2[[3]];
{0} == % - lambda2diffsExplicit // Factor
```

```
True
```

For a neo-Y this reduces to

```
1/4 VA SA^2 (r - R)/(r R) /. SA → (Dm + αDm + tm) - (Df + αDf + tf) /. trysub3 /. realsol2[[3]];
{0} == % - lambda2diffsExplicit /. k → 0 // Factor
```

True

And for a neo-W this reduces to

```
1/4 VA SA^2 (r - R)/(r R) + dpAYmXm/2 (2 αDm - 2 αDf + tm - tf) /. SA → (Dm + αDm + tm) - (Df + αDf + tf) /.
   trysub3 /. realsol2[[3]];
{0} == % - lambda2diffsExplicit /. k → 1 // Factor
```

True

When r=1/2 the neo-W case reduces to the neo-Y case as

```
dpAYmXm /. realsol2[[3]] /. r → 1 / 2
```

0

Now consider only R=1/2 and r<1/2 (a new sex chromosome arises).

When the only haploid selection is male drive a neo-W can invade only when the following is positive

```
1/4 VA SA^2 (r - R)/(r R) + dpAYmXm/2 (2 αDm - 2 αDf + tm - tf) /. SA → (Dm + αDm + tm) - (Df + αDf + tf) /.
        trysub3 /. realsol2[[3]] /. hAf → hAm /. hAm → hA /.
     R → 1 / 2 /. tf → 0 /. tm → 0 /. α1f → 0 // Simplify
```

$$-\frac{4\,(-1+2\,r)\,\text{sAf}\,\text{sAm}\,((-1+\text{hA})\,\text{sAf}+(-1+\text{hA})\,\text{sAm}-2\,\alpha 1\text{m})\,\alpha 1\text{m}^2\,(\text{hA}\,(\text{sAf}+\text{sAm})+2\,\alpha 1\text{m})}{(1-2\,\text{hA})^2\,r\,(\text{sAf}+\text{sAm})^4}$$

The denominator is always positive. The two selection terms in parentheses in the numerator are positive when there is a stable polymorphism

```
stabconds /. hAf → hAm /. hAm → hA /. R → 1 / 2 /. tf → 0 /. tm → 0 /. α1f → 0 // Simplify
```

$\{(-1+\text{hA})\,(\text{sAf}+\text{sAm}) > 2\,\alpha 1\text{m},\ \text{hA}\,(\text{sAf}+\text{sAm})+2\,\alpha 1\text{m} > 0\}$

Thus we need sAf sAm > 0 for a neo-W to invade when the only haploid selection is male drive.

When the only haploid selection is female drive a neo-W can invade only when the following is positive

```
1/4 VA SA^2 (r - R)/(r R) + dpAYmXm/2 (2 αDm - 2 αDf + tm - tf) /. SA → (Dm + αDm + tm) - (Df + αDf + tf) /.
        trysub3 /. realsol2[[3]] /. hAf → hAm /. hAm → hA /.
     R → 1 / 2 /. tf → 0 /. tm → 0 /. α1m → 0 // Simplify
```

$$-\frac{4\,(-1+2\,r)\,\text{sAf}\,\text{sAm}\,((-1+\text{hA})\,\text{sAf}+(-1+\text{hA})\,\text{sAm}-2\,\alpha 1\text{f})\,\alpha 1\text{f}^2\,(\text{hA}\,(\text{sAf}+\text{sAm})+2\,\alpha 1\text{f})}{(1-2\,\text{hA})^2\,r\,(\text{sAf}+\text{sAm})^4}$$

The denominator is always positive. The two selection terms in parentheses in the numerator are positive when there is a stable polymorphism

```
stabconds /. hAf → hAm /. hAm → hA /. R → 1 / 2 /. tf → 0 /. tm → 0 /. α1m → 0 // Simplify
```

$\{(-1+\text{hA})\,(\text{sAf}+\text{sAm}) > 2\,\alpha 1\text{f},\ \text{hA}\,(\text{sAf}+\text{sAm})+2\,\alpha 1\text{f} > 0\}$

Thus we need sAf sAm > 0 for a neo-W to invade when the only haploid selection is female drive.

When the only haploid selection is male gamete competition a neo-W can invade only when the following is positive

```
1/4 VA SA^2 (r - R)/(r R) + dpAYmXm/2 (2 αDm - 2 αDf + tm - tf) /. SA → (Dm + αDm + tm) - (Df + αDf + tf) /.
        trysub3 /. realsol2[[3]] /. hAf → hAm /.
    hAm → hA /. tf → 0 /. α1m → 0 /. α1f → 0 // Simplify
```

$$-\left(\text{sAf}\,(\text{R}\,(\text{sAf}-\text{sAm})+2\,\text{r}\,((-1+\text{R})\,\text{sAf}+\text{R}\,\text{sAm}))\,((-1+\text{hA})\,\text{sAf}+(-1+\text{hA})\,\text{sAm}-\text{tm})\,\text{tm}^2\,(\text{hA}\,(\text{sAf}+\text{sAm})+\text{tm})\right)\Big/\left(2\,(1-2\,\text{hA})^2\,\text{r}\,\text{R}\,(\text{sAf}+\text{sAm})^4\right)$$

The denominator is always positive. The two selection terms in parentheses in the numerator are positive when there is a stable polymorphism

```
stabconds /. hAf → hAm /. hAm → hA /. R → 1 / 2 /. tf → 0 /. α1m → 0 /. α1f → 0 // Simplify
```

$$\{(-1+\text{hA})\,(\text{sAf}+\text{sAm})>\text{tm},\ \text{hA}\,(\text{sAf}+\text{sAm})+\text{tm}>0\}$$

Thus we need sAf (sAf - sAm) < 0 for a neo-W to invade when the only haploid selection is male gamete competition.

When the only haploid selection is female gamete competition a neo-W can invade only when the following is positive

```
1/4 VA SA^2 (r - R)/(r R) + dpAYmXm/2 (2 αDm - 2 αDf + tm - tf) /. SA → (Dm + αDm + tm) - (Df + αDf + tf) /.
        trysub3 /. realsol2[[3]] /. hAf → hAm /. hAm → hA /.
    R → 1 / 2 /. tm → 0 /. α1m → 0 /. α1f → 0 // Simplify
```

$$-\frac{(-1+2\,\text{r})\,(\text{sAf}-\text{sAm})\,\text{sAm}\,((-1+\text{hA})\,\text{sAf}+(-1+\text{hA})\,\text{sAm}-\text{tf})\,\text{tf}^2\,(\text{hA}\,(\text{sAf}+\text{sAm})+\text{tf})}{2\,(1-2\,\text{hA})^2\,\text{r}\,(\text{sAf}+\text{sAm})^4}$$

The denominator is always positive. The two selection terms in parentheses in the numerator are positive when there is a stable polymorphism

```
stabconds /. hAf → hAm /. hAm → hA /. R → 1 / 2 /. tm → 0 /. α1m → 0 /. α1f → 0 // Simplify
```

$$\{(-1+\text{hA})\,(\text{sAf}+\text{sAm})>\text{tf},\ \text{hA}\,(\text{sAf}+\text{sAm})+\text{tf}>0\}$$

Thus we need (sAf-sAm) sAm > 0 for a neo-W to invade when the only haploid selection is male gamete competition.

Notice that the invasion fitness of a neo-W into a XY system is the same as a neo-Y into a ZW system (at least up to order ϵ^2)

```
1/4 VA SA^2 (r - R)/(r R) + dpAYmXm/2 (2 αDm - 2 αDf + tm - tf) /. SA → (Dm + αDm + tm) - (Df + αDf + tf) /.
   trysub3 /. realsol2[[3]];
1/4 VA SA^2 (r - R)/(r R) + dpAYmXm/2 (2 αDm - 2 αDf + tm - tf) /. SA → (Dm + αDm + tm) - (Df + αDf + tf) /.
                 trysub3 /. realsol2[[3]] /. sAf → temp /. sAm → sAf /. temp → sAm /.
           hAf → temp /. hAm → hAf /. temp → hAm /. tf → temp /. tm → tf /.
      temp → tm /. α1m → temp /. α1f → α1m /. temp → α1f;
% ==
  %% //
 Simplify
```

```
True
```

Finally, note that the k=1/2 invasion fitness can be written as a function of the neo-Y and neo-W invasion fitnesses evaluated at R=1/2

```
1/2 (1/2 (((1/4 VA SA^2 (r - R)/(r R) /. R → 1/2) +
          (1/4 VA SA^2 (r - R)/(r R) + dpAYmXm/2 (2 αDm - 2 αDf + tm - tf) /. R → 1/2)))) /.
     SA → (Dm + αDm + tm) - (Df + αDf + tf) /. trysub3 /. realsol2[[3]];
{0} == % - lambda2diffsExplicit /. k → 1/2 // Simplify
```

```
True
```

and this is the same regardless of whether the ancestral SD system is XY or ZW

```
1/2 (1/2 (((1/4 VA SA^2 (r - R)/(r R) /. R → 1/2) +
          (1/4 VA SA^2 (r - R)/(r R) + dpAYmXm/2 (2 αDm - 2 αDf + tm - tf) /. R → 1/2)))) /.
     SA → (Dm + αDm + tm) - (Df + αDf + tf) /. trysub3 /. realsol2[[3]];
1/2 (1/2 (((1/4 VA SA^2 (r - R)/(r R) /. R → 1/2) + (1/4 VA SA^2 (r - R)/(r R) + dpAYmXm/2 (2 αDm - 2 αDf + tm - tf) /.
                         R → 1/2))) /. SA → (Dm + αDm + tm) - (Df + αDf + tf) /. trysub3 /.
               realsol2[[3]] /. sAf → temp /. sAm → sAf /. temp → sAm /.
           hAf → temp /. hAm → hAf /. temp → hAm /. tf → temp /. tm → tf /.
      temp → tm /. α1m → temp /. α1f → α1m /. temp → α1f;
% ==
  %% //
 Simplify
```

```
True
```

Figures

Plotting parameters

Where to save plots

```
SetDirectory[NotebookDirectory[]];
(*sets current directory to be location of this file*)
plotdir = "Plots/";(*directory to save figures in*)
```

Remove lines from region plots when saving

```
rasterTrick[plot_] := Show[plot,
  Prolog → {Opacity[0], Texture[{{{0, 0, 0, 0}}}], VertexTextureCoordinates →
     {{0, 0}, {1, 0}, {1, 1}}, Polygon[{{0, 0}, {.1, 0}, {.1, .1}}]}]
```

Universal aesthetics

```
lwd = 0.006;(*line thickness as fraction of plot width*)
letpos = {0.05, 0.95}; (*relative location of panel letter*)
ylabpos = {-0.16, 0.5}; (*relative location of y axis label*)
ylabposregion = {-0.1, 0.5};(*for region plots*)
inches = 72;
xsize = 5 inches; (*width of panel*)
aspectratio = 1 / Sqrt[2]; (*aspect ratio of panel*)
ticksize = {0, 0.01}; (*size of tick marks*)
pad = {{50, 20}, {40, 20}}; (*white space around plot {{left,right},{bottom,top}}*)
padregion = {{40, 10}, {40, 30}};
(*white space around region plot {{left,right},{bottom,top}}*)
posplotscale = 1;
```

Basic plot style (λ vs cM plots)

```
Clear[plotstyle]
plotstyle[xmin_, xmax_, xtickmin_, xtickmax_,
  xint_, ymin_, ymax_, ytickmin_, ytickmax_, yint_] := {
  PlotRange → {{xmin, xmax}, {ymin, ymax}},
  ImageSize → {xsize * posplotscale, xsize aspectratio * posplotscale},
  AspectRatio → aspectratio,
  PlotRangePadding → 0,
  FrameTicks → {Table[{x, x, ticksize}, {x, xtickmin, xtickmax, xint}],
    Table[{y, y, ticksize}, {y, ytickmin, ytickmax, yint}]},
  FrameTicksStyle → {{Directive[Black, Thickness[lwd]],
     Directive[Black, Thickness[lwd]]},
    {Directive[Black, Thickness[lwd]], Directive[Black, Thickness[lwd]]}},
  FrameStyle → {{Black, Thickness[lwd]}, {Black, Thickness[lwd]}, None, None},
  BaseStyle → {FontFamily → "Helvetica", FontSize → 14 * posplotscale},
  ImagePadding → pad,
  PlotRangeClipping → False
 }
```

Log-linear plot style (λ vs R plots)

```
Clear[loglinearplotstyle]
loglinearplotstyle[xmin_, xmax_, xmarks_,
  ymin_, ymax_, ytickmin_, ytickmax_, yint_, recrate_] := {
  PlotRange → {Log@{xmin, xmax}, {ymin, ymax}},
  ImageSize → {xsize * posplotscale, xsize aspectratio * posplotscale},
  AspectRatio → aspectratio,
  PlotRangePadding → 0,
  Frame → {{True, False}, {True, False}},
  FrameTicks → {{Table[{y, y, ticksize}, {y, ytickmin, ytickmax, yint}], None},
    {Append[Table[{Log@x, x, ticksize}, {x, xmarks}],
      {Log@recrate, "r", ticksize}], None}},
  FrameTicksStyle → {{Directive[Black, Thickness[lwd], FontColor → Black],
     Directive[Black, Thickness[lwd]]},
    {Directive[Black, Thickness[lwd]], Directive[Black, Thickness[lwd]]}},
  FrameStyle -> Directive[Black, Thickness[lwd]],
  BaseStyle → {FontFamily → "Helvetica", FontSize → 14 * posplotscale},
  ImagePadding → pad
 }
```

Log-linear inset plot style (frequency dynamics)

```
Clear[insetloglinearplotstyle]
insetloglinearplotstyle[xmin_, xmax_,
  xmarks_, ymin_, ymax_, ytickmin_, ytickmax_, yint_] := {
  PlotRange → {Log@{xmin, xmax}, {ymin, ymax}},
  ImageSize → {xsize, xsize aspectratio },
  AspectRatio → aspectratio,
  PlotRangePadding → 0,
  Frame → {{True, False}, {True, False}},
  FrameTicks → {{Table[{y, y, ticksize}, {y, ytickmin, ytickmax, yint}], None},
    {Table[{Log@x, x, ticksize}, {x, xmarks}], None}},
  FrameTicksStyle → {{Directive[Black, Thickness[lwd], FontColor → Black],
     Directive[Black, Thickness[lwd]]},
    {Directive[Black, Thickness[lwd]], Directive[Black, Thickness[lwd]]}},
  FrameStyle -> Directive[Black, Thickness[lwd]],
  BaseStyle → {FontFamily → "Helvetica", FontSize → 8 * 4 / 5},
  ImagePadding → pad
 }
```

Region plot style (where neo-W invades)

```
Clear[regionplotstyle]
regionplotstyle[xmin_, xmax_, xtickmin_,
  xtickmax_, xint_, ymin_, ymax_, ytickmin_, ytickmax_, yint_] := {
  PlotRange → {{xmin, xmax}, {ymin, ymax}},
  ImageSize → {xsize, xsize},
  PlotRangePadding → 0,
  FrameTicks → {Table[{x, x, ticksize}, {x, xtickmin, xtickmax, xint}],
    Table[{y, y, ticksize}, {y, ytickmin, ytickmax, yint}], None, None},
  FrameTicksStyle → {{Directive[Black, Thickness[lwd]],
     Directive[Black, Thickness[lwd]]}, {Directive[Black, Thickness[lwd]],
     Directive[Black, Thickness[lwd], FontColor → White]}},
  FrameStyle → {{Black, Thickness[lwd]}, {Black, Thickness[lwd]},
    {Black, Thickness[lwd]}, {Black, Thickness[lwd]}},
  BaseStyle → {FontFamily → "Helvetica", FontSize → 14},
  ImagePadding → padregion,
  PlotRangeClipping → False
 }
```

Useful functions

Haldane’s mapping function

Haldane’s (1919) mapping function: probability of an odd number of crossovers between positions y and z (for y and z sufficiently far apart; Eq 3), where y and z are measured in centiMorgans

```
pcross[y_, z_] := (1 - Exp[-2 * Sqrt[(y - z)^2] / 100]) / 2 // N
```

$$\text{pcross}[y\_, z\_] := \frac{1 - \text{Exp}\left[-2 * \sqrt{(y-z)^2} \middle/ 100\right]}{2} \text{ // N}$$

Recombination probabilities (no interference)

```
setr[x_, a_, m_] :=
 If[
  x < m < a || a < m < x,
  pcross[x, m] (1 - pcross[m, a]) + (1 - pcross[x, m]) pcross[m, a],
  pcross[x, a]
 ]

setR[x_, a_, m_] :=
 If[
  a < x < m || m < x < a,
  pcross[a, x] (1 - pcross[x, m]) + (1 - pcross[a, x]) pcross[x, m],
  pcross[a, m]
 ]

setρ[x_, a_, m_] :=
 If[
  x < a < m || m < a < x,
  pcross[x, a] (1 - pcross[a, m]) + (1 - pcross[x, a]) pcross[a, m],
  pcross[x, m]
 ]
```

XY resident equilibrium and stability

Resident equilibria

```
(*differenceEqs//Simplify*)
```

```
Clear[equilXY]
equilXY[FAA_, FAa_, Faa_, MAA_, MAa_, Maa_, wAf_, waf_, wAm_, wam_, αf_, αm_, r_] :=
 equilXY[FAA, FAa, Faa, MAA, MAa, Maa, wAf, waf, wAm, wam, αf, αm, r] =
  {pXf, pXm, pYm, q} /.
   NSolve[{(Faa pXf (-1 + pXf + pXm - pXf pXm) waf wam - FAA (-1 + pXf) pXf pXm wAf wAm +
          FAa (-pXm waf wAm + pXf ((-1 + pXm) wAf wam + pXm waf wAm)) (pXf - αf)) /
        (Faa (-1 + pXf) (-1 + pXm) waf wam + FAA pXf pXm wAf wAm +
          FAa (pXm waf wAm - pXf ((-1 + pXm) wAf wam + pXm waf wAm))), (2 Maa (-1 + pXf)
           pXm (-1 + pYm) (-1 + q) waf wam + MAA pXf pYm (1 + 2 pXm (-1 + q)) wAf wAm -
          2 MAa (pYm waf wAm (pXm - pXm q - r αm) + pXf (pXm (-1 + q) ((-1 + pYm) wAf wam +
                  pYm waf wAm) + ((-1 + pYm + r - pYm r) wAf wam + pYm r waf wAm) αm))) /
        (2 (Maa (-1 + pXf) (-1 + pYm) waf wam + MAA pXf pYm wAf wAm +
             MAa (pYm waf wAm - pXf ((-1 + pYm) wAf wam + pYm waf wAm)))),
       (-2 Maa (-1 + pXf) (-1 + pYm) pYm q waf wam + MAA pXf pYm (1 - 2 pYm q) wAf wAm +
          2 MAa (pYm waf wAm (-pYm q + αm - r αm) + pXf (pYm^2 q (wAf wam + waf wAm) + r wAf
                  wam αm - pYm (q wAf wam + r wAf wam αm + waf wAm αm - r waf wAm αm)))) /
        (2 (Maa (-1 + pXf) (-1 + pYm) waf wam + MAA pXf pYm wAf wAm +
             MAa (pYm waf wAm - pXf ((-1 + pYm) wAf wam + pYm waf wAm)))),
       (-Maa (-1 + pXf) (-1 + pYm) (-1 + 2 q) waf wam + MAA pXf pYm (1 - 2 q) wAf wAm +
          2 MAa (pYm waf wAm (-q + r + αm - 2 r αm) + pXf ((-1 + pYm) wAf wam
                 (-1 + q + r + αm - 2 r αm) + pYm waf wAm (q - αm + r (-1 + 2 αm))))) /
        (2 (Maa (-1 + pXf) (-1 + pYm) waf wam + MAA pXf pYm wAf wAm + MAa (pYm waf wAm - pXf
                ((-1 + pYm) wAf wam + pYm waf wAm))))} == {0, 0, 0, 0}, {pXf, pXm, pYm, q}]
```

Eigenvalues of internal stability matrix

```
(*matIntFull//Simplify*)
```

```
Clear[internstabXY];
internstabXY[FAA_, FAa_, Faa_, MAA_, MAa_, Maa_,
  wAf_, waf_, wAm_, wam_, αf_, αm_, r_, pXf_, pXm_, pYm_, q_] :=
 internstabXY[FAA, FAa, Faa, MAA, MAa, Maa, wAf, waf, wAm, wam, αf, αm, r,
   pXf, pXm, pYm, q] = Eigenvalues[{{(waf wAf (-FAa FAA pXm^2 wAm^2 (-1 + αf) -
          Faa (-1 + pXm) wam (FAA pXm wAm - FAa (-1 + pXm) wam αf))) /
      (Faa (-1 + pXf) (-1 + pXm) waf wam + FAA pXf pXm wAf wAm +
         FAa (pXm waf wAm - pXf ((-1 + pXm) wAf wam + pXm waf wAm)))^2, (wam wAm (-FAa FAA
           pXf^2 wAf^2 (-1 + αf) - Faa (-1 + pXf) waf (FAA pXf wAf - FAa (-1 + pXf) waf αf))) /
      (Faa (-1 + pXf) (-1 + pXm) waf wam + FAA pXf pXm wAf wAm +
         FAa (pXm waf wAm - pXf ((-1 + pXm) wAf wam + pXm waf wAm)))^2, 0}, {- (waf wAf
         (Maa (-1 + pYm) wam (MAA pYm wAm + 2 MAa (-1 + pYm) (-1 + r) wam αm) + 2 MAa pYm
            wAm (-1 + αm) (-MAA pYm (-1 + r) wAm + 2 MAa (-1 + pYm) (-1 + 2 r) wam αm))) /
      (Maa (-1 + pXf) (-1 + pYm) waf wam + MAA pXf pYm wAf wAm + 2 MAa
           (pYm waf wAm (1 - αm + r (-1 + 2 αm)) + pXf (wAf wam αm + r ((-1 + pYm) wAf wam -
                   pYm waf wAm) (-1 + 2 αm) + pYm (waf wAm (-1 + αm) - wAf wam αm))))^2,
     0, (wam wAm (-Maa (-1 + pXf) waf (MAA pXf wAf - 2 MAa (-1 + pXf) r waf αm) +
          2 MAa pXf wAf (-1 + αm) (-MAA pXf r wAf + 2 MAa (-1 + pXf) (-1 + 2 r) waf αm))) /
      (Maa (-1 + pXf) (-1 + pYm) waf wam + MAA pXf pYm wAf wAm + 2 MAa
           (pYm waf wAm (1 - αm + r (-1 + 2 αm)) + pXf (wAf wam αm + r ((-1 + pYm) wAf wam -
                   pYm waf wAm) (-1 + 2 αm) + pYm (waf wAm (-1 + αm) - wAf wam αm))))^2},
    {(waf wAf (-Maa (-1 + pYm) wam (MAA pYm wAm - 2 MAa (-1 + pYm) r wam αm) +
          2 MAa pYm wAm (-1 + αm) (-MAA pYm r wAm + 2 MAa (-1 + pYm) (-1 + 2 r) wam αm))) /
      (Maa (-1 + pXf) (-1 + pYm) waf wam + MAA pXf pYm wAf wAm -
         2 MAa (pYm waf wAm (-αm + r (-1 + 2 αm)) +
              pXf (pYm waf wAm (r + αm - 2 r αm) + (-1 + pYm) wAf wam (1 - r - αm + 2 r αm))))^2,
     0, - (wam wAm (Maa (-1 + pXf) waf (MAA pXf wAf + 2 MAa (-1 + pXf) (-1 + r) waf αm) +
            2 MAa pXf wAf (-1 + αm)
              (-MAA pXf (-1 + r) wAf + 2 MAa (-1 + pXf) (-1 + 2 r) waf αm))) /
      (Maa (-1 + pXf) (-1 + pYm) waf wam + MAA pXf pYm wAf wAm -
         2 MAa (pYm waf wAm (-αm + r (-1 + 2 αm)) + pXf
               (pYm waf wAm (r + αm - 2 r αm) + (-1 + pYm) wAf wam (1 - r - αm + 2 r αm))))^2}}]
```

The following uses this cutoff to avoid numerical problems

```
cutoff = 10^(-12);
```

Restricting answers to cases of polymorphic equilibria that are internally stable

```
Clear[sieveXY]
sieveXY[FAA_, FAa_, Faa_, MAA_, MAa_, Maa_, wAf_, waf_, wAm_, wam_, αf_, αm_, r_] :=
 sieveXY[FAA, FAa, Faa, MAA, MAa, Maa, wAf, waf, wAm, wam, αf, αm, r] =
  Block[{}, For[i = 1; write = {}, i ≤ (max = Length[eq =
         equilXY[FAA, FAa, Faa, MAA, MAa, Maa, wAf, waf, wAm, wam, αf, αm, r]]), i++,
    If[Length[test = Cases[eq[[i]], x_ /; ((-cutoff ≤ Re[x] ≤ 1 + cutoff) &&
             Abs[Im[x]] < cutoff)]] == 4 &&
      (Chop[eq[[i]]] ≠ {0, 0, 0,}) && (Chop[eq[[i]] - {1, 1, 1, 1}] ≠ {0, 0, 0,}) &&
      (Max[Abs[internstabXY[FAA, FAa, Faa, MAA, MAa, Maa, wAf, waf, wAm,
           wam, αf, αm, r, eq[[i, 1]], eq[[i, 2]], eq[[i, 3]], eq[[i, 4]]]]] <
        1), write = Append[write, eq[[i]]]]];
   Sort[write]
  ]
```

Invasion of neo-W into XY

Invasion fitness (external stability)

```
(*charpolyk1//Simplify*)
```

```
Clear[invasionplotXY]
invasionplotXY[tryFAA_, tryFAa_, tryFaa_, tryMAA_, tryMAa_, tryMaa_,
  trywAf_, trywaf_, trywAm_, trywam_, tryαf_, tryαm_, tryr_, tryR_, tryρ_] :=
 Block[{valideq, temp, pXf, pXm, pYf, pYm, q, subs, r, R, ρ},
  valideq = sieveXY[tryFAA, tryFAa, tryFaa, tryMAA, tryMAa,
    tryMaa, trywAf, trywaf, trywAm, trywam, tryαf, tryαm, tryr];
  If[Length[valideq] == 1,
   temp = Flatten[valideq];
   pXf = temp[[1]];
   pXm = temp[[2]];
   pYf = 0;
   pYm = temp[[3]];
   q = temp[[4]];
   subs =
    {FAA → tryFAA, FAa → tryFAa, Faa → tryFaa, MAA → tryMAA, MAa → tryMAa, Maa → tryMaa,
     wAf → trywAf, waf → trywaf, wAm → trywAm, wam → trywam, αf → tryαf, αm → tryαm};
   r = tryr;
   R = tryR;
   ρ = tryρ;
   Max[
    λ /. NSolve[0 == (λ^4 (2 Faa^2 (-1 + pXf) (-1 + pXm) (-1 + q) waf^2 wam^2 λ (1 - pYm q - 2 λ +
              2 pXf λ + 2 q λ - 2 pXf q λ + pXm (-1 + q) (1 + 2 (-1 + pXf) λ)) -
           Faa waf wam (FAA wAf wAm (pYm q (-1 + pYm q + 2 (-1 + pXf) (-1 + q) λ) - pXm^2
                 (-1 + q)^2 (-1 + 2 λ + 8 (-1 + pXf) pXf λ^2) + pXm (-1 + q) (1 + 2 pYm q
                   (-1 + λ) - 2 (1 + (-1 + pXf) q) λ + 8 (-1 + pXf) pXf (-1 + q) λ^2)) +
              2 FAa (wAf wam ((1 + pXm (-1 + q) - pYm q) (-1 + R) αf (1 - pYm q - 2 λ +
                      2 pXf λ + 2 q λ - 2 pXf q λ + pXm (-1 + q) (1 + 2 (-1 + pXf) λ)) +
                   pXf (-1 + pXm) (-1 + q) λ (1 - pYm q - 4 λ + 4 pXf λ +
                      4 q λ - 4 pXf q λ + pXm (-1 + q) (1 + 4 (-1 + pXf) λ))) +
                 (-1 + pXf) (-1 + q) waf wAm λ (2 pYm q (-1 + R) (-1 + αf) +
                   pXm^2 (-1 + q) (3 + 2 R (-1 + αf) - 2 αf - 4 λ + 4 pXf λ) +
                   pXm (3 + 2 R (-1 + αf) - 2 αf - 4 λ + 4 pXf λ + q (pYm (-3 + 2 R + 2 αf -
```

$$
\begin{aligned}
&2\,R\,\alpha f) + 2\,(-1 + R + \alpha f - R\,\alpha f + 2\,\lambda - 2\,pXf\,\lambda)))))) + \\
&2\,(FAA^2\,pXf\,pXm\,(-1 + q)\,wAf^2\,wAm^2\,\lambda\,(pYm\,q + pXm\,(-1 + q)\,(-1 + 2\,pXf\,\lambda)) - \\
&FAa\,FAA\,wAf\,wAm\,(pYm\,q\,(pYm\,q\,waf\,wAm\,(-1 + R + \alpha f - R\,\alpha f) - pXf\,(-1 + q) \\
&wAf\,wam\,\lambda) + pXm^2\,(-1 + q)^2\,(pXf\,wAf\,wam\,\lambda\,(-1 + 2\,(-1 + R)\,\alpha f + \\
&4\,pXf\,\lambda) + waf\,wAm\,(-1 + \alpha f + \lambda + pXf\,\lambda - 2\,pXf\,\alpha f\,\lambda - 4\,pXf\,\lambda^2 + \\
&4\,pXf^2\,\lambda^2 + R\,(-1 + \alpha f)\,(-1 + 2\,pXf\,\lambda))) - pXm\,(-1 + q) \\
&(pXf\,wAf\,wam\,\lambda\,(1 + 2\,\alpha f - 2\,R\,\alpha f - 4\,pXf\,\lambda + q\,(-1 + 4\,pXf\,\lambda)) + \\
&pYm\,q\,(pXf\,wAf\,wam\,(-1 + 2\,(-1 + R)\,\alpha f)\,\lambda + waf\,wAm\,(-2 + 2\,\alpha f + \\
&\lambda + pXf\,\lambda - 2\,pXf\,\alpha f\,\lambda + 2\,R\,(-1 + \alpha f)\,(-1 + pXf\,\lambda))))) + \\
&2\,FAa^2\,(wAf\,wam\,(pYm^2\,q^2\,waf\,wAm\,\alpha f\,(-1 - 2\,R\,(-1 + \alpha f) + \alpha f) + \\
&pXf\,(-1 + q)\,wAf\,wam\,\lambda\,(\alpha f - R\,\alpha f + pXf\,(-1 + q)\,\lambda) + \\
&pYm\,q\,(pXf\,(-1 + q)\,(-1 + R)\,wAf\,wam\,\alpha f\,\lambda + waf\,wAm \\
&(-1 + \alpha f)\,((-1 + 2\,R)\,\alpha f + pXf\,(-1 + q)\,(-1 + R)\,\lambda))) + \\
&pXm^2\,(-1 + q)^2\,(pXf\,wAf^2\,wam^2\,\lambda\,((-1 + R)\,\alpha f + pXf\,\lambda) + \\
&(-1 + pXf)\,waf^2\,wAm^2\,\lambda\,(1 + R\,(-1 + \alpha f) - \alpha f - \lambda + pXf\,\lambda) + \\
&waf\,wAf\,wam\,wAm\,((1 - 2\,R)\,\alpha f^2 + pXf\,\lambda\,(1 - R + 2\,(-1 + pXf)\,\lambda) + \\
&\alpha f\,(-1 + \lambda - 2\,pXf\,\lambda + R\,(2 + (-1 + 2\,pXf)\,\lambda)))) - \\
&pXm\,(-1 + q)\,((-1 + pXf)\,pYm\,q\,(-1 + R)\,waf^2\,wAm^2\,(-1 + \alpha f)\,\lambda + \\
&pXf\,wAf^2\,wam^2\,\lambda\,((-2 + q + pYm\,q)\,(-1 + R)\,\alpha f + 2\,pXf\,(-1 + q)\,\lambda) + \\
&waf\,wAf\,wam\,wAm\,(-(-1 + 2\,pYm\,q)\,(-1 + 2\,R)\,\alpha f^2 + pXf\,\lambda \\
&(-1 + R + 2\,\lambda - 2\,pXf\,\lambda + q\,(1 + pYm - R - pYm\,R - 2\,\lambda + 2\,pXf\,\lambda)) + \\
&\alpha f\,(1 - \lambda + 2\,pXf\,\lambda - pXf\,q\,\lambda + R\,(-2 + \lambda - 2\,pXf\,\lambda + pXf\,q\,\lambda) + \\
&pYm\,q\,(-2 + \lambda - 2\,pXf\,\lambda + R\,(4 - \lambda + 2\,pXf\,\lambda)))))))) \\
&(2\,Faa^2\,(-1 + pXf)\,(-1 + pXm)\,(-1 + q)\,waf^2\,wam^2\,\lambda\,(1 - 2\,\lambda + 2\,pXf\,\lambda + \\
&2\,q\,\lambda - 2\,pXf\,q\,\lambda + pXm\,(-1 + q)\,(1 + 2\,(-1 + pXf)\,\lambda - \rho) + \\
&pYm\,q\,(-1 + \rho) - \rho) + 2\,FAA^2\,pXf\,pXm\,(-1 + q)\,wAf^2 \\
&wAm^2\,\lambda\,(-pYm\,q\,(-1 + \rho) + pXm\,(-1 + q)\,(-1 + 2\,pXf\,\lambda + \rho)) - \\
&FAa\,FAA\,wAf\,wAm\,(pYm\,q\,(-1 + \rho)\,(2\,pXf\,(-1 + q)\,wAf\,wam\,\lambda + \\
&pYm\,q\,waf\,wAm\,(-1 + \alpha f)\,(-2 + r + R + \rho)) + pXm^2\,(-1 + q)^2 \\
&(2\,pXf\,wAf\,wam\,\lambda\,(-1 + 4\,pXf\,\lambda + \rho + \alpha f\,(-2 + r + R + \rho)) + waf\,wAm \\
&(-2 + 2\,\alpha f + 2\,\lambda + 2\,pXf\,\lambda - 4\,pXf\,\alpha f\,\lambda - 8\,pXf\,\lambda^2 + 8\,pXf^2\,\lambda^2 + 3\,\rho - 3\,\alpha f \\
&\rho - 2\,\lambda\,\rho + 2\,pXf\,\alpha f\,\lambda\,\rho - \rho^2 + \alpha f\,\rho^2 + r\,(-1 + \alpha f)\,(-1 + 2\,pXf\,\lambda + \rho) + \\
&R\,(-1 + \alpha f)\,(-1 + 2\,pXf\,\lambda + \rho))) - 2\,pXm\,(-1 + q)\,(pXf\,wAf\,wam \\
&\lambda\,(1 - 4\,pXf\,\lambda - \rho - \alpha f\,(-2 + r + R + \rho) + q\,(-1 + 4\,pXf\,\lambda + \rho)) + \\
&pYm\,q\,(pXf\,wAf\,wam\,\lambda\,(-1 + \rho + \alpha f\,(-2 + r + R + \rho)) + waf\,wAm\,(-2 + 2\,\alpha f + \\
&\lambda + pXf\,\lambda - 2\,pXf\,\alpha f\,\lambda + 3\,\rho - 3\,\alpha f\,\rho - \lambda\,\rho + pXf\,\alpha f\,\lambda\,\rho - \rho^2 + \alpha f\,\rho^2 + \\
&r\,(-1 + \alpha f)\,(-1 + pXf\,\lambda + \rho) + R\,(-1 + \alpha f)\,(-1 + pXf\,\lambda + \rho))))) + \\
&2\,FAa^2\,(pXm^2\,(-1 + q)^2\,((-1 + pXf)\,waf^2\,wAm^2\,\lambda\,(2 + r\,(-1 + \alpha f) + \\
&R\,(-1 + \alpha f) - 2\,\alpha f - 2\,\lambda + 2\,pXf\,\lambda - \rho + \alpha f\,\rho) + \\
&pXf\,wAf^2\,wam^2\,\lambda\,(2\,pXf\,\lambda + \alpha f\,(-2 + r + R + \rho)) + waf\,wAf\,wam \\
&wAm\,(pXf\,\lambda\,(2 - r - R - 4\,\lambda + 4\,pXf\,\lambda - \rho) + 2\,(-1 + r + R)\,\alpha f^2\,(-1 + \rho) + \\
&\alpha f\,(-2 + 2\,\lambda - 4\,pXf\,\lambda + R\,(2 - \lambda + 2\,pXf\,\lambda - 2\,\rho) + \\
&r\,(2 + (-1 + 2\,pXf)\,\lambda - 2\,\rho) + 2\,\rho - \lambda\,\rho + 2\,pXf\,\lambda\,\rho))) + \\
&wAf\,wam\,(2\,pYm^2\,q^2\,(-1 + r + R)\,waf\,wAm\,(-1 + \alpha f)\,\alpha f\,(-1 + \rho) - \\
&pXf\,(-1 + q)\,wAf\,wam\,\lambda\,(-2\,pXf\,(-1 + q)\,\lambda + \alpha f\,(-2 + r + R + \rho)) +
\end{aligned}
$$

```
              pYm q (pXf (-1 + q) wAf wam αf λ (-2 + r + R + ρ) - waf wAm (-1 + αf)
                  (2 (-1 + r + R) αf (-1 + ρ) - pXf (-1 + q) λ (-2 + r + R + ρ)))) -
           pXm (-1 + q) ((-1 + pXf) pYm q waf^2 wAm^2 (-1 + αf) λ (-2 + r + R + ρ) +
              pXf wAf^2 wam^2 λ (4 pXf (-1 + q) λ + (-2 + q + pYm q) αf (-2 + r + R + ρ)) +
              waf wAf wam wAm (2 (-1 + 2 pYm q) (-1 + r + R) αf^2 (-1 + ρ) +
                 αf (2 - 2 R - 2 λ + 4 pXf λ - 2 pXf q λ + R λ - 2 pXf R λ + pXf q R λ - 2 ρ +
                    2 R ρ + λ ρ - 2 pXf λ ρ + pXf q λ ρ + r (-2 + λ - 2 pXf λ + pXf q λ +
                       2 ρ) + pYm q (-4 + 2 λ - 4 pXf λ + R (4 - λ + 2 pXf λ - 4 ρ) +
                       r (4 + (-1 + 2 pXf) λ - 4 ρ) + 4 ρ - λ ρ + 2 pXf λ ρ)) + pXf λ
                  (-2 + r + R + 4 λ - 4 pXf λ + ρ - q (-2 + r + R + 4 λ - 4 pXf λ +
                       ρ + pYm (-2 + r + R + ρ)))))) + Faa waf wam (FAA wAf wAm
            (pXm^2 (-1 + q)^2 (8 (-1 + pXf) pXf λ^2 - 2 λ (-1 + ρ) - (-1 + ρ)^2) -
              pYm q (1 + 2 (-1 + pXf + q - pXf q) λ + pYm q (-1 + ρ) - ρ) (-1 + ρ) +
              pXm (-1 + q) (-8 (-1 + pXf) pXf (-1 + q) λ^2 - 2 (1 + (-1 + pXf) q)
                 λ (-1 + ρ) - (-1 + ρ)^2 + 2 pYm q (-1 + ρ) (-1 + λ + ρ))) +
           FAa (wAf wam (-2 pXf (-1 + pXm) (-1 + q) λ (1 - 4 λ + 4 pXf λ + 4 q λ -
                    4 pXf q λ + pXm (-1 + q) (1 + 4 (-1 + pXf) λ - ρ) + pYm q (-1 + ρ) -
                    ρ) + (1 + pXm (-1 + q) - pYm q) αf (-2 + r + R + ρ) (-1 + 2 λ -
                    2 pXf λ - 2 q λ + 2 pXf q λ - pXm (-1 + q) (1 + 2 (-1 + pXf) λ - ρ) +
                    ρ + pYm (q - q ρ))) - 2 (-1 + pXf) (-1 + q) waf
              wAm λ (pYm q (-1 + αf) (-2 + r + R + ρ) + pXm^2 (-1 + q)
                  (3 + r (-1 + αf) + R (-1 + αf) - 2 αf - 4 λ + 4 pXf λ - 2 ρ + αf ρ) -
                 pXm (-3 + r + R + 2 αf - r αf - R αf + 4 λ - 4 pXf λ + 2 ρ - αf ρ +
                    q (2 - R + r (-1 + αf) - 2 αf + R αf - 4 λ + 4 pXf λ - ρ + αf ρ + pYm
                       (3 + r (-1 + αf) + R (-1 + αf) - 2 αf - 2 ρ + αf ρ))))))))) /
      (16 (-1 + q)^4 (Faa (-1 + pXf) (-1 + pXm) waf wam + FAA pXf pXm wAf wAm +
           FAa (pXm waf wAm - pXf ((-1 + pXm) wAf wam +
                 pXm waf wAm)))^4) /. subs, λ]] - 1
  ]
 ]
```

Invasion of neo-W into XY (allows two equilibria)

```
Clear[invasionplotXY2]
invasionplotXY2[
  {tryFAA_, tryFAa_, tryFaa_, tryMAA_, tryMAa_, tryMaa_, tryFA_, tryFa_, tryMA_,
   tryMa_, tryFdA_, tryMdA_, tryr_, tryR_, tryρ_}, {pXf_, pXm_, pYm_, q_}] :=
 Block[{subs, r, R, ρ},
  subs =
   {FAA → tryFAA, FAa → tryFAa, Faa → tryFaa, MAA → tryMAA, MAa → tryMAa, Maa → tryMaa,
    wAf → tryFA, waf → tryFa, wAm → tryMA, wam → tryMa, αf →  tryFdA, αm →  tryMdA};
  r = tryr;
  R = tryR;
  ρ = tryρ;
  Max[
   λ /. NSolve[0 == 0 == (λ^4 (2 Faa^2 (-1 + pXf) (-1 + pXm) (-1 + q) waf^2 wam^2 λ (1 - pYm q - 2 λ +
                2 pXf λ + 2 q λ - 2 pXf q λ + pXm (-1 + q) (1 + 2 (-1 + pXf) λ)) -
            Faa waf wam (FAA wAf wAm (pYm q (-1 + pYm q + 2 (-1 + pXf) (-1 + q) λ) -
```

$$\mathrm{pXm}^2\,(-1+q)^2\,\left(-1+2\lambda+8\,(-1+\mathrm{pXf})\,\mathrm{pXf}\,\lambda^2\right)+\mathrm{pXm}\,(-1+q)\,\left(1+2\,\mathrm{pYm}\,q\right.$$
$$\left.(-1+\lambda)-2\,(1+(-1+\mathrm{pXf})\,q)\,\lambda+8\,(-1+\mathrm{pXf})\,\mathrm{pXf}\,(-1+q)\,\lambda^2\right)\Big)+$$
$$2\,\mathrm{FAa}\,\Big(\mathrm{wAf}\,\mathrm{wam}\,((1+\mathrm{pXm}\,(-1+q)-\mathrm{pYm}\,q)\,(-1+R)\,\alpha f\,(1-\mathrm{pYm}\,q-2\lambda+$$
$$2\,\mathrm{pXf}\,\lambda+2\,q\,\lambda-2\,\mathrm{pXf}\,q\,\lambda+\mathrm{pXm}\,(-1+q)\,(1+2\,(-1+\mathrm{pXf})\,\lambda))+$$
$$\mathrm{pXf}\,(-1+\mathrm{pXm})\,(-1+q)\,\lambda\,(1-\mathrm{pYm}\,q-4\lambda+4\,\mathrm{pXf}\,\lambda+4\,q\,\lambda-$$
$$4\,\mathrm{pXf}\,q\,\lambda+\mathrm{pXm}\,(-1+q)\,(1+4\,(-1+\mathrm{pXf})\,\lambda)))+$$
$$(-1+\mathrm{pXf})\,(-1+q)\,\mathrm{waf}\,\mathrm{wAm}\,\lambda\,\Big(2\,\mathrm{pYm}\,q\,(-1+R)\,(-1+\alpha f)+$$
$$\mathrm{pXm}^2\,(-1+q)\,(3+2\,R\,(-1+\alpha f)-2\,\alpha f-4\lambda+4\,\mathrm{pXf}\,\lambda)+$$
$$\mathrm{pXm}\,(3+2\,R\,(-1+\alpha f)-2\,\alpha f-4\lambda+4\,\mathrm{pXf}\,\lambda+q\,(\mathrm{pYm}\,(-3+2\,R+2\,\alpha f-$$
$$2\,R\,\alpha f)+2\,(-1+R+\alpha f-R\,\alpha f+2\lambda-2\,\mathrm{pXf}\,\lambda))))\Big)\Big)+$$
$$2\,\Big(\mathrm{FAA}^2\,\mathrm{pXf}\,\mathrm{pXm}\,(-1+q)\,\mathrm{wAf}^2\,\mathrm{wAm}^2\,\lambda\,(\mathrm{pYm}\,q+\mathrm{pXm}\,(-1+q)\,(-1+2\,\mathrm{pXf}\,\lambda))-$$
$$\mathrm{FAa}\,\mathrm{FAA}\,\mathrm{wAf}\,\mathrm{wAm}\,\Big(\mathrm{pYm}\,q\,(\mathrm{pYm}\,q\,\mathrm{waf}\,\mathrm{wAm}\,(-1+R+\alpha f-R\,\alpha f)-\mathrm{pXf}\,(-1+q)$$
$$\mathrm{wAf}\,\mathrm{wam}\,\lambda)+\mathrm{pXm}^2\,(-1+q)^2\,\Big(\mathrm{pXf}\,\mathrm{wAf}\,\mathrm{wam}\,\lambda\,(-1+2\,(-1+R)\,\alpha f+$$
$$4\,\mathrm{pXf}\,\lambda)+\mathrm{waf}\,\mathrm{wAm}\,\Big(-1+\alpha f+\lambda+\mathrm{pXf}\,\lambda-2\,\mathrm{pXf}\,\alpha f\,\lambda-4\,\mathrm{pXf}\,\lambda^2+$$
$$4\,\mathrm{pXf}^2\,\lambda^2+R\,(-1+\alpha f)\,(-1+2\,\mathrm{pXf}\,\lambda)\Big)\Big)-\mathrm{pXm}\,(-1+q)$$
$$(\mathrm{pXf}\,\mathrm{wAf}\,\mathrm{wam}\,\lambda\,(1+2\,\alpha f-2\,R\,\alpha f-4\,\mathrm{pXf}\,\lambda+q\,(-1+4\,\mathrm{pXf}\,\lambda))+$$
$$\mathrm{pYm}\,q\,(\mathrm{pXf}\,\mathrm{wAf}\,\mathrm{wam}\,(-1+2\,(-1+R)\,\alpha f)\,\lambda+\mathrm{waf}\,\mathrm{wAm}\,(-2+2\,\alpha f+$$
$$\lambda+\mathrm{pXf}\,\lambda-2\,\mathrm{pXf}\,\alpha f\,\lambda+2\,R\,(-1+\alpha f)\,(-1+\mathrm{pXf}\,\lambda))))\Big)+$$
$$2\,\mathrm{FAa}^2\,\Big(\mathrm{wAf}\,\mathrm{wam}\,\Big(\mathrm{pYm}^2\,q^2\,\mathrm{waf}\,\mathrm{wAm}\,\alpha f\,(-1-2\,R\,(-1+\alpha f)+\alpha f)+$$
$$\mathrm{pXf}\,(-1+q)\,\mathrm{wAf}\,\mathrm{wam}\,\lambda\,(\alpha f-R\,\alpha f+\mathrm{pXf}\,(-1+q)\,\lambda)+$$
$$\mathrm{pYm}\,q\,(\mathrm{pXf}\,(-1+q)\,(-1+R)\,\mathrm{wAf}\,\mathrm{wam}\,\alpha f\,\lambda+\mathrm{waf}\,\mathrm{wAm}$$
$$(-1+\alpha f)\,((-1+2\,R)\,\alpha f+\mathrm{pXf}\,(-1+q)\,(-1+R)\,\lambda))\Big)+$$
$$\mathrm{pXm}^2\,(-1+q)^2\,\Big(\mathrm{pXf}\,\mathrm{wAf}^2\,\mathrm{wam}^2\,\lambda\,((-1+R)\,\alpha f+\mathrm{pXf}\,\lambda)+$$
$$(-1+\mathrm{pXf})\,\mathrm{waf}^2\,\mathrm{wAm}^2\,\lambda\,(1+R\,(-1+\alpha f)-\alpha f-\lambda+\mathrm{pXf}\,\lambda)+$$
$$\mathrm{waf}\,\mathrm{wAf}\,\mathrm{wam}\,\mathrm{wAm}\,\Big((1-2\,R)\,\alpha f^2+\mathrm{pXf}\,\lambda\,(1-R+2\,(-1+\mathrm{pXf})\,\lambda)+$$
$$\alpha f\,(-1+\lambda-2\,\mathrm{pXf}\,\lambda+R\,(2+(-1+2\,\mathrm{pXf})\,\lambda))\Big)\Big)-$$
$$\mathrm{pXm}\,(-1+q)\,\Big((-1+\mathrm{pXf})\,\mathrm{pYm}\,q\,(-1+R)\,\mathrm{waf}^2\,\mathrm{wAm}^2\,(-1+\alpha f)\,\lambda+$$
$$\mathrm{pXf}\,\mathrm{wAf}^2\,\mathrm{wam}^2\,\lambda\,((-2+q+\mathrm{pYm}\,q)\,(-1+R)\,\alpha f+2\,\mathrm{pXf}\,(-1+q)\,\lambda)+$$
$$\mathrm{waf}\,\mathrm{wAf}\,\mathrm{wam}\,\mathrm{wAm}\,\Big(-(-1+2\,\mathrm{pYm}\,q)\,(-1+2\,R)\,\alpha f^2+\mathrm{pXf}\,\lambda$$
$$(-1+R+2\lambda-2\,\mathrm{pXf}\,\lambda+q\,(1+\mathrm{pYm}-R-\mathrm{pYm}\,R-2\lambda+2\,\mathrm{pXf}\,\lambda))+$$
$$\alpha f\,(1-\lambda+2\,\mathrm{pXf}\,\lambda-\mathrm{pXf}\,q\,\lambda+R\,(-2+\lambda-2\,\mathrm{pXf}\,\lambda+\mathrm{pXf}\,q\,\lambda)+$$
$$\mathrm{pYm}\,q\,(-2+\lambda-2\,\mathrm{pXf}\,\lambda+R\,(4-\lambda+2\,\mathrm{pXf}\,\lambda)))\Big)\Big)\Big)\Big)$$
$$\Big(2\,\mathrm{Faa}^2\,(-1+\mathrm{pXf})\,(-1+\mathrm{pXm})\,(-1+q)\,\mathrm{waf}^2\,\mathrm{wam}^2\,\lambda\,(1-2\lambda+2\,\mathrm{pXf}\,\lambda+$$
$$2\,q\,\lambda-2\,\mathrm{pXf}\,q\,\lambda+\mathrm{pXm}\,(-1+q)\,(1+2\,(-1+\mathrm{pXf})\,\lambda-\rho)+$$
$$\mathrm{pYm}\,q\,(-1+\rho)-\rho)+2\,\mathrm{FAA}^2\,\mathrm{pXf}\,\mathrm{pXm}\,(-1+q)\,\mathrm{wAf}^2$$
$$\mathrm{wAm}^2\,\lambda\,(-\mathrm{pYm}\,q\,(-1+\rho)+\mathrm{pXm}\,(-1+q)\,(-1+2\,\mathrm{pXf}\,\lambda+\rho))-$$
$$\mathrm{FAa}\,\mathrm{FAA}\,\mathrm{wAf}\,\mathrm{wAm}\,\Big(\mathrm{pYm}\,q\,(-1+\rho)\,(2\,\mathrm{pXf}\,(-1+q)\,\mathrm{wAf}\,\mathrm{wam}\,\lambda+$$
$$\mathrm{pYm}\,q\,\mathrm{waf}\,\mathrm{wAm}\,(-1+\alpha f)\,(-2+r+R+\rho))+$$
$$\mathrm{pXm}^2\,(-1+q)^2\,\Big(2\,\mathrm{pXf}\,\mathrm{wAf}\,\mathrm{wam}\,\lambda\,(-1+4\,\mathrm{pXf}\,\lambda+\rho+\alpha f\,(-2+r+R+\rho))+$$
$$\mathrm{waf}\,\mathrm{wAm}\,\Big(-2+2\,\alpha f+2\lambda+2\,\mathrm{pXf}\,\lambda-4\,\mathrm{pXf}\,\alpha f\,\lambda-8\,\mathrm{pXf}\,\lambda^2+8\,\mathrm{pXf}^2\,\lambda^2+$$
$$3\rho-3\,\alpha f\,\rho-2\lambda\rho+2\,\mathrm{pXf}\,\alpha f\,\lambda\rho-\rho^2+\alpha f\,\rho^2+r\,(-1+\alpha f)$$
$$(-1+2\,\mathrm{pXf}\,\lambda+\rho)+R\,(-1+\alpha f)\,(-1+2\,\mathrm{pXf}\,\lambda+\rho)\Big)\Big)-2\,\mathrm{pXm}\,(-1+q)$$
$$\Big(\mathrm{pXf}\,\mathrm{wAf}\,\mathrm{wam}\,\lambda\,(1-4\,\mathrm{pXf}\,\lambda-\rho-\alpha f\,(-2+r+R+\rho))+q\,(-1+4\,\mathrm{pXf}\,\lambda+\rho))+$$
$$\mathrm{pYm}\,q\,\Big(\mathrm{pXf}\,\mathrm{wAf}\,\mathrm{wam}\,\lambda\,(-1+\rho+\alpha f\,(-2+r+R+\rho))+\mathrm{waf}\,\mathrm{wAm}\,\Big(-2+2\,\alpha f+$$
$$\lambda+\mathrm{pXf}\,\lambda-2\,\mathrm{pXf}\,\alpha f\,\lambda+3\rho-3\,\alpha f\,\rho-\lambda\rho+\mathrm{pXf}\,\alpha f\,\lambda\rho-\rho^2+\alpha f\,\rho^2+$$

```
                    r (-1 + αf) (-1 + pXf λ + ρ) + R (-1 + αf) (-1 + pXf λ + ρ))))) +
           2 FAa^2 (pXm^2 (-1 + q)^2 ((-1 + pXf) waf^2 wAm^2 λ (2 + r (-1 + αf) +
                    R (-1 + αf) - 2 αf - 2 λ + 2 pXf λ - ρ + αf ρ) +
                 pXf wAf^2 wam^2 λ (2 pXf λ + αf (-2 + r + R + ρ)) + waf wAf wam
                  wAm (pXf λ (2 - r - R - 4 λ + 4 pXf λ - ρ) + 2 (-1 + r + R) αf^2 (-1 + ρ) +
                    αf (-2 + 2 λ - 4 pXf λ + R (2 - λ + 2 pXf λ - 2 ρ) +
                       r (2 + (-1 + 2 pXf) λ - 2 ρ) + 2 ρ - λ ρ + 2 pXf λ ρ))) +
              wAf wam (2 pYm^2 q^2 (-1 + r + R) waf wAm (-1 + αf) αf (-1 + ρ) -
                 pXf (-1 + q) wAf wam λ (-2 pXf (-1 + q) λ + αf (-2 + r + R + ρ)) +
                 pYm q (pXf (-1 + q) wAf wam αf λ (-2 + r + R + ρ) - waf wAm (-1 + αf)
                      (2 (-1 + r + R) αf (-1 + ρ) - pXf (-1 + q) λ (-2 + r + R + ρ)))) -
              pXm (-1 + q) ((-1 + pXf) pYm q waf^2 wAm^2 (-1 + αf) λ (-2 + r + R + ρ) +
                 pXf wAf^2 wam^2 λ (4 pXf (-1 + q) λ + (-2 + q + pYm q) αf (-2 + r + R + ρ)) +
                 waf wAf wam wAm (2 (-1 + 2 pYm q) (-1 + r + R) αf^2 (-1 + ρ) +
                    αf (2 - 2 R - 2 λ + 4 pXf λ - 2 pXf q λ + R λ - 2 pXf R λ + pXf q R λ - 2 ρ +
                       2 R ρ + λ ρ - 2 pXf λ ρ + pXf q λ ρ + r (-2 + λ - 2 pXf λ + pXf q λ +
                          2 ρ) + pYm q (-4 + 2 λ - 4 pXf λ + R (4 - λ + 2 pXf λ - 4 ρ) +
                          r (4 + (-1 + 2 pXf) λ - 4 ρ) + 4 ρ - λ ρ + 2 pXf λ ρ)) + pXf λ
                     (-2 + r + R + 4 λ - 4 pXf λ + ρ - q (-2 + r + R + 4 λ - 4 pXf λ +
                          ρ + pYm (-2 + r + R + ρ)))))) + Faa waf wam (FAA wAf wAm
               (pXm^2 (-1 + q)^2 (8 (-1 + pXf) pXf λ^2 - 2 λ (-1 + ρ) - (-1 + ρ)^2) -
                 pYm q (1 + 2 (-1 + pXf + q - pXf q) λ + pYm q (-1 + ρ) - ρ) (-1 + ρ) +
                 pXm (-1 + q) (-8 (-1 + pXf) pXf (-1 + q) λ^2 - 2 (1 + (-1 + pXf) q)
                     λ (-1 + ρ) - (-1 + ρ)^2 + 2 pYm q (-1 + ρ) (-1 + λ + ρ))) +
              FAa (wAf wam (-2 pXf (-1 + pXm) (-1 + q) λ (1 - 4 λ + 4 pXf λ +
                          4 q λ - 4 pXf q λ + pXm (-1 + q) (1 + 4 (-1 + pXf) λ - ρ) +
                          pYm q (-1 + ρ) - ρ) + (1 + pXm (-1 + q) - pYm q) αf
                       (-2 + r + R + ρ) (-1 + 2 λ - 2 pXf λ - 2 q λ + 2 pXf q λ -
                          pXm (-1 + q) (1 + 2 (-1 + pXf) λ - ρ) + ρ + pYm (q - q ρ))) -
                 2 (-1 + pXf) (-1 + q) waf wAm λ (pYm q (-1 + αf) (-2 + r + R + ρ) +
                    pXm^2 (-1 + q) (3 + r (-1 + αf) + R (-1 + αf) - 2 αf - 4 λ + 4 pXf λ - 2 ρ +
                       αf ρ) - pXm (-3 + r + R + 2 αf - r αf - R αf + 4 λ - 4 pXf λ + 2 ρ -
                       αf ρ + q (2 - R + r (-1 + αf) - 2 αf + R αf - 4 λ + 4 pXf λ - ρ + αf ρ +
                          pYm (3 + r (-1 + αf) + R (-1 + αf) - 2 αf - 2 ρ + αf ρ)))))))) /
        (16 (-1 + q)^4 (Faa (-1 + pXf) (-1 + pXm) waf wam + FAA pXf pXm wAf wAm + FAa
                (pXm waf wAm - pXf ((-1 + pXm) wAf wam + pXm waf wAm)))^4) /. subs, λ]] - 1
  ]
```

Recursions

Code to convert allele frequencies (from the equilibrium calculations) into haplotype frequencies, as needed for the simulations:
[Assumes that all haplotypes convert from M to m at a small rate, pm. This will cause imbalanced sex ratios at first, so ignore the first few generations, as it reequilibrates.]

```
Clear[startgen]
startgen[{pXf_, pXm_, pYm_, q_}, pm_] :=
 startgen[{pXf, pXm, pYm, q}, pm] =
  {XAMf, XaMf, XAmf, Xamf, YAMf, YaMf, YAmf,
    Yamf, XAMm, XaMm, XAmm, Xamm, YAMm, YaMm, YAmm, Yamm} /. {
    XAMf → pXf (1 - pm),
    XaMf → (1 - pXf) (1 - pm),
    YAMf → 0 (1 - pm),
    YaMf → 0 (1 - pm),
    XAMm → (1 - q) pXm (1 - pm),
    XaMm → (1 - q) (1 - pXm) (1 - pm),
    YAMm → q pYm (1 - pm),
    YaMm → q (1 - pYm) (1 - pm),
    XAmf → pXf (pm),
    Xamf → (1 - pXf) (pm),
    YAmf → 0 (pm),
    Yamf → 0 (pm),
    XAmm → (1 - q) pXm (pm),
    Xamm → (1 - q) (1 - pXm) (pm),
    YAmm → q pYm (pm),
    Yamm → q (1 - pYm) (pm)
   }
```

Recursion for haplotype frequencies

```
Clear[generation];
generation[{FAA_, FAa_, Faa_, MAA_, MAa_, Maa_, wAf_, waf_, wAm_, wam_,
   αf_, αm_, r_, R_, ρ_, k_}, {XAMf_, XaMf_, XAmf_, Xamf_, YAMf_, YaMf_,
   YAmf_, Yamf_, XAMm_, XaMm_, XAmm_, Xamm_, YAMm_, YaMm_, YAmm_, Yamm_}] :=
 generation[{FAA, FAa, Faa, MAA, MAa, Maa, wAf, waf, wAm, wam, αf, αm, r, R, ρ, k},
   {XAMf, XaMf, XAmf, Xamf, YAMf, YaMf, YAmf, Yamf,
    XAMm, XaMm, XAmm, Xamm, YAMm, YaMm, YAmm, Yamm}] =
  Block[
   {
    kXXMM = 1,
    kXYMM = 0,
    kYYMM = 0,
    kXXMm = k,
    kXYMm = k,
    kYYMm = k,
    kXXmm = k,
    kXYmm = k,
    kYYmm = k
   },

   (*HAPLOID COMPETITION*)

   (*mean fitness of female gametes*)
   wbarHapFemale = (wAf XAMf + waf XaMf + wAf XAmf + waf Xamf) +
     (wAf YAMf + waf YaMf + wAf YAmf + waf Yamf);

   (*non-mutant eggs*)
   XAMfs = wAf XAMf / wbarHapFemale;
   XaMfs = waf XaMf / wbarHapFemale;
   YAMfs = wAf YAMf / wbarHapFemale;
```

```
YaMfs = waf YaMf / wbarHapFemale;

(*mutant eggs*)
XAmfs = wAf XAmf / wbarHapFemale;
Xamfs = waf Xamf / wbarHapFemale;
YAmfs = wAf YAmf / wbarHapFemale;
Yamfs = waf Yamf / wbarHapFemale;

(*mean fitness of male gametes*)
wbarHapMale = (wAm XAMm + wam XaMm + wAm XAmm + wam Xamm) +
   (wAm YAMm + wam YaMm + wAm YAmm + wam Yamm);

(*non-mutant male gametes*)
XAMms = wAm XAMm / wbarHapMale;
XaMms = wam XaMm / wbarHapMale;
YAMms =  wAm YAMm / wbarHapMale;
YaMms =  wam YaMm / wbarHapMale;

(*mutant male gametes*)
XAmms = wAm XAmm / wbarHapMale;
Xamms = wam Xamm / wbarHapMale;
YAmms =  wAm YAmm / wbarHapMale;
Yamms =  wam Yamm / wbarHapMale;

(*RANDOM MATING*)

(*MM Homozygotes*)
(*XM-XM females*)
XAMXAMfemale = kXXMM (XAMfs XAMms);
XAMXaMfemale = kXXMM (XAMfs XaMms + XaMfs XAMms );
XaMXaMfemale = kXXMM (XaMfs XaMms);
(*XM-XM males*)
XAMXAMmale = (1 - kXXMM) (XAMfs XAMms);
XAMXaMmale = (1 - kXXMM) (XAMfs XaMms + XaMfs XAMms );
XaMXaMmale = (1 - kXXMM) (XaMfs XaMms);

(*XM-YM females*)
XAMYAMfemale = kXYMM (XAMfs YAMms + YAMfs XAMms);
XAMYaMfemale = kXYMM (XAMfs YaMms + YaMfs XAMms);
XaMYAMfemale = kXYMM (XaMfs YAMms + YAMfs XaMms);
XaMYaMfemale = kXYMM (XaMfs YaMms + YaMfs XaMms);
(*XM-YM males*)
XAMYAMmale = (1 - kXYMM) (XAMfs YAMms + YAMfs XAMms);
XAMYaMmale = (1 - kXYMM) (XAMfs YaMms + YaMfs XAMms);
XaMYAMmale = (1 - kXYMM) (XaMfs YAMms + YAMfs XaMms);
XaMYaMmale = (1 - kXYMM) (XaMfs YaMms + YaMfs XaMms);

(*YM-YM females*)
YAMYAMfemale = kYYMM (YAMfs YAMms);
YAMYaMfemale = kYYMM (YAMfs YaMms + YaMfs YAMms);
YaMYaMfemale = kYYMM (YaMfs YaMms);
(*YM-YM males*)
YAMYAMmale = (1 - kYYMM) (YAMfs YAMms);
YAMYaMmale = (1 - kYYMM) (YAMfs YaMms + YaMfs YAMms);
YaMYaMmale = (1 - kYYMM) (YaMfs YaMms);
```

```
(*Mm heterozygotes*)
(*XM-Xm females*)
XAmXAMfemale = kXXMm (XAmfs XAMms + XAMfs XAmms);
XAmXaMfemale = kXXMm (XAmfs XaMms + XaMfs XAmms );
XAMXamfemale = kXXMm (XAMfs Xamms + Xamfs XAMms);
XamXaMfemale = kXXMm (Xamfs XaMms + XaMfs Xamms);
(*XM-Xm males*)
XAmXAMmale = (1 - kXXMm) (XAmfs XAMms + XAMfs XAmms);
XAmXaMmale = (1 - kXXMm) (XAmfs XaMms + XaMfs XAmms );
XAMXammale = (1 - kXXMm) (XAMfs Xamms + Xamfs XAMms);
XamXaMmale = (1 - kXXMm) (Xamfs XaMms + XaMfs Xamms);

(*Xm-YM females*)
XAmYAMfemale = kXYMm (XAmfs YAMms + YAMfs XAmms);
XAmYaMfemale = kXYMm (XAmfs YaMms + YaMfs XAmms);
XamYAMfemale = kXYMm (Xamfs YAMms + YAMfs Xamms);
XamYaMfemale = kXYMm (Xamfs YaMms + YaMfs Xamms);
(*Xm-YM males*)
XAmYAMmale = (1 - kXYMm) (XAmfs YAMms + YAMfs XAmms);
XAmYaMmale = (1 - kXYMm) (XAmfs YaMms + YaMfs XAmms);
XamYAMmale = (1 - kXYMm) (Xamfs YAMms + YAMfs Xamms);
XamYaMmale = (1 - kXYMm) (Xamfs YaMms + YaMfs Xamms);

(*XM-Ym females*)
XAMYAmfemale = kXYMm (XAMfs YAmms + YAmfs XAMms);
XAMYamfemale = kXYMm (XAMfs Yamms + Yamfs XAMms );
XaMYAmfemale = kXYMm (XaMfs YAmms + YAmfs XaMms );
XaMYamfemale = kXYMm (XaMfs Yamms + Yamfs XaMms);
(*XM-Ym females*)
XAMYAmmale = (1 - kXYMm) (XAMfs YAmms + YAmfs XAMms);
XAMYammale = (1 - kXYMm) (XAMfs Yamms + Yamfs XAMms );
XaMYAmmale = (1 - kXYMm) (XaMfs YAmms + YAmfs XaMms );
XaMYammale = (1 - kXYMm) (XaMfs Yamms + Yamfs XaMms);

(*Ym-YM females*)
YAmYAMfemale = kYYMm (YAmfs YAMms + YAMfs YAmms);
YAmYaMfemale = kYYMm (YAmfs YaMms + YaMfs YAmms);
YamYAMfemale = kYYMm (Yamfs YAMms + YAMfs Yamms);
YamYaMfemale = kYYMm (Yamfs YaMms + YaMfs Yamms);
(*Ym-YM males*)
YAmYAMmale = (1 - kYYMm) (YAmfs YAMms + YAMfs YAmms);
YAmYaMmale = (1 - kYYMm) (YAmfs YaMms + YaMfs YAmms);
YamYAMmale = (1 - kYYMm) (Yamfs YAMms + YAMfs Yamms);
YamYaMmale = (1 - kYYMm) (Yamfs YaMms + YaMfs Yamms);

(*mm heterozygotes*)
(*Xm-Xm females*)
XAmXAmfemale = kXXmm (XAmfs XAmms);
XAmXamfemale = kXXmm (XAmfs Xamms + Xamfs XAmms );
XamXamfemale = kXXmm (Xamfs Xamms);
(*Xm-Xm males*)
XAmXAmmale = (1 - kXXmm) (XAmfs XAmms);
XAmXammale = (1 - kXXmm) (XAmfs Xamms + Xamfs XAmms );
XamXammale = (1 - kXXmm) (Xamfs Xamms);
```

```
(*Xm-Ym females*)
XAmYAmfemale = kXYmm (XAmfs YAmms + YAmfs XAmms );
XAmYamfemale = kXYmm (XAmfs Yamms + Yamfs XAmms );
XamYAmfemale = kXYmm (Xamfs YAmms + YAmfs Xamms );
XamYamfemale = kXYmm (Xamfs Yamms + Yamfs Xamms);
(*Xm-Ym males*)
XAmYAmmale = (1 - kXYmm) (XAmfs YAmms + YAmfs XAmms );
XAmYammale = (1 - kXYmm) (XAmfs Yamms + Yamfs XAmms );
XamYAmmale = (1 - kXYmm) (Xamfs YAmms + YAmfs Xamms );
XamYammale = (1 - kXYmm) (Xamfs Yamms + Yamfs Xamms);

(*Ym-Ym females*)
YAmYAmfemale = kYYmm (YAmfs YAmms);
YAmYamfemale = kYYmm (YAmfs Yamms + Yamfs YAmms);
YamYamfemale = kYYmm (Yamfs Yamms);
(*Ym-Ym males*)
YAmYAmmale = (1 - kYYmm) (YAmfs YAmms);
YAmYammale = (1 - kYYmm) (YAmfs Yamms + Yamfs YAmms);
YamYammale = (1 - kYYmm) (Yamfs Yamms);

(*DIPLOID SELECTION*)

(*mean male fitness*)
wbarM =
 MAA XAMXAMmale + MAa XAMXaMmale + Maa XaMXaMmale +
  MAA XAMYAMmale + MAa (XAMYaMmale + XaMYAMmale) + Maa XaMYaMmale +
  MAA YAMYAMmale + MAa YAMYaMmale + Maa YaMYaMmale +
  MAA XAmXAMmale + MAa (XAmXaMmale + XAMXammale) + Maa XamXaMmale +
  MAA XAmYAMmale + MAa (XAmYaMmale + XamYAMmale) + Maa XamYaMmale +
  MAA XAMYAmmale + MAa (XAMYammale + XaMYAmmale) + Maa XaMYammale +
  MAA YAmYAMmale + MAa (YAmYaMmale + YamYAMmale) + Maa YamYaMmale +
  MAA XAmXAmmale + MAa XAmXammale + Maa XamXammale +
  MAA XAmYAmmale + MAa (XAmYammale + XamYAmmale) + Maa XamYammale +
  MAA YAmYAmmale + MAa YAmYammale + Maa YamYammale;

XAMXAMmales = MAA XAMXAMmale / wbarM;
XAMXaMmales = MAa XAMXaMmale / wbarM;
XaMXaMmales = Maa XaMXaMmale / wbarM;

XAMYAMmales = MAA XAMYAMmale / wbarM;
XAMYaMmales = MAa XAMYaMmale / wbarM;
XaMYAMmales = MAa XaMYAMmale / wbarM;
XaMYaMmales = Maa XaMYaMmale / wbarM;

YAMYAMmales = MAA YAMYAMmale / wbarM;
YAMYaMmales = MAa YAMYaMmale / wbarM;
YaMYaMmales = Maa YaMYaMmale / wbarM;

XAmXAMmales = MAA XAmXAMmale / wbarM;
XAmXaMmales = MAa XAmXaMmale / wbarM;
XAMXammales = MAa XAMXammale / wbarM;
XamXaMmales = Maa XamXaMmale / wbarM;

XAmYAMmales = MAA XAmYAMmale / wbarM;
```

```
XAmYaMmales = MAa XAmYaMmale / wbarM;
XamYAMmales = MAa XamYAMmale / wbarM;
XamYaMmales = Maa XamYaMmale / wbarM;

XAMYAmmales = MAA XAMYAmmale / wbarM;
XAMYammales = MAa XAMYammale / wbarM;
XaMYAmmales = MAa XaMYAmmale / wbarM;
XaMYammales = Maa XaMYammale / wbarM;

YAmYAMmales = MAA YAmYAMmale / wbarM;
YAmYaMmales = MAa YAmYaMmale / wbarM;
YamYAMmales = MAa YamYAMmale / wbarM;
YamYaMmales = Maa YamYaMmale / wbarM;

XAmXAmmales = MAA XAmXAmmale / wbarM;
XAmXammales = MAa XAmXammale / wbarM;
XamXammales = Maa XamXammale / wbarM;

XAmYAmmales = MAA XAmYAmmale / wbarM;
XAmYammales = MAa XAmYammale / wbarM;
XamYAmmales = MAa XamYAmmale / wbarM;
XamYammales = Maa XamYammale / wbarM;

YAmYAmmales = MAA YAmYAmmale / wbarM;
YAmYammales = MAa YAmYammale / wbarM;
YamYammales = Maa YamYammale / wbarM;

(*mean female fitness*)
wbarF = FAA XAMXAMfemale + FAa XAMXaMfemale + Faa XaMXaMfemale +
   FAA XAMYAMfemale + FAa (XAMYaMfemale + XaMYAMfemale) + Faa XaMYaMfemale +
   FAA YAMYAMfemale + FAa YAMYaMfemale + Faa YaMYaMfemale +
   FAA XAmXAMfemale + FAa XAmXaMfemale + FAa XAMXamfemale + Faa XamXaMfemale +
   FAA XAmYAMfemale + FAa XAmYaMfemale + FAa XamYAMfemale + Faa XamYaMfemale +
   FAA XAMYAmfemale + FAa XAMYamfemale + FAa XaMYAmfemale + Faa XaMYamfemale +
   FAA YAmYAMfemale + FAa YAmYaMfemale + FAa YamYAMfemale + Faa YamYaMfemale +
   FAA XAmXAmfemale + FAa XAmXamfemale + Faa XamXamfemale +
   FAA XAmYAmfemale + FAa XAmYamfemale + FAa XamYAmfemale + Faa XamYamfemale +
   FAA YAmYAmfemale + FAa YAmYamfemale + Faa YamYamfemale;

XAMXAMfemales = FAA XAMXAMfemale / wbarF;
XAMXaMfemales = FAa XAMXaMfemale / wbarF;
XaMXaMfemales = Faa XaMXaMfemale / wbarF;

XAMYAMfemales = FAA XAMYAMfemale / wbarF;
XAMYaMfemales = FAa XAMYaMfemale / wbarF;
XaMYAMfemales = FAa XaMYAMfemale / wbarF;
XaMYaMfemales = Faa XaMYaMfemale / wbarF;

YAMYAMfemales = FAA YAMYAMfemale / wbarF;
YAMYaMfemales = FAa YAMYaMfemale / wbarF;
YaMYaMfemales = Faa YaMYaMfemale / wbarF;

XAmXAMfemales = FAA XAmXAMfemale / wbarF;
XAmXaMfemales = FAa XAmXaMfemale / wbarF;
XAMXamfemales = FAa XAMXamfemale / wbarF;
```

```
XamXaMfemales = Faa XamXaMfemale / wbarF;

XAmYAMfemales = FAA XAmYAMfemale / wbarF;
XAmYaMfemales = FAa XAmYaMfemale / wbarF;
XamYAMfemales = FAa XamYAMfemale / wbarF;
XamYaMfemales = Faa XamYaMfemale / wbarF;

XAMYAmfemales = FAA XAMYAmfemale / wbarF;
XAMYamfemales = FAa XAMYamfemale / wbarF;
XaMYAmfemales = FAa XaMYAmfemale / wbarF;
XaMYamfemales = Faa XaMYamfemale / wbarF;

YAmYAMfemales = FAA YAmYAMfemale / wbarF;
YAmYaMfemales = FAa YAmYaMfemale / wbarF;
YamYAMfemales = FAa YamYAMfemale / wbarF;
YamYaMfemales = Faa YamYaMfemale / wbarF;

XAmXAmfemales = FAA XAmXAmfemale / wbarF;
XAmXamfemales = FAa XAmXamfemale / wbarF;
XamXamfemales = Faa XamXamfemale / wbarF;

XAmYAmfemales = FAA XAmYAmfemale / wbarF;
XAmYamfemales = FAa XAmYamfemale / wbarF;
XamYAmfemales = FAa XamYAmfemale / wbarF;
XamYamfemales = Faa XamYamfemale / wbarF;

YAmYAmfemales = FAA YAmYAmfemale / wbarF;
YAmYamfemales = FAa YAmYamfemale / wbarF;
YamYamfemales = Faa YamYamfemale / wbarF;

(*MEIOSIS*)

xx11ms = XAMXAMmales;
xx12ms = XAMXaMmales;
xx22ms = XaMXaMmales;

xx13ms = XAmXAMmales;
xx23ms = XAmXaMmales;
xx14ms = XAMXammales;
xx24ms = XamXaMmales;

xx33ms = XAmXAmmales;
xx34ms = XAmXammales;
xx44ms = XamXammales;

xy11ms = XAMYAMmales;
xy12ms = XAMYaMmales;
xy21ms = XaMYAMmales;
xy22ms = XaMYaMmales;

xy31ms = XAmYAMmales;
xy32ms = XAmYaMmales;
xy41ms = XamYAMmales;
xy42ms = XamYaMmales;
```

```
xy13ms = XAMYAmmales;
xy14ms = XAMYammales;
xy23ms = XaMYAmmales;
xy24ms = XaMYammales;

xy33ms = XAmYAmmales;
xy34ms = XAmYammales;
xy43ms = XamYAmmales;
xy44ms = XamYammales;

yy11ms = YAMYAMmales;
yy12ms = YAMYaMmales;
yy22ms = YaMYaMmales;

yy13ms = YAmYAMmales;
yy23ms = YAmYaMmales;
yy14ms = YamYAMmales;
yy24ms = YamYaMmales;

yy33ms = YAmYAmmales;
yy34ms = YAmYammales;
yy44ms = YamYammales;

xx11fs = XAMXAMfemales;
xx12fs = XAMXaMfemales;
xx22fs = XaMXaMfemales;

xx13fs = XAmXAMfemales;
xx23fs = XAmXaMfemales;
xx14fs = XAMXamfemales;
xx24fs = XamXaMfemales;

xx33fs = XAmXAmfemales;
xx34fs = XAmXamfemales;
xx44fs = XamXamfemales;

xy11fs = XAMYAMfemales;
xy12fs = XAMYaMfemales;
xy21fs = XaMYAMfemales;
xy22fs = XaMYaMfemales;

xy31fs = XAmYAMfemales;
xy32fs = XAmYaMfemales;
xy41fs = XamYAMfemales;
xy42fs = XamYaMfemales;

xy13fs = XAMYAmfemales;
xy14fs = XAMYamfemales;
xy23fs = XaMYAmfemales;
xy24fs = XaMYamfemales;

xy33fs = XAmYAmfemales;
xy34fs = XAmYamfemales;
xy43fs = XamYAmfemales;
xy44fs = XamYamfemales;
```

Printed by Wolfram Mathematica Student Edition

```
yy11fs = YAMYAMfemales;
yy12fs = YAMYaMfemales;
yy22fs = YaMYaMfemales;

yy13fs = YAmYAMfemales;
yy23fs = YAmYaMfemales;
yy14fs = YamYAMfemales;
yy24fs = YamYaMfemales;

yy33fs = YAmYAmfemales;
yy34fs = YAmYamfemales;
yy44fs = YamYamfemales;

(*X bearing ovules*)
nextXAMf = xx11fs + xx13fs / 2 + (xx12fs + xx14fs * (1 - R) + xx23fs * R) αf +
  xy11fs / 2 + (xy12fs (1 - r) + xy21fs (r)) αf +
  (xy13fs (1 - ρ) + xy31fs (ρ)) / 2 +
  xy14fs * αf +
  (xy14fs (- (R + r + ρ)) +
     xy41fs (r + ρ - R) +
     xy23fs (R + r - ρ) +
     xy32fs (R + ρ - r)) αf / 2;

nextXaMf = xx22fs + xx24fs / 2 + (xx12fs + xx23fs * (1 - R) + xx14fs * R) (1 - αf) +
  xy22fs / 2 + (xy12fs (r) + xy21fs (1 - r)) (1 - αf) +
  (xy24fs (1 - ρ) + xy42fs (ρ)) / 2 +
  xy23fs * (1 - αf) +
  (xy14fs (R + r - ρ) +
     xy41fs (R + ρ - r) +
     xy23fs (- (R + r + ρ)) +
     xy32fs (r + ρ - R)) (1 - αf) / 2;

nextXAmf = xx33fs + xx13fs / 2 + (xx23fs * (1 - R) + R * xx14fs + xx34fs) αf +
  xy33fs / 2 + (xy34fs (1 - r) + xy43fs (r)) αf +
  (xy13fs (ρ) + xy31fs (1 - ρ)) / 2 +
  xy32fs * αf +
  (xy14fs (R + ρ - r) +
     xy41fs (R + r - ρ) +
     xy23fs (r + ρ - R) +
     xy32fs (- (R + r + ρ))) αf / 2;

nextXamf = xx44fs + xx24fs / 2 + (xx14fs * (1 - R) + xx23fs * R + xx34fs) (1 - αf) +
  xy44fs / 2 + (xy34fs (r) + xy43fs (1 - r)) (1 - αf) +
  (xy24fs (ρ) + xy42fs (1 - ρ)) / 2 +
  xy41fs * (1 - αf) +
  (xy14fs (r + ρ - R) +
     xy41fs (- (R + r + ρ)) +
     xy23fs (R + ρ - r) +
     xy32fs (R + r - ρ)) (1 - αf) / 2;

(*Y bearing ovules*)
nextYAMf = yy11fs + yy13fs / 2 + (yy12fs + yy14fs * (1 - R) + yy23fs * R) αf +
  xy11fs / 2 + (xy12fs (r) + xy21fs (1 - r)) (αf) +
  (xy13fs (ρ) + xy31fs (1 - ρ)) / 2 +
```

```
  xy41fs * αf +
  (xy14fs (r + ρ - R) +
      xy41fs (- (R + r + ρ)) +
     xy23fs (R + ρ - r) +
     xy32fs (R + r - ρ)) αf / 2;

nextYaMf = yy22fs + yy24fs / 2 + (yy12fs + yy23fs * (1 - R) + yy14fs * R) (1 - αf) +
  xy22fs / 2 + (xy12fs (1 - r) + xy21fs (r)) (1 - αf) +
  (xy24fs (ρ) + xy42fs (1 - ρ)) / 2 +
  xy32fs * (1 - αf) +
  (xy14fs (R + ρ - r) +
     xy41fs (R + r - ρ) +
     xy23fs (r + ρ - R) +
     xy32fs (- (R + r + ρ))) (1 - αf) / 2;

nextYAmf = yy33fs + yy13fs / 2 + (yy23fs * (1 - R) + R * yy14fs + yy34fs) αf +
  xy33fs / 2 + (xy34fs (r) + xy43fs (1 - r)) (αf) +
  (xy13fs (1 - ρ) + xy31fs (ρ)) / 2 +
  xy23fs * αf +
  (xy14fs (R + r - ρ) +
     xy41fs (R + ρ - r) +
     xy23fs (- (R + r + ρ)) +
     xy32fs (r + ρ - R)) αf / 2;

nextYamf = yy44fs + yy24fs / 2 + (yy14fs * (1 - R) + yy23fs * R + yy34fs) (1 - αf) +
  xy44fs / 2 + (xy34fs (1 - r) + xy43fs (r)) (1 - αf) +
  (xy24fs (1 - ρ) + xy42fs (ρ)) / 2 +
  xy14fs * (1 - αf) +
  (xy14fs (- (R + r + ρ)) +
     xy41fs (r + ρ - R) +
     xy23fs (R + r - ρ) +
     xy32fs (R + ρ - r)) (1 - αf) / 2;

(*X bearing pollen*)
nextXAMm = xx11ms + xx13ms / 2 + (xx12ms + xx14ms * (1 - R) + xx23ms * R) αm +
  xy11ms / 2 + (xy12ms (1 - r) + xy21ms (r)) αm +
  (xy13ms (1 - ρ) + xy31ms (ρ)) / 2 +
  xy14ms * αm +
  (xy14ms (- (R + r + ρ)) +
     xy41ms (r + ρ - R) +
     xy23ms (R + r - ρ) +
     xy32ms (R + ρ - r)) αm / 2;

nextXaMm = xx22ms + xx24ms / 2 + (xx12ms + xx23ms * (1 - R) + xx14ms * R) (1 - αm) +
  xy22ms / 2 + (xy12ms (r) + xy21ms (1 - r)) (1 - αm) +
  (xy24ms (1 - ρ) + xy42ms (ρ)) / 2 +
  xy23ms * (1 - αm) +
  (xy14ms (R + r - ρ) +
     xy41ms (R + ρ - r) +
     xy23ms (- (R + r + ρ)) +
     xy32ms (r + ρ - R)) (1 - αm) / 2;

nextXAmm = xx33ms + xx13ms / 2 + (xx23ms * (1 - R) + R * xx14ms + xx34ms) αm +
  xy33ms / 2 + (xy34ms (1 - r) + xy43ms (r)) αm +
  (xy13ms (ρ) + xy31ms (1 - ρ)) / 2 +
```

```
    xy32ms * αm +
    (xy14ms (R + ρ - r) +
       xy41ms (R + r - ρ) +
       xy23ms (r + ρ - R) +
       xy32ms (- (R + r + ρ))) αm / 2;

  nextXamm = xx44ms + xx24ms / 2 + (xx14ms * (1 - R) + xx23ms * R + xx34ms) (1 - αm) +
    xy44ms / 2 + (xy34ms (r) + xy43ms (1 - r)) (1 - αm) +
    (xy24ms (ρ) + xy42ms (1 - ρ)) / 2 +
    xy41ms * (1 - αm) +
    (xy14ms (r + ρ - R) +
       xy41ms (- (R + r + ρ)) +
       xy23ms (R + ρ - r) +
       xy32ms (R + r - ρ)) (1 - αm) / 2;

  (*Y bearing pollen*)
  nextYAMm = yy11ms + yy13ms / 2 + (yy12ms + yy14ms * (1 - R) + yy23ms * R) αm +
    xy11ms / 2 + (xy12ms (r) + xy21ms (1 - r)) (αm) +
    (xy13ms (ρ) + xy31ms (1 - ρ)) / 2 +
    xy41ms * αm +
    (xy14ms (r + ρ - R) +
       xy41ms (- (R + r + ρ)) +
       xy23ms (R + ρ - r) +
       xy32ms (R + r - ρ)) αm / 2;

  nextYaMm = yy22ms + yy24ms / 2 + (yy12ms + yy23ms * (1 - R) + yy14ms * R) (1 - αm) +
    xy22ms / 2 + (xy12ms (1 - r) + xy21ms (r)) (1 - αm) +
    (xy24ms (ρ) + xy42ms (1 - ρ)) / 2 +
    xy32ms * (1 - αm) +
    (xy14ms (R + ρ - r) +
       xy41ms (R + r - ρ) +
       xy23ms (r + ρ - R) +
       xy32ms (- (R + r + ρ))) (1 - αm) / 2;

  nextYAmm = yy33ms + yy13ms / 2 + (yy23ms * (1 - R) + R * yy14ms + yy34ms) αm +
    xy33ms / 2 + (xy34ms (r) + xy43ms (1 - r)) (αm) +
    (xy13ms (1 - ρ) + xy31ms (ρ)) / 2 +
    xy23ms * αm +
    (xy14ms (R + r - ρ) +
       xy41ms (R + ρ - r) +
       xy23ms (- (R + r + ρ)) +
       xy32ms (r + ρ - R)) αm / 2;

  nextYamm = yy44ms + yy24ms / 2 + (yy14ms * (1 - R) + yy23ms * R + yy34ms) (1 - αm) +
    xy44ms / 2 + (xy34ms (1 - r) + xy43ms (r)) (1 - αm) +
    (xy24ms (1 - ρ) + xy42ms (ρ)) / 2 +
    xy14ms * (1 - αm) +
    (xy14ms (- (R + r + ρ)) +
       xy41ms (r + ρ - R) +
       xy23ms (R + r - ρ) +
       xy32ms (R + ρ - r)) (1 - αm) / 2;

  {nextXAMf, nextXaMf, nextXAmf, nextXamf, nextYAMf, nextYaMf, nextYAmf, nextYamf,
   nextXAMm, nextXaMm, nextXAmm, nextXamm, nextYAMm, nextYaMm, nextYAmm, nextYamm}
]
```

Sex ratio

Fraction males among zygotes

```
(*fraction female*)
(*XAMXAMfemale+XAMXaMfemale+XaMXaMfemale+
   XAMYAMfemale+XAMYaMfemale+XaMYAMfemale+XaMYaMfemale+
   YAMYAMfemale+YAMYaMfemale+YaMYaMfemale+
   XAmXAMfemale+XAmXaMfemale+XAMXamfemale+XamXaMfemale+
   XAmYAMfemale+XAmYaMfemale+XamYAMfemale+XamYaMfemale+
   XAMYAmfemale+XAMYamfemale+XaMYAmfemale+XaMYamfemale+
   YAmYAMfemale+YAmYaMfemale+YamYAMfemale+YamYaMfemale+
   XAmXAmfemale+XAmXamfemale+XamXamfemale+
   XAmYAmfemale+XAmYamfemale+XamYAmfemale+XamYamfemale+
   YAmYAmfemale+YAmYamfemale+YamYamfemale/.SUBS//Factor*)
```

```
Clear[sexratio]
sexratio[{FAA_, FAa_, Faa_, MAA_, MAa_, Maa_, wAf_, waf_, wAm_, wam_, αf_,
    αm_, r_, R_, ρ_, k_}, {XAMf_, XaMf_, XAmf_, Xamf_, YAMf_, YaMf_, YAmf_,
    Yamf_, XAMm_, XaMm_, XAmm_, Xamm_, YAMm_, YaMm_, YAmm_, Yamm_}] :=
  sexratio[{FAA, FAa, Faa, MAA, MAa, Maa, wAf, waf, wAm, wam, αf, αm, r, R, ρ, k},
    {XAMf, XaMf, XAmf, Xamf, YAMf, YaMf, YAmf, Yamf,
     XAMm, XaMm, XAmm, Xamm, YAMm, YaMm, YAmm, Yamm}] =
   1 - (k waf wam Xamf Xamm + k waf wam XaMf Xamm + k wAf wam XAmf Xamm + k wAf wam XAMf Xamm +
        k waf wam Xamf XaMm + waf wam XaMf XaMm + k wAf wam XAmf XaMm + wAf wam XAMf XaMm +
        k waf wAm Xamf XAmm + k waf wAm XaMf XAmm + k wAf wAm XAmf XAmm +
        k wAf wAm XAMf XAmm + k waf wAm Xamf XAMm + waf wAm XaMf XAMm + k wAf wAm XAmf XAMm +
        wAf wAm XAMf XAMm + k waf wam Xamm Yamf + k waf wam XaMm Yamf + k waf wAm XAmm Yamf +
        k waf wAm XAMm Yamf + k waf wam Xamm YaMf + k waf wAm XAmm YaMf + k wAf wam Xamm YAmf +
        k wAf wam XaMm YAmf + k wAf wAm XAmm YAmf + k wAf wAm XAMm YAmf + k wAf wam Xamm YAMf +
        k wAf wAm XAmm YAMf + k waf wam Xamf Yamm + k waf wam XaMf Yamm + k wAf wam XAmf Yamm +
        k wAf wam XAMf Yamm + k waf wam Yamf Yamm + k waf wam YaMf Yamm + k wAf wam YAmf Yamm +
        k wAf wam YAMf Yamm + k waf wam Xamf YaMm + k wAf wam XAmf YaMm + k waf wam Yamf YaMm +
        k wAf wam YAmf YaMm + k waf wAm Xamf YAmm + k waf wAm XaMf YAmm + k wAf wAm XAmf YAmm +
        k wAf wAm XAMf YAmm + k waf wAm Yamf YAmm + k waf wAm YaMf YAmm +
        k wAf wAm YAmf YAmm + k wAf wAm YAMf YAmm + k waf wAm Xamf YAMm +
        k wAf wAm XAmf YAMm + k waf wAm Yamf YAMm + k wAf wAm YAmf YAMm) /
     ((waf Xamf + waf XaMf + wAf XAmf + wAf XAMf + waf Yamf + waf YaMf + wAf YAmf + wAf YAMf)
       (wam Xamm + wam XaMm + wAm XAmm + wAm XAMm +
         wam Yamm + wam YaMm + wAm YAmm + wAm YAMm));
```

Figure 2 - region plot of neo-W haplotype invasion with tight linkage and no haploid selection

Plotting parameters

```
xmin = 0;
xmax = 1.5;
xtickmin = 0;
xtickmax = 1;
xint = 1;
ymin = xmin;
ymax = xmax;
ytickmin = xtickmin;
ytickmax = xtickmax;
yint = xint;
```

Panel A - A favoured in females

Parameters

```
params = {
    wAm → 1, wam → 1, wAf → 1, waf → 1,
    αm → 1 / 2, αf → 1 / 2,
    MAa → 1, FAa → 1,
    Faa → 0.85,
    FAA → 1.05
  };
```

No recombination eigenvalues

```
WAinvA = λmA1 /. reverse /. pAveM → (1 - q) pXm + q pYm /. equilA0 /. params // Simplify;
WainvA = λma1 /. reverse /. pAveM → (1 - q) pXm + q pYm /. equilA0 /. params // Simplify;
WAinvB = λmA1 /. reverse /. pAveM → (1 - q) pXm + q pYm /. equilB0 /. params // Simplify;
WainvB = λma1 /. reverse /. pAveM → (1 - q) pXm + q pYm /. equilB0 /. params // Simplify;
```

Maximum absolute no recombination eigenvalue from the full characteristic polynomial

```
λWsolA =
  Max[Abs[λ /. Solve[0 == charpolyk1 /. r → 0 /. R → 0 /. ρ → 0 /. equilA0 /. params, λ] //
      Simplify]];
λWsolB = Max[Abs[λ /. Solve[0 == charpolyk1 /. r → 0 /. R → 0 /. ρ → 0 /. equilB0 /.
         params, λ] // Simplify]];
```

Plot

Region plots of invasion

```
(*neo-WA invades XY from equilA*)
plotWAinvA =
  RegionPlot[{
    (validcondA /. params) && (*valid*)
     (stabcondA /. Rf → 0 /. Rm → 0 /. equilA0 /. params) && (*internally stable*)
```

```
      1 < WAinvA (*invasion*)
    },
    {Maa, 0, 3 / 2}, {MAA, 0, 3 / 2},
    PlotStyle → {Gray, Opacity[0.5]},
    BoundaryStyle → None
   ];

(*neo-Wa invades XY from equilA*)
plotWainvA =
  RegionPlot[{
     (validcondA /. params) &&
      (stabcondA /. Rf → 0 /. Rm → 0 /. equilA0 /. params) &&
      1 < WainvA
    },
    {Maa, 0, 3 / 2}, {MAA, 0, 3 / 2},
    PlotStyle → {Gray, Opacity[0.5]},
    BoundaryStyle → None
   ];

(*neo-WA invades XY from equilB*)
plotWAinvB =
  RegionPlot[{
     (stabcondB /. Rf → 0 /. Rm → 0 /. equilB0 /. params) && (*internally stable*)
      1 < WAinvB(*invasion*)
    },
    {Maa, 0, 3 / 2}, {MAA, 0, 3 / 2},
    PlotStyle → {Gray, Opacity[0.5]},
    BoundaryStyle → None
   ];

(*neo-Wa invades XY from equilB*)
plotWainvB =
  RegionPlot[{
     (stabcondB /. Rf → 0 /. Rm → 0 /. equilB0 /. params) &&
      1 < WainvB
    },
    {Maa, 0, 3 / 2}, {MAA, 0, 3 / 2},
    PlotStyle → {Gray, Opacity[0.5]},
    BoundaryStyle → None
   ];

(*Ya equilibrium internally stable*)
plotYaStable =
  RegionPlot[{
     (stabcondA /. Rf → 0 /. Rm → 0 /. equilA0 /. params) ||
      (stabcondB /. Rf → 0 /. Rm → 0 /. equilB0 /. params)
    },
    {Maa, 0, 3 / 2}, {MAA, 0, 3 / 2},
    PlotStyle → White,
    BoundaryStyle → {Black, Thick}
   ];

(*Ya equilibrium internally unstable*)
plotYaUnstable =
  RegionPlot[{
```

```
      1 < 2
     },
     {Maa, 0, 3 / 2}, {MAA, 0, 3 / 2},
     PlotStyle → None,
     BoundaryStyle → None,
     Mesh → 50,
     MeshFunctions → {#1 - #2 &},
     MeshStyle → Black
    ];

plotA =
  Show[

   plotYaUnstable,
   plotYaStable,
   plotWAinvA,
   plotWainvA,
   plotWAinvB,
   plotWainvB,

   FrameLabel → {"Relative fitness of aa males", ""},
   Epilog → {
     Text[Style["A", Bold], Scaled@{0.05, 0.95}],
     Text[Style["Λ_{W'A}^{(XY)}>1"], {1.29, 0.5}],
     Text[Style["Λ_{W'A}^{(XY)}<1 & Λ_{W'a}^{(XY)}<1"], {0.5, 0.5}],
     Text[Style["Y-a equilibrium unstable", Bold],
       {0.5, 1.25}, Background → White],
     Text[Style["*", Bold], {1.2, 0.85}],
     Rotate[Text[
        Style["Relative fitness of AA males"], Scaled@ylabposregion], 90 Degree],
     Text[Style["A favoured in females", Bold], Scaled@{0.5, 1.05}]
    },

   regionplotstyle[xmin, xmax, xtickmin,
    xtickmax, xint, ymin, ymax, ytickmin, ytickmax, yint]
  ]
```

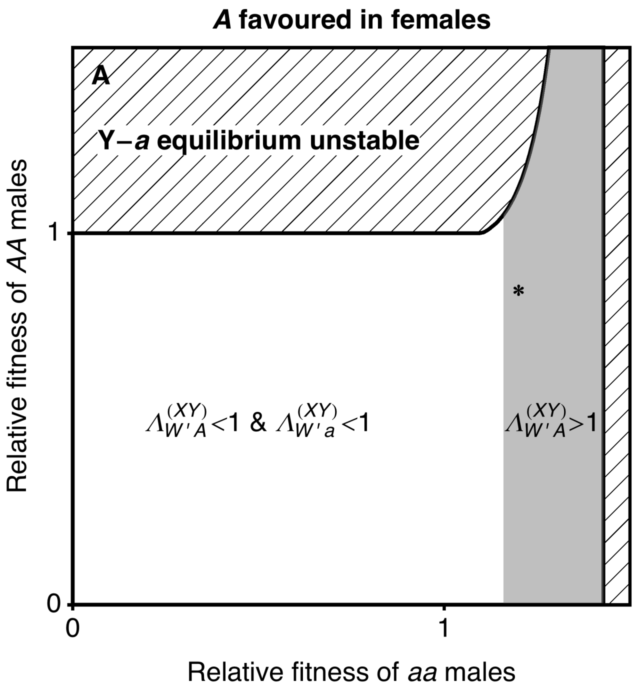

Notice that this is consistent with the solution from the full characteristic polynomial (but here we don’t know which eigenvalue belongs to which haplotype)

```
(*neo-W invades XY from equilA*)
plotWinvA =
  RegionPlot[{
    (validcondA /. params) && (*valid*)
     (stabcondA /. Rf → 0 /. Rm → 0 /. equilA0 /. params) && (*internally stable*)
     1 < λWsolA (*invasion*)
   },
   {Maa, 0, 3 / 2}, {MAA, 0, 3 / 2},
   PlotStyle → {Gray, Opacity[0.5]},
   BoundaryStyle → None
  ];

(*neo-W invades XY from equilB*)
plotWinvB =
  RegionPlot[{
    (stabcondB /. Rf → 0 /. Rm → 0 /. equilB0 /. params) && (*internally stable*)
     1 < λWsolB(*invasion*)
   },
   {Maa, 0, 3 / 2}, {MAA, 0, 3 / 2},
   PlotStyle → {Gray, Opacity[0.5]},
   BoundaryStyle → None
  ];
```

```
Show[

 plotWinvA,
 plotWinvB,
 plotYaStable,

 regionplotstyle[xmin, xmax, xtickmin,
  xtickmax, xint, ymin, ymax, ytickmin, ytickmax, yint]

]
```

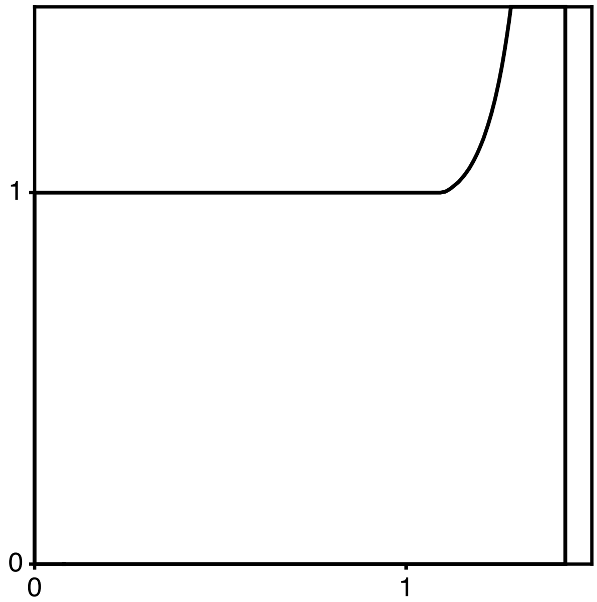

Panel B - a favoured in females

Parameters

```
params = {
   wAm → 1, wam → 1, wAf → 1, waf → 1,
   αm → 1 / 2, αf → 1 / 2,
   MAa → 1, FAa → 1,
   Faa → 1.05,
   FAA → 0.85
  };
```

No recombination eigenvalues

```
WAinvA = λmA1 /. reverse /. pAveM → (1 - q) pXm + q pYm /. equilA0 /. params // Simplify;
WainvA = λma1 /. reverse /. pAveM → (1 - q) pXm + q pYm /. equilA0 /. params // Simplify;
WAinvB = λmA1 /. reverse /. pAveM → (1 - q) pXm + q pYm /. equilB0 /. params // Simplify;
WainvB = λma1 /. reverse /. pAveM → (1 - q) pXm + q pYm /. equilB0 /. params // Simplify;
```

Maximum absolute no recombination eigenvalue from the full characteristic polynomial

```
λWsolA =
  Max[Abs[λ /. Solve[0 == charpolyExt /. k → 1 /. r → 0 /. R → 0 /. ρ → 0 /. equilA0 /.
        params, λ] // Simplify]];
λWsolB = Max[Abs[λ /. Solve[0 == charpolyExt /. k → 1 /. r → 0 /. R → 0 /. ρ → 0 /.
        equilB0 /. params, λ] // Simplify]];
```

Solve::ratnz : Solve was unable to solve the system with inexact coefficients. The
answer was obtained by solving a corresponding exact system and numericizing the result. ≫

Plot

Region plots of invasion

```
(*neo-WA invades XY from equilA*)
plotWAinvA =
  RegionPlot[{
    (validcondA /. params) && (*valid*)
     (stabcondA /. Rf → 0 /. Rm → 0 /. equilA0 /. params) && (*internally stable*)
     1 < WAinvA (*invasion*)
   },
   {Maa, 0, 3 / 2}, {MAA, 0, 3 / 2},
   PlotStyle → {Gray, Opacity[0.5]},
   BoundaryStyle → None
  ];

(*neo-Wa invades XY from equilA*)
plotWainvA =
  RegionPlot[{
    (validcondA /. params) &&
     (stabcondA /. Rf → 0 /. Rm → 0 /. equilA0 /. params) &&
     1 < WainvA
   },
   {Maa, 0, 3 / 2}, {MAA, 0, 3 / 2},
   PlotStyle → {Gray, Opacity[0.5]},
   BoundaryStyle → None
  ];

(*neo-WA invades XY from equilB*)
plotWAinvB =
  RegionPlot[{
    (stabcondB /. Rf → 0 /. Rm → 0 /. equilB0 /. params) && (*internally stable*)
     1 < WAinvB(*invasion*)
   },
   {Maa, 0, 3 / 2}, {MAA, 0, 3 / 2},
   PlotStyle → {Gray, Opacity[0.5]},
   BoundaryStyle → None
  ];

(*neo-Wa invades XY from equilB*)
plotWainvB =
  RegionPlot[{
    (stabcondB /. Rf → 0 /. Rm → 0 /. equilB0 /. params) &&
     1 < WainvB
   },
   {Maa, 0, 3 / 2}, {MAA, 0, 3 / 2},
```

```
    PlotStyle → {Gray, Opacity[0.5]},
    BoundaryStyle → None
   ];

(*Ya equilibrium internally stable*)
plotYaStable =
  RegionPlot[{
     (stabcondA /. Rf → 0 /. Rm → 0 /. equilA0 /. params) ||
      (stabcondB /. Rf → 0 /. Rm → 0 /. equilB0 /. params)
    },
    {Maa, 0, 3 / 2}, {MAA, 0, 3 / 2},
    PlotStyle → White,
    BoundaryStyle → {Black, Thick}
   ];

(*Ya equilibrium internally unstable*)
plotYaUnstable =
  RegionPlot[{
     1 < 2
    },
    {Maa, 0, 3 / 2}, {MAA, 0, 3 / 2},
    PlotStyle → None,
    BoundaryStyle → None,
    Mesh → 50,
    MeshFunctions → {#1 - #2 &},
    MeshStyle → Black
   ];
```

```
plotB =
 Show[

  plotYaUnstable,
  plotYaStable,
  plotWAinvA,
  plotWainvA,
  plotWAinvB,
  plotWainvB,

  Graphics[{Thick, Black, Line[{{0.85, 0.25}, {1, 0.45}}]}],

  FrameLabel → {"Relative fitness of aa males", ""},
  Epilog → {
    Text[Style["B", Bold], Scaled@{0.05, 0.95}],
    Text[Style["Λ(XY)W'a >1"], {1, 0.5}, {-1, 0}, Background → White],
    Text[Style["Λ(XY)W'A >1 & Λ(XY)W'a >1"], {0.4, 0.5}],
    Text[Style["Y-a equilibrium unstable", Bold],
     {0.5, 1.25}, Background → White],
    Text[Style["†", 16, Bold], {0.75, 0.75}],
    Text[Style["a favoured in females", Bold], Scaled@{0.5, 1.05}]
   },

  regionplotstyle[xmin, xmax, xtickmin,
   xtickmax, xint, ymin, ymax, ytickmin, ytickmax, yint]

 ]
```

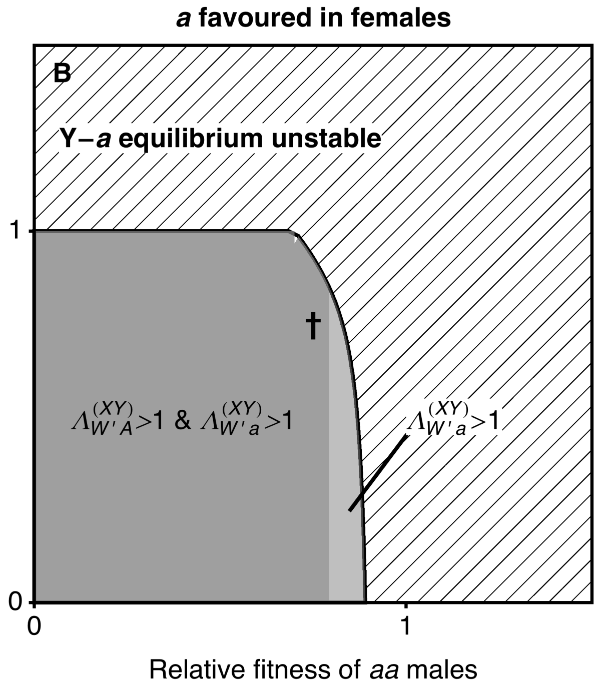

Notice that this is consistent with the solution from the full characteristic polynomial (but here we don't know which eigenvalue belongs to which haplotype)

```
(*neo-W invades XY from equilA*)
plotWinvA =
  RegionPlot[{
     (validcondA /. params) && (*valid*)
      (stabcondA /. Rf → 0 /. Rm → 0 /. equilA0 /. params) && (*internally stable*)
      1 < λWsolA (*invasion*)
    },
    {Maa, 0, 3 / 2}, {MAA, 0, 3 / 2},
    PlotStyle → {Gray, Opacity[0.5]},
    BoundaryStyle → None
  ];

(*neo-W invades XY from equilB*)
plotWinvB =
  RegionPlot[{
     (stabcondB /. Rf → 0 /. Rm → 0 /. equilB0 /. params) && (*internally stable*)
      1 < λWsolB(*invasion*)
    },
    {Maa, 0, 3 / 2}, {MAA, 0, 3 / 2},
    PlotStyle → {Gray, Opacity[0.5]},
    BoundaryStyle → None
  ];
```

```
Show[

 plotWinvA,
 plotWinvB,
 plotYaStable,

 regionplotstyle[xmin, xmax, xtickmin,
  xtickmax, xint, ymin, ymax, ytickmin, ytickmax, yint]

]
```

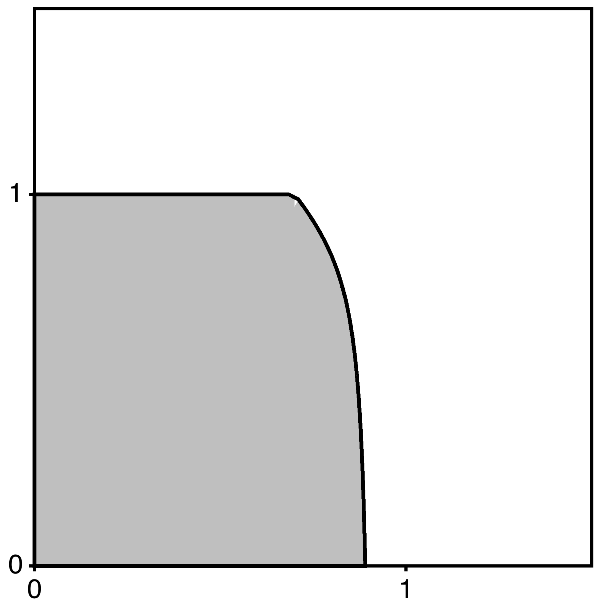

Panel C - overdominance in females

Parameters

```
params = {
   wAm → 1, wam → 1, wAf → 1, waf → 1,
   αm → 1 / 2, αf → 1 / 2,
   MAa → 1, FAa → 1,
   Faa → 0.6,
   FAA → 0.6
  };
```

No recombination eigenvalues

```
WAinvA = λmA1 /. reverse /. pAveM → (1 - q) pXm + q pYm /. equilA0 /. params // Simplify;
WainvA = λma1 /. reverse /. pAveM → (1 - q) pXm + q pYm /. equilA0 /. params // Simplify;
WAinvB = λmA1 /. reverse /. pAveM → (1 - q) pXm + q pYm /. equilB0 /. params // Simplify;
WainvB = λma1 /. reverse /. pAveM → (1 - q) pXm + q pYm /. equilB0 /. params // Simplify;
```

Maximum absolute no recombination eigenvalue from the full characteristic polynomial

```
λWsolA =
  Max[Abs[λ /. Solve[0 == charpolyExt /. k → 1 /. r → 0 /. R → 0 /. ρ → 0 /. equilA0 /.
        params, λ] // Simplify]];
λWsolB = Max[Abs[λ /. Solve[0 == charpolyExt /. k → 1 /. r → 0 /. R → 0 /. ρ → 0 /.
         equilB0 /. params, λ] // Simplify]];
```

Solve::ratnz : Solve was unable to solve the system with inexact coefficients. The answer was obtained by solving a corresponding exact system and numericizing the result. ≫

Plots

Region plots of invasion

```
(*neo-WA invades XY from equilA*)
plotWAinvA =
  RegionPlot[{
    (validcondA /. params) && (*valid*)
     (stabcondA /. Rf → 0 /. Rm → 0 /. equilA0 /. params) && (*internally stable*)
     1 < WAinvA (*invasion*)
   },
   {Maa, 0, 3 / 2}, {MAA, 0, 3 / 2},
   PlotStyle → {Gray, Opacity[0.5]},
   BoundaryStyle → None
  ];

(*neo-Wa invades XY from equilA*)
plotWainvA =
  RegionPlot[{
    (validcondA /. params) &&
     (stabcondA /. Rf → 0 /. Rm → 0 /. equilA0 /. params) &&
     1 < WainvA
   },
   {Maa, 0, 3 / 2}, {MAA, 0, 3 / 2},
   PlotStyle → {Gray, Opacity[0.5]},
   BoundaryStyle → None
  ];

(*neo-WA invades XY from equilB*)
plotWAinvB =
  RegionPlot[{
    (stabcondB /. Rf → 0 /. Rm → 0 /. equilB0 /. params) && (*internally stable*)
     1 < WAinvB(*invasion*)
   },
   {Maa, 0, 3 / 2}, {MAA, 0, 3 / 2},
   PlotStyle → {Gray, Opacity[0.5]},
   BoundaryStyle → None
  ];

(*neo-Wa invades XY from equilB*)
plotWainvB =
  RegionPlot[{
    (stabcondB /. Rf → 0 /. Rm → 0 /. equilB0 /. params) &&
     1 < WainvB
   },
   {Maa, 0, 3 / 2}, {MAA, 0, 3 / 2},
```

```
   PlotStyle → {Gray, Opacity[0.5]},
   BoundaryStyle → None
  ];

(*Ya equilibrium internally stable*)
plotYaStable =
  RegionPlot[{
    (stabcondA /. Rf → 0 /. Rm → 0 /. equilA0 /. params) ||
     (stabcondB /. Rf → 0 /. Rm → 0 /. equilB0 /. params)
   },
   {Maa, 0, 3 / 2}, {MAA, 0, 3 / 2},
   PlotStyle → White,
   BoundaryStyle → {Black, Thick}
  ];

(*Ya equilibrium internally unstable*)
plotYaUnstable =
  RegionPlot[{
    1 < 2
   },
   {Maa, 0, 3 / 2}, {MAA, 0, 3 / 2},
   PlotStyle → None,
   BoundaryStyle → None,
   Mesh → 50,
   MeshFunctions → {#1 - #2 &},
   MeshStyle → Black
  ];
```

```
plotC =
 Show[

  plotYaUnstable,
  plotYaStable,
  plotWAinvA,
  plotWainvA,
  plotWAinvB,
  plotWainvB,

  FrameLabel → {"Relative fitness of aa males", ""},
  Epilog → {
    Text[Style["C", Bold], Scaled@{0.05, 0.95}],
    Text[Style["Λ(XY)W'A >1"], {1, 0.5}],
    Text[Style["Λ(XY)W'A >1 &
Λ(XY)W'a >1"], {0.325, 0.5}],
    Text[Style["Y-a equilibrium unstable", Bold],
     {0.5, 1.25}, Background → White],
    Text[Style["†", Bold], {0.5, 0.7}],
    Text[Style["overdominance in females", Bold], Scaled@{0.5, 1.05}]
   },

  regionplotstyle[xmin, xmax, xtickmin,
   xtickmax, xint, ymin, ymax, ytickmin, ytickmax, yint]

 ]
```

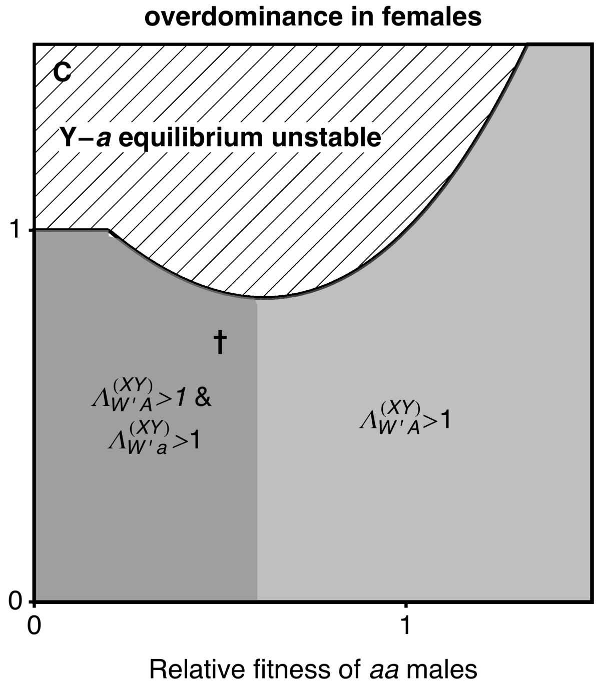

```
? Text
```

Text[*expr*] displays with *expr* in plain text format.
Text[*expr*, *coords*] is a graphics primitive that
displays the textual form of *expr* centered at the point specified by *coords*. >>

Notice that this is consistent with the solution from the full characteristic polynomial (but here we don’t know which eigenvalue belongs to which haplotype)

```
(*neo-W invades XY from equilA*)
plotWinvA =
  RegionPlot[{
    (validcondA /. params) && (*valid*)
     (stabcondA /. Rf → 0 /. Rm → 0 /. equilA0 /. params) && (*internally stable*)
     1 < λWsolA (*invasion*)
   },
   {Maa, 0, 3 / 2}, {MAA, 0, 3 / 2},
   PlotStyle → {Gray, Opacity[0.5]},
   BoundaryStyle → None
  ];

(*neo-W invades XY from equilB*)
plotWinvB =
  RegionPlot[{
    (stabcondB /. Rf → 0 /. Rm → 0 /. equilB0 /. params) && (*internally stable*)
     1 < λWsolB(*invasion*)
   },
   {Maa, 0, 3 / 2}, {MAA, 0, 3 / 2},
   PlotStyle → {Gray, Opacity[0.5]},
   BoundaryStyle → None
  ];
```

```
Show[

 plotWinvA,
 plotWinvB,
 plotYaStable,

 regionplotstyle[xmin, xmax, xtickmin,
  xtickmax, xint, ymin, ymax, ytickmin, ytickmax, yint]

]
```

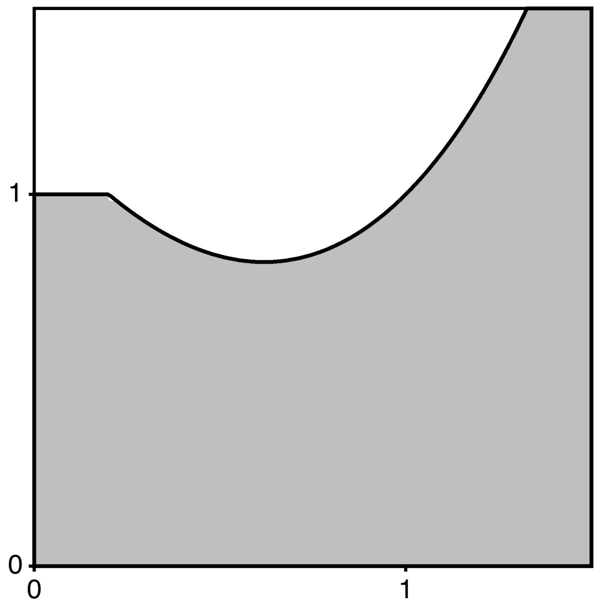

All panels

```
GraphicsRow[
 {plotA, plotB, plotC},
 Spacings → -45
]

Export[plotdir <> "Region_plot_combined.eps", % // rasterTrick];
```

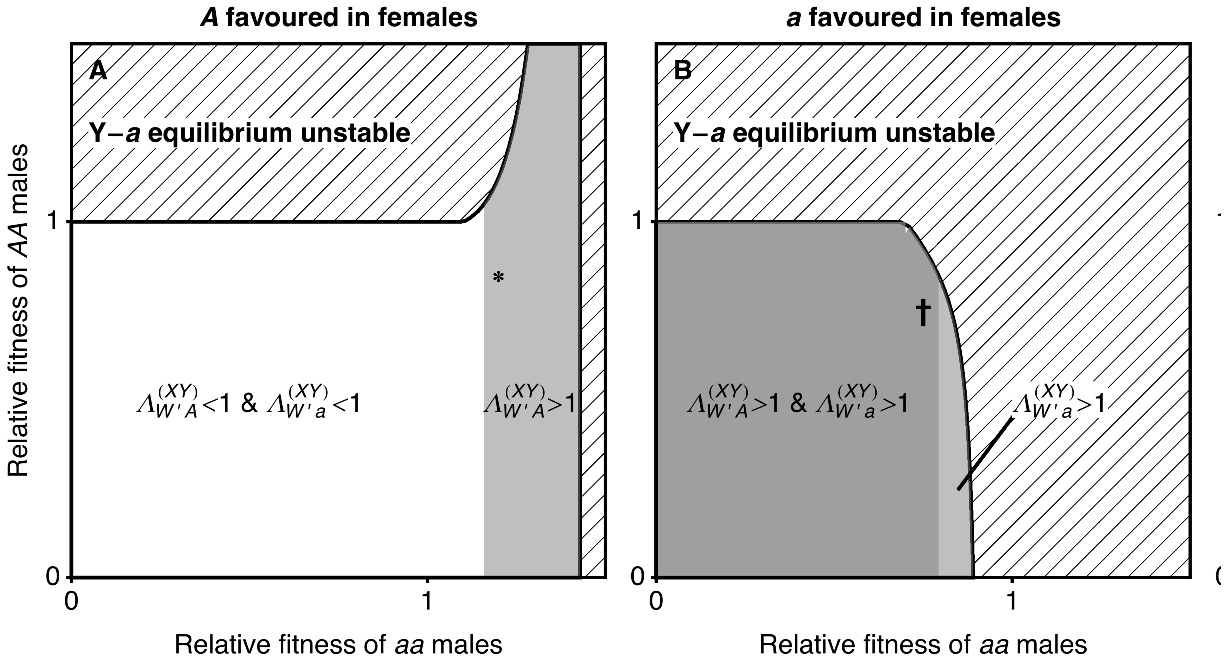

Figure 3 - neo-W invasion with sexual antagonism for loose and tight linkage

Panel A - neo-W invasion fitness as function of selected locus location, with tight linkage

Parameters

```
(*selection parameters*)
tryFAA = 1.05;
tryFAa = 1;
tryFaa = 0.85;
tryMAA = 0.85;
tryMAa = 1;
tryMaa = 1.2;
trywAf = 1;
trywaf = 1;
trywAm = 1;
trywam = 1;
tryαf = 0.5;
tryαm = 0.5;

(*position of X and M loci*)
x = -5;
m = 5;

(*x limits*)
xmin = -10;
xmax = 10;
xtickmin = -10;
xtickmax = 10;
xint  = 5;

npoints = 36;
```

Plot

```
Clear[a]
positionplotA =
 Show[

  (*region of tighter linkage*)
  RegionPlot[
   x > 0,
   {x, xmin, xmax},
   {y, ymin, ymax},
   PlotStyle → LightGray,
   BoundaryStyle → None,
   AxesOrigin → {xmin, ymin},
   Frame → {True, True, False, False}
  ],

  (*neo-W into XY*)
  ListPlot[
   Table[
    {
     a,
     invasionplotXY[tryFAA, tryFAa, tryFaa, tryMAA, tryMAa, tryMaa, trywAf, trywaf,
      trywAm, trywam, tryαf, tryαm, setr[x, a, m], setR[x, a, m], setρ[x, a, m]]
    },
    {a, xmin, xmax, (xmax - xmin) / (npoints)}
   ],
   Joined → True,
   PlotRange → All,
   PlotStyle → Directive[Black, Thickness[lwd]],
   AxesOrigin → {xmin, ymin},
   Frame → {True, True, False, False}
  ],

  Plot[0, {x, xmin, xmax}, PlotStyle → {Black, Dotted}],

  FrameLabel → {"Chromosomal position of selected locus (cM)", ""},
  Epilog → {
    Text[Style["A", Bold], Scaled@letpos],
    Rotate[Text["Invasion fitness (λ_W'^(XY)-1)", Scaled@{-0.15, 0.5}], 90 Degree],
    Text["tighter sex-linkage", {m, ymax * 0.9}],
    Text["(R < r)", {m, ymax * 0.75}],
    Text["ancestral SDR", {x, ymax * 0.5}],
    Text["novel SDR", {m, ymin * 0.75}],
    Arrow[{{x, ymax * 0.4}, {x, 0}}],
    Arrow[{{m, ymin * 0.5}, {m, 0}}],
    Text[Style["*", Bold], {-1, 0.03}]
   },

  plotstyle[xmin, xmax, xtickmin,
   xtickmax, xint, ymin, ymax, ytickmin, ytickmax, yint]

 ]
```

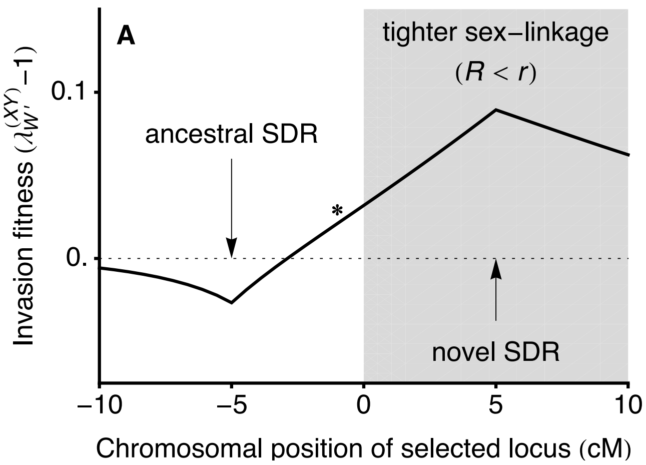
A
tighter sex−linkage
(R < r)
ancestral SDR
*
novel SDR
0.1
0.
−10
−5
0
5
10
Invasion fitness ($\lambda_{W'}^{(XY)}$−1)
Chromosomal position of selected locus (cM)

Panel B - neo-W invasion fitness as function of selected locus location, with loose linkage

Parameters

```
(*selection parameters*)
tryFAA = 1.05;
tryFAa = 1;
tryFaa = 0.85;
tryMAA = 0.85;
tryMAa = 1;
tryMaa = 1.2;
trywAf = 1;
trywaf = 1;
trywAm = 1;
trywam = 1;
tryαf = 0.5;
tryαm = 0.5;

(*position of X and M loci*)
x = -50;
m = 50;

(*x limits*)
xmin = -100;
xmax = 100;
xtickmin = -100;
xtickmax = 100;
xint = 50;

(*y limits*)
ymin = -0.075;
ymax = 0.15;
ytickmin = -0.1;
ytickmax = 0.1;
yint = 0.1;

(*number of data points*)
npoints = 36;
```

Plot

```
Clear[a]
positionplotB =
 Show[

  (*region of tighter linkage*)
  RegionPlot[
   x > 0,
   {x, xmin, xmax},
   {y, ymin, ymax},
   PlotStyle → LightGray,
   BoundaryStyle → None,
   AxesOrigin → {xmin, ymin},
   Frame → {True, True, False, False}
  ],

  (*neo-W into XY*)
  ListPlot[
   Table[
    {
     a,
     invasionplotXY[tryFAA, tryFAa, tryFaa, tryMAA, tryMAa, tryMaa, trywAf, trywaf,
      trywAm, trywam, tryαf, tryαm, setr[x, a, m], setR[x, a, m], setρ[x, a, m]]
    },
    {a, xmin, xmax, (xmax - xmin) / npoints}
   ],
   Joined → True,
   PlotRange → All,
   PlotStyle → Directive[Black, Thickness[lwd]],
   AxesOrigin → {xmin, ymin},
   Frame → {True, True, False, False}
  ],

  Plot[0, {x, xmin, xmax}, PlotStyle → {Black, Dotted}],

  FrameLabel → {"Chromosomal position of selected locus (cM)", ""},
  Epilog → {
    Text[Style["B", Bold], Scaled@letpos],
    (*Rotate[Text["Invasion fitness (λ_W^(XY)-1)",Scaled@ylabpos],90 Degree],*)
    Text["ancestral SDR", {x, ymax * 0.5}],
    Text["novel SDR", {m, ymin * 0.75}],
    Arrow[{{x, ymax * 0.4}, {x, 0}}],
    Arrow[{{m, ymin * 0.5}, {m, 0}}]
   },

  plotstyle[xmin, xmax, xtickmin,
   xtickmax, xint, ymin, ymax, ytickmin, ytickmax, yint]

 ]
```

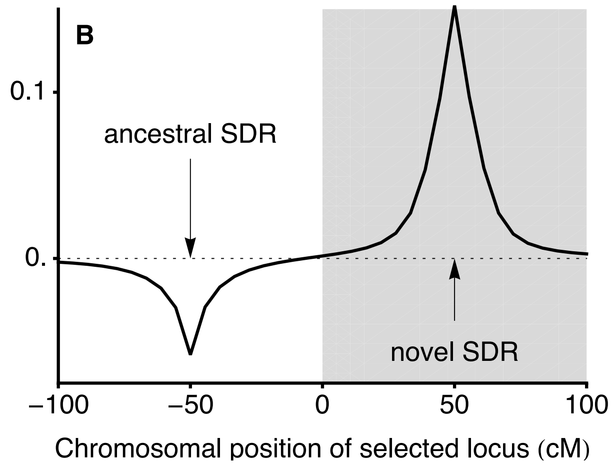

Panel C - neo-W invasion fitness as function of recombination with selected locus, with inset of neo-W temporal dynamics

Parameters

Collect selection parameters used above

```
subpar = {FAA → tryFAA, FAa → tryFAa, Faa → tryFaa,
   MAA → tryMAA, MAa → tryMAa, Maa → tryMaa, wAf → trywAf,
   waf → trywaf, wAm → trywAm, wam → trywam, αf → tryαf, αm → tryαm};
```

Set recombination rates

```
tryr = 0.005;
tryRs = {0.001, 0.02, 0.1, 0.5};

(*x limits*)
xmin = 0.0008;
xmax = 0.5;
xmarks = tryRs;
```

Main plot

```
tab = Table[
   equil = sieveXY[tryFAA, tryFAa, tryFaa, tryMAA, tryMAa,
      tryMaa, trywAf, trywaf, trywAm, trywam, tryαf, tryαm, tryr][[1]];
  λ /. Solve[(λ^2 + (-(λmA1 + λma1) + (χmA1 + χma1)) λ + (λmA1 - χmA1) (λma1 - χma1) -
              χmA1 χma1 /. pAveM → (1 - q) pXm + q pYm /. reverse /. subpar /. R → 0.5^b /.
        {pXf → equil[[1]], pXm → equil[[2]], pYm → equil[[3]],
         q → equil[[4]]}) == 0, λ], {b, 10, 1, -1/2}];
eigen1 = Transpose[Join[{Table[0.5^b, {b, 10, 1, -1/2}]},
     {Transpose[tab][[1]] - 1}]];
eigen2 = Transpose[Join[{Table[0.5^b, {b, 10, 1, -1/2}]},
     {Transpose[tab][[2]] - 1}]];
```

```
temp = Show[

  (*region of tighter linkage*)
  ListLogLinearPlot[{{xmin, ymax}, {tryr, ymax}},
   Joined → True, PlotStyle → {White, Thin}, Filling → ymin,
   FillingStyle → LightGray, PlotRange → {{xmin, xmax}, {ymin, ymax}}],
  ListLogLinearPlot[{{tryr, ymax}, {tryr, ymin}},
   Joined → True, PlotStyle → {White, Thin}],

  (*neo-W invasion fitness*)
  Show[
   ListLogLinearPlot[eigen2,
    Joined → True, PlotStyle → {Thick, Black}, PlotRange → All]
  ],

  (*0 line*)
  ListLogLinearPlot[{{xmin, 0}, {xmax, 0}}, Joined → True, PlotStyle → {Black}],

  (*mark recombination rates used in inset*)
  ListLogLinearPlot[{{0.001, -0.01}, {0.001, 0.01}},
   Joined → True, PlotStyle → {Black, Thick}],
  ListLogLinearPlot[{{0.02, -0.01}, {0.02, 0.01}},
   Joined → True, PlotStyle → {Red, Thick}],
  ListLogLinearPlot[{{0.1, -0.01}, {0.1, 0.01}},
   Joined → True, PlotStyle → {Blue, Thick}],
  ListLogLinearPlot[{{0.5, -0.01}, {0.5, 0.01}},
   Joined → True, PlotStyle → {Green, Thick}],

  FrameLabel → {"Rate of recombination (R)", ""},

  loglinearplotstyle[xmin, xmax, xmarks, ymin, ymax, ytickmin, ytickmax, yint, tryr]
 ]
```

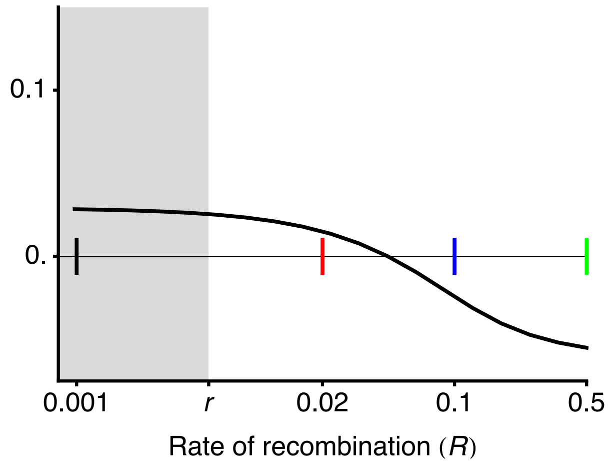

Make sure we get the same answer with the full characteristic polynomial

```
plot2 = ListLogLinearPlot[
   Table[{0.5^b, invasionplotXY[tryFAA, tryFAa, tryFaa, tryMAA, tryMAa,
      tryMaa, trywAf, trywaf, trywAm, trywam, tryαf, tryαm, tryr,
      0.5^b, tryr (1 - 0.5^b) + 0.5^b (1 - tryr)]}, {b, 10, 1, -1 / 2}],
   PlotStyle → {Red}, PlotRange → {{xmin, xmax}, {ymin, ymax}}];
Show[
 temp,
 plot2]
```

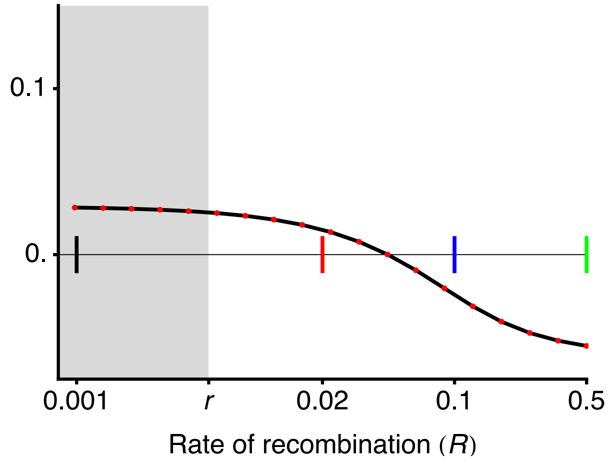

Inset parameters

```
trypm = 0.01;(*initial mutant frequency*)
tryk = 1;(*neo-W*)
startplot = 5;(*start plotting at this generation*)
endtime = 5000;(*number of generations to simulate*)
modvec = {0, 0, 1, 1, 0, 0, 1, 1, 0, 0, 0, 0, 0, 0, 0, 0};
(*use to get freq of neo-W amongst females from recursion simulation output*)
```

Inset

```
tryR = tryRs[[1]];
param = {tryFAA, tryFAa, tryFaa, tryMAA, tryMAa, tryMaa, trywAf, trywaf, trywAm,
   trywam, tryαf, tryαm, tryr, tryR, tryr (1 - tryR) + tryR (1 - tryr), tryk};
run[0] = generation[param, startgen[sieveXY[tryFAA, tryFAa, tryFaa, tryMAA, tryMAa,
       tryMaa, trywAf, trywaf, trywAm, trywam, tryαf, tryαm, tryr][[1]], trypm]];
For[time = 1, time ≤ endtime, time++, run[time] = generation[param, run[time - 1]]
]
MODtab1Black =
  Table[{Round[Exp[exptime]],
      generation[param, run[Round[Exp[exptime]]]].modvec
      ---------------------------------------------------
          sexratio[param, run[Round[Exp[exptime]]]]       },
   {exptime, Log[startplot], Log[endtime], 0.1}];
MOD1Black = ListLogLinearPlot[MODtab1Black, Joined → True,
  PlotRange → {{startplot, endtime}, {0, 1}}, PlotStyle → {Black}]
```

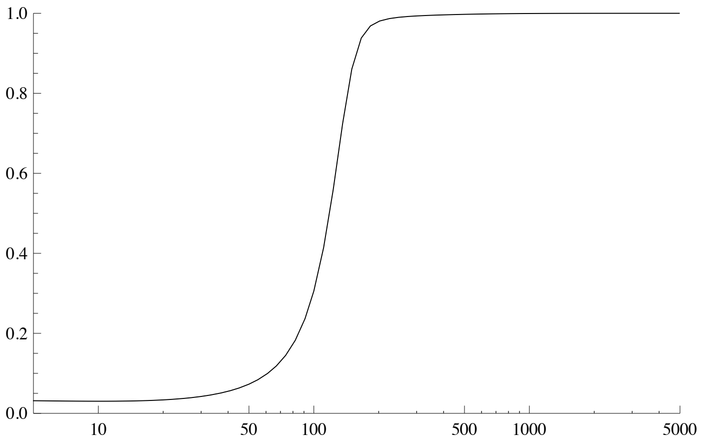

```
tryR = tryRs[[2]];
param = {tryFAA, tryFAa, tryFaa, tryMAA, tryMAa, tryMaa, trywAf, trywaf, trywAm,
   trywam, tryαf, tryαm, tryr, tryR, tryr (1 - tryR) + tryR (1 - tryr), tryk};
run[0] = generation[param, startgen[sieveXY[tryFAA, tryFAa, tryFaa, tryMAA, tryMAa,
      tryMaa, trywAf, trywaf, trywAm, trywam, tryαf, tryαm, tryr][[1]], trypm]];
For[time = 1, time ≤ endtime, time++, run[time] = generation[param, run[time - 1]]
]
MODtab1Red =
  Table[{Round[Exp[exptime]], generation[param, run[Round[Exp[exptime]]]].modvec / sexratio[param, run[Round[Exp[exptime]]]]},
   {exptime, Log[startplot], Log[endtime], 0.1}];
MOD1Red = ListLogLinearPlot[MODtab1Red, Joined → True,
  PlotRange → {{startplot, endtime}, {0, 1}}, PlotStyle → {Red}]
```

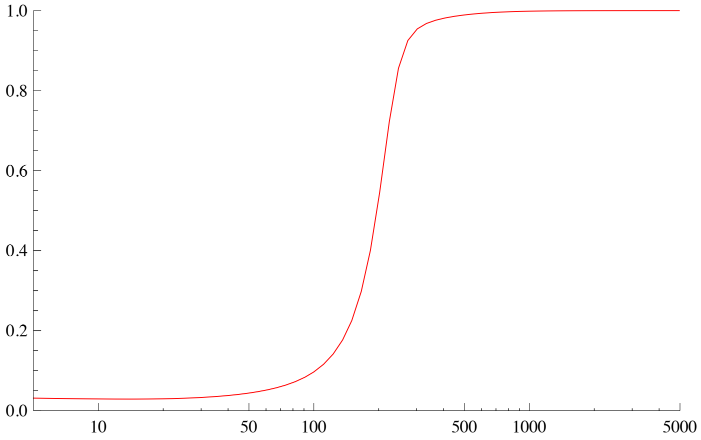

```
tryR = tryRs[[3]];
param = {tryFAA, tryFAa, tryFaa, tryMAA, tryMAa, tryMaa, trywAf, trywaf, trywAm,
   trywam, tryαf, tryαm, tryr, tryR, tryr (1 - tryR) + tryR (1 - tryr), tryk};
run[0] = generation[param, startgen[sieveXY[tryFAA, tryFAa, tryFaa, tryMAA, tryMAa,
      tryMaa, trywAf, trywaf, trywAm, trywam, tryαf, tryαm, tryr][[1]], trypm]];
For[time = 1, time ≤ endtime, time++, run[time] = generation[param, run[time - 1]]
]
MODtab1Blue =
  Table[{Round[Exp[exptime]], generation[param, run[Round[Exp[exptime]]]].modvec / sexratio[param, run[Round[Exp[exptime]]]]},
   {exptime, Log[startplot], Log[endtime], 0.1}];
MOD1Blue = ListLogLinearPlot[MODtab1Blue, Joined → True,
  PlotRange → {{startplot, endtime}, {0, 1}}, PlotStyle → {Blue}]
```

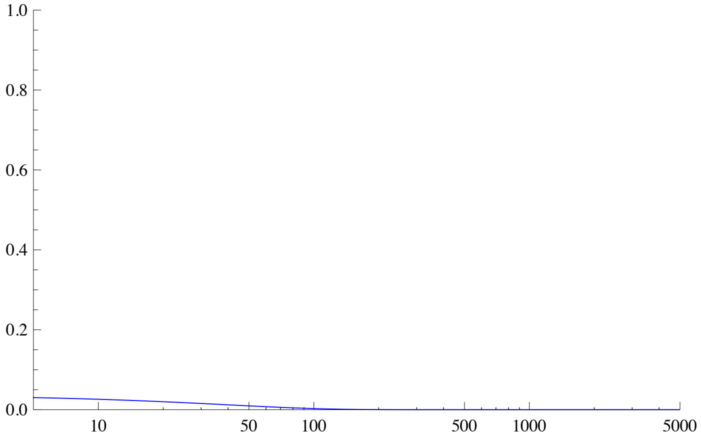

```
tryR = tryRs[[4]];
param = {tryFAA, tryFAa, tryFaa, tryMAA, tryMAa, tryMaa, trywAf, trywaf, trywAm,
   trywam, tryαf, tryαm, tryr, tryR, tryr (1 - tryR) + tryR (1 - tryr), tryk};
run[0] = generation[param, startgen[sieveXY[tryFAA, tryFAa, tryFaa, tryMAA, tryMAa,
      tryMaa, trywAf, trywaf, trywAm, trywam, tryαf, tryαm, tryr][[1]], trypm]];
For[time = 1, time ≤ endtime, time++, run[time] = generation[param, run[time - 1]]
]
MODtab1Green =
  Table[{Round[Exp[exptime]], generation[param, run[Round[Exp[exptime]]]].modvec / sexratio[param, run[Round[Exp[exptime]]]]},
   {exptime, Log[startplot], Log[endtime], 0.1}];
MOD1Green = ListLogLinearPlot[MODtab1Green, Joined → True,
  PlotRange → {{startplot, endtime}, {0, 1}}, PlotStyle → {Green}]
```

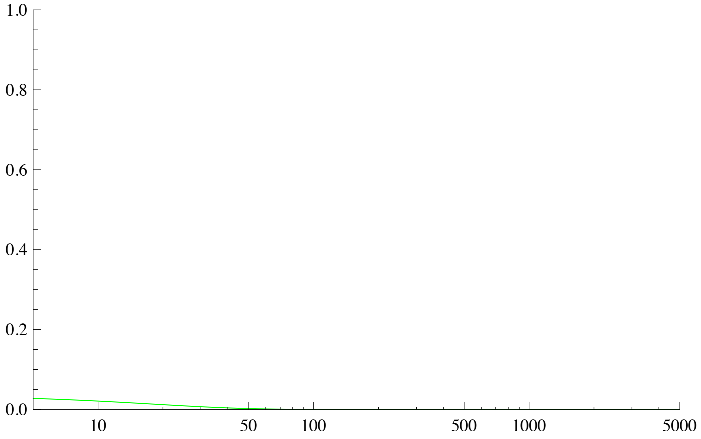

Main plot + inset

```
tempcomb =
 Show[
  temp,

  Epilog → {
    Inset[
     Show[
      MOD1Black, MOD1Red, MOD1Blue, MOD1Green,
      FrameLabel → {Style["Generation", 11], Style["frequency females
 with neo-W", 11]},
      FrameTicksStyle → Directive[8],
      insetloglinearplotstyle[
       startplot, endtime, {10, 100, 1000}, 0, 1.05, 0, 1, 0.5]
     ],
     {-0, 0.17}, {Right, Top}, {3.6 * 5 / 4, 3.6 * 5 / 4 * Sqrt[2]}
    ],
    Text[Style["C", Bold], Scaled@letpos]
   }

 ]
```

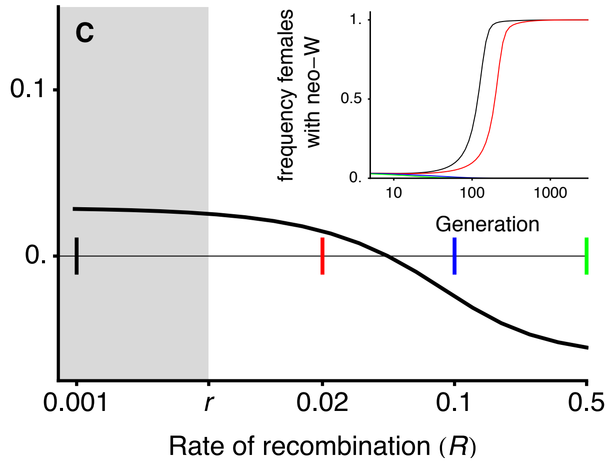


```
? FrameTicksStyle
```

FrameTicksStyle is an option for 2D graphics functions that specifies how frame ticks should be rendered. ≫

All panels

```
GraphicsRow[
 {positionplotA, positionplotB, tempcomb},
 Spacings → -30
]

Export[plotdir <> "PositionPlot_SexAntagTighter.eps",
  Magnify[%(*//rasterTrick*), 9.5 / 10], ImageSize → {10 inches, 2 inches}];
Export[plotdir <> "PositionPlot_SexAntagTighter.pdf",
  Magnify[%%(*//rasterTrick*), 9.5 / 10], ImageSize → {10 inches, 2 inches}];
```

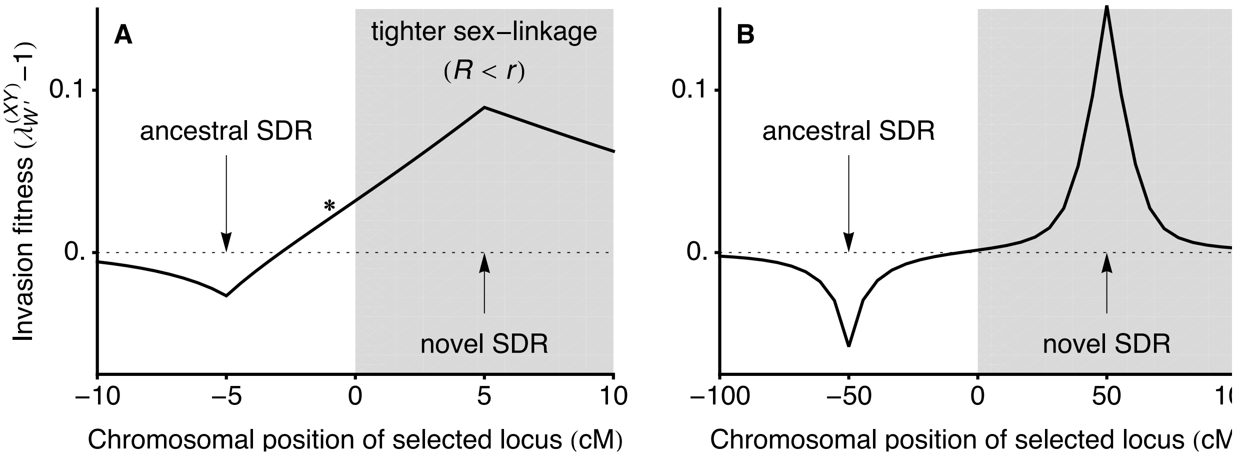

Figure 4 - plot of neo-W invasion fitness as function of selected locus location, with haploid selection

Plotting parameters

```
(*Pad={{40,20},{50,30}};  (*space around plot*)*)
xplotmin = -50;
xplotmax = 50;
xplotinterval = 25;
yplotmin = -0.04;
yplotmax = 0.07;
yplotinterval = 0.02;
(*ylabpos={-0.1,0.5};  (*relative location of y axis label position*)*)
ytickmin = -0.1;
ytickmax = 0.1;
yint = 0.1;

npoints = 120;
```

Panel A - meiotic drive in males

Parameters

```
x = -25;
m = 25;

trysAf = 0.1;
tryhAf = 0.7;
tryhAm = 0.7;
trysAm = 0.1;
tryt = 0;
tryFAA = 1 + trysAf;
tryFAa = 1 + tryhAf trysAf;
tryFaa = 1;
tryMAA = 1 + trysAm;
tryMAa = 1 + tryhAm trysAm;
tryMaa = 1;
trywAm = 1 + tryt;
trywam = 1;
trywAf = 1;
trywaf = 1;
tryαm = 0.45;
tryαf = 0.5;
```

Plot

```
plotA =
 Show[

  (*region of tighter linkage*)
  RegionPlot[x > 0, {x, xplotmin, xplotmax}, {y, yplotmin, yplotmax},
   PlotStyle → {LightGray},
   BoundaryStyle → None,
   Frame → {True, True, False, False}
  ],

  (*neo-W into XY*)
  ListPlot[DeleteCases[Table[{a,
      invasionplotXY[tryFAA, tryFAa, tryFaa, tryMAA, tryMAa, tryMaa, trywAf, trywaf,
       trywAm, trywam, tryαf, tryαm, setr[x, a, m], setR[x, a, m], setρ[x, a, m]]},
     {a, xplotmin, xplotmax, (xplotmax - xplotmin) / npoints}], {n_, Null}, 2],
   Joined → True,
   PlotRange → {yplotmin, All},
   PlotStyle → Directive[Black, Thickness[lwd]]
  ],

  Plot[0, {x, xplotmin, xplotmax}, PlotStyle → {Black, Dotted}],

  Epilog → {
    Text[Style["A", Bold], Scaled@letpos],
    Rotate[
     Text[Style["Invasion fitness (λW'^(XY)-1)"], Scaled@{-0.1, 0.5}], 90 Degree],
    Text[Style["tighter sex-linkage"], {25, yplotmax * 0.9}],
    Text["(R < r)", {25, yplotmax * 0.75}],
    Text[Style["meiotic drive in males", Bold],
     (*{xplotmax,yplotmin*0.75}*)Scaled@{0.5, 1.05}, {0, 0}],
    Text["ancestral SDR", {x, yplotmin * 0.75}],
    Text["novel SDR", {m, yplotmin * 0.75}],
    Arrow[{{-25, yplotmin * 0.5}, {-25, 0}}],
    Arrow[{{25, yplotmin * 0.5}, {25, 0}}]
   },
  FrameLabel → {"Chromosomal position of selected locus (cM)", ""},
  plotstyle[xplotmin, xplotmax, xplotmin, xplotmax,
   xplotinterval, yplotmin, yplotmax, ytickmin, ytickmax, yint]

 ]
```

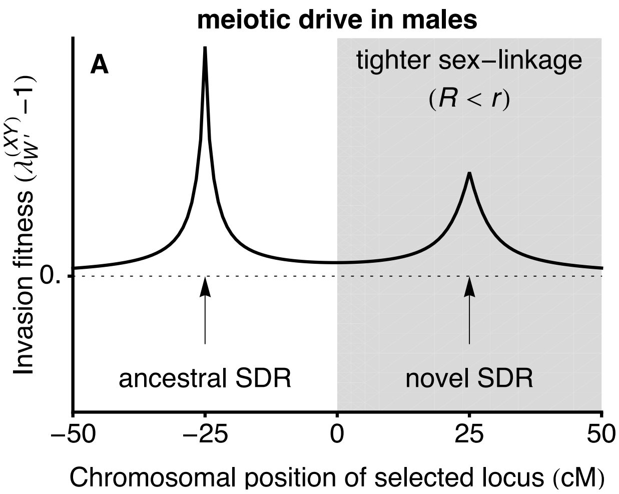

Panel B - haploid competition in males

Parameters

```
x = -25;
m = 25;

trysAf = 0.05;
tryhAf = 0.7;
tryhAm = 0.7;
trysAm = 0.15;
tryt = -0.1;
tryFAA = 1 + trysAf;
tryFAa = 1 + tryhAf trysAf;
tryFaa = 1;
tryMAA = 1 + trysAm;
tryMAa = 1 + tryhAm trysAm;
tryMaa = 1;
trywAm = 1 + tryt;
trywam = 1;
trywAf = 1;
trywaf = 1;
tryαm = 0.5;
tryαf = 0.5;
```

Plot

```
plotB =
 Show[

  (*region of tighter linkage*)
  RegionPlot[x > 0, {x, xplotmin, xplotmax}, {y, yplotmin, yplotmax},
   PlotStyle → {LightGray},
   BoundaryStyle → None,
   Frame → {True, True, False, False}
  ],

  (*neo-W into XY*)
  ListPlot[DeleteCases[Table[{a,
       invasionplotXY[tryFAA, tryFAa, tryFaa, tryMAA, tryMAa, tryMaa, trywAf, trywaf,
        trywAm, trywam, tryαf, tryαm, setr[x, a, m], setR[x, a, m], setρ[x, a, m]]},
     {a, xplotmin, xplotmax, (xplotmax - xplotmin) / npoints}], {n_, Null}, 2],
   Joined → True,
   PlotRange → {yplotmin, All},
   PlotStyle → Directive[Black, Thickness[lwd]]
  ],

  Plot[0, {x, xplotmin, xplotmax}, PlotStyle → {Black, Dotted}],

  Epilog → {
    Text[Style["B", Bold], Scaled@letpos],
    (*Rotate[
     Text[Style["Invasion fitness (λ-1)",14],Scaled@ylabpos],90 Degree],*)
    Text[Style["haploid competition in males", Bold],
     (*{xplotmax,yplotmin*0.75}*)Scaled@{0.5, 1.05}, {0, 0}],
    Text["ancestral SDR", {x, yplotmin * 0.75}],
    Text["novel SDR", {m, yplotmin * 0.75}],
    Arrow[{{x, yplotmin * 0.5}, {x, 0}}],
    Arrow[{{m, yplotmin * 0.5}, {m, 0}}]
   },
  FrameLabel → {"Chromosomal position of selected locus (cM)", ""},
  plotstyle[xplotmin, xplotmax, xplotmin, xplotmax,
   xplotinterval, yplotmin, yplotmax, ytickmin, ytickmax, yint]
 ]
```

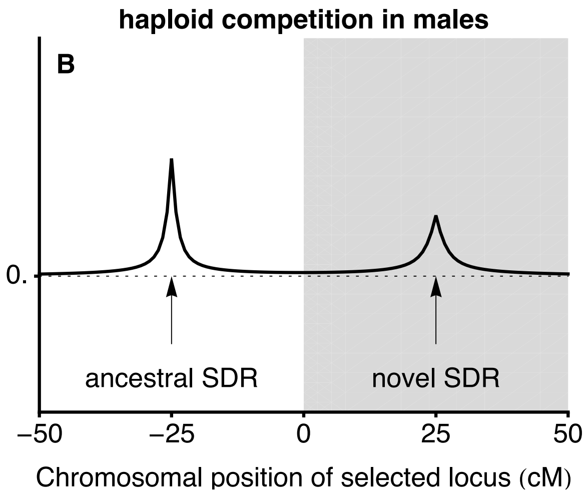

All panels

```
GraphicsRow[
 {plotA, plotB},
 Spacings → -30
]

Export[plotdir <> "PositionPlot.eps", %(*//rasterTrick*)];
```

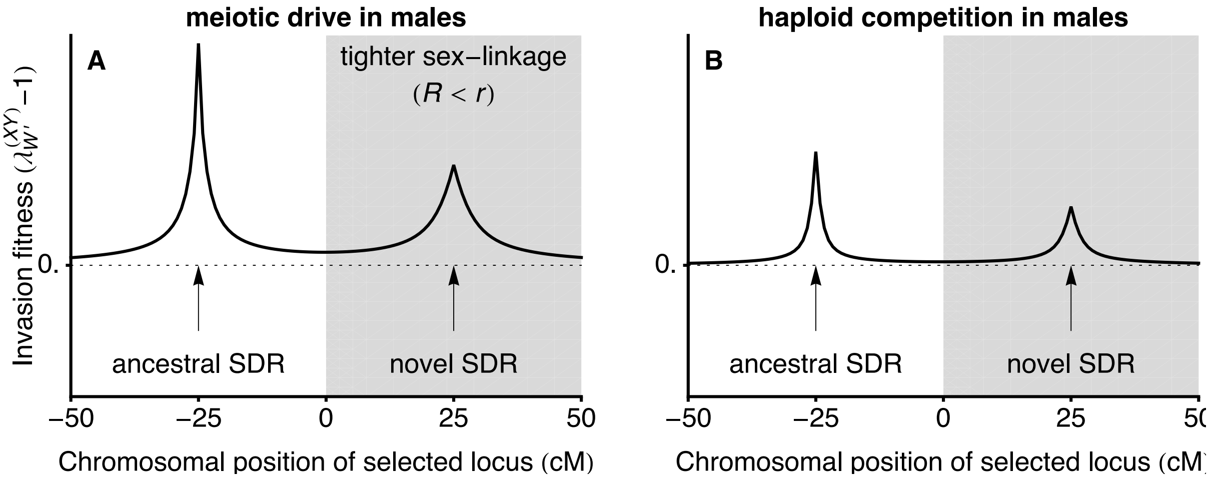

Figure 5 - sex ratio is not a good predictor of neo-SD invasion

Parameters

Plotting parameters

```
yplotmin = -0.075;
yplotmax = 0.15;
yplotinterval = 0.02;
```

```
Pad = {{50, 20}, {40, 30}}; (*space around plot*)
letpos = {.05, .93}; (*relative location of letter, eg A, in figure*)
ylabpos = {-0.175, 0.5}; (*relative location of y axis label position*)
```

Extra parameters

```
modvec = {0, 0, 1, 1, 0, 0, 1, 1, 0, 0, 0, 0, 0, 0, 0, 0}; (*fraction female with W*)
modvec2 = {1, 1, 1, 1, 1, 1, 1, 1, 0, 0, 0, 0, 0, 0, 0, 0};(*fraction female*)
```

```
loglinearplotINVoptions = {Frame → {{True, False}, {True, False}},
    PlotRangeClipping → False,
    ImagePadding → Pad,
    FrameStyle -> Directive[Black, Thickness[lwd]],
    FrameTicksStyle → {{Directive[Black, Thickness[lwd], FontColor → Black],
        Directive[Black, Thickness[lwd]]},
       {Directive[Black, Thickness[lwd]], Directive[Black, Thickness[lwd]]}},
    FrameTicks → {{{5, 5, {0, 0.01}}, {10, 10, {0, 0.01}}, {100, 100, {0, 0.01}},
        {1000, 1000, {0, 0.01}}}, Table[{y, y, ticksize}, {y, 0, 1, 0.5}]},
    BaseStyle → {FontFamily → "Helvetica", FontSize → 12},
    ImageSize → {xsize, xsize aspectratio},
    AspectRatio → aspectratio,
    Axes → True};
```

```
loglinearplotINVSRoptions = {Frame → {{True, False}, {True, False}},
    PlotRangeClipping → False,
    ImagePadding → Pad,
    FrameStyle -> Directive[Black, Thickness[lwd]],
    FrameTicksStyle → {{Directive[Black, Thickness[lwd], FontColor → Black],
        Directive[Black, Thickness[lwd]]},
       {Directive[Black, Thickness[lwd]], Directive[Black, Thickness[lwd]]}},
    FrameTicks → {{{5, 5, {0, 0.01}}, {10, 10, {0, 0.01}}, {50, 50, {0, 0.01}},
        {100, 100, {0, 0.01}}, {500, 500, {0, 0.01}}, {1000, 1000, {0, 0.01}}},
       Table[{y, y, ticksize}, {y, 0.4, 0.6, 0.1}]},
    BaseStyle → {FontFamily → "Helvetica", FontSize → 12},
    ImageSize → {xsize, xsize aspectratio },
    AspectRatio → aspectratio,
    Axes → True};
```

```
startplot = 5;
endtime = 1000;
tryR = 0;
trypm = 0.01;
tryk = 1;
```

Panel A - neo-W invading XY

Parameters

```
trys = 0.2;
tryh = 0.7;
tryαDm = -0.1;
tryFAA = 1 + trys ;
tryFAa = 1 + trys tryh;
tryFaa = 1;
tryMAA = 1 + trys;
tryMAa = 1 + trys tryh;
tryMaa = 1;
trywAf = 1;
trywaf = 1;
trywAm = 1;
trywam = 1;
tryαf = 0.5;
tryαm = 0.5 + tryαDm;
tryr = 0.02;
subpar1 =
  {FAA → tryFAA, FAa → tryFAa, Faa → tryFaa, MAA → tryMAA, MAa → tryMAa, Maa → tryMaa,
   wAf -> trywAf, waf -> trywaf, wAm -> trywAm, wam -> trywam, αf -> tryαf, αm -> tryαm};
```

Plots

```
tryR = 0.001;
param = {tryFAA, tryFAa, tryFaa, tryMAA, tryMAa, tryMaa, trywAf, trywaf, trywAm,
   trywam, tryαf, tryαm, tryr, tryR, tryr (1 - tryR) + tryR (1 - tryr), tryk};
run[0] = generation[param, startgen[sieveXY[tryFAA, tryFAa, tryFaa, tryMAA, tryMAa,
       tryMaa, trywAf, trywaf, trywAm, trywam, tryαf, tryαm, tryr][[1]], trypm]];
For[time = 1, time ≤ endtime, time++, run[time] = generation[param, run[time - 1]]
]
MODtab2Black =
  Table[{Round[Exp[exptime]], generation[param, run[Round[Exp[exptime]]]].modvec / sexratio[param, run[Round[Exp[exptime]]]]},
   {exptime, Log[startplot], Log[endtime], 0.1}];
MODBlack = ListLogLinearPlot[MODtab2Black, Joined → True,
   PlotRange → {{startplot, endtime}, {0, 1.05}},
   PlotStyle → {Black}, loglinearplotINVoptions];
SRtabBlack = Table[{Round[Exp[exptime]], sexratio[param, run[Round[Exp[exptime]]]]},
   {exptime, Log[startplot], Log[endtime], 0.1}];
SRBlack = ListLogLinearPlot[SRtabBlack, Joined → True,
   PlotRange → {{startplot, endtime}, {0.4, 0.601}},
   PlotStyle → {Black, Thick}, loglinearplotINVSRoptions];
```

```
tryR = 0.02;
param = {tryFAA, tryFAa, tryFaa, tryMAA, tryMAa, tryMaa, trywAf, trywaf, trywAm,
   trywam, tryαf, tryαm, tryr, tryR, tryr (1 - tryR) + tryR (1 - tryr), tryk};
run[0] = generation[param, startgen[sieveXY[tryFAA, tryFAa, tryFaa, tryMAA, tryMAa,
      tryMaa, trywAf, trywaf, trywAm, trywam, tryαf, tryαm, tryr][[1]], trypm]];
For[time = 1, time ≤ endtime, time++, run[time] = generation[param, run[time - 1]]
]
MODtabRed =
  Table[{Round[Exp[exptime]], generation[param, run[Round[Exp[exptime]]]].modvec / sexratio[param, run[Round[Exp[exptime]]]]},
   {exptime, Log[startplot], Log[endtime], 0.1}];
MODRed = ListLogLinearPlot[MODtabRed, Joined → True, PlotRange →
    {{startplot, endtime}, {0, 1.05}}, PlotStyle → {Red}, loglinearplotINVoptions];
SRtabRed = Table[{Round[Exp[exptime]], sexratio[param, run[Round[Exp[exptime]]]]},
   {exptime, Log[startplot], Log[endtime], 0.1}];
SRRed = ListLogLinearPlot[SRtabRed, Joined → True,
   PlotRange → {{startplot, endtime}, {0.4, 0.601}},
   PlotStyle → {Red, Thick}, loglinearplotINVSRoptions];

tryR = 0.1;
param = {tryFAA, tryFAa, tryFaa, tryMAA, tryMAa, tryMaa, trywAf, trywaf, trywAm,
   trywam, tryαf, tryαm, tryr, tryR, tryr (1 - tryR) + tryR (1 - tryr), tryk};
run[0] = generation[param, startgen[sieveXY[tryFAA, tryFAa, tryFaa, tryMAA, tryMAa,
      tryMaa, trywAf, trywaf, trywAm, trywam, tryαf, tryαm, tryr][[1]], trypm]];
For[time = 1, time ≤ endtime, time++, run[time] = generation[param, run[time - 1]]
]
MODtabBlue =
  Table[{Round[Exp[exptime]], generation[param, run[Round[Exp[exptime]]]].modvec / sexratio[param, run[Round[Exp[exptime]]]]},
   {exptime, Log[startplot], Log[endtime], 0.1}];
MODBlue = ListLogLinearPlot[MODtabBlue, Joined → True, PlotRange →
    {{startplot, endtime}, {0, 1.05}}, PlotStyle → {Blue}, loglinearplotINVoptions];
SRtabBlue = Table[{Round[Exp[exptime]], sexratio[param, run[Round[Exp[exptime]]]]},
   {exptime, Log[startplot], Log[endtime], 0.1}];
SRBlue = ListLogLinearPlot[SRtabBlue, Joined → True,
   PlotRange → {{startplot, endtime}, {0.4, 0.601}},
   PlotStyle → {Blue, Thick}, loglinearplotINVSRoptions];
```

```
tryR = 0.5;
param = {tryFAA, tryFAa, tryFaa, tryMAA, tryMAa, tryMaa, trywAf, trywaf, trywAm,
   trywam, tryαf, tryαm, tryr, tryR, tryr (1 - tryR) + tryR (1 - tryr), tryk};
run[0] = generation[param, startgen[sieveXY[tryFAA, tryFAa, tryFaa, tryMAA, tryMAa,
      tryMaa, trywAf, trywaf, trywAm, trywam, tryαf, tryαm, tryr][[1]], trypm]];
For[time = 1, time ≤ endtime, time++, run[time] = generation[param, run[time - 1]]
]
MODtabGreen =
  Table[{Round[Exp[exptime]], generation[param, run[Round[Exp[exptime]]]].modvec / sexratio[param, run[Round[Exp[exptime]]]]},
   {exptime, Log[startplot], Log[endtime], 0.1}];
MODGreen = ListLogLinearPlot[MODtabGreen, Joined → True,
   PlotRange → {{startplot, endtime}, {0, 1.05}},
   PlotStyle → {Green}, loglinearplotINVoptions];
SRtabGreen = Table[{Round[Exp[exptime]], sexratio[param, run[Round[Exp[exptime]]]]},
   {exptime, Log[startplot], Log[endtime], 0.1}];
SRGreen = ListLogLinearPlot[SRtabGreen, Joined → True,
   PlotRange → {{startplot, endtime}, {0.4, 0.601}},
   PlotStyle → {Green, Thick}, loglinearplotINVSRoptions];
```

```
plotA = Legended[Show[SRBlack, SRRed, SRBlue, SRGreen,
   ImagePadding → Pad,
   FrameLabel → {{Style["Zygotic sex ratio (ζ, fraction male)", FontSize → 14],
      ""}, {Style["Generation", FontSize → 14], ""}},
   Epilog → {Inset[Show[MODBlack, MODRed, MODBlue, MODGreen, (*ImageSize→150,*)
        BaseStyle → {TextStyle → Bold, FontFamily → "Helvetica", FontSize → 8},
        FrameLabel → {{Style["frequency of females
 with neo-W", FontSize → 8], ""}, {Style["", FontSize → 14], ""}}],
       Scaled@{1.05, -0.15}, {Right, Bottom}, {3.4, 3.4 * Sqrt[2]}],
      Text[Style["A", 14, Bold], Scaled@letpos],
      Text[Style["neo-W", Bold, 14], Scaled@{0.5, 1.1}]}],
  Placed[
   LineLegend[
    {Directive[Thickness[lwd * 20], Black],
     Directive[Thickness[lwd * 20], Red],
     Directive[Thickness[lwd * 20], Blue],
     Directive[Thickness[lwd * 20], Green]
    },
    {Style["R=0.001", 12],
     Style["R=0.02", 12],
     Style["R=0.1", 12],
     Style["R=0.5", 12]
    },
    LegendFunction → "Frame",
    LegendLayout → "Column"
   ],
   Scaled@{0.85, 0.875}
  ]
 ]
```

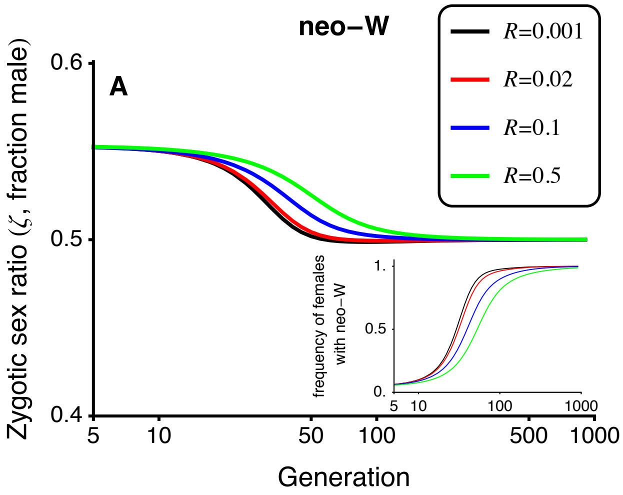

Panel B - neo-Y invading ZW

Parameters

Here we are going to adjust the fitness parameters only to change definition of sex.
Also have to adjust sex ratio to 1-sexratio because it gives the frequency of the ancestrally heterozygous sex.

```
trys = 0.2;
tryh = 0.7;
tryαDm = -0.1;
tryFAA = 1 + trys ;
tryFAa = 1 + trys tryh;
tryFaa = 1;
tryMAA = 1 + trys;
tryMAa = 1 + trys tryh;
tryMaa = 1;
trywAf = 1;
trywaf = 1;
trywAm = 1;
trywam = 1;
tryαf = 0.5 + tryαDm;
tryαm = 0.5;
tryr = 0.02;
subpar1 =
  {FAA → tryFAA, FAa → tryFAa, Faa → tryFaa, MAA → tryMAA, MAa → tryMAa, Maa → tryMaa,
   wAf -> trywAf, waf -> trywaf, wAm -> trywAm, wam -> trywam, αf -> tryαf, αm -> tryαm};
```

Plots

```
tryR = 0.001;
param = {tryFAA, tryFAa, tryFaa, tryMAA, tryMAa, tryMaa, trywAf, trywaf, trywAm,
   trywam, tryαf, tryαm, tryr, tryR, tryr (1 - tryR) + tryR (1 - tryr), tryk};
run[0] = generation[param, startgen[sieveXY[tryFAA, tryFAa, tryFaa, tryMAA, tryMAa,
      tryMaa, trywAf, trywaf, trywAm, trywam, tryαf, tryαm, tryr][[1]], trypm]];
For[time = 1, time ≤ endtime, time++, run[time] = generation[param, run[time - 1]]
]
MODtab2Black =
  Table[{Round[Exp[exptime]], generation[param, run[Round[Exp[exptime]]]].modvec / (1 - sexratio[param, run[Round[Exp[exptime]]]])},
   {exptime, Log[startplot], Log[endtime], 0.1}];
MODBlack = ListLogLinearPlot[MODtab2Black, Joined → True,
   PlotRange → {{startplot, endtime}, {0, 1.05}},
   PlotStyle → {Black}, loglinearplotINVoptions];
SRtabBlack = Table[{Round[Exp[exptime]],
    1 - sexratio[param, run[Round[Exp[exptime]]]]},
   {exptime, Log[startplot], Log[endtime], 0.1}];
SRBlack = ListLogLinearPlot[SRtabBlack, Joined → True,
   PlotRange → {{startplot, endtime}, {0.4, 0.601}},
   PlotStyle → {Black, Thick}, loglinearplotINVSRoptions];
```

```
tryR = 0.02;
param = {tryFAA, tryFAa, tryFaa, tryMAA, tryMAa, tryMaa, trywAf, trywaf, trywAm,
   trywam, tryαf, tryαm, tryr, tryR, tryr (1 - tryR) + tryR (1 - tryr), tryk};
run[0] = generation[param, startgen[sieveXY[tryFAA, tryFAa, tryFaa, tryMAA, tryMAa,
      tryMaa, trywAf, trywaf, trywAm, trywam, tryαf, tryαm, tryr][[1]], trypm]];
For[time = 1, time ≤ endtime, time++, run[time] = generation[param, run[time - 1]]
]
MODtabRed =
  Table[{Round[Exp[exptime]], generation[param, run[Round[Exp[exptime]]]].modvec / (1 - sexratio[param, run[Round[Exp[exptime]]]])},
   {exptime, Log[startplot], Log[endtime], 0.1}];
MODRed = ListLogLinearPlot[MODtabRed, Joined → True, PlotRange →
    {{startplot, endtime}, {0, 1.05}}, PlotStyle → {Red}, loglinearplotINVoptions];
SRtabRed = Table[{Round[Exp[exptime]],
    1 - sexratio[param, run[Round[Exp[exptime]]]]},
   {exptime, Log[startplot], Log[endtime], 0.1}];
SRRed = ListLogLinearPlot[SRtabRed, Joined → True,
   PlotRange → {{startplot, endtime}, {0.4, 0.601}},
   PlotStyle → {Red, Thick}, loglinearplotINVSRoptions];

tryR = 0.1;
param = {tryFAA, tryFAa, tryFaa, tryMAA, tryMAa, tryMaa, trywAf, trywaf, trywAm,
   trywam, tryαf, tryαm, tryr, tryR, tryr (1 - tryR) + tryR (1 - tryr), tryk};
run[0] = generation[param, startgen[sieveXY[tryFAA, tryFAa, tryFaa, tryMAA, tryMAa,
      tryMaa, trywAf, trywaf, trywAm, trywam, tryαf, tryαm, tryr][[1]], trypm]];
For[time = 1, time ≤ endtime, time++, run[time] = generation[param, run[time - 1]]
]
MODtabBlue =
  Table[{Round[Exp[exptime]], generation[param, run[Round[Exp[exptime]]]].modvec / (1 - sexratio[param, run[Round[Exp[exptime]]]])},
   {exptime, Log[startplot], Log[endtime], 0.1}];
MODBlue = ListLogLinearPlot[MODtabBlue, Joined → True, PlotRange →
    {{startplot, endtime}, {0, 1.05}}, PlotStyle → {Blue}, loglinearplotINVoptions];
SRtabBlue = Table[{Round[Exp[exptime]],
    1 - sexratio[param, run[Round[Exp[exptime]]]]},
   {exptime, Log[startplot], Log[endtime], 0.1}];
SRBlue = ListLogLinearPlot[SRtabBlue, Joined → True,
   PlotRange → {{startplot, endtime}, {0.4, 0.601}},
   PlotStyle → {Blue, Thick}, loglinearplotINVSRoptions];
```

```
tryR = 0.5;
param = {tryFAA, tryFAa, tryFaa, tryMAA, tryMAa, tryMaa, trywAf, trywaf, trywAm,
   trywam, tryαf, tryαm, tryr, tryR, tryr (1 - tryR) + tryR (1 - tryr), tryk};
run[0] = generation[param, startgen[sieveXY[tryFAA, tryFAa, tryFaa, tryMAA, tryMAa,
      tryMaa, trywAf, trywaf, trywAm, trywam, tryαf, tryαm, tryr][[1]], trypm]];
For[time = 1, time ≤ endtime, time++, run[time] = generation[param, run[time - 1]]
]
MODtabGreen =
  Table[{Round[Exp[exptime]], generation[param, run[Round[Exp[exptime]]]].modvec / (1 - sexratio[param, run[Round[Exp[exptime]]]])},
   {exptime, Log[startplot], Log[endtime], 0.1}];
MODGreen = ListLogLinearPlot[MODtabGreen, Joined → True,
   PlotRange → {{startplot, endtime}, {0, 1.05}},
   PlotStyle → {Green}, loglinearplotINVoptions];
SRtabGreen = Table[{Round[Exp[exptime]],
    1 - sexratio[param, run[Round[Exp[exptime]]]]},
   {exptime, Log[startplot], Log[endtime], 0.1}];
SRGreen = ListLogLinearPlot[SRtabGreen, Joined → True,
   PlotRange → {{startplot, endtime}, {0.4, 0.601}},
   PlotStyle → {Green, Thick}, loglinearplotINVSRoptions];

plotB = Show[SRBlack, SRRed, SRBlue, SRGreen,
  ImagePadding → Pad,
  FrameLabel →
   {{Style["", FontSize → 14], ""}, {Style["Generation", FontSize → 14], ""}},
  Epilog → {Inset[Show[MODBlack, MODRed, MODBlue, MODGreen, (*ImageSize→150,*)
      BaseStyle → {TextStyle → Bold, FontFamily → "Helvetica", FontSize → 8},
      FrameLabel → {{Style["frequency of males
with neo-Y", FontSize → 8], ""}, {Style["", FontSize → 14], ""}}],
     Scaled@{1.05, -0.15}, {Right, Bottom}, {3.4, 3.4 * Sqrt[2]}],
    Text[Style["B", 14, Bold], Scaled@letpos],
    Text[Style["neo-Y", Bold, 14], Scaled@{0.5, 1.1}]}
 ]
```

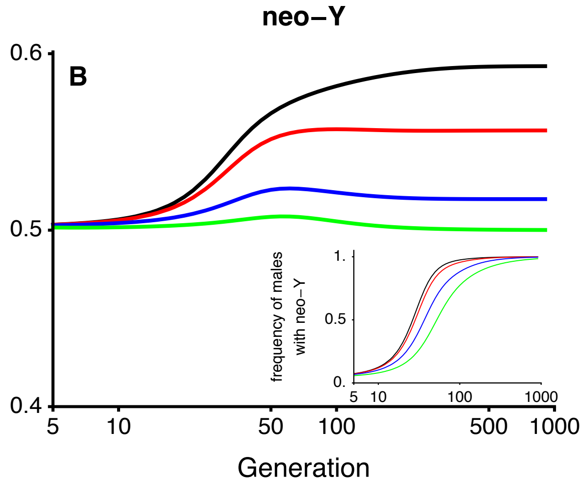

All panels

```
GraphicsRow[{plotA, plotB}, Spacings → -35]
```

```
Export[plotdir <> "Temporal_SR.eps", %(*//rasterTrick*)];
```

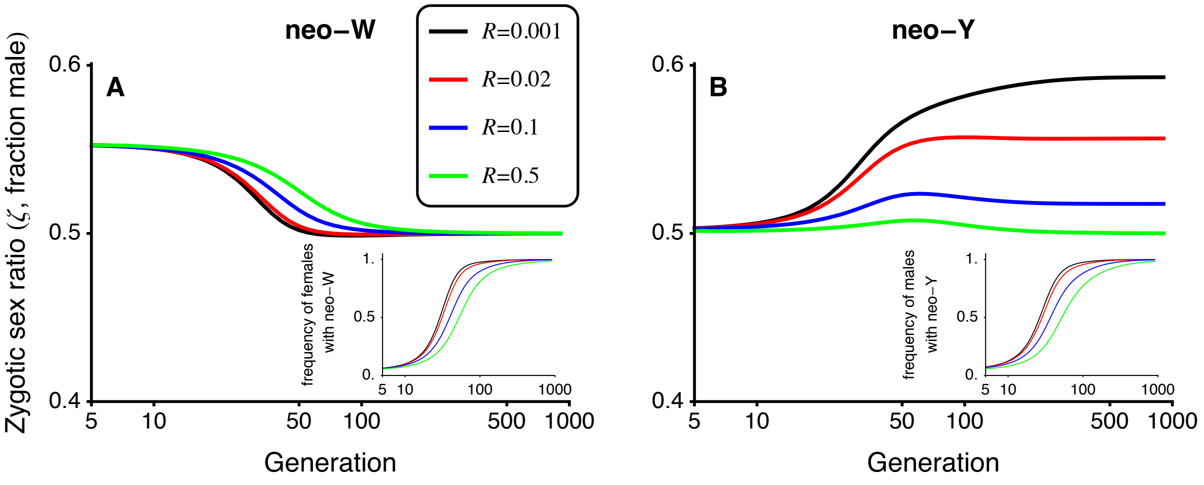

Figure S.1 - neo-W invasion fitness as a function of the position of the selected locus with overdominance in males, no haploid selection

Plotting parameters

```
(*Pad={{50,20},{50,20}};  (*space around plot*)*)
yplotmin = -0.075;
yplotmax = 0.15;
yplotinterval = 0.02;
(*ylabpos={-0.15,0.5};  (*relative location of y axis label position*)*)
```

Panel A - loose linkage; overdominance in males and directional selection for a in females

Parameters

```
x = -25;
m = 25;

tryt = 0;
tryFAA = 0.85;
tryFAa = 1;
tryFaa = 1.05;
tryMAA = 0.75;
tryMAa = 1;
tryMaa = 0.75;
tryMA = 1 + tryt;
tryMa = 1;
tryFA = 1;
tryFa = 1;
tryMdA = 0.5;
tryFdA = 0.5;

xplotmin = -50;
xplotmax = 50;
xplotinterval = 25;
npoints = 32;
```

Plot

```
positionplotA =
 Legended[
  Show[

   (*region of tighter linkage*)
   RegionPlot[x > 0, {x, xplotmin, xplotmax}, {y, yplotmin, yplotmax},
    PlotStyle → {LightGray},
    BoundaryStyle → None,
    PlotRange → {yplotmin, yplotmax},
    AxesOrigin → {xplotmin, yplotmin},
    Frame → {True, True, False, False}
   ],

   (*neo-W into XY*)
   ListPlot[
    Table[{a, invasionplotXY2[{tryFAA, tryFAa, tryFaa, tryMAA, tryMAa, tryMaa,
        tryFA, tryFa, tryMA, tryMa, tryFdA, tryMdA, setr[x, a, m], setR[x, a, m],
        setρ[x, a, m]}, sieveXY[tryFAA, tryFAa, tryFaa, tryMAA, tryMAa, tryMaa,
         tryFA, tryFa, tryMA, tryMa, tryFdA, tryMdA, setr[x, a, m]][[1]]]},
      {a, xplotmin, xplotmax, (xplotmax - xplotmin) / npoints}],
    Joined → True,
    PlotRange → {yplotmin, All},
    PlotStyle → Directive[Blue, Thickness[lwd]],
```

```
     AxesOrigin → {xplotmin, yplotmin},
     Frame → {True, True, False, False}
    ],
    ListPlot[Table[{a,
       invasionplotXY2[{tryFAA, tryFAa, tryFaa, tryMAA, tryMAa, tryMaa, tryFA, tryFa,
         tryMA, tryMa, tryFdA, tryMdA, setr[x, a, m], setR[x, a, m], setρ[x, a, m]},
        sieveXY[tryFAA, tryFAa, tryFaa, tryMAA, tryMAa, tryMaa, tryFA, tryFa,
          tryMA, tryMa, tryFdA, tryMdA, setr[x, a, m]][[2]]]}, {a, -26, -24, 0.25}],
     Joined → True,
     PlotRange → {yplotmin, All},
     PlotStyle → Directive[Black, Thickness[lwd]],
     AxesOrigin → {xplotmin, yplotmin},
     Frame → {True, True, False, False}
    ],

    Plot[0, {x, xplotmin, xplotmax}, PlotStyle → {Black, Dotted}],

    plotstyle[xplotmin, xplotmax, xplotmin, xplotmax,
     xplotinterval, yplotmin, yplotmax, ytickmin, ytickmax, yint],
    Epilog → {
      Text[Style["A", Bold], Scaled@letpos],
      Rotate[
       Text[Style["Invasion fitness (λ_W^(XY)-1)"], Scaled@{-0.15, 0.5}], 90 Degree]
      (*,Text[Style["tighter sex-linkage",14],{m,yplotmax*0.9}],*)
      (*,Text["ancestral SDR",{x,yplotmin*0.75}],
      Text["novel SDR",{m,yplotmin*0.75}],
      Arrow[{{x,yplotmin*0.5},{x,0}}],
      Arrow[{{m,yplotmin*0.5},{m,0}}]*)
     }

   ],
   Placed[
    LineLegend[
     {Directive[Thickness[0.1], Black],
      Directive[Thickness[0.1], Blue]
     },
     {Style["Y-a equilibrium", 14 * posplotscale],
      Style["Y-A equilibrium", 14 * posplotscale]
     },
     LegendFunction → "Frame",
     LegendLayout → "Column"
    ],
    Scaled@{0.7, 0.75}
   ]
  ]
```

Part::partw : Part 2 of {{0.0283897, 0.0313209, 0.959256, 0.5}} does not exist. ≫

Part::partw : Part 2 of {{0.0213283, 0.0235138, 0.969581, 0.5}} does not exist. ≫

Part::partw : Part 2 of {{0.0213283, 0.0235138, 0.969581, 0.5}} does not exist. ≫

General::stop : Further output of Part::partw will be suppressed during this calculation. ≫

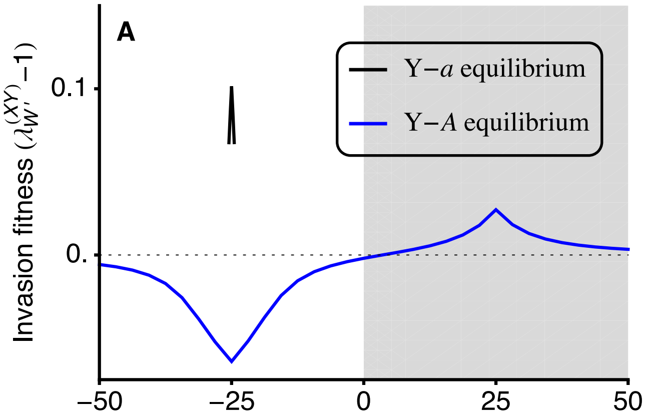

Panel B - tight linkage; overdominance in males and directional selection for a in females

Parameters

```
x = -5;
m = 5;

tryt = 0;
tryFAA = 0.85;
tryFAa = 1;
tryFaa = 1.05;
tryMAA = 0.75;
tryMAa = 1;
tryMaa = 0.75;
tryMA = 1 + tryt;
tryMa = 1;
tryFA = 1;
tryFa = 1;
tryMdA = 0.5;
tryFdA = 0.5;

xplotmin = -10;
xplotmax = 10;
xplotinterval = 5;
npoints = 32;
```

Plot

```
positionplotB =
 (*Legended[*)
 Show[

  (*region of tighter linkage*)
  RegionPlot[x > 0, {x, xplotmin, xplotmax}, {y, yplotmin, yplotmax},
   PlotStyle → {LightGray},
```

```
  BoundaryStyle → None,
  PlotRange → {yplotmin, yplotmax},
  AxesOrigin → {xplotmin, yplotmin},
  Frame → {True, True, False, False}
 ],

 (*neo-W into XY*)
 ListPlot[
  Table[{a, invasionplotXY2[{tryFAA, tryFAa, tryFaa, tryMAA, tryMAa, tryMaa,
      tryFA, tryFa, tryMA, tryMa, tryFdA, tryMdA, setr[x, a, m], setR[x, a, m],
      setρ[x, a, m]}, sieveXY[tryFAA, tryFAa, tryFaa, tryMAA, tryMAa, tryMaa,
       tryFA, tryFa, tryMA, tryMa, tryFdA, tryMdA, setr[x, a, m]][[1]]]},
   {a, xplotmin, xplotmax, (xplotmax - xplotmin) / npoints}],
  Joined → True,
  PlotRange → {yplotmin, All},
  PlotStyle → Directive[Blue, Thickness[lwd]],
  AxesOrigin → {xplotmin, yplotmin},
  Frame → {True, True, False, False}
 ],
 ListPlot[Table[{a,
    invasionplotXY2[{tryFAA, tryFAa, tryFaa, tryMAA, tryMAa, tryMaa, tryFA, tryFa,
      tryMA, tryMa, tryFdA, tryMdA, setr[x, a, m], setR[x, a, m], setρ[x, a, m]},
     sieveXY[tryFAA, tryFAa, tryFaa, tryMAA, tryMAa, tryMaa, tryFA, tryFa,
       tryMA, tryMa, tryFdA, tryMdA, setr[x, a, m]][[2]]]}, {a, -6, -4, 0.25}],
  Joined → True,
  PlotRange → {yplotmin, All},
  PlotStyle → Directive[Black, Thickness[lwd]],
  AxesOrigin → {xplotmin, yplotmin},
  Frame → {True, True, False, False}
 ],

 Plot[0, {x, xplotmin, xplotmax}, PlotStyle → {Black, Dotted}],

 plotstyle[xplotmin, xplotmax, xplotmin, xplotmax,
  xplotinterval, yplotmin, yplotmax, ytickmin, ytickmax, yint],
 Epilog → {
   Text[Style["B", Bold], Scaled@letpos]
  },
 PlotRangeClipping → False
](*,
 Placed[
  LineLegend[
   {Directive[Thickness[0.005],Black],
    Directive[Thickness[0.005],Blue]
   },
   {Style["Ya equilibrium",16],
    Style["YA equilibrium",16]
   },
   (*LegendFunction→"Frame",*)
   LegendLayout→"Column"
  ],
  Scaled@{0.72,0.65}
 ]
]*)
```

Part::partw : Part 2 of {{0.0283897, 0.0313209, 0.959256, 0.5}} does not exist. ≫

Part::partw : Part 2 of {{0.0213283, 0.0235138, 0.969581, 0.5}} does not exist. ≫

Part::partw : Part 2 of {{0.0213283, 0.0235138, 0.969581, 0.5}} does not exist. ≫

General::stop : Further output of Part::partw will be suppressed during this calculation. ≫

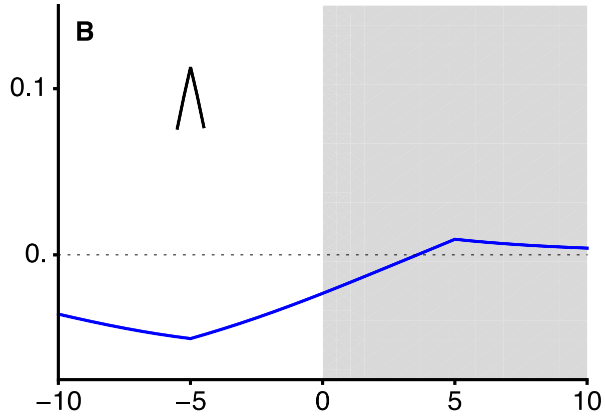

Panel C - loose linkage; overdominance in both sexes

Parameters

```
x = -25;
m = 25;

tryt = 0;
tryFAA = 0.6;
tryFAa = 1;
tryFaa = 0.6;
tryMAA = 0.7;
tryMAa = 1;
tryMaa = 0.5;
tryMA = 1 + tryt;
tryMa = 1;
tryFA = 1;
tryFa = 1;
tryMdA = 0.5;
tryFdA = 0.5;

xplotmin = -50;
xplotmax = 50;
xplotinterval = 25;
npoints = 36;
```

Plot

```
positionplotC =
 Show[
```

```
(*region of tighter linkage*)
RegionPlot[x > 0, {x, xplotmin, xplotmax}, {y, yplotmin, yplotmax},
 PlotStyle → {LightGray},
 BoundaryStyle → None,
 PlotRange → {yplotmin, yplotmax},
 AxesOrigin → {xplotmin, yplotmin},
 Frame → {True, True, False, False}
],

(*neo-W into XY*)
ListPlot[
 Table[{a, invasionplotXY2[{tryFAA, tryFAa, tryFaa, tryMAA, tryMAa, tryMaa,
     tryFA, tryFa, tryMA, tryMa, tryFdA, tryMdA, setr[x, a, m], setR[x, a, m],
     setρ[x, a, m]}, sieveXY[tryFAA, tryFAa, tryFaa, tryMAA, tryMAa, tryMaa,
      tryFA, tryFa, tryMA, tryMa, tryFdA, tryMdA, setr[x, a, m]][[1]]]},
  {a, xplotmin, xplotmax, (xplotmax - xplotmin) / npoints}],
 Joined → True,
 PlotRange → {yplotmin, All},
 PlotStyle → Directive[Blue, Thickness[lwd]],
 AxesOrigin → {xplotmin, yplotmin},
 Frame → {True, True, False, False}
],
ListPlot[Table[{a,
   invasionplotXY2[{tryFAA, tryFAa, tryFaa, tryMAA, tryMAa, tryMaa, tryFA, tryFa,
     tryMA, tryMa, tryFdA, tryMdA, setr[x, a, m], setR[x, a, m], setρ[x, a, m]},
    sieveXY[tryFAA, tryFAa, tryFaa, tryMAA, tryMAa, tryMaa, tryFA, tryFa,
      tryMA, tryMa, tryFdA, tryMdA, setr[x, a, m]][[2]]]}, {a, -27, -23, 0.25}],
 Joined → True,
 PlotRange → {yplotmin, All},
 PlotStyle → Directive[Black, Thickness[lwd]],
 AxesOrigin → {xplotmin, yplotmin},
 Frame → {True, True, False, False}
],

Plot[0, {x, xplotmin, xplotmax}, PlotStyle → {Black, Dotted}],

plotstyle[xplotmin, xplotmax, xplotmin, xplotmax,
 xplotinterval, yplotmin, yplotmax, ytickmin, ytickmax, yint],
FrameLabel → {"Chromosomal position of selected locus (cM)", ""},
Epilog → {
  Text[Style["C", Bold], Scaled@letpos],
  Rotate[
   Text[Style["Invasion fitness (λ_{W'}^{(XY)}-1)"], Scaled@{-0.15, 0.5}], 90 Degree]
  (*,Text[Style["tighter sex-linkage",14],{m,yplotmax*0.9}],*)
  , Text["ancestral SDR", {x, yplotmin * 0.75}],
  Text["novel SDR", {m, yplotmin * 0.75}],
  Arrow[{{x, yplotmin * 0.5}, {x, 0}}],
  Arrow[{{m, yplotmin * 0.5}, {m, 0}}]
 },
PlotRangeClipping → False

]
```

Part::partw : Part 2 of {{0.40879, 0.351282, 0.936149, 0.5}} does not exist. ≫

Part::partw : Part 2 of {{0.404106, 0.344002, 0.944328, 0.5}} does not exist. ≫

Part::partw : Part 2 of {{0.399301, 0.336588, 0.952469, 0.5}} does not exist. ≫

General::stop : Further output of Part::partw will be suppressed during this calculation. ≫

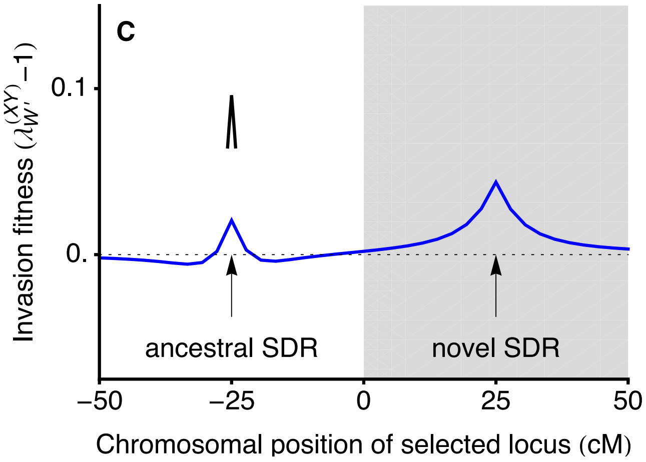

Panel D - tight linkage and overdominance in both sexes

Parameters

```
x = -5;
m = 5;

tryt = 0;
tryFAA = 0.6;
tryFAa = 1;
tryFaa = 0.6;
tryMAA = 0.7;
tryMAa = 1;
tryMaa = 0.5;
tryMA = 1 + tryt;
tryMa = 1;
tryFA = 1;
tryFa = 1;
tryMdA = 0.5;
tryFdA = 0.5;

xplotmin = -10;
xplotmax = 10;
xplotinterval = 5;
npoints = 36;
```

Plot

Printed by Wolfram Mathematica Student Edition

```
positionplotD =
 Show[

  (*region of tighter linkage*)
  RegionPlot[x > 0, {x, xplotmin, xplotmax}, {y, yplotmin, yplotmax},
   PlotStyle → {LightGray},
   BoundaryStyle → None,
   PlotRange → {yplotmin, yplotmax},
   AxesOrigin → {xplotmin, yplotmin},
   Frame → {True, True, False, False}
  ],

  (*neo-W into XY*)
  ListPlot[
   Table[{a, invasionplotXY2[{tryFAA, tryFAa, tryFaa, tryMAA, tryMAa, tryMaa,
        tryFA, tryFa, tryMA, tryMa, tryFdA, tryMdA, setr[x, a, m], setR[x, a, m],
        setρ[x, a, m]}, sieveXY[tryFAA, tryFAa, tryFaa, tryMAA, tryMAa, tryMaa,
         tryFA, tryFa, tryMA, tryMa, tryFdA, tryMdA, setr[x, a, m]][[1]]]},
     {a, xplotmin, xplotmax, (xplotmax - xplotmin) / npoints}],
   Joined → True,
   PlotRange → {yplotmin, All},
   PlotStyle → Directive[Blue, Thickness[lwd]],
   AxesOrigin → {xplotmin, yplotmin},
   Frame → {True, True, False, False}
  ],
  ListPlot[Table[{a,
      invasionplotXY2[{tryFAA, tryFAa, tryFaa, tryMAA, tryMAa, tryMaa, tryFA, tryFa,
        tryMA, tryMa, tryFdA, tryMdA, setr[x, a, m], setR[x, a, m], setρ[x, a, m]},
       sieveXY[tryFAA, tryFAa, tryFaa, tryMAA, tryMAa, tryMaa, tryFA, tryFa,
         tryMA, tryMa, tryFdA, tryMdA, setr[x, a, m]][[2]]]}, {a, -6, -4, 0.25}],
   Joined → True,
   PlotRange → {yplotmin, All},
   PlotStyle → Directive[Black, Thickness[lwd]],
   AxesOrigin → {xplotmin, yplotmin},
   Frame → {True, True, False, False}
  ],

  Plot[0, {x, xplotmin, xplotmax}, PlotStyle → {Black, Dotted}],

  plotstyle[xplotmin, xplotmax, xplotmin, xplotmax,
   xplotinterval, yplotmin, yplotmax, ytickmin, ytickmax, yint],
  FrameLabel → {"Chromosomal position of selected locus (cM)", ""},
  Epilog → {
    Text[Style["D", Bold], Scaled@letpos],
    (*Rotate[Text[Style["Invasion fitness (λ-1)",14],Scaled@ylabpos],90 Degree],
    Text[Style["tighter sex-linkage",14],{m,yplotmax*0.9}],*)
    Text["ancestral SDR", {x, yplotmin * 0.75}],
    Text["novel SDR", {m, yplotmin * 0.75}],
    Arrow[{{x, yplotmin * 0.5}, {x, 0}}],
    Arrow[{{m, yplotmin * 0.5}, {m, 0}}]
   },
  PlotRangeClipping → False
 ]
```

Part::partw : Part 2 of {{0.389312, 0.321357, 0.968596, 0.5}} does not exist. ≫

Part::partw : Part 2 of {{0.389312, 0.321357, 0.968596, 0.5}} does not exist. ≫

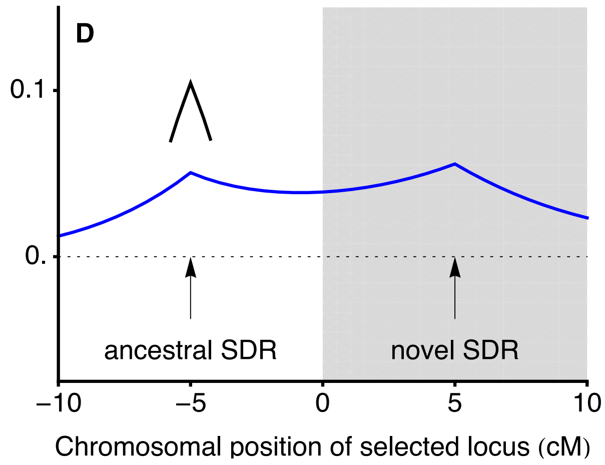

Combined

```
GraphicsGrid[{{positionplotA, positionplotB}, {positionplotC, positionplotD}},
 Spacings → {-36, -30},
 Epilog → {
   Rotate[Text[Style["overdominance in males", 14 * posplotscale,
      Bold, FontFamily → "Helvetica"], Scaled@{1.5, 1.05}], -π / 2],
   Rotate[Text[Style["overdominance in both sexes", 14 * posplotscale,
      Bold, FontFamily → "Helvetica"], Scaled@{1.5, 0.1}], -π / 2],
   Rotate[Text[Style["loose linkage", 14 * posplotscale, Bold,
      FontFamily → "Helvetica"], Scaled@{0.05, 1.475}], 0],
   Rotate[Text[Style["tight linkage", 14 * posplotscale, Bold,
      FontFamily → "Helvetica"], Scaled@{1.05, 1.475}], 0]
  }
]

Export[plotdir <> "PositionPlot_Overdominance.eps", %(*//rasterTrick*)];
```

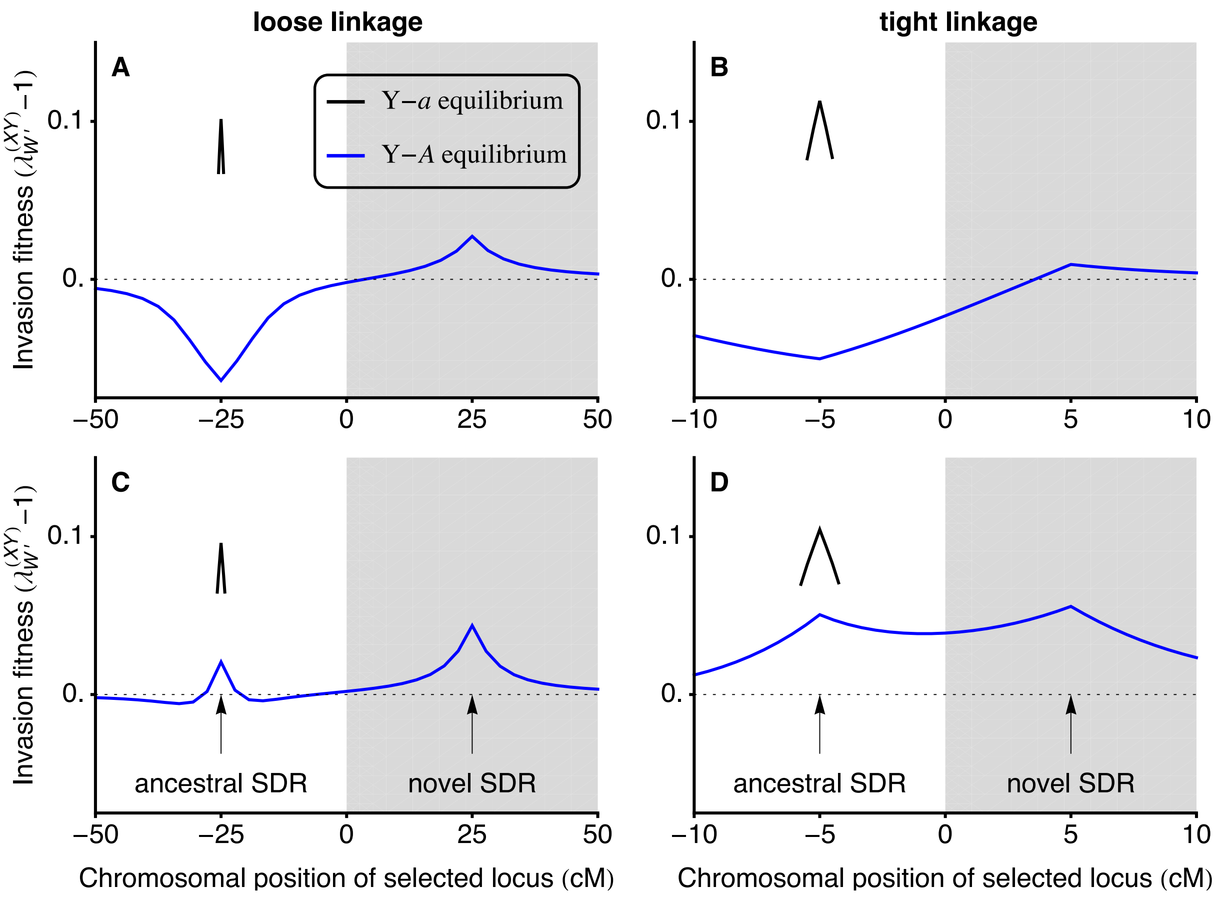

Figure S.2 - neo-W invasion dynamics for tight linkage (r ~ 0) with overdominance in males, no haploid selection

Plotting parameters

```
Pad = {{50, 20}, {50, 30}}; (*space around plot*)
yplotmin = -0.075;
yplotmax = 0.15;
yplotinterval = 0.02;
ylabpos = {-0.13, 0.5}; (*relative location of y axis label position*)
```

Parameters

```
tryFAA2 = 0.85;
tryFAa2 = 1;
tryFaa2 = 1.05;
tryMAA2 = 0.75;
tryMAa2 = 1;
tryMaa2 = 0.75;
trywAf2 = 1;
trywaf2 = 1;
trywAm2 = 1;
trywam2 = 1;
tryαf2 = 0.5;
tryαm2 = 0.5;
tryr2 = 0.005;
tryR2 = 0;
subpar2 = {FAA -> tryFAA2, FAa -> tryFAa2, Faa -> tryFaa2, MAA -> tryMAA2,
   MAa -> tryMAa2, Maa -> tryMaa2, wAf -> trywAf2, waf -> trywaf2,
   wAm -> trywAm2, wam -> trywam2, αf -> tryαf2, αm -> tryαm2};

tryFAA3 = 0.6;
tryFAa3 = 1;
tryFaa3 = 0.6;
tryMAA3 = 0.7;
tryMAa3 = 1;
tryMaa3 = 0.5;
trywAf3 = 1;
trywaf3 = 1;
trywAm3 = 1;
trywam3 = 1;
tryαf3 = 0.5;
tryαm3 = 0.5;
tryr3 = 0.005;
tryR3 = 0;
subpar3 = {FAA -> tryFAA3, FAa -> tryFAa3, Faa -> tryFaa3, MAA -> tryMAA3,
   MAa -> tryMAa3, Maa -> tryMaa3, wAf -> trywAf3, waf -> trywaf3,
   wAm -> trywAm3, wam -> trywam3, αf -> tryαf3, αm -> tryαm3};
```

Extra parameters

```
modvec = {0, 0, 1, 1, 0, 0, 1, 1, 0, 0, 0, 0, 0, 0, 0, 0};

loglinearplotINVoptions = {Frame → {{True, False}, {True, False}},
   PlotRangeClipping → False,
   ImagePadding → Pad,
   FrameStyle -> Directive[Black, Thickness[lwd]],
   FrameTicksStyle → {{Directive[Black, Thickness[lwd], FontColor → Black],
      Directive[Black, Thickness[lwd]]},
     {Directive[Black, Thickness[lwd]], Directive[Black, Thickness[lwd]]}},
   FrameTicks → {{{5, 5, {0, 0.01}}, {10, 10, {0, 0.01}}, {50, 50, {0, 0.01}},
      {100, 100, {0, 0.01}}, {500, 500, {0, 0.01}}, {1000, 1000, {0, 0.01}},
      {5000, 5000, {0, 0.01}}}, Table[{y, y, ticksize}, {y, 0, 0.5, 0.25}]},
   BaseStyle → {FontFamily → "Helvetica", FontSize → 12},
   ImageSize → {xsize, xsize aspectratio },
   AspectRatio → aspectratio,
   Axes → True};

startplot = 5;
endtime = 5000;
tryR = 0;
trypm = 0.01;
tryk = 1;
```

Panel A - a favoured in females, r = 0

```
tryr2 = 0;

tryR = 0.001;
param =
  {tryFAA2, tryFAa2, tryFaa2, tryMAA2, tryMAa2, tryMaa2, trywAf2, trywaf2, trywAm2,
   trywam2, tryαf2, tryαm2, tryr2, tryR, tryr2 (1 - tryR) + tryR (1 - tryr2), tryk};
run[0] = generation[param, startgen[
    sieveXY[tryFAA2, tryFAa2, tryFaa2, tryMAA2, tryMAa2, tryMaa2, trywAf2,
      trywaf2, trywAm2, trywam2, tryαf2, tryαm2, tryr2][[2]], trypm]];
For[time = 1, time ≤ endtime, time++, run[time] = generation[param, run[time - 1]]
]
MODtab2Blackr0 =
  Table[{Round[Exp[exptime]], generation[param, run[Round[Exp[exptime]]]].modvec},
   {exptime, Log[startplot], Log[endtime], 0.1}];
MOD2Blackr0 = ListLogLinearPlot[MODtab2Blackr0, Joined → True,
   PlotRange → {{startplot, endtime}, {0, 0.5}},
   PlotStyle → {Black, Thick}, loglinearplotINVoptions];
```

Solve::ratnz : Solve was unable to solve the system with inexact coefficients. The answer was obtained by solving a corresponding exact system and numericizing the result. ≫

```
tryR = 0.02;
param =
  {tryFAA2, tryFAa2, tryFaa2, tryMAA2, tryMAa2, tryMaa2, trywAf2, trywaf2, trywAm2,
   trywam2, tryαf2, tryαm2, tryr2, tryR, tryr2 (1 - tryR) + tryR (1 - tryr2), tryk};
run[0] = generation[param, startgen[
     sieveXY[tryFAA2, tryFAa2, tryFaa2, tryMAA2, tryMAa2, tryMaa2, trywAf2,
       trywaf2, trywAm2, trywam2, tryαf2, tryαm2, tryr2][[2]], trypm]];
For[time = 1, time ≤ endtime, time++, run[time] = generation[param, run[time - 1]]
]
MODtab2Redr0 =
  Table[{Round[Exp[exptime]], generation[param, run[Round[Exp[exptime]]]].modvec},
   {exptime, Log[startplot], Log[endtime], 0.1}];
MOD2Redr0 = ListLogLinearPlot[MODtab2Redr0, Joined → True,
   PlotRange → {{startplot, endtime}, {0, 0.5}},
   PlotStyle → {Red, Thick}, loglinearplotINVoptions];

tryR = 0.1;
param =
  {tryFAA2, tryFAa2, tryFaa2, tryMAA2, tryMAa2, tryMaa2, trywAf2, trywaf2, trywAm2,
   trywam2, tryαf2, tryαm2, tryr2, tryR, tryr2 (1 - tryR) + tryR (1 - tryr2), tryk};
run[0] = generation[param, startgen[
     sieveXY[tryFAA2, tryFAa2, tryFaa2, tryMAA2, tryMAa2, tryMaa2, trywAf2,
       trywaf2, trywAm2, trywam2, tryαf2, tryαm2, tryr2][[2]], trypm]];
For[time = 1, time ≤ endtime, time++, run[time] = generation[param, run[time - 1]]
]
MODtab2Bluer0 =
  Table[{Round[Exp[exptime]], generation[param, run[Round[Exp[exptime]]]].modvec},
   {exptime, Log[startplot], Log[endtime], 0.1}];
MOD2Bluer0 = ListLogLinearPlot[MODtab2Bluer0, Joined → True,
   PlotRange → {{startplot, endtime}, {0, 0.5}},
   PlotStyle → {Blue, Thick}, loglinearplotINVoptions];

tryR = 0.5;
param =
  {tryFAA2, tryFAa2, tryFaa2, tryMAA2, tryMAa2, tryMaa2, trywAf2, trywaf2, trywAm2,
   trywam2, tryαf2, tryαm2, tryr2, tryR, tryr2 (1 - tryR) + tryR (1 - tryr2), tryk};
run[0] = generation[param, startgen[
     sieveXY[tryFAA2, tryFAa2, tryFaa2, tryMAA2, tryMAa2, tryMaa2, trywAf2,
       trywaf2, trywAm2, trywam2, tryαf2, tryαm2, tryr2][[2]], trypm]];
For[time = 1, time ≤ endtime, time++, run[time] = generation[param, run[time - 1]]
]
MODtab2Greenr0 =
  Table[{Round[Exp[exptime]], generation[param, run[Round[Exp[exptime]]]].modvec},
   {exptime, Log[startplot], Log[endtime], 0.1}];
MOD2Greenr0 = ListLogLinearPlot[MODtab2Greenr0, Joined → True,
   PlotRange → {{startplot, endtime}, {0, 0.5}},
   PlotStyle → {Green, Thick}, loglinearplotINVoptions];
```

```
partA = Legended[Show[MOD2Blackr0, MOD2Redr0, MOD2Bluer0, MOD2Greenr0,
   FrameLabel →
    {{Style["neo-W frequency", FontSize → 14], ""}, {Style["", FontSize → 14], ""}},
   Epilog → {
     Text[Style["A", 14, Bold], Scaled@letpos],
     Text[Style["r = 0", 14, Bold], Scaled@{0.5, 1.02}]
    }],
  Placed[
   LineLegend[
    {Directive[Thickness[0.1], Black],
     Directive[Thickness[0.1], Red],
     Directive[Thickness[0.1], Blue],
     Directive[Thickness[0.1], Green]
    },
    {Style["R=0.001", 14],
     Style["R=0.02", 14],
     Style["R=0.1", 14],
     Style["R=0.5", 14]
    },
    LegendFunction → "Frame",
    LegendLayout → "Column"
   ],
   Scaled@{0.825, 0.4}
  ]
 ]
```

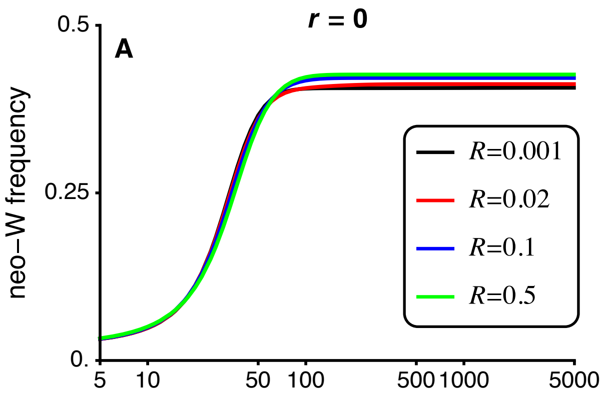

Panel B - a favoured in females, r = 0.005

```
tryr2 = 0.005;
```

```
tryR = 0.001;
param =
  {tryFAA2, tryFAa2, tryFaa2, tryMAA2, tryMAa2, tryMaa2, trywAf2, trywaf2, trywAm2,
   trywam2, tryαf2, tryαm2, tryr2, tryR, tryr2 (1 - tryR) + tryR (1 - tryr2), tryk};
run[0] = generation[param, startgen[
     sieveXY[tryFAA2, tryFAa2, tryFaa2, tryMAA2, tryMAa2, tryMaa2, trywAf2,
       trywaf2, trywAm2, trywam2, tryαf2, tryαm2, tryr2][[2]], trypm]];
For[time = 1, time ≤ endtime, time++, run[time] = generation[param, run[time - 1]]
]
MODtab2Black =
  Table[{Round[Exp[exptime]], generation[param, run[Round[Exp[exptime]]]].modvec},
   {exptime, Log[startplot], Log[endtime], 0.1}];
MOD2Black = ListLogLinearPlot[MODtab2Black, Joined → True,
   PlotRange → {{startplot, endtime}, {0, 0.5}},
   PlotStyle → {Black, Thick}, loglinearplotINVoptions];

tryR = 0.02;
param =
  {tryFAA2, tryFAa2, tryFaa2, tryMAA2, tryMAa2, tryMaa2, trywAf2, trywaf2, trywAm2,
   trywam2, tryαf2, tryαm2, tryr2, tryR, tryr2 (1 - tryR) + tryR (1 - tryr2), tryk};
run[0] = generation[param, startgen[
     sieveXY[tryFAA2, tryFAa2, tryFaa2, tryMAA2, tryMAa2, tryMaa2, trywAf2,
       trywaf2, trywAm2, trywam2, tryαf2, tryαm2, tryr2][[2]], trypm]];
For[time = 1, time ≤ endtime, time++, run[time] = generation[param, run[time - 1]]
]
MODtab2Red =
  Table[{Round[Exp[exptime]], generation[param, run[Round[Exp[exptime]]]].modvec},
   {exptime, Log[startplot], Log[endtime], 0.1}];
MOD2Red = ListLogLinearPlot[MODtab2Red, Joined → True,
   PlotRange → {{startplot, endtime}, {0, 0.5}},
   PlotStyle → {Red, Thick}, loglinearplotINVoptions];

tryR = 0.1;
param =
  {tryFAA2, tryFAa2, tryFaa2, tryMAA2, tryMAa2, tryMaa2, trywAf2, trywaf2, trywAm2,
   trywam2, tryαf2, tryαm2, tryr2, tryR, tryr2 (1 - tryR) + tryR (1 - tryr2), tryk};
run[0] = generation[param, startgen[
     sieveXY[tryFAA2, tryFAa2, tryFaa2, tryMAA2, tryMAa2, tryMaa2, trywAf2,
       trywaf2, trywAm2, trywam2, tryαf2, tryαm2, tryr2][[2]], trypm]];
For[time = 1, time ≤ endtime, time++, run[time] = generation[param, run[time - 1]]
]
MODtab2Blue =
  Table[{Round[Exp[exptime]], generation[param, run[Round[Exp[exptime]]]].modvec},
   {exptime, Log[startplot], Log[endtime], 0.1}];
MOD2Blue = ListLogLinearPlot[MODtab2Blue, Joined → True,
   PlotRange → {{startplot, endtime}, {0, 0.5}},
   PlotStyle → {Blue, Thick}, loglinearplotINVoptions];
```

```
tryR = 0.5;
param =
  {tryFAA2, tryFAa2, tryFaa2, tryMAA2, tryMAa2, tryMaa2, trywAf2, trywaf2, trywAm2,
   trywam2, tryαf2, tryαm2, tryr2, tryR, tryr2 (1 - tryR) + tryR (1 - tryr2), tryk};
run[0] = generation[param, startgen[
     sieveXY[tryFAA2, tryFAa2, tryFaa2, tryMAA2, tryMAa2, tryMaa2, trywAf2,
       trywaf2, trywAm2, trywam2, tryαf2, tryαm2, tryr2][[2]], trypm]];
For[time = 1, time ≤ endtime, time++, run[time] = generation[param, run[time - 1]]
]
MODtab2Green =
  Table[{Round[Exp[exptime]], generation[param, run[Round[Exp[exptime]]]].modvec},
   {exptime, Log[startplot], Log[endtime], 0.1}];
MOD2Green = ListLogLinearPlot[MODtab2Green, Joined → True,
   PlotRange → {{startplot, endtime}, {0, 0.5}},
   PlotStyle → {Green, Thick}, loglinearplotINVoptions];

partB = Show[MOD2Black, MOD2Red, MOD2Blue, MOD2Green,
  (*FrameLabel→{{Style["neo-W frequency",FontSize→14],""},
    {Style["Generation",FontSize→14],""}},*)
  Epilog → {
    Text[Style["B", 14, Bold], Scaled@letpos],
    Text[Style["r = 0.005", 14, Bold], Scaled@{0.5, 1.02}]
   }]
```

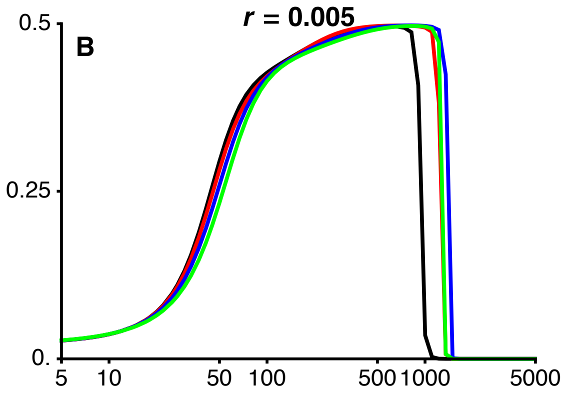

Panel C - overdominance in females, r = 0

```
tryr3 = 0;
```

```
tryR = 0.001;
param =
  {tryFAA3, tryFAa3, tryFaa3, tryMAA3, tryMAa3, tryMaa3, trywAf3, trywaf3, trywAm3,
   trywam3, tryαf3, tryαm3, tryr3, tryR, tryr3 (1 - tryR) + tryR (1 - tryr3), tryk};
run[0] = generation[param, startgen[
     sieveXY[tryFAA3, tryFAa3, tryFaa3, tryMAA3, tryMAa3, tryMaa3, trywAf3,
       trywaf3, trywAm3, trywam3, tryαf3, tryαm3, tryr3][[2]], trypm]];
For[time = 1, time ≤ endtime, time++, run[time] = generation[param, run[time - 1]]
]
MODtab3Blackr0 =
  Table[{Round[Exp[exptime]], generation[param, run[Round[Exp[exptime]]]].modvec},
   {exptime, Log[startplot], Log[endtime], 0.1}];
MOD3Blackr0 = ListLogLinearPlot[MODtab3Blackr0, Joined → True,
   PlotRange → {{startplot, endtime}, {0, 0.5}},
   PlotStyle → {Black, Thick}, loglinearplotINVoptions];
```

Solve::ratnz : Solve was unable to solve the system with inexact coefficients. The answer was obtained by solving a corresponding exact system and numericizing the result. ≫

```
tryR = 0.02;
param =
  {tryFAA3, tryFAa3, tryFaa3, tryMAA3, tryMAa3, tryMaa3, trywAf3, trywaf3, trywAm3,
   trywam3, tryαf3, tryαm3, tryr3, tryR, tryr3 (1 - tryR) + tryR (1 - tryr3), tryk};
run[0] = generation[param, startgen[
     sieveXY[tryFAA3, tryFAa3, tryFaa3, tryMAA3, tryMAa3, tryMaa3, trywAf3,
       trywaf3, trywAm3, trywam3, tryαf3, tryαm3, tryr3][[2]], trypm]];
For[time = 1, time ≤ endtime, time++, run[time] = generation[param, run[time - 1]]
]
MODtab3Redr0 =
  Table[{Round[Exp[exptime]], generation[param, run[Round[Exp[exptime]]]].modvec},
   {exptime, Log[startplot], Log[endtime], 0.1}];
MOD3Redr0 = ListLogLinearPlot[MODtab3Redr0, Joined → True,
   PlotRange → {{startplot, endtime}, {0, 0.5}},
   PlotStyle → {Red, Thick}, loglinearplotINVoptions];

tryR = 0.1;
param =
  {tryFAA3, tryFAa3, tryFaa3, tryMAA3, tryMAa3, tryMaa3, trywAf3, trywaf3, trywAm3,
   trywam3, tryαf3, tryαm3, tryr3, tryR, tryr3 (1 - tryR) + tryR (1 - tryr3), tryk};
run[0] = generation[param, startgen[
     sieveXY[tryFAA3, tryFAa3, tryFaa3, tryMAA3, tryMAa3, tryMaa3, trywAf3,
       trywaf3, trywAm3, trywam3, tryαf3, tryαm3, tryr3][[2]], trypm]];
For[time = 1, time ≤ endtime, time++, run[time] = generation[param, run[time - 1]]
]
MODtab3Bluer0 =
  Table[{Round[Exp[exptime]], generation[param, run[Round[Exp[exptime]]]].modvec},
   {exptime, Log[startplot], Log[endtime], 0.1}];
MOD3Bluer0 = ListLogLinearPlot[MODtab3Bluer0, Joined → True,
   PlotRange → {{startplot, endtime}, {0, 0.5}},
   PlotStyle → {Blue, Thick}, loglinearplotINVoptions];
```

```
tryR = 0.5;
param =
  {tryFAA3, tryFAa3, tryFaa3, tryMAA3, tryMAa3, tryMaa3, trywAf3, trywaf3, trywAm3,
   trywam3, tryαf3, tryαm3, tryr3, tryR, tryr3 (1 - tryR) + tryR (1 - tryr3), tryk};
run[0] = generation[param, startgen[
     sieveXY[tryFAA3, tryFAa3, tryFaa3, tryMAA3, tryMAa3, tryMaa3, trywAf3,
       trywaf3, trywAm3, trywam3, tryαf3, tryαm3, tryr3][[2]], trypm]];
For[time = 1, time ≤ endtime, time++, run[time] = generation[param, run[time - 1]]
]
MODtab3Greenr0 =
  Table[{Round[Exp[exptime]], generation[param, run[Round[Exp[exptime]]]].modvec},
   {exptime, Log[startplot], Log[endtime], 0.1}];
MOD3Greenr0 = ListLogLinearPlot[MODtab3Greenr0, Joined → True,
   PlotRange → {{startplot, endtime}, {0, 0.5}},
   PlotStyle → {Green, Thick}, loglinearplotINVoptions];

partC = Show[MOD3Blackr0, MOD3Redr0, MOD3Bluer0, MOD3Greenr0,
  FrameLabel → {{Style["neo-W frequency", FontSize → 14], ""},
    {Style["Generation", FontSize → 14], ""}},
  Epilog → {
    Text[Style["C", 14, Bold], Scaled@letpos](*,
    Text[Style["r=0",14,Bold],Scaled@{0.5,1.02}]*)
   }]
```

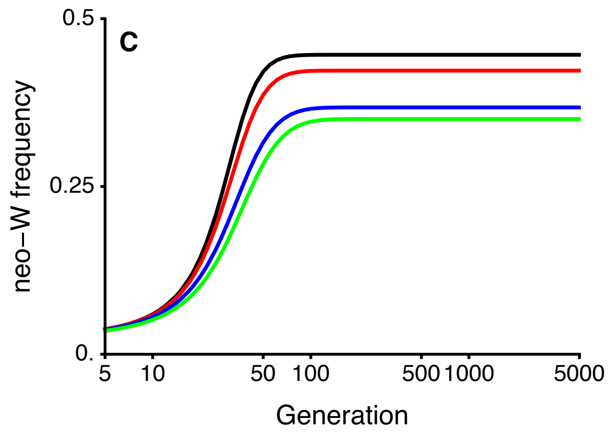

Panel D - overdominance in females, r = 0.005

```
tryr3 = 0.005;
```

```
tryR = 0.001;
param =
  {tryFAA3, tryFAa3, tryFaa3, tryMAA3, tryMAa3, tryMaa3, trywAf3, trywaf3, trywAm3,
   trywam3, tryαf3, tryαm3, tryr3, tryR, tryr3 (1 - tryR) + tryR (1 - tryr3), tryk};
run[0] = generation[param, startgen[
     sieveXY[tryFAA3, tryFAa3, tryFaa3, tryMAA3, tryMAa3, tryMaa3, trywAf3,
       trywaf3, trywAm3, trywam3, tryαf3, tryαm3, tryr3][[2]], trypm]];
For[time = 1, time ≤ endtime, time++, run[time] = generation[param, run[time - 1]]
]
MODtab3Black =
  Table[{Round[Exp[exptime]], generation[param, run[Round[Exp[exptime]]]].modvec},
   {exptime, Log[startplot], Log[endtime], 0.1}];
MOD3Black = ListLogLinearPlot[MODtab3Black, Joined → True,
   PlotRange → {{startplot, endtime}, {0, 0.5}},
   PlotStyle → {Black, Thick}, loglinearplotINVoptions];

tryR = 0.02;
param =
  {tryFAA3, tryFAa3, tryFaa3, tryMAA3, tryMAa3, tryMaa3, trywAf3, trywaf3, trywAm3,
   trywam3, tryαf3, tryαm3, tryr3, tryR, tryr3 (1 - tryR) + tryR (1 - tryr3), tryk};
run[0] = generation[param, startgen[
     sieveXY[tryFAA3, tryFAa3, tryFaa3, tryMAA3, tryMAa3, tryMaa3, trywAf3,
       trywaf3, trywAm3, trywam3, tryαf3, tryαm3, tryr3][[2]], trypm]];
For[time = 1, time ≤ endtime, time++, run[time] = generation[param, run[time - 1]]
]
MODtab3Red =
  Table[{Round[Exp[exptime]], generation[param, run[Round[Exp[exptime]]]].modvec},
   {exptime, Log[startplot], Log[endtime], 0.1}];
MOD3Red = ListLogLinearPlot[MODtab3Red, Joined → True,
   PlotRange → {{startplot, endtime}, {0, 0.5}},
   PlotStyle → {Red, Thick}, loglinearplotINVoptions];

tryR = 0.1;
param =
  {tryFAA3, tryFAa3, tryFaa3, tryMAA3, tryMAa3, tryMaa3, trywAf3, trywaf3, trywAm3,
   trywam3, tryαf3, tryαm3, tryr3, tryR, tryr3 (1 - tryR) + tryR (1 - tryr3), tryk};
run[0] = generation[param, startgen[
     sieveXY[tryFAA3, tryFAa3, tryFaa3, tryMAA3, tryMAa3, tryMaa3, trywAf3,
       trywaf3, trywAm3, trywam3, tryαf3, tryαm3, tryr3][[2]], trypm]];
For[time = 1, time ≤ endtime, time++, run[time] = generation[param, run[time - 1]]
]
MODtab3Blue =
  Table[{Round[Exp[exptime]], generation[param, run[Round[Exp[exptime]]]].modvec},
   {exptime, Log[startplot], Log[endtime], 0.1}];
MOD3Blue = ListLogLinearPlot[MODtab3Blue, Joined → True,
   PlotRange → {{startplot, endtime}, {0, 0.5}},
   PlotStyle → {Blue, Thick}, loglinearplotINVoptions];
```

```
tryR = 0.5;
param =
  {tryFAA3, tryFAa3, tryFaa3, tryMAA3, tryMAa3, tryMaa3, trywAf3, trywaf3, trywAm3,
   trywam3, tryαf3, tryαm3, tryr3, tryR, tryr3 (1 - tryR) + tryR (1 - tryr3), tryk};
run[0] = generation[param, startgen[
    sieveXY[tryFAA3, tryFAa3, tryFaa3, tryMAA3, tryMAa3, tryMaa3, trywAf3,
      trywaf3, trywAm3, trywam3, tryαf3, tryαm3, tryr3][[2]], trypm]];
For[time = 1, time ≤ endtime, time++, run[time] = generation[param, run[time - 1]]
]
MODtab3Green =
  Table[{Round[Exp[exptime]], generation[param, run[Round[Exp[exptime]]]].modvec},
   {exptime, Log[startplot], Log[endtime], 0.1}];
MOD3Green = ListLogLinearPlot[MODtab3Green, Joined → True,
   PlotRange → {{startplot, endtime}, {0, 0.5}},
   PlotStyle → {Green, Thick}, loglinearplotINVoptions];
```

```
partD = Show[MOD3Black, MOD3Red, MOD3Blue, MOD3Green,
  FrameLabel →
   {{Style["", FontSize → 14], ""}, {Style["Generation", FontSize → 14], ""}},
  Epilog → {
    Text[Style["D", 14, Bold], Scaled@letpos](*,
    Text[Style["r=0.005",14,Bold],Scaled@{0.5,1.02}]*)
   }]
```

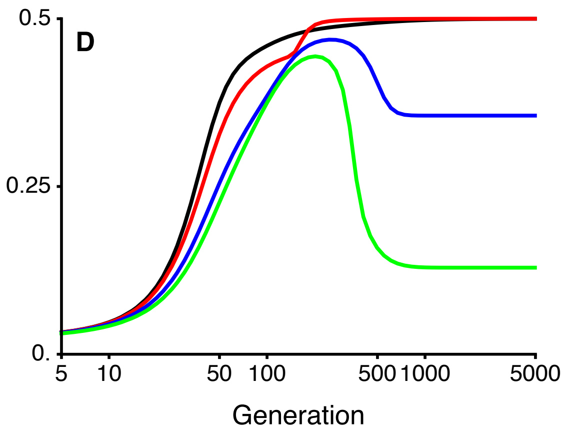

All panels

```
GraphicsGrid[{{partA, partB}, {partC, partD}}, Spacings → -30]
```

```
Export[plotdir <> "Temporal_Overdominance.eps", %(*//rasterTrick*)];
```

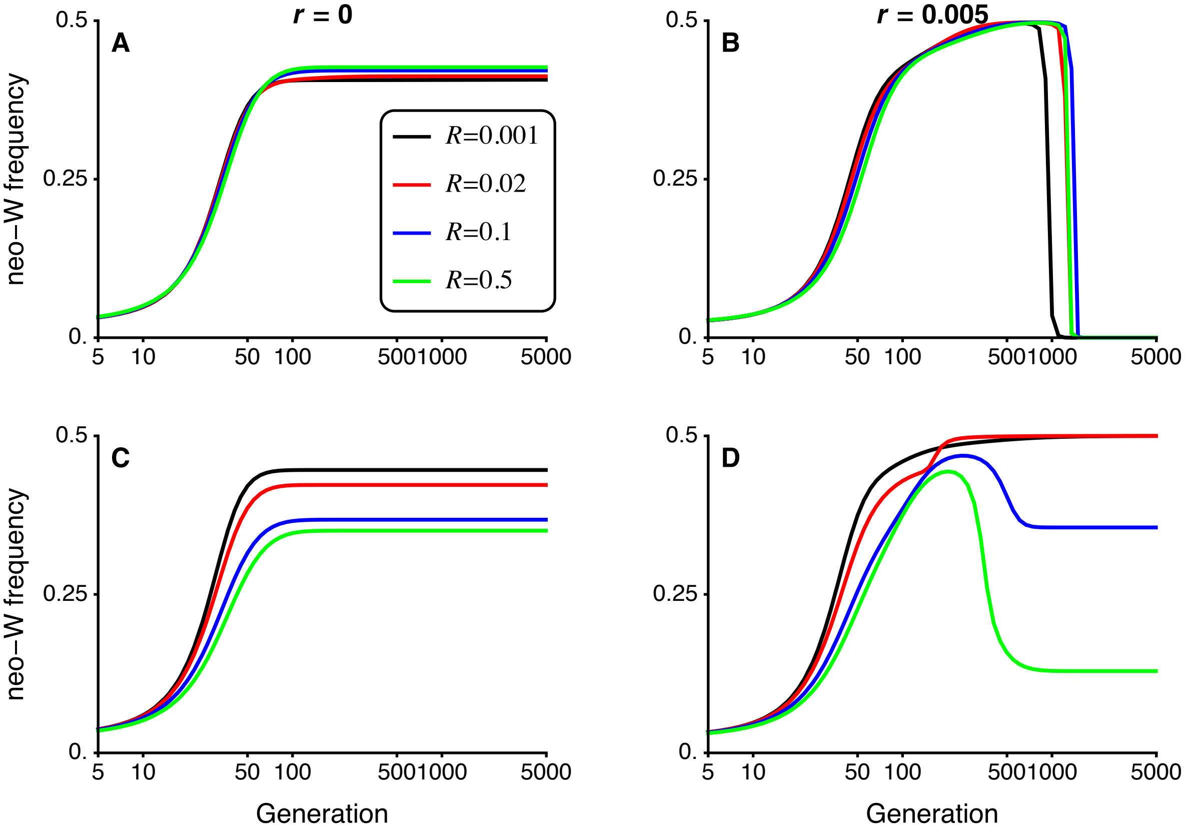

Figure S.3 - sexual antagonism and haploid selection allows less closely linked neo-W to invade

Plotting parameters

```
Pad = {{50, 30}, {50, 30}}; (*space around plot*)
yplotmin = -0.075;
yplotmax = 0.15;
yplotinterval = 0.02;
ylabpos = {-0.15, 0.5}; (*relative location of y axis label position*)
```

```
startplot = 5;
endtime = 5000;
tryR = 0;
trypm = 0.01;
tryk = 1;

loglinearplotINVoptions = {Frame → {{True, False}, {True, False}},
   FrameStyle -> Directive[Black, Thickness[lwd]],
   FrameTicksStyle → {{Directive[Black, Thickness[lwd], FontColor → Black],
      Directive[Black, Thickness[lwd]]},
     {Directive[Black, Thickness[lwd]], Directive[Black, Thickness[lwd]]}},
   FrameTicks → {{{5, 5, {0, 0.01}}, {10, 10, {0, 0.01}}, {50, 50, {0, 0.01}},
      {100, 100, {0, 0.01}}, (*{500,500,{0,0.01}},*){1000, 1000, {0, 0.01}}
      (*,{5000,5000,{0,0.01}}*)}, Table[{y, y, ticksize}, {y, 0, 0.5, 0.5}]},
   BaseStyle → {FontFamily → "Helvetica", FontSize → 12},
   ImageSize → {xsize, xsize aspectratio },
   AspectRatio → aspectratio,
   Axes → True,
   FrameLabel → {{Style["neo-W frequency", FontSize → 14], ""},
     {Style["Generation", FontSize → 14], ""}}};

loglinearplotRλoptions = {Frame → {{True, False}, {True, False}},
   ImageSize → {xsize, xsize aspectratio },
   AspectRatio → aspectratio,
   PlotRangePadding → 0,
   BaseStyle → {FontFamily → "Helvetica", FontSize → 14},
   ImagePadding → Pad,
   PlotRangeClipping → False,
   FrameStyle -> Directive[Black, Thickness[lwd]],
   FrameTicksStyle → {{Directive[Black, Thickness[lwd], FontColor → Black],
      Directive[Black, Thickness[lwd]]},
     {Directive[Black, Thickness[lwd]], Directive[Black, Thickness[lwd]]}},
   FrameTicks → {{{0.001, 0.001, {0, 0.01}}, {0.005, "r", {0, 0.01}},
      {0.01, 0.01, {0, 0.01}}, {0.05, 0.05, {0, 0.01}}, {0.5, 0.5, {0, 0.01}}},
     Table[{y, y, ticksize}, {y, -0.1, 0.2, 0.1}]},
   BaseStyle → {FontFamily → "Helvetica", FontSize → 12},
   ImageSize → {xsize, xsize aspectratio },
   AspectRatio → aspectratio,
   Axes → True,
   FrameLabel →
    {{"", ""}, {Style["Rate of recombination (R)", FontSize → 14], ""}}};

letposB = {0.05, 0.95};
```

Panel A

Parameters

```
x = -5;
m = 5;

tryFAA = 1 + 1 / 20;
tryFAa = 1;
tryFaa = 1 - 3 / 20;
tryMAA = 1 - 3 / 20;
tryMAa = 1;
tryMaa = 1 + 1 / 20;
trywAf = 1;
trywaf = 1;
trywAm = 1;
trywam = 1;
tryαf = 1 / 2;
tryαm = 1 / 2 - 8 / 100;

xplotmin = -10;
xplotmax = 10;
xplotinterval = 5;
npoints = 28;

tryr = 0.005;
```

Plot

```
positionplot =
 Show[

  (*region of tighter linkage*)
  RegionPlot[x > 0, {x, xplotmin, xplotmax}, {y, yplotmin, yplotmax},
   PlotStyle → {LightGray},
   BoundaryStyle → None,
   PlotRange → {yplotmin, yplotmax},
   AxesOrigin → {xplotmin, yplotmin},
   Frame → {True, True, False, False}
  ],

  (*neo-W into XY*)
  ListPlot[Table[{a,
      invasionplotXY[tryFAA, tryFAa, tryFaa, tryMAA, tryMAa, tryMaa, trywAf, trywaf,
       trywAm, trywam, tryαf, tryαm, setr[x, a, m], setR[x, a, m], setρ[x, a, m]]},
     {a, xplotmin, xplotmax, (xplotmax - xplotmin) / npoints}],
   Joined → True,
   PlotRange → {yplotmin, All},
   PlotStyle → Directive[Black, Thickness[lwd]],
   AxesOrigin → {xplotmin, yplotmin},
   Frame → {True, True, False, False}
  ],
```

```
Plot[0, {x, xplotmin, xplotmax}, PlotStyle → {Black, Dotted}],

PlotRange → {{xplotmin, xplotmax}, {yplotmin, yplotmax}},
ImageSize → {xsize, xsize aspectratio },
AspectRatio → aspectratio,
PlotRangePadding → 0,
FrameTicks → {Table[{x, x, ticksize}, {x, xplotmin, xplotmax, xplotinterval}],
  Table[{y, y, ticksize}, {y, -0.1, 0.2, 0.1}]},
FrameTicksStyle → {{Directive[Black, Thickness[lwd]],
   Directive[Black, Thickness[lwd]]},
  {Directive[Black, Thickness[lwd]], Directive[Black, Thickness[lwd]]}},
FrameStyle → {{Black, Thickness[lwd]}, {Black, Thickness[lwd]}, None, None},
FrameLabel → {"Chromosomal position of selected locus (cM)", ""},
BaseStyle → {FontFamily → "Helvetica", FontSize → 14},
ImagePadding → Pad,
Epilog → {
  Text[Style["A", 14 * 4 / 5, Bold], Scaled@letposB],
  Rotate[Text[Style[(*"Invasion fitness (λ-1)"*)"", 14 * 4 / 5],
    Scaled@ylabpos], 90 Degree],
  Rotate[Text[Style["Invasion fitness (λ_W'^(XY)-1)", 14], Scaled@ylabpos],
   90 Degree],
  (*Text[Style["tighter sex-linkage",14],{m,yplotmax*0.9}],*)
  Text["ancestral SDR", {x, yplotmin * 0.75}],
  Text["novel SDR", {m, yplotmin * 0.75}],
  Arrow[{{x, yplotmin * 0.5}, {x, 0}}],
  Arrow[{{m, yplotmin * 0.5}, {m, 0}}]
 },
PlotRangeClipping → False

]

(*Export[plotdir<>"PositionPlotB.pdf",%];*)
```

Solve::ratnz : Solve was unable to solve the system with inexact coefficients. The answer was obtained by solving a corresponding exact system and numericizing the result. ≫

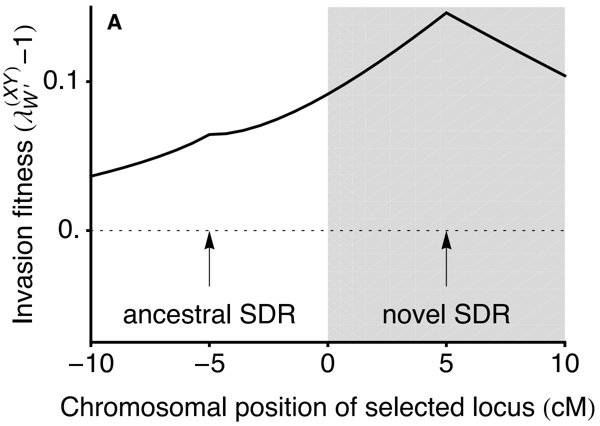

Panel B

Plot

```
subpar1 =
  {FAA → tryFAA, FAa → tryFAa, Faa → tryFaa, MAA → tryMAA, MAa → tryMAa, Maa → tryMaa,
   wAf → trywAf, waf → trywaf, wAm → trywAm, wam → trywam, αf → tryαf, αm → tryαm};

tab = Table[
   equil = sieveXY[tryFAA, tryFAa, tryFaa, tryMAA, tryMAa,
      tryMaa, trywAf, trywaf, trywAm, trywam, tryαf, tryαm, tryr][[1]];
   λ /. Solve[(λ^2 + (- (λmA1 + λma1) + (χmA1 + χma1)) λ + (λmA1 - χmA1) (λma1 - χma1) -
              χmA1 χma1 /. reverse /. pAveM → (1 - q) pXm + q pYm /. subpar1 /. R → 0.5^b /.
          {pXf → equil[[1]], pXm → equil[[2]], pYm → equil[[3]],
           q → equil[[4]]}) == 0, λ], {b, 10, 1, -1 / 2}];
eigen1 = Transpose[Join[{Table[0.5^b, {b, 10, 1, -1 / 2}]},
     {Transpose[tab][[1]] - 1}]];
eigen2 = Transpose[Join[{Table[0.5^b, {b, 10, 1, -1 / 2}]},
     {Transpose[tab][[2]] - 1}]];
plot1 = Show[
  ListLogLinearPlot[eigen2, Joined → True, PlotStyle → {Thick, Black},
   PlotRange → {{0.0008, 0.5}, {yplotmin, yplotmax}},
   loglinearplotRλoptions]
 ]
```

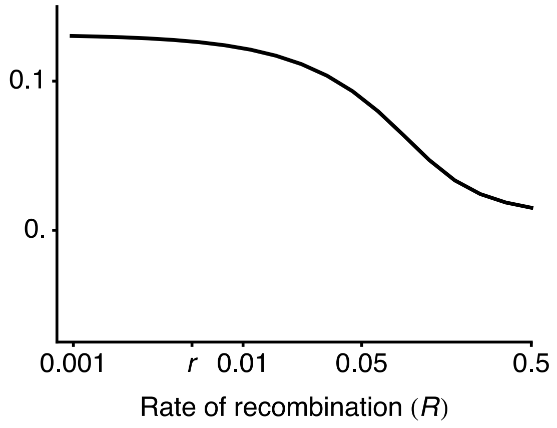

```
temp = Show[
  ListLogLinearPlot[{{0.0008, yplotmax}, {tryr, yplotmax}}, Joined → True,
   PlotStyle → {Black, Thin}, Filling → yplotmin, FillingStyle → LightGray,
   loglinearplotRλoptions, PlotRange -> {{0.0008, 0.5}, {yplotmin, yplotmax}}],
  plot1,
  ListLogLinearPlot[{{tryr, yplotmax}, {tryr, yplotmin}},
   Joined → True, PlotStyle → {Black, Thin}],
  ListLogLinearPlot[{{0.001, -0.01}, {0.001, 0.01}},
   Joined → True, PlotStyle → {Black, Thick}],
  ListLogLinearPlot[{{0.02, -0.01}, {0.02, 0.01}},
   Joined → True, PlotStyle → {Red, Thick}],
  ListLogLinearPlot[{{0.1, -0.01}, {0.1, 0.01}},
   Joined → True, PlotStyle → {Blue, Thick}],
  ListLogLinearPlot[{{0.5, -0.01}, {0.5, 0.01}},
   Joined → True, PlotStyle → {Green, Thick}],
  GridLines → {{0.005}, {1}}(*,
  GridLinesStyle→Black*)]
```

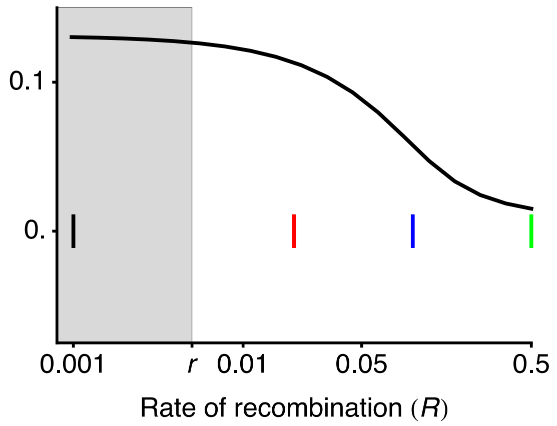

Ensure this is consistent with the full characteristic polynomial result

```
plot2 = ListLogLinearPlot[Table[{0.5^b,
      invasionplotXY[tryFAA, tryFAa, tryFaa, tryMAA, tryMAa, tryMaa, trywAf, trywaf,
       trywAm, trywam, tryαf, tryαm, tryr, 0.5^b, tryr (1 - 0.5^b) + 0.5^b (1 - tryr)]},
     {b, 10, 1, -1 / 2}], Joined → True, PlotStyle → {Thick, Red, Dashed},
    PlotRange → {{0.0008, 0.5}, {yplotmin, yplotmax}},
    loglinearplotRλoptions];
Show[plot1, plot2]
```

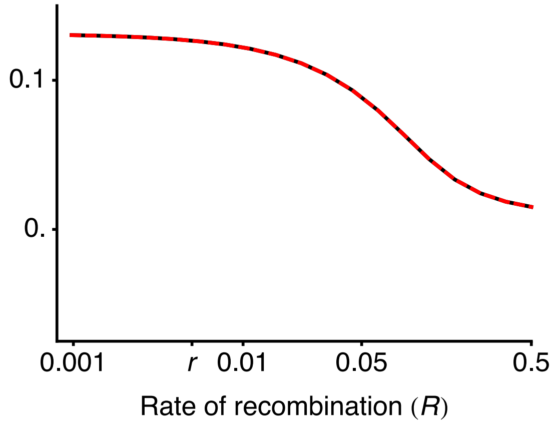

Panel C

```
modvec = {0, 0, 1, 1, 0, 0, 1, 1, 0, 0, 0, 0, 0, 0, 0, 0};
```

```
tryR = 0.001;
param = {tryFAA, tryFAa, tryFaa, tryMAA, tryMAa, tryMaa, trywAf, trywaf, trywAm,
   trywam, tryαf, tryαm, tryr, tryR, tryr (1 - tryR) + tryR (1 - tryr), tryk};
run[0] = generation[param, startgen[sieveXY[tryFAA, tryFAa, tryFaa, tryMAA, tryMAa,
       tryMaa, trywAf, trywaf, trywAm, trywam, tryαf, tryαm, tryr][[1]], trypm]];
For[time = 1, time ≤ endtime, time++, run[time] = generation[param, run[time - 1]]
]
MODtab1Black =
  Table[{Round[Exp[exptime]], generation[param, run[Round[Exp[exptime]]]].modvec},
   {exptime, Log[startplot], Log[endtime], 0.1}];
MOD1Black = ListLogLinearPlot[MODtab1Black, Joined → True,
  PlotRange → {{startplot, endtime}, {0, 0.5}},
  PlotStyle → {Black, Thick}, loglinearplotINVoptions]
```

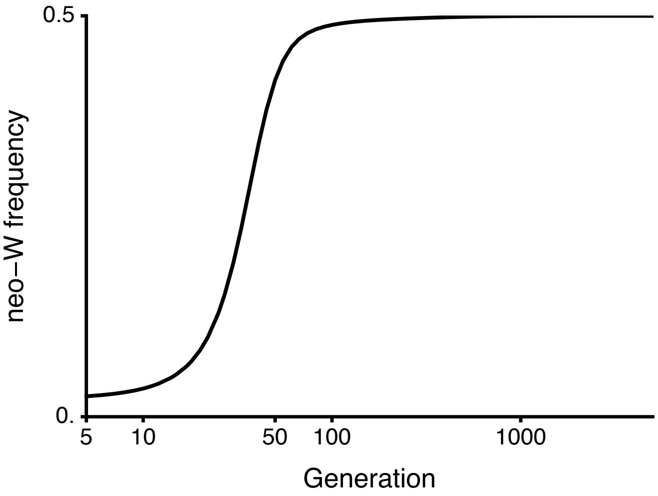

```
tryR = 0.02;
param = {tryFAA, tryFAa, tryFaa, tryMAA, tryMAa, tryMaa, trywAf, trywaf, trywAm,
   trywam, tryαf, tryαm, tryr, tryR, tryr (1 - tryR) + tryR (1 - tryr), tryk};
run[0] = generation[param, startgen[sieveXY[tryFAA, tryFAa, tryFaa, tryMAA, tryMAa,
      tryMaa, trywAf, trywaf, trywAm, trywam, tryαf, tryαm, tryr][[1]], trypm]];
For[time = 1, time ≤ endtime, time++, run[time] = generation[param, run[time - 1]]
]
MODtab1Red =
  Table[{Round[Exp[exptime]], generation[param, run[Round[Exp[exptime]]]].modvec},
   {exptime, Log[startplot], Log[endtime], 0.1}];
MOD1Red = ListLogLinearPlot[MODtab1Red, Joined → True,
  PlotRange → {{startplot, endtime}, {0, 0.5}},
  PlotStyle → {Red, Thick}, loglinearplotINVoptions]
```

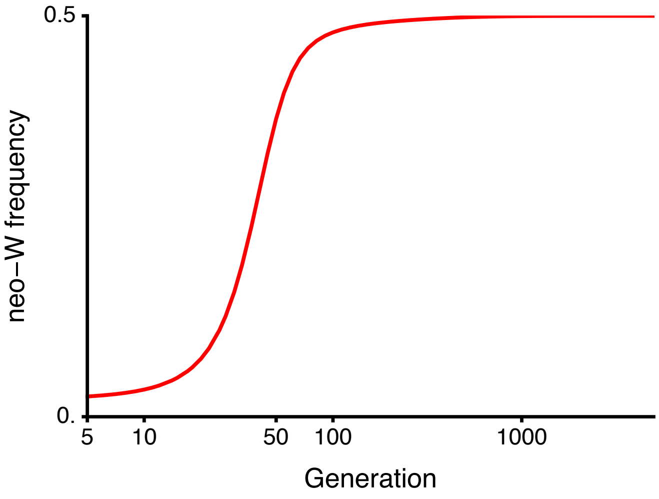

```
tryR = 0.1;
param = {tryFAA, tryFAa, tryFaa, tryMAA, tryMAa, tryMaa, trywAf, trywaf, trywAm,
   trywam, tryαf, tryαm, tryr, tryR, tryr (1 - tryR) + tryR (1 - tryr), tryk};
run[0] = generation[param, startgen[sieveXY[tryFAA, tryFAa, tryFaa, tryMAA, tryMAa,
       tryMaa, trywAf, trywaf, trywAm, trywam, tryαf, tryαm, tryr][[1]], trypm]];
For[time = 1, time ≤ endtime, time++, run[time] = generation[param, run[time - 1]]
]
MODtab1Blue =
  Table[{Round[Exp[exptime]], generation[param, run[Round[Exp[exptime]]]].modvec},
   {exptime, Log[startplot], Log[endtime], 0.1}];
MOD1Blue = ListLogLinearPlot[MODtab1Blue, Joined → True,
  PlotRange → {{startplot, endtime}, {0, 0.5}},
  PlotStyle → {Blue, Thick}, loglinearplotINVoptions]
```

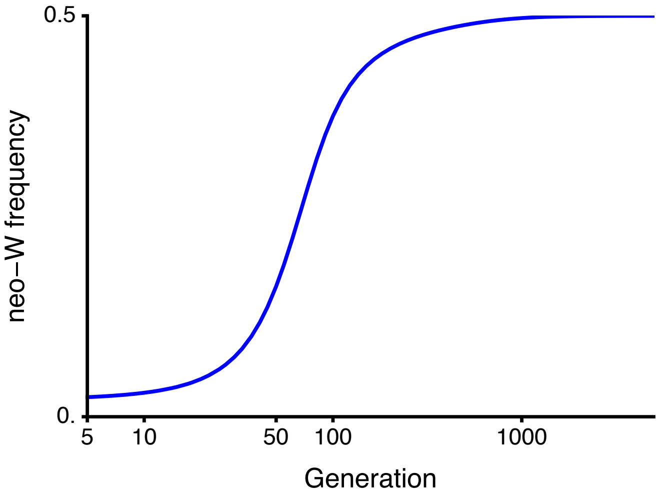

```
tryR = 0.5;
param = {tryFAA, tryFAa, tryFaa, tryMAA, tryMAa, tryMaa, trywAf, trywaf, trywAm,
   trywam, tryαf, tryαm, tryr, tryR, tryr (1 - tryR) + tryR (1 - tryr), tryk};
run[0] = generation[param, startgen[sieveXY[tryFAA, tryFAa, tryFaa, tryMAA, tryMAa,
      tryMaa, trywAf, trywaf, trywAm, trywam, tryαf, tryαm, tryr][[1]], trypm]];
For[time = 1, time ≤ endtime, time++, run[time] = generation[param, run[time - 1]]
]
MODtab1Green =
  Table[{Round[Exp[exptime]], generation[param, run[Round[Exp[exptime]]]].modvec},
   {exptime, Log[startplot], Log[endtime], 0.1}];
MOD1Green = ListLogLinearPlot[MODtab1Green, Joined → True,
  PlotRange → {{startplot, endtime}, {0, 0.5}},
  PlotStyle → {Green, Thick}, loglinearplotINVoptions]
```

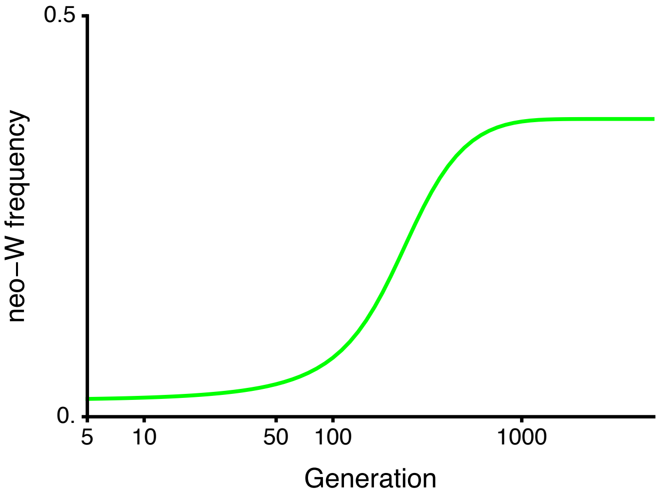

All panels

```
GraphicsRow[{
  positionplot,
  Show[temp, Graphics[Text[Style["B", 14, Bold], Scaled@letposB]],
   LogLinearPlot[0, {x, 0.0008, 0.5}, PlotStyle → {Black, Dotted}]],
  Show[MOD1Black, MOD1Red, MOD1Blue, MOD1Green,
   Graphics[Text[Style["C", 14, Bold], Scaled@letposB]],
   Epilog → Rotate[Text[Style["neo-W frequency", 14], Scaled@ylabpos], 90 Degree],
   ImagePadding → Pad]
 },
 Spacings → -60
]

Export[plotdir <> "PositionPlot_SexAntagTighter_MaleDrive.eps",
  Magnify[%(*//rasterTrick*), 8 / 10]];
```

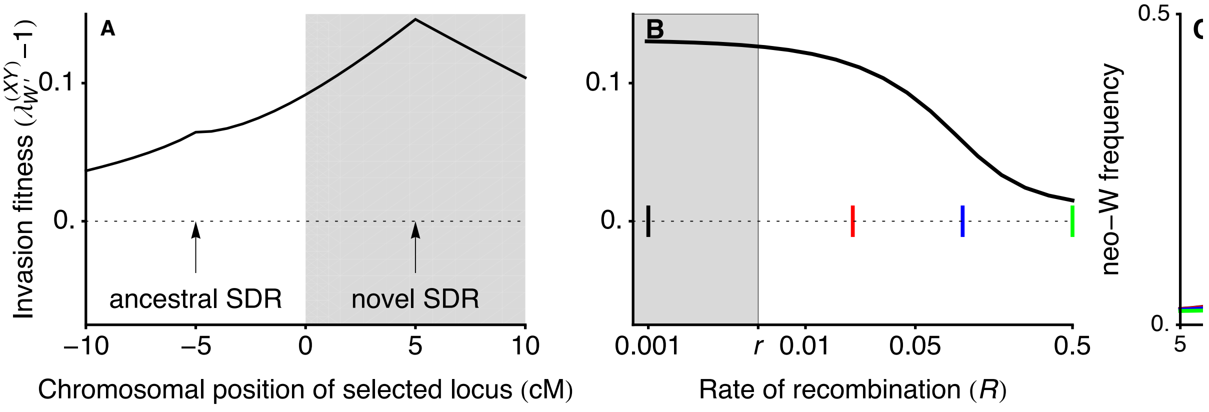

Figure S.4 - neo-W haplotype invasion with meiotic drive in males

Panel A - A favoured in females, a drives in males

Parameters

```
params = {
    wAm → 1, wam → 1, wAf → 1, waf → 1,
    αm → 1 / 2 - 8 / 100,
    αf → 1 / 2,
    MAa → 1, FAa → 1,
    Faa → 1 - 3 / 20,
    FAA → 1 + 1 / 20
  };
```

Making the sex-ratio

```
freqMale /. equilB0 /. params // N
```

```
0.58
```

No recombination eigenvalues

```
WAinvA = λmA1 /. reverse /. pAveM → (1 - q) pXm + q pYm /. equilA0 /. params // Simplify;
WainvA = λma1 /. reverse /. pAveM → (1 - q) pXm + q pYm /. equilA0 /. params // Simplify;
WAinvB = λmA1 /. reverse /. pAveM → (1 - q) pXm + q pYm /. equilB0 /. params // Simplify;
WainvB = λma1 /. reverse /. pAveM → (1 - q) pXm + q pYm /. equilB0 /. params // Simplify;
```

Maximum absolute no recombination eigenvalue from the full characteristic polynomial

```
λWsolA =
  Max[Abs[λ /. Solve[0 == charpolyk1 /. r → 0 /. R → 0 /. ρ → 0 /. equilA0 /. params, λ] //
      Simplify]];
λWsolB = Max[Abs[λ /. Solve[0 == charpolyk1 /. r → 0 /. R → 0 /. ρ → 0 /. equilB0 /.
          params, λ] // Simplify]];
```

Plot

Region plots of invasion

```
(*neo-WA invades XY from equilA*)
plotWAinvA =
  RegionPlot[{
     (validcondA /. params) && (*valid*)
      (stabcondA /. Rf → 0 /. Rm → 0 /. equilA0 /. params) && (*internally stable*)
      1 < WAinvA (*invasion*)
    },
    {Maa, 0, 3 / 2}, {MAA, 0, 3 / 2},
    PlotStyle → {Gray, Opacity[0.5]},
    BoundaryStyle → None
  ];

(*neo-Wa invades XY from equilA*)
```

```
plotWainvA =
  RegionPlot[{
    (validcondA /. params) &&
     (stabcondA /. Rf → 0 /. Rm → 0 /. equilA0 /. params) &&
     1 < WainvA
   },
   {Maa, 0, 3 / 2}, {MAA, 0, 3 / 2},
   PlotStyle → {Gray, Opacity[0.5]},
   BoundaryStyle → None
  ];

(*neo-WA invades XY from equilB*)
plotWAinvB =
  RegionPlot[{
    (stabcondB /. Rf → 0 /. Rm → 0 /. equilB0 /. params) && (*internally stable*)
     1 < WAinvB(*invasion*)
   },
   {Maa, 0, 3 / 2}, {MAA, 0, 3 / 2},
   PlotStyle → {Gray, Opacity[0.5]},
   BoundaryStyle → None
  ];

(*neo-Wa invades XY from equilB*)
plotWainvB =
  RegionPlot[{
    (stabcondB /. Rf → 0 /. Rm → 0 /. equilB0 /. params) &&
     1 < WainvB
   },
   {Maa, 0, 3 / 2}, {MAA, 0, 3 / 2},
   PlotStyle → {Gray, Opacity[0.5]},
   BoundaryStyle → None
  ];

(*Ya equilibrium internally stable*)
plotYaStable =
  RegionPlot[{
    (stabcondA /. Rf → 0 /. Rm → 0 /. equilA0 /. params) ||
     (stabcondB /. Rf → 0 /. Rm → 0 /. equilB0 /. params)
   },
   {Maa, 0, 3 / 2}, {MAA, 0, 3 / 2},
   PlotStyle → White,
   BoundaryStyle → {Black, Thick}
  ];

(*Ya equilibrium internally unstable*)
plotYaUnstable =
  RegionPlot[{
    1 + 1 == 2
   },
   {Maa, 0, 3 / 2}, {MAA, 0, 3 / 2},
   PlotStyle → None,
   BoundaryStyle → None,
   Mesh → 50,
   MeshFunctions → {#1 - #2 &},
   MeshStyle → Black
```

```
plotA =
 Show[

  plotYaUnstable,
  plotYaStable,
  plotWAinvA,
  plotWainvA,
  plotWAinvB,
  plotWainvB,

  Graphics[{Thick, Black, Line[{{1.15, 0.3}, {1.275, 0.55}}]}],
  PlotRange → {{0, 1.5}, {0, 1.5}},
  ImageSize → {xsize, xsize},
  PlotRangePadding → 0,
  FrameTicks → {Table[{x, x, ticksize}, {x, 0, 1, 1}],
    Table[{y, y, ticksize}, {y, 0, 1, 1}], None, None},
  FrameTicksStyle → {{Directive[Black, Thickness[lwd]],
     Directive[Black, Thickness[lwd]]}, {Directive[Black, Thickness[lwd]],
     Directive[Black, Thickness[lwd], FontColor → White]}},
  FrameStyle → {{Black, Thickness[lwd]}, {Black, Thickness[lwd]},
    {Black, Thickness[lwd]}, {Black, Thickness[lwd]}},
  (*FrameLabel→{"Relative fitness of aa males",""},*)
  BaseStyle → {FontFamily → "Helvetica", FontSize → 14},
  ImagePadding → Pad,
  Epilog → {
    Text[Style["A", 14, Bold], Scaled@{0.05, 0.95}],
    Text[Style["Λ_{W'A}^(XY) >1 & Λ_{W'a}^(XY) >1", 14], {0.5, 0.5}],
    Text[Style["Λ_{W'A}^(XY) >1
&
Λ_{W'a}^(XY) <1", 12], {1.355, 0.725}, Background → White],
    Text[Style["*", 18], {1.05, 0.85}],
    Rotate[
     Text[Style["Relative fitness of AA males", 14], Scaled@ylabpos], 90 Degree]
   },
  PlotLabel → Style["A favoured in females", 16, Black, Bold],
  PlotRangeClipping → False
 ]
```

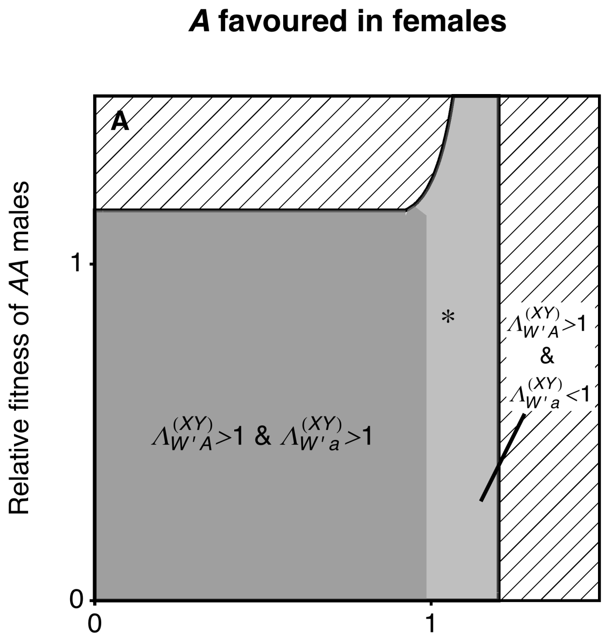

Notice that this is consistent with the solution from the full characteristic polynomial (but here we don't know which eigenvalue belongs to which haplotype)

```
(*neo-W invades XY from equilA*)
plotWinvA =
  RegionPlot[{
    (validcondA /. params) && (*valid*)
     (stabcondA /. Rf → 0 /. Rm → 0 /. equilA0 /. params) && (*internally stable*)
     1 < λWsolA (*invasion*)
   },
   {Maa, 0, 3 / 2}, {MAA, 0, 3 / 2},
   PlotStyle → {Gray, Opacity[0.5]},
   BoundaryStyle → None
  ];

(*neo-W invades XY from equilB*)
plotWinvB =
  RegionPlot[{
    (stabcondB /. Rf → 0 /. Rm → 0 /. equilB0 /. params) && (*internally stable*)
     1 < λWsolB(*invasion*)
   },
   {Maa, 0, 3 / 2}, {MAA, 0, 3 / 2},
   PlotStyle → {Gray, Opacity[0.5]},
   BoundaryStyle → None
  ];
```

```
Show[

 plotWinvA,
 plotWinvB,
 plotYaStable,

 PlotRange → {{0, 1.5}, {0, 1.5}},
 ImageSize → {xsize, xsize},
 PlotRangePadding → 0,
 FrameTicks → {Table[{x, x, ticksize}, {x, 0, 1, 1}],
   Table[{y, y, ticksize}, {y, 0, 1, 1}], None, None},
 FrameTicksStyle → {{Directive[Black, Thickness[lwd]],
    Directive[Black, Thickness[lwd]]}, {Directive[Black, Thickness[lwd]],
    Directive[Black, Thickness[lwd], FontColor → White]}},
 FrameStyle → {{Black, Thickness[lwd]}, {Black, Thickness[lwd]},
   {Black, Thickness[lwd]}, {Black, Thickness[lwd]}},
 FrameLabel → {"Relative fitness of aa males", ""},
 BaseStyle → {FontFamily → "Helvetica", FontSize → 14},
 ImagePadding → Pad,
 Epilog → {
   Text[Style["Ya equilibrium unstable", 14], {0.5, 1.25}],
   Rotate[
    Text[Style["Relative fitness of AA males", 14], Scaled@ylabpos], 90 Degree]
  },
 PlotLabel → Style["A favoured in females", 16, Black, Bold],
 PlotRangeClipping → False
]
```

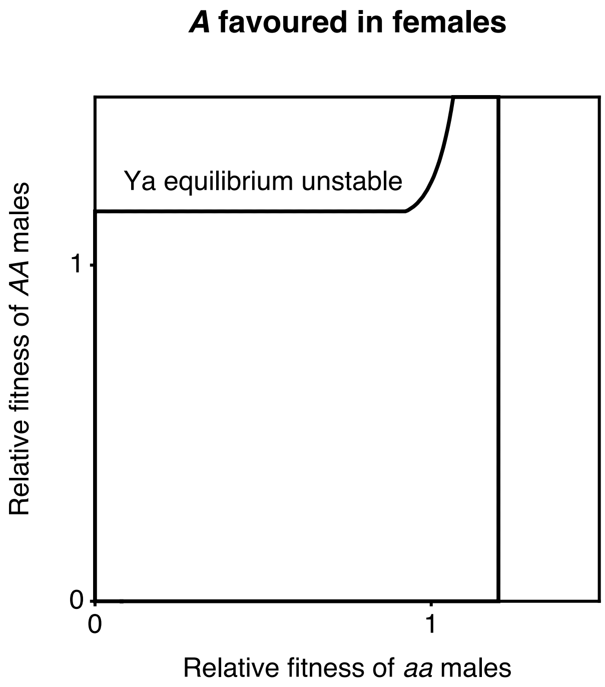

Panel B - a favoured in females, a drives in males

Parameters

```
params = {
   wAm → 1, wam → 1, wAf → 1, waf → 1,
   αm → 1 / 2 - 8 / 100,
   αf → 1 / 2,
   MAa → 1, FAa → 1,
   FAA → 1 - 3 / 20,
   Faa → 1 + 1 / 20
  };
```

No recombination eigenvalues

```
WAinvA = λmA1 /. reverse /. pAveM → (1 - q) pXm + q pYm /. equilA0 /. params // Simplify;
WainvA = λma1 /. reverse /. pAveM → (1 - q) pXm + q pYm /. equilA0 /. params // Simplify;
WAinvB = λmA1 /. reverse /. pAveM → (1 - q) pXm + q pYm /. equilB0 /. params // Simplify;
WainvB = λma1 /. reverse /. pAveM → (1 - q) pXm + q pYm /. equilB0 /. params // Simplify;
```

Maximum absolute no recombination eigenvalue from the full characteristic polynomial

```
λWsolA =
  Max[Abs[λ /. Solve[0 == charpolyk1 /. r → 0 /. R → 0 /. ρ → 0 /. equilA0 /. params, λ] //
      Simplify]];
λWsolB = Max[Abs[λ /. Solve[0 == charpolyk1 /. r → 0 /. R → 0 /. ρ → 0 /. equilB0 /.
         params, λ] // Simplify]];
```

Plot

Region plots of invasion

```
(*neo-WA invades XY from equilA*)
plotWAinvA =
  RegionPlot[{
     (validcondA /. params) && (*valid*)
      (stabcondA /. Rf → 0 /. Rm → 0 /. equilA0 /. params) && (*internally stable*)
      1 < WAinvA (*invasion*)
    },
    {Maa, 0, 3 / 2}, {MAA, 0, 3 / 2},
    PlotStyle → {Gray, Opacity[0.5]},
    BoundaryStyle → None
   ];

(*neo-Wa invades XY from equilA*)
plotWainvA =
  RegionPlot[{
     (validcondA /. params) &&
      (stabcondA /. Rf → 0 /. Rm → 0 /. equilA0 /. params) &&
      1 < WainvA
    },
    {Maa, 0, 3 / 2}, {MAA, 0, 3 / 2},
    PlotStyle → {Gray, Opacity[0.5]},
    BoundaryStyle → None
   ];
```

```
(*neo-WA invades XY from equilB*)
plotWAinvB =
  RegionPlot[{
    (stabcondB /. Rf → 0 /. Rm → 0 /. equilB0 /. params) && (*internally stable*)
     1 < WAinvB(*invasion*)
   },
   {Maa, 0, 3 / 2}, {MAA, 0, 3 / 2},
   PlotStyle → {Gray, Opacity[0.5]},
   BoundaryStyle → None
  ];

(*neo-Wa invades XY from equilB*)
plotWainvB =
  RegionPlot[{
    (stabcondB /. Rf → 0 /. Rm → 0 /. equilB0 /. params) &&
     1 < WainvB
   },
   {Maa, 0, 3 / 2}, {MAA, 0, 3 / 2},
   PlotStyle → {Gray, Opacity[0.5]},
   BoundaryStyle → None
  ];

(*Ya equilibrium internally stable*)
plotYaStable =
  RegionPlot[{
    (stabcondA /. Rf → 0 /. Rm → 0 /. equilA0 /. params) ||
     (stabcondB /. Rf → 0 /. Rm → 0 /. equilB0 /. params)
   },
   {Maa, 0, 3 / 2}, {MAA, 0, 3 / 2},
   PlotStyle → White,
   BoundaryStyle → {Black, Thick}
  ];

(*Ya equilibrium internally unstable*)
plotYaUnstable =
  RegionPlot[{
    1 < 5
   },
   {Maa, 0, 3 / 2}, {MAA, 0, 3 / 2},
   PlotStyle → None,
   BoundaryStyle → None,
   Mesh → 50,
   MeshFunctions → {#1 - #2 &},
   MeshStyle → Black
  ];
```

Printed by Wolfram Mathematica Student Edition

```
plotB =
 Show[

  plotYaUnstable,
  plotYaStable,
  plotWAinvA,
  plotWainvA,
  plotWAinvB,
  plotWainvB,

  Graphics[{Thick, Black, Line[{{0.9, 0.5}, {0.75, 0.25}}]}],
  PlotRange → {{0, 1.5}, {0, 1.5}},
  ImageSize → {xsize, xsize},
  PlotRangePadding → 0,
  FrameTicks → {Table[{x, x, ticksize}, {x, 0, 1, 1}],
    Table[{y, y, ticksize}, {y, 0, 1, 1}], None, None},
  FrameTicksStyle → {{Directive[Black, Thickness[lwd]],
     Directive[Black, Thickness[lwd]]}, {Directive[Black, Thickness[lwd]],
     Directive[Black, Thickness[lwd], FontColor → White]}},
  FrameStyle → {{Black, Thickness[lwd]}, {Black, Thickness[lwd]},
    {Black, Thickness[lwd]}, {Black, Thickness[lwd]}},
  (*FrameLabel→{"Relative fitness of aa males",""},*)
  BaseStyle → {FontFamily → "Helvetica", FontSize → 14},
  ImagePadding → Pad,
  Epilog → {
    Text[Style["B", 14, Bold], Scaled@{0.05, 0.95}],
    Text[Style["Λ_{W'A}^{(XY)} >1
&
Λ_{W'a}^{(XY)} >1", 14], {0.35, 0.5}],
    Text[Style["Λ_{W'A}^{(XY)} <1
&
Λ_{W'a}^{(XY)} >1", 14], {0.9, 0.5}, {-1.0, 0}, Background → White](*,
    Rotate[
     Text[Style["Relative fitness of AA males",14],Scaled@ylabpos],90 Degree]*)
   },
  PlotLabel → Style["a favoured in females", 16, Black, Bold],
  PlotRangeClipping → False
 ]
```

_a_ favoured in females

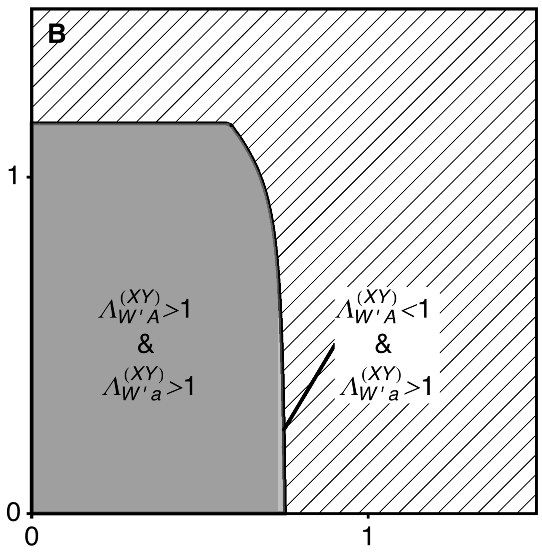

Notice that this is consistent with the solution from the full characteristic polynomial (but here we don’t know which eigenvalue belongs to which haplotype)

```
(*neo-W invades XY from equilA*)
plotWinvA =
  RegionPlot[{
    (validcondA /. params) && (*valid*)
     (stabcondA /. Rf → 0 /. Rm → 0 /. equilA0 /. params) && (*internally stable*)
     1 < λWsolA (*invasion*)
   },
   {Maa, 0, 3 / 2}, {MAA, 0, 3 / 2},
   PlotStyle → {Gray, Opacity[0.5]},
   BoundaryStyle → None
  ];

(*neo-W invades XY from equilB*)
plotWinvB =
  RegionPlot[{
    (stabcondB /. Rf → 0 /. Rm → 0 /. equilB0 /. params) && (*internally stable*)
     1 < λWsolB(*invasion*)
   },
   {Maa, 0, 3 / 2}, {MAA, 0, 3 / 2},
   PlotStyle → {Gray, Opacity[0.5]},
   BoundaryStyle → None
  ];
```

```
Show[

 plotWinvA,
 plotWinvB,
 plotYaStable,

 PlotRange → {{0, 1.5}, {0, 1.5}},
 ImageSize → {xsize, xsize},
 PlotRangePadding → 0,
 FrameTicks → {Table[{x, x, ticksize}, {x, 0, 1, 1}],
   Table[{y, y, ticksize}, {y, 0, 1, 1}], None, None},
 FrameTicksStyle → {{Directive[Black, Thickness[lwd]],
    Directive[Black, Thickness[lwd]]}, {Directive[Black, Thickness[lwd]],
    Directive[Black, Thickness[lwd], FontColor → White]}},
 FrameStyle → {{Black, Thickness[lwd]}, {Black, Thickness[lwd]},
   {Black, Thickness[lwd]}, {Black, Thickness[lwd]}},
 FrameLabel → {"Relative fitness of aa males", ""},
 BaseStyle → {FontFamily → "Helvetica", FontSize → 14},
 ImagePadding → Pad,
 Epilog → {
   Text[Style["Ya equilibrium unstable", 14], {0.5, 1.25}],
   Rotate[
    Text[Style["Relative fitness of AA males", 14], Scaled@ylabpos], 90 Degree]
  },
 PlotLabel → Style["a favoured in females", 16, Black, Bold],
 PlotRangeClipping → False
]
```

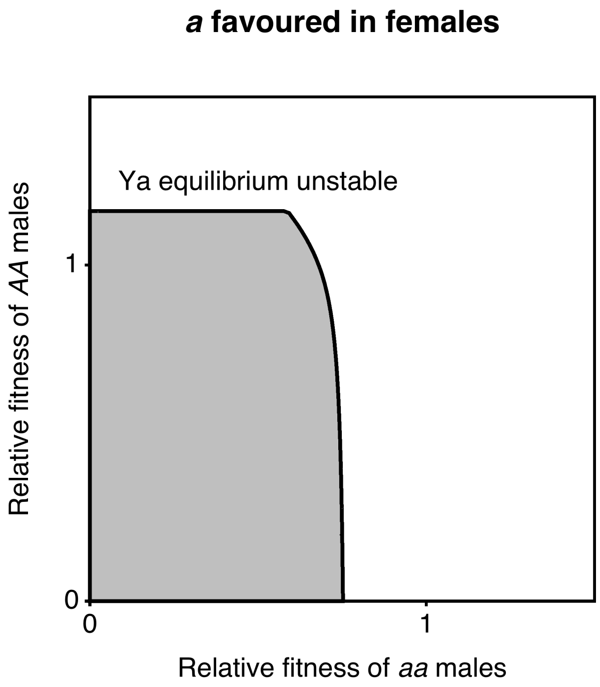

Panel C - overdominance in females, a drives in males

Parameters

```
params = {
    wAm → 1, wam → 1, wAf → 1, waf → 1,
    αm → 1 / 2 - 8 / 100,
    αf → 1 / 2,
    MAa → 1, FAa → 1,
    FAA → 1 - 4 / 10,
    Faa → 1 - 4 / 10
  };
```

No recombination eigenvalues

```
WAinvA = λmA1 /. reverse /. pAveM → (1 - q) pXm + q pYm /. equilA0 /. params // Simplify;
WainvA = λma1 /. reverse /. pAveM → (1 - q) pXm + q pYm /. equilA0 /. params // Simplify;
WAinvB = λmA1 /. reverse /. pAveM → (1 - q) pXm + q pYm /. equilB0 /. params // Simplify;
WainvB = λma1 /. reverse /. pAveM → (1 - q) pXm + q pYm /. equilB0 /. params // Simplify;
```

Maximum absolute no recombination eigenvalue from the full characteristic polynomial

```
λWsolA =
  Max[Abs[λ /. Solve[0 == charpolyk1 /. r → 0 /. R → 0 /. ρ → 0 /. equilA0 /. params, λ] //
      Simplify]];
λWsolB = Max[Abs[λ /. Solve[0 == charpolyk1 /. r → 0 /. R → 0 /. ρ → 0 /. equilB0 /.
        params, λ] // Simplify]];
```

Plot

Region plots of invasion

```
(*neo-WA invades XY from equilA*)
plotWAinvA =
  RegionPlot[{
     (validcondA /. params) && (*valid*)
      (stabcondA /. Rf → 0 /. Rm → 0 /. equilA0 /. params) && (*internally stable*)
      1 < WAinvA (*invasion*)
    },
    {Maa, 0, 3 / 2}, {MAA, 0, 3 / 2},
    PlotStyle → {Gray, Opacity[0.5]},
    BoundaryStyle → None
  ];

(*neo-Wa invades XY from equilA*)
plotWainvA =
  RegionPlot[{
     (validcondA /. params) &&
      (stabcondA /. Rf → 0 /. Rm → 0 /. equilA0 /. params) &&
      1 < WainvA
    },
    {Maa, 0, 3 / 2}, {MAA, 0, 3 / 2},
    PlotStyle → {Gray, Opacity[0.5]},
    BoundaryStyle → None
  ];
```

```
(*neo-WA invades XY from equilB*)
plotWAinvB =
  RegionPlot[{
     (stabcondB /. Rf → 0 /. Rm → 0 /. equilB0 /. params) && (*internally stable*)
      1 < WAinvB(*invasion*)
    },
    {Maa, 0, 3 / 2}, {MAA, 0, 3 / 2},
    PlotStyle → {Gray, Opacity[0.5]},
    BoundaryStyle → None
  ];

(*neo-Wa invades XY from equilB*)
plotWainvB =
  RegionPlot[{
     (stabcondB /. Rf → 0 /. Rm → 0 /. equilB0 /. params) &&
      1 < WainvB
    },
    {Maa, 0, 3 / 2}, {MAA, 0, 3 / 2},
    PlotStyle → {Gray, Opacity[0.5]},
    BoundaryStyle → None
  ];

(*Ya equilibrium internally stable*)
plotYaStable =
  RegionPlot[{
     (stabcondA /. Rf → 0 /. Rm → 0 /. equilA0 /. params) ||
      (stabcondB /. Rf → 0 /. Rm → 0 /. equilB0 /. params)
    },
    {Maa, 0, 3 / 2}, {MAA, 0, 3 / 2},
    PlotStyle → White,
    BoundaryStyle → {Black, Thick}
  ];

(*Ya equilibrium internally unstable*)
plotYaUnstable =
  RegionPlot[{
     2 < 3
    },
    {Maa, 0, 3 / 2}, {MAA, 0, 3 / 2},
    PlotStyle → None,
    BoundaryStyle → None,
    Mesh → 50,
    MeshFunctions → {#1 - #2 &},
    MeshStyle → Black
  ];
```

```
plotC =
 Show[

  plotYaUnstable,
  plotYaStable,
  plotWAinvA,
  plotWainvA,
  plotWAinvB,
  plotWainvB,

  PlotRange → {{0, 1.5}, {0, 1.5}},
  ImageSize → {xsize, xsize},
  PlotRangePadding → 0,
  FrameTicks → {Table[{x, x, ticksize}, {x, 0, 1, 1}],
    Table[{y, y, ticksize}, {y, 0, 1, 1}], None, None},
  FrameTicksStyle → {{Directive[Black, Thickness[lwd]],
     Directive[Black, Thickness[lwd]]}, {Directive[Black, Thickness[lwd]],
     Directive[Black, Thickness[lwd], FontColor → White]}},
  FrameStyle → {{Black, Thickness[lwd]}, {Black, Thickness[lwd]},
    {Black, Thickness[lwd]}, {Black, Thickness[lwd]}},
  (*FrameLabel→{"Relative fitness of aa males",""},*)
  BaseStyle → {FontFamily → "Helvetica", FontSize → 14},
  ImagePadding → Pad,
  Epilog → {
    Text[Style["C", 14, Bold], Scaled@{0.05, 0.95}],
    Text[Style["Λ_{W'A}^{(XY)} >1
&
Λ_{W'a}^{(XY)} >1", 14], {0.45, 0.5}, {1, 0}],
    Text[Style["Λ_{W'A}^{(XY)} >1 & Λ_{W'a}^{(XY)} <1", 14], {1.1, 0.5}](*,
    Rotate[
     Text[Style["Relative fitness of AA males",14],Scaled@ylabpos],90 Degree]*)
   },
  PlotLabel → Style["overdominance in females", 16, Black, Bold],
  PlotRangeClipping → False
 ]
```

overdominance in females

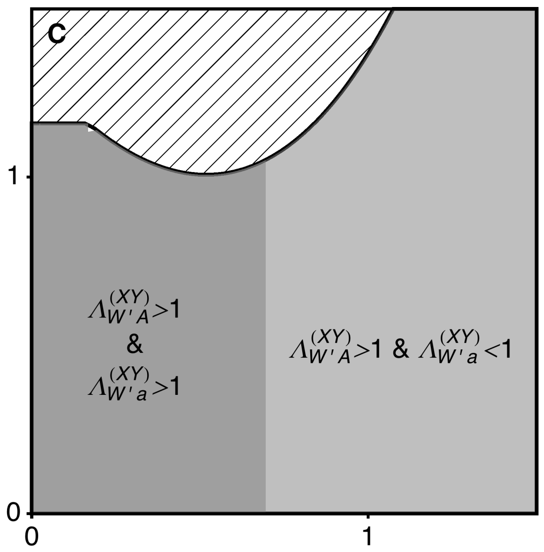

Notice that this is consistent with the solution from the full characteristic polynomial (but here we don’t know which eigenvalue belongs to which haplotype)

```
(*neo-W invades XY from equilA*)
plotWinvA =
  RegionPlot[{
    (validcondA /. params) && (*valid*)
     (stabcondA /. Rf → 0 /. Rm → 0 /. equilA0 /. params) && (*internally stable*)
     1 < λWsolA (*invasion*)
   },
   {Maa, 0, 3 / 2}, {MAA, 0, 3 / 2},
   PlotStyle → {Gray, Opacity[0.5]},
   BoundaryStyle → None
  ];

(*neo-W invades XY from equilB*)
plotWinvB =
  RegionPlot[{
    (stabcondB /. Rf → 0 /. Rm → 0 /. equilB0 /. params) && (*internally stable*)
     1 < λWsolB(*invasion*)
   },
   {Maa, 0, 3 / 2}, {MAA, 0, 3 / 2},
   PlotStyle → {Gray, Opacity[0.5]},
   BoundaryStyle → None
  ];
```

```
Show[

 plotWinvA,
 plotWinvB,
 plotYaStable,

 PlotRange → {{0, 1.5}, {0, 1.5}},
 ImageSize → {xsize, xsize},
 PlotRangePadding → 0,
 FrameTicks → {Table[{x, x, ticksize}, {x, 0, 1, 1}],
   Table[{y, y, ticksize}, {y, 0, 1, 1}], None, None},
 FrameTicksStyle → {{Directive[Black, Thickness[lwd]],
    Directive[Black, Thickness[lwd]]}, {Directive[Black, Thickness[lwd]],
    Directive[Black, Thickness[lwd], FontColor → White]}},
 FrameStyle → {{Black, Thickness[lwd]}, {Black, Thickness[lwd]},
   {Black, Thickness[lwd]}, {Black, Thickness[lwd]}},
 FrameLabel → {"Relative fitness of aa males", ""},
 BaseStyle → {FontFamily → "Helvetica", FontSize → 14},
 ImagePadding → Pad,
 Epilog → {
   Text[Style["Ya equilibrium unstable", 14], {0.5, 1.25}],
   Rotate[
    Text[Style["Relative fitness of AA males", 14], Scaled@ylabpos], 90 Degree]
  },
 PlotLabel → Style["A favoured in females", 16, Black, Bold],
 PlotRangeClipping → False
]
```

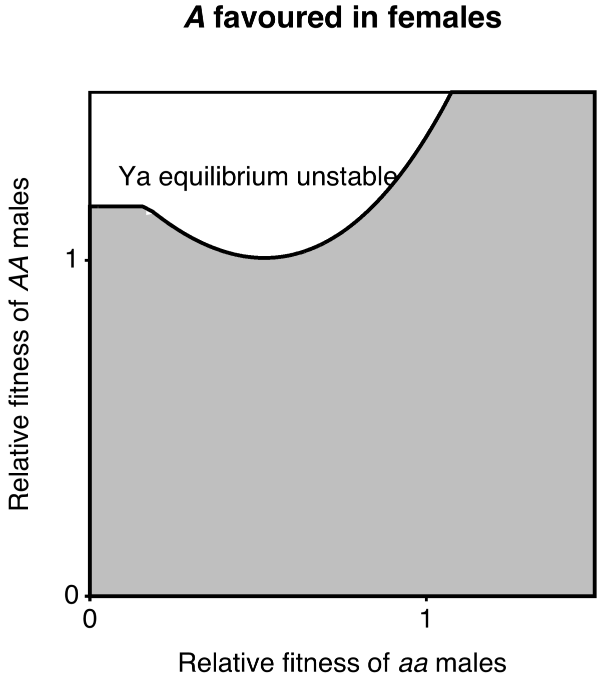

Panel D - A favoured in females, A drives in males

Parameters

```
params = {
   wAm → 1, wam → 1, wAf → 1, waf → 1,
   αm → 1 / 2 + 8 / 100,
   αf → 1 / 2,
   MAa → 1, FAa → 1,
   Faa → 1 - 3 / 20,
   FAA → 1 + 1 / 20
  };
```

No recombination eigenvalues

```
WAinvA = λmA1 /. reverse /. pAveM → (1 - q) pXm + q pYm /. equilA0 /. params // Simplify;
WainvA = λma1 /. reverse /. pAveM → (1 - q) pXm + q pYm /. equilA0 /. params // Simplify;
WAinvB = λmA1 /. reverse /. pAveM → (1 - q) pXm + q pYm /. equilB0 /. params // Simplify;
WainvB = λma1 /. reverse /. pAveM → (1 - q) pXm + q pYm /. equilB0 /. params // Simplify;
```

Maximum absolute no recombination eigenvalue from the full characteristic polynomial

```
λWsolA =
  Max[Abs[λ /. Solve[0 == charpolyk1 /. r → 0 /. R → 0 /. ρ → 0 /. equilA0 /. params, λ] //
      Simplify]];
λWsolB = Max[Abs[λ /. Solve[0 == charpolyk1 /. r → 0 /. R → 0 /. ρ → 0 /. equilB0 /.
         params, λ] // Simplify]];
```

Plot

Region plots of invasion

```
(*neo-WA invades XY from equilA*)
plotWAinvA =
  RegionPlot[{
     (validcondA /. params) && (*valid*)
      (stabcondA /. Rf → 0 /. Rm → 0 /. equilA0 /. params) && (*internally stable*)
      1 < WAinvA (*invasion*)
    },
    {Maa, 0, 3 / 2}, {MAA, 0, 3 / 2},
    PlotStyle → {Gray, Opacity[0.5]},
    BoundaryStyle → None
   ];

(*neo-Wa invades XY from equilA*)
plotWainvA =
  RegionPlot[{
     (validcondA /. params) &&
      (stabcondA /. Rf → 0 /. Rm → 0 /. equilA0 /. params) &&
      1 < WainvA
    },
    {Maa, 0, 3 / 2}, {MAA, 0, 3 / 2},
    PlotStyle → {Gray, Opacity[0.5]},
    BoundaryStyle → None
   ];
```

```
(*neo-WA invades XY from equilB*)
plotWAinvB =
  RegionPlot[{
     (stabcondB /. Rf → 0 /. Rm → 0 /. equilB0 /. params) && (*internally stable*)
      1 < WAinvB(*invasion*)
    },
    {Maa, 0, 3 / 2}, {MAA, 0, 3 / 2},
    PlotStyle → {Gray, Opacity[0.5]},
    BoundaryStyle → None
  ];

(*neo-Wa invades XY from equilB*)
plotWainvB =
  RegionPlot[{
     (stabcondB /. Rf → 0 /. Rm → 0 /. equilB0 /. params) &&
      1 < WainvB
    },
    {Maa, 0, 3 / 2}, {MAA, 0, 3 / 2},
    PlotStyle → {Gray, Opacity[0.5]},
    BoundaryStyle → None
  ];

(*Ya equilibrium internally stable*)
plotYaStable =
  RegionPlot[{
     (stabcondA /. Rf → 0 /. Rm → 0 /. equilA0 /. params) ||
      (stabcondB /. Rf → 0 /. Rm → 0 /. equilB0 /. params)
    },
    {Maa, 0, 3 / 2}, {MAA, 0, 3 / 2},
    PlotStyle → White,
    BoundaryStyle → {Black, Thick}
  ];

(*Ya equilibrium internally unstable*)
plotYaUnstable =
  RegionPlot[{
     1 < 2
    },
    {Maa, 0, 3 / 2}, {MAA, 0, 3 / 2},
    PlotStyle → None,
    BoundaryStyle → None,
    Mesh → 50,
    MeshFunctions → {#1 - #2 &},
    MeshStyle → Black
  ];
```

```
plotD =
 Show[

  plotYaUnstable,
  plotYaStable,
  plotWAinvA,
  plotWainvA,
  plotWAinvB,
  plotWainvB,

  Graphics[{Thick, Black, Line[{{1.35, 0.5}, {1.475, 0.75}}]}],
  PlotRange → {{0, 1.5}, {0, 1.5}},
  ImageSize → {xsize, xsize},
  PlotRangePadding → 0,
  FrameTicks → {Table[{x, x, ticksize}, {x, 0, 1, 1}],
    Table[{y, y, ticksize}, {y, 0, 1, 1}], None, None},
  FrameTicksStyle → {{Directive[Black, Thickness[lwd]],
     Directive[Black, Thickness[lwd]]}, {Directive[Black, Thickness[lwd]],
     Directive[Black, Thickness[lwd], FontColor → White]}},
  FrameStyle → {{Black, Thickness[lwd]}, {Black, Thickness[lwd]},
    {Black, Thickness[lwd]}, {Black, Thickness[lwd]}},
  FrameLabel → {"Relative fitness of aa males", ""},
  BaseStyle → {FontFamily → "Helvetica", FontSize → 14},
  ImagePadding → Pad,
  Epilog → {
    Text[Style["D", 14, Bold], Scaled@{0.05, 0.95}],
    Text[Style["Λ_{W'A}^{(XY)} <1 & Λ_{W'a}^{(XY)} <1", 14], {0.5, 0.5}],
    Text[Style["Λ_{W'A}^{(XY)} >1
&
Λ_{W'a}^{(XY)} <1", 14], {1.35, 0.5}, {1, 0}],
    Rotate[
     Text[Style["Relative fitness of AA males", 14], Scaled@ylabpos], 90 Degree]
   },
  PlotLabel → Style[(*"A favoured in females"*)"", 16, Black, Bold],
  PlotRangeClipping → False
 ]
```

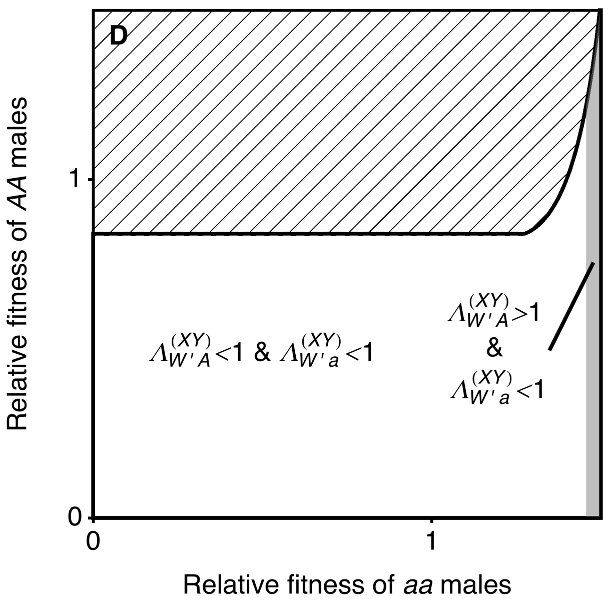

Notice that this is consistent with the solution from the full characteristic polynomial (but here we don't know which eigenvalue belongs to which haplotype)

```
(*neo-W invades XY from equilA*)
plotWinvA =
  RegionPlot[{
    (validcondA /. params) && (*valid*)
     (stabcondA /. Rf → 0 /. Rm → 0 /. equilA0 /. params) && (*internally stable*)
     1 < λWsolA (*invasion*)
   },
   {Maa, 0, 3 / 2}, {MAA, 0, 3 / 2},
   PlotStyle → {Gray, Opacity[0.5]},
   BoundaryStyle → None
  ];

(*neo-W invades XY from equilB*)
plotWinvB =
  RegionPlot[{
    (stabcondB /. Rf → 0 /. Rm → 0 /. equilB0 /. params) && (*internally stable*)
     1 < λWsolB(*invasion*)
   },
   {Maa, 0, 3 / 2}, {MAA, 0, 3 / 2},
   PlotStyle → {Gray, Opacity[0.5]},
   BoundaryStyle → None
  ];
```

```
Show[

 plotWinvA,
 plotWinvB,
 plotYaStable,

 PlotRange → {{0, 1.5}, {0, 1.5}},
 ImageSize → {xsize, xsize},
 PlotRangePadding → 0,
 FrameTicks → {Table[{x, x, ticksize}, {x, 0, 1, 1}],
   Table[{y, y, ticksize}, {y, 0, 1, 1}], None, None},
 FrameTicksStyle → {{Directive[Black, Thickness[lwd]],
    Directive[Black, Thickness[lwd]]}, {Directive[Black, Thickness[lwd]],
    Directive[Black, Thickness[lwd], FontColor → White]}},
 FrameStyle → {{Black, Thickness[lwd]}, {Black, Thickness[lwd]},
   {Black, Thickness[lwd]}, {Black, Thickness[lwd]}},
 FrameLabel → {"Relative fitness of aa males", ""},
 BaseStyle → {FontFamily → "Helvetica", FontSize → 14},
 ImagePadding → Pad,
 Epilog → {
   Text[Style["Ya equilibrium unstable", 14], {0.5, 1.25}],
   Rotate[
    Text[Style["Relative fitness of AA males", 14], Scaled@ylabpos], 90 Degree]
  },
 PlotLabel → Style["A favoured in females", 16, Black, Bold],
 PlotRangeClipping → False
]
```

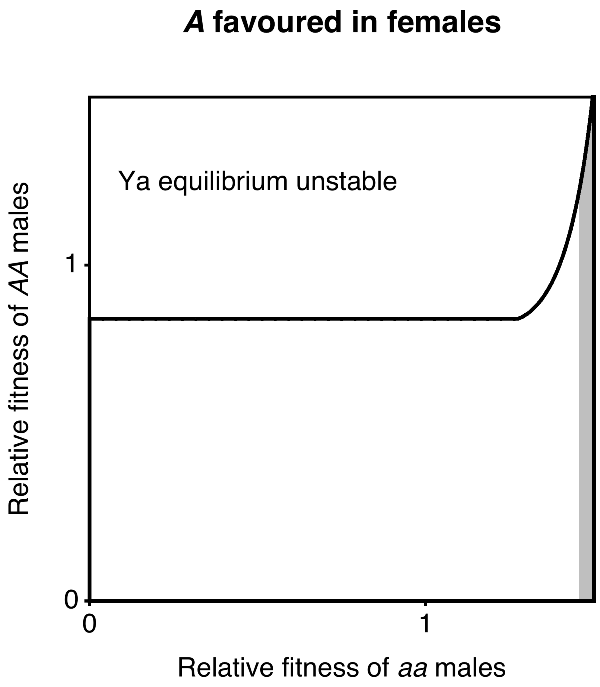

Panel E - a favoured in females, A drives in males

Parameters

```
params = {
    wAm → 1, wam → 1, wAf → 1, waf → 1,
    αm → 1 / 2 + 8 / 100,
    αf → 1 / 2,
    MAa → 1, FAa → 1,
    FAA → 1 - 3 / 20,
    Faa → 1 + 1 / 20
  };
```

No recombination eigenvalues

```
WAinvA = λmA1 /. reverse /. pAveM → (1 - q) pXm + q pYm /. equilA0 /. params // Simplify;
WainvA = λma1 /. reverse /. pAveM → (1 - q) pXm + q pYm /. equilA0 /. params // Simplify;
WAinvB = λmA1 /. reverse /. pAveM → (1 - q) pXm + q pYm /. equilB0 /. params // Simplify;
WainvB = λma1 /. reverse /. pAveM → (1 - q) pXm + q pYm /. equilB0 /. params // Simplify;
```

Maximum absolute no recombination eigenvalue from the full characteristic polynomial

```
λWsolA =
  Max[Abs[λ /. Solve[0 == charpolyk1 /. r → 0 /. R → 0 /. ρ → 0 /. equilA0 /. params, λ] //
      Simplify]];
λWsolB = Max[Abs[λ /. Solve[0 == charpolyk1 /. r → 0 /. R → 0 /. ρ → 0 /. equilB0 /.
         params, λ] // Simplify]];
```

Plot

Region plots of invasion

```
(*neo-WA invades XY from equilA*)
plotWAinvA =
  RegionPlot[{
     (validcondA /. params) && (*valid*)
      (stabcondA /. Rf → 0 /. Rm → 0 /. equilA0 /. params) && (*internally stable*)
      1 < WAinvA (*invasion*)
    },
    {Maa, 0, 3 / 2}, {MAA, 0, 3 / 2},
    PlotStyle → {Gray, Opacity[0.5]},
    BoundaryStyle → None
  ];

(*neo-Wa invades XY from equilA*)
plotWainvA =
  RegionPlot[{
     (validcondA /. params) &&
      (stabcondA /. Rf → 0 /. Rm → 0 /. equilA0 /. params) &&
      1 < WainvA
    },
    {Maa, 0, 3 / 2}, {MAA, 0, 3 / 2},
    PlotStyle → {Gray, Opacity[0.5]},
    BoundaryStyle → None
  ];
```

```
(*neo-WA invades XY from equilB*)
plotWAinvB =
  RegionPlot[{
    (stabcondB /. Rf → 0 /. Rm → 0 /. equilB0 /. params) && (*internally stable*)
     1 < WAinvB(*invasion*)
   },
   {Maa, 0, 3 / 2}, {MAA, 0, 3 / 2},
   PlotStyle → {Gray, Opacity[0.5]},
   BoundaryStyle → None
  ];

(*neo-Wa invades XY from equilB*)
plotWainvB =
  RegionPlot[{
    (stabcondB /. Rf → 0 /. Rm → 0 /. equilB0 /. params) &&
     1 < WainvB
   },
   {Maa, 0, 3 / 2}, {MAA, 0, 3 / 2},
   PlotStyle → {Gray, Opacity[0.5]},
   BoundaryStyle → None
  ];

(*Ya equilibrium internally stable*)
plotYaStable =
  RegionPlot[{
    (stabcondA /. Rf → 0 /. Rm → 0 /. equilA0 /. params) ||
     (stabcondB /. Rf → 0 /. Rm → 0 /. equilB0 /. params)
   },
   {Maa, 0, 3 / 2}, {MAA, 0, 3 / 2},
   PlotStyle → White,
   BoundaryStyle → {Black, Thick}
  ];

(*Ya equilibrium internally unstable*)
plotYaUnstable =
  RegionPlot[{
    1 > 0
   },
   {Maa, 0, 3 / 2}, {MAA, 0, 3 / 2},
   PlotStyle → None,
   BoundaryStyle → None,
   Mesh → 50,
   MeshFunctions → {#1 - #2 &},
   MeshStyle → Black
  ];
```

```
plotE =
 Show[

  plotYaUnstable,
  plotYaStable,
  plotWAinvA,
  plotWainvA,
  plotWAinvB,
  plotWainvB,

  Graphics[{Thick, Black, Line[{{1.1, 0.5}, {0.95, 0.25}}]}],
  PlotRange → {{0, 1.5}, {0, 1.5}},
  ImageSize → {xsize, xsize},
  PlotRangePadding → 0,
  FrameTicks → {Table[{x, x, ticksize}, {x, 0, 1, 1}],
    Table[{y, y, ticksize}, {y, 0, 1, 1}], None, None},
  FrameTicksStyle → {{Directive[Black, Thickness[lwd]],
     Directive[Black, Thickness[lwd]]}, {Directive[Black, Thickness[lwd]],
     Directive[Black, Thickness[lwd], FontColor → White]}},
  FrameStyle → {{Black, Thickness[lwd]}, {Black, Thickness[lwd]},
    {Black, Thickness[lwd]}, {Black, Thickness[lwd]}},
  FrameLabel → {"Relative fitness of aa males", ""},
  BaseStyle → {FontFamily → "Helvetica", FontSize → 14},
  ImagePadding → Pad,
  Epilog → {
    Text[Style["E", 14, Bold], Scaled@{0.05, 0.95}],
    Text[Style["Λ_{W'A}^{(XY)}<1 & Λ_{W'a}^{(XY)}>1", 14], {0.4, 0.5}],
    Text[Style["Λ_{W'A}^{(XY)}<1
&
Λ_{W'a}^{(XY)}<1", 14], {1.1, 0.5}, {-1, 0}, Background → White](*,
    Rotate[
     Text[Style["Relative fitness of AA males",14],Scaled@ylabpos],90 Degree]*)
   },
  PlotLabel → Style[(*"a favoured in females"*)"", 16, Black, Bold],
  PlotRangeClipping → False
 ]
```

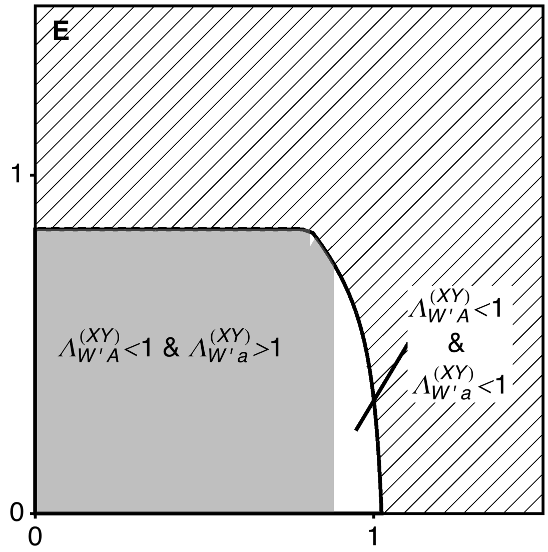

Notice that this is consistent with the solution from the full characteristic polynomial (but here we don't know which eigenvalue belongs to which haplotype)

```
(*neo-W invades XY from equilA*)
plotWinvA =
  RegionPlot[{
     (validcondA /. params) && (*valid*)
      (stabcondA /. Rf → 0 /. Rm → 0 /. equilA0 /. params) && (*internally stable*)
      1 < λWsolA (*invasion*)
    },
    {Maa, 0, 3 / 2}, {MAA, 0, 3 / 2},
    PlotStyle → {Gray, Opacity[0.5]},
    BoundaryStyle → None
  ];

(*neo-W invades XY from equilB*)
plotWinvB =
  RegionPlot[{
     (stabcondB /. Rf → 0 /. Rm → 0 /. equilB0 /. params) && (*internally stable*)
      1 < λWsolB(*invasion*)
    },
    {Maa, 0, 3 / 2}, {MAA, 0, 3 / 2},
    PlotStyle → {Gray, Opacity[0.5]},
    BoundaryStyle → None
  ];
```

```
Show[

 plotWinvA,
 plotWinvB,
 plotYaStable,

 PlotRange → {{0, 1.5}, {0, 1.5}},
 ImageSize → {xsize, xsize},
 PlotRangePadding → 0,
 FrameTicks → {Table[{x, x, ticksize}, {x, 0, 1, 1}],
   Table[{y, y, ticksize}, {y, 0, 1, 1}], None, None},
 FrameTicksStyle → {{Directive[Black, Thickness[lwd]],
    Directive[Black, Thickness[lwd]]}, {Directive[Black, Thickness[lwd]],
    Directive[Black, Thickness[lwd], FontColor → White]}},
 FrameStyle → {{Black, Thickness[lwd]}, {Black, Thickness[lwd]},
   {Black, Thickness[lwd]}, {Black, Thickness[lwd]}},
 FrameLabel → {"Relative fitness of aa males", ""},
 BaseStyle → {FontFamily → "Helvetica", FontSize → 14},
 ImagePadding → Pad,
 Epilog → {
   Text[Style["Ya equilibrium unstable", 14], {0.5, 1.25}],
   Rotate[
    Text[Style["Relative fitness of AA males", 14], Scaled@ylabpos], 90 Degree]
  },
 PlotLabel → Style["a favoured in females", 16, Black, Bold],
 PlotRangeClipping → False
]
```

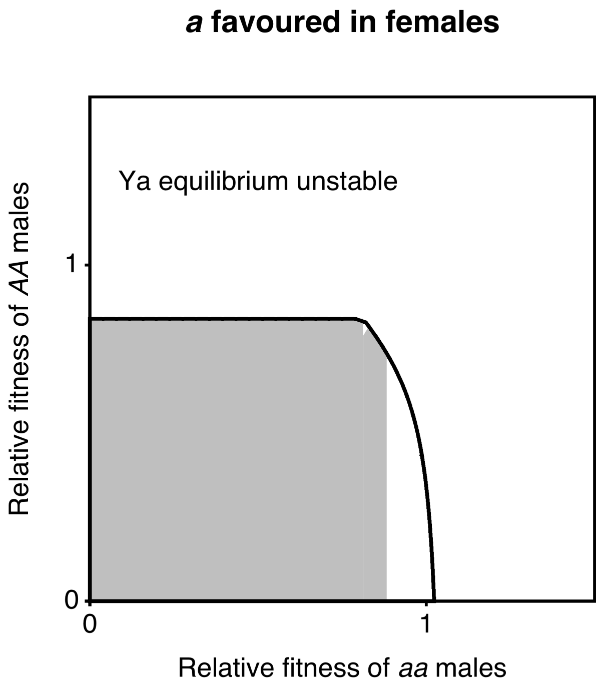

Panel F - overdominance in females, A drives in males

Parameters

```
params = {
    wAm → 1, wam → 1, wAf → 1, waf → 1,
    αm → 1 / 2 + 8 / 100,
    αf → 1 / 2,
    MAa → 1, FAa → 1,
    FAA → 1 - 4 / 10,
    Faa → 1 - 4 / 10
  };
```

No recombination eigenvalues

```
WAinvA = λmA1 /. reverse /. pAveM → (1 - q) pXm + q pYm /. equilA0 /. params // Simplify;
WainvA = λma1 /. reverse /. pAveM → (1 - q) pXm + q pYm /. equilA0 /. params // Simplify;
WAinvB = λmA1 /. reverse /. pAveM → (1 - q) pXm + q pYm /. equilB0 /. params // Simplify;
WainvB = λma1 /. reverse /. pAveM → (1 - q) pXm + q pYm /. equilB0 /. params // Simplify;
```

Maximum absolute no recombination eigenvalue from the full characteristic polynomial

```
λWsolA =
  Max[Abs[λ /. Solve[0 == charpolyk1 /. r → 0 /. R → 0 /. ρ → 0 /. equilA0 /. params, λ] //
      Simplify]];
λWsolB = Max[Abs[λ /. Solve[0 == charpolyk1 /. r → 0 /. R → 0 /. ρ → 0 /. equilB0 /.
         params, λ] // Simplify]];
```

Plot

Region plots of invasion

```
(*neo-WA invades XY from equilA*)
plotWAinvA =
  RegionPlot[{
     (validcondA /. params) && (*valid*)
      (stabcondA /. Rf → 0 /. Rm → 0 /. equilA0 /. params) && (*internally stable*)
      1 < WAinvA (*invasion*)
    },
    {Maa, 0, 3 / 2}, {MAA, 0, 3 / 2},
    PlotStyle → {Gray, Opacity[0.5]},
    BoundaryStyle → None
  ];

(*neo-Wa invades XY from equilA*)
plotWainvA =
  RegionPlot[{
     (validcondA /. params) &&
      (stabcondA /. Rf → 0 /. Rm → 0 /. equilA0 /. params) &&
      1 < WainvA
    },
    {Maa, 0, 3 / 2}, {MAA, 0, 3 / 2},
    PlotStyle → {Gray, Opacity[0.5]},
    BoundaryStyle → None
  ];
```

```
(*neo-WA invades XY from equilB*)
plotWAinvB =
  RegionPlot[{
     (stabcondB /. Rf → 0 /. Rm → 0 /. equilB0 /. params) && (*internally stable*)
      1 < WAinvB(*invasion*)
    },
    {Maa, 0, 3 / 2}, {MAA, 0, 3 / 2},
    PlotStyle → {Gray, Opacity[0.5]},
    BoundaryStyle → None
  ];

(*neo-Wa invades XY from equilB*)
plotWainvB =
  RegionPlot[{
     (stabcondB /. Rf → 0 /. Rm → 0 /. equilB0 /. params) &&
      1 < WainvB
    },
    {Maa, 0, 3 / 2}, {MAA, 0, 3 / 2},
    PlotStyle → {Gray, Opacity[0.5]},
    BoundaryStyle → None
  ];

(*Ya equilibrium internally stable*)
plotYaStable =
  RegionPlot[{
     (stabcondA /. Rf → 0 /. Rm → 0 /. equilA0 /. params) ||
      (stabcondB /. Rf → 0 /. Rm → 0 /. equilB0 /. params)
    },
    {Maa, 0, 3 / 2}, {MAA, 0, 3 / 2},
    PlotStyle → White,
    BoundaryStyle → {Black, Thick}
  ];

(*Ya equilibrium internally unstable*)
plotYaUnstable =
  RegionPlot[{
     1 + 1 < 3
    },
    {Maa, 0, 3 / 2}, {MAA, 0, 3 / 2},
    PlotStyle → None,
    BoundaryStyle → None,
    Mesh → 50,
    MeshFunctions → {#1 - #2 &},
    MeshStyle → Black
  ];
```

```
plotF =
 Show[

  plotYaUnstable,
  plotYaStable,
  plotWAinvA,
  plotWainvA,
  plotWAinvB,
  plotWainvB,

  Graphics[{Thick, Black, Line[{{0.65, 0.9}, {0.475, 0.5}}]}],
  PlotRange → {{0, 1.5}, {0, 1.5}},
  ImageSize → {xsize, xsize},
  PlotRangePadding → 0,
  FrameTicks → {Table[{x, x, ticksize}, {x, 0, 1, 1}],
    Table[{y, y, ticksize}, {y, 0, 1, 1}], None, None},
  FrameTicksStyle → {{Directive[Black, Thickness[lwd]],
     Directive[Black, Thickness[lwd]]}, {Directive[Black, Thickness[lwd]],
     Directive[Black, Thickness[lwd], FontColor → White]}},
  FrameStyle → {{Black, Thickness[lwd]}, {Black, Thickness[lwd]},
    {Black, Thickness[lwd]}, {Black, Thickness[lwd]}},
  FrameLabel → {"Relative fitness of aa males", ""},
  BaseStyle → {FontFamily → "Helvetica", FontSize → 14},
  ImagePadding → Pad,
  Epilog → {
    Text[Style["F", 14, Bold], Scaled@{0.05, 0.95}],
    Text[Style["Λ_{W'A}^{(XY)} >1
&
Λ_{W'a}^{(XY)} >1", 14], {0.4, 0.4}, {1, 0}],
    Text[Style["Λ_{W'A}^{(XY)} >1
&
Λ_{W'a}^{(XY)} <1", 14], {1.4, 0.4}, {1, 0}],
    Text[Style["Λ_{W'A}^{(XY)} <1
&
Λ_{W'a}^{(XY)} <1", 14], {0.6, 0.4}, {-1, 0}],
    Text[Style["Λ_{W'A}^{(XY)} <1
&
Λ_{W'a}^{(XY)} >1", 14], {0.65, 0.9}, {-1, 0}, Background → White](*,
    Rotate[
     Text[Style["Relative fitness of AA males",14],Scaled@ylabpos],90 Degree]*)
   },
  PlotLabel → Style[(*"overdominance in females"*)"", 16, Black, Bold],
  PlotRangeClipping → False
 ]
```

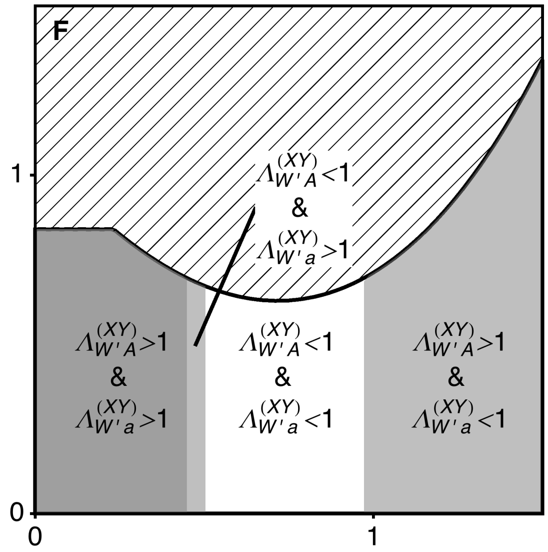

Notice that this is consistent with the solution from the full characteristic polynomial (but here we don't know which eigenvalue belongs to which haplotype)

```
(*neo-W invades XY from equilA*)
plotWinvA =
  RegionPlot[{
    (validcondA /. params) && (*valid*)
     (stabcondA /. Rf → 0 /. Rm → 0 /. equilA0 /. params) && (*internally stable*)
     1 < λWsolA (*invasion*)
   },
   {Maa, 0, 3 / 2}, {MAA, 0, 3 / 2},
   PlotStyle → {Gray, Opacity[0.5]},
   BoundaryStyle → None
  ];

(*neo-W invades XY from equilB*)
plotWinvB =
  RegionPlot[{
    (stabcondB /. Rf → 0 /. Rm → 0 /. equilB0 /. params) && (*internally stable*)
     1 < λWsolB(*invasion*)
   },
   {Maa, 0, 3 / 2}, {MAA, 0, 3 / 2},
   PlotStyle → {Gray, Opacity[0.5]},
   BoundaryStyle → None
  ];
```

```
Show[

 plotWinvA,
 plotWinvB,
 plotYaStable,

 PlotRange → {{0, 1.5}, {0, 1.5}},
 ImageSize → {xsize, xsize},
 PlotRangePadding → 0,
 FrameTicks → {Table[{x, x, ticksize}, {x, 0, 1, 1}],
   Table[{y, y, ticksize}, {y, 0, 1, 1}], None, None},
 FrameTicksStyle → {{Directive[Black, Thickness[lwd]],
    Directive[Black, Thickness[lwd]]}, {Directive[Black, Thickness[lwd]],
    Directive[Black, Thickness[lwd], FontColor → White]}},
 FrameStyle → {{Black, Thickness[lwd]}, {Black, Thickness[lwd]},
   {Black, Thickness[lwd]}, {Black, Thickness[lwd]}},
 FrameLabel → {"Relative fitness of aa males", ""},
 BaseStyle → {FontFamily → "Helvetica", FontSize → 14},
 ImagePadding → Pad,
 Epilog → {
   Text[Style["Ya equilibrium unstable", 14], {0.5, 1.25}],
   Rotate[
    Text[Style["Relative fitness of AA males", 14], Scaled@ylabpos], 90 Degree]
  },
 PlotLabel → Style["overdominance in females", 16, Black, Bold],
 PlotRangeClipping → False
]
```

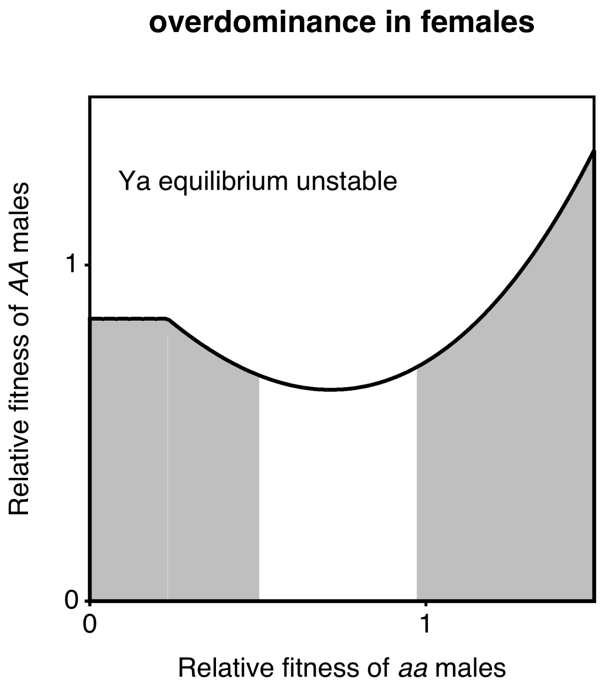

All Panels

```
GraphicsGrid[{
   {plotA, plotB, plotC},
   {plotD, plotE, plotF}
  },
  Spacings → -50
]

Export[plotdir <> "Region_plot_combined_MaleDrive.eps", % // rasterTrick];
```

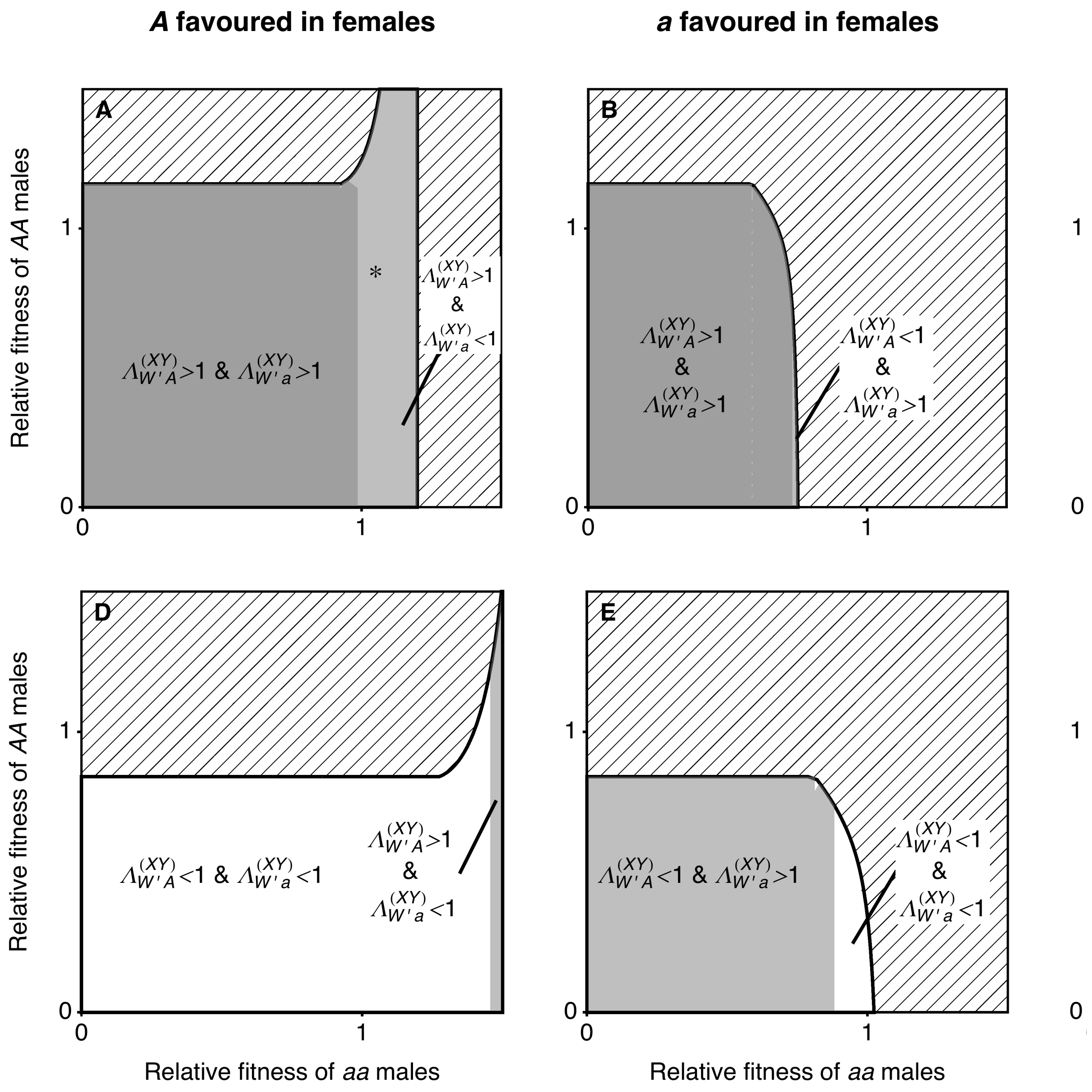

Figure S.5 - neo-W haplotype invasion with haploid competition in males

Panel A - A favoured in females, haploid competition favours a in males

Parameters

```
params = {
    wAm → 1, wam → 1 + 8 / 50, wAf → 1, waf → 1,
    αm → 1 / 2, αf → 1 / 2,
    MAa → 1, FAa → 1,
    Faa → 1 - 3 / 20,
    FAA → 1 + 1 / 20
  };
```

Making the sex-ratio

```
freqMale /. equilB0 /. params // N
```

```
0.537037
```

No recombination eigenvalues

```
WAinvA = λmA1 /. reverse /. pAveM → (1 - q) pXm + q pYm /. equilA0 /. params // Simplify;
WainvA = λma1 /. reverse /. pAveM → (1 - q) pXm + q pYm /. equilA0 /. params // Simplify;
WAinvB = λmA1 /. reverse /. pAveM → (1 - q) pXm + q pYm /. equilB0 /. params // Simplify;
WainvB = λma1 /. reverse /. pAveM → (1 - q) pXm + q pYm /. equilB0 /. params // Simplify;
```

Maximum absolute no recombination eigenvalue from the full characteristic polynomial

```
λWsolA =
  Max[Abs[λ /. Solve[0 == charpolyk1 /. r → 0 /. R → 0 /. ρ → 0 /. equilA0 /. params, λ] //
      Simplify]];
λWsolB = Max[Abs[λ /. Solve[0 == charpolyk1 /. r → 0 /. R → 0 /. ρ → 0 /. equilB0 /.
          params, λ] // Simplify]];
```

Plot

Region plots of invasion

```
(*neo-WA invades XY from equilA*)
plotWAinvA =
  RegionPlot[{
     (validcondA /. params) && (*valid*)
      (stabcondA /. Rf → 0 /. Rm → 0 /. equilA0 /. params) && (*internally stable*)
      1 < WAinvA (*invasion*)
    },
    {Maa, 0, 3 / 2}, {MAA, 0, 3 / 2},
    PlotStyle → {Gray, Opacity[0.5]},
    BoundaryStyle → None
  ];
```

```
(*neo-Wa invades XY from equilA*)
plotWainvA =
  RegionPlot[{
    (validcondA /. params) &&
     (stabcondA /. Rf → 0 /. Rm → 0 /. equilA0 /. params) &&
     1 < WainvA
   },
   {Maa, 0, 3 / 2}, {MAA, 0, 3 / 2},
   PlotStyle → {Gray, Opacity[0.5]},
   BoundaryStyle → None
  ];

(*neo-WA invades XY from equilB*)
plotWAinvB =
  RegionPlot[{
    (stabcondB /. Rf → 0 /. Rm → 0 /. equilB0 /. params) && (*internally stable*)
     1 < WAinvB(*invasion*)
   },
   {Maa, 0, 3 / 2}, {MAA, 0, 3 / 2},
   PlotStyle → {Gray, Opacity[0.5]},
   BoundaryStyle → None
  ];

(*neo-Wa invades XY from equilB*)
plotWainvB =
  RegionPlot[{
    (stabcondB /. Rf → 0 /. Rm → 0 /. equilB0 /. params) &&
     1 < WainvB
   },
   {Maa, 0, 3 / 2}, {MAA, 0, 3 / 2},
   PlotStyle → {Gray, Opacity[0.5]},
   BoundaryStyle → None
  ];

(*Ya equilibrium internally stable*)
plotYaStable =
  RegionPlot[{
    (stabcondA /. Rf → 0 /. Rm → 0 /. equilA0 /. params) ||
     (stabcondB /. Rf → 0 /. Rm → 0 /. equilB0 /. params)
   },
   {Maa, 0, 3 / 2}, {MAA, 0, 3 / 2},
   PlotStyle → White,
   BoundaryStyle → {Black, Thick}
  ];

(*Ya equilibrium internally unstable*)
plotYaUnstable =
  RegionPlot[{
    1 < 2
   },
   {Maa, 0, 3 / 2}, {MAA, 0, 3 / 2},
   PlotStyle → None,
   BoundaryStyle → None,
   Mesh → 50,
   MeshFunctions → {#1 - #2 &},
```

```
    MeshStyle → Black
   ];

plotA =
  Show[

   plotYaUnstable,
   plotYaStable,
   plotWAinvA,
   plotWainvA,
   plotWAinvB,
   plotWainvB,


   PlotRange → {{0, 1.5}, {0, 1.5}},
   ImageSize → {xsize, xsize},
   PlotRangePadding → 0,
   FrameTicks → {Table[{x, x, ticksize}, {x, 0, 1, 1}],
     Table[{y, y, ticksize}, {y, 0, 1, 1}], None, None},
   FrameTicksStyle → {{Directive[Black, Thickness[lwd]],
      Directive[Black, Thickness[lwd]]}, {Directive[Black, Thickness[lwd]],
      Directive[Black, Thickness[lwd], FontColor → White]}},
   FrameStyle → {{Black, Thickness[lwd]}, {Black, Thickness[lwd]},
     {Black, Thickness[lwd]}, {Black, Thickness[lwd]}},
   (*FrameLabel→{"Relative fitness of aa males",""},*)
   BaseStyle → {FontFamily → "Helvetica", FontSize → 14},
   ImagePadding → Pad,
   Epilog → {
     Text[Style["A", 14, Bold], Scaled@{0.05, 0.95}],
     Text[Style["Λ_{W'A}^{(XY)}>1 & Λ_{W'a}^{(XY)}<1", 14], {0.5, 0.5}],
     Rotate[
      Text[Style["Relative fitness of AA males", 14], Scaled@ylabpos], 90 Degree]
    },
   PlotLabel → Style["A favoured in females", 16, Black, Bold],
   PlotRangeClipping → False
  ]
```

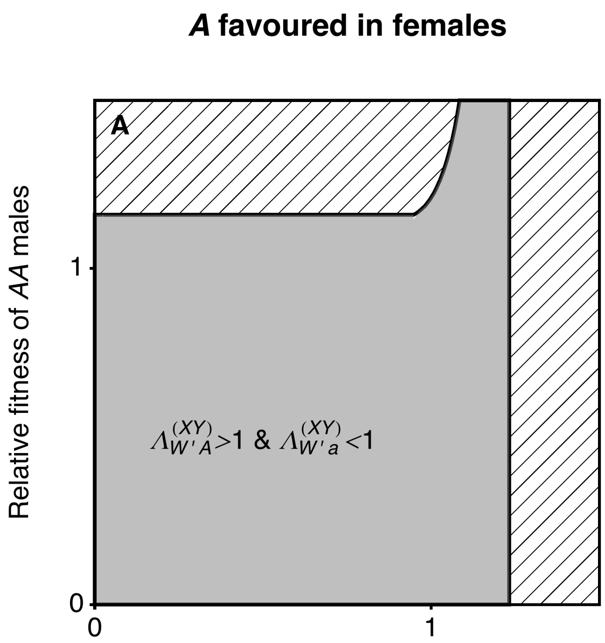

Notice that this is consistent with the solution from the full characteristic polynomial (but here we don't know which eigenvalue belongs to which haplotype)

```
(*neo-W invades XY from equilA*)
plotWinvA =
  RegionPlot[{
    (validcondA /. params) && (*valid*)
     (stabcondA /. Rf → 0 /. Rm → 0 /. equilA0 /. params) && (*internally stable*)
     1 < λWsolA (*invasion*)
   },
   {Maa, 0, 3 / 2}, {MAA, 0, 3 / 2},
   PlotStyle → {Gray, Opacity[0.5]},
   BoundaryStyle → None
  ];

(*neo-W invades XY from equilB*)
plotWinvB =
  RegionPlot[{
    (stabcondB /. Rf → 0 /. Rm → 0 /. equilB0 /. params) && (*internally stable*)
     1 < λWsolB(*invasion*)
   },
   {Maa, 0, 3 / 2}, {MAA, 0, 3 / 2},
   PlotStyle → {Gray, Opacity[0.5]},
   BoundaryStyle → None
  ];
```

```
Show[

 plotWinvA,
 plotWinvB,
 plotYaStable,

 PlotRange → {{0, 1.5}, {0, 1.5}},
 ImageSize → {xsize, xsize},
 PlotRangePadding → 0,
 FrameTicks → {Table[{x, x, ticksize}, {x, 0, 1, 1}],
   Table[{y, y, ticksize}, {y, 0, 1, 1}], None, None},
 FrameTicksStyle → {{Directive[Black, Thickness[lwd]],
    Directive[Black, Thickness[lwd]]}, {Directive[Black, Thickness[lwd]],
    Directive[Black, Thickness[lwd], FontColor → White]}},
 FrameStyle → {{Black, Thickness[lwd]}, {Black, Thickness[lwd]},
   {Black, Thickness[lwd]}, {Black, Thickness[lwd]}},
 FrameLabel → {"Relative fitness of aa males", ""},
 BaseStyle → {FontFamily → "Helvetica", FontSize → 14},
 ImagePadding → Pad,
 Epilog → {
   Text[Style["Ya equilibrium unstable", 14], {0.5, 1.25}],
   Rotate[
    Text[Style["Relative fitness of AA males", 14], Scaled@ylabpos], 90 Degree]
  },
 PlotLabel → Style["A favoured in females", 16, Black, Bold],
 PlotRangeClipping → False
]
```

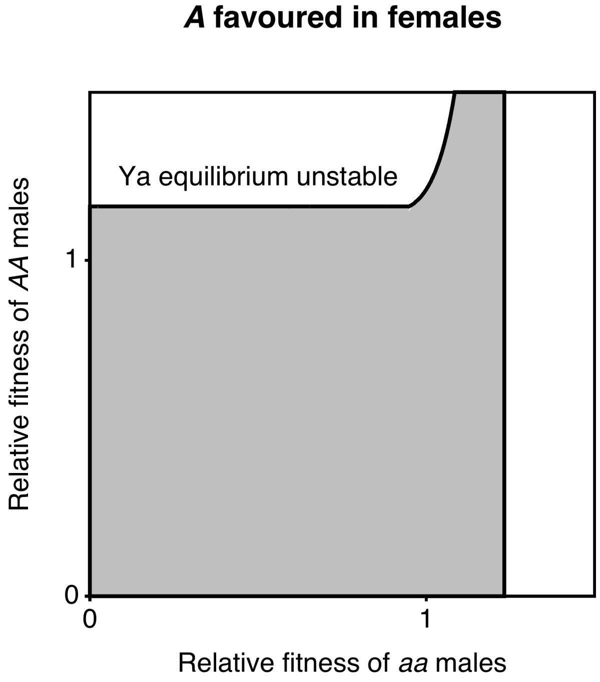

Panel B - a favoured in females, haploid competition favours a in males

Parameters

```
params = {
    wAm → 1, wam → 1 + 8 / 50, wAf → 1, waf → 1,
    αm → 1 / 2, αf → 1 / 2,
    MAa → 1, FAa → 1,
    FAA → 1 - 3 / 20,
    Faa → 1 + 1 / 20
  };
```

No recombination eigenvalues

```
WAinvA = λmA1 /. reverse /. pAveM → (1 - q) pXm + q pYm /. equilA0 /. params // Simplify;
WainvA = λma1 /. reverse /. pAveM → (1 - q) pXm + q pYm /. equilA0 /. params // Simplify;
WAinvB = λmA1 /. reverse /. pAveM → (1 - q) pXm + q pYm /. equilB0 /. params // Simplify;
WainvB = λma1 /. reverse /. pAveM → (1 - q) pXm + q pYm /. equilB0 /. params // Simplify;
```

Maximum absolute no recombination eigenvalue from the full characteristic polynomial

```
λWsolA =
  Max[Abs[λ /. Solve[0 == charpolyk1 /. r → 0 /. R → 0 /. ρ → 0 /. equilA0 /. params, λ] //
      Simplify]];
λWsolB = Max[Abs[λ /. Solve[0 == charpolyk1 /. r → 0 /. R → 0 /. ρ → 0 /. equilB0 /.
        params, λ] // Simplify]];
```

Plot

Region plots of invasion

```
(*neo-WA invades XY from equilA*)
plotWAinvA =
  RegionPlot[{
     (validcondA /. params) && (*valid*)
      (stabcondA /. Rf → 0 /. Rm → 0 /. equilA0 /. params) && (*internally stable*)
      1 < WAinvA (*invasion*)
    },
    {Maa, 0, 3 / 2}, {MAA, 0, 3 / 2},
    PlotStyle → {Gray, Opacity[0.5]},
    BoundaryStyle → None
  ];

(*neo-Wa invades XY from equilA*)
plotWainvA =
  RegionPlot[{
     (validcondA /. params) &&
      (stabcondA /. Rf → 0 /. Rm → 0 /. equilA0 /. params) &&
      1 < WainvA
    },
    {Maa, 0, 3 / 2}, {MAA, 0, 3 / 2},
    PlotStyle → {Gray, Opacity[0.5]},
    BoundaryStyle → None
  ];
```

Printed by Wolfram Mathematica Student Edition

```
(*neo-WA invades XY from equilB*)
plotWAinvB =
  RegionPlot[{
    (stabcondB /. Rf → 0 /. Rm → 0 /. equilB0 /. params) && (*internally stable*)
     1 < WAinvB(*invasion*)
   },
   {Maa, 0, 3 / 2}, {MAA, 0, 3 / 2},
   PlotStyle → {Gray, Opacity[0.5]},
   BoundaryStyle → None
  ];

(*neo-Wa invades XY from equilB*)
plotWainvB =
  RegionPlot[{
    (stabcondB /. Rf → 0 /. Rm → 0 /. equilB0 /. params) &&
     1 < WainvB
   },
   {Maa, 0, 3 / 2}, {MAA, 0, 3 / 2},
   PlotStyle → {Gray, Opacity[0.5]},
   BoundaryStyle → None
  ];

(*Ya equilibrium internally stable*)
plotYaStable =
  RegionPlot[{
    (stabcondA /. Rf → 0 /. Rm → 0 /. equilA0 /. params) ||
     (stabcondB /. Rf → 0 /. Rm → 0 /. equilB0 /. params)
   },
   {Maa, 0, 3 / 2}, {MAA, 0, 3 / 2},
   PlotStyle → None,
   BoundaryStyle → {Black, Thick}
  ];

(*Ya equilibrium internally unstable*)
plotYaUnstable =
  RegionPlot[{
    1 < 2
   },
   {Maa, 0, 3 / 2}, {MAA, 0, 3 / 2},
   PlotStyle → None,
   BoundaryStyle → None,
   Mesh → 50,
   MeshFunctions → {#1 - #2 &},
   MeshStyle → Black
  ];
```

```
plotB =
 Show[

  plotYaUnstable,
  plotYaStable,
  plotWAinvA,
  plotWainvA,
  plotWAinvB,
  plotWainvB,

  Graphics[{Thick, Black, Line[{{0.9, 0.5}, {0.75, 0.25}}]}],
  PlotRange → {{0, 1.5}, {0, 1.5}},
  ImageSize → {xsize, xsize},
  PlotRangePadding → 0,
  FrameTicks → {Table[{x, x, ticksize}, {x, 0, 1, 1}],
    Table[{y, y, ticksize}, {y, 0, 1, 1}], None, None},
  FrameTicksStyle → {{Directive[Black, Thickness[lwd]],
     Directive[Black, Thickness[lwd]]}, {Directive[Black, Thickness[lwd]],
     Directive[Black, Thickness[lwd], FontColor → White]}},
  FrameStyle → {{Black, Thickness[lwd]}, {Black, Thickness[lwd]},
    {Black, Thickness[lwd]}, {Black, Thickness[lwd]}},
  (*FrameLabel→{"Relative fitness of aa males",""},*)
  BaseStyle → {FontFamily → "Helvetica", FontSize → 14},
  ImagePadding → Pad,
  Epilog → {
    Text[Style["B", 14, Bold], Scaled@{0.05, 0.95}],
    Text[Style["Λ^(XY)_W'A >1
&
Λ^(XY)_W'a >1", 14], {0.35, 0.5}],
    Text[Style["Λ^(XY)_W'A <1
&
Λ^(XY)_W'a >1", 14], {0.9, 0.5}, {-1.2, 0}, Background → White](*,
    Rotate[
     Text[Style["Relative fitness of AA males",14],Scaled@ylabpos],90 Degree]*)
   },
  PlotLabel → Style["a favoured in females", 16, Black, Bold],
  PlotRangeClipping → False
 ]
```

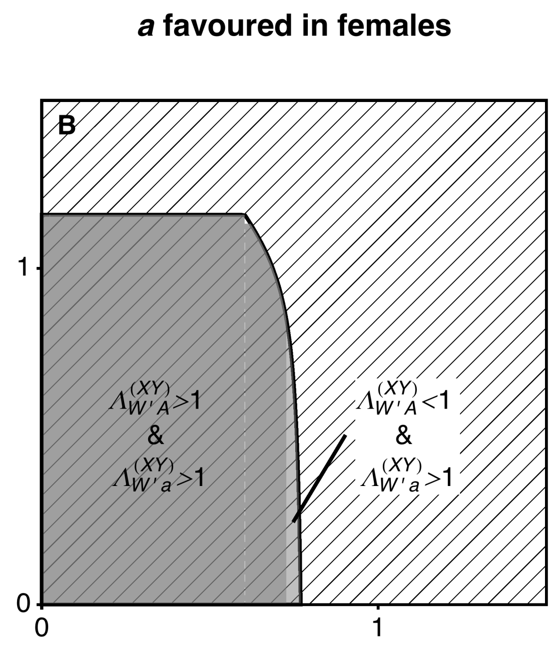

Notice that this is consistent with the solution from the full characteristic polynomial (but here we don't know which eigenvalue belongs to which haplotype)

```
(*neo-W invades XY from equilA*)
plotWinvA =
  RegionPlot[{
    (validcondA /. params) && (*valid*)
     (stabcondA /. Rf → 0 /. Rm → 0 /. equilA0 /. params) && (*internally stable*)
     1 < λWsolA (*invasion*)
   },
   {Maa, 0, 3 / 2}, {MAA, 0, 3 / 2},
   PlotStyle → {Gray, Opacity[0.5]},
   BoundaryStyle → None
  ];

(*neo-W invades XY from equilB*)
plotWinvB =
  RegionPlot[{
    (stabcondB /. Rf → 0 /. Rm → 0 /. equilB0 /. params) && (*internally stable*)
     1 < λWsolB(*invasion*)
   },
   {Maa, 0, 3 / 2}, {MAA, 0, 3 / 2},
   PlotStyle → {Gray, Opacity[0.5]},
   BoundaryStyle → None
  ];
```

```
Show[

 plotWinvA,
 plotWinvB,
 plotYaStable,

 PlotRange → {{0, 1.5}, {0, 1.5}},
 ImageSize → {xsize, xsize},
 PlotRangePadding → 0,
 FrameTicks → {Table[{x, x, ticksize}, {x, 0, 1, 1}],
   Table[{y, y, ticksize}, {y, 0, 1, 1}], None, None},
 FrameTicksStyle → {{Directive[Black, Thickness[lwd]],
    Directive[Black, Thickness[lwd]]}, {Directive[Black, Thickness[lwd]],
    Directive[Black, Thickness[lwd], FontColor → White]}},
 FrameStyle → {{Black, Thickness[lwd]}, {Black, Thickness[lwd]},
   {Black, Thickness[lwd]}, {Black, Thickness[lwd]}},
 FrameLabel → {"Relative fitness of aa males", ""},
 BaseStyle → {FontFamily → "Helvetica", FontSize → 14},
 ImagePadding → Pad,
 Epilog → {
   Text[Style["Ya equilibrium unstable", 14], {0.5, 1.25}],
   Rotate[
    Text[Style["Relative fitness of AA males", 14], Scaled@ylabpos], 90 Degree]
  },
 PlotLabel → Style["a favoured in females", 16, Black, Bold],
 PlotRangeClipping → False
]
```

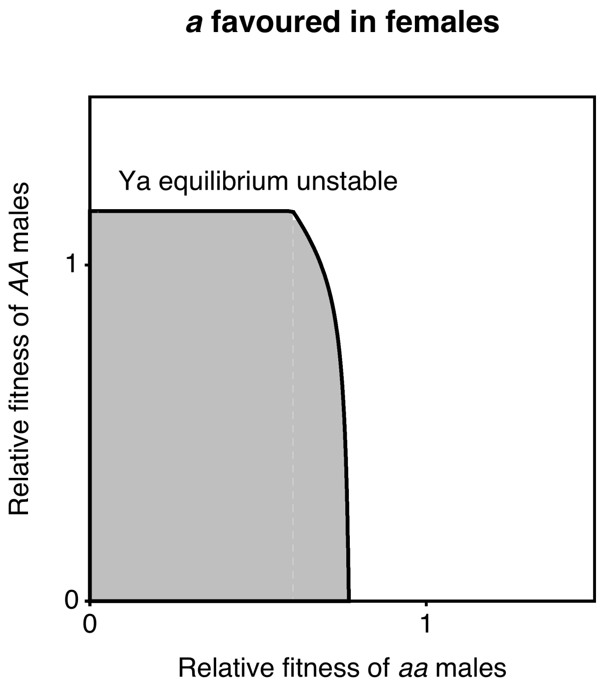

Panel C - overdominance in females, haploid competition favours a in males

Parameters

```
params = {
    wAm → 1, wam → 1 + 8 / 50, wAf → 1, waf → 1,
    αm → 1 / 2, αf → 1 / 2,
    MAa → 1, FAa → 1,
    FAA → 1 - 4 / 10,
    Faa → 1 - 4 / 10
  };
```

No recombination eigenvalues

```
WAinvA = λmA1 /. reverse /. pAveM → (1 - q) pXm + q pYm /. equilA0 /. params // Simplify;
WainvA = λma1 /. reverse /. pAveM → (1 - q) pXm + q pYm /. equilA0 /. params // Simplify;
WAinvB = λmA1 /. reverse /. pAveM → (1 - q) pXm + q pYm /. equilB0 /. params // Simplify;
WainvB = λma1 /. reverse /. pAveM → (1 - q) pXm + q pYm /. equilB0 /. params // Simplify;
```

Maximum absolute no recombination eigenvalue from the full characteristic polynomial

```
λWsolA =
  Max[Abs[λ /. Solve[0 == charpolyk1 /. r → 0 /. R → 0 /. ρ → 0 /. equilA0 /. params, λ] //
      Simplify]];
λWsolB = Max[Abs[λ /. Solve[0 == charpolyk1 /. r → 0 /. R → 0 /. ρ → 0 /. equilB0 /.
         params, λ] // Simplify]];
```

Plot

Region plots of invasion

```
(*neo-WA invades XY from equilA*)
plotWAinvA =
  RegionPlot[{
     (validcondA /. params) && (*valid*)
      (stabcondA /. Rf → 0 /. Rm → 0 /. equilA0 /. params) && (*internally stable*)
      1 < WAinvA (*invasion*)
    },
    {Maa, 0, 3 / 2}, {MAA, 0, 3 / 2},
    PlotStyle → {Gray, Opacity[0.5]},
    BoundaryStyle → None
  ];

(*neo-Wa invades XY from equilA*)
plotWainvA =
  RegionPlot[{
     (validcondA /. params) &&
      (stabcondA /. Rf → 0 /. Rm → 0 /. equilA0 /. params) &&
      1 < WainvA
    },
    {Maa, 0, 3 / 2}, {MAA, 0, 3 / 2},
    PlotStyle → {Gray, Opacity[0.5]},
    BoundaryStyle → None
  ];
```

```
(*neo-WA invades XY from equilB*)
plotWAinvB =
  RegionPlot[{
    (stabcondB /. Rf → 0 /. Rm → 0 /. equilB0 /. params) && (*internally stable*)
     1 < WAinvB(*invasion*)
   },
   {Maa, 0, 3 / 2}, {MAA, 0, 3 / 2},
   PlotStyle → {Gray, Opacity[0.5]},
   BoundaryStyle → None
  ];

(*neo-Wa invades XY from equilB*)
plotWainvB =
  RegionPlot[{
    (stabcondB /. Rf → 0 /. Rm → 0 /. equilB0 /. params) &&
     1 < WainvB
   },
   {Maa, 0, 3 / 2}, {MAA, 0, 3 / 2},
   PlotStyle → {Gray, Opacity[0.5]},
   BoundaryStyle → None
  ];

(*Ya equilibrium internally stable*)
plotYaStable =
  RegionPlot[{
    (stabcondA /. Rf → 0 /. Rm → 0 /. equilA0 /. params) ||
     (stabcondB /. Rf → 0 /. Rm → 0 /. equilB0 /. params)
   },
   {Maa, 0, 3 / 2}, {MAA, 0, 3 / 2},
   PlotStyle → White,
   BoundaryStyle → {Black, Thick}
  ];

(*Ya equilibrium internally unstable*)
plotYaUnstable =
  RegionPlot[{
    1 < 2
   },
   {Maa, 0, 3 / 2}, {MAA, 0, 3 / 2},
   PlotStyle → None,
   BoundaryStyle → None,
   Mesh → 50,
   MeshFunctions → {#1 - #2 &},
   MeshStyle → Black
  ];
```

```
plotC =
 Show[

  plotYaUnstable,
  plotYaStable,
  plotWAinvA,
  plotWainvA,
  plotWAinvB,
  plotWainvB,

  PlotRange → {{0, 1.5}, {0, 1.5}},
  ImageSize → {xsize, xsize},
  PlotRangePadding → 0,
  FrameTicks → {Table[{x, x, ticksize}, {x, 0, 1, 1}],
    Table[{y, y, ticksize}, {y, 0, 1, 1}], None, None},
  FrameTicksStyle → {{Directive[Black, Thickness[lwd]],
     Directive[Black, Thickness[lwd]]}, {Directive[Black, Thickness[lwd]],
     Directive[Black, Thickness[lwd], FontColor → White]}},
  FrameStyle → {{Black, Thickness[lwd]}, {Black, Thickness[lwd]},
    {Black, Thickness[lwd]}, {Black, Thickness[lwd]}},
  (*FrameLabel→{"Relative fitness of aa males",""},*)
  BaseStyle → {FontFamily → "Helvetica", FontSize → 14},
  ImagePadding → Pad,
  Epilog → {
    Text[Style["C", 14, Bold], Scaled@{0.05, 0.95}],
    Text[Style["Λ_{W'A}^{(XY)} >1
&
Λ_{W'a}^{(XY)} >1", 14], {0.45, 0.5}, {1, 0}],
    Text[Style["Λ_{W'A}^{(XY)} >1 & Λ_{W'a}^{(XY)} <1", 14], {1.1, 0.5}](*,
    Rotate[
     Text[Style["Relative fitness of AA males",14],Scaled@ylabpos],90 Degree]*)
   },
  PlotLabel → Style["overdominance in females", 16, Black, Bold],
  PlotRangeClipping → False
 ]
```

overdominance in females

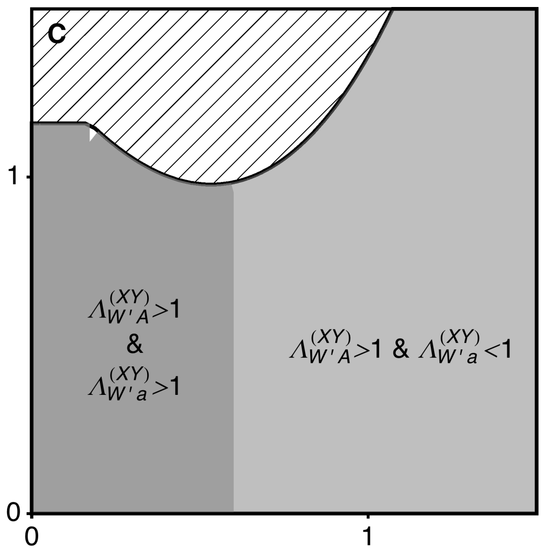

Notice that this is consistent with the solution from the full characteristic polynomial (but here we don't know which eigenvalue belongs to which haplotype)

```
(*neo-W invades XY from equilA*)
plotWinvA =
  RegionPlot[{
    (validcondA /. params) && (*valid*)
     (stabcondA /. Rf → 0 /. Rm → 0 /. equilA0 /. params) && (*internally stable*)
     1 < λWsolA (*invasion*)
   },
   {Maa, 0, 3 / 2}, {MAA, 0, 3 / 2},
   PlotStyle → {Gray, Opacity[0.5]},
   BoundaryStyle → None
  ];

(*neo-W invades XY from equilB*)
plotWinvB =
  RegionPlot[{
    (stabcondB /. Rf → 0 /. Rm → 0 /. equilB0 /. params) && (*internally stable*)
     1 < λWsolB(*invasion*)
   },
   {Maa, 0, 3 / 2}, {MAA, 0, 3 / 2},
   PlotStyle → {Gray, Opacity[0.5]},
   BoundaryStyle → None
  ];
```

```
Show[

 plotWinvA,
 plotWinvB,
 plotYaStable,

 PlotRange → {{0, 1.5}, {0, 1.5}},
 ImageSize → {xsize, xsize},
 PlotRangePadding → 0,
 FrameTicks → {Table[{x, x, ticksize}, {x, 0, 1, 1}],
   Table[{y, y, ticksize}, {y, 0, 1, 1}], None, None},
 FrameTicksStyle → {{Directive[Black, Thickness[lwd]],
    Directive[Black, Thickness[lwd]]}, {Directive[Black, Thickness[lwd]],
    Directive[Black, Thickness[lwd], FontColor → White]}},
 FrameStyle → {{Black, Thickness[lwd]}, {Black, Thickness[lwd]},
   {Black, Thickness[lwd]}, {Black, Thickness[lwd]}},
 FrameLabel → {"Relative fitness of aa males", ""},
 BaseStyle → {FontFamily → "Helvetica", FontSize → 14},
 ImagePadding → Pad,
 Epilog → {
   Text[Style["Ya equilibrium unstable", 14], {0.5, 1.25}],
   Rotate[
    Text[Style["Relative fitness of AA males", 14], Scaled@ylabpos], 90 Degree]
  },
 PlotLabel → Style["A favoured in females", 16, Black, Bold],
 PlotRangeClipping → False
]
```

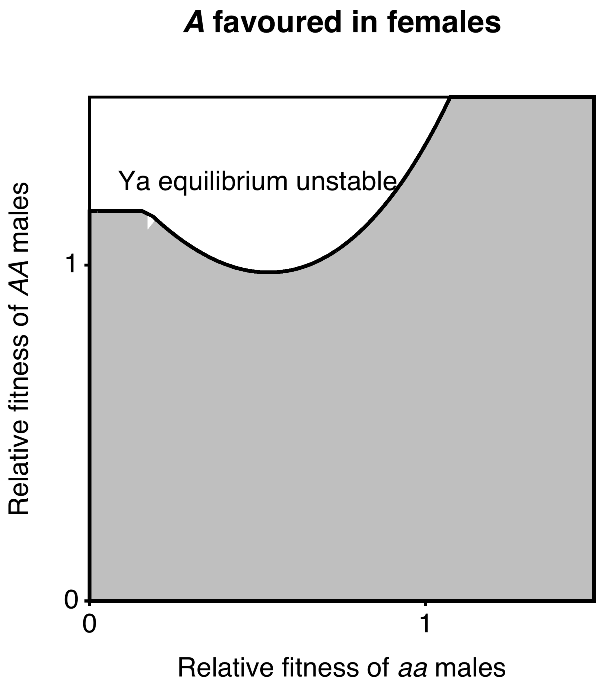

Panel D - A favoured in females, haploid competition favours A in males

Parameters

```
params = {
    wAm → 1 + 8 / 50, wam → 1, wAf → 1, waf → 1,
    αm → 1 / 2, αf → 1 / 2,
    MAa → 1, FAa → 1,
    Faa → 1 - 3 / 20,
    FAA → 1 + 1 / 20
  };
```

No recombination eigenvalues

```
WAinvA = λmA1 /. reverse /. pAveM → (1 - q) pXm + q pYm /. equilA0 /. params // Simplify;
WainvA = λma1 /. reverse /. pAveM → (1 - q) pXm + q pYm /. equilA0 /. params // Simplify;
WAinvB = λmA1 /. reverse /. pAveM → (1 - q) pXm + q pYm /. equilB0 /. params // Simplify;
WainvB = λma1 /. reverse /. pAveM → (1 - q) pXm + q pYm /. equilB0 /. params // Simplify;
```

Maximum absolute no recombination eigenvalue from the full characteristic polynomial

```
λWsolA =
  Max[Abs[λ /. Solve[0 == charpolyk1 /. r → 0 /. R → 0 /. ρ → 0 /. equilA0 /. params, λ] //
      Simplify]];
λWsolB = Max[Abs[λ /. Solve[0 == charpolyk1 /. r → 0 /. R → 0 /. ρ → 0 /. equilB0 /.
        params, λ] // Simplify]];
```

Plot

Region plots of invasion

```
(*neo-WA invades XY from equilA*)
plotWAinvA =
  RegionPlot[{
     (validcondA /. params) && (*valid*)
      (stabcondA /. Rf → 0 /. Rm → 0 /. equilA0 /. params) && (*internally stable*)
     1 < WAinvA (*invasion*)
    },
    {Maa, 0, 3 / 2}, {MAA, 0, 3 / 2},
    PlotStyle → {Gray, Opacity[0.5]},
    BoundaryStyle → None
  ];

(*neo-Wa invades XY from equilA*)
plotWainvA =
  RegionPlot[{
     (validcondA /. params) &&
      (stabcondA /. Rf → 0 /. Rm → 0 /. equilA0 /. params) &&
     1 < WainvA
    },
    {Maa, 0, 3 / 2}, {MAA, 0, 3 / 2},
    PlotStyle → {Gray, Opacity[0.5]},
    BoundaryStyle → None
  ];
```

```
(*neo-WA invades XY from equilB*)
plotWAinvB =
  RegionPlot[{
    (stabcondB /. Rf → 0 /. Rm → 0 /. equilB0 /. params) && (*internally stable*)
     1 < WAinvB(*invasion*)
   },
   {Maa, 0, 3 / 2}, {MAA, 0, 3 / 2},
   PlotStyle → {Gray, Opacity[0.5]},
   BoundaryStyle → None
  ];

(*neo-Wa invades XY from equilB*)
plotWainvB =
  RegionPlot[{
    (stabcondB /. Rf → 0 /. Rm → 0 /. equilB0 /. params) &&
     1 < WainvB
   },
   {Maa, 0, 3 / 2}, {MAA, 0, 3 / 2},
   PlotStyle → {Gray, Opacity[0.5]},
   BoundaryStyle → None
  ];

(*Ya equilibrium internally stable*)
plotYaStable =
  RegionPlot[{
    (stabcondA /. Rf → 0 /. Rm → 0 /. equilA0 /. params) ||
     (stabcondB /. Rf → 0 /. Rm → 0 /. equilB0 /. params)
   },
   {Maa, 0, 3 / 2}, {MAA, 0, 3 / 2},
   PlotStyle → White,
   BoundaryStyle → {Black, Thick}
  ];

(*Ya equilibrium internally unstable*)
plotYaUnstable =
  RegionPlot[{
    1 < 2
   },
   {Maa, 0, 3 / 2}, {MAA, 0, 3 / 2},
   PlotStyle → None,
   BoundaryStyle → None,
   Mesh → 50,
   MeshFunctions → {#1 - #2 &},
   MeshStyle → Black
  ];
```

```
plotD =
 Show[

  plotYaUnstable,
  plotYaStable,
  plotWAinvA,
  plotWainvA,
  plotWAinvB,
  plotWainvB,

  Graphics[{Thick, Black, Line[{{1.35, 0.5}, {1.475, 0.75}}]}],
  PlotRange → {{0, 1.5}, {0, 1.5}},
  ImageSize → {xsize, xsize},
  PlotRangePadding → 0,
  FrameTicks → {Table[{x, x, ticksize}, {x, 0, 1, 1}],
    Table[{y, y, ticksize}, {y, 0, 1, 1}], None, None},
  FrameTicksStyle → {{Directive[Black, Thickness[lwd]],
     Directive[Black, Thickness[lwd]]}, {Directive[Black, Thickness[lwd]],
     Directive[Black, Thickness[lwd], FontColor → White]}},
  FrameStyle → {{Black, Thickness[lwd]}, {Black, Thickness[lwd]},
    {Black, Thickness[lwd]}, {Black, Thickness[lwd]}},
  FrameLabel → {"Relative fitness of aa males", ""},
  BaseStyle → {FontFamily → "Helvetica", FontSize → 14},
  ImagePadding → Pad,
  Epilog → {
    Text[Style["D", 14, Bold], Scaled@{0.05, 0.95}],
    Text[Style["Λ_{W'A}^{(XY)} <1 & Λ_{W'a}^{(XY)} <1", 14], {0.5, 0.5}],
    Text[Style["Λ_{W'A}^{(XY)} >1
&
Λ_{W'a}^{(XY)} <1", 14], {1.35, 0.5}, {1, 0}],
    Rotate[
     Text[Style["Relative fitness of AA males", 14], Scaled@ylabpos], 90 Degree]
   },
  PlotLabel → Style[(*"A favoured in females"*)"", 16, Black, Bold],
  PlotRangeClipping → False
 ]
```

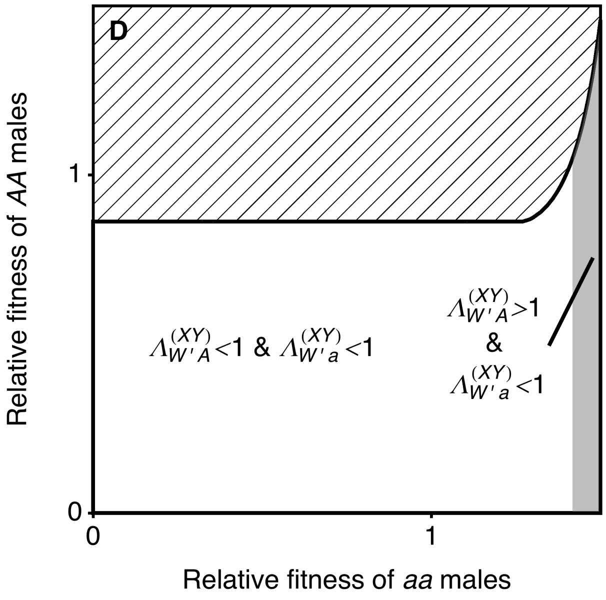

Notice that this is consistent with the solution from the full characteristic polynomial (but here we don't know which eigenvalue belongs to which haplotype)

```
(*neo-W invades XY from equilA*)
plotWinvA =
  RegionPlot[{
    (validcondA /. params) && (*valid*)
     (stabcondA /. Rf → 0 /. Rm → 0 /. equilA0 /. params) && (*internally stable*)
     1 < λWsolA (*invasion*)
   },
   {Maa, 0, 3 / 2}, {MAA, 0, 3 / 2},
   PlotStyle → {Gray, Opacity[0.5]},
   BoundaryStyle → None
  ];

(*neo-W invades XY from equilB*)
plotWinvB =
  RegionPlot[{
    (stabcondB /. Rf → 0 /. Rm → 0 /. equilB0 /. params) && (*internally stable*)
     1 < λWsolB(*invasion*)
   },
   {Maa, 0, 3 / 2}, {MAA, 0, 3 / 2},
   PlotStyle → {Gray, Opacity[0.5]},
   BoundaryStyle → None
  ];
```

```
Show[

 plotWinvA,
 plotWinvB,
 plotYaStable,

 PlotRange → {{0, 1.5}, {0, 1.5}},
 ImageSize → {xsize, xsize},
 PlotRangePadding → 0,
 FrameTicks → {Table[{x, x, ticksize}, {x, 0, 1, 1}],
   Table[{y, y, ticksize}, {y, 0, 1, 1}], None, None},
 FrameTicksStyle → {{Directive[Black, Thickness[lwd]],
    Directive[Black, Thickness[lwd]]}, {Directive[Black, Thickness[lwd]],
    Directive[Black, Thickness[lwd], FontColor → White]}},
 FrameStyle → {{Black, Thickness[lwd]}, {Black, Thickness[lwd]},
   {Black, Thickness[lwd]}, {Black, Thickness[lwd]}},
 FrameLabel → {"Relative fitness of aa males", ""},
 BaseStyle → {FontFamily → "Helvetica", FontSize → 14},
 ImagePadding → Pad,
 Epilog → {
   Text[Style["Ya equilibrium unstable", 14], {0.5, 1.25}],
   Rotate[
    Text[Style["Relative fitness of AA males", 14], Scaled@ylabpos], 90 Degree]
  },
 PlotLabel → Style["A favoured in females", 16, Black, Bold],
 PlotRangeClipping → False
]
```

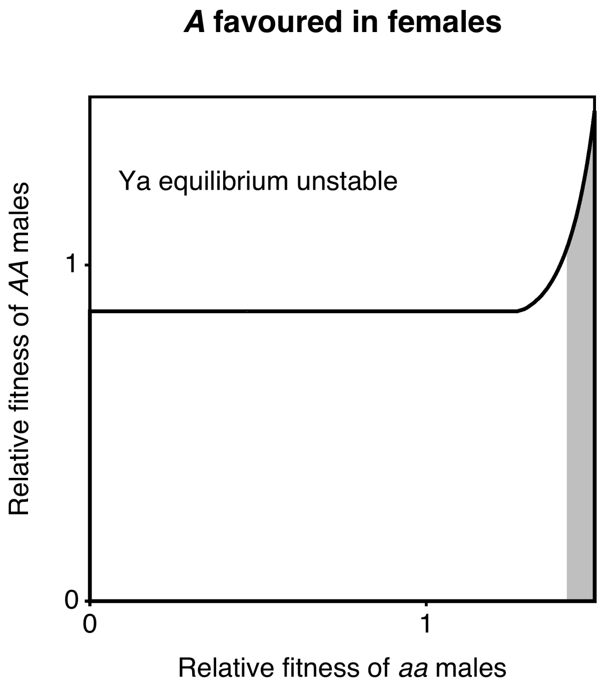

Panel E - a favoured in females, haploid competition favours A in males

Parameters

```
params = {
    wAm → 1 + 8 / 50, wam → 1, wAf → 1, waf → 1,
    αm → 1 / 2, αf → 1 / 2,
    MAa → 1, FAa → 1,
    FAA → 1 - 3 / 20,
    Faa → 1 + 1 / 20
   };
```

No recombination eigenvalues

```
WAinvA = λmA1 /. reverse /. pAveM → (1 - q) pXm + q pYm /. equilA0 /. params // Simplify;
WainvA = λma1 /. reverse /. pAveM → (1 - q) pXm + q pYm /. equilA0 /. params // Simplify;
WAinvB = λmA1 /. reverse /. pAveM → (1 - q) pXm + q pYm /. equilB0 /. params // Simplify;
WainvB = λma1 /. reverse /. pAveM → (1 - q) pXm + q pYm /. equilB0 /. params // Simplify;
```

Maximum absolute no recombination eigenvalue from the full characteristic polynomial

```
λWsolA =
  Max[Abs[λ /. Solve[0 == charpolyk1 /. r → 0 /. R → 0 /. ρ → 0 /. equilA0 /. params, λ] //
      Simplify]];
λWsolB = Max[Abs[λ /. Solve[0 == charpolyk1 /. r → 0 /. R → 0 /. ρ → 0 /. equilB0 /.
        params, λ] // Simplify]];
```

Plot

Region plots of invasion

```
(*neo-WA invades XY from equilA*)
plotWAinvA =
  RegionPlot[{
     (validcondA /. params) && (*valid*)
      (stabcondA /. Rf → 0 /. Rm → 0 /. equilA0 /. params) && (*internally stable*)
      1 < WAinvA (*invasion*)
    },
    {Maa, 0, 3 / 2}, {MAA, 0, 3 / 2},
    PlotStyle → {Gray, Opacity[0.5]},
    BoundaryStyle → None
  ];

(*neo-Wa invades XY from equilA*)
plotWainvA =
  RegionPlot[{
     (validcondA /. params) &&
      (stabcondA /. Rf → 0 /. Rm → 0 /. equilA0 /. params) &&
      1 < WainvA
    },
    {Maa, 0, 3 / 2}, {MAA, 0, 3 / 2},
    PlotStyle → {Gray, Opacity[0.5]},
    BoundaryStyle → None
  ];
```

```
(*neo-WA invades XY from equilB*)
plotWAinvB =
  RegionPlot[{
    (stabcondB /. Rf → 0 /. Rm → 0 /. equilB0 /. params) && (*internally stable*)
     1 < WAinvB(*invasion*)
   },
   {Maa, 0, 3 / 2}, {MAA, 0, 3 / 2},
   PlotStyle → {Gray, Opacity[0.5]},
   BoundaryStyle → None
  ];

(*neo-Wa invades XY from equilB*)
plotWainvB =
  RegionPlot[{
    (stabcondB /. Rf → 0 /. Rm → 0 /. equilB0 /. params) &&
     1 < WainvB
   },
   {Maa, 0, 3 / 2}, {MAA, 0, 3 / 2},
   PlotStyle → {Gray, Opacity[0.5]},
   BoundaryStyle → None
  ];

(*Ya equilibrium internally stable*)
plotYaStable =
  RegionPlot[{
    (stabcondA /. Rf → 0 /. Rm → 0 /. equilA0 /. params) ||
     (stabcondB /. Rf → 0 /. Rm → 0 /. equilB0 /. params)
   },
   {Maa, 0, 3 / 2}, {MAA, 0, 3 / 2},
   PlotStyle → White,
   BoundaryStyle → {Black, Thick}
  ];

(*Ya equilibrium internally unstable*)
plotYaUnstable =
  RegionPlot[{
    1 < 2
   },
   {Maa, 0, 3 / 2}, {MAA, 0, 3 / 2},
   PlotStyle → None,
   BoundaryStyle → None,
   Mesh → 50,
   MeshFunctions → {#1 - #2 &},
   MeshStyle → Black
  ];
```

```
plotE =
 Show[

  plotYaUnstable,
  plotYaStable,
  plotWAinvA,
  plotWainvA,
  plotWAinvB,
  plotWainvB,

  Graphics[{Thick, Black, Line[{{1.1, 0.5}, {0.95, 0.25}}]}],
  PlotRange → {{0, 1.5}, {0, 1.5}},
  ImageSize → {xsize, xsize},
  PlotRangePadding → 0,
  FrameTicks → {Table[{x, x, ticksize}, {x, 0, 1, 1}],
    Table[{y, y, ticksize}, {y, 0, 1, 1}], None, None},
  FrameTicksStyle → {{Directive[Black, Thickness[lwd]],
     Directive[Black, Thickness[lwd]]}, {Directive[Black, Thickness[lwd]],
     Directive[Black, Thickness[lwd], FontColor → White]}},
  FrameStyle → {{Black, Thickness[lwd]}, {Black, Thickness[lwd]},
    {Black, Thickness[lwd]}, {Black, Thickness[lwd]}},
  FrameLabel → {"Relative fitness of aa males", ""},
  BaseStyle → {FontFamily → "Helvetica", FontSize → 14},
  ImagePadding → Pad,
  Epilog → {
    Text[Style["E", 14, Bold], Scaled@{0.05, 0.95}],
    Text[Style["Λ(XY)W'A >1 & Λ(XY)W'a >1", 14], {0.4, 0.5}],
    Text[Style["Λ(XY)W'A <1
&
Λ(XY)W'a >1", 14], {1.1, 0.5}, {-1, 0}, Background → White](*,
    Rotate[
     Text[Style["Relative fitness of AA males",14],Scaled@ylabpos],90 Degree]*)
   },
  PlotLabel → Style[(*"a favoured in females"*)"", 16, Black, Bold],
  PlotRangeClipping → False
 ]
```

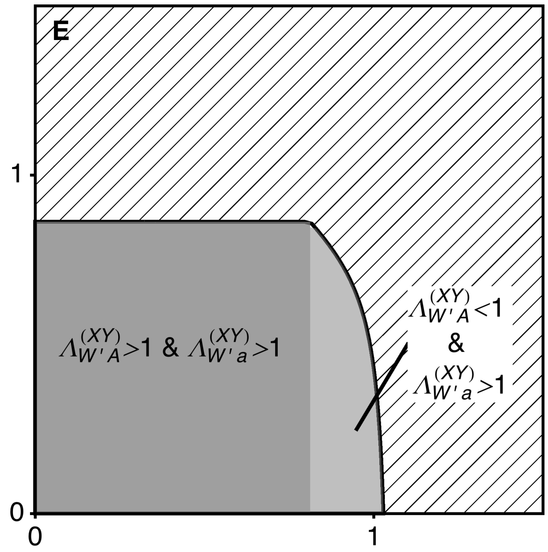

Notice that this is consistent with the solution from the full characteristic polynomial (but here we don't know which eigenvalue belongs to which haplotype)

```
(*neo-W invades XY from equilA*)
plotWinvA =
  RegionPlot[{
    (validcondA /. params) && (*valid*)
     (stabcondA /. Rf → 0 /. Rm → 0 /. equilA0 /. params) && (*internally stable*)
     1 < λWsolA (*invasion*)
   },
   {Maa, 0, 3 / 2}, {MAA, 0, 3 / 2},
   PlotStyle → {Gray, Opacity[0.5]},
   BoundaryStyle → None
  ];

(*neo-W invades XY from equilB*)
plotWinvB =
  RegionPlot[{
    (stabcondB /. Rf → 0 /. Rm → 0 /. equilB0 /. params) && (*internally stable*)
     1 < λWsolB(*invasion*)
   },
   {Maa, 0, 3 / 2}, {MAA, 0, 3 / 2},
   PlotStyle → {Gray, Opacity[0.5]},
   BoundaryStyle → None
  ];
```

```
Show[

 plotWinvA,
 plotWinvB,
 plotYaStable,

 PlotRange → {{0, 1.5}, {0, 1.5}},
 ImageSize → {xsize, xsize},
 PlotRangePadding → 0,
 FrameTicks → {Table[{x, x, ticksize}, {x, 0, 1, 1}],
   Table[{y, y, ticksize}, {y, 0, 1, 1}], None, None},
 FrameTicksStyle → {{Directive[Black, Thickness[lwd]],
    Directive[Black, Thickness[lwd]]}, {Directive[Black, Thickness[lwd]],
    Directive[Black, Thickness[lwd], FontColor → White]}},
 FrameStyle → {{Black, Thickness[lwd]}, {Black, Thickness[lwd]},
   {Black, Thickness[lwd]}, {Black, Thickness[lwd]}},
 FrameLabel → {"Relative fitness of aa males", ""},
 BaseStyle → {FontFamily → "Helvetica", FontSize → 14},
 ImagePadding → Pad,
 Epilog → {
   Text[Style["Ya equilibrium unstable", 14], {0.5, 1.25}],
   Rotate[
    Text[Style["Relative fitness of AA males", 14], Scaled@ylabpos], 90 Degree]
  },
 PlotLabel → Style["a favoured in females", 16, Black, Bold],
 PlotRangeClipping → False
]
```

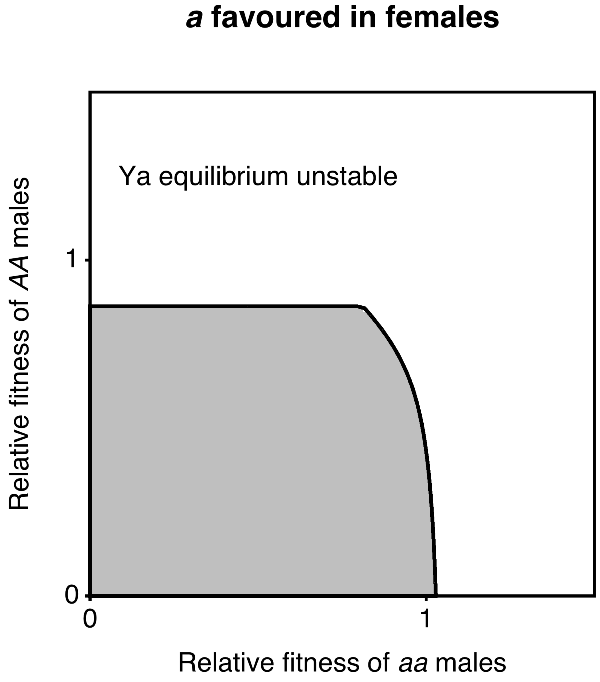

Panel F - overdominance in females, haploid competition favours A in males

Parameters

```
params = {
    wAm → 1 + 8 / 50, wam → 1, wAf → 1, waf → 1,
    αm → 1 / 2, αf → 1 / 2,
    MAa → 1, FAa → 1,
    FAA → 1 - 4 / 10,
    Faa → 1 - 4 / 10
  };
```

No recombination eigenvalues

```
WAinvA = λmA1 /. reverse /. pAveM → (1 - q) pXm + q pYm /. equilA0 /. params // Simplify;
WainvA = λma1 /. reverse /. pAveM → (1 - q) pXm + q pYm /. equilA0 /. params // Simplify;
WAinvB = λmA1 /. reverse /. pAveM → (1 - q) pXm + q pYm /. equilB0 /. params // Simplify;
WainvB = λma1 /. reverse /. pAveM → (1 - q) pXm + q pYm /. equilB0 /. params // Simplify;
```

Maximum absolute no recombination eigenvalue from the full characteristic polynomial

```
λWsolA =
  Max[Abs[λ /. Solve[0 == charpolyk1 /. r → 0 /. R → 0 /. ρ → 0 /. equilA0 /. params, λ] //
      Simplify]];
λWsolB = Max[Abs[λ /. Solve[0 == charpolyk1 /. r → 0 /. R → 0 /. ρ → 0 /. equilB0 /.
         params, λ] // Simplify]];
```

Plot

Region plots of invasion

```
(*neo-WA invades XY from equilA*)
plotWAinvA =
  RegionPlot[{
     (validcondA /. params) && (*valid*)
      (stabcondA /. Rf → 0 /. Rm → 0 /. equilA0 /. params) && (*internally stable*)
      1 < WAinvA (*invasion*)
    },
    {Maa, 0, 3 / 2}, {MAA, 0, 3 / 2},
    PlotStyle → {Gray, Opacity[0.5]},
    BoundaryStyle → None
  ];

(*neo-Wa invades XY from equilA*)
plotWainvA =
  RegionPlot[{
     (validcondA /. params) &&
      (stabcondA /. Rf → 0 /. Rm → 0 /. equilA0 /. params) &&
      1 < WainvA
    },
    {Maa, 0, 3 / 2}, {MAA, 0, 3 / 2},
    PlotStyle → {Gray, Opacity[0.5]},
    BoundaryStyle → None
  ];
```

```
(*neo-WA invades XY from equilB*)
plotWAinvB =
  RegionPlot[{
    (stabcondB /. Rf → 0 /. Rm → 0 /. equilB0 /. params) && (*internally stable*)
     1 < WAinvB(*invasion*)
   },
   {Maa, 0, 3 / 2}, {MAA, 0, 3 / 2},
   PlotStyle → {Gray, Opacity[0.5]},
   BoundaryStyle → None
  ];

(*neo-Wa invades XY from equilB*)
plotWainvB =
  RegionPlot[{
    (stabcondB /. Rf → 0 /. Rm → 0 /. equilB0 /. params) &&
     1 < WainvB
   },
   {Maa, 0, 3 / 2}, {MAA, 0, 3 / 2},
   PlotStyle → {Gray, Opacity[0.5]},
   BoundaryStyle → None
  ];

(*Ya equilibrium internally stable*)
plotYaStable =
  RegionPlot[{
    (stabcondA /. Rf → 0 /. Rm → 0 /. equilA0 /. params) ||
     (stabcondB /. Rf → 0 /. Rm → 0 /. equilB0 /. params)
   },
   {Maa, 0, 3 / 2}, {MAA, 0, 3 / 2},
   PlotStyle → White,
   BoundaryStyle → {Black, Thick}
  ];

(*Ya equilibrium internally unstable*)
plotYaUnstable =
  RegionPlot[{
    1 < 2
   },
   {Maa, 0, 3 / 2}, {MAA, 0, 3 / 2},
   PlotStyle → None,
   BoundaryStyle → None,
   Mesh → 50,
   MeshFunctions → {#1 - #2 &},
   MeshStyle → Black
  ];
```

```
plotF =
 Show[

  plotYaUnstable,
  plotYaStable,
  plotWAinvA,
  plotWainvA,
  plotWAinvB,
  plotWainvB,

  PlotRange → {{0, 1.5}, {0, 1.5}},
  ImageSize → {xsize, xsize},
  PlotRangePadding → 0,
  FrameTicks → {Table[{x, x, ticksize}, {x, 0, 1, 1}],
    Table[{y, y, ticksize}, {y, 0, 1, 1}], None, None},
  FrameTicksStyle → {{Directive[Black, Thickness[lwd]],
     Directive[Black, Thickness[lwd]]}, {Directive[Black, Thickness[lwd]],
     Directive[Black, Thickness[lwd], FontColor → White]}},
  FrameStyle → {{Black, Thickness[lwd]}, {Black, Thickness[lwd]},
    {Black, Thickness[lwd]}, {Black, Thickness[lwd]}},
  FrameLabel → {"Relative fitness of aa males", ""},
  BaseStyle → {FontFamily → "Helvetica", FontSize → 14},
  ImagePadding → Pad,
  Epilog → {
    Text[Style["F", 14, Bold], Scaled@{0.05, 0.95}],
    Text[Style["Λ_{W'A}^(XY) >1
&
Λ_{W'a}^(XY) >1", 14], {0.4, 0.4}, {1, 0}],
    Text[Style["Λ_{W'A}^(XY) <1 & Λ_{W'a}^(XY) >1", 14], {1, 0.4}](*,
    Rotate[
     Text[Style["Relative fitness of AA males",14],Scaled@ylabpos],90 Degree]*)
   },
  PlotLabel → Style[(*"overdominance in females"*)"", 16, Black, Bold],
  PlotRangeClipping → False
 ]
```

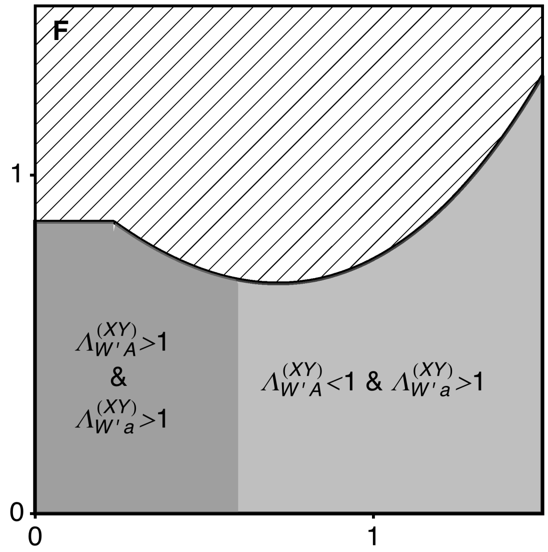

Notice that this is consistent with the solution from the full characteristic polynomial (but here we don't know which eigenvalue belongs to which haplotype)

```
(*neo-W invades XY from equilA*)
plotWinvA =
  RegionPlot[{
    (validcondA /. params) && (*valid*)
     (stabcondA /. Rf → 0 /. Rm → 0 /. equilA0 /. params) && (*internally stable*)
     1 < λWsolA (*invasion*)
   },
   {Maa, 0, 3 / 2}, {MAA, 0, 3 / 2},
   PlotStyle → {Gray, Opacity[0.5]},
   BoundaryStyle → None
  ];

(*neo-W invades XY from equilB*)
plotWinvB =
  RegionPlot[{
    (stabcondB /. Rf → 0 /. Rm → 0 /. equilB0 /. params) && (*internally stable*)
     1 < λWsolB(*invasion*)
   },
   {Maa, 0, 3 / 2}, {MAA, 0, 3 / 2},
   PlotStyle → {Gray, Opacity[0.5]},
   BoundaryStyle → None
  ];
```

```
Show[

 plotWinvA,
 plotWinvB,
 plotYaStable,

 PlotRange → {{0, 1.5}, {0, 1.5}},
 ImageSize → {xsize, xsize},
 PlotRangePadding → 0,
 FrameTicks → {Table[{x, x, ticksize}, {x, 0, 1, 1}],
   Table[{y, y, ticksize}, {y, 0, 1, 1}], None, None},
 FrameTicksStyle → {{Directive[Black, Thickness[lwd]],
    Directive[Black, Thickness[lwd]]}, {Directive[Black, Thickness[lwd]],
    Directive[Black, Thickness[lwd], FontColor → White]}},
 FrameStyle → {{Black, Thickness[lwd]}, {Black, Thickness[lwd]},
   {Black, Thickness[lwd]}, {Black, Thickness[lwd]}},
 FrameLabel → {"Relative fitness of aa males", ""},
 BaseStyle → {FontFamily → "Helvetica", FontSize → 14},
 ImagePadding → Pad,
 Epilog → {
   Text[Style["Ya equilibrium unstable", 14], {0.5, 1.25}],
   Rotate[
    Text[Style["Relative fitness of AA males", 14], Scaled@ylabpos], 90 Degree]
  },
 PlotLabel → Style["overdominance in females", 16, Black, Bold],
 PlotRangeClipping → False
]
```

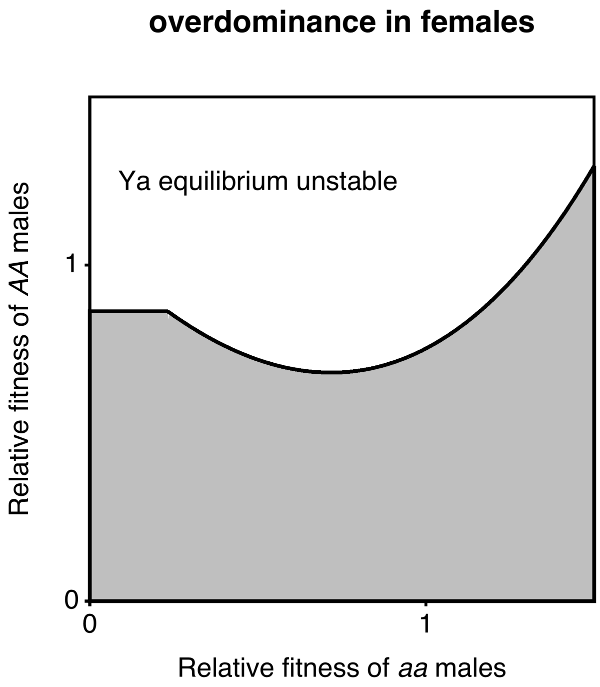

All Panels

```
GraphicsGrid[{
   {plotA, plotB, plotC},
   {plotD, plotE, plotF}
  },
 Spacings → -50
]

Export[plotdir <> "Region_plot_combined_MaleGS.eps", % // rasterTrick];
```

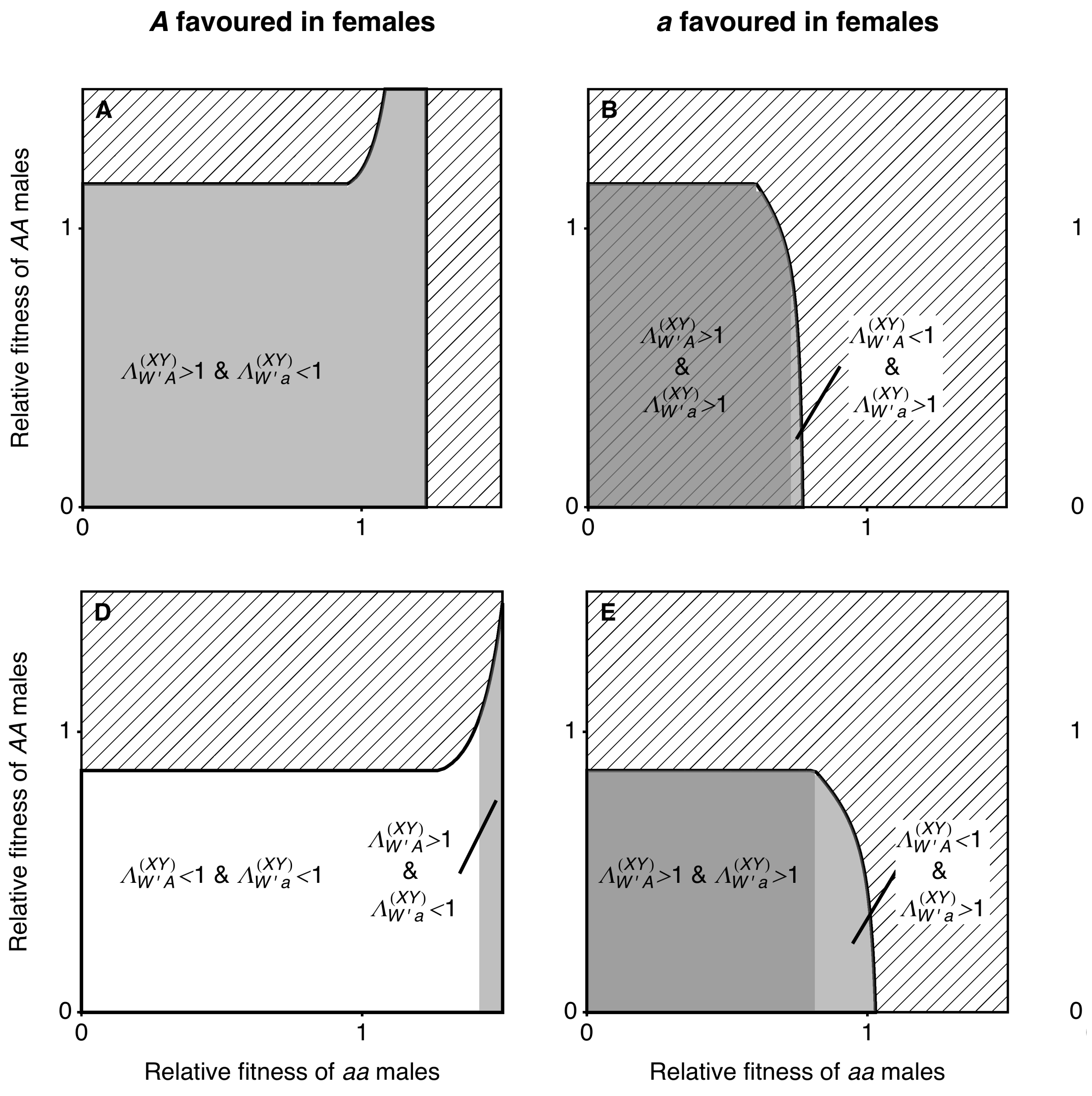

Figure S.6 - neo-W haplotype invasion with meiotic drive in females

Panel A - A favoured in females, a drives in females

Parameters

```
params = {
    wAm → 1, wam → 1, wAf → 1, waf → 1,
    αf → 1 / 2 - 8 / 100,
    αm → 1 / 2,
    MAa → 1, FAa → 1,
    Faa → 1 - 3 / 20,
    FAA → 1 + 1 / 20
  };
```

Making the sex-ratio

```
freqMale /. equilB0 /. params // N
```

```
0.5
```

No recombination eigenvalues

```
WAinvA = λmA1 /. reverse /. pAveM → (1 - q) pXm + q pYm /. equilA0 /. params // Simplify;
WainvA = λma1 /. reverse /. pAveM → (1 - q) pXm + q pYm /. equilA0 /. params // Simplify;
WAinvB = λmA1 /. reverse /. pAveM → (1 - q) pXm + q pYm /. equilB0 /. params // Simplify;
WainvB = λma1 /. reverse /. pAveM → (1 - q) pXm + q pYm /. equilB0 /. params // Simplify;
```

Maximum absolute no recombination eigenvalue from the full characteristic polynomial

```
λWsolA =
  Max[Abs[λ /. Solve[0 == charpolyk1 /. r → 0 /. R → 0 /. ρ → 0 /. equilA0 /. params, λ] //
      Simplify]];
λWsolB = Max[Abs[λ /. Solve[0 == charpolyk1 /. r → 0 /. R → 0 /. ρ → 0 /. equilB0 /.
        params, λ] // Simplify]];
```

Plot

Region plots of invasion

```
(*neo-WA invades XY from equilA*)
plotWAinvA =
  RegionPlot[{
    (validcondA /. params) && (*valid*)
     (stabcondA /. Rf → 0 /. Rm → 0 /. equilA0 /. params) && (*internally stable*)
     1 < WAinvA (*invasion*)
   },
   {Maa, 0, 3 / 2}, {MAA, 0, 3 / 2},
   PlotStyle → {Gray, Opacity[0.5]},
   BoundaryStyle → None
  ];
```

```
(*neo-Wa invades XY from equilA*)
plotWainvA =
  RegionPlot[{
    (validcondA /. params) &&
     (stabcondA /. Rf → 0 /. Rm → 0 /. equilA0 /. params) &&
     1 < WainvA
   },
   {Maa, 0, 3 / 2}, {MAA, 0, 3 / 2},
   PlotStyle → {Gray, Opacity[0.5]},
   BoundaryStyle → None
  ];

(*neo-WA invades XY from equilB*)
plotWAinvB =
  RegionPlot[{
    (stabcondB /. Rf → 0 /. Rm → 0 /. equilB0 /. params) && (*internally stable*)
     1 < WAinvB(*invasion*)
   },
   {Maa, 0, 3 / 2}, {MAA, 0, 3 / 2},
   PlotStyle → {Gray, Opacity[0.5]},
   BoundaryStyle → None
  ];

(*neo-Wa invades XY from equilB*)
plotWainvB =
  RegionPlot[{
    (stabcondB /. Rf → 0 /. Rm → 0 /. equilB0 /. params) &&
     1 < WainvB
   },
   {Maa, 0, 3 / 2}, {MAA, 0, 3 / 2},
   PlotStyle → {Gray, Opacity[0.5]},
   BoundaryStyle → None
  ];

(*Ya equilibrium internally stable*)
plotYaStable =
  RegionPlot[{
    (stabcondA /. Rf → 0 /. Rm → 0 /. equilA0 /. params) ||
     (stabcondB /. Rf → 0 /. Rm → 0 /. equilB0 /. params)
   },
   {Maa, 0, 3 / 2}, {MAA, 0, 3 / 2},
   PlotStyle → White,
   BoundaryStyle → {Black, Thick}
  ];

(*Ya equilibrium internally unstable*)
plotYaUnstable =
  RegionPlot[{
    1 < 2
   },
   {Maa, 0, 3 / 2}, {MAA, 0, 3 / 2},
   PlotStyle → None,
   BoundaryStyle → None,
   Mesh → 50,
```

```
    MeshFunctions → {#1 - #2 &},
    MeshStyle → Black
   ];

plotA =
  Show[

   plotYaUnstable,
   plotYaStable,
   plotWAinvA,
   plotWainvA,
   plotWAinvB,
   plotWainvB,

   PlotRange → {{0, 1.5}, {0, 1.5}},
   ImageSize → {xsize, xsize},
   PlotRangePadding → 0,
   FrameTicks → {Table[{x, x, ticksize}, {x, 0, 1, 1}],
     Table[{y, y, ticksize}, {y, 0, 1, 1}], None, None},
   FrameTicksStyle → {{Directive[Black, Thickness[lwd]],
      Directive[Black, Thickness[lwd]]}, {Directive[Black, Thickness[lwd]],
      Directive[Black, Thickness[lwd], FontColor → White]}},
   FrameStyle → {{Black, Thickness[lwd]}, {Black, Thickness[lwd]},
     {Black, Thickness[lwd]}, {Black, Thickness[lwd]}},
   (*FrameLabel→{"Relative fitness of aa males",""},*)
   BaseStyle → {FontFamily → "Helvetica", FontSize → 14},
   ImagePadding → Pad,
   Epilog → {
     Text[Style["A", 14, Bold], Scaled@{0.05, 0.95}],
     Text[Style["Λ_{W'A}^(XY) <1 & Λ_{W'a}^(XY) <1", 14], {0.5, 0.5}],
     Rotate[
      Text[Style["Relative fitness of AA males", 14], Scaled@ylabpos], 90 Degree]
    },
   PlotLabel → Style["A favoured in females", 16, Black, Bold],
   PlotRangeClipping → False
  ]
```

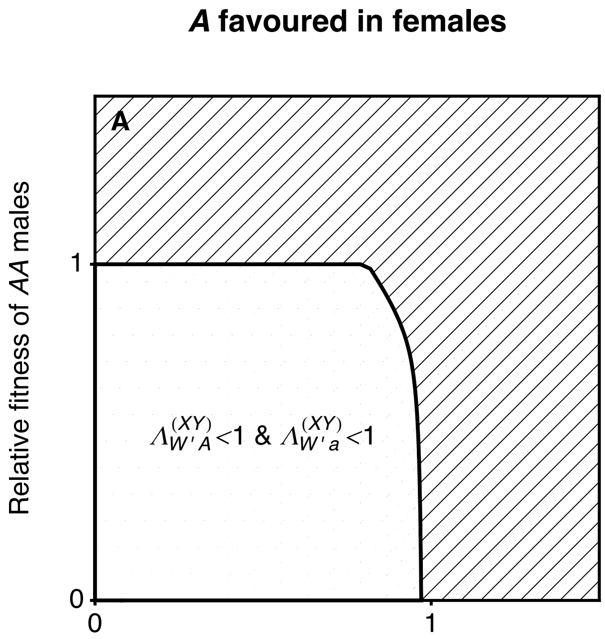

Notice that this is consistent with the solution from the full characteristic polynomial (but here we don’t know which eigenvalue belongs to which haplotype)

```
(*neo-W invades XY from equilA*)
plotWinvA =
  RegionPlot[{
    (validcondA /. params) && (*valid*)
     (stabcondA /. Rf → 0 /. Rm → 0 /. equilA0 /. params) && (*internally stable*)
     1 < λWsolA (*invasion*)
   },
   {Maa, 0, 3 / 2}, {MAA, 0, 3 / 2},
   PlotStyle → {Gray, Opacity[0.5]},
   BoundaryStyle → None
  ];

(*neo-W invades XY from equilB*)
plotWinvB =
  RegionPlot[{
    (stabcondB /. Rf → 0 /. Rm → 0 /. equilB0 /. params) && (*internally stable*)
     1 < λWsolB(*invasion*)
   },
   {Maa, 0, 3 / 2}, {MAA, 0, 3 / 2},
   PlotStyle → {Gray, Opacity[0.5]},
   BoundaryStyle → None
  ];
```

```
Show[

 plotWinvA,
 plotWinvB,
 plotYaStable,

 PlotRange → {{0, 1.5}, {0, 1.5}},
 ImageSize → {xsize, xsize},
 PlotRangePadding → 0,
 FrameTicks → {Table[{x, x, ticksize}, {x, 0, 1, 1}],
   Table[{y, y, ticksize}, {y, 0, 1, 1}], None, None},
 FrameTicksStyle → {{Directive[Black, Thickness[lwd]],
    Directive[Black, Thickness[lwd]]}, {Directive[Black, Thickness[lwd]],
    Directive[Black, Thickness[lwd], FontColor → White]}},
 FrameStyle → {{Black, Thickness[lwd]}, {Black, Thickness[lwd]},
   {Black, Thickness[lwd]}, {Black, Thickness[lwd]}},
 FrameLabel → {"Relative fitness of aa males", ""},
 BaseStyle → {FontFamily → "Helvetica", FontSize → 14},
 ImagePadding → Pad,
 Epilog → {
   Text[Style["Ya equilibrium unstable", 14], {0.5, 1.25}],
   Rotate[
    Text[Style["Relative fitness of AA males", 14], Scaled@ylabpos], 90 Degree]
  },
 PlotLabel → Style["A favoured in females", 16, Black, Bold],
 PlotRangeClipping → False
]
```

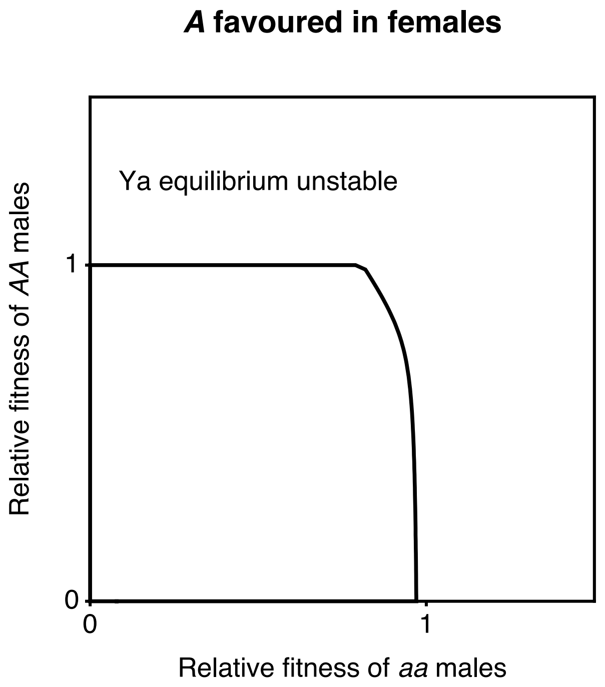

Panel B - a favoured in females, a drives in females

Parameters

```
params = {
    wAm → 1, wam → 1, wAf → 1, waf → 1,
    αf → 1 / 2 - 8 / 100,
    αm → 1 / 2,
    MAa → 1, FAa → 1,
    FAA → 1 - 3 / 20,
    Faa → 1 + 1 / 20
  };
```

No recombination eigenvalues

```
WAinvA = λmA1 /. reverse /. pAveM → (1 - q) pXm + q pYm /. equilA0 /. params // Simplify;
WainvA = λma1 /. reverse /. pAveM → (1 - q) pXm + q pYm /. equilA0 /. params // Simplify;
WAinvB = λmA1 /. reverse /. pAveM → (1 - q) pXm + q pYm /. equilB0 /. params // Simplify;
WainvB = λma1 /. reverse /. pAveM → (1 - q) pXm + q pYm /. equilB0 /. params // Simplify;
```

Maximum absolute no recombination eigenvalue from the full characteristic polynomial

```
λWsolA =
  Max[Abs[λ /. Solve[0 == charpolyk1 /. r → 0 /. R → 0 /. ρ → 0 /. equilA0 /. params, λ] //
      Simplify]];
λWsolB = Max[Abs[λ /. Solve[0 == charpolyk1 /. r → 0 /. R → 0 /. ρ → 0 /. equilB0 /.
          params, λ] // Simplify]];
```

Plot

Region plots of invasion

```
(*neo-WA invades XY from equilA*)
plotWAinvA =
  RegionPlot[{
     (validcondA /. params) && (*valid*)
      (stabcondA /. Rf → 0 /. Rm → 0 /. equilA0 /. params) && (*internally stable*)
      1 < WAinvA (*invasion*)
    },
    {Maa, 0, 3 / 2}, {MAA, 0, 3 / 2},
    PlotStyle → {Gray, Opacity[0.5]},
    BoundaryStyle → None
  ];

(*neo-Wa invades XY from equilA*)
plotWainvA =
  RegionPlot[{
     (validcondA /. params) &&
      (stabcondA /. Rf → 0 /. Rm → 0 /. equilA0 /. params) &&
      1 < WainvA
    },
    {Maa, 0, 3 / 2}, {MAA, 0, 3 / 2},
    PlotStyle → {Gray, Opacity[0.5]},
    BoundaryStyle → None
  ];
```

```
(*neo-WA invades XY from equilB*)
plotWAinvB =
  RegionPlot[{
    (stabcondB /. Rf → 0 /. Rm → 0 /. equilB0 /. params) && (*internally stable*)
     1 < WAinvB(*invasion*)
   },
   {Maa, 0, 3 / 2}, {MAA, 0, 3 / 2},
   PlotStyle → {Gray, Opacity[0.5]},
   BoundaryStyle → None
  ];

(*neo-Wa invades XY from equilB*)
plotWainvB =
  RegionPlot[{
    (stabcondB /. Rf → 0 /. Rm → 0 /. equilB0 /. params) &&
     1 < WainvB
   },
   {Maa, 0, 3 / 2}, {MAA, 0, 3 / 2},
   PlotStyle → {Gray, Opacity[0.5]},
   BoundaryStyle → None
  ];

(*Ya equilibrium internally stable*)
plotYaStable =
  RegionPlot[{
    (stabcondA /. Rf → 0 /. Rm → 0 /. equilA0 /. params) ||
     (stabcondB /. Rf → 0 /. Rm → 0 /. equilB0 /. params)
   },
   {Maa, 0, 3 / 2}, {MAA, 0, 3 / 2},
   PlotStyle → White,
   BoundaryStyle → {Black, Thick}
  ];

(*Ya equilibrium internally unstable*)
plotYaUnstable =
  RegionPlot[{
    1 < 2
   },
   {Maa, 0, 3 / 2}, {MAA, 0, 3 / 2},
   PlotStyle → None,
   BoundaryStyle → None,
   Mesh → 50,
   MeshFunctions → {#1 - #2 &},
   MeshStyle → Black
  ];
```

```
plotB =
 Show[

  plotYaUnstable,
  plotYaStable,
  plotWAinvA,
  plotWainvA,
  plotWAinvB,
  plotWainvB,

  PlotRange → {{0, 1.5}, {0, 1.5}},
  ImageSize → {xsize, xsize},
  PlotRangePadding → 0,
  FrameTicks → {Table[{x, x, ticksize}, {x, 0, 1, 1}],
    Table[{y, y, ticksize}, {y, 0, 1, 1}], None, None},
  FrameTicksStyle → {{Directive[Black, Thickness[lwd]],
     Directive[Black, Thickness[lwd]]}, {Directive[Black, Thickness[lwd]],
     Directive[Black, Thickness[lwd], FontColor → White]}},
  FrameStyle → {{Black, Thickness[lwd]}, {Black, Thickness[lwd]},
    {Black, Thickness[lwd]}, {Black, Thickness[lwd]}},
  (*FrameLabel→{"Relative fitness of aa males",""},*)
  BaseStyle → {FontFamily → "Helvetica", FontSize → 14},
  ImagePadding → Pad,
  Epilog → {
    Text[Style["B", 14, Bold], Scaled@{0.05, 0.95}],
    Text[Style["Λ_{W'A}^{(XY)} <1
&
Λ_{W'a}^{(XY)} >1", 14], {0.3, 0.5}](*,
    Rotate[
     Text[Style["Relative fitness of AA males",14],Scaled@ylabpos],90 Degree]*)
   },
  PlotLabel → Style["a favoured in females", 16, Black, Bold],
  PlotRangeClipping → False
 ]
```

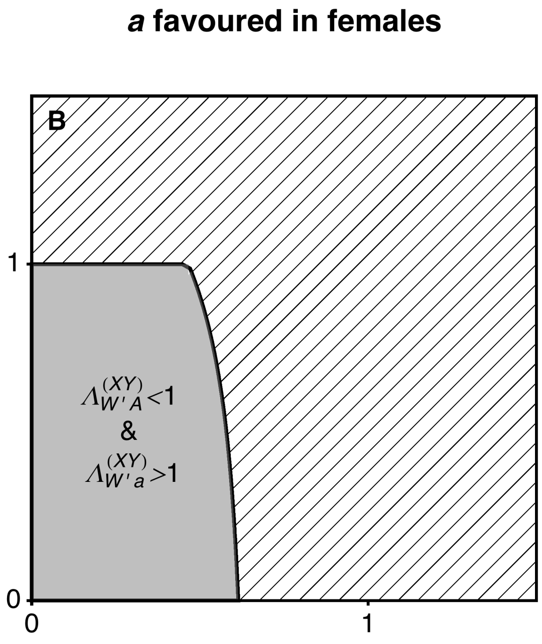

Notice that this is consistent with the solution from the full characteristic polynomial (but here we don't know which eigenvalue belongs to which haplotype)

```
(*neo-W invades XY from equilA*)
plotWinvA =
  RegionPlot[{
    (validcondA /. params) && (*valid*)
     (stabcondA /. Rf → 0 /. Rm → 0 /. equilA0 /. params) && (*internally stable*)
     1 < λWsolA (*invasion*)
   },
   {Maa, 0, 3 / 2}, {MAA, 0, 3 / 2},
   PlotStyle → {Gray, Opacity[0.5]},
   BoundaryStyle → None
  ];

(*neo-W invades XY from equilB*)
plotWinvB =
  RegionPlot[{
    (stabcondB /. Rf → 0 /. Rm → 0 /. equilB0 /. params) && (*internally stable*)
     1 < λWsolB(*invasion*)
   },
   {Maa, 0, 3 / 2}, {MAA, 0, 3 / 2},
   PlotStyle → {Gray, Opacity[0.5]},
   BoundaryStyle → None
  ];
```

```
Show[

 plotWinvA,
 plotWinvB,
 plotYaStable,

 PlotRange → {{0, 1.5}, {0, 1.5}},
 ImageSize → {xsize, xsize},
 PlotRangePadding → 0,
 FrameTicks → {Table[{x, x, ticksize}, {x, 0, 1, 1}],
   Table[{y, y, ticksize}, {y, 0, 1, 1}], None, None},
 FrameTicksStyle → {{Directive[Black, Thickness[lwd]],
    Directive[Black, Thickness[lwd]]}, {Directive[Black, Thickness[lwd]],
    Directive[Black, Thickness[lwd], FontColor → White]}},
 FrameStyle → {{Black, Thickness[lwd]}, {Black, Thickness[lwd]},
   {Black, Thickness[lwd]}, {Black, Thickness[lwd]}},
 FrameLabel → {"Relative fitness of aa males", ""},
 BaseStyle → {FontFamily → "Helvetica", FontSize → 14},
 ImagePadding → Pad,
 Epilog → {
   Text[Style["Ya equilibrium unstable", 14], {0.5, 1.25}],
   Rotate[
    Text[Style["Relative fitness of AA males", 14], Scaled@ylabpos], 90 Degree]
  },
 PlotLabel → Style["a favoured in females", 16, Black, Bold],
 PlotRangeClipping → False
]
```

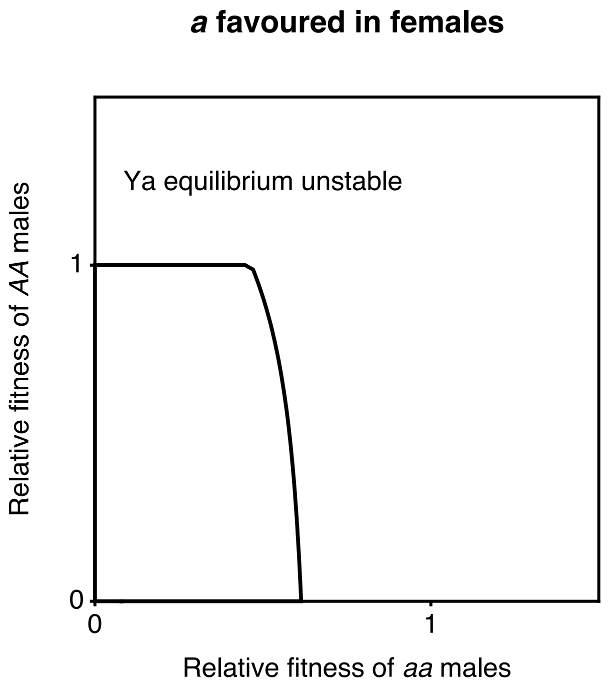

Panel C - overdominance in females, a drives in females

Parameters

```
params = {
   wAm → 1, wam → 1, wAf → 1, waf → 1,
   αf → 1 / 2 - 8 / 100,
   αm → 1 / 2,
   MAa → 1, FAa → 1,
   FAA → 1 - 4 / 10,
   Faa → 1 - 4 / 10
  };
```

No recombination eigenvalues

```
WAinvA = λmA1 /. reverse /. pAveM → (1 - q) pXm + q pYm /. equilA0 /. params // Simplify;
WainvA = λma1 /. reverse /. pAveM → (1 - q) pXm + q pYm /. equilA0 /. params // Simplify;
WAinvB = λmA1 /. reverse /. pAveM → (1 - q) pXm + q pYm /. equilB0 /. params // Simplify;
WainvB = λma1 /. reverse /. pAveM → (1 - q) pXm + q pYm /. equilB0 /. params // Simplify;
```

Maximum absolute no recombination eigenvalue from the full characteristic polynomial

```
λWsolA =
  Max[Abs[λ /. Solve[0 == charpolyk1 /. r → 0 /. R → 0 /. ρ → 0 /. equilA0 /. params, λ] //
      Simplify]];
λWsolB = Max[Abs[λ /. Solve[0 == charpolyk1 /. r → 0 /. R → 0 /. ρ → 0 /. equilB0 /.
         params, λ] // Simplify]];
```

Plot

Region plots of invasion

```
(*neo-WA invades XY from equilA*)
plotWAinvA =
  RegionPlot[{
     (validcondA /. params) && (*valid*)
      (stabcondA /. Rf → 0 /. Rm → 0 /. equilA0 /. params) && (*internally stable*)
      1 < WAinvA (*invasion*)
    },
    {Maa, 0, 3 / 2}, {MAA, 0, 3 / 2},
    PlotStyle → {Gray, Opacity[0.5]},
    BoundaryStyle → None
  ];

(*neo-Wa invades XY from equilA*)
plotWainvA =
  RegionPlot[{
     (validcondA /. params) &&
      (stabcondA /. Rf → 0 /. Rm → 0 /. equilA0 /. params) &&
      1 < WainvA
    },
    {Maa, 0, 3 / 2}, {MAA, 0, 3 / 2},
    PlotStyle → {Gray, Opacity[0.5]},
    BoundaryStyle → None
  ];
```

```
(*neo-WA invades XY from equilB*)
plotWAinvB =
  RegionPlot[{
     (stabcondB /. Rf → 0 /. Rm → 0 /. equilB0 /. params) && (*internally stable*)
      1 < WAinvB(*invasion*)
    },
    {Maa, 0, 3 / 2}, {MAA, 0, 3 / 2},
    PlotStyle → {Gray, Opacity[0.5]},
    BoundaryStyle → None
  ];

(*neo-Wa invades XY from equilB*)
plotWainvB =
  RegionPlot[{
     (stabcondB /. Rf → 0 /. Rm → 0 /. equilB0 /. params) &&
      1 < WainvB
    },
    {Maa, 0, 3 / 2}, {MAA, 0, 3 / 2},
    PlotStyle → {Gray, Opacity[0.5]},
    BoundaryStyle → None
  ];

(*Ya equilibrium internally stable*)
plotYaStable =
  RegionPlot[{
     (stabcondA /. Rf → 0 /. Rm → 0 /. equilA0 /. params) ||
      (stabcondB /. Rf → 0 /. Rm → 0 /. equilB0 /. params)
    },
    {Maa, 0, 3 / 2}, {MAA, 0, 3 / 2},
    PlotStyle → White,
    BoundaryStyle → {Black, Thick}
  ];

(*Ya equilibrium internally unstable*)
plotYaUnstable =
  RegionPlot[{
     1 < 2
    },
    {Maa, 0, 3 / 2}, {MAA, 0, 3 / 2},
    PlotStyle → None,
    BoundaryStyle → None,
    Mesh → 50,
    MeshFunctions → {#1 - #2 &},
    MeshStyle → Black
  ];
```

```
plotC =
 Show[

  plotYaUnstable,
  plotYaStable,
  plotWAinvA,
  plotWainvA,
  plotWAinvB,
  plotWainvB,

  Graphics[{Thick, Black, Line[{{0.5, 0.9}, {0.4, 0.5}}]}],
  PlotRange → {{0, 1.5}, {0, 1.5}},
  ImageSize → {xsize, xsize},
  PlotRangePadding → 0,
  FrameTicks → {Table[{x, x, ticksize}, {x, 0, 1, 1}],
    Table[{y, y, ticksize}, {y, 0, 1, 1}], None, None},
  FrameTicksStyle → {{Directive[Black, Thickness[lwd]],
     Directive[Black, Thickness[lwd]]}, {Directive[Black, Thickness[lwd]],
     Directive[Black, Thickness[lwd], FontColor → White]}},
  FrameStyle → {{Black, Thickness[lwd]}, {Black, Thickness[lwd]},
    {Black, Thickness[lwd]}, {Black, Thickness[lwd]}},
  (*FrameLabel→{"Relative fitness of aa males",""},*)
  BaseStyle → {FontFamily → "Helvetica", FontSize → 14},
  ImagePadding → Pad,
  Epilog → {
    Text[Style["C", 14, Bold], Scaled@{0.05, 0.95}],
    Text[Style["Λ_{W'A}^(XY) >1
&
Λ_{W'a}^(XY) >1", 14], {0.29, 0.4}, {1, 0}],
    Text[Style["Λ_{W'A}^(XY) <1
&
Λ_{W'a}^(XY) >1", 14], {0.5, 0.9}, {-1, 0}, Background → White],
    Text[Style["Λ_{W'A}^(XY) <1
&
Λ_{W'a}^(XY) <1", 14], {0.82, 0.3}, {1, 0}],
    Text[Style["Λ_{W'A}^(XY) >1
&
Λ_{W'a}^(XY) <1", 14], {1.3, 0.4}, {1, 0}](*,
    Rotate[
     Text[Style["Relative fitness of AA males",14],Scaled@ylabpos],90 Degree]*)
   },
  PlotLabel → Style["overdominance in females", 16, Black, Bold],
  PlotRangeClipping → False
 ]
```

overdominance in females

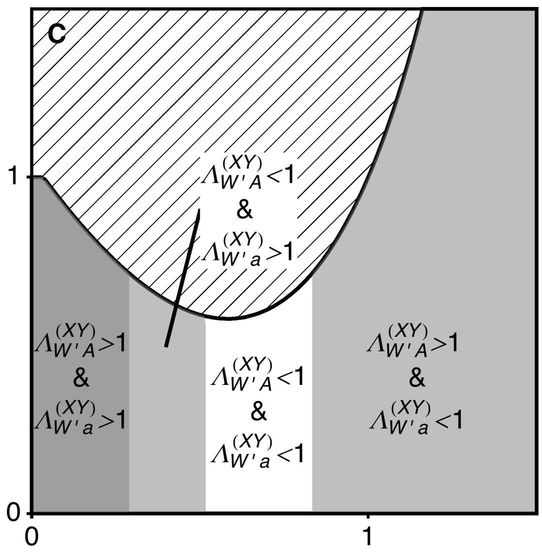

Notice that this is consistent with the solution from the full characteristic polynomial (but here we don’t know which eigenvalue belongs to which haplotype)

```
(*neo-W invades XY from equilA*)
plotWinvA =
  RegionPlot[{
    (validcondA /. params) && (*valid*)
     (stabcondA /. Rf → 0 /. Rm → 0 /. equilA0 /. params) && (*internally stable*)
     1 < λWsolA (*invasion*)
   },
   {Maa, 0, 3 / 2}, {MAA, 0, 3 / 2},
   PlotStyle → {Gray, Opacity[0.5]},
   BoundaryStyle → None
  ];

(*neo-W invades XY from equilB*)
plotWinvB =
  RegionPlot[{
    (stabcondB /. Rf → 0 /. Rm → 0 /. equilB0 /. params) && (*internally stable*)
     1 < λWsolB(*invasion*)
   },
   {Maa, 0, 3 / 2}, {MAA, 0, 3 / 2},
   PlotStyle → {Gray, Opacity[0.5]},
   BoundaryStyle → None
  ];
```

```
Show[

 plotWinvA,
 plotWinvB,
 plotYaStable,

 PlotRange → {{0, 1.5}, {0, 1.5}},
 ImageSize → {xsize, xsize},
 PlotRangePadding → 0,
 FrameTicks → {Table[{x, x, ticksize}, {x, 0, 1, 1}],
   Table[{y, y, ticksize}, {y, 0, 1, 1}], None, None},
 FrameTicksStyle → {{Directive[Black, Thickness[lwd]],
    Directive[Black, Thickness[lwd]]}, {Directive[Black, Thickness[lwd]],
    Directive[Black, Thickness[lwd], FontColor → White]}},
 FrameStyle → {{Black, Thickness[lwd]}, {Black, Thickness[lwd]},
   {Black, Thickness[lwd]}, {Black, Thickness[lwd]}},
 FrameLabel → {"Relative fitness of aa males", ""},
 BaseStyle → {FontFamily → "Helvetica", FontSize → 14},
 ImagePadding → Pad,
 Epilog → {
   Text[Style["Ya equilibrium unstable", 14], {0.5, 1.25}],
   Rotate[
    Text[Style["Relative fitness of AA males", 14], Scaled@ylabpos], 90 Degree]
  },
 PlotLabel → Style["A favoured in females", 16, Black, Bold],
 PlotRangeClipping → False
]
```

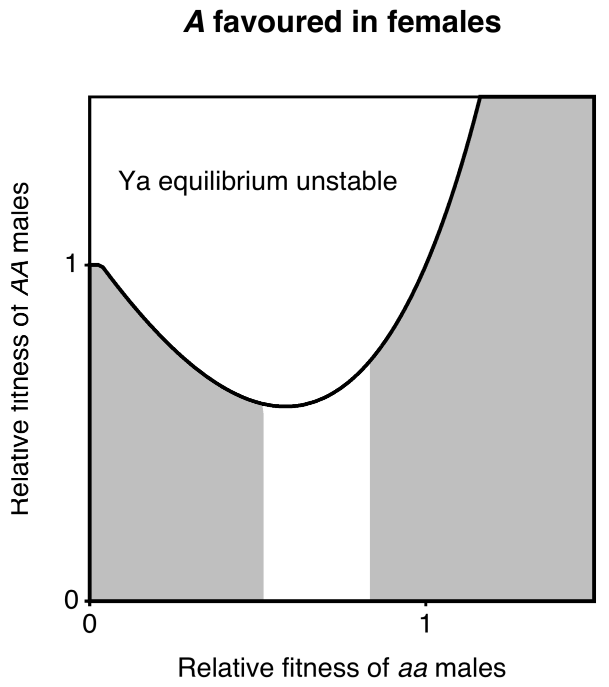

Panel D - A favoured in females, A drives in females

Parameters

```
params = {
    wAm → 1, wam → 1, wAf → 1, waf → 1,
    αf → 1 / 2 + 8 / 100,
    αm → 1 / 2,
    MAa → 1, FAa → 1,
    Faa → 1 - 3 / 20,
    FAA → 1 + 1 / 20
  };
```

No recombination eigenvalues

```
WAinvA = λmA1 /. reverse /. pAveM → (1 - q) pXm + q pYm /. equilA0 /. params // Simplify;
WainvA = λma1 /. reverse /. pAveM → (1 - q) pXm + q pYm /. equilA0 /. params // Simplify;
WAinvB = λmA1 /. reverse /. pAveM → (1 - q) pXm + q pYm /. equilB0 /. params // Simplify;
WainvB = λma1 /. reverse /. pAveM → (1 - q) pXm + q pYm /. equilB0 /. params // Simplify;
```

Maximum absolute no recombination eigenvalue from the full characteristic polynomial

```
λWsolA =
  Max[Abs[λ /. Solve[0 == charpolyk1 /. r → 0 /. R → 0 /. ρ → 0 /. equilA0 /. params, λ] //
      Simplify]];
λWsolB = Max[Abs[λ /. Solve[0 == charpolyk1 /. r → 0 /. R → 0 /. ρ → 0 /. equilB0 /.
         params, λ] // Simplify]];
```

Plot

Region plots of invasion

```
(*neo-WA invades XY from equilA*)
plotWAinvA =
  RegionPlot[{
     (validcondA /. params) && (*valid*)
      (stabcondA /. Rf → 0 /. Rm → 0 /. equilA0 /. params) && (*internally stable*)
      1 < WAinvA (*invasion*)
    },
    {Maa, 0, 3 / 2}, {MAA, 0, 3 / 2},
    PlotStyle → {Gray, Opacity[0.5]},
    BoundaryStyle → None
  ];

(*neo-Wa invades XY from equilA*)
plotWainvA =
  RegionPlot[{
     (validcondA /. params) &&
      (stabcondA /. Rf → 0 /. Rm → 0 /. equilA0 /. params) &&
      1 < WainvA
    },
    {Maa, 0, 3 / 2}, {MAA, 0, 3 / 2},
    PlotStyle → {Gray, Opacity[0.5]},
    BoundaryStyle → None
  ];
```

```
(*neo-WA invades XY from equilB*)
plotWAinvB =
  RegionPlot[{
     (stabcondB /. Rf → 0 /. Rm → 0 /. equilB0 /. params) && (*internally stable*)
      1 < WAinvB(*invasion*)
    },
    {Maa, 0, 3 / 2}, {MAA, 0, 3 / 2},
    PlotStyle → {Gray, Opacity[0.5]},
    BoundaryStyle → None
  ];

(*neo-Wa invades XY from equilB*)
plotWainvB =
  RegionPlot[{
     (stabcondB /. Rf → 0 /. Rm → 0 /. equilB0 /. params) &&
      1 < WainvB
    },
    {Maa, 0, 3 / 2}, {MAA, 0, 3 / 2},
    PlotStyle → {Gray, Opacity[0.5]},
    BoundaryStyle → None
  ];

(*Ya equilibrium internally stable*)
plotYaStable =
  RegionPlot[{
     (stabcondA /. Rf → 0 /. Rm → 0 /. equilA0 /. params) ||
      (stabcondB /. Rf → 0 /. Rm → 0 /. equilB0 /. params)
    },
    {Maa, 0, 3 / 2}, {MAA, 0, 3 / 2},
    PlotStyle → White,
    BoundaryStyle → {Black, Thick}
  ];

(*Ya equilibrium internally unstable*)
plotYaUnstable =
  RegionPlot[{
     1 < 2
    },
    {Maa, 0, 3 / 2}, {MAA, 0, 3 / 2},
    PlotStyle → None,
    BoundaryStyle → None,
    Mesh → 50,
    MeshFunctions → {#1 - #2 &},
    MeshStyle → Black
  ];
```

```
plotD =
 Show[

  plotYaUnstable,
  plotYaStable,
  plotWAinvA,
  plotWainvA,
  plotWAinvB,
  plotWainvB,

  PlotRange → {{0, 1.5}, {0, 1.5}},
  ImageSize → {xsize, xsize},
  PlotRangePadding → 0,
  FrameTicks → {Table[{x, x, ticksize}, {x, 0, 1, 1}],
    Table[{y, y, ticksize}, {y, 0, 1, 1}], None, None},
  FrameTicksStyle → {{Directive[Black, Thickness[lwd]],
     Directive[Black, Thickness[lwd]]}, {Directive[Black, Thickness[lwd]],
     Directive[Black, Thickness[lwd], FontColor → White]}},
  FrameStyle → {{Black, Thickness[lwd]}, {Black, Thickness[lwd]},
    {Black, Thickness[lwd]}, {Black, Thickness[lwd]}},
  FrameLabel → {"Relative fitness of aa males", ""},
  BaseStyle → {FontFamily → "Helvetica", FontSize → 14},
  ImagePadding → Pad,
  Epilog → {
    Text[Style["D", 14, Bold], Scaled@{0.05, 0.95}],
    Text[Style["Λ_{W'A}^{(XY)} >1 & Λ_{W'a}^{(XY)} <1", 14], {0.75, 0.5}],
    Rotate[
     Text[Style["Relative fitness of AA males", 14], Scaled@ylabpos], 90 Degree]
   },
  PlotLabel → Style[(*"A favoured in females"*)"", 16, Black, Bold],
  PlotRangeClipping → False
 ]
```

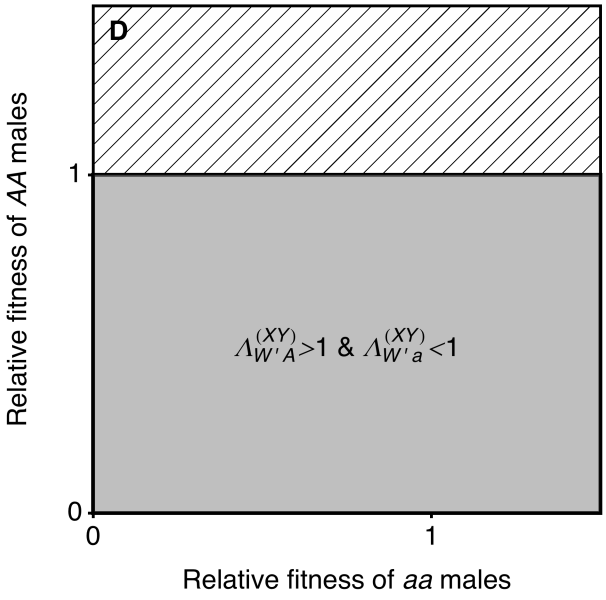

Notice that this is consistent with the solution from the full characteristic polynomial (but here we don’t know which eigenvalue belongs to which haplotype)

```
(*neo-W invades XY from equilA*)
plotWinvA =
  RegionPlot[{
    (validcondA /. params) && (*valid*)
     (stabcondA /. Rf → 0 /. Rm → 0 /. equilA0 /. params) && (*internally stable*)
     1 < λWsolA (*invasion*)
   },
   {Maa, 0, 3 / 2}, {MAA, 0, 3 / 2},
   PlotStyle → {Gray, Opacity[0.5]},
   BoundaryStyle → None
  ];

(*neo-W invades XY from equilB*)
plotWinvB =
  RegionPlot[{
    (stabcondB /. Rf → 0 /. Rm → 0 /. equilB0 /. params) && (*internally stable*)
     1 < λWsolB(*invasion*)
   },
   {Maa, 0, 3 / 2}, {MAA, 0, 3 / 2},
   PlotStyle → {Gray, Opacity[0.5]},
   BoundaryStyle → None
  ];
```

```
Show[

 plotWinvA,
 plotWinvB,
 plotYaStable,

 PlotRange → {{0, 1.5}, {0, 1.5}},
 ImageSize → {xsize, xsize},
 PlotRangePadding → 0,
 FrameTicks → {Table[{x, x, ticksize}, {x, 0, 1, 1}],
   Table[{y, y, ticksize}, {y, 0, 1, 1}], None, None},
 FrameTicksStyle → {{Directive[Black, Thickness[lwd]],
    Directive[Black, Thickness[lwd]]}, {Directive[Black, Thickness[lwd]],
    Directive[Black, Thickness[lwd], FontColor → White]}},
 FrameStyle → {{Black, Thickness[lwd]}, {Black, Thickness[lwd]},
   {Black, Thickness[lwd]}, {Black, Thickness[lwd]}},
 FrameLabel → {"Relative fitness of aa males", ""},
 BaseStyle → {FontFamily → "Helvetica", FontSize → 14},
 ImagePadding → Pad,
 Epilog → {
   Text[Style["Ya equilibrium unstable", 14], {0.5, 1.25}],
   Rotate[
    Text[Style["Relative fitness of AA males", 14], Scaled@ylabpos], 90 Degree]
  },
 PlotLabel → Style["A favoured in females", 16, Black, Bold],
 PlotRangeClipping → False
]
```

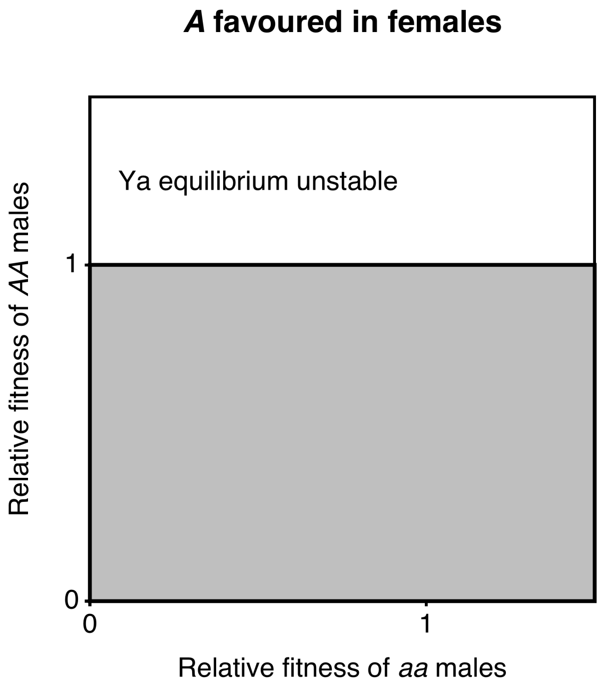

Panel E - a favoured in females, A drives in females

Parameters

```
params = {
    wAm → 1, wam → 1, wAf → 1, waf → 1,
    αf → 1 / 2 + 8 / 100,
    αm → 1 / 2,
    MAa → 1, FAa → 1,
    FAA → 1 - 3 / 20,
    Faa → 1 + 1 / 20
   };
```

No recombination eigenvalues

```
WAinvA = λmA1 /. reverse /. pAveM → (1 - q) pXm + q pYm /. equilA0 /. params // Simplify;
WainvA = λma1 /. reverse /. pAveM → (1 - q) pXm + q pYm /. equilA0 /. params // Simplify;
WAinvB = λmA1 /. reverse /. pAveM → (1 - q) pXm + q pYm /. equilB0 /. params // Simplify;
WainvB = λma1 /. reverse /. pAveM → (1 - q) pXm + q pYm /. equilB0 /. params // Simplify;
```

Maximum absolute no recombination eigenvalue from the full characteristic polynomial

```
λWsolA =
  Max[Abs[λ /. Solve[0 == charpolyk1 /. r → 0 /. R → 0 /. ρ → 0 /. equilA0 /. params, λ] //
      Simplify]];
λWsolB = Max[Abs[λ /. Solve[0 == charpolyk1 /. r → 0 /. R → 0 /. ρ → 0 /. equilB0 /.
         params, λ] // Simplify]];
```

Plot

Region plots of invasion

```
(*neo-WA invades XY from equilA*)
plotWAinvA =
  RegionPlot[{
     (validcondA /. params) && (*valid*)
      (stabcondA /. Rf → 0 /. Rm → 0 /. equilA0 /. params) && (*internally stable*)
      1 < WAinvA (*invasion*)
    },
    {Maa, 0, 3 / 2}, {MAA, 0, 3 / 2},
    PlotStyle → {Gray, Opacity[0.5]},
    BoundaryStyle → None
  ];

(*neo-Wa invades XY from equilA*)
plotWainvA =
  RegionPlot[{
     (validcondA /. params) &&
      (stabcondA /. Rf → 0 /. Rm → 0 /. equilA0 /. params) &&
      1 < WainvA
    },
    {Maa, 0, 3 / 2}, {MAA, 0, 3 / 2},
    PlotStyle → {Gray, Opacity[0.5]},
    BoundaryStyle → None
  ];
```

```
(*neo-WA invades XY from equilB*)
plotWAinvB =
  RegionPlot[{
    (stabcondB /. Rf → 0 /. Rm → 0 /. equilB0 /. params) && (*internally stable*)
     1 < WAinvB(*invasion*)
   },
   {Maa, 0, 3 / 2}, {MAA, 0, 3 / 2},
   PlotStyle → {Gray, Opacity[0.5]},
   BoundaryStyle → None
  ];

(*neo-Wa invades XY from equilB*)
plotWainvB =
  RegionPlot[{
    (stabcondB /. Rf → 0 /. Rm → 0 /. equilB0 /. params) &&
     1 < WainvB
   },
   {Maa, 0, 3 / 2}, {MAA, 0, 3 / 2},
   PlotStyle → {Gray, Opacity[0.5]},
   BoundaryStyle → None
  ];

(*Ya equilibrium internally stable*)
plotYaStable =
  RegionPlot[{
    (stabcondA /. Rf → 0 /. Rm → 0 /. equilA0 /. params) ||
     (stabcondB /. Rf → 0 /. Rm → 0 /. equilB0 /. params)
   },
   {Maa, 0, 3 / 2}, {MAA, 0, 3 / 2},
   PlotStyle → White,
   BoundaryStyle → {Black, Thick}
  ];

(*Ya equilibrium internally unstable*)
plotYaUnstable =
  RegionPlot[{
    1 < 2
   },
   {Maa, 0, 3 / 2}, {MAA, 0, 3 / 2},
   PlotStyle → None,
   BoundaryStyle → None,
   Mesh → 50,
   MeshFunctions → {#1 - #2 &},
   MeshStyle → Black
  ];
```

```
plotE =
 Show[

  plotYaUnstable,
  plotYaStable,
  plotWAinvA,
  plotWainvA,
  plotWAinvB,
  plotWainvB,

  PlotRange → {{0, 1.5}, {0, 1.5}},
  ImageSize → {xsize, xsize},
  PlotRangePadding → 0,
  FrameTicks → {Table[{x, x, ticksize}, {x, 0, 1, 1}],
    Table[{y, y, ticksize}, {y, 0, 1, 1}], None, None},
  FrameTicksStyle → {{Directive[Black, Thickness[lwd]],
     Directive[Black, Thickness[lwd]]}, {Directive[Black, Thickness[lwd]],
     Directive[Black, Thickness[lwd], FontColor → White]}},
  FrameStyle → {{Black, Thickness[lwd]}, {Black, Thickness[lwd]},
    {Black, Thickness[lwd]}, {Black, Thickness[lwd]}},
  FrameLabel → {"Relative fitness of aa males", ""},
  BaseStyle → {FontFamily → "Helvetica", FontSize → 14},
  ImagePadding → Pad,
  Epilog → {
    Text[Style["E", 14, Bold], Scaled@{0.05, 0.95}],
    Text[Style["Λ_{W'A}^{(XY)}<1 & Λ_{W'a}^{(XY)}>1", 14], {0.6, 0.5}](*,
    Rotate[
     Text[Style["Relative fitness of AA males",14],Scaled@ylabpos],90 Degree]*)
   },
  PlotLabel → Style[(*"a favoured in females"*)"", 16, Black, Bold],
  PlotRangeClipping → False
 ]
```

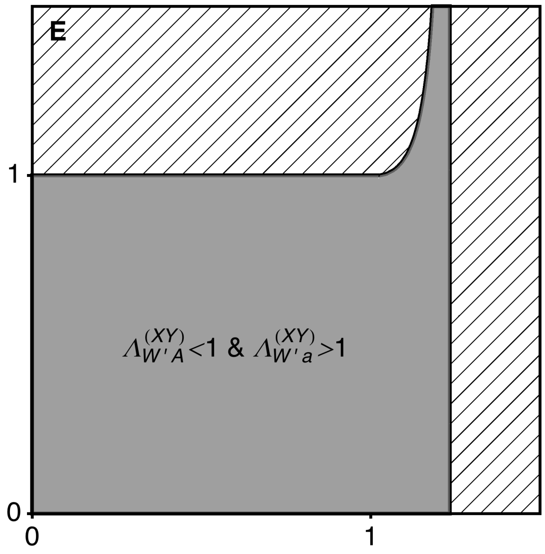

Relative fitness of *aa* males

Notice that this is consistent with the solution from the full characteristic polynomial (but here we don't know which eigenvalue belongs to which haplotype)

```
(*neo-W invades XY from equilA*)
plotWinvA =
  RegionPlot[{
     (validcondA /. params) && (*valid*)
      (stabcondA /. Rf → 0 /. Rm → 0 /. equilA0 /. params) && (*internally stable*)
      1 < λWsolA (*invasion*)
    },
    {Maa, 0, 3 / 2}, {MAA, 0, 3 / 2},
    PlotStyle → {Gray, Opacity[0.5]},
    BoundaryStyle → None
  ];

(*neo-W invades XY from equilB*)
plotWinvB =
  RegionPlot[{
     (stabcondB /. Rf → 0 /. Rm → 0 /. equilB0 /. params) && (*internally stable*)
      1 < λWsolB(*invasion*)
    },
    {Maa, 0, 3 / 2}, {MAA, 0, 3 / 2},
    PlotStyle → {Gray, Opacity[0.5]},
    BoundaryStyle → None
  ];
```

```
Show[

 plotWinvA,
 plotWinvB,
 plotYaStable,

 PlotRange → {{0, 1.5}, {0, 1.5}},
 ImageSize → {xsize, xsize},
 PlotRangePadding → 0,
 FrameTicks → {Table[{x, x, ticksize}, {x, 0, 1, 1}],
   Table[{y, y, ticksize}, {y, 0, 1, 1}], None, None},
 FrameTicksStyle → {{Directive[Black, Thickness[lwd]],
    Directive[Black, Thickness[lwd]]}, {Directive[Black, Thickness[lwd]],
    Directive[Black, Thickness[lwd], FontColor → White]}},
 FrameStyle → {{Black, Thickness[lwd]}, {Black, Thickness[lwd]},
   {Black, Thickness[lwd]}, {Black, Thickness[lwd]}},
 FrameLabel → {"Relative fitness of aa males", ""},
 BaseStyle → {FontFamily → "Helvetica", FontSize → 14},
 ImagePadding → Pad,
 Epilog → {
   Text[Style["Ya equilibrium unstable", 14], {0.5, 1.25}],
   Rotate[
    Text[Style["Relative fitness of AA males", 14], Scaled@ylabpos], 90 Degree]
  },
 PlotLabel → Style["a favoured in females", 16, Black, Bold],
 PlotRangeClipping → False
]
```

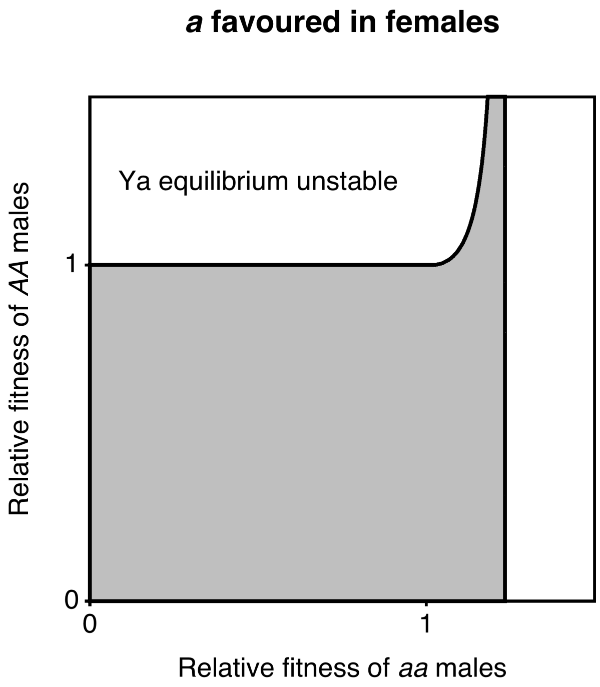

Panel F - overdominance in females, A drives in females

Parameters

```
params = {
    wAm → 1, wam → 1, wAf → 1, waf → 1,
    αf → 1 / 2 + 8 / 100,
    αm → 1 / 2,
    MAa → 1, FAa → 1,
    FAA → 1 - 4 / 10,
    Faa → 1 - 4 / 10
  };
```

No recombination eigenvalues

```
WAinvA = λmA1 /. reverse /. pAveM → (1 - q) pXm + q pYm /. equilA0 /. params // Simplify;
WainvA = λma1 /. reverse /. pAveM → (1 - q) pXm + q pYm /. equilA0 /. params // Simplify;
WAinvB = λmA1 /. reverse /. pAveM → (1 - q) pXm + q pYm /. equilB0 /. params // Simplify;
WainvB = λma1 /. reverse /. pAveM → (1 - q) pXm + q pYm /. equilB0 /. params // Simplify;
```

Maximum absolute no recombination eigenvalue from the full characteristic polynomial

```
λWsolA =
  Max[Abs[λ /. Solve[0 == charpolyk1 /. r → 0 /. R → 0 /. ρ → 0 /. equilA0 /. params, λ] //
      Simplify]];
λWsolB = Max[Abs[λ /. Solve[0 == charpolyk1 /. r → 0 /. R → 0 /. ρ → 0 /. equilB0 /.
          params, λ] // Simplify]];
```

Plot

Region plots of invasion

```
(*neo-WA invades XY from equilA*)
plotWAinvA =
  RegionPlot[{
     (validcondA /. params) && (*valid*)
      (stabcondA /. Rf → 0 /. Rm → 0 /. equilA0 /. params) && (*internally stable*)
      1 < WAinvA (*invasion*)
    },
    {Maa, 0, 3 / 2}, {MAA, 0, 3 / 2},
    PlotStyle → {Gray, Opacity[0.5]},
    BoundaryStyle → None
  ];

(*neo-Wa invades XY from equilA*)
plotWainvA =
  RegionPlot[{
     (validcondA /. params) &&
      (stabcondA /. Rf → 0 /. Rm → 0 /. equilA0 /. params) &&
      1 < WainvA
    },
    {Maa, 0, 3 / 2}, {MAA, 0, 3 / 2},
    PlotStyle → {Gray, Opacity[0.5]},
    BoundaryStyle → None
  ];
```

```
(*neo-WA invades XY from equilB*)
plotWAinvB =
  RegionPlot[{
     (stabcondB /. Rf → 0 /. Rm → 0 /. equilB0 /. params) && (*internally stable*)
      1 < WAinvB(*invasion*)
    },
    {Maa, 0, 3 / 2}, {MAA, 0, 3 / 2},
    PlotStyle → {Gray, Opacity[0.5]},
    BoundaryStyle → None
   ];

(*neo-Wa invades XY from equilB*)
plotWainvB =
  RegionPlot[{
     (stabcondB /. Rf → 0 /. Rm → 0 /. equilB0 /. params) &&
      1 < WainvB
    },
    {Maa, 0, 3 / 2}, {MAA, 0, 3 / 2},
    PlotStyle → {Gray, Opacity[0.5]},
    BoundaryStyle → None
   ];

(*Ya equilibrium internally stable*)
plotYaStable =
  RegionPlot[{
     (stabcondA /. Rf → 0 /. Rm → 0 /. equilA0 /. params) ||
      (stabcondB /. Rf → 0 /. Rm → 0 /. equilB0 /. params)
    },
    {Maa, 0, 3 / 2}, {MAA, 0, 3 / 2},
    PlotStyle → White,
    BoundaryStyle → {Black, Thick}
   ];

(*Ya equilibrium internally unstable*)
plotYaUnstable =
  RegionPlot[{
     1 < 2
    },
    {Maa, 0, 3 / 2}, {MAA, 0, 3 / 2},
    PlotStyle → None,
    BoundaryStyle → None,
    Mesh → 50,
    MeshFunctions → {#1 - #2 &},
    MeshStyle → Black
   ];
```

```
plotF =
 Show[

  plotYaUnstable,
  plotYaStable,
  plotWAinvA,
  plotWainvA,
  plotWAinvB,
  plotWainvB,

  PlotRange → {{0, 1.5}, {0, 1.5}},
  ImageSize → {xsize, xsize},
  PlotRangePadding → 0,
  FrameTicks → {Table[{x, x, ticksize}, {x, 0, 1, 1}],
    Table[{y, y, ticksize}, {y, 0, 1, 1}], None, None},
  FrameTicksStyle → {{Directive[Black, Thickness[lwd]],
     Directive[Black, Thickness[lwd]]}, {Directive[Black, Thickness[lwd]],
     Directive[Black, Thickness[lwd], FontColor → White]}},
  FrameStyle → {{Black, Thickness[lwd]}, {Black, Thickness[lwd]},
    {Black, Thickness[lwd]}, {Black, Thickness[lwd]}},
  FrameLabel → {"Relative fitness of aa males", ""},
  BaseStyle → {FontFamily → "Helvetica", FontSize → 14},
  ImagePadding → Pad,
  Epilog → {
    Text[Style["F", 14, Bold], Scaled@{0.05, 0.95}],
    Text[Style["Λ_{W'A}^{(XY)} >1
&
Λ_{W'a}^{(XY)} >1", 14], {0.35, 0.5}],
    Text[Style["Λ_{W'A}^{(XY)} >1 & Λ_{W'a}^{(XY)} <1", 14], {1.1, 0.5}](*,
    Rotate[
     Text[Style["Relative fitness of AA males",14],Scaled@ylabpos],90 Degree]*)
   },
  PlotLabel → Style[(*"overdominance in females"*)"", 16, Black, Bold],
  PlotRangeClipping → False
 ]
```

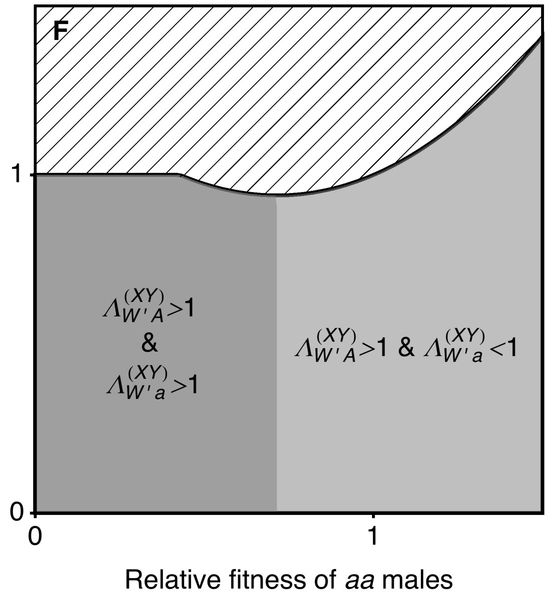

Notice that this is consistent with the solution from the full characteristic polynomial (but here we don't know which eigenvalue belongs to which haplotype)

```
(*neo-W invades XY from equilA*)
plotWinvA =
  RegionPlot[{
    (validcondA /. params) && (*valid*)
     (stabcondA /. Rf → 0 /. Rm → 0 /. equilA0 /. params) && (*internally stable*)
     1 < λWsolA (*invasion*)
   },
   {Maa, 0, 3 / 2}, {MAA, 0, 3 / 2},
   PlotStyle → {Gray, Opacity[0.5]},
   BoundaryStyle → None
  ];

(*neo-W invades XY from equilB*)
plotWinvB =
  RegionPlot[{
    (stabcondB /. Rf → 0 /. Rm → 0 /. equilB0 /. params) && (*internally stable*)
     1 < λWsolB(*invasion*)
   },
   {Maa, 0, 3 / 2}, {MAA, 0, 3 / 2},
   PlotStyle → {Gray, Opacity[0.5]},
   BoundaryStyle → None
  ];
```

```
Show[

 plotWinvA,
 plotWinvB,
 plotYaStable,

 PlotRange → {{0, 1.5}, {0, 1.5}},
 ImageSize → {xsize, xsize},
 PlotRangePadding → 0,
 FrameTicks → {Table[{x, x, ticksize}, {x, 0, 1, 1}],
   Table[{y, y, ticksize}, {y, 0, 1, 1}], None, None},
 FrameTicksStyle → {{Directive[Black, Thickness[lwd]],
    Directive[Black, Thickness[lwd]]}, {Directive[Black, Thickness[lwd]],
    Directive[Black, Thickness[lwd], FontColor → White]}},
 FrameStyle → {{Black, Thickness[lwd]}, {Black, Thickness[lwd]},
   {Black, Thickness[lwd]}, {Black, Thickness[lwd]}},
 FrameLabel → {"Relative fitness of aa males", ""},
 BaseStyle → {FontFamily → "Helvetica", FontSize → 14},
 ImagePadding → Pad,
 Epilog → {
   Text[Style["Ya equilibrium unstable", 14], {0.5, 1.25}],
   Rotate[
    Text[Style["Relative fitness of AA males", 14], Scaled@ylabpos], 90 Degree]
  },
 PlotLabel → Style["overdominance in females", 16, Black, Bold],
 PlotRangeClipping → False
]
```

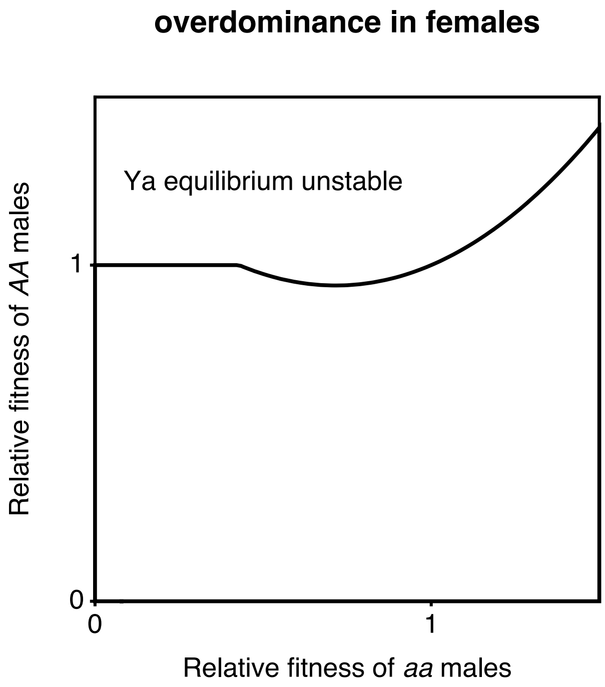

All Panels

```
GraphicsGrid[{
   {plotA, plotB, plotC},
   {plotD, plotE, plotF}
  },
  Spacings → -50
]

Export[plotdir <> "Region_plot_combined_FemaleDrive.eps", % // rasterTrick];
```

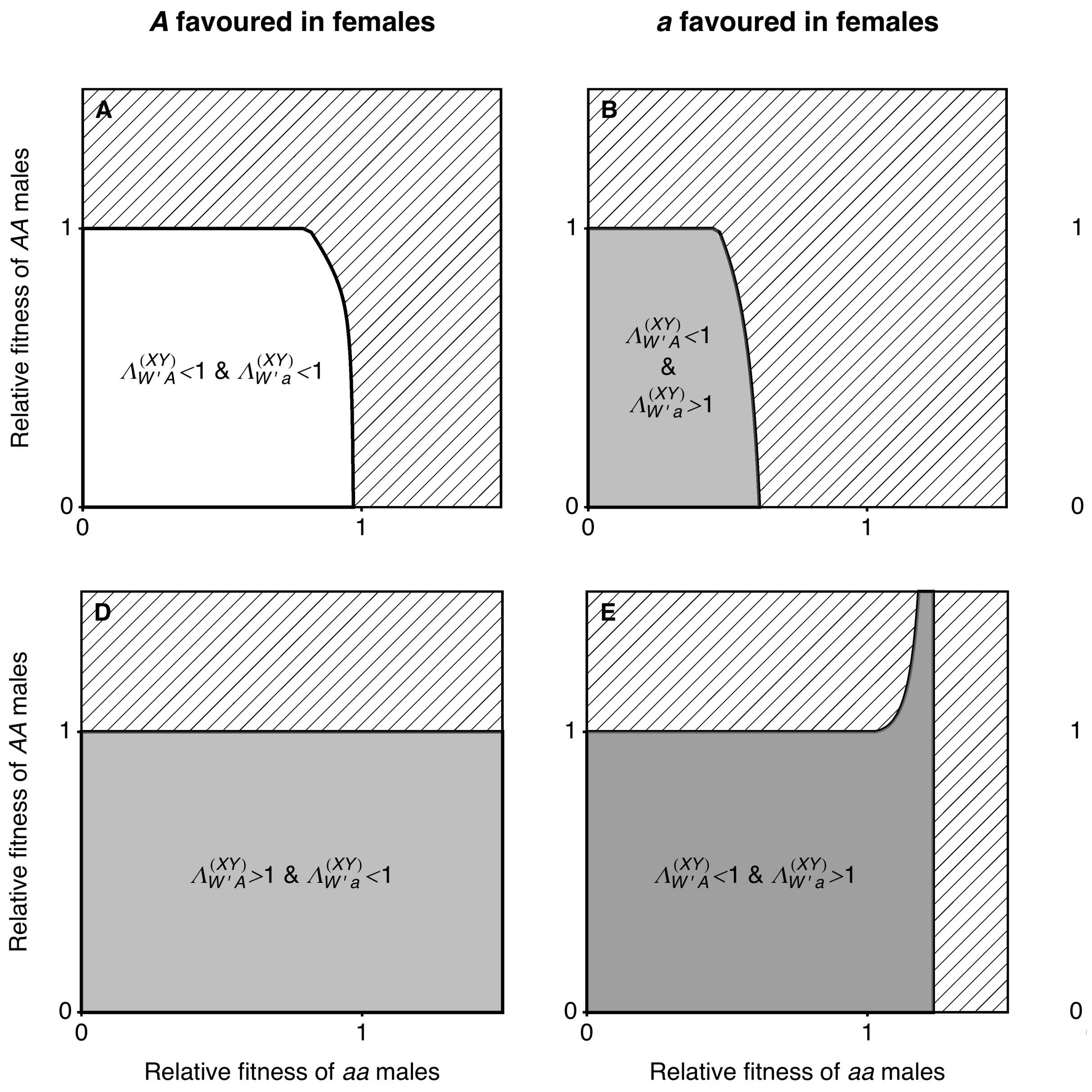

Figure S.7 - neo-W haplotype invasion with haploid competition in females

Panel A - A favoured in females, haploid competition favours a in females

Parameters

```
params = {
    wAm → 1, wam → 1, wAf → 1, waf → 1 + 8 / 50,
    αm → 1 / 2, αf → 1 / 2,
    MAa → 1, FAa → 1,
    Faa → 1 - 3 / 20,
    FAA → 1 + 1 / 20
  };
```

Making the sex-ratio

```
freqMale /. equilB0 /. params // N
```

```
0.5
```

No recombination eigenvalues

```
WAinvA = λmA1 /. reverse /. pAveM → (1 - q) pXm + q pYm /. equilA0 /. params // Simplify;
WainvA = λma1 /. reverse /. pAveM → (1 - q) pXm + q pYm /. equilA0 /. params // Simplify;
WAinvB = λmA1 /. reverse /. pAveM → (1 - q) pXm + q pYm /. equilB0 /. params // Simplify;
WainvB = λma1 /. reverse /. pAveM → (1 - q) pXm + q pYm /. equilB0 /. params // Simplify;
```

Maximum absolute no recombination eigenvalue from the full characteristic polynomial

```
λWsolA =
  Max[Abs[λ /. Solve[0 == charpolyk1 /. r → 0 /. R → 0 /. ρ → 0 /. equilA0 /. params, λ] //
      Simplify]];
λWsolB = Max[Abs[λ /. Solve[0 == charpolyk1 /. r → 0 /. R → 0 /. ρ → 0 /. equilB0 /.
         params, λ] // Simplify]];
```

Plot

Region plots of invasion

```
(*neo-WA invades XY from equilA*)
plotWAinvA =
  RegionPlot[{
     (validcondA /. params) && (*valid*)
      (stabcondA /. Rf → 0 /. Rm → 0 /. equilA0 /. params) && (*internally stable*)
      1 < WAinvA (*invasion*)
    },
    {Maa, 0, 3 / 2}, {MAA, 0, 3 / 2},
    PlotStyle → {Gray, Opacity[0.5]},
    BoundaryStyle → None
  ];
```

```
(*neo-Wa invades XY from equilA*)
plotWainvA =
  RegionPlot[{
    (validcondA /. params) &&
     (stabcondA /. Rf → 0 /. Rm → 0 /. equilA0 /. params) &&
     1 < WainvA
   },
   {Maa, 0, 3 / 2}, {MAA, 0, 3 / 2},
   PlotStyle → {Gray, Opacity[0.5]},
   BoundaryStyle → None
  ];

(*neo-WA invades XY from equilB*)
plotWAinvB =
  RegionPlot[{
    (stabcondB /. Rf → 0 /. Rm → 0 /. equilB0 /. params) && (*internally stable*)
     1 < WAinvB(*invasion*)
   },
   {Maa, 0, 3 / 2}, {MAA, 0, 3 / 2},
   PlotStyle → {Gray, Opacity[0.5]},
   BoundaryStyle → None
  ];

(*neo-Wa invades XY from equilB*)
plotWainvB =
  RegionPlot[{
    (stabcondB /. Rf → 0 /. Rm → 0 /. equilB0 /. params) &&
     1 < WainvB
   },
   {Maa, 0, 3 / 2}, {MAA, 0, 3 / 2},
   PlotStyle → {Gray, Opacity[0.5]},
   BoundaryStyle → None
  ];

(*Ya equilibrium internally stable*)
plotYaStable =
  RegionPlot[{
    (stabcondA /. Rf → 0 /. Rm → 0 /. equilA0 /. params) ||
     (stabcondB /. Rf → 0 /. Rm → 0 /. equilB0 /. params)
   },
   {Maa, 0, 3 / 2}, {MAA, 0, 3 / 2},
   PlotStyle → White,
   BoundaryStyle → {Black, Thick}
  ];

(*Ya equilibrium internally unstable*)
plotYaUnstable =
  RegionPlot[{
    1 < 2
   },
   {Maa, 0, 3 / 2}, {MAA, 0, 3 / 2},
   PlotStyle → None,
   BoundaryStyle → None,
   Mesh → 50,
   MeshFunctions → {#1 - #2 &},
```

```
    MeshStyle → Black
   ];

plotA =
  Show[

   plotYaUnstable,
   plotYaStable,
   plotWAinvA,
   plotWainvA,
   plotWAinvB,
   plotWainvB,

   Graphics[{Thick, Black, Line[{{1.15, 0.5}, {1.025, 0.25}}]}],
   Graphics[{Thick, Black, Line[{{0.9, 0.8}, {0.7, 1.1}}]}],
   PlotRange → {{0, 1.5}, {0, 1.5}},
   ImageSize → {xsize, xsize},
   PlotRangePadding → 0,
   FrameTicks → {Table[{x, x, ticksize}, {x, 0, 1, 1}],
     Table[{y, y, ticksize}, {y, 0, 1, 1}], None, None},
   FrameTicksStyle → {{Directive[Black, Thickness[lwd]],
      Directive[Black, Thickness[lwd]]}, {Directive[Black, Thickness[lwd]],
      Directive[Black, Thickness[lwd], FontColor → White]}},
   FrameStyle → {{Black, Thickness[lwd]}, {Black, Thickness[lwd]},
     {Black, Thickness[lwd]}, {Black, Thickness[lwd]}},
   (*FrameLabel→{"Relative fitness of aa males",""},*)
   BaseStyle → {FontFamily → "Helvetica", FontSize → 14},
   ImagePadding → Pad,
   Epilog → {
     Text[Style["A", 14, Bold], Scaled@{0.05, 0.95}],
     Text[Style["Λ_{W'A}^(XY) <1 & Λ_{W'a}^(XY) >1", 14], {0.4, 0.5}],
     Text[Style["Λ_{W'A}^(XY) >1
  &
  Λ_{W'a}^(XY) <1", 14], {1.3, 0.7}, Background → White],
     Text[Style["Λ_{W'A}^(XY) <1 & Λ_{W'a}^(XY) <1", 14], {0.6, 1.15}, Background → White],
     Rotate[
      Text[Style["Relative fitness of AA males", 14], Scaled@ylabpos], 90 Degree]
    },
   PlotLabel → Style["A favoured in females", 16, Black, Bold],
   PlotRangeClipping → False
  ]
```

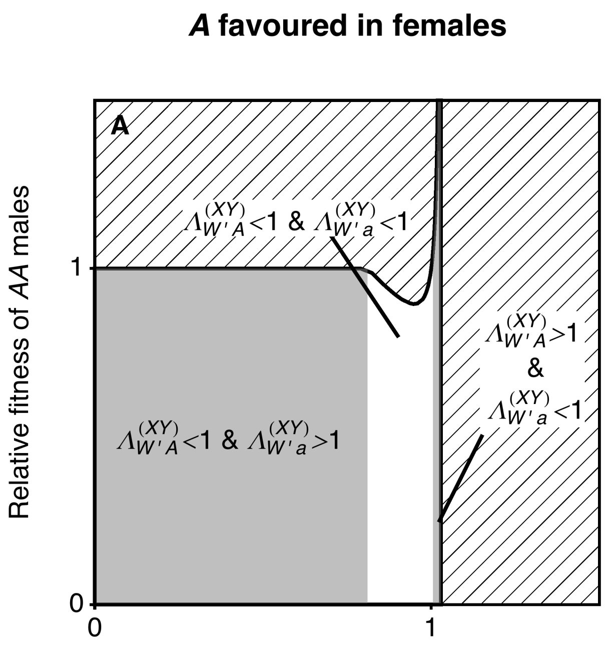

Notice that this is consistent with the solution from the full characteristic polynomial (but here we don't know which eigenvalue belongs to which haplotype)

```
(*neo-W invades XY from equilA*)
plotWinvA =
  RegionPlot[{
    (validcondA /. params) && (*valid*)
     (stabcondA /. Rf → 0 /. Rm → 0 /. equilA0 /. params) && (*internally stable*)
     1 < λWsolA (*invasion*)
   },
   {Maa, 0, 3 / 2}, {MAA, 0, 3 / 2},
   PlotStyle → {Gray, Opacity[0.5]},
   BoundaryStyle → None
  ];

(*neo-W invades XY from equilB*)
plotWinvB =
  RegionPlot[{
    (stabcondB /. Rf → 0 /. Rm → 0 /. equilB0 /. params) && (*internally stable*)
     1 < λWsolB(*invasion*)
   },
   {Maa, 0, 3 / 2}, {MAA, 0, 3 / 2},
   PlotStyle → {Gray, Opacity[0.5]},
   BoundaryStyle → None
  ];
```

```
Show[

 plotWinvA,
 plotWinvB,
 plotYaStable,

 PlotRange → {{0, 1.5}, {0, 1.5}},
 ImageSize → {xsize, xsize},
 PlotRangePadding → 0,
 FrameTicks → {Table[{x, x, ticksize}, {x, 0, 1, 1}],
   Table[{y, y, ticksize}, {y, 0, 1, 1}], None, None},
 FrameTicksStyle → {{Directive[Black, Thickness[lwd]],
    Directive[Black, Thickness[lwd]]}, {Directive[Black, Thickness[lwd]],
    Directive[Black, Thickness[lwd], FontColor → White]}},
 FrameStyle → {{Black, Thickness[lwd]}, {Black, Thickness[lwd]},
   {Black, Thickness[lwd]}, {Black, Thickness[lwd]}},
 FrameLabel → {"Relative fitness of aa males", ""},
 BaseStyle → {FontFamily → "Helvetica", FontSize → 14},
 ImagePadding → Pad,
 Epilog → {
   Text[Style["Ya equilibrium unstable", 14], {0.5, 1.25}],
   Rotate[
    Text[Style["Relative fitness of AA males", 14], Scaled@ylabpos], 90 Degree]
  },
 PlotLabel → Style["A favoured in females", 16, Black, Bold],
 PlotRangeClipping → False
]
```

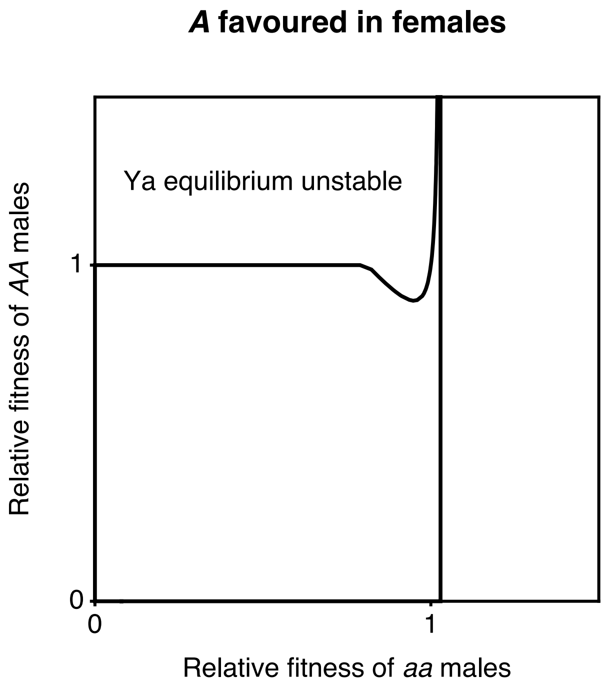

Panel B - a favoured in females, haploid competition favours a in females

Parameters

```
params = {
    wAm → 1, wam → 1, wAf → 1, waf → 1 + 8 / 50,
    αm → 1 / 2, αf → 1 / 2,
    MAa → 1, FAa → 1,
    FAA → 1 - 3 / 20,
    Faa → 1 + 1 / 20
  };
```

No recombination eigenvalues

```
WAinvA = λmA1 /. reverse /. pAveM → (1 - q) pXm + q pYm /. equilA0 /. params // Simplify;
WainvA = λma1 /. reverse /. pAveM → (1 - q) pXm + q pYm /. equilA0 /. params // Simplify;
WAinvB = λmA1 /. reverse /. pAveM → (1 - q) pXm + q pYm /. equilB0 /. params // Simplify;
WainvB = λma1 /. reverse /. pAveM → (1 - q) pXm + q pYm /. equilB0 /. params // Simplify;
```

Maximum absolute no recombination eigenvalue from the full characteristic polynomial

```
λWsolA =
  Max[Abs[λ /. Solve[0 == charpolyk1 /. r → 0 /. R → 0 /. ρ → 0 /. equilA0 /. params, λ] //
      Simplify]];
λWsolB = Max[Abs[λ /. Solve[0 == charpolyk1 /. r → 0 /. R → 0 /. ρ → 0 /. equilB0 /.
        params, λ] // Simplify]];
```

Plot

Region plots of invasion

```
(*neo-WA invades XY from equilA*)
plotWAinvA =
  RegionPlot[{
     (validcondA /. params) && (*valid*)
      (stabcondA /. Rf → 0 /. Rm → 0 /. equilA0 /. params) && (*internally stable*)
      1 < WAinvA (*invasion*)
    },
    {Maa, 0, 3 / 2}, {MAA, 0, 3 / 2},
    PlotStyle → {Gray, Opacity[0.5]},
    BoundaryStyle → None
  ];

(*neo-Wa invades XY from equilA*)
plotWainvA =
  RegionPlot[{
     (validcondA /. params) &&
      (stabcondA /. Rf → 0 /. Rm → 0 /. equilA0 /. params) &&
      1 < WainvA
    },
    {Maa, 0, 3 / 2}, {MAA, 0, 3 / 2},
    PlotStyle → {Gray, Opacity[0.5]},
    BoundaryStyle → None
  ];
```

```
(*neo-WA invades XY from equilB*)
plotWAinvB =
  RegionPlot[{
    (stabcondB /. Rf → 0 /. Rm → 0 /. equilB0 /. params) && (*internally stable*)
     1 < WAinvB(*invasion*)
   },
   {Maa, 0, 3 / 2}, {MAA, 0, 3 / 2},
   PlotStyle → {Gray, Opacity[0.5]},
   BoundaryStyle → None
  ];

(*neo-Wa invades XY from equilB*)
plotWainvB =
  RegionPlot[{
    (stabcondB /. Rf → 0 /. Rm → 0 /. equilB0 /. params) &&
     1 < WainvB
   },
   {Maa, 0, 3 / 2}, {MAA, 0, 3 / 2},
   PlotStyle → {Gray, Opacity[0.5]},
   BoundaryStyle → None
  ];

(*Ya equilibrium internally stable*)
plotYaStable =
  RegionPlot[{
    (stabcondA /. Rf → 0 /. Rm → 0 /. equilA0 /. params) ||
     (stabcondB /. Rf → 0 /. Rm → 0 /. equilB0 /. params)
   },
   {Maa, 0, 3 / 2}, {MAA, 0, 3 / 2},
   PlotStyle → White,
   BoundaryStyle → {Black, Thick}
  ];

(*Ya equilibrium internally unstable*)
plotYaUnstable =
  RegionPlot[{
    1 < 2
   },
   {Maa, 0, 3 / 2}, {MAA, 0, 3 / 2},
   PlotStyle → None,
   BoundaryStyle → None,
   Mesh → 50,
   MeshFunctions → {#1 - #2 &},
   MeshStyle → Black
  ];
```

```
plotB =
 Show[

  plotYaUnstable,
  plotYaStable,
  plotWAinvA,
  plotWainvA,
  plotWAinvB,
  plotWainvB,

  Graphics[{Thick, Black, Line[{{0.75, 0.5}, {0.55, 0.25}}]}],
  PlotRange → {{0, 1.5}, {0, 1.5}},
  ImageSize → {xsize, xsize},
  PlotRangePadding → 0,
  FrameTicks → {Table[{x, x, ticksize}, {x, 0, 1, 1}],
    Table[{y, y, ticksize}, {y, 0, 1, 1}], None, None},
  FrameTicksStyle → {{Directive[Black, Thickness[lwd]],
     Directive[Black, Thickness[lwd]]}, {Directive[Black, Thickness[lwd]],
     Directive[Black, Thickness[lwd], FontColor → White]}},
  FrameStyle → {{Black, Thickness[lwd]}, {Black, Thickness[lwd]},
    {Black, Thickness[lwd]}, {Black, Thickness[lwd]}},
  (*FrameLabel→{"Relative fitness of aa males",""},*)
  BaseStyle → {FontFamily → "Helvetica", FontSize → 14},
  ImagePadding → Pad,
  Epilog → {
    Text[Style["B", 14, Bold], Scaled@{0.05, 0.95}],
    Text[Style["Λ_{W'A}^(XY) >1
&
Λ_{W'a}^(XY) >1", 14], {0.3, 0.5}],
    Text[Style["Λ_{W'A}^(XY) <1
&
Λ_{W'a}^(XY) >1", 14], {0.75, 0.55}, {-1.1, 0.05}, Background → White](*,
    Rotate[
     Text[Style["Relative fitness of AA males",14],Scaled@ylabpos],90 Degree]*)
   },
  PlotLabel → Style["a favoured in females", 16, Black, Bold],
  PlotRangeClipping → False
 ]
```

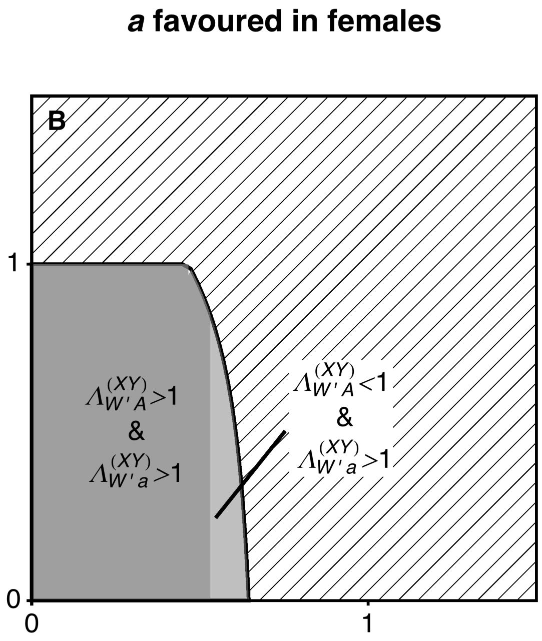

Notice that this is consistent with the solution from the full characteristic polynomial (but here we don't know which eigenvalue belongs to which haplotype)

```
(*neo-W invades XY from equilA*)
plotWinvA =
  RegionPlot[{
     (validcondA /. params) && (*valid*)
      (stabcondA /. Rf → 0 /. Rm → 0 /. equilA0 /. params) && (*internally stable*)
      1 < λWsolA (*invasion*)
    },
    {Maa, 0, 3 / 2}, {MAA, 0, 3 / 2},
    PlotStyle → {Gray, Opacity[0.5]},
    BoundaryStyle → None
  ];

(*neo-W invades XY from equilB*)
plotWinvB =
  RegionPlot[{
     (stabcondB /. Rf → 0 /. Rm → 0 /. equilB0 /. params) && (*internally stable*)
      1 < λWsolB(*invasion*)
    },
    {Maa, 0, 3 / 2}, {MAA, 0, 3 / 2},
    PlotStyle → {Gray, Opacity[0.5]},
    BoundaryStyle → None
  ];
```

```
Show[

 plotWinvA,
 plotWinvB,
 plotYaStable,

 PlotRange → {{0, 1.5}, {0, 1.5}},
 ImageSize → {xsize, xsize},
 PlotRangePadding → 0,
 FrameTicks → {Table[{x, x, ticksize}, {x, 0, 1, 1}],
   Table[{y, y, ticksize}, {y, 0, 1, 1}], None, None},
 FrameTicksStyle → {{Directive[Black, Thickness[lwd]],
    Directive[Black, Thickness[lwd]]}, {Directive[Black, Thickness[lwd]],
    Directive[Black, Thickness[lwd], FontColor → White]}},
 FrameStyle → {{Black, Thickness[lwd]}, {Black, Thickness[lwd]},
   {Black, Thickness[lwd]}, {Black, Thickness[lwd]}},
 FrameLabel → {"Relative fitness of aa males", ""},
 BaseStyle → {FontFamily → "Helvetica", FontSize → 14},
 ImagePadding → Pad,
 Epilog → {
   Text[Style["Ya equilibrium unstable", 14], {0.5, 1.25}],
   Rotate[
    Text[Style["Relative fitness of AA males", 14], Scaled@ylabpos], 90 Degree]
  },
 PlotLabel → Style["a favoured in females", 16, Black, Bold],
 PlotRangeClipping → False
]
```

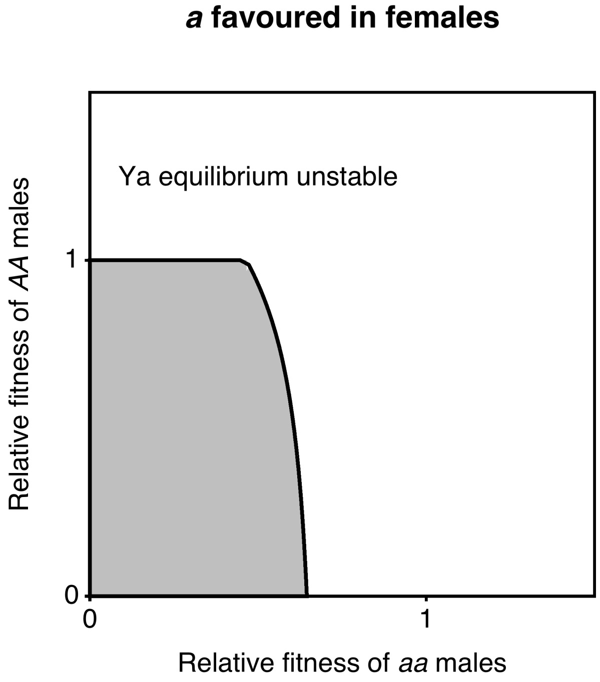

Panel C - overdominance in females, haploid competition favours a in females

Parameters

```
params = {
    wAm → 1, wam → 1, wAf → 1, waf → 1 + 8 / 50,
    αm → 1 / 2, αf → 1 / 2,
    MAa → 1, FAa → 1,
    FAA → 1 - 4 / 10,
    Faa → 1 - 4 / 10
   };
```

No recombination eigenvalues

```
WAinvA = λmA1 /. reverse /. pAveM → (1 - q) pXm + q pYm /. equilA0 /. params // Simplify;
WainvA = λma1 /. reverse /. pAveM → (1 - q) pXm + q pYm /. equilA0 /. params // Simplify;
WAinvB = λmA1 /. reverse /. pAveM → (1 - q) pXm + q pYm /. equilB0 /. params // Simplify;
WainvB = λma1 /. reverse /. pAveM → (1 - q) pXm + q pYm /. equilB0 /. params // Simplify;
```

Maximum absolute no recombination eigenvalue from the full characteristic polynomial

```
λWsolA =
  Max[Abs[λ /. Solve[0 == charpolyk1 /. r → 0 /. R → 0 /. ρ → 0 /. equilA0 /. params, λ] //
      Simplify]];
λWsolB = Max[Abs[λ /. Solve[0 == charpolyk1 /. r → 0 /. R → 0 /. ρ → 0 /. equilB0 /.
         params, λ] // Simplify]];
```

Plot

Region plots of invasion

```
(*neo-WA invades XY from equilA*)
plotWAinvA =
  RegionPlot[{
    (validcondA /. params) && (*valid*)
     (stabcondA /. Rf → 0 /. Rm → 0 /. equilA0 /. params) && (*internally stable*)
     1 < WAinvA (*invasion*)
   },
   {Maa, 0, 3 / 2}, {MAA, 0, 3 / 2},
   PlotStyle → {Gray, Opacity[0.5]},
   BoundaryStyle → None
  ];

(*neo-Wa invades XY from equilA*)
plotWainvA =
  RegionPlot[{
    (validcondA /. params) &&
     (stabcondA /. Rf → 0 /. Rm → 0 /. equilA0 /. params) &&
     1 < WainvA
   },
   {Maa, 0, 3 / 2}, {MAA, 0, 3 / 2},
   PlotStyle → {Gray, Opacity[0.5]},
   BoundaryStyle → None
  ];
```

```
(*neo-WA invades XY from equilB*)
plotWAinvB =
  RegionPlot[{
    (stabcondB /. Rf → 0 /. Rm → 0 /. equilB0 /. params) && (*internally stable*)
     1 < WAinvB(*invasion*)
   },
   {Maa, 0, 3 / 2}, {MAA, 0, 3 / 2},
   PlotStyle → {Gray, Opacity[0.5]},
   BoundaryStyle → None
  ];

(*neo-Wa invades XY from equilB*)
plotWainvB =
  RegionPlot[{
    (stabcondB /. Rf → 0 /. Rm → 0 /. equilB0 /. params) &&
     1 < WainvB
   },
   {Maa, 0, 3 / 2}, {MAA, 0, 3 / 2},
   PlotStyle → {Gray, Opacity[0.5]},
   BoundaryStyle → None
  ];

(*Ya equilibrium internally stable*)
plotYaStable =
  RegionPlot[{
    (stabcondA /. Rf → 0 /. Rm → 0 /. equilA0 /. params) ||
     (stabcondB /. Rf → 0 /. Rm → 0 /. equilB0 /. params)
   },
   {Maa, 0, 3 / 2}, {MAA, 0, 3 / 2},
   PlotStyle → White,
   BoundaryStyle → {Black, Thick}
  ];

(*Ya equilibrium internally unstable*)
plotYaUnstable =
  RegionPlot[{
    1 < 2
   },
   {Maa, 0, 3 / 2}, {MAA, 0, 3 / 2},
   PlotStyle → None,
   BoundaryStyle → None,
   Mesh → 50,
   MeshFunctions → {#1 - #2 &},
   MeshStyle → Black
  ];
```

```
plotC =
 Show[

  plotYaUnstable,
  plotYaStable,
  plotWAinvA,
  plotWainvA,
  plotWAinvB,
  plotWainvB,

  PlotRange → {{0, 1.5}, {0, 1.5}},
  ImageSize → {xsize, xsize},
  PlotRangePadding → 0,
  FrameTicks → {Table[{x, x, ticksize}, {x, 0, 1, 1}],
    Table[{y, y, ticksize}, {y, 0, 1, 1}], None, None},
  FrameTicksStyle → {{Directive[Black, Thickness[lwd]],
     Directive[Black, Thickness[lwd]]}, {Directive[Black, Thickness[lwd]],
     Directive[Black, Thickness[lwd], FontColor → White]}},
  FrameStyle → {{Black, Thickness[lwd]}, {Black, Thickness[lwd]},
    {Black, Thickness[lwd]}, {Black, Thickness[lwd]}},
  (*FrameLabel→{"Relative fitness of aa males",""},*)
  BaseStyle → {FontFamily → "Helvetica", FontSize → 14},
  ImagePadding → Pad,
  Epilog → {
    Text[Style["C", 14, Bold], Scaled@{0.05, 0.95}],
    Text[Style["Λ_{W'A}^{(XY)} >1
&
Λ_{W'a}^{(XY)} >1", 14], {0.4, 0.4}, {1, 0}],
    Text[Style["Λ_{W'A}^{(XY)} >1 & Λ_{W'a}^{(XY)} <1", 14], {1, 0.4}](*,
    Rotate[
     Text[Style["Relative fitness of AA males",14],Scaled@ylabpos],90 Degree]*)
   },
  PlotLabel → Style["overdominance in females", 16, Black, Bold],
  PlotRangeClipping → False
 ]
```

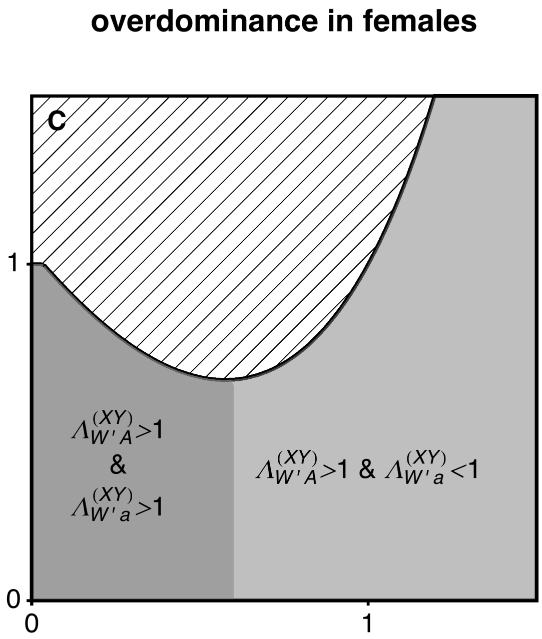

Notice that this is consistent with the solution from the full characteristic polynomial (but here we don’t know which eigenvalue belongs to which haplotype)

```
(*neo-W invades XY from equilA*)
plotWinvA =
  RegionPlot[{
    (validcondA /. params) && (*valid*)
     (stabcondA /. Rf → 0 /. Rm → 0 /. equilA0 /. params) && (*internally stable*)
     1 < λWsolA (*invasion*)
   },
   {Maa, 0, 3 / 2}, {MAA, 0, 3 / 2},
   PlotStyle → {Gray, Opacity[0.5]},
   BoundaryStyle → None
  ];

(*neo-W invades XY from equilB*)
plotWinvB =
  RegionPlot[{
    (stabcondB /. Rf → 0 /. Rm → 0 /. equilB0 /. params) && (*internally stable*)
     1 < λWsolB(*invasion*)
   },
   {Maa, 0, 3 / 2}, {MAA, 0, 3 / 2},
   PlotStyle → {Gray, Opacity[0.5]},
   BoundaryStyle → None
  ];
```

```
Show[

 plotWinvA,
 plotWinvB,
 plotYaStable,

 PlotRange → {{0, 1.5}, {0, 1.5}},
 ImageSize → {xsize, xsize},
 PlotRangePadding → 0,
 FrameTicks → {Table[{x, x, ticksize}, {x, 0, 1, 1}],
   Table[{y, y, ticksize}, {y, 0, 1, 1}], None, None},
 FrameTicksStyle → {{Directive[Black, Thickness[lwd]],
    Directive[Black, Thickness[lwd]]}, {Directive[Black, Thickness[lwd]],
    Directive[Black, Thickness[lwd], FontColor → White]}},
 FrameStyle → {{Black, Thickness[lwd]}, {Black, Thickness[lwd]},
   {Black, Thickness[lwd]}, {Black, Thickness[lwd]}},
 FrameLabel → {"Relative fitness of aa males", ""},
 BaseStyle → {FontFamily → "Helvetica", FontSize → 14},
 ImagePadding → Pad,
 Epilog → {
   Text[Style["Ya equilibrium unstable", 14], {0.5, 1.25}],
   Rotate[
    Text[Style["Relative fitness of AA males", 14], Scaled@ylabpos], 90 Degree]
  },
 PlotLabel → Style["A favoured in females", 16, Black, Bold],
 PlotRangeClipping → False
]
```

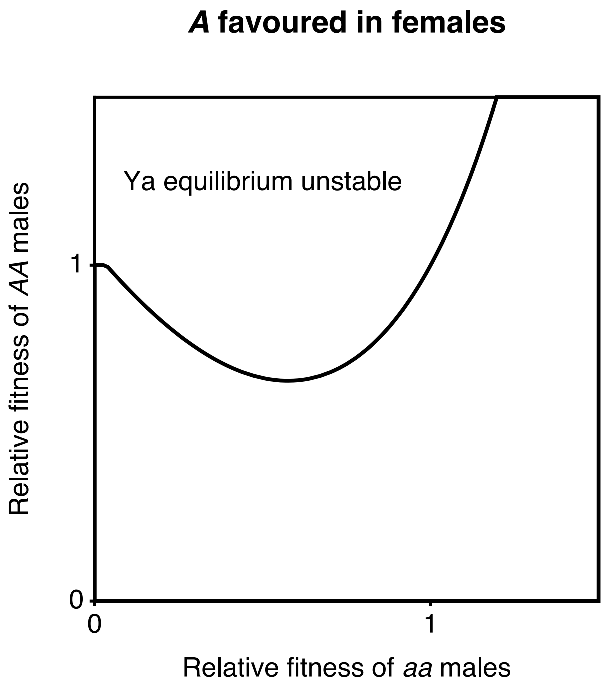

Panel D - A favoured in females, haploid competition favours A in females

Parameters

```
params = {
    wAm → 1, wam → 1, wAf → 1 + 8 / 50, waf → 1,
    αm → 1 / 2, αf → 1 / 2,
    MAa → 1, FAa → 1,
    Faa → 1 - 3 / 20,
    FAA → 1 + 1 / 20
  };
```

No recombination eigenvalues

```
WAinvA = λmA1 /. reverse /. pAveM → (1 - q) pXm + q pYm /. equilA0 /. params // Simplify;
WainvA = λma1 /. reverse /. pAveM → (1 - q) pXm + q pYm /. equilA0 /. params // Simplify;
WAinvB = λmA1 /. reverse /. pAveM → (1 - q) pXm + q pYm /. equilB0 /. params // Simplify;
WainvB = λma1 /. reverse /. pAveM → (1 - q) pXm + q pYm /. equilB0 /. params // Simplify;
```

Maximum absolute no recombination eigenvalue from the full characteristic polynomial

```
λWsolA =
  Max[Abs[λ /. Solve[0 == charpolyk1 /. r → 0 /. R → 0 /. ρ → 0 /. equilA0 /. params, λ] //
      Simplify]];
λWsolB = Max[Abs[λ /. Solve[0 == charpolyk1 /. r → 0 /. R → 0 /. ρ → 0 /. equilB0 /.
         params, λ] // Simplify]];
```

Plot

Region plots of invasion

```
(*neo-WA invades XY from equilA*)
plotWAinvA =
  RegionPlot[{
     (validcondA /. params) && (*valid*)
      (stabcondA /. Rf → 0 /. Rm → 0 /. equilA0 /. params) && (*internally stable*)
      1 < WAinvA (*invasion*)
    },
    {Maa, 0, 3 / 2}, {MAA, 0, 3 / 2},
    PlotStyle → {Gray, Opacity[0.5]},
    BoundaryStyle → None
  ];

(*neo-Wa invades XY from equilA*)
plotWainvA =
  RegionPlot[{
     (validcondA /. params) &&
      (stabcondA /. Rf → 0 /. Rm → 0 /. equilA0 /. params) &&
      1 < WainvA
    },
    {Maa, 0, 3 / 2}, {MAA, 0, 3 / 2},
    PlotStyle → {Gray, Opacity[0.5]},
    BoundaryStyle → None
  ];
```

```
(*neo-WA invades XY from equilB*)
plotWAinvB =
  RegionPlot[{
    (stabcondB /. Rf → 0 /. Rm → 0 /. equilB0 /. params) && (*internally stable*)
     1 < WAinvB(*invasion*)
   },
   {Maa, 0, 3 / 2}, {MAA, 0, 3 / 2},
   PlotStyle → {Gray, Opacity[0.5]},
   BoundaryStyle → None
  ];

(*neo-Wa invades XY from equilB*)
plotWainvB =
  RegionPlot[{
    (stabcondB /. Rf → 0 /. Rm → 0 /. equilB0 /. params) &&
     1 < WainvB
   },
   {Maa, 0, 3 / 2}, {MAA, 0, 3 / 2},
   PlotStyle → {Gray, Opacity[0.5]},
   BoundaryStyle → None
  ];

(*Ya equilibrium internally stable*)
plotYaStable =
  RegionPlot[{
    (stabcondA /. Rf → 0 /. Rm → 0 /. equilA0 /. params) ||
     (stabcondB /. Rf → 0 /. Rm → 0 /. equilB0 /. params)
   },
   {Maa, 0, 3 / 2}, {MAA, 0, 3 / 2},
   PlotStyle → White,
   BoundaryStyle → {Black, Thick}
  ];

(*Ya equilibrium internally unstable*)
plotYaUnstable =
  RegionPlot[{
    1 < 2
   },
   {Maa, 0, 3 / 2}, {MAA, 0, 3 / 2},
   PlotStyle → None,
   BoundaryStyle → None,
   Mesh → 50,
   MeshFunctions → {#1 - #2 &},
   MeshStyle → Black
  ];
```

```
plotD =
 Show[

  plotYaUnstable,
  plotYaStable,
  plotWAinvA,
  plotWainvA,
  plotWAinvB,
  plotWainvB,

  Graphics[{Thick, Black, Line[{{1.35, 0.5}, {1.475, 0.75}}]}],
  PlotRange → {{0, 1.5}, {0, 1.5}},
  ImageSize → {xsize, xsize},
  PlotRangePadding → 0,
  FrameTicks → {Table[{x, x, ticksize}, {x, 0, 1, 1}],
    Table[{y, y, ticksize}, {y, 0, 1, 1}], None, None},
  FrameTicksStyle → {{Directive[Black, Thickness[lwd]],
     Directive[Black, Thickness[lwd]]}, {Directive[Black, Thickness[lwd]],
     Directive[Black, Thickness[lwd], FontColor → White]}},
  FrameStyle → {{Black, Thickness[lwd]}, {Black, Thickness[lwd]},
    {Black, Thickness[lwd]}, {Black, Thickness[lwd]}},
  FrameLabel → {"Relative fitness of aa males", ""},
  BaseStyle → {FontFamily → "Helvetica", FontSize → 14},
  ImagePadding → Pad,
  Epilog → {
    Text[Style["D", 14, Bold], Scaled@{0.05, 0.95}],
    Text[Style["Λ_{W'A}^{(XY)}<1 & Λ_{W'a}^{(XY)}<1", 14], {0.5, 0.5}],
    Text[Style["Λ_{W'A}^{(XY)}>1
&
Λ_{W'a}^{(XY)}<1", 14], {1.35, 0.5}, {1, 0}],
    Rotate[
     Text[Style["Relative fitness of AA males", 14], Scaled@ylabpos], 90 Degree]
   },
  PlotLabel → Style[(*"A favoured in females"*)"", 16, Black, Bold],
  PlotRangeClipping → False
 ]
```

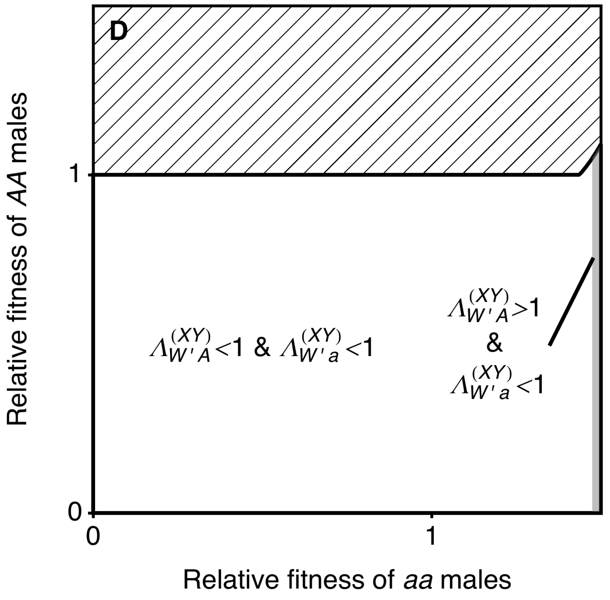

Notice that this is consistent with the solution from the full characteristic polynomial (but here we don't know which eigenvalue belongs to which haplotype)

```
(*neo-W invades XY from equilA*)
plotWinvA =
  RegionPlot[{
    (validcondA /. params) && (*valid*)
     (stabcondA /. Rf → 0 /. Rm → 0 /. equilA0 /. params) && (*internally stable*)
     1 < λWsolA (*invasion*)
   },
   {Maa, 0, 3 / 2}, {MAA, 0, 3 / 2},
   PlotStyle → {Gray, Opacity[0.5]},
   BoundaryStyle → None
  ];

(*neo-W invades XY from equilB*)
plotWinvB =
  RegionPlot[{
    (stabcondB /. Rf → 0 /. Rm → 0 /. equilB0 /. params) && (*internally stable*)
     1 < λWsolB(*invasion*)
   },
   {Maa, 0, 3 / 2}, {MAA, 0, 3 / 2},
   PlotStyle → {Gray, Opacity[0.5]},
   BoundaryStyle → None
  ];
```

```
Show[

 plotWinvA,
 plotWinvB,
 plotYaStable,

 PlotRange → {{0, 1.5}, {0, 1.5}},
 ImageSize → {xsize, xsize},
 PlotRangePadding → 0,
 FrameTicks → {Table[{x, x, ticksize}, {x, 0, 1, 1}],
   Table[{y, y, ticksize}, {y, 0, 1, 1}], None, None},
 FrameTicksStyle → {{Directive[Black, Thickness[lwd]],
    Directive[Black, Thickness[lwd]]}, {Directive[Black, Thickness[lwd]],
    Directive[Black, Thickness[lwd], FontColor → White]}},
 FrameStyle → {{Black, Thickness[lwd]}, {Black, Thickness[lwd]},
   {Black, Thickness[lwd]}, {Black, Thickness[lwd]}},
 FrameLabel → {"Relative fitness of aa males", ""},
 BaseStyle → {FontFamily → "Helvetica", FontSize → 14},
 ImagePadding → Pad,
 Epilog → {
   Text[Style["Ya equilibrium unstable", 14], {0.5, 1.25}],
   Rotate[
    Text[Style["Relative fitness of AA males", 14], Scaled@ylabpos], 90 Degree]
  },
 PlotLabel → Style["A favoured in females", 16, Black, Bold],
 PlotRangeClipping → False
]
```

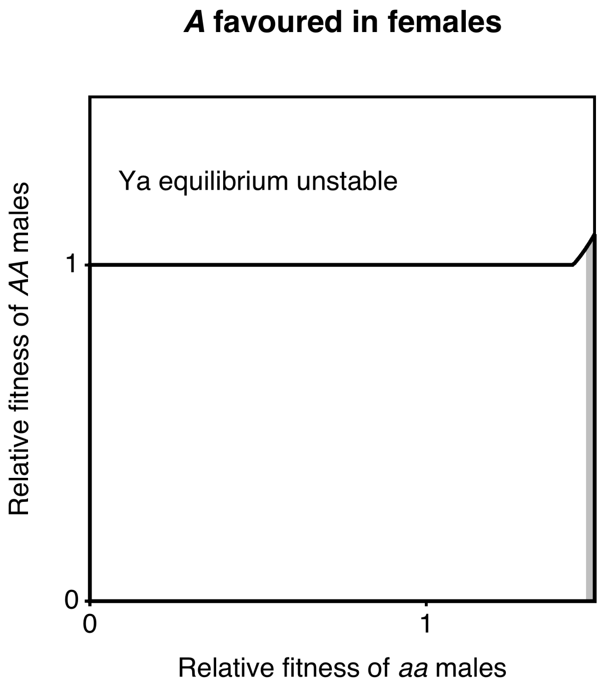

Panel E - a favoured in females, haploid competition favours A in females

Parameters

```
params = {
    wAm → 1, wam → 1, wAf → 1 + 8 / 50, waf → 1,
    αm → 1 / 2, αf → 1 / 2,
    MAa → 1, FAa → 1,
    FAA → 1 - 3 / 20,
    Faa → 1 + 1 / 20
   };
```

No recombination eigenvalues

```
WAinvA = λmA1 /. reverse /. pAveM → (1 - q) pXm + q pYm /. equilA0 /. params // Simplify;
WainvA = λma1 /. reverse /. pAveM → (1 - q) pXm + q pYm /. equilA0 /. params // Simplify;
WAinvB = λmA1 /. reverse /. pAveM → (1 - q) pXm + q pYm /. equilB0 /. params // Simplify;
WainvB = λma1 /. reverse /. pAveM → (1 - q) pXm + q pYm /. equilB0 /. params // Simplify;
```

Maximum absolute no recombination eigenvalue from the full characteristic polynomial

```
λWsolA =
  Max[Abs[λ /. Solve[0 == charpolyk1 /. r → 0 /. R → 0 /. ρ → 0 /. equilA0 /. params, λ] //
      Simplify]];
λWsolB = Max[Abs[λ /. Solve[0 == charpolyk1 /. r → 0 /. R → 0 /. ρ → 0 /. equilB0 /.
         params, λ] // Simplify]];
```

Plot

Region plots of invasion

```
(*neo-WA invades XY from equilA*)
plotWAinvA =
  RegionPlot[{
     (validcondA /. params) && (*valid*)
      (stabcondA /. Rf → 0 /. Rm → 0 /. equilA0 /. params) && (*internally stable*)
      1 < WAinvA (*invasion*)
    },
    {Maa, 0, 3 / 2}, {MAA, 0, 3 / 2},
    PlotStyle → {Gray, Opacity[0.5]},
    BoundaryStyle → None
   ];

(*neo-Wa invades XY from equilA*)
plotWainvA =
  RegionPlot[{
     (validcondA /. params) &&
      (stabcondA /. Rf → 0 /. Rm → 0 /. equilA0 /. params) &&
      1 < WainvA
    },
    {Maa, 0, 3 / 2}, {MAA, 0, 3 / 2},
    PlotStyle → {Gray, Opacity[0.5]},
    BoundaryStyle → None
   ];
```

```
(*neo-WA invades XY from equilB*)
plotWAinvB =
  RegionPlot[{
    (stabcondB /. Rf → 0 /. Rm → 0 /. equilB0 /. params) && (*internally stable*)
     1 < WAinvB(*invasion*)
   },
   {Maa, 0, 3 / 2}, {MAA, 0, 3 / 2},
   PlotStyle → {Gray, Opacity[0.5]},
   BoundaryStyle → None
  ];

(*neo-Wa invades XY from equilB*)
plotWainvB =
  RegionPlot[{
    (stabcondB /. Rf → 0 /. Rm → 0 /. equilB0 /. params) &&
     1 < WainvB
   },
   {Maa, 0, 3 / 2}, {MAA, 0, 3 / 2},
   PlotStyle → {Gray, Opacity[0.5]},
   BoundaryStyle → None
  ];

(*Ya equilibrium internally stable*)
plotYaStable =
  RegionPlot[{
    (stabcondA /. Rf → 0 /. Rm → 0 /. equilA0 /. params) ||
     (stabcondB /. Rf → 0 /. Rm → 0 /. equilB0 /. params)
   },
   {Maa, 0, 3 / 2}, {MAA, 0, 3 / 2},
   PlotStyle → White,
   BoundaryStyle → {Black, Thick}
  ];

(*Ya equilibrium internally unstable*)
plotYaUnstable =
  RegionPlot[{
    1 < 2
   },
   {Maa, 0, 3 / 2}, {MAA, 0, 3 / 2},
   PlotStyle → None,
   BoundaryStyle → None,
   Mesh → 50,
   MeshFunctions → {#1 - #2 &},
   MeshStyle → Black
  ];
```

```
plotE =
 Show[

  plotYaUnstable,
  plotYaStable,
  plotWAinvA,
  plotWainvA,
  plotWAinvB,
  plotWainvB,

  Graphics[{Thick, Black, Line[{{1.15, 1.1}, {0.9, 1.15}}]}],
  PlotRange → {{0, 1.5}, {0, 1.5}},
  ImageSize → {xsize, xsize},
  PlotRangePadding → 0,
  FrameTicks → {Table[{x, x, ticksize}, {x, 0, 1, 1}],
    Table[{y, y, ticksize}, {y, 0, 1, 1}], None, None},
  FrameTicksStyle → {{Directive[Black, Thickness[lwd]],
     Directive[Black, Thickness[lwd]]}, {Directive[Black, Thickness[lwd]],
     Directive[Black, Thickness[lwd], FontColor → White]}},
  FrameStyle → {{Black, Thickness[lwd]}, {Black, Thickness[lwd]},
    {Black, Thickness[lwd]}, {Black, Thickness[lwd]}},
  FrameLabel → {"Relative fitness of aa males", ""},
  BaseStyle → {FontFamily → "Helvetica", FontSize → 14},
  ImagePadding → Pad,
  Epilog → {
    Text[Style["E", 14, Bold], Scaled@{0.05, 0.95}],
    Text[Style["Λ^(XY)_W'A >1 & Λ^(XY)_W'a >1", 14], {0.4, 0.5}],
    Text[Style["Λ^(XY)_W'A >1 & Λ^(XY)_W'a <1", 14], {0.5, 1.2}, Background → White](*,
    Rotate[
     Text[Style["Relative fitness of AA males",14],Scaled@ylabpos],90 Degree]*)
   },
  PlotLabel → Style[(*"a favoured in females"*)"", 16, Black, Bold],
  PlotRangeClipping → False
 ]
```

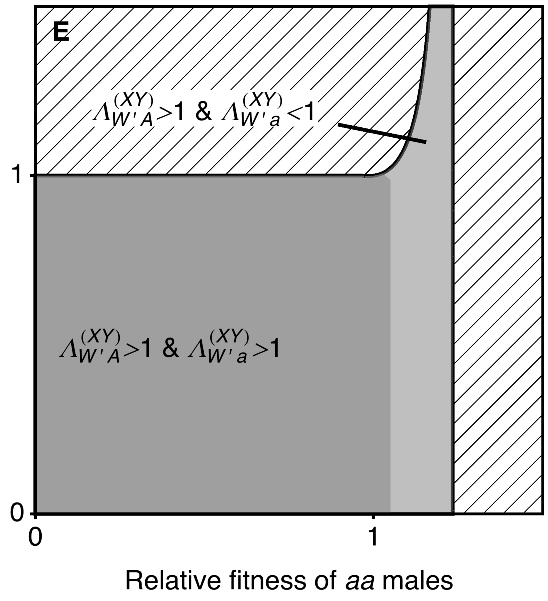

Notice that this is consistent with the solution from the full characteristic polynomial (but here we don’t know which eigenvalue belongs to which haplotype)

```
(*neo-W invades XY from equilA*)
plotWinvA =
  RegionPlot[{
    (validcondA /. params) && (*valid*)
     (stabcondA /. Rf → 0 /. Rm → 0 /. equilA0 /. params) && (*internally stable*)
     1 < λWsolA (*invasion*)
   },
   {Maa, 0, 3 / 2}, {MAA, 0, 3 / 2},
   PlotStyle → {Gray, Opacity[0.5]},
   BoundaryStyle → None
  ];

(*neo-W invades XY from equilB*)
plotWinvB =
  RegionPlot[{
    (stabcondB /. Rf → 0 /. Rm → 0 /. equilB0 /. params) && (*internally stable*)
     1 < λWsolB(*invasion*)
   },
   {Maa, 0, 3 / 2}, {MAA, 0, 3 / 2},
   PlotStyle → {Gray, Opacity[0.5]},
   BoundaryStyle → None
  ];
```

```
Show[

 plotWinvA,
 plotWinvB,
 plotYaStable,

 PlotRange → {{0, 1.5}, {0, 1.5}},
 ImageSize → {xsize, xsize},
 PlotRangePadding → 0,
 FrameTicks → {Table[{x, x, ticksize}, {x, 0, 1, 1}],
   Table[{y, y, ticksize}, {y, 0, 1, 1}], None, None},
 FrameTicksStyle → {{Directive[Black, Thickness[lwd]],
    Directive[Black, Thickness[lwd]]}, {Directive[Black, Thickness[lwd]],
    Directive[Black, Thickness[lwd], FontColor → White]}},
 FrameStyle → {{Black, Thickness[lwd]}, {Black, Thickness[lwd]},
   {Black, Thickness[lwd]}, {Black, Thickness[lwd]}},
 FrameLabel → {"Relative fitness of aa males", ""},
 BaseStyle → {FontFamily → "Helvetica", FontSize → 14},
 ImagePadding → Pad,
 Epilog → {
   Text[Style["Ya equilibrium unstable", 14], {0.5, 1.25}],
   Rotate[
    Text[Style["Relative fitness of AA males", 14], Scaled@ylabpos], 90 Degree]
  },
 PlotLabel → Style["a favoured in females", 16, Black, Bold],
 PlotRangeClipping → False
]
```

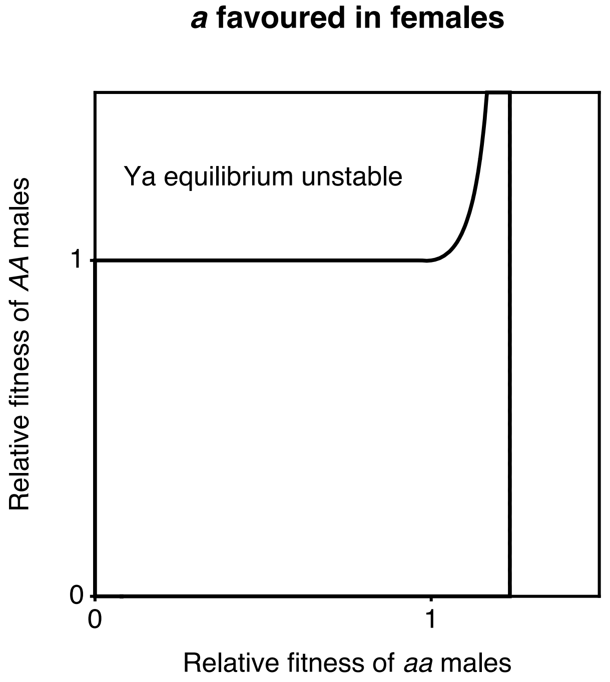

Panel F - overdominance in females, haploid competition favours A in females

Parameters

```
params = {
    wAm → 1, wam → 1, wAf → 1 + 8 / 50, waf → 1,
    αm → 1 / 2, αf → 1 / 2,
    MAa → 1, FAa → 1,
    FAA → 1 - 4 / 10,
    Faa → 1 - 4 / 10
   };
```

No recombination eigenvalues

```
WAinvA = λmA1 /. reverse /. pAveM → (1 - q) pXm + q pYm /. equilA0 /. params // Simplify;
WainvA = λma1 /. reverse /. pAveM → (1 - q) pXm + q pYm /. equilA0 /. params // Simplify;
WAinvB = λmA1 /. reverse /. pAveM → (1 - q) pXm + q pYm /. equilB0 /. params // Simplify;
WainvB = λma1 /. reverse /. pAveM → (1 - q) pXm + q pYm /. equilB0 /. params // Simplify;
```

Maximum absolute no recombination eigenvalue from the full characteristic polynomial

```
λWsolA =
  Max[Abs[λ /. Solve[0 == charpolyk1 /. r → 0 /. R → 0 /. ρ → 0 /. equilA0 /. params, λ] //
     Simplify]];
λWsolB = Max[Abs[λ /. Solve[0 == charpolyk1 /. r → 0 /. R → 0 /. ρ → 0 /. equilB0 /.
        params, λ] // Simplify]];
```

Plot

Region plots of invasion

```
(*neo-WA invades XY from equilA*)
plotWAinvA =
  RegionPlot[{
    (validcondA /. params) && (*valid*)
     (stabcondA /. Rf → 0 /. Rm → 0 /. equilA0 /. params) && (*internally stable*)
     1 < WAinvA (*invasion*)
   },
   {Maa, 0, 3 / 2}, {MAA, 0, 3 / 2},
   PlotStyle → {Gray, Opacity[0.5]},
   BoundaryStyle → None
  ];

(*neo-Wa invades XY from equilA*)
plotWainvA =
  RegionPlot[{
    (validcondA /. params) &&
     (stabcondA /. Rf → 0 /. Rm → 0 /. equilA0 /. params) &&
     1 < WainvA
   },
   {Maa, 0, 3 / 2}, {MAA, 0, 3 / 2},
   PlotStyle → {Gray, Opacity[0.5]},
   BoundaryStyle → None
  ];
```

```
(*neo-WA invades XY from equilB*)
plotWAinvB =
  RegionPlot[{
    (stabcondB /. Rf → 0 /. Rm → 0 /. equilB0 /. params) && (*internally stable*)
     1 < WAinvB(*invasion*)
   },
   {Maa, 0, 3 / 2}, {MAA, 0, 3 / 2},
   PlotStyle → {Gray, Opacity[0.5]},
   BoundaryStyle → None
  ];

(*neo-Wa invades XY from equilB*)
plotWainvB =
  RegionPlot[{
    (stabcondB /. Rf → 0 /. Rm → 0 /. equilB0 /. params) &&
     1 < WainvB
   },
   {Maa, 0, 3 / 2}, {MAA, 0, 3 / 2},
   PlotStyle → {Gray, Opacity[0.5]},
   BoundaryStyle → None
  ];

(*Ya equilibrium internally stable*)
plotYaStable =
  RegionPlot[{
    (stabcondA /. Rf → 0 /. Rm → 0 /. equilA0 /. params) ||
     (stabcondB /. Rf → 0 /. Rm → 0 /. equilB0 /. params)
   },
   {Maa, 0, 3 / 2}, {MAA, 0, 3 / 2},
   PlotStyle → White,
   BoundaryStyle → {Black, Thick}
  ];

(*Ya equilibrium internally unstable*)
plotYaUnstable =
  RegionPlot[{
    1 < 2
   },
   {Maa, 0, 3 / 2}, {MAA, 0, 3 / 2},
   PlotStyle → None,
   BoundaryStyle → None,
   Mesh → 50,
   MeshFunctions → {#1 - #2 &},
   MeshStyle → Black
  ];
```

```
plotF =
 Show[

  plotYaUnstable,
  plotYaStable,
  plotWAinvA,
  plotWainvA,
  plotWAinvB,
  plotWainvB,

  PlotRange → {{0, 1.5}, {0, 1.5}},
  ImageSize → {xsize, xsize},
  PlotRangePadding → 0,
  FrameTicks → {Table[{x, x, ticksize}, {x, 0, 1, 1}],
    Table[{y, y, ticksize}, {y, 0, 1, 1}], None, None},
  FrameTicksStyle → {{Directive[Black, Thickness[lwd]],
     Directive[Black, Thickness[lwd]]}, {Directive[Black, Thickness[lwd]],
     Directive[Black, Thickness[lwd], FontColor → White]}},
  FrameStyle → {{Black, Thickness[lwd]}, {Black, Thickness[lwd]},
    {Black, Thickness[lwd]}, {Black, Thickness[lwd]}},
  FrameLabel → {"Relative fitness of aa males", ""},
  BaseStyle → {FontFamily → "Helvetica", FontSize → 14},
  ImagePadding → Pad,
  Epilog → {
    Text[Style["F", 14, Bold], Scaled@{0.05, 0.95}],
    Text[Style["Λ_{W'A}^(XY) >1
&
Λ_{W'a}^(XY) >1", 14], {0.4, 0.4}, {1, 0}],
    Text[Style["Λ_{W'A}^(XY) <1 & Λ_{W'a}^(XY) >1", 14], {1, 0.4}](*,
    Rotate[
     Text[Style["Relative fitness of AA males",14],Scaled@ylabpos],90 Degree]*)
   },
  PlotLabel → Style[(*"overdominance in females"*)"", 16, Black, Bold],
  PlotRangeClipping → False
 ]
```

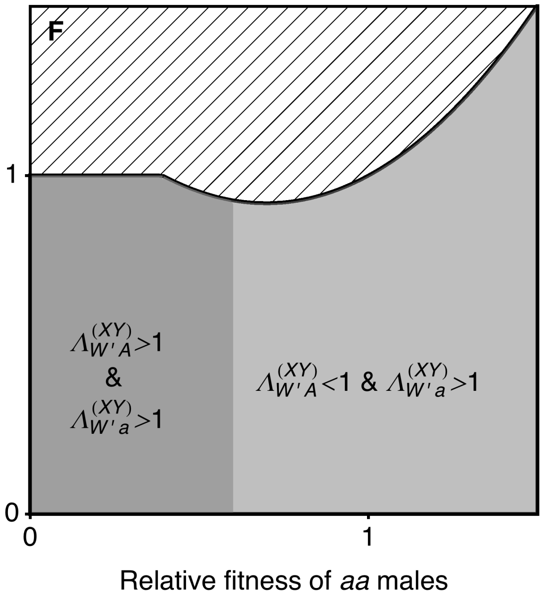

Notice that this is consistent with the solution from the full characteristic polynomial (but here we don't know which eigenvalue belongs to which haplotype)

```
(*neo-W invades XY from equilA*)
plotWinvA =
  RegionPlot[{
     (validcondA /. params) && (*valid*)
      (stabcondA /. Rf → 0 /. Rm → 0 /. equilA0 /. params) && (*internally stable*)
      1 < λWsolA (*invasion*)
    },
    {Maa, 0, 3 / 2}, {MAA, 0, 3 / 2},
    PlotStyle → {Gray, Opacity[0.5]},
    BoundaryStyle → None
  ];

(*neo-W invades XY from equilB*)
plotWinvB =
  RegionPlot[{
     (stabcondB /. Rf → 0 /. Rm → 0 /. equilB0 /. params) && (*internally stable*)
      1 < λWsolB(*invasion*)
    },
    {Maa, 0, 3 / 2}, {MAA, 0, 3 / 2},
    PlotStyle → {Gray, Opacity[0.5]},
    BoundaryStyle → None
  ];
```

```
Show[

 plotWinvA,
 plotWinvB,
 plotYaStable,

 PlotRange → {{0, 1.5}, {0, 1.5}},
 ImageSize → {xsize, xsize},
 PlotRangePadding → 0,
 FrameTicks → {Table[{x, x, ticksize}, {x, 0, 1, 1}],
   Table[{y, y, ticksize}, {y, 0, 1, 1}], None, None},
 FrameTicksStyle → {{Directive[Black, Thickness[lwd]],
    Directive[Black, Thickness[lwd]]}, {Directive[Black, Thickness[lwd]],
    Directive[Black, Thickness[lwd], FontColor → White]}},
 FrameStyle → {{Black, Thickness[lwd]}, {Black, Thickness[lwd]},
   {Black, Thickness[lwd]}, {Black, Thickness[lwd]}},
 FrameLabel → {"Relative fitness of aa males", ""},
 BaseStyle → {FontFamily → "Helvetica", FontSize → 14},
 ImagePadding → Pad,
 Epilog → {
   Text[Style["Ya equilibrium unstable", 14], {0.5, 1.25}],
   Rotate[
    Text[Style["Relative fitness of AA males", 14], Scaled@ylabpos], 90 Degree]
  },
 PlotLabel → Style["overdominance in females", 16, Black, Bold],
 PlotRangeClipping → False
]
```

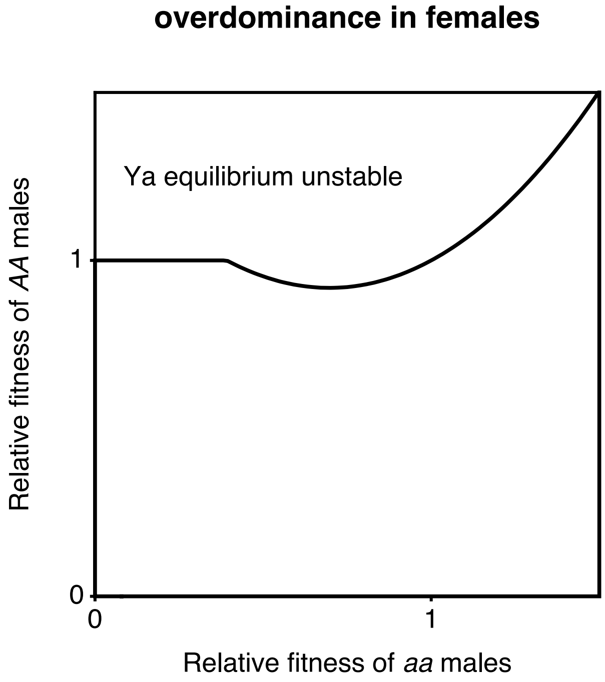

All Panels

```
GraphicsGrid[{
   {plotA, plotB, plotC},
   {plotD, plotE, plotF}
  },
  Spacings → -50
]

Export[plotdir <> "Region_plot_combined_FemaleGS.eps", % // rasterTrick];
```

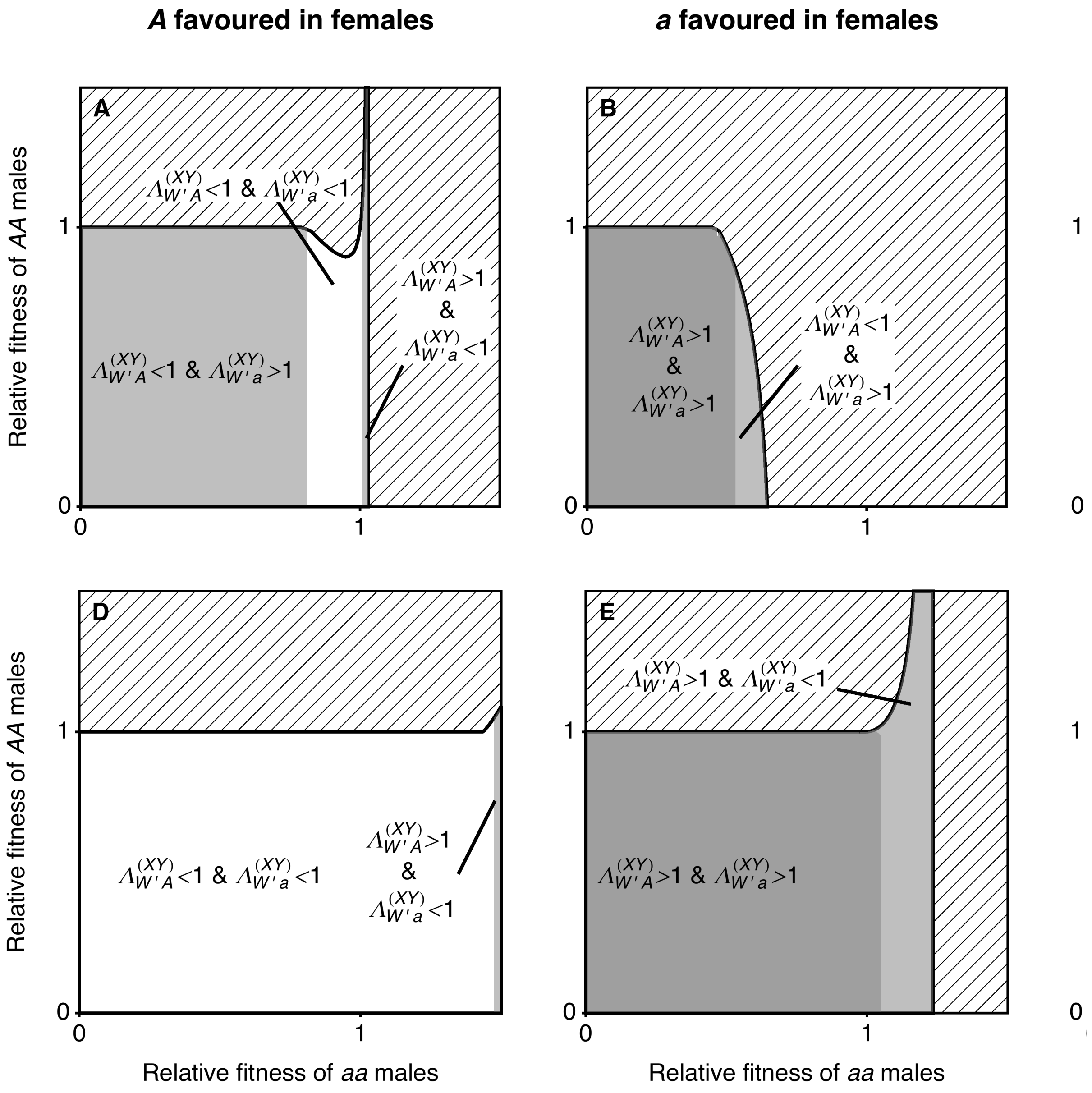

Figure S.8 - neo-W haplotype invasion with ploidally-antagonistic selection

extra parameters (move to top)

```
tryr = 1 / 200;
tryk = 1;
trypm = 0.01;
```

```
startplot = 5;
endtime = 15 000;
```

```
loglinearplotINVoptions = {
   Frame → {{True, False}, {True, False}},
   FrameStyle → Directive[Black, Thickness[lwd]],
   FrameTicksStyle → {{Directive[Black, Thickness[lwd], FontColor → Black],
      Directive[Black, Thickness[lwd]]},
     {Directive[Black, Thickness[lwd]], Directive[Black, Thickness[lwd]]}},
   FrameTicks → {{(*{5,5,{0,0.01}},*){10, 10, {0, 0.01}}, (*{50,50,{0,0.01}},*)
      {100, 100, {0, 0.01}}, (*{500,500,{0,0.01}},*){1000, 1000, {0, 0.01}}
      (*,{5000,5000,{0,0.01}}*), {10 000, 10 000, {0, 0.01}}},
     Table[{y, y, ticksize}, {y, 0, 0.5, 0.5}]},
   BaseStyle → {FontFamily → "Helvetica", FontSize → 14},
   ImageSize → {xsize, xsize aspectratio },
   AspectRatio → aspectratio,
   Axes → True,
   FrameLabel → {Style["Generation", FontSize → 14],},
   ImagePadding → Pad
  };
```

```
modvec = {0, 0, 1, 1, 0, 0, 1, 1, 0, 0, 0, 0, 0, 0, 0, 0};
```

Panel A - invasion region with drive in males

Parameters

```
params = {
   wAm → 1, wam → 1, wAf → 1, waf → 1,
   αm → (1 + αmd) / 2, αf → 1 / 2,
   Maa → 1, Faa → 1,
   MAa → 1 + (3 / 4) s, FAa → 1 + (3 / 4) s,
   MAA → 1 + s, FAA → 1 + s
  };
```

No recombination eigenvalues

```
WAinvA = λmA1 /. reverse /. pAveM → (1 - q) pXm + q pYm /. equilA0 /. params // Simplify;
WainvA = λma1 /. reverse /. pAveM → (1 - q) pXm + q pYm /. equilA0 /. params // Simplify;
WAinvB = λmA1 /. reverse /. pAveM → (1 - q) pXm + q pYm /. equilB0 /. params // Simplify;
WainvB = λma1 /. reverse /. pAveM → (1 - q) pXm + q pYm /. equilB0 /. params // Simplify;
```

Maximum absolute no recombination eigenvalue from the full characteristic polynomial

```
λWsolA = Max[Abs[λ /. Solve[
      0 == charpolyk1 /. r → 0 /. R → 0 /. ρ → 0 /. equilA0 /. params // Simplify, λ]]];
λWsolB = Max[Abs[λ /. Solve[0 == charpolyk1 /. r → 0 /. R → 0 /. ρ → 0 /. equilB0 /.
        params // Simplify, λ]]];
```

Plot

Region plots of invasion

```
(*neo-WA invades XY from equilA*)
plotWAinvA =
  RegionPlot[{
    (validcondA /. params) && (*valid*)
     (stabcondA /. Rf → 0 /. Rm → 0 /. equilA0 /. params) && (*internally stable*)
     1 < WAinvA (*invasion*)
   },
   {αmd, -1, 1}, {s, -1, 1},
   PlotStyle → {Gray, Opacity[0.5]},
   BoundaryStyle → None
  ];

(*neo-Wa invades XY from equilA*)
plotWainvA =
  RegionPlot[{
    (validcondA /. params) &&
     (stabcondA /. Rf → 0 /. Rm → 0 /. equilA0 /. params) &&
     1 < WainvA
   },
   {αmd, -1, 1}, {s, -1, 1},
   PlotStyle → {Gray, Opacity[0.5]},
   BoundaryStyle → None
  ];

(*neo-WA invades XY from equilB*)
plotWAinvB =
  RegionPlot[{
    (stabcondB /. Rf → 0 /. Rm → 0 /. equilB0 /. params) && (*internally stable*)
     1 < WAinvB(*invasion*)
   },
   {αmd, -1, 1}, {s, -1, 1},
   PlotStyle → {Gray, Opacity[0.5]},
   BoundaryStyle → None
  ];

(*neo-Wa invades XY from equilB*)
plotWainvB =
  RegionPlot[{
    (stabcondB /. Rf → 0 /. Rm → 0 /. equilB0 /. params) &&
     1 < WainvB
   },
   {αmd, -1, 1}, {s, -1, 1},
   PlotStyle → {Gray, Opacity[0.5]},
   BoundaryStyle → None
```

```
  ];

(*Ya equilibrium internally stable*)
plotYaStable =
  RegionPlot[{
     (stabcondA /. Rf → 0 /. Rm → 0 /. equilA0 /. params) ||
      (stabcondB /. Rf → 0 /. Rm → 0 /. equilB0 /. params)
    },
    {αmd, -1, 1}, {s, -1, 1},
    PlotStyle → White,
    BoundaryStyle → {Black, Thick}
   ];

(*Ya equilibrium internally unstable*)
plotYaUnstable =
  RegionPlot[{
     1 < 2
    },
    {αmd, -1, 1}, {s, -1, 1},
    PlotStyle → None,
    BoundaryStyle → None,
    Mesh → 50,
    MeshFunctions → {#1 - #2 &},
    MeshStyle → Black
   ];
```

```
plotA =
 Show[

  plotYaUnstable,
  plotYaStable,
  plotWAinvA,
  plotWainvA,
  plotWAinvB,
  plotWainvB,

  Graphics[{Thick, Black, Line[{{-0.15, 0.5}, {0.1, 0.5}}]}],
  PlotRange → {{-1, 1}, {-1, 1}},
  ImageSize → {xsize, xsize},
  PlotRangePadding → 0,
  FrameTicks → {Table[{x, x, ticksize}, {x, -1, 1, 1}],
    Table[{y, y, ticksize}, {y, -1, 1, 1}], None, None},
  FrameTicksStyle → {{Directive[Black, Thickness[lwd]],
     Directive[Black, Thickness[lwd]]}, {Directive[Black, Thickness[lwd]],
     Directive[Black, Thickness[lwd], FontColor → White]}},
  FrameStyle → {{Black, Thickness[lwd]}, {Black, Thickness[lwd]},
    {Black, Thickness[lwd]}, {Black, Thickness[lwd]}},
  FrameLabel → {"Drive for A in males", ""},
  BaseStyle → {FontFamily → "Helvetica", FontSize → 14},
  ImagePadding → Pad,
  Epilog → {
    Text[Style["A", 14, Bold], Scaled@{0.95, 0.95}],
    Text[Style["Λ_{W'A}^{(XY)} >1
&
   Λ_{W'a}^{(XY)} >1", 14], {-0.55, 0.75}],
    Text[Style["Λ_{W'A}^{(XY)} >1
&
Λ_{W'a}^{(XY)} <1", 14], {0.2, 0.5}, Background → White],
    Text[Style["*", 16, Bold], {-0.1, 0.25}],
    Rotate[Text[Style["Diploid selection for A", 14], Scaled@ylabpos], 90 Degree]
   },
  PlotLabel → Style["drive in males", 16, Black, Bold],
  PlotRangeClipping → False
 ]
```

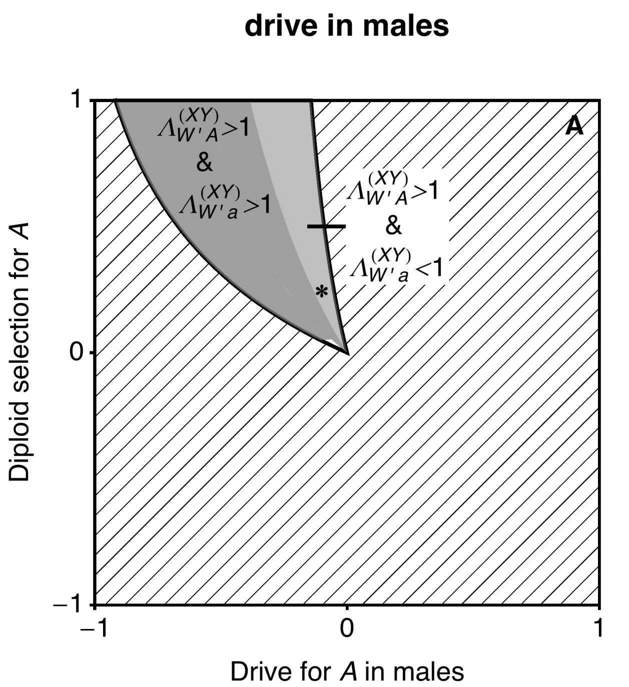

Notice that this is consistent with the solution from the full characteristic polynomial (but here we don't know which eigenvalue belongs to which haplotype)

```
(*neo-W invades XY from equilA*)
plotWinvA =
  RegionPlot[{
    (validcondA /. params) && (*valid*)
     (stabcondA /. Rf → 0 /. Rm → 0 /. equilA0 /. params) && (*internally stable*)
     1 < λWsolA(*invasion*)
   },
   {αmd, -1, 1}, {s, -1, 1},
   PlotStyle → {Gray, Opacity[0.5]},
   BoundaryStyle → None
  ];

(*neo-W invades XY from equilB*)
plotWinvB =
  RegionPlot[{
    (stabcondB /. Rf → 0 /. Rm → 0 /. equilB0 /. params) && (*internally stable*)
     1 < λWsolB(*invasion*)
   },
   {αmd, -1, 1}, {s, -1, 1},
   PlotStyle → {Gray, Opacity[0.5]},
   BoundaryStyle → None
  ];
```

```
Show[

 plotWinvA,
 plotWinvB,
 plotYaStable,

 PlotRange → {{-1, 1}, {-1, 1}},
 ImageSize → {xsize, xsize},
 PlotRangePadding → 0,
 FrameTicks → {Table[{x, x, ticksize}, {x, -1, 1, 1}],
   Table[{y, y, ticksize}, {y, -1, 1, 1}], None, None},
 FrameTicksStyle → {{Directive[Black, Thickness[lwd]],
    Directive[Black, Thickness[lwd]]}, {Directive[Black, Thickness[lwd]],
    Directive[Black, Thickness[lwd], FontColor → White]}},
 FrameStyle → {{Black, Thickness[lwd]}, {Black, Thickness[lwd]},
   {Black, Thickness[lwd]}, {Black, Thickness[lwd]}},
 BaseStyle → {FontFamily → "Helvetica", FontSize → 14},
 ImagePadding → Pad,
 PlotRangeClipping → False
]
```

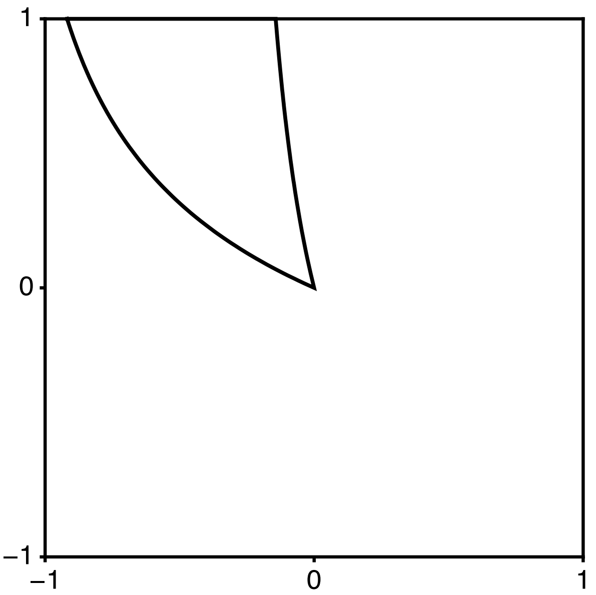

Panel B - invasion region with haploid competition in males

Parameters

```
params = {
    wAm → 1 + t, wam → 1, wAf → 1, waf → 1,
    αm → 1 / 2, αf → 1 / 2,
    Maa → 1, Faa → 1,
    MAa → 1 + (3 / 4) s, FAa → 1 + (3 / 4) s,
    MAA → 1 + s, FAA → 1 + s
  };
```

No recombination eigenvalues

```
WAinvA = λmA1 /. reverse /. pAveM → (1 - q) pXm + q pYm /. equilA0 /. params // Simplify;
WainvA = λma1 /. reverse /. pAveM → (1 - q) pXm + q pYm /. equilA0 /. params // Simplify;
WAinvB = λmA1 /. reverse /. pAveM → (1 - q) pXm + q pYm /. equilB0 /. params // Simplify;
WainvB = λma1 /. reverse /. pAveM → (1 - q) pXm + q pYm /. equilB0 /. params // Simplify;
```

Maximum absolute no recombination eigenvalue from the full characteristic polynomial

```
λWsolA = Max[Abs[λ /. Solve[
        0 == charpolyk1 /. r → 0 /. R → 0 /. ρ → 0 /. equilA0 /. params // Simplify, λ]]];
λWsolB = Max[Abs[λ /. Solve[0 == charpolyk1 /. r → 0 /. R → 0 /. ρ → 0 /. equilB0 /.
          params // Simplify, λ]]];
```

Plot

Region plots of invasion

```
(*neo-WA invades XY from equilA*)
plotWAinvA =
  RegionPlot[{
     (validcondA /. params) && (*valid*)
      (stabcondA /. Rf → 0 /. Rm → 0 /. equilA0 /. params) && (*internally stable*)
      1 < WAinvA (*invasion*)
    },
    {t, -1, 1}, {s, -1, 1},
    PlotStyle → {Gray, Opacity[0.5]},
    BoundaryStyle → None
  ];

(*neo-Wa invades XY from equilA*)
plotWainvA =
  RegionPlot[{
     (validcondA /. params) &&
      (stabcondA /. Rf → 0 /. Rm → 0 /. equilA0 /. params) &&
      1 < WainvA
    },
    {t, -1, 1}, {s, -1, 1},
    PlotStyle → {Gray, Opacity[0.5]},
    BoundaryStyle → None
  ];

(*neo-WA invades XY from equilB*)
```

```
plotWAinvB =
  RegionPlot[{
    (stabcondB /. Rf → 0 /. Rm → 0 /. equilB0 /. params) && (*internally stable*)
     1 < WAinvB(*invasion*)
   },
   {t, -1, 1}, {s, -1, 1},
   PlotStyle → {Gray, Opacity[0.5]},
   BoundaryStyle → None
  ];

(*neo-Wa invades XY from equilB*)
plotWainvB =
  RegionPlot[{
    (stabcondB /. Rf → 0 /. Rm → 0 /. equilB0 /. params) &&
     1 < WainvB
   },
   {t, -1, 1}, {s, -1, 1},
   PlotStyle → {Gray, Opacity[0.5]},
   BoundaryStyle → None
  ];

(*Ya equilibrium internally stable*)
plotYaStable =
  RegionPlot[{
    (stabcondA /. Rf → 0 /. Rm → 0 /. equilA0 /. params) ||
     (stabcondB /. Rf → 0 /. Rm → 0 /. equilB0 /. params)
   },
   {t, -1, 1}, {s, -1, 1},
   PlotStyle → White,
   BoundaryStyle → {Black, Thick}
  ];

(*Ya equilibrium internally unstable*)
plotYaUnstable =
  RegionPlot[{
    1 < 2
   },
   {αmd, -1, 1}, {s, -1, 1},
   PlotStyle → None,
   BoundaryStyle → None,
   Mesh → 50,
   MeshFunctions → {#1 - #2 &},
   MeshStyle → Black
  ];
```

```
plotB =
 Show[

  plotYaUnstable,
  plotYaStable,
  plotWAinvA,
  plotWainvA,
  plotWAinvB,
  plotWainvB,

  PlotRange → {{-1, 1}, {-1, 1}},
  ImageSize → {xsize, xsize},
  PlotRangePadding → 0,
  FrameTicks → {Table[{x, x, ticksize}, {x, -1, 1, 1}],
    Table[{y, y, ticksize}, {y, -1, 1, 1}], None, None},
  FrameTicksStyle → {{Directive[Black, Thickness[lwd]],
     Directive[Black, Thickness[lwd]]}, {Directive[Black, Thickness[lwd]],
     Directive[Black, Thickness[lwd], FontColor → White]}},
  FrameStyle → {{Black, Thickness[lwd]}, {Black, Thickness[lwd]},
    {Black, Thickness[lwd]}, {Black, Thickness[lwd]}},
  FrameLabel → {"Haploid competition for A in males", ""},
  BaseStyle → {FontFamily → "Helvetica", FontSize → 14},
  ImagePadding → Pad,
  Epilog → {
    Text[Style["B", 14, Bold], Scaled@{0.95, 0.95}],
    Text[Style["Λ_{W'A}^(XY) >1
&
  Λ_{W'a}^(XY) <1", 14], {-0.4, 0.6}],
    Text[Style["*", 16, Bold], {-0.1, 0.1}]
   },
  PlotLabel → Style["haploid competition in males", 16, Black, Bold],
  PlotRangeClipping → False
 ]
```

haploid competition in males

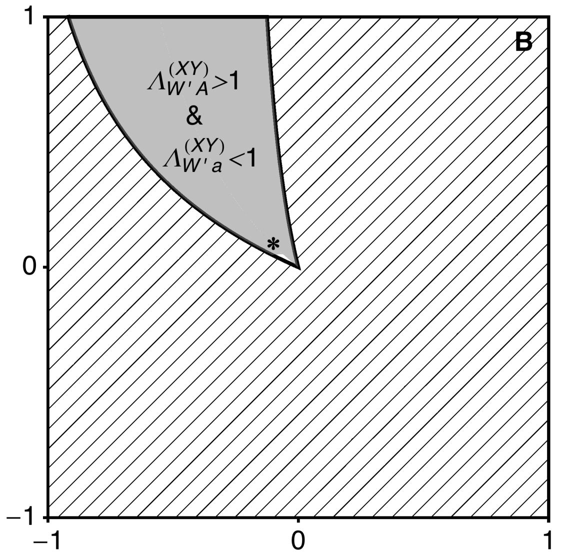

Haploid competition for *A* in males

Notice that this is consistent with the solution from the full characteristic polynomial (but here we don’t know which eigenvalue belongs to which haplotype)

```
(*neo-W invades XY from equilA*)
plotWinvA =
  RegionPlot[{
    (validcondA /. params) && (*valid*)
     (stabcondA /. Rf → 0 /. Rm → 0 /. equilA0 /. params) && (*internally stable*)
     1 < λWsolA(*invasion*)
   },
   {t, -1, 1}, {s, -1, 1},
   PlotStyle → {Gray, Opacity[0.5]},
   BoundaryStyle → None
  ];

(*neo-W invades XY from equilB*)
plotWinvB =
  RegionPlot[{
    (stabcondB /. Rf → 0 /. Rm → 0 /. equilB0 /. params) && (*internally stable*)
     1 < λWsolB(*invasion*)
   },
   {t, -1, 1}, {s, -1, 1},
   PlotStyle → {Gray, Opacity[0.5]},
   BoundaryStyle → None
  ];
```

```
Show[

 plotWinvA,
 plotWinvB,
 plotYaStable,

 PlotRange → {{-1, 1}, {-1, 1}},
 ImageSize → {xsize, xsize},
 PlotRangePadding → 0,
 FrameTicks → {Table[{x, x, ticksize}, {x, -1, 1, 1}],
   Table[{y, y, ticksize}, {y, -1, 1, 1}], None, None},
 FrameTicksStyle → {{Directive[Black, Thickness[lwd]],
    Directive[Black, Thickness[lwd]]}, {Directive[Black, Thickness[lwd]],
    Directive[Black, Thickness[lwd], FontColor → White]}},
 FrameStyle → {{Black, Thickness[lwd]}, {Black, Thickness[lwd]},
   {Black, Thickness[lwd]}, {Black, Thickness[lwd]}},
 BaseStyle → {FontFamily → "Helvetica", FontSize → 14},
 ImagePadding → Pad,
 PlotRangeClipping → False
]
```

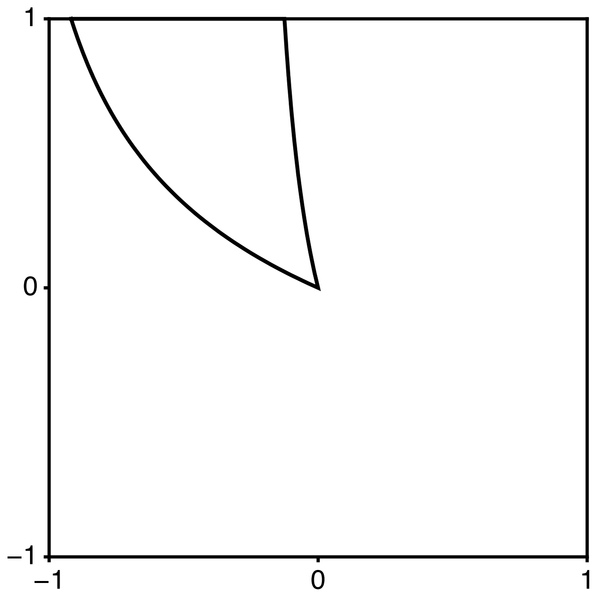

Panel C - invasion region with drive in females

Parameters

```
params = {
   wAm → 1, wam → 1, wAf → 1, waf → 1,
   αm → 1 / 2, αf → (1 + αmd) / 2,
   Maa → 1, Faa → 1,
   MAa → 1 + (3 / 4) s, FAa → 1 + (3 / 4) s,
   MAA → 1 + s, FAA → 1 + s
  };
```

No recombination eigenvalues

```
WAinvA = λmA1 /. reverse /. pAveM → (1 - q) pXm + q pYm /. equilA0 /. params // Simplify;
WainvA = λma1 /. reverse /. pAveM → (1 - q) pXm + q pYm /. equilA0 /. params // Simplify;
WAinvB = λmA1 /. reverse /. pAveM → (1 - q) pXm + q pYm /. equilB0 /. params // Simplify;
WainvB = λma1 /. reverse /. pAveM → (1 - q) pXm + q pYm /. equilB0 /. params // Simplify;
```

Maximum absolute no recombination eigenvalue from the full characteristic polynomial

```
λWsolA = Max[Abs[λ /. Solve[
      0 == charpolyk1 /. r → 0 /. R → 0 /. ρ → 0 /. equilA0 /. params // Simplify, λ]]];
λWsolB = Max[Abs[λ /. Solve[0 == charpolyk1 /. r → 0 /. R → 0 /. ρ → 0 /. equilB0 /.
        params // Simplify, λ]]];
```

Plot

Region plots of invasion

```
(*neo-WA invades XY from equilA*)
plotWAinvA =
  RegionPlot[{
    (validcondA /. params) && (*valid*)
     (stabcondA /. Rf → 0 /. Rm → 0 /. equilA0 /. params) && (*internally stable*)
     1 < WAinvA (*invasion*)
   },
   {αmd, -1, 1}, {s, -1, 1},
   PlotStyle → {Gray, Opacity[0.5]},
   BoundaryStyle → None
  ];

(*neo-Wa invades XY from equilA*)
plotWainvA =
  RegionPlot[{
    (validcondA /. params) &&
     (stabcondA /. Rf → 0 /. Rm → 0 /. equilA0 /. params) &&
     1 < WainvA
   },
   {αmd, -1, 1}, {s, -1, 1},
   PlotStyle → {Gray, Opacity[0.5]},
   BoundaryStyle → None
  ];

(*neo-WA invades XY from equilB*)
```

```
plotWAinvB =
  RegionPlot[{
    (stabcondB /. Rf → 0 /. Rm → 0 /. equilB0 /. params) && (*internally stable*)
     1 < WAinvB(*invasion*)
   },
   {αmd, -1, 1}, {s, -1, 1},
   PlotStyle → {Gray, Opacity[0.5]},
   BoundaryStyle → None
  ];

(*neo-Wa invades XY from equilB*)
plotWainvB =
  RegionPlot[{
    (stabcondB /. Rf → 0 /. Rm → 0 /. equilB0 /. params) &&
     1 < WainvB
   },
   {αmd, -1, 1}, {s, -1, 1},
   PlotStyle → {Gray, Opacity[0.5]},
   BoundaryStyle → None
  ];

(*Ya equilibrium internally stable*)
plotYaStable =
  RegionPlot[{
    (stabcondA /. Rf → 0 /. Rm → 0 /. equilA0 /. params) ||
     (stabcondB /. Rf → 0 /. Rm → 0 /. equilB0 /. params)
   },
   {αmd, -1, 1}, {s, -1, 1},
   PlotStyle → White,
   BoundaryStyle → {Black, Thick}
  ];

(*Ya equilibrium internally unstable*)
plotYaUnstable =
  RegionPlot[{
    1 < 2
   },
   {αmd, -1, 1}, {s, -1, 1},
   PlotStyle → None,
   BoundaryStyle → None,
   Mesh → 50,
   MeshFunctions → {#1 - #2 &},
   MeshStyle → Black
  ];
```

```
plotC =
 Show[

  plotYaUnstable,
  plotYaStable,
  plotWAinvA,
  plotWainvA,
  plotWAinvB,
  plotWainvB,

  Graphics[{Thick, Black, Line[{{0.5, -0.1}, {0.5, 0.1}}]}],
  Graphics[{Thick, Black, Line[{{0.5, -0.5}, {0.4, -0.7}}]}],
  PlotRange → {{-1, 1}, {-1, 1}},
  ImageSize → {xsize, xsize},
  PlotRangePadding → 0,
  FrameTicks → {Table[{x, x, ticksize}, {x, -1, 1, 1}],
    Table[{y, y, ticksize}, {y, -1, 1, 1}], None, None},
  FrameTicksStyle → {{Directive[Black, Thickness[lwd]],
     Directive[Black, Thickness[lwd]]}, {Directive[Black, Thickness[lwd]],
     Directive[Black, Thickness[lwd], FontColor → White]}},
  FrameStyle → {{Black, Thickness[lwd]}, {Black, Thickness[lwd]},
    {Black, Thickness[lwd]}, {Black, Thickness[lwd]}},
  FrameLabel → {"Drive for A in females", ""},
  BaseStyle → {FontFamily → "Helvetica", FontSize → 14},
  ImagePadding → Pad,
  Epilog → {
    Text[Style["C", 14, Bold], Scaled@{0.95, 0.95}],
    Text[Style["Λ_{W'A}^(XY) >1
&
Λ_{W'a}^(XY) <1", 14], {0.5, 0.3}, Background → White],
    Text[Style["Λ_{W'A}^(XY) >1
&
Λ_{W'a}^(XY) >1", 14], {0.15, -0.75}, Background → White],
    Text[Style["*", 16, Bold], {0.2, -0.1}]
   },
  PlotLabel → Style["drive in females", 16, Black, Bold],
  PlotRangeClipping → False
 ]
```

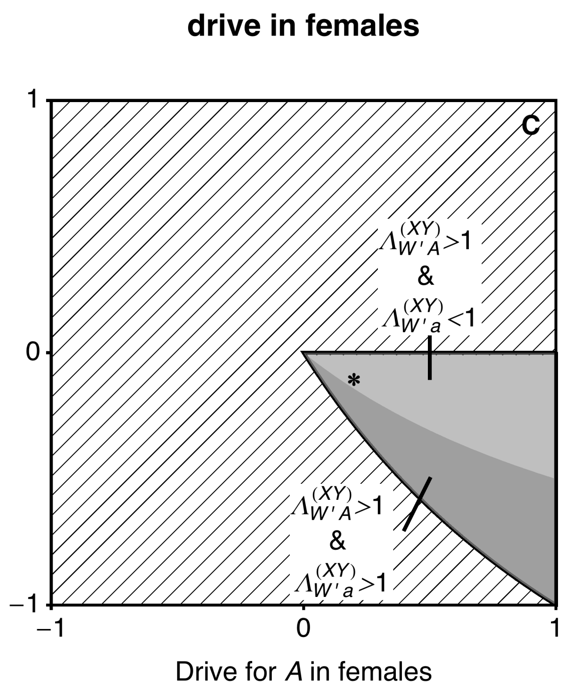

Notice that this is consistent with the solution from the full characteristic polynomial (but here we don't know which eigenvalue belongs to which haplotype)

```
(*neo-W invades XY from equilA*)
plotWinvA =
  RegionPlot[{
    (validcondA /. params) && (*valid*)
     (stabcondA /. Rf → 0 /. Rm → 0 /. equilA0 /. params) && (*internally stable*)
     1 < λWsolA(*invasion*)
   },
   {αmd, -1, 1}, {s, -1, 1},
   PlotStyle → {Gray, Opacity[0.5]},
   BoundaryStyle → None
  ];

(*neo-W invades XY from equilB*)
plotWinvB =
  RegionPlot[{
    (stabcondB /. Rf → 0 /. Rm → 0 /. equilB0 /. params) && (*internally stable*)
     1 < λWsolB(*invasion*)
   },
   {αmd, -1, 1}, {s, -1, 1},
   PlotStyle → {Gray, Opacity[0.5]},
   BoundaryStyle → None
  ];
```

```
Show[

 plotWinvA,
 plotWinvB,
 plotYaStable,

 PlotRange → {{-1, 1}, {-1, 1}},
 ImageSize → {xsize, xsize},
 PlotRangePadding → 0,
 FrameTicks → {Table[{x, x, ticksize}, {x, -1, 1, 1}],
   Table[{y, y, ticksize}, {y, -1, 1, 1}], None, None},
 FrameTicksStyle → {{Directive[Black, Thickness[lwd]],
    Directive[Black, Thickness[lwd]]}, {Directive[Black, Thickness[lwd]],
    Directive[Black, Thickness[lwd], FontColor → White]}},
 FrameStyle → {{Black, Thickness[lwd]}, {Black, Thickness[lwd]},
   {Black, Thickness[lwd]}, {Black, Thickness[lwd]}},
 BaseStyle → {FontFamily → "Helvetica", FontSize → 14},
 ImagePadding → Pad,
 PlotRangeClipping → False
]
```

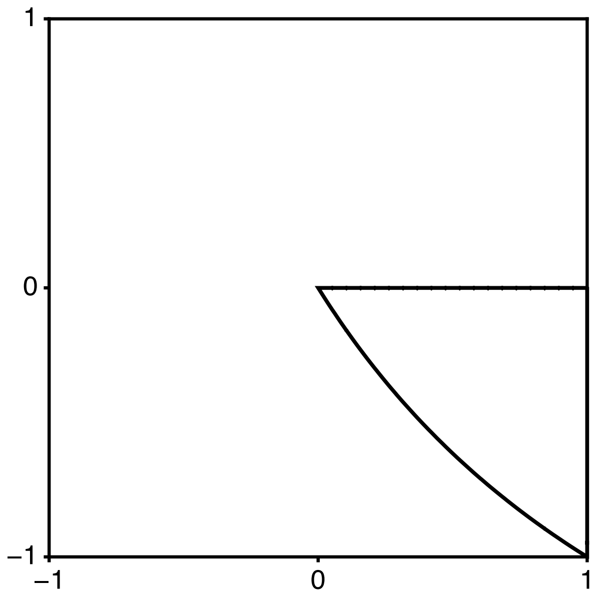

Panel D - invasion region with haploid competition in females

Parameters

```
params = {
    wAm → 1, wam → 1, wAf → 1 + t, waf → 1,
    αm → 1 / 2, αf → 1 / 2,
    Maa → 1, Faa → 1,
    MAa → 1 + (3 / 4) s, FAa → 1 + (3 / 4) s,
    MAA → 1 + s, FAA → 1 + s
   };
```

No recombination eigenvalues

```
WAinvA = λmA1 /. reverse /. pAveM → (1 - q) pXm + q pYm /. equilA0 /. params // Simplify;
WainvA = λma1 /. reverse /. pAveM → (1 - q) pXm + q pYm /. equilA0 /. params // Simplify;
WAinvB = λmA1 /. reverse /. pAveM → (1 - q) pXm + q pYm /. equilB0 /. params // Simplify;
WainvB = λma1 /. reverse /. pAveM → (1 - q) pXm + q pYm /. equilB0 /. params // Simplify;
```

Maximum absolute no recombination eigenvalue from the full characteristic polynomial

```
λWsolA = Max[Abs[λ /. Solve[
        0 == charpolyk1 /. r → 0 /. R → 0 /. ρ → 0 /. equilA0 /. params // Simplify, λ]]];
λWsolB = Max[Abs[λ /. Solve[0 == charpolyk1 /. r → 0 /. R → 0 /. ρ → 0 /. equilB0 /.
          params // Simplify, λ]]];
```

Plot

Region plots of invasion

```
(*neo-WA invades XY from equilA*)
plotWAinvA =
  RegionPlot[{
     (validcondA /. params) && (*valid*)
      (stabcondA /. Rf → 0 /. Rm → 0 /. equilA0 /. params) && (*internally stable*)
      1 < WAinvA (*invasion*)
    },
    {t, -1, 1}, {s, -1, 1},
    PlotStyle → {Gray, Opacity[0.5]},
    BoundaryStyle → None
   ];

(*neo-Wa invades XY from equilA*)
plotWainvA =
  RegionPlot[{
     (validcondA /. params) &&
      (stabcondA /. Rf → 0 /. Rm → 0 /. equilA0 /. params) &&
      1 < WainvA
    },
    {t, -1, 1}, {s, -1, 1},
    PlotStyle → {Gray, Opacity[0.5]},
    BoundaryStyle → None
   ];

(*neo-WA invades XY from equilB*)
```

```
plotWAinvB =
  RegionPlot[{
    (stabcondB /. Rf → 0 /. Rm → 0 /. equilB0 /. params) && (*internally stable*)
     1 < WAinvB(*invasion*)
   },
   {t, -1, 1}, {s, -1, 1},
   PlotStyle → {Gray, Opacity[0.5]},
   BoundaryStyle → None
  ];

(*neo-Wa invades XY from equilB*)
plotWainvB =
  RegionPlot[{
    (stabcondB /. Rf → 0 /. Rm → 0 /. equilB0 /. params) &&
     1 < WainvB
   },
   {t, -1, 1}, {s, -1, 1},
   PlotStyle → {Gray, Opacity[0.5]},
   BoundaryStyle → None
  ];

(*Ya equilibrium internally stable*)
plotYaStable =
  RegionPlot[{
    (stabcondA /. Rf → 0 /. Rm → 0 /. equilA0 /. params) ||
     (stabcondB /. Rf → 0 /. Rm → 0 /. equilB0 /. params)
   },
   {t, -1, 1}, {s, -1, 1},
   PlotStyle → White,
   BoundaryStyle → {Black, Thick}
  ];

(*Ya equilibrium internally unstable*)
plotYaUnstable =
  RegionPlot[{
    1 < 2
   },
   {αmd, -1, 1}, {s, -1, 1},
   PlotStyle → None,
   BoundaryStyle → None,
   Mesh → 50,
   MeshFunctions → {#1 - #2 &},
   MeshStyle → Black
  ];
```

```
plotD =
 Show[

  plotYaUnstable,
  plotYaStable,
  plotWAinvA,
  plotWainvA,
  plotWAinvB,
  plotWainvB,

  PlotRange → {{-1, 1}, {-1, 1}},
  ImageSize → {xsize, xsize},
  PlotRangePadding → 0,
  FrameTicks → {Table[{x, x, ticksize}, {x, -1, 1, 1}],
    Table[{y, y, ticksize}, {y, -1, 1, 1}], None, None},
  FrameTicksStyle → {{Directive[Black, Thickness[lwd]],
     Directive[Black, Thickness[lwd]]}, {Directive[Black, Thickness[lwd]],
     Directive[Black, Thickness[lwd], FontColor → White]}},
  FrameStyle → {{Black, Thickness[lwd]}, {Black, Thickness[lwd]},
    {Black, Thickness[lwd]}, {Black, Thickness[lwd]}},
  FrameLabel → {"Haploid competition for A in females", ""},
  BaseStyle → {FontFamily → "Helvetica", FontSize → 14},
  ImagePadding → Pad,
  Epilog → {
    Text[Style["D", 14, Bold], Scaled@{0.95, 0.95}],
    Text[Style["Λ_{W'A}^{(XY)} >1
&
Λ_{W'a}^{(XY)} <1", 14], {0.75, -0.25}],
    Text[Style["*", 16, Bold], {0.1, -0.05}]
   },
  PlotLabel → Style["haploid competition in females", 16, Black, Bold],
  PlotRangeClipping → False
 ]
```

haploid competition in females

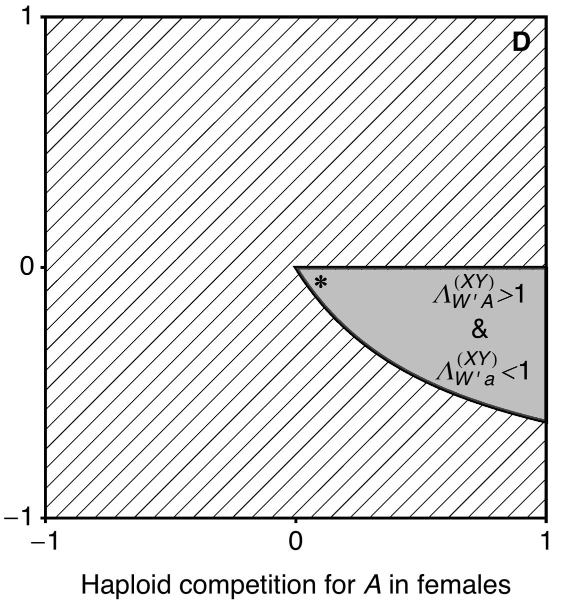

Notice that this is consistent with the solution from the full characteristic polynomial (but here we don't know which eigenvalue belongs to which haplotype)

```
(*neo-W invades XY from equilA*)
plotWinvA =
  RegionPlot[{
    (validcondA /. params) && (*valid*)
     (stabcondA /. Rf → 0 /. Rm → 0 /. equilA0 /. params) && (*internally stable*)
     1 < λWsolA(*invasion*)
   },
   {t, -1, 1}, {s, -1, 1},
   PlotStyle → {Gray, Opacity[0.5]},
   BoundaryStyle → None
  ];

(*neo-W invades XY from equilB*)
plotWinvB =
  RegionPlot[{
    (stabcondB /. Rf → 0 /. Rm → 0 /. equilB0 /. params) && (*internally stable*)
     1 < λWsolB(*invasion*)
   },
   {t, -1, 1}, {s, -1, 1},
   PlotStyle → {Gray, Opacity[0.5]},
   BoundaryStyle → None
  ];
```

```
Show[

 plotWinvA,
 plotWinvB,
 plotYaStable,

 PlotRange → {{-1, 1}, {-1, 1}},
 ImageSize → {xsize, xsize},
 PlotRangePadding → 0,
 FrameTicks → {Table[{x, x, ticksize}, {x, -1, 1, 1}],
   Table[{y, y, ticksize}, {y, -1, 1, 1}], None, None},
 FrameTicksStyle → {{Directive[Black, Thickness[lwd]],
    Directive[Black, Thickness[lwd]]}, {Directive[Black, Thickness[lwd]],
    Directive[Black, Thickness[lwd], FontColor → White]}},
 FrameStyle → {{Black, Thickness[lwd]}, {Black, Thickness[lwd]},
   {Black, Thickness[lwd]}, {Black, Thickness[lwd]}},
 BaseStyle → {FontFamily → "Helvetica", FontSize → 14},
 ImagePadding → Pad,
 PlotRangeClipping → False
]
```

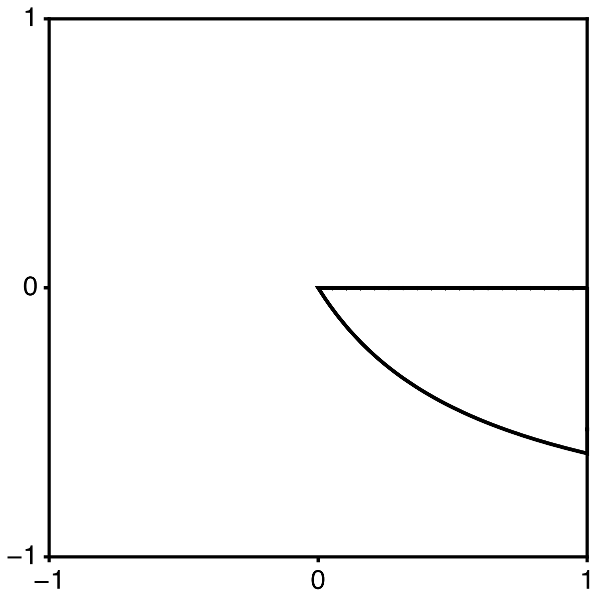

Panel E - temporal invasion dynamic with meiotic drive in males

Parameters

```
params = {
   wAm → 1, wam → 1, wAf → 1, waf → 1,
   αm → (1 + αmd) / 2, αf → 1 / 2,
   Maa → 1, Faa → 1,
   MAa → 1 + (3 / 4) s, FAa → 1 + (3 / 4) s,
   MAA → 1 + s, FAA → 1 + s
  };
```

```
moreparams = {
   s → 1 / 4,
   αmd → - 1 / 10
  };
```

```
tryFAA = FAA /. params /. moreparams;
tryFAa = FAa /. params /. moreparams;
tryFaa = Faa /. params /. moreparams;
tryMAA = MAA /. params /. moreparams;
tryMAa = MAa /. params /. moreparams;
tryMaa = Maa /. params /. moreparams;
trywAf = wAf /. params /. moreparams;
trywaf = waf /. params /. moreparams;
trywAm = wAm /. params /. moreparams;
trywam = wam /. params /. moreparams;
tryαf = αf /. params /. moreparams;
tryαm = αm /. params /. moreparams;
```

Plots

```
tryR = 0.001;
param = {tryFAA, tryFAa, tryFaa, tryMAA, tryMAa, tryMaa, trywAf, trywaf, trywAm,
   trywam, tryαf, tryαm, tryr, tryR, tryr (1 - tryR) + tryR (1 - tryr), tryk};
run[0] = generation[param, startgen[sieveXY[tryFAA, tryFAa, tryFaa, tryMAA, tryMAa,
       tryMaa, trywAf, trywaf, trywAm, trywam, tryαf, tryαm, tryr][[1]], trypm]];
For[time = 1, time ≤ endtime, time++, run[time] = generation[param, run[time - 1]]
]
MODtab1Black =
  Table[{Round[Exp[exptime]], generation[param, run[Round[Exp[exptime]]]].modvec},
   {exptime, Log[startplot], Log[endtime], 0.1}];
MOD1Black = ListLogLinearPlot[MODtab1Black, Joined → True,
  PlotRange → {{startplot, endtime}, {0, 0.55}},
  PlotStyle → {Black, Thick}, loglinearplotINVoptions]
```

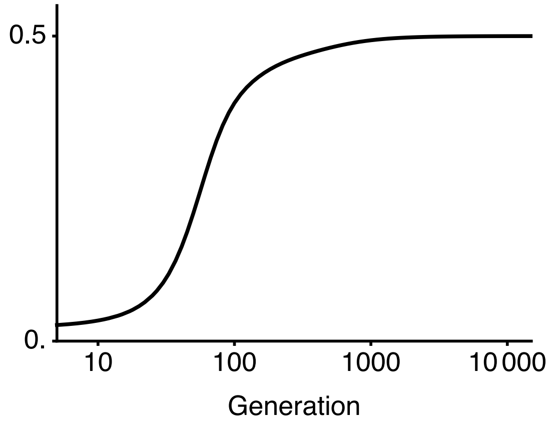

```
tryR = 0.02;
param = {tryFAA, tryFAa, tryFaa, tryMAA, tryMAa, tryMaa, trywAf, trywaf, trywAm,
   trywam, tryαf, tryαm, tryr, tryR, tryr (1 - tryR) + tryR (1 - tryr), tryk};
run[0] = generation[param, startgen[sieveXY[tryFAA, tryFAa, tryFaa, tryMAA, tryMAa,
     tryMaa, trywAf, trywaf, trywAm, trywam, tryαf, tryαm, tryr][[1]], trypm]];
For[time = 1, time ≤ endtime, time++, run[time] = generation[param, run[time - 1]]
]
MODtab1Red =
  Table[{Round[Exp[exptime]], generation[param, run[Round[Exp[exptime]]]].modvec},
   {exptime, Log[startplot], Log[endtime], 0.1}];
MOD1Red = ListLogLinearPlot[MODtab1Red, Joined → True,
  PlotRange → {{startplot, endtime}, {0, 0.55}},
  PlotStyle → {Red, Thick}, loglinearplotINVoptions]
```

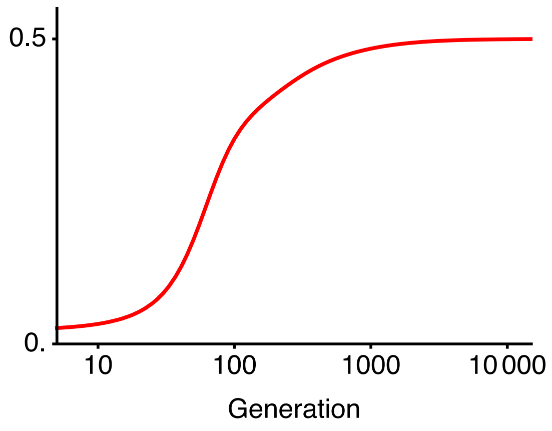

```
tryR = 0.1;
param = {tryFAA, tryFAa, tryFaa, tryMAA, tryMAa, tryMaa, trywAf, trywaf, trywAm,
   trywam, tryαf, tryαm, tryr, tryR, tryr (1 - tryR) + tryR (1 - tryr), tryk};
run[0] = generation[param, startgen[sieveXY[tryFAA, tryFAa, tryFaa, tryMAA, tryMAa,
       tryMaa, trywAf, trywaf, trywAm, trywam, tryαf, tryαm, tryr][[1]], trypm]];
For[time = 1, time ≤ endtime, time++, run[time] = generation[param, run[time - 1]]
]
MODtab1Blue =
  Table[{Round[Exp[exptime]], generation[param, run[Round[Exp[exptime]]]].modvec},
   {exptime, Log[startplot], Log[endtime], 0.1}];
MOD1Blue = ListLogLinearPlot[MODtab1Blue, Joined → True,
  PlotRange → {{startplot, endtime}, {0, 0.55}},
  PlotStyle → {Blue, Thick}, loglinearplotINVoptions]
```

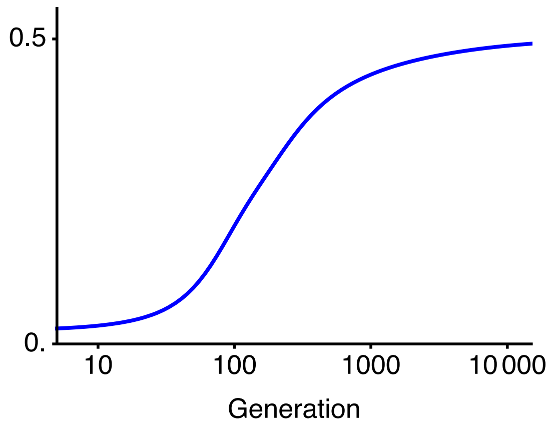

```
tryR = 0.5;
param = {tryFAA, tryFAa, tryFaa, tryMAA, tryMAa, tryMaa, trywAf, trywaf, trywAm,
   trywam, tryαf, tryαm, tryr, tryR, tryr (1 - tryR) + tryR (1 - tryr), tryk};
run[0] = generation[param, startgen[sieveXY[tryFAA, tryFAa, tryFaa, tryMAA, tryMAa,
       tryMaa, trywAf, trywaf, trywAm, trywam, tryαf, tryαm, tryr][[1]], trypm]];
For[time = 1, time ≤ endtime, time++, run[time] = generation[param, run[time - 1]]
]
MODtab1Green =
  Table[{Round[Exp[exptime]], generation[param, run[Round[Exp[exptime]]]].modvec},
   {exptime, Log[startplot], Log[endtime], 0.1}];
MOD1Green = ListLogLinearPlot[MODtab1Green, Joined → True,
  PlotRange → {{startplot, endtime}, {0, 0.55}},
  PlotStyle → {Green, Thick}, loglinearplotINVoptions]
```

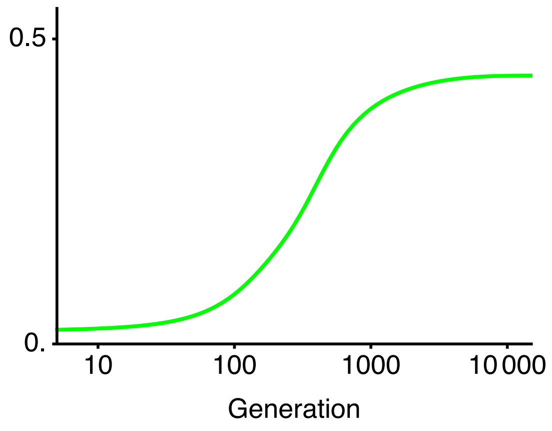


```
plotE = Show[MOD1Black, MOD1Red, MOD1Blue, MOD1Green,
  LogLinearPlot[0.5, {x, 5, endtime}, PlotStyle → {Black, Dashed}],
  Epilog → {
    Rotate[Text[Style["neo-W frequency", 14], Scaled@ylabpos], 90 Degree],
    Text[Style["E", 14, Bold], Scaled@{0.95, 0.95}]
   }
  , PlotRangeClipping → False]
```

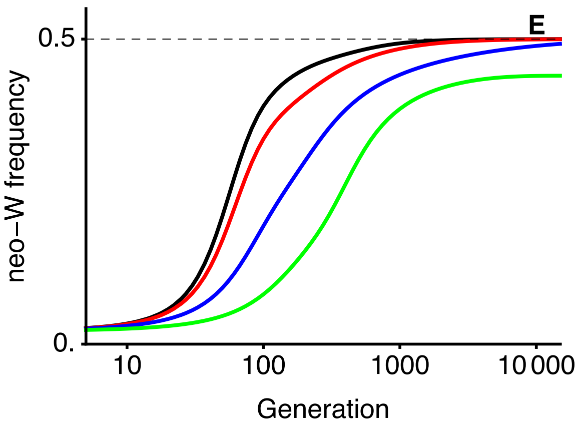

Panel F - temporal invasion dynamic with haploid competition in males

Parameters

```
params = {
   wAm → 1 + t, wam → 1, wAf → 1, waf → 1,
   αm → 1 / 2, αf → 1 / 2,
   Maa → 1, Faa → 1,
   MAa → 1 + (3 / 4) s, FAa → 1 + (3 / 4) s,
   MAA → 1 + s, FAA → 1 + s
  };
```

```
moreparams = {
   s → 1 / 10,
   t → -1 / 10
  };
```

```
tryFAA = FAA /. params /. moreparams;
tryFAa = FAa /. params /. moreparams;
tryFaa = Faa /. params /. moreparams;
tryMAA = MAA /. params /. moreparams;
tryMAa = MAa /. params /. moreparams;
tryMaa = Maa /. params /. moreparams;
trywAf = wAf /. params /. moreparams;
trywaf = waf /. params /. moreparams;
trywAm = wAm /. params /. moreparams;
trywam = wam /. params /. moreparams;
tryαf = αf /. params /. moreparams;
tryαm = αm /. params /. moreparams;
```

Plots

```
tryR = 0.001;
param = {tryFAA, tryFAa, tryFaa, tryMAA, tryMAa, tryMaa, trywAf, trywaf, trywAm,
   trywam, tryαf, tryαm, tryr, tryR, tryr (1 - tryR) + tryR (1 - tryr), tryk};
run[0] = generation[param, startgen[sieveXY[tryFAA, tryFAa, tryFaa, tryMAA, tryMAa,
      tryMaa, trywAf, trywaf, trywAm, trywam, tryαf, tryαm, tryr][[1]], trypm]];
For[time = 1, time ≤ endtime, time++, run[time] = generation[param, run[time - 1]]
]
MODtab1Black =
  Table[{Round[Exp[exptime]], generation[param, run[Round[Exp[exptime]]]].modvec},
   {exptime, Log[startplot], Log[endtime], 0.1}];
MOD1Black = ListLogLinearPlot[MODtab1Black, Joined → True,
  PlotRange → {{startplot, endtime}, {0, 0.55}},
  PlotStyle → {Black, Thick}, loglinearplotINVoptions]
```

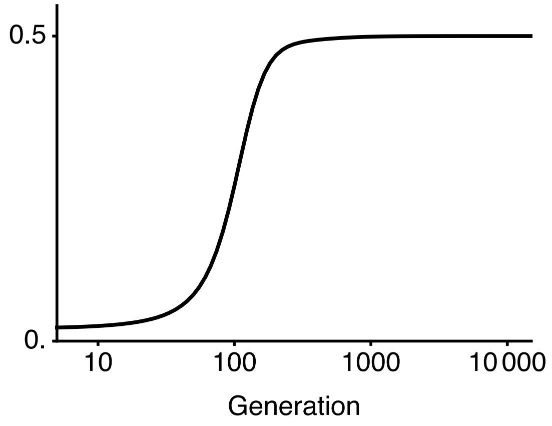

```
tryR = 0.02;
param = {tryFAA, tryFAa, tryFaa, tryMAA, tryMAa, tryMaa, trywAf, trywaf, trywAm,
   trywam, tryαf, tryαm, tryr, tryR, tryr (1 - tryR) + tryR (1 - tryr), tryk};
run[0] = generation[param, startgen[sieveXY[tryFAA, tryFAa, tryFaa, tryMAA, tryMAa,
      tryMaa, trywAf, trywaf, trywAm, trywam, tryαf, tryαm, tryr][[1]], trypm]];
For[time = 1, time ≤ endtime, time++, run[time] = generation[param, run[time - 1]]
]
MODtab1Red =
  Table[{Round[Exp[exptime]], generation[param, run[Round[Exp[exptime]]]].modvec},
   {exptime, Log[startplot], Log[endtime], 0.1}];
MOD1Red = ListLogLinearPlot[MODtab1Red, Joined → True,
  PlotRange → {{startplot, endtime}, {0, 0.55}},
  PlotStyle → {Red, Thick}, loglinearplotINVoptions]
```

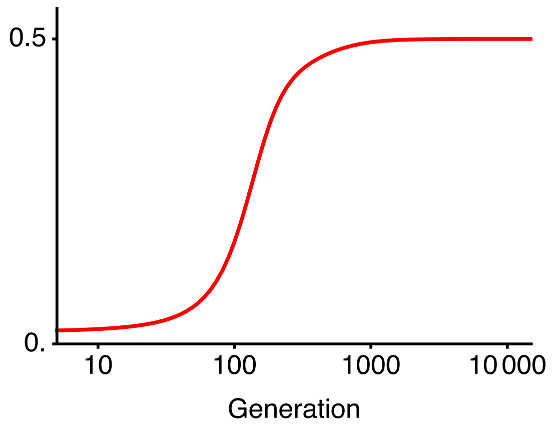

```
tryR = 0.1;
param = {tryFAA, tryFAa, tryFaa, tryMAA, tryMAa, tryMaa, trywAf, trywaf, trywAm,
   trywam, tryαf, tryαm, tryr, tryR, tryr (1 - tryR) + tryR (1 - tryr), tryk};
run[0] = generation[param, startgen[sieveXY[tryFAA, tryFAa, tryFaa, tryMAA, tryMAa,
      tryMaa, trywAf, trywaf, trywAm, trywam, tryαf, tryαm, tryr][[1]], trypm]];
For[time = 1, time ≤ endtime, time++, run[time] = generation[param, run[time - 1]]
]
MODtab1Blue =
  Table[{Round[Exp[exptime]], generation[param, run[Round[Exp[exptime]]]].modvec},
   {exptime, Log[startplot], Log[endtime], 0.1}];
MOD1Blue = ListLogLinearPlot[MODtab1Blue, Joined → True,
  PlotRange → {{startplot, endtime}, {0, 0.55}},
  PlotStyle → {Blue, Thick}, loglinearplotINVoptions]
```

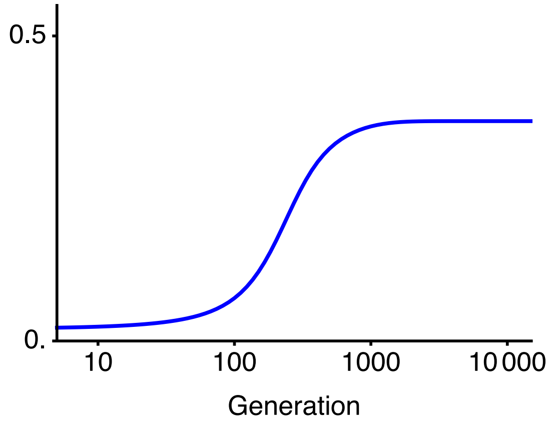

```
tryR = 0.5;
param = {tryFAA, tryFAa, tryFaa, tryMAA, tryMAa, tryMaa, trywAf, trywaf, trywAm,
    trywam, tryαf, tryαm, tryr, tryR, tryr (1 - tryR) + tryR (1 - tryr), tryk};
run[0] = generation[param, startgen[sieveXY[tryFAA, tryFAa, tryFaa, tryMAA, tryMAa,
       tryMaa, trywAf, trywaf, trywAm, trywam, tryαf, tryαm, tryr][[1]], trypm]];
For[time = 1, time ≤ endtime, time++, run[time] = generation[param, run[time - 1]]
]
MODtab1Green =
  Table[{Round[Exp[exptime]], generation[param, run[Round[Exp[exptime]]]].modvec},
   {exptime, Log[startplot], Log[endtime], 0.1}];
MOD1Green = ListLogLinearPlot[MODtab1Green, Joined → True,
  PlotRange → {{startplot, endtime}, {0, 0.55}},
  PlotStyle → {Green, Thick}, loglinearplotINVoptions]
```

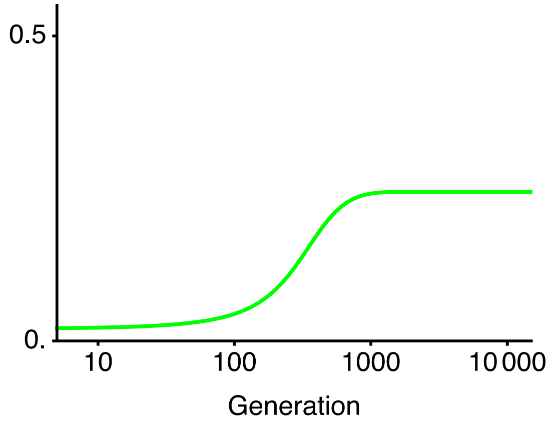


```
plotF = Show[MOD1Black, MOD1Red, MOD1Blue, MOD1Green,
  LogLinearPlot[0.5, {x, 5, endtime}, PlotStyle → {Black, Dashed}],
  Epilog → {
    Text[Style["F", 14, Bold], Scaled@{0.95, 0.95}]
   }
  , PlotRangeClipping → False]
```

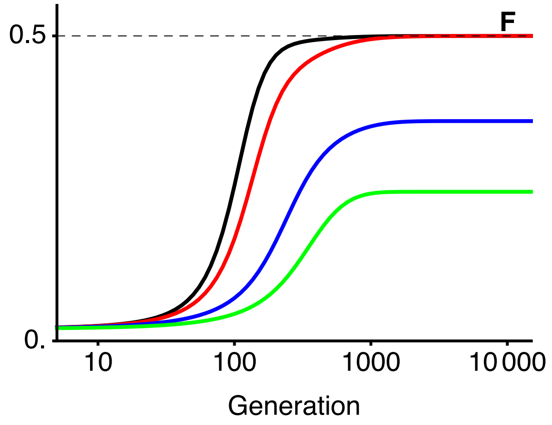

Panel G - temporal invasion dynamic with meiotic drive in females

Parameters

```
params = {
   wAm → 1, wam → 1, wAf → 1, waf → 1,
   αm → 1 / 2, αf → (1 + αmd) / 2,
   Maa → 1, Faa → 1,
   MAa → 1 + (3 / 4) s, FAa → 1 + (3 / 4) s,
   MAA → 1 + s, FAA → 1 + s
  };
```

```
moreparams = {
   s → - 1 / 10,
   αmd → 1 / 5
  };
```

```
tryFAA = FAA /. params /. moreparams;
tryFAa = FAa /. params /. moreparams;
tryFaa = Faa /. params /. moreparams;
tryMAA = MAA /. params /. moreparams;
tryMAa = MAa /. params /. moreparams;
tryMaa = Maa /. params /. moreparams;
trywAf = wAf /. params /. moreparams;
trywaf = waf /. params /. moreparams;
trywAm = wAm /. params /. moreparams;
trywam = wam /. params /. moreparams;
tryαf = αf /. params /. moreparams;
tryαm = αm /. params /. moreparams;
```

Plots

```
tryR = 0.001;
param = {tryFAA, tryFAa, tryFaa, tryMAA, tryMAa, tryMaa, trywAf, trywaf, trywAm,
   trywam, tryαf, tryαm, tryr, tryR, tryr (1 - tryR) + tryR (1 - tryr), tryk};
run[0] = generation[param, startgen[sieveXY[tryFAA, tryFAa, tryFaa, tryMAA, tryMAa,
      tryMaa, trywAf, trywaf, trywAm, trywam, tryαf, tryαm, tryr][[1]], trypm]];
For[time = 1, time ≤ endtime, time++, run[time] = generation[param, run[time - 1]]
]
MODtab1Black =
  Table[{Round[Exp[exptime]], generation[param, run[Round[Exp[exptime]]]].modvec},
   {exptime, Log[startplot], Log[endtime], 0.1}];
MOD1Black = ListLogLinearPlot[MODtab1Black, Joined → True,
  PlotRange → {{startplot, endtime}, {0, 0.55}},
  PlotStyle → {Black, Thick}, loglinearplotINVoptions]
```

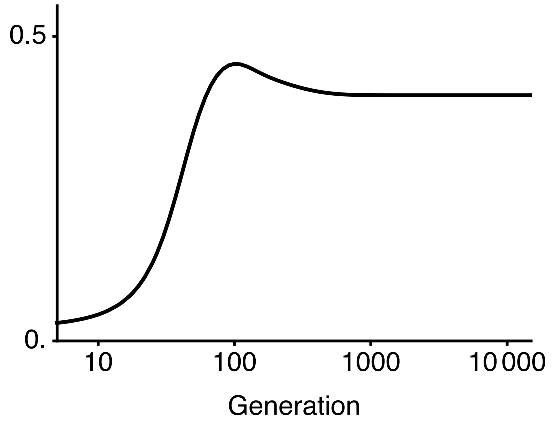

```
tryR = 0.02;
param = {tryFAA, tryFAa, tryFaa, tryMAA, tryMAa, tryMaa, trywAf, trywaf, trywAm,
   trywam, tryαf, tryαm, tryr, tryR, tryr (1 - tryR) + tryR (1 - tryr), tryk};
run[0] = generation[param, startgen[sieveXY[tryFAA, tryFAa, tryFaa, tryMAA, tryMAa,
      tryMaa, trywAf, trywaf, trywAm, trywam, tryαf, tryαm, tryr][[1]], trypm]];
For[time = 1, time ≤ endtime, time++, run[time] = generation[param, run[time - 1]]
]
MODtab1Red =
  Table[{Round[Exp[exptime]], generation[param, run[Round[Exp[exptime]]]].modvec},
   {exptime, Log[startplot], Log[endtime], 0.1}];
MOD1Red = ListLogLinearPlot[MODtab1Red, Joined → True,
  PlotRange → {{startplot, endtime}, {0, 0.75}},
  PlotStyle → {Red, Thick}, loglinearplotINVoptions]
```

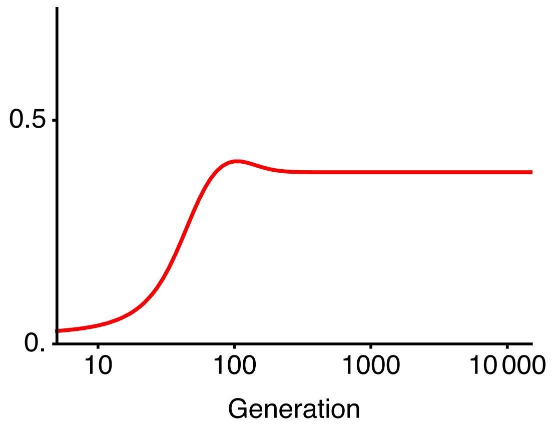

```
tryR = 0.1;
param = {tryFAA, tryFAa, tryFaa, tryMAA, tryMAa, tryMaa, trywAf, trywaf, trywAm,
   trywam, tryαf, tryαm, tryr, tryR, tryr (1 - tryR) + tryR (1 - tryr), tryk};
run[0] = generation[param, startgen[sieveXY[tryFAA, tryFAa, tryFaa, tryMAA, tryMAa,
      tryMaa, trywAf, trywaf, trywAm, trywam, tryαf, tryαm, tryr][[1]], trypm]];
For[time = 1, time ≤ endtime, time++, run[time] = generation[param, run[time - 1]]
]
MODtab1Blue =
  Table[{Round[Exp[exptime]], generation[param, run[Round[Exp[exptime]]]].modvec},
   {exptime, Log[startplot], Log[endtime], 0.1}];
MOD1Blue = ListLogLinearPlot[MODtab1Blue, Joined → True,
  PlotRange → {{startplot, endtime}, {0, 0.75}},
  PlotStyle → {Blue, Thick}, loglinearplotINVoptions]
```

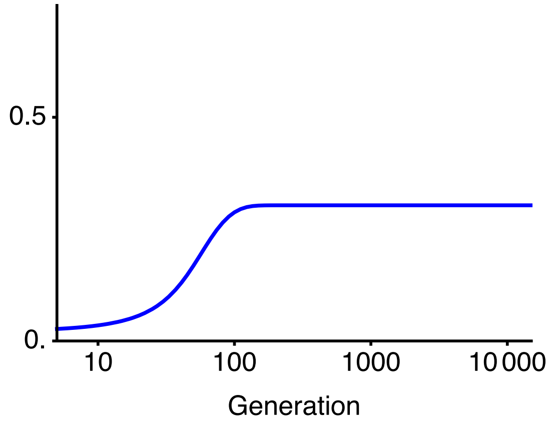

```
tryR = 0.5;
param = {tryFAA, tryFAa, tryFaa, tryMAA, tryMAa, tryMaa, trywAf, trywaf, trywAm,
   trywam, tryαf, tryαm, tryr, tryR, tryr (1 - tryR) + tryR (1 - tryr), tryk};
run[0] = generation[param, startgen[sieveXY[tryFAA, tryFAa, tryFaa, tryMAA, tryMAa,
      tryMaa, trywAf, trywaf, trywAm, trywam, tryαf, tryαm, tryr][[1]], trypm]];
For[time = 1, time ≤ endtime, time++, run[time] = generation[param, run[time - 1]]
]
MODtab1Green =
  Table[{Round[Exp[exptime]], generation[param, run[Round[Exp[exptime]]]].modvec},
   {exptime, Log[startplot], Log[endtime], 0.1}];
MOD1Green = ListLogLinearPlot[MODtab1Green, Joined → True,
  PlotRange → {{startplot, endtime}, {0, 0.75}},
  PlotStyle → {Green, Thick}, loglinearplotINVoptions]
```

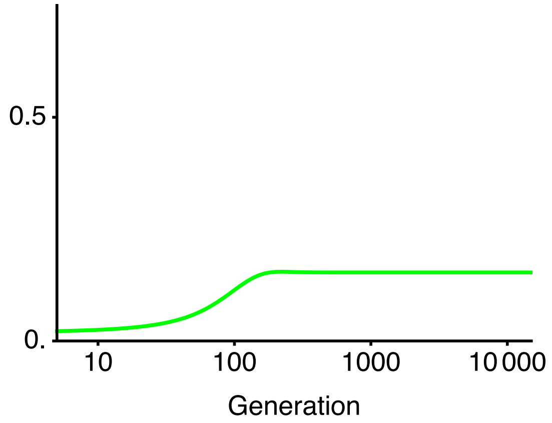


```
plotG = Show[MOD1Black, MOD1Red, MOD1Blue, MOD1Green,
  LogLinearPlot[0.5, {x, 5, endtime}, PlotStyle → {Black, Dashed}],
  Epilog → {
    Text[Style["G", 14, Bold], Scaled@{0.95, 0.95}]
   }
  , PlotRangeClipping → False]
```

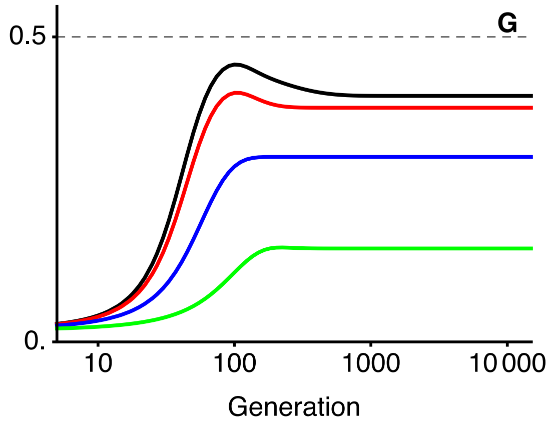

Panel H - temporal invasion dynamic with haploid competition in females

Parameters

```
params = {
   wAm → 1, wam → 1, wAf → 1 + t, waf → 1,
   αm → 1 / 2, αf → 1 / 2,
   Maa → 1, Faa → 1,
   MAa → 1 + (3 / 4) s, FAa → 1 + (3 / 4) s,
   MAA → 1 + s, FAA → 1 + s
  };

moreparams = {
   s → - 1 / 20,
   t → 1 / 10
  };

tryFAA = FAA /. params /. moreparams;
tryFAa = FAa /. params /. moreparams;
tryFaa = Faa /. params /. moreparams;
tryMAA = MAA /. params /. moreparams;
tryMAa = MAa /. params /. moreparams;
tryMaa = Maa /. params /. moreparams;
trywAf = wAf /. params /. moreparams;
trywaf = waf /. params /. moreparams;
trywAm = wAm /. params /. moreparams;
trywam = wam /. params /. moreparams;
tryαf = αf /. params /. moreparams;
tryαm = αm /. params /. moreparams;
```

Plots

```
tryR = 0.001;
param = {tryFAA, tryFAa, tryFaa, tryMAA, tryMAa, tryMaa, trywAf, trywaf, trywAm,
   trywam, tryαf, tryαm, tryr, tryR, tryr (1 - tryR) + tryR (1 - tryr), tryk};
run[0] = generation[param, startgen[sieveXY[tryFAA, tryFAa, tryFaa, tryMAA, tryMAa,
      tryMaa, trywAf, trywaf, trywAm, trywam, tryαf, tryαm, tryr][[1]], trypm]];
For[time = 1, time ≤ endtime, time++, run[time] = generation[param, run[time - 1]]
]
MODtab1Black =
  Table[{Round[Exp[exptime]], generation[param, run[Round[Exp[exptime]]]].modvec},
   {exptime, Log[startplot], Log[endtime], 0.1}];
MOD1Black = ListLogLinearPlot[MODtab1Black, Joined → True,
  PlotRange → {{startplot, endtime}, {0, 0.55}},
  PlotStyle → {Black, Thick}, loglinearplotINVoptions]
```

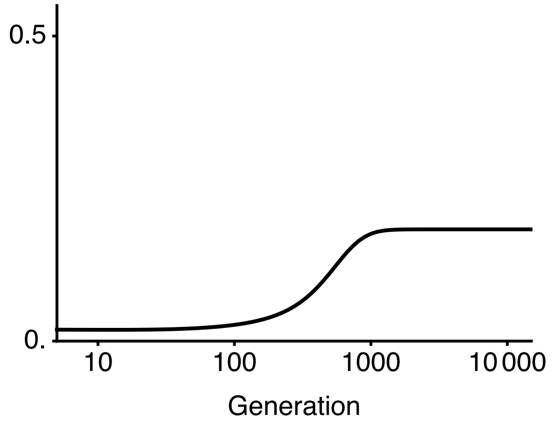

```
tryR = 0.02;
param = {tryFAA, tryFAa, tryFaa, tryMAA, tryMAa, tryMaa, trywAf, trywaf, trywAm,
   trywam, tryαf, tryαm, tryr, tryR, tryr (1 - tryR) + tryR (1 - tryr), tryk};
run[0] = generation[param, startgen[sieveXY[tryFAA, tryFAa, tryFaa, tryMAA, tryMAa,
      tryMaa, trywAf, trywaf, trywAm, trywam, tryαf, tryαm, tryr][[1]], trypm]];
For[time = 1, time ≤ endtime, time++, run[time] = generation[param, run[time - 1]]
]
MODtab1Red =
  Table[{Round[Exp[exptime]], generation[param, run[Round[Exp[exptime]]]].modvec},
   {exptime, Log[startplot], Log[endtime], 0.1}];
MOD1Red = ListLogLinearPlot[MODtab1Red, Joined → True,
  PlotRange → {{startplot, endtime}, {0, 0.55}},
  PlotStyle → {Red, Thick}, loglinearplotINVoptions]
```

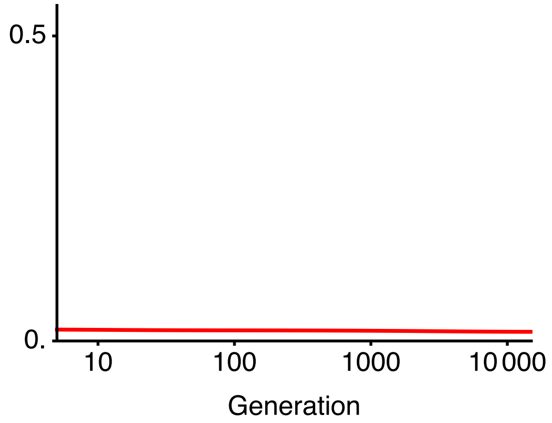

```
tryR = 0.1;
param = {tryFAA, tryFAa, tryFaa, tryMAA, tryMAa, tryMaa, trywAf, trywaf, trywAm,
   trywam, tryαf, tryαm, tryr, tryR, tryr (1 - tryR) + tryR (1 - tryr), tryk};
run[0] = generation[param, startgen[sieveXY[tryFAA, tryFAa, tryFaa, tryMAA, tryMAa,
       tryMaa, trywAf, trywaf, trywAm, trywam, tryαf, tryαm, tryr][[1]], trypm]];
For[time = 1, time ≤ endtime, time++, run[time] = generation[param, run[time - 1]]
]
MODtab1Blue =
  Table[{Round[Exp[exptime]], generation[param, run[Round[Exp[exptime]]]].modvec},
   {exptime, Log[startplot], Log[endtime], 0.1}];
MOD1Blue = ListLogLinearPlot[MODtab1Blue, Joined → True,
  PlotRange → {{startplot, endtime}, {0, 0.55}},
  PlotStyle → {Blue, Thick}, loglinearplotINVoptions]
```

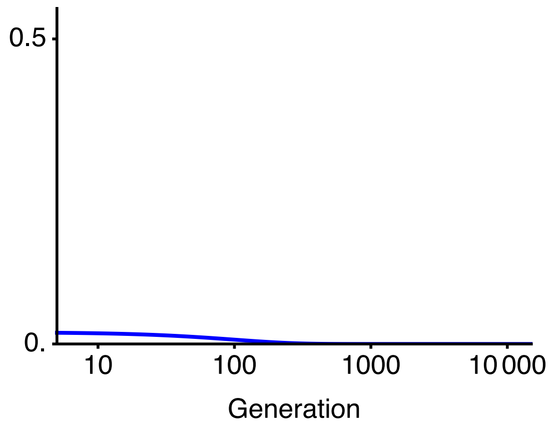

```
tryR = 0.5;
param = {tryFAA, tryFAa, tryFaa, tryMAA, tryMAa, tryMaa, trywAf, trywaf, trywAm,
   trywam, tryαf, tryαm, tryr, tryR, tryr (1 - tryR) + tryR (1 - tryr), tryk};
run[0] = generation[param, startgen[sieveXY[tryFAA, tryFAa, tryFaa, tryMAA, tryMAa,
      tryMaa, trywAf, trywaf, trywAm, trywam, tryαf, tryαm, tryr][[1]], trypm]];
For[time = 1, time ≤ endtime, time++, run[time] = generation[param, run[time - 1]]
]
MODtab1Green =
  Table[{Round[Exp[exptime]], generation[param, run[Round[Exp[exptime]]]].modvec},
   {exptime, Log[startplot], Log[endtime], 0.1}];
MOD1Green = ListLogLinearPlot[MODtab1Green, Joined → True,
  PlotRange → {{startplot, endtime}, {0, 0.55}},
  PlotStyle → {Green, Thick}, loglinearplotINVoptions]
```

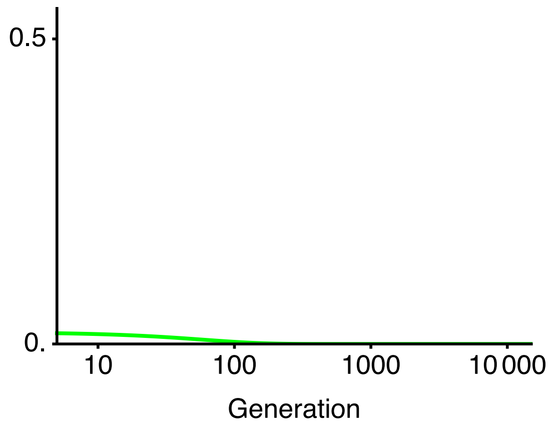


```
plotH = Show[MOD1Black, MOD1Red, MOD1Blue, MOD1Green,
  LogLinearPlot[0.5, {x, 5, endtime}, PlotStyle → {Black, Dashed}],
  Epilog → {
    Rotate[Text[Style["", 14], Scaled@ylabpos], 90 Degree],
    Text[Style["H", 14, Bold], Scaled@{0.95, 0.95}]
   }
  , PlotRangeClipping → False]
```

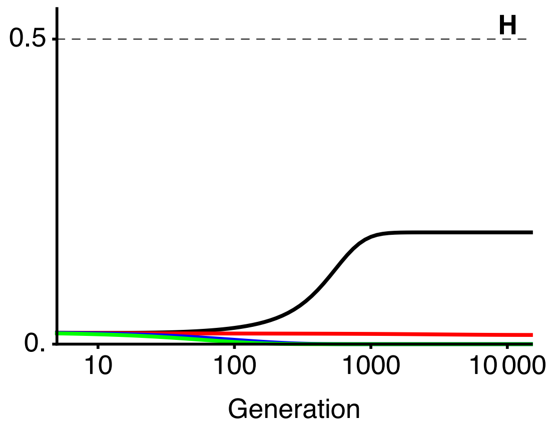

All panels

```
GraphicsGrid[{
  {plotA, plotB, plotC, plotD},
  {plotE, plotF, plotG, plotH}
 },
 Spacings → {-25, -50}]

Export[plotdir <> "All_plot_combined_PloidAntag.eps", % // rasterTrick];
```

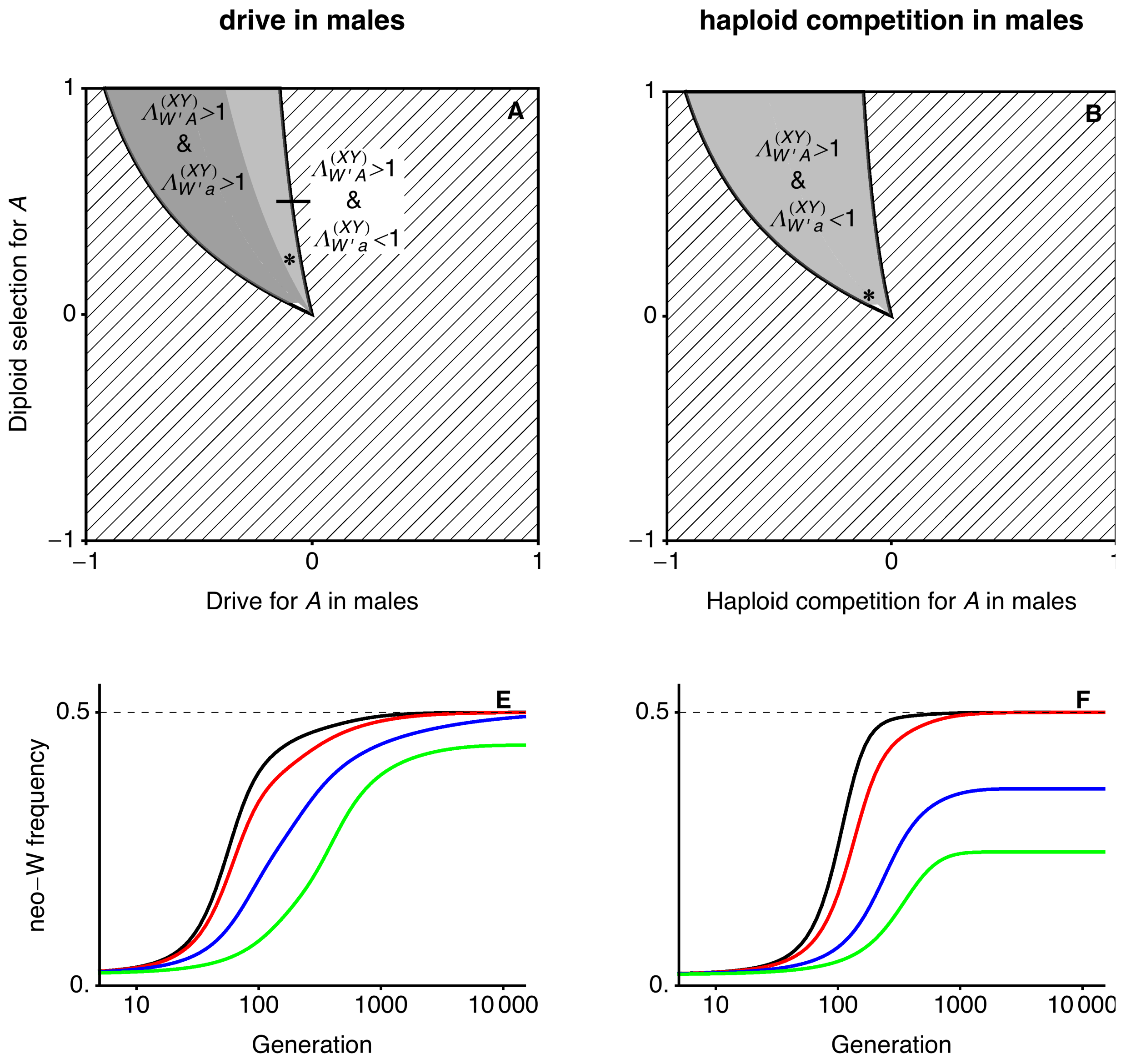

Figure S.9 - polymorphic sex determination can be stable

extra params

```
startplot = 5;
endtime = 5000;
trypm = 0.01;
tryk = 1;

Pad = {{50, 10}, {40, 30}};

loglinearplotINVoptions = {
   Frame → {{True, False}, {True, False}},
   FrameStyle → Directive[Black, Thickness[lwd]],
   FrameTicksStyle → {{Directive[Black, Thickness[lwd], FontColor → Black],
      Directive[Black, Thickness[lwd]]},
     {Directive[Black, Thickness[lwd]], Directive[Black, Thickness[lwd]]}},
   FrameTicks → {{{5, 5, {0, 0.01}}, {10, 10, {0, 0.01}}, {50, 50, {0, 0.01}},
      {100, 100, {0, 0.01}}, (*{500,500,{0,0.01}},*){1000, 1000, {0, 0.01}}
      (*,{5000,5000,{0,0.01}}*)}, Table[{y, y, ticksize}, {y, 0, 1, 0.5}]},
   BaseStyle → {FontFamily → "Helvetica", FontSize → 14},
   ImageSize → {xsize, xsize aspectratio },
   AspectRatio → aspectratio,
   Axes → True,
   FrameLabel → {Style["Generation", FontSize → 14],},
   ImagePadding → Pad
  };
```

Panel A - ploidy-antagonism with male drive (parameters from fig 4B green)

Parameters

```
tryFAA = 1 + (1 / 5);
tryFAa = 1 + (7 / 10) (1 / 5);
tryFaa = 1;
tryMAA = 1 + (1 / 5);
tryMAa = 1 + (7 / 10) (1 / 5);
tryMaa = 1;
trywAf = 1;
trywaf = 1;
trywAm = 1;
trywam = 1;
tryαf = 1 / 2;
tryαm = 1 / 2 - (1 / 10);
tryr = 1 / 50;
tryR = 1 / 2;

param = {tryFAA, tryFAa, tryFaa, tryMAA, tryMAa, tryMaa, trywAf, trywaf, trywAm,
   trywam, tryαf, tryαm, tryr, tryR, tryr (1 - tryR) + tryR (1 - tryr), tryk};
```

Simulation

```
sieveXY[tryFAA, tryFAa, tryFaa, tryMAA, tryMAa,
  tryMaa, trywAf, trywaf, trywAm, trywam, tryαf, tryαm, tryr][[1]]
```

```
{0.65153, 0.615375, 0.0840379, 0.556532}
```

```
run[0] =
  generation[param, startgen[sieveXY[tryFAA, tryFAa, tryFaa, tryMAA, tryMAa, tryMaa,
      trywAf, trywaf, trywAm, trywam, tryαf, tryαm, tryr][[1]], trypm]];
For[time = 1, time ≤ endtime, time++, run[time] = generation[param, run[time - 1]]]
```

Plot neo-W frequency in females

```
Wfemvec = {0, 0, 1, 1, 0, 0, 1, 1, 0, 0, 0, 0, 0, 0, 0, 0};
Table[{Round[Exp[exptime]], generation[param, run[Round[Exp[exptime]]]].Wfemvec},
  {exptime, Log[startplot], Log[endtime], 0.1}];
Wfem = ListLogLinearPlot[%, Joined → True, PlotRange → {{startplot, endtime}, {0, 1}},
  PlotStyle → {Red, Thick}, loglinearplotINVoptions]
```

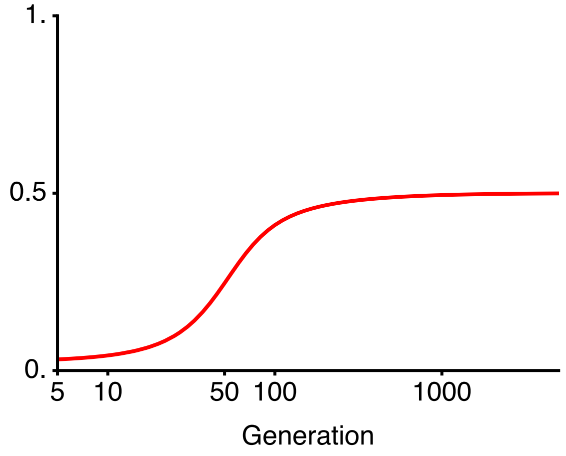

Plot Y frequency in females

```
Yfemvec = {0, 0, 0, 0, 1, 1, 1, 1, 0, 0, 0, 0, 0, 0, 0, 0};
Table[{Round[Exp[exptime]], generation[param, run[Round[Exp[exptime]]]].Yfemvec},
  {exptime, Log[startplot], Log[endtime], 0.1}];
Yfem = ListLogLinearPlot[%, Joined → True, PlotRange → {{startplot, endtime}, {0, 1}},
  PlotStyle → {Blue, Thick}, loglinearplotINVoptions]
```

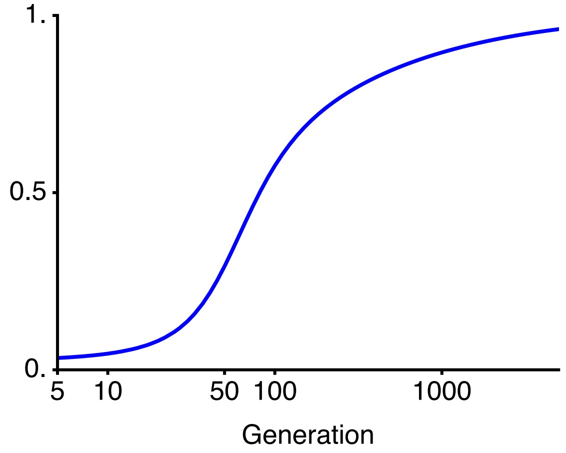

Plot Y frequency in males

```
Ymalvec = {0, 0, 0, 0, 0, 0, 0, 0, 0, 0, 0, 0, 1, 1, 1, 1};
Table[{Round[Exp[exptime]], generation[param, run[Round[Exp[exptime]]]].Ymalvec},
  {exptime, Log[startplot], Log[endtime], 0.1}];
Ymal = ListLogLinearPlot[%, Joined → True, PlotRange → {{startplot, endtime}, {0, 1}},
  PlotStyle → {Blue, Thick, Dashing[Large]}, loglinearplotINVoptions]
```

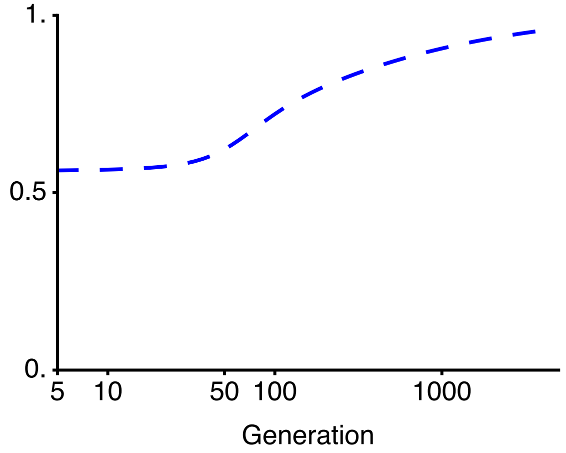

Plot A frequency in females

```
Afemvec = {1, 0, 1, 0, 1, 0, 1, 0, 0, 0, 0, 0, 0, 0, 0, 0};
Table[
  {Round[Exp[exptime]], generation[param, run[Round[Exp[exptime]]]].Afemvec / 2},
  {exptime, Log[startplot], Log[endtime], 0.1}];
Afem = ListLogLinearPlot[%, Joined → True,
  PlotRange → {{startplot, endtime}, {0, 1.1}},
  PlotStyle → {Black, Thick}, loglinearplotINVoptions]
```

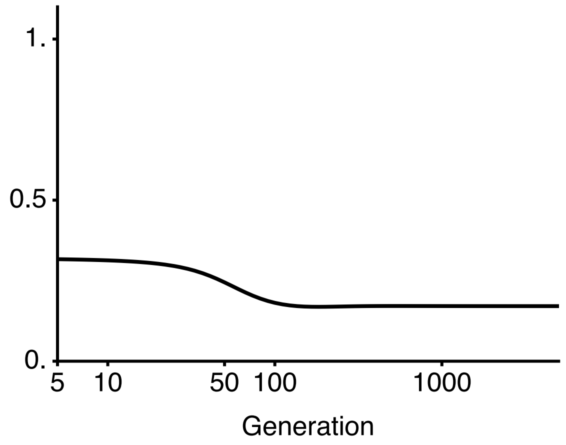

Plot A frequency in males

```
Amalvec = {0, 0, 0, 0, 0, 0, 0, 0, 1, 0, 1, 0, 1, 0, 1, 0};
Table[
  {Round[Exp[exptime]], generation[param, run[Round[Exp[exptime]]]].Amalvec / 2},
  {exptime, Log[startplot], Log[endtime], 0.1}];
Amal = ListLogLinearPlot[%, Joined → True,
  PlotRange → {{startplot, endtime}, {0, 1.1}},
  PlotStyle → {Black, Thick, Dashing[Large]}, loglinearplotINVoptions]
```

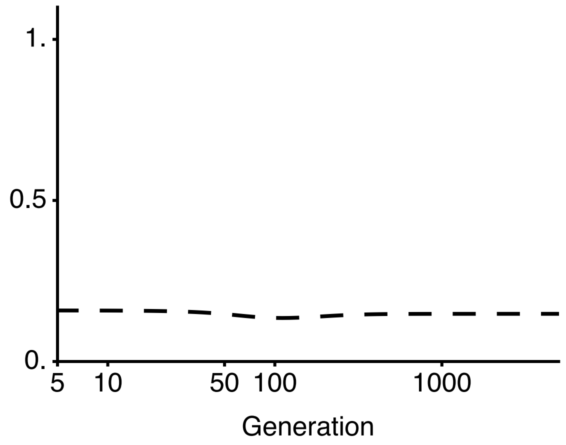

Plot all

```
plotA = Show[Wfem, Yfem, Ymal, Afem, Amal,
  LogLinearPlot[0.5, {x, 5, endtime}, PlotStyle → {Black, Dashed}]]
```

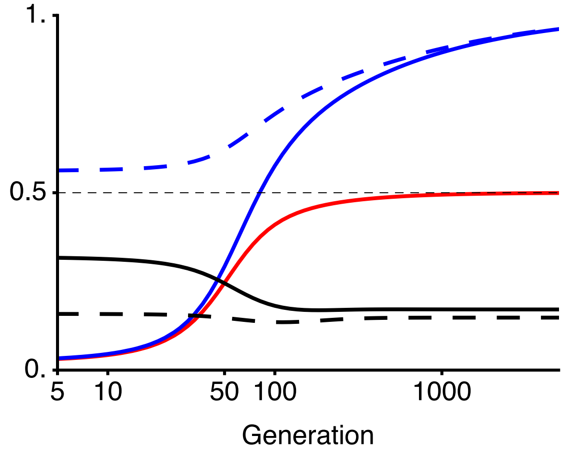

Panel B - overdominance in both sexes, no haploid selection (parameters from fig S.2C green)

Parameters

```
tryFAA = 0.6;
tryFAa = 1;
tryFaa = 0.6;
tryMAA = 0.7;
tryMAa = 1;
tryMaa = 0.5;
trywAf = 1;
trywaf = 1;
trywAm = 1;
trywam = 1;
tryαf = 1 / 2;
tryαm = 1 / 2;
tryr = 0.005;
tryR = 1 / 2;

param = {tryFAA, tryFAa, tryFaa, tryMAA, tryMAa, tryMaa, trywAf, trywaf, trywAm,
   trywam, tryαf, tryαm, tryr, tryR, tryr (1 - tryR) + tryR (1 - tryr), tryk};
```

Simulation

```
sieveXY[tryFAA, tryFAa, tryFaa, tryMAA, tryMAa,
  tryMaa, trywAf, trywaf, trywAm, trywam, tryαf, tryαm, tryr][[2]]
{0.720702, 0.82555, 0.0461837, 0.5}
```

```
run[0] =
  generation[param, startgen[sieveXY[tryFAA, tryFAa, tryFaa, tryMAA, tryMAa, tryMaa,
      trywAf, trywaf, trywAm, trywam, tryαf, tryαm, tryr][[2]], trypm]];
For[time = 1, time ≤ endtime, time++, run[time] = generation[param, run[time - 1]]]
```

Plot neo-W frequency in females

```
Wfemvec = {0, 0, 1, 1, 0, 0, 1, 1, 0, 0, 0, 0, 0, 0, 0, 0};
Table[{Round[Exp[exptime]], generation[param, run[Round[Exp[exptime]]]].Wfemvec},
  {exptime, Log[startplot], Log[endtime], 0.1}];
Wfem = ListLogLinearPlot[%, Joined → True, PlotRange → {{startplot, endtime}, {0, 1}},
  PlotStyle → {Red, Thick}, loglinearplotINVoptions]
```

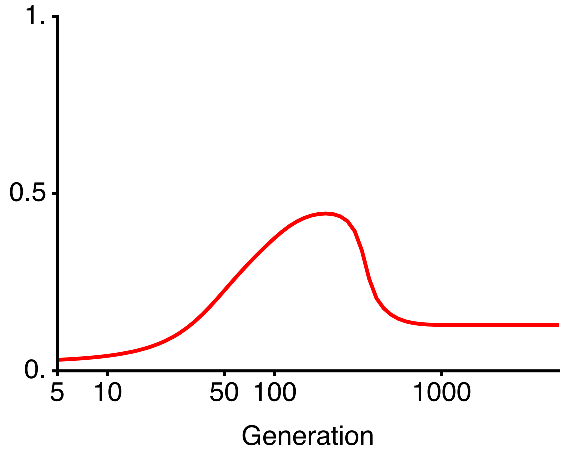

Plot Y frequency in females

```
Yfemvec = {0, 0, 0, 0, 1, 1, 1, 1, 0, 0, 0, 0, 0, 0, 0, 0};
Table[{Round[Exp[exptime]], generation[param, run[Round[Exp[exptime]]]].Yfemvec},
  {exptime, Log[startplot], Log[endtime], 0.1}];
Yfem = ListLogLinearPlot[%, Joined → True, PlotRange → {{startplot, endtime}, {0, 1}},
  PlotStyle → {Blue, Thick}, loglinearplotINVoptions]
```

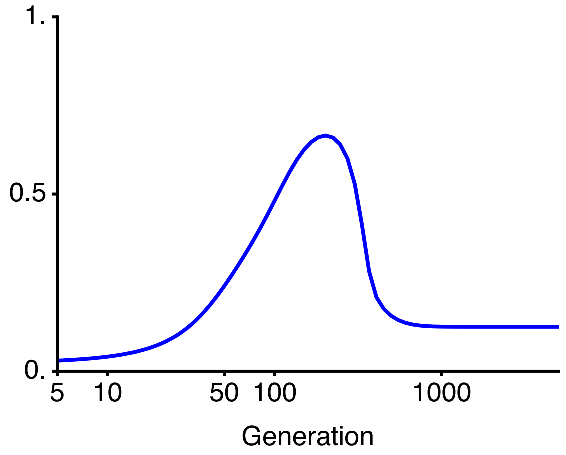

Plot Y frequency in males

```
Ymalvec = {0, 0, 0, 0, 0, 0, 0, 0, 0, 0, 0, 0, 1, 1, 1, 1};
Table[{Round[Exp[exptime]], generation[param, run[Round[Exp[exptime]]]].Ymalvec},
  {exptime, Log[startplot], Log[endtime], 0.1}];
Ymal = ListLogLinearPlot[%, Joined → True, PlotRange → {{startplot, endtime}, {0, 1}},
  PlotStyle → {Blue, Thick, Dashing[Large]}, loglinearplotINVoptions]
```

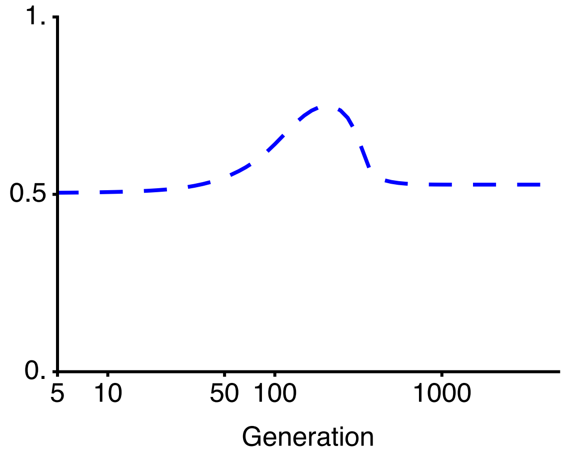

Plot A frequency in females

```
Afemvec = {1, 0, 1, 0, 1, 0, 1, 0, 0, 0, 0, 0, 0, 0, 0, 0};
Table[
  {Round[Exp[exptime]], generation[param, run[Round[Exp[exptime]]]].Afemvec / 2},
  {exptime, Log[startplot], Log[endtime], 0.1}];
Afem = ListLogLinearPlot[%, Joined → True,
  PlotRange → {{startplot, endtime}, {0, 1.1}},
  PlotStyle → {Black, Thick}, loglinearplotINVoptions]
```

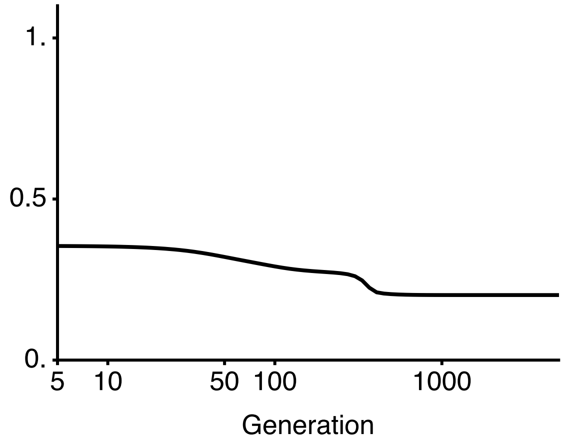

Plot A frequency in males

```
Amalvec = {0, 0, 0, 0, 0, 0, 0, 0, 1, 0, 1, 0, 1, 0, 1, 0};
Table[
  {Round[Exp[exptime]], generation[param, run[Round[Exp[exptime]]]].Amalvec / 2},
  {exptime, Log[startplot], Log[endtime], 0.1}];
Amal = ListLogLinearPlot[%, Joined → True,
  PlotRange → {{startplot, endtime}, {0, 1.1}},
  PlotStyle → {Black, Thick, Dashing[Large]}, loglinearplotINVoptions]
```

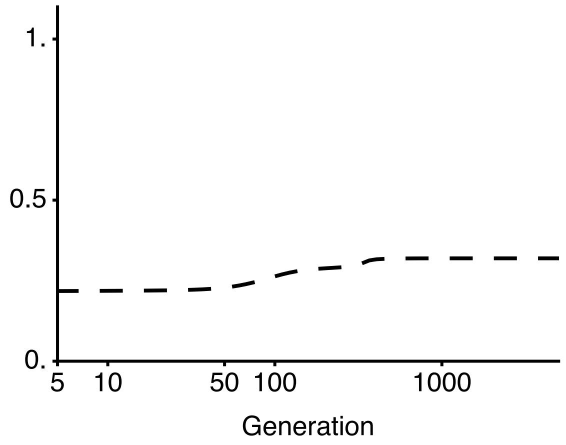

Plot all

```
plotB = Show[Wfem, Yfem, Ymal, Afem, Amal,
  LogLinearPlot[0.5, {x, 5, endtime}, PlotStyle → {Black, Dashed}]]
```

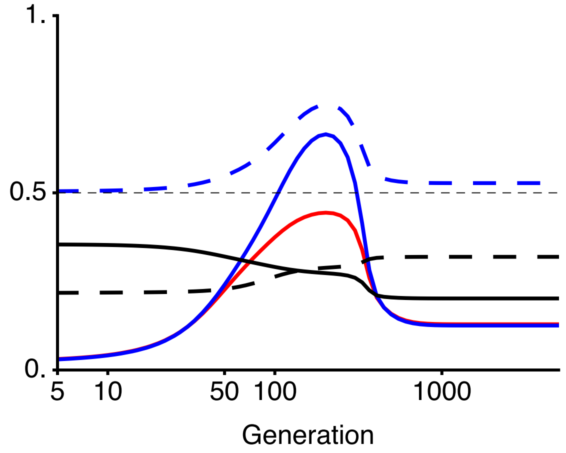

Panel C - sex-antagonism with male drive (parameters from fig S.3C green)

Parameters

```
tryFAA = 1.05;
tryFAa = 1;
tryFaa = 0.85;
tryMAA = 0.85;
tryMAa = 1;
tryMaa = 1.05;
trywAf = 1;
trywaf = 1;
trywAm = 1;
trywam = 1;
tryαf = 1 / 2;
tryαm = 1 / 2 - 8 / 100;
tryr = 0.005;
tryR = 1 / 2;

param = {tryFAA, tryFAa, tryFaa, tryMAA, tryMAa, tryMaa, trywAf, trywaf, trywAm,
   trywam, tryαf, tryαm, tryr, tryR, tryr (1 - tryR) + tryR (1 - tryr), tryk};
```

Simulation

```
sieveXY[tryFAA, tryFAa, tryFaa, tryMAA, tryMAa,
  tryMaa, trywAf, trywaf, trywAm, trywam, tryαf, tryαm, tryr][[1]]
{0.418811, 0.363484, 0.00693408, 0.531719}

run[0] =
  generation[param, startgen[sieveXY[tryFAA, tryFAa, tryFaa, tryMAA, tryMAa, tryMaa,
      trywAf, trywaf, trywAm, trywam, tryαf, tryαm, tryr][[1]], trypm]];
For[time = 1, time ≤ endtime, time++, run[time] = generation[param, run[time - 1]]]
```

Plot neo-W frequency in females

```
Wfemvec = {0, 0, 1, 1, 0, 0, 1, 1, 0, 0, 0, 0, 0, 0, 0, 0};
Table[{Round[Exp[exptime]], generation[param, run[Round[Exp[exptime]]]].Wfemvec},
  {exptime, Log[startplot], Log[endtime], 0.1}];
Wfem = ListLogLinearPlot[%, Joined → True, PlotRange → {{startplot, endtime}, {0, 1}},
  PlotStyle → {Red, Thick}, loglinearplotINVoptions]
```

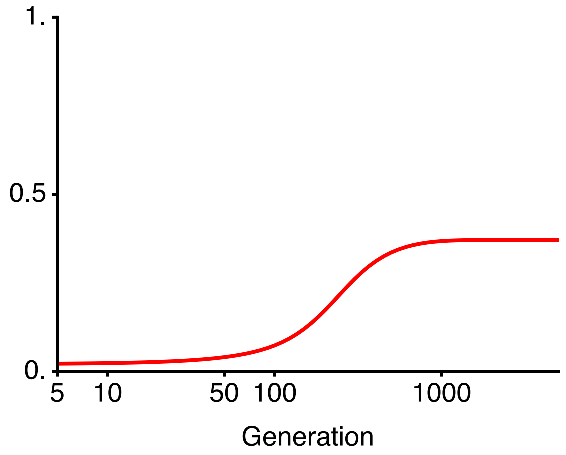

Plot Y frequency in females

```
Yfemvec = {0, 0, 0, 0, 1, 1, 1, 1, 0, 0, 0, 0, 0, 0, 0, 0};
Table[{Round[Exp[exptime]], generation[param, run[Round[Exp[exptime]]]].Yfemvec},
  {exptime, Log[startplot], Log[endtime], 0.1}];
Yfem = ListLogLinearPlot[%, Joined → True, PlotRange → {{startplot, endtime}, {0, 1}},
  PlotStyle → {Blue, Thick}, loglinearplotINVoptions]
```

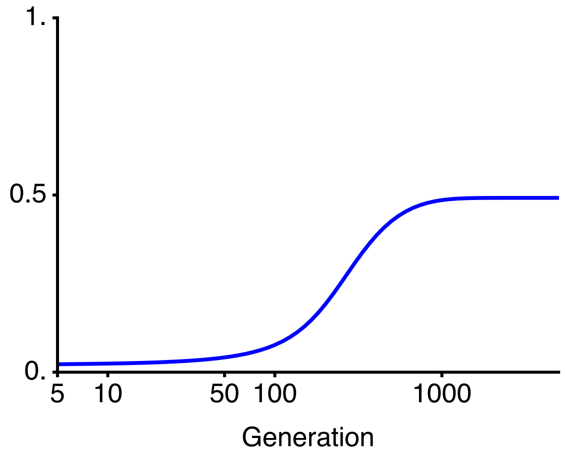

Plot Y frequency in males

```
Ymalvec = {0, 0, 0, 0, 0, 0, 0, 0, 0, 0, 0, 0, 1, 1, 1, 1};
Table[{Round[Exp[exptime]], generation[param, run[Round[Exp[exptime]]]].Ymalvec},
  {exptime, Log[startplot], Log[endtime], 0.1}];
Ymal = ListLogLinearPlot[%, Joined → True, PlotRange → {{startplot, endtime}, {0, 1}},
  PlotStyle → {Blue, Thick, Dashing[Large]}, loglinearplotINVoptions]
```

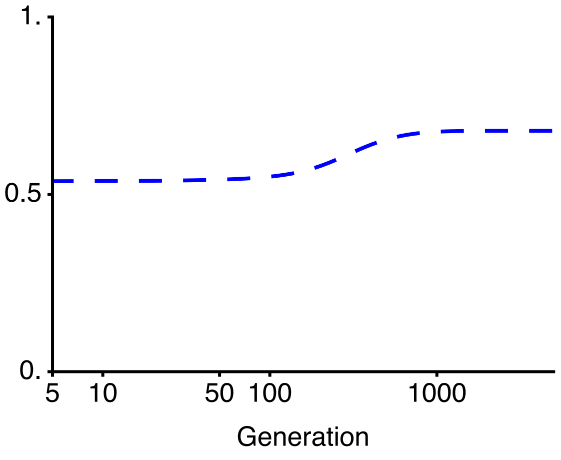

Plot A frequency in females

```
Afemvec = {1, 0, 1, 0, 1, 0, 1, 0, 0, 0, 0, 0, 0, 0, 0, 0};
Table[
  {Round[Exp[exptime]], generation[param, run[Round[Exp[exptime]]]].Afemvec / 2},
  {exptime, Log[startplot], Log[endtime], 0.1}];
Afem = ListLogLinearPlot[%, Joined → True,
  PlotRange → {{startplot, endtime}, {0, 1.1}},
  PlotStyle → {Black, Thick}, loglinearplotINVoptions]
```

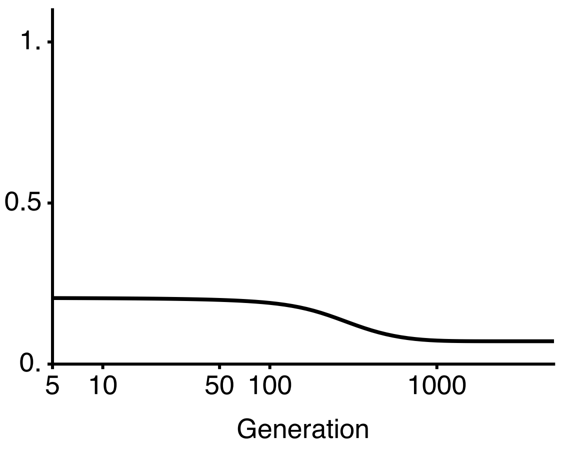

Plot A frequency in males

```
Amalvec = {0, 0, 0, 0, 0, 0, 0, 0, 1, 0, 1, 0, 1, 0, 1, 0};
Table[
  {Round[Exp[exptime]], generation[param, run[Round[Exp[exptime]]]].Amalvec / 2},
  {exptime, Log[startplot], Log[endtime], 0.1}];
Amal = ListLogLinearPlot[%, Joined → True,
  PlotRange → {{startplot, endtime}, {0, 1.1}},
  PlotStyle → {Black, Thick, Dashing[Large]}, loglinearplotINVoptions]
```

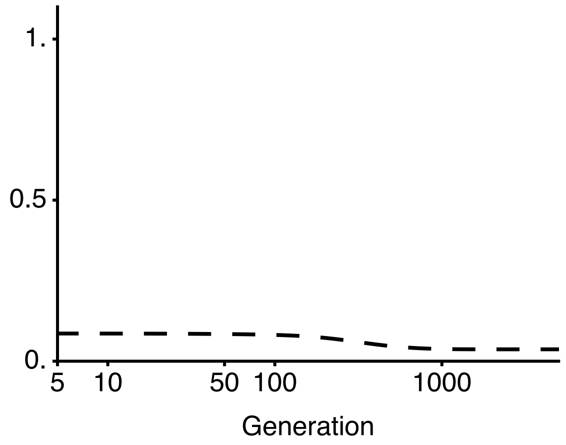

Plot all

```
plotC = Show[Wfem, Yfem, Ymal, Afem, Amal,
  LogLinearPlot[0.5, {x, 5, endtime}, PlotStyle → {Black, Dashed}]]
```

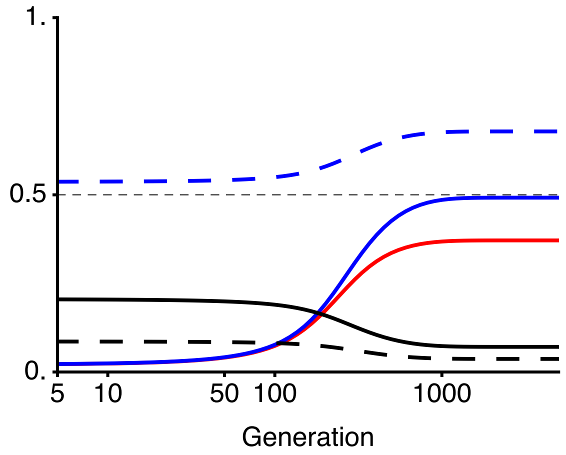

Panel D - ploidy-antagonism with pollen competition (parameters from fig S.8F green)

Parameters

```
tryFAA = 1 + (1 / 10);
tryFAa = 1 + (3 / 4) (1 / 10);
tryFaa = 1;
tryMAA = 1 + (1 / 10);
tryMAa = 1 + (3 / 4) (1 / 10);
tryMaa = 1;
trywAf = 1;
trywaf = 1;
trywAm = 1 - 1 / 10;
trywam = 1;
tryαf = 1 / 2;
tryαm = 1 / 2;
tryr = 1 / 200;
tryR = 1 / 2;

param = {tryFAA, tryFAa, tryFaa, tryMAA, tryMAa, tryMaa, trywAf, trywaf, trywAm,
   trywam, tryαf, tryαm, tryr, tryR, tryr (1 - tryR) + tryR (1 - tryr), tryk};
```

Simulation

```
sieveXY[tryFAA, tryFAa, tryFaa, tryMAA, tryMAa,
  tryMaa, trywAf, trywaf, trywAm, trywam, tryαf, tryαm, tryr][[1]]
{0.819467, 0.82566, 0.0576155, 0.5}

run[0] =
  generation[param, startgen[sieveXY[tryFAA, tryFAa, tryFaa, tryMAA, tryMAa, tryMaa,
      trywAf, trywaf, trywAm, trywam, tryαf, tryαm, tryr][[1]], trypm]];
For[time = 1, time ≤ endtime, time++, run[time] = generation[param, run[time - 1]]]
```

Plot neo-W frequency in females

```
Wfemvec = {0, 0, 1, 1, 0, 0, 1, 1, 0, 0, 0, 0, 0, 0, 0, 0};
Table[{Round[Exp[exptime]], generation[param, run[Round[Exp[exptime]]]].Wfemvec},
  {exptime, Log[startplot], Log[endtime], 0.1}];
Wfem = ListLogLinearPlot[%, Joined → True, PlotRange → {{startplot, endtime}, {0, 1}},
  PlotStyle → {Red, Thick}, loglinearplotINVoptions]
```

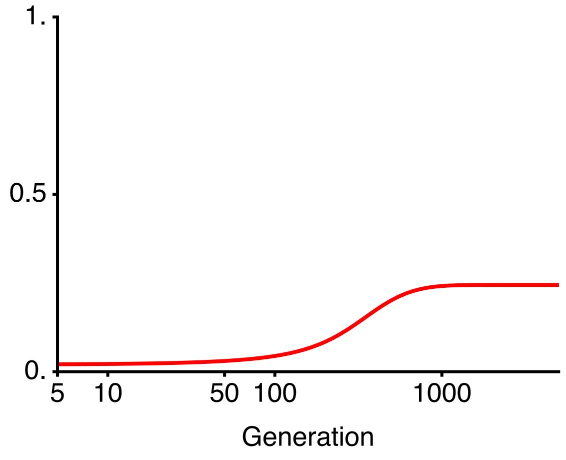

Plot Y frequency in females

```
Yfemvec = {0, 0, 0, 0, 1, 1, 1, 1, 0, 0, 0, 0, 0, 0, 0, 0};
Table[{Round[Exp[exptime]], generation[param, run[Round[Exp[exptime]]]].Yfemvec},
  {exptime, Log[startplot], Log[endtime], 0.1}];
Yfem = ListLogLinearPlot[%, Joined → True, PlotRange → {{startplot, endtime}, {0, 1}},
  PlotStyle → {Blue, Thick}, loglinearplotINVoptions]
```

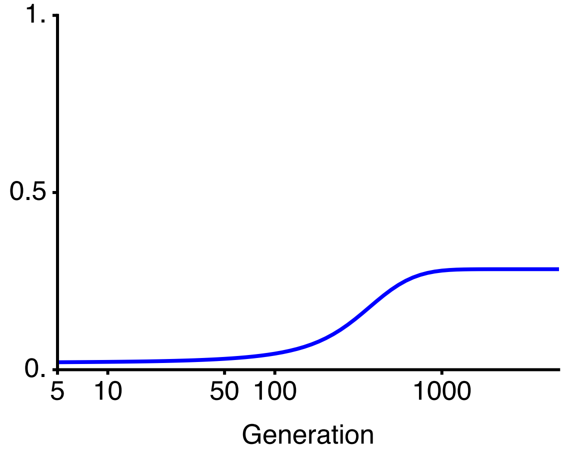

Plot Y frequency in males

```
Ymalvec = {0, 0, 0, 0, 0, 0, 0, 0, 0, 0, 0, 0, 1, 1, 1, 1};
Table[{Round[Exp[exptime]], generation[param, run[Round[Exp[exptime]]]].Ymalvec},
  {exptime, Log[startplot], Log[endtime], 0.1}];
Ymal = ListLogLinearPlot[%, Joined → True, PlotRange → {{startplot, endtime}, {0, 1}},
  PlotStyle → {Blue, Thick, Dashing[Large]}, loglinearplotINVoptions]
```

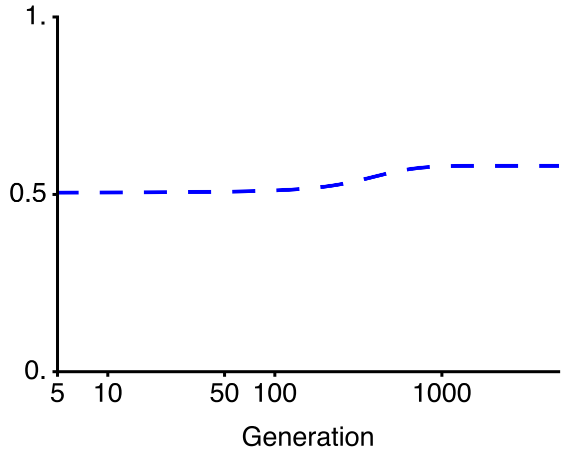

Plot A frequency in females

```
Afemvec = {1, 0, 1, 0, 1, 0, 1, 0, 0, 0, 0, 0, 0, 0, 0, 0};
Table[
  {Round[Exp[exptime]], generation[param, run[Round[Exp[exptime]]]].Afemvec / 2},
  {exptime, Log[startplot], Log[endtime], 0.1}];
Afem = ListLogLinearPlot[%, Joined → True,
  PlotRange → {{startplot, endtime}, {0, 1.1}},
  PlotStyle → {Black, Thick}, loglinearplotINVoptions]
```

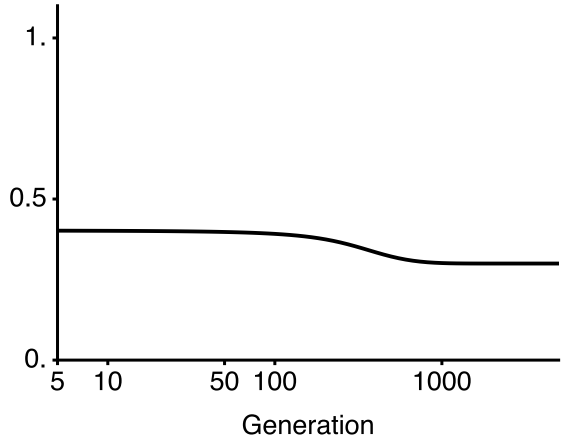

Plot A frequency in males

```
Amalvec = {0, 0, 0, 0, 0, 0, 0, 0, 1, 0, 1, 0, 1, 0, 1, 0};
Table[
  {Round[Exp[exptime]], generation[param, run[Round[Exp[exptime]]]].Amalvec / 2},
  {exptime, Log[startplot], Log[endtime], 0.1}];
Amal = ListLogLinearPlot[%, Joined → True,
  PlotRange → {{startplot, endtime}, {0, 1.1}},
  PlotStyle → {Black, Thick, Dashing[Large]}, loglinearplotINVoptions]
```

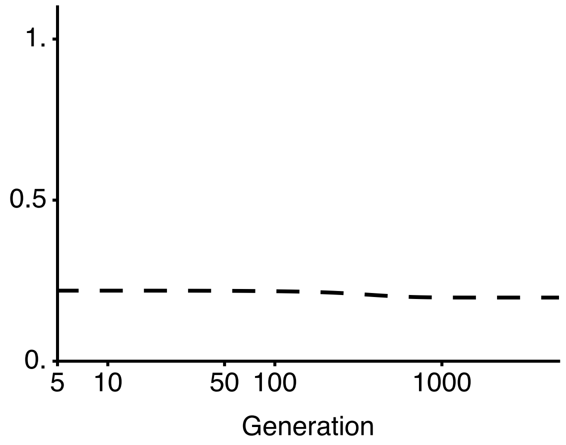

Plot all

```
plotD = Show[Wfem, Yfem, Ymal, Afem, Amal,
  LogLinearPlot[0.5, {x, 5, endtime}, PlotStyle → {Black, Dashed}]]
```

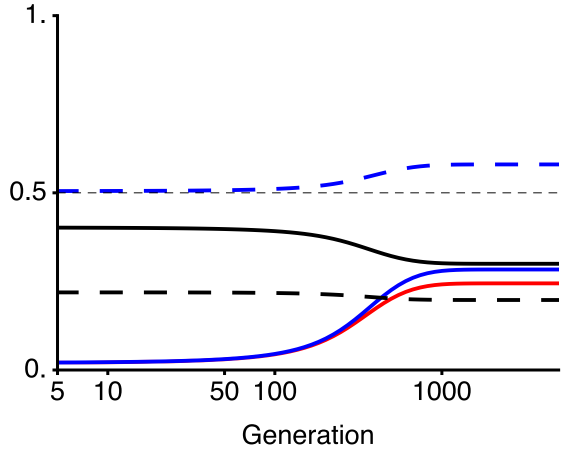

All panels

```
GraphicsRow[
 {
  Show[
   plotA,
   Epilog → {
     Rotate[Text[Style["Frequency", 16], Scaled@{-0.15, 0.5}], π / 2],
     Text[Style["A", 16, Bold], Scaled@{0.05, 0.95}],
     Text[Style["ploidy-antagonism", 16], Scaled@{0.5, 0.95}]
    },
   PlotRangeClipping → False
  ],
  Show[plotB,
   Epilog → {
     Text[Style["B", 16, Bold], Scaled@{0.05, 0.95}],
     Text[Style["overdominance", 16], Scaled@{0.5, 0.95}]
    }
  ],
  Show[plotC,
   Epilog → {
     Text[Style["C", 16, Bold], Scaled@{0.05, 0.95}],
     Text[Style["sex-antagonism", 16], Scaled@{0.5, 0.95}]
    }
  ](*,
  Show[plotD,
   Epilog→{
     Text[Style["D",16,Bold],Scaled@{0.05,0.95}]
    }
  ]*)
 },
 Spacings → -50
]

Export[plotdir <> "Freq_plot_combined_PloidAntag.eps",
  Magnify[%(*//rasterTrick*), 7.5 / 10]];
```

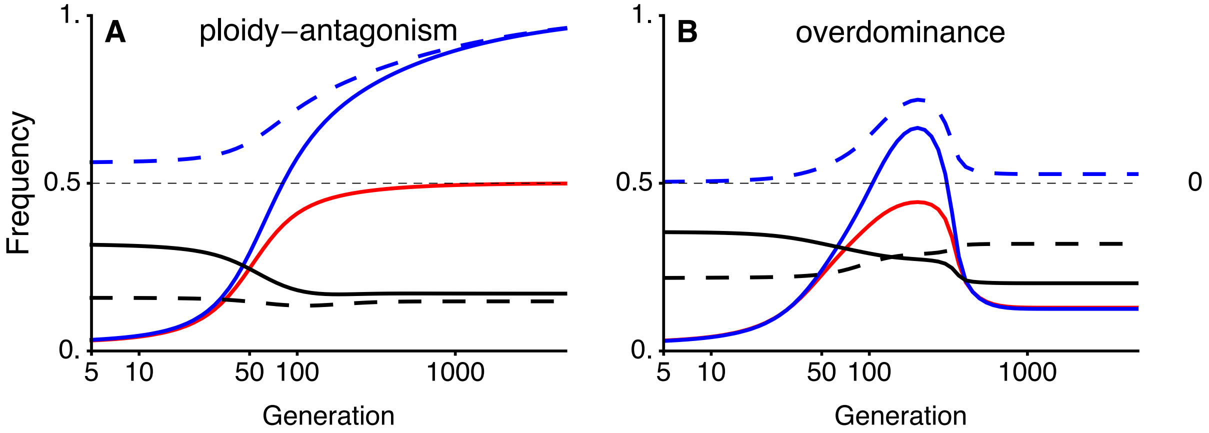
A
ploidy−antagonism
B
overdominance
Frequency
1.
0.5
0.
5
10
50
100
1000
Generation
0
