# Supplementary material for: Haploid selection, sex ratio bias, and transitions between sex-determining systems
Source: PLoS Biol. 2018 Jun 25;16(6):e2005609. doi: 10.1371/journal.pbio.2005609 (PMC6042799; doi:10.1371/journal.pbio.2005609)
Supplement: S1 Table — (PDF) [file pbio.2005609.s004.pdf]

Table S1. Substitutions for different loci orders assuming no interference.

Order of loci	Substitution
SDR-A-M	$\rho = r(1-R) + R(1-r)$
SDR-M-A	$r = \rho(1-R) + R(1-\rho)$
A-SDR-M	$R = r(1-\rho) + \rho(1-r)$
