# Supplementary material for: Haploid selection, sex ratio bias, and transitions between sex-determining systems
Source: PLoS Biol. 2018 Jun 25;16(6):e2005609. doi: 10.1371/journal.pbio.2005609 (PMC6042799; doi:10.1371/journal.pbio.2005609)
Supplement: S2 Table — (PDF) [file pbio.2005609.s005.pdf]

Table S2. Mean fitnesses and zygotic sex ratio in the resident population (M fixed, XY sex determination)

Sex & life cycle stage	Mean fitness
female gametes ($\bar{w}_H^{♀}$)	$p_X^{♀} w_A^{♀} + (1 - p_X^{♀}) w_a^{♀}$
X-bearing male gametes ($\bar{w}_{HX}^{♂}$)	$p_X^{♂} w_A^{♂} + (1 - p_X^{♂}) w_a^{♂}$
Y-bearing male gametes ($\bar{w}_{HY}^{♂}$)	$p_Y^{♂} w_A^{♂} + (1 - p_Y^{♂}) w_a^{♂}$
male gametes ($\bar{w}_H^{♂}$)	$(1 - q)\bar{w}_{HX}^{♂} + q\bar{w}_{HY}^{♂}$
females ($\bar{w}^{♀}$)	$\big[p_X^{♀} w_A^{♀} p_X^{♂} w_A^{♂} w_{AA}^{♀} +$ $(1 - p_X^{♀}) w_a^{♀} p_X^{♂} w_A^{♂} w_{Aa}^{♀} +$ $p_X^{♀} w_A^{♀} (1 - p_X^{♂}) w_a^{♂} w_{Aa}^{♀} +$ $(1 - p_X^{♀}) w_a^{♀} (1 - p_X^{♂}) w_a^{♂} w_{aa}^{♀}\big] / \left(\bar{w}_H^{♀} \bar{w}_{HX}^{♂}\right)$
males ($\bar{w}^{♂}$)	$\big[p_X^{♀} w_A^{♀} p_Y^{♂} w_A^{♂} w_{AA}^{♂} +$ $(1 - p_X^{♀}) w_a^{♀} p_Y^{♂} w_A^{♂} w_{Aa}^{♂} +$ $p_X^{♀} w_A^{♀} (1 - p_Y^{♂}) w_a^{♂} w_{Aa}^{♂} +$ $(1 - p_X^{♀}) w_a^{♀} (1 - p_Y^{♂}) w_a^{♂} w_{aa}^{♂}\big] / \left(\bar{w}_H^{♀} \bar{w}_{HY}^{♂}\right)$
fraction zygotes male (ζ)	$q \left[p_Y^{♂} w_A^{♂} + (1 - p_Y^{♂}) w_a^{♂}\right] / \bar{w}_H^{♂}$
