# Supplementary material for: Haploid selection, sex ratio bias, and transitions between sex-determining systems
Source: PLoS Biol. 2018 Jun 25;16(6):e2005609. doi: 10.1371/journal.pbio.2005609 (PMC6042799; doi:10.1371/journal.pbio.2005609)
Supplement: S1 Text — (PDF) [file pbio.2005609.s006.pdf]

S1 Appendix

Recursion equations

In each generation we census the genotype frequencies in male and female gametes/gametophytes (hereafter, gametes) between meiosis (and any meiotic drive) and gametic competition. At this stage we denote the frequencies of X- and Y-bearing gametes from males and females x_i° and y_i°. The superscript $\circ \in \{♂, ♀\}$ specifies the sex of the diploid that the gamete came from. The subscript $i \in \{1, 2, 3, 4\}$ specifies the genotype at the selected locus **A** and at the novel sex-determining locus **M**, where $1 = AM$, $2 = aM$, $3 = Am$, and $4 = am$. The gamete frequencies from each sex sum to one, $\sum_i x_i^\circ + y_i^\circ = 1$.

Competition then occurs among gametes of the same sex (e.g., among eggs and among sperm separately) according to the genotype at the **A** locus ($w_1^\circ = w_3^\circ = w_A^\circ$, $w_2^\circ = w_4^\circ = w_a^\circ$, see Table 1). The genotype frequencies after gametic competition are $x_i^{\circ,s} = w_i^\circ x_i^\circ / \bar{w}_H^\circ$ and $y_i^{\circ,s} = w_i^\circ y_i^\circ / \bar{w}_H^\circ$, where $\bar{w}_H^\circ = \sum_i w_i^\circ x_i^\circ + w_i^\circ y_i^\circ$ is the mean fitness of male ($\circ = ♂$) or female ($\circ = ♀$) gametes.

Random mating then occurs between gametes to produce diploid zygotes. The frequencies of XX zygotes are then denoted as xx_{ij}, XY zygotes as xy_{ij}, and YY zygotes as yy_{ij}, where **A** and **M** locus genotypes are given by $i, j \in \{1, 2, 3, 4\}$, as above. In XY zygotes, the haplotype inherited from an X-bearing gamete is given by i and the haplotype from a Y-bearing gamete is given by j. In XX and YY zygotes, individuals with diploid genotype ij are equivalent to those with diploid genotype ji; for simplicity, we use xx_{ij} and yy_{ij} with $i \neq j$ to denote the average of these frequencies, $xx_{ij} = (x_i^{♀,s} x_j^{♂,s} + x_j^{♀,s} x_i^{♂,s})/2$ and $yy_{ij} = (y_i^{♀,s} y_j^{♂,s} + y_j^{♀,s} y_i^{♂,s})/2$.

Denoting the **M** locus genotype by $b \in \{MM, Mm, mm\}$ and the **X** locus genotype by $c \in \{XX, XY, YY\}$, zygotes develop as females with probability k_{bc}. Therefore, the frequencies of XX females are given by $xx_{ij}^♀ = k_{bc} xx_{ij}$, XY females are given by $xy_{ij}^♀ = k_{bc} xy_{ij}$, and YY females are given by $yy_{ij}^♀ = k_{bc} yy_{ij}$. Similarly, XX male frequencies are $xx_{ij}^♂ = (1 - k_{bc}) xx_{ij}$, XY male frequencies are $xy_{ij}^♂ = (1 - k_{bc}) xy_{ij}$, and YY males frequencies are $yy_{ij}^♂ = (1 - k_{bc}) yy_{ij}$. This notation allows both the ancestral and novel sex-determining regions to determine zygotic sex according to an XY system, a ZW system, or an environmental sex-determining system. In addition, we can consider any epistatic dominance relationship between the two sex-determining loci. Here, we assume that the ancestral sex-determining system (**X** locus) is XY ($k_{MMXX} = 1$ and $k_{MMXY} = k_{MMYY} = 0$) or ZW ($k_{MMZZ} = 0$ and $k_{MMZW} = k_{MMWW} = 1$) and epistatically recessive to a dominant novel sex-determining locus, **M** ($k_{Mmc} = k_{mmc} = k$).

Selection among diploids then occurs according to the diploid genotype at the **A** locus, $l \in \{AA, Aa, aa\}$, for an individual of type ij (see Table 1). The diploid frequencies after selection in sex $\circ$ are given by $xx_{ij}^{\circ,s} = w_l^\circ xx_{ij} / \bar{w}_D^\circ$, $xy_{ij}^{\circ,s} = w_l^\circ xy_{ij} / \bar{w}_D^\circ$, and $yy_{ij}^{\circ,s} = w_l^\circ yy_{ij} / \bar{w}_D^\circ$, where $\bar{w}_D^\circ = \sum_{i=1}^4 \sum_{j=1}^4 w_l^\circ xx_{ij} + w_l^\circ xy_{ij} + w_l^\circ yy_{ij}$ is the mean fitness of diploids of sex $\circ$.

Finally, these diploids undergo meiosis to produce the next generation of gametes. Recombination and sex-specific meiotic drive occur during meiosis. Here, we allow any relative locations for the **X**, **A**, and **M** loci by using three parameters to describe the recombination rates between them. R is the recombination rate between the **A** and **M** loci, ρ is the recombination rate between the **M** and **X** loci, and r is the recombination rate between the **A** and **X** loci (Fig 1). S1 Table shows replacements that can be made for each possible ordering of the loci assuming that there is no cross-over interference. During meiosis in sex $\circ$, meiotic drive occurs such that,

in Aa heterozygotes, a fraction α° of gametes produced carry the A allele and $(1-\alpha^\circ)$ carry the a allele.

Among gametes from sex $\circ$, the frequencies of haplotypes (before gametic competition) in the next generation are given by

$$\begin{aligned} x_1^{\circ\prime} =& xx_{11}^{\circ,s} + xx_{13}^{\circ,s}/2 + (xx_{12}^{\circ,s} + xx_{14}^{\circ,s})\alpha^\circ \\ &- R(xx_{14}^{\circ,s} - xx_{23}^{\circ,s})\alpha^\circ \\ &+ (xy_{11}^{\circ,s} + xy_{13}^{\circ,s})/2 + (xy_{12}^{\circ,s} + xy_{14}^{\circ,s})\alpha^\circ \\ &- r(xy_{12}^{\circ,s} - xy_{21}^{\circ,s})\alpha^\circ - \rho(xy_{13}^{\circ,s} - xy_{31}^{\circ,s})/2 \\ &+ \big[-(R+r+\rho)xy_{14}^{\circ,s} + (R+\rho-r)xy_{41}^{\circ,s} \\ &+ (R+r-\rho)xy_{23}^{\circ,s} + (R+\rho-r)xy_{32}^{\circ,s}\big]\alpha^\circ/2 \end{aligned} \tag{S1.1a}$$

$$\begin{aligned} x_2^{\circ\prime} =& xx_{22}^{\circ,s} + xx_{24}^{\circ,s}/2 + (xx_{12}^{\circ,s} + xx_{23}^{\circ,s})\alpha^\circ \\ &- R(xx_{23}^{\circ,s} - xx_{14}^{\circ,s})\alpha^\circ \\ &(xy_{22}^{\circ,s} + xy_{24}^{\circ,s})/2 + (xy_{21}^{\circ,s} + xy_{23}^{\circ,s})(1-\alpha^\circ) \\ &- r(xy_{21}^{\circ,s} - xy_{12}^{\circ,s})(1-\alpha^\circ) - \rho(xy_{24}^{\circ,s} - xy_{42}^{\circ,s})/2 \\ &+ \big[-(R+r+\rho)xy_{23}^{\circ,s} + (R+\rho-r)xy_{32}^{\circ,s} \\ &+ (R+r-\rho)xy_{14}^{\circ,s} + (R+\rho-r)xy_{41}^{\circ,s}\big](1-\alpha^\circ)/2 \end{aligned} \tag{S1.1b}$$

$$\begin{aligned} x_3^{\circ\prime} =& xx_{33}^{\circ,s} + xx_{13}^{\circ,s}/2 + (xx_{23}^{\circ,s} + xx_{34}^{\circ,s})\alpha^\circ \\ &- R(xx_{23}^{\circ,s} - xx_{14}^{\circ,s})\alpha^\circ \\ &(xy_{33}^{\circ,s} + xy_{31}^{\circ,s})/2 + (xy_{32}^{\circ,s} + xy_{34}^{\circ,s})\alpha^\circ \\ &- r(xy_{34}^{\circ,s} - xy_{43}^{\circ,s})\alpha^\circ - \rho(xy_{31}^{\circ,s} - xy_{13}^{\circ,s})/2 \\ &+ \big[-(R+r+\rho)xy_{32}^{\circ,s} + (R+\rho-r)xy_{23}^{\circ,s} \\ &+ (R+r-\rho)xy_{41}^{\circ,s} + (R+\rho-r)xy_{14}^{\circ,s}\big]\alpha^\circ/2 \end{aligned} \tag{S1.1c}$$

$$\begin{aligned} x_4^{\circ\prime} =& xx_{44}^{\circ,s} + xx_{34}^{\circ,s}/2 + (xx_{14}^{\circ,s} + xx_{24}^{\circ,s})\alpha^\circ \\ &- R(xx_{14}^{\circ,s} - xx_{23}^{\circ,s})\alpha^\circ \\ &(xy_{44}^{\circ,s} + xy_{42}^{\circ,s})/2 + (xy_{41}^{\circ,s} + xy_{43}^{\circ,s})(1-\alpha^\circ) \\ &- r(xy_{43}^{\circ,s} - xy_{34}^{\circ,s})(1-\alpha^\circ) - \rho(xy_{42}^{\circ,s} - xy_{24}^{\circ,s})/2 \\ &+ \big[-(R+r+\rho)xy_{41}^{\circ,s} + (R+\rho-r)xy_{14}^{\circ,s} \\ &+ (R+r-\rho)xy_{32}^{\circ,s} + (R+\rho-r)xy_{23}^{\circ,s}\big](1-\alpha^\circ)/2 \end{aligned} \tag{S1.1d}$$

$$\begin{aligned} y_1^{\circ\prime} =& yy_{11}^{\circ,s} + yy_{13}^{\circ,s}/2 + (yy_{12}^{\circ,s} + yy_{14}^{\circ,s})\alpha^\circ \\ &- R(yy_{14}^{\circ,s} - yy_{23}^{\circ,s})\alpha^\circ \\ &(xy_{11}^{\circ,s} + xy_{31}^{\circ,s})/2 + (xy_{21}^{\circ,s} + xy_{41}^{\circ,s})\alpha^\circ \\ &- r(xy_{21}^{\circ,s} - xy_{12}^{\circ,s})\alpha^\circ - \rho(xy_{31}^{\circ,s} - xy_{13}^{\circ,s})/2 \\ &+ \big[-(R+r+\rho)xy_{41}^{\circ,s} + (R+\rho-r)xy_{14}^{\circ,s} \\ &+ (R+r-\rho)xy_{32}^{\circ,s} + (R+\rho-r)xy_{23}^{\circ,s}\big]\alpha^\circ/2 \end{aligned} \tag{S1.1e}$$

$$\begin{aligned}
y_2^{\circ\prime} =& yy_{22}^{\circ,s} + yy_{24}^{\circ,s}/2 + (yy_{12}^{\circ,s} + yy_{23}^{\circ,s})\alpha^\circ \\
& - R(yy_{23}^{\circ,s} - yy_{14}^{\circ,s})\alpha^\circ \\
& (xy_{22}^{\circ,s} + xy_{42}^{\circ,s})/2 + (xy_{12}^{\circ,s} + xy_{32}^{\circ,s})(1-\alpha^\circ) \\
& - r(xy_{12}^{\circ,s} - xy_{21}^{\circ,s})(1-\alpha^\circ) - \rho(xy_{42}^{\circ,s} - xy_{24}^{\circ,s})/2 \\
& + \big[-(R+r+\rho)xy_{32}^{\circ,s} + (R+\rho-r)xy_{23}^{\circ,s} \\
& + (R+r-\rho)xy_{41}^{\circ,s} + (R+\rho-r)xy_{14}^{\circ,s}\big](1-\alpha^\circ)/2
\end{aligned} \tag{S1.1f}$$

$$\begin{aligned}
y_3^{\circ\prime} =& yy_{33}^{\circ,s} + yy_{13}^{\circ,s}/2 + (yy_{23}^{\circ,s} + yy_{34}^{\circ,s})\alpha^\circ \\
& - R(yy_{23}^{\circ,s} - yy_{14}^{\circ,s})\alpha^\circ \\
& (xy_{33}^{\circ,s} + xy_{13}^{\circ,s})/2 + (xy_{23}^{\circ,s} + xy_{43}^{\circ,s})\alpha^\circ \\
& - r(xy_{43}^{\circ,s} - xy_{34}^{\circ,s})\alpha^\circ - \rho(xy_{13}^{\circ,s} - xy_{31}^{\circ,s})/2 \\
& + \big[-(R+r+\rho)xy_{23}^{\circ,s} + (R+\rho-r)xy_{32}^{\circ,s} \\
& + (R+r-\rho)xy_{14}^{\circ,s} + (R+\rho-r)xy_{41}^{\circ,s}\big]\alpha^\circ/2
\end{aligned} \tag{S1.1g}$$

$$\begin{aligned}
y_4^{\circ\prime} =& yy_{44}^{\circ,s} + yy_{34}^{\circ,s}/2 + (yy_{14}^{\circ,s} + yy_{24}^{\circ,s})\alpha^\circ \\
& - R(yy_{14}^{\circ,s} - yy_{23}^{\circ,s})\alpha^\circ \\
& (xy_{44}^{\circ,s} + xy_{24}^{\circ,s})/2 + (xy_{14}^{\circ,s} + xy_{34}^{\circ,s})(1-\alpha^\circ) \\
& - r(xy_{34}^{\circ,s} - xy_{43}^{\circ,s})(1-\alpha^\circ) - \rho(xy_{24}^{\circ,s} - xy_{42}^{\circ,s})/2 \\
& + \big[-(R+r+\rho)xy_{14}^{\circ,s} + (R+\rho-r)xy_{41}^{\circ,s} \\
& + (R+r-\rho)xy_{23}^{\circ,s} + (R+\rho-r)xy_{32}^{\circ,s}\big](1-\alpha^\circ)/2.
\end{aligned} \tag{S1.1h}$$

The full system is therefore described by 16 recurrence equations (three diallelic loci in two sexes, $2^3 \times 2 = 16$). However, not all diploid types are produced under certain sex-determining systems. For example, with the M allele fixed and an ancestral XY sex-determining system, there are XX females and XY males ($x_3^\circ = x_4^\circ = y_3^\male = y_4^\male = y_i^\female = 0,\ \forall\ i$). In this case, the system only involves six recursion equations, which we assume below to calculate the equilibria.
