# Supplementary material for: Haploid selection, sex ratio bias, and transitions between sex-determining systems
Source: PLoS Biol. 2018 Jun 25;16(6):e2005609. doi: 10.1371/journal.pbio.2005609 (PMC6042799; doi:10.1371/journal.pbio.2005609)
Supplement: S2 Text — (PDF) [file pbio.2005609.s007.pdf]

S2 Appendix

Resident equilibria and stability

In the resident population (allele M fixed), we follow the frequency of A in X-bearing female gametes, $p_X^{\female}$, and X-bearing male gametes, $p_X^{\male}$, and Y-bearing male gametes, $p_Y^{\male}$. We also track the total frequency of Y among male gametes, q, which may deviate from $1/2$ due to meiotic drive in males. These four variables determine the frequencies of the six resident gamete types, which sum to one in each sex: $x_1^{\female} = \hat{p}_X^{\female}$, $x_2^{\female} = 1 - \hat{p}_X^{\female}$, $x_1^{\male} = (1-q)\hat{p}_X^{\male}$, $x_2^{\male} = (1-q)(1-\hat{p}_X^{\male})$, $y_1^{\male} = q\hat{p}_Y^{\male}$, and $y_2^{\male} = q(1-\hat{p}_Y^{\male})$. Mean fitnesses in the resident population are given in S2 Table.

Various forms of selection can maintain a polymorphism at the **A** locus, including sexually antagonistic selection, overdominance, conflicts between diploid selection and selection upon haploid genotypes (ploidally-antagonsitic selection, [1]), or a combination of these selective regimes (see below).

In particular special cases, e.g., no sex-differences in selection or meiotic drive ($s^{\male} = s^{\female}$, $h^{\male} = h^{\female}$, and $\alpha^{\male} = \alpha^{\female} = 1/2$), the equilibrium allele frequency and stability can be calculated analytically without assuming anything about the relative strengths of selection and recombination. However, here, we focus on two regimes (tight linkage between **A** and **X** and weak selection) in order to make fewer assumptions about fitnesses.

Tight linkage between X and A loci

We first calculate the equilibria in the ancestral population when the recombination rate between the **X** and **A** loci is small (r of order ϵ). Selection at the **A** locus will not affect evolution at the novel sex-determining locus, **M**, if one allele is fixed on all backgrounds. We therefore focus on the five equilibria that maintain both A and a alleles, four of which are given to leading order by:

$$(A) \quad \hat{p}_Y^{\male} = 0, \ \hat{q} = \frac{1}{2}\left(1 - \alpha_\Delta^{\male}\frac{w_{Aa}^{\male}\phi}{w_{Aa}^{\male}\phi + w_{aa}^{\male}\psi}\right), \tag{S2.1a}$$

$$\hat{p}_X^{\female} = \frac{w_a^{\female}\phi}{w_a^{\female}\phi + w_A^{\female}\psi}, \ \hat{p}_X^{\male} = \frac{(1+\alpha_\Delta^{\male})w_{Aa}^{\male}\phi}{(1+\alpha_\Delta^{\male})w_{Aa}^{\male}\phi + w_{aa}^{\male}\psi}$$

$$(A') \quad \hat{p}_Y^{\male} = 1, \ \hat{q} = \frac{1}{2}\left(1 + \alpha_\Delta^{\male}\frac{w_{Aa}^{\male}\phi'}{w_{Aa}^{\male}\phi' + w_{AA}^{\male}\psi'}\right), \tag{S2.1b}$$

$$\hat{p}_X^{\female} = 1 - \frac{w_A^{\female}\phi'}{w_A^{\female}\phi' + w_a^{\female}\psi'}, \ \hat{p}_X^{\male} = 1 - \frac{(1-\alpha_\Delta^{\male})w_{Aa}^{\male}\phi'}{(1-\alpha_\Delta^{\male})w_{Aa}^{\male}\phi' + w_{AA}^{\male}\psi'}$$

$$(B) \quad \hat{p}_Y^{\male} = 0, \ \hat{p}_X^{\female} = 1, \ \hat{p}_X^{\male} = 1, \ \hat{q} = (1-\alpha_\Delta^{\male})/2 \tag{S2.1c}$$

$$(B') \quad \hat{p}_Y^{\male} = 1, \ \hat{p}_X^{\female} = 0, \ \hat{p}_X^{\male} = 0, \ \hat{q} = (1+\alpha_\Delta^{\male})/2 \tag{S2.1d}$$

$$\begin{aligned}
\phi &= (1+\alpha_\Delta^{\female})w_A^{\female}w_{Aa}^{\female}\left[w_a^{\male}w_{aa}^{\male} + (1+\alpha_\Delta^{\male})w_A^{\male}w_{Aa}^{\male}\right]/2 - w_a^{\male}w_a^{\female}w_{aa}^{\male}w_{aa}^{\female} \\
\psi &= (1-\alpha_\Delta^{\female})w_a^{\female}w_{Aa}^{\female}\left[w_a^{\male}w_{aa}^{\male} + (1+\alpha_\Delta^{\male})w_A^{\male}w_{Aa}^{\male}\right]/2 - (1+\alpha_\Delta^{\male})w_A^{\male}w_A^{\female}w_{Aa}^{\male}w_{AA}^{\female} \\
\phi' &= (1-\alpha_\Delta^{\female})w_a^{\female}w_{Aa}^{\female}\left[w_A^{\male}w_{AA}^{\male} + (1-\alpha_\Delta^{\male})w_a^{\male}w_{Aa}^{\male}\right]/2 - w_A^{\male}w_A^{\female}w_{AA}^{\male}w_{AA}^{\female} \\
\psi' &= (1+\alpha_\Delta^{\female})w_A^{\female}w_{Aa}^{\female}\left[w_A^{\male}w_{AA}^{\male} + (1-\alpha_\Delta^{\male})w_a^{\male}w_{Aa}^{\male}\right]/2 - (1-\alpha_\Delta^{\male})w_a^{\male}w_a^{\female}w_{Aa}^{\male}w_{aa}^{\female}.
\end{aligned}$$

A fifth equilibrium (C) also exists where A is present at an intermediate frequency on the Y background ($0 < \hat{p}_Y^{♂} < 1$). However, equilibrium (C) is never locally stable when $r \approx 0$ and is therefore not considered further. Thus, the Y background can either be fixed for the a allele (equilibria (A) and (B)) or the A allele (equilibria (A') and (B')). The X background can then either be polymorphic (equilibria (A) and (A')) or fixed for the alternative allele (equilibria (B) and (B')). Since equilibria (A) and (B) are equivalent to equilibria (A') and (B') with the labelling of A and a alleles interchanged, we discuss only equilibria (A) and (B), in which the Y background is fixed for the a allele. If there is no haploid selection ($\alpha_\Delta^{\circ} = 0$, $w_A^{\circ} = w_a^{\circ} = 1$), these equilibria are equivalent to those found by [2] and [3].

We next calculate when equilibria (A) and (B) are locally stable for $r = 0$. According to the 'small parameter theory' [4, 5], these stability properties are unaffected by small amounts of recombination between the sex-determining locus and the **A** locus, although equilibrium frequencies may be slightly altered. For the a allele to be stably fixed on the Y background we need $\bar{w}_{Ya}^{♂} > \bar{w}_{YA}^{♂}$ where $\bar{w}_{Ya}^{♂} = w_a^{♂}\left[\hat{p}_X^{♀}(1-\alpha_\Delta^{♂})w_A^{♀}w_{Aa}^{♂} + (1-\hat{p}_X^{♀})w_a^{♀}w_{aa}^{♂}\right]$ and $\bar{w}_{YA}^{♂} = w_A^{♂}\left[\hat{p}_X^{♀}w_A^{♀}w_{AA}^{♂} + (1-\hat{p}_X^{♀})(1+\alpha_\Delta^{♂})w_a^{♀}w_{Aa}^{♂}\right]$. That is, Y-$a$ haplotypes must have higher fitness than Y-A haplotypes. Substituting in $\hat{p}_X^{♀}$ from Eq (S2.1), fixation of the a allele on the Y background requires that $\gamma_i > 0$ where $\gamma_{(A)} = w_a^{♂}\left[(1-\alpha_\Delta^{♂})w_{Aa}^{♂}\phi + w_{aa}^{♂}\psi\right] - w_A^{♂}\left[w_{AA}^{♂}\phi + (1+\alpha_\Delta^{♂})w_{Aa}^{♂}\psi\right]$ for equilibrium (A) and $\gamma_{(B)} = (1-\alpha_\Delta^{♂})w_a^{♂}w_{Aa}^{♂} - w_A^{♂}w_{AA}^{♂}$ for equilibrium (B). Stability of a polymorphism on the X background (equilibrium (A)) further requires that $\phi > 0$ and $\psi > 0$. Fixation of the a allele on the X background (equilibrium (B)) can be stable only if equilibrium (A) is not, as it requires $\psi < 0$.

Selection weak relative to recombination

Here, we assume that selection is weak relative to recombination (s°, t°, α_Δ° of order ϵ). The maintenance of a polymorphism at the **A** locus then requires that

$$\begin{aligned} &0 < -\left[(1-h^{♀})s^{♀} + (1-h^{♂})s^{♂} + t^{♀} + t^{♂} + \alpha_\Delta^{♀} + \alpha_\Delta^{♂}\right] \\ \text{and}\quad &0 < h^{♀}s^{♀} + h^{♂}s^{♂} + t^{♀} + t^{♂} + \alpha_\Delta^{♀} + \alpha_\Delta^{♂}. \end{aligned} \tag{S2.2}$$

which indicates that a polymorphism can be maintained by various selective regimes.

Given that a polymorphism is maintained at the **A** locus by weak selection, the frequencies of A in each type of gamete are the same ($\hat{p}_X^{♀} = \hat{p}_X^{♂} = \hat{p}_Y^{♂} = \bar{p}$) and given, to leading order, by

$$\bar{p} = \frac{h^{♀}s^{♀} + h^{♂}s^{♂} + t^{♀} + t^{♂} + \alpha_\Delta^{♀} + \alpha_\Delta^{♂}}{(2h^{♀}-1)s^{♀} + (2h^{♂}-1)s^{♂}} + O(\epsilon). \tag{S2.3}$$

Differences in frequency between gamete types are of $O(\epsilon)$:

$$\begin{aligned} \hat{p}_X^{♂} - \hat{p}_X^{♀} &= V_A\left(D^{♂} - D^{♀} + \alpha_\Delta^{♂} - \alpha_\Delta^{♀}\right) + O(\epsilon^2) \\ \hat{p}_Y^{♂} - \hat{p}_X^{♀} &= V_A\left[D^{♂} - D^{♀} + \alpha_\Delta^{♂} - \alpha_\Delta^{♀} + (1-2r)(t^{♂} - t^{♀})\right]/2r + O(\epsilon^2) \\ \hat{p}_Y^{♂} - \hat{p}_X^{♂} &= V_A\left(D^{♂} - D^{♀} + \alpha_\Delta^{♂} - \alpha_\Delta^{♀} + t^{♂} - t^{♀}\right)(1-2r)/2r + O(\epsilon^2), \end{aligned} \tag{S2.4}$$

where $V_A = \bar{p}(1-\bar{p})$ is the variance in the frequency of A and $D^{\circ} = \left[\bar{p}s^{\circ} + (1-\bar{p})h^{\circ}s^{\circ}\right] - \left[\bar{p}h^{\circ}s^{\circ} + (1-\bar{p})\right]$ corresponds to the difference in fitness between A and a alleles in diploids of sex $\circ \in \{♀, ♂\}$. The frequency of Y-bearing male gametes depends upon the difference in the frequency of the A allele between X- and Y-bearing male gametes and the strength of meiotic drive in favour of the A allele in males, $q = 1/2 + \alpha_\Delta^{♂}(\hat{p}_Y^{♂} - \hat{p}_X^{♂})/2 + O(\epsilon^3)$. Without gametic competition or drive ($\alpha_\Delta^{\circ} = t^{\circ} = 0$) our results reduce to those of [6].

References

1. Immler S, Arnqvist G, Otto SP. Ploidally antagonistic selection maintains stable genetic polymorphism. Evolution. 2012;66(1):55–65.
2. Lloyd DG, Webb C. Secondary sex characters in plants. Botanical Review. 1977;43:177–216.
3. Otto SP. Selective maintenance of recombination between the sex chromosomes. Journal of Evolutionary Biology. 2014;27:1431–1442.
4. Karlin S, McGregor J. Application of method of small parameters to multi-niche population genetic models. Theoretical Population Biology. 1972;3(2):186–209.
5. Karlin S, McGregor J. Polymorphisms for genetic and ecological systems with weak coupling. Theoretical Population Biology. 1972;3(2):210–238.
6. van Doorn GS, Kirkpatrick M. Turnover of sex chromosomes induced by sexual conflict. Nature. 2007;449(7164):909–912.
