# Supplementary material for: Haploid selection, sex ratio bias, and transitions between sex-determining systems
Source: PLoS Biol. 2018 Jun 25;16(6):e2005609. doi: 10.1371/journal.pbio.2005609 (PMC6042799; doi:10.1371/journal.pbio.2005609)
Supplement: S3 Text — (PDF) [file pbio.2005609.s008.pdf]

S3 Appendix

Invasion conditions

A rare sex-determining allele, m, will spread in an XY sex-determining system when the leading eigenvalue, $\lambda_m^{(XY)}$, of the Jacobian matrix derived from the eight mutant recursion equations (given by S1.1c,d,g,h), evaluated at the ancestral equilibrium, is greater than one. Because a neo-Y (neo-W) is always in males (females) and is epistatically dominant to the ancestral sex-determining locus, the characteristic polynomial factors into two quadratics times $(\lambda_m^{(XY)})^4$, which greatly simplifies analysis. One quadratic governs the change in the frequency of the new sex determination factor associated with the A and a allele frequencies, summing across backgrounds at the old sex-determining locus (i.e., summing haplotypes bearing the original X and Y alleles). The second quadratic describes the dynamics of the difference in the m-A and m-a haplotypes between X and Y backgrounds. Asymptotically, this difference is constrained to grow at a rate equal to or less than the sum while the m allele is rare, because the frequencies can never become negative. We therefore focus on the leading eigenvalue, $\lambda_m^{(XY)}$, which is the largest root of the quadratic $f(x) = x^2 + bx + c = 0$, where $b = -(\Lambda_{mA}^{(XY)} + \Lambda_{ma}^{(XY)}) + (\chi_{mA}^{(XY)} + \chi_{ma}^{(XY)})$ and $c = (\Lambda_{mA}^{(XY)} - \chi_{mA}^{(XY)})(\Lambda_{ma}^{(XY)} - \chi_{ma}^{(XY)}) - \chi_{mA}^{(XY)}\chi_{ma}^{(XY)}$ (details in S1 File), see Table 2.

When $R = 0$ the two roots are $\Lambda_{mA}^{(XY)}$ and $\Lambda_{ma}^{(XY)}$, and the leading eigenvalue is the larger of the two. When $R > 0$ then $f(\Lambda_{mA}^{(XY)})$ and $f(\Lambda_{ma}^{(XY)})$ are of opposite signs, and the leading eigenvalue must fall between these two quantities (details in S1 File). Thus, $\lambda_m^{(XY)} > 1$ if both $\Lambda_{mA}^{(XY)} > 1$ and $\Lambda_{ma}^{(XY)} > 1$; similarly, $\lambda_m^{(XY)} < 1$ if both $\Lambda_{mA}^{(XY)} < 1$ and $\Lambda_{ma}^{(XY)} < 1$. If only one haplotypic growth rate is greater than one ($\Lambda_{ma}^{(XY)} < 1 < \Lambda_{mA}^{(XY)}$ or $\Lambda_{mA}^{(XY)} < 1 < \Lambda_{ma}^{(XY)}$), then $\lambda_m^{(XY)}$ is greater than one when condition (1) is met. Thus, the invasion of a new sex-determining allele is determined by the haplotypic growth rates ($\Lambda_{mj}^{(XY)}$ terms), which do not account for loss due to recombination, and the dissociative force that breaks apart these haplotypes by recombination ($\chi_{mj}^{(XY)}$). For tight linkage between the ancestral sex-determining locus (**X**) and the selected locus (**A**) we can calculate these terms explicitly (see below). For weak selection we approximate the leading eigenvalue with a Taylor series. The leading eigenvalue for any k is given up to order ϵ^2 by Eq (4).

Tight linkage between A and X ($r \approx 0$)

Here, we explore the conditions under which a neo-W invades an XY system assuming that the **A** locus is initially in tight linkage with the ancestral sex-determining locus ($r \approx 0$). We disregard neo-Y mutations, which never spread given that the ancestral population is at a stable equilibrium (see S1 File for proof).

Starting with the simpler (B) equilibrium, the haplotypic growth rates ($\Lambda_{mi}^{(XY)}$) and dissociative forces ($\chi_{mi}^{(XY)}$) are

$$\Lambda_{W'A}^{(XY)} = \left[w_A^{♂}(1+\alpha_\Delta^{♂})\right]^{-1} \frac{w_A^{♀}}{w_A^{♀}} \frac{\left[w_A^{♂}(1+\alpha_\Delta^{♂})w_{AA}^{♀} + w_a^{♂}(1-\alpha_\Delta^{♂})w_{Aa}^{♀}(1+\alpha_\Delta^{♀})\right]}{2w_{AA}^{♀}} \tag{S3.1a}$$

$$\Lambda_{W'a}^{(XY)} = \left[w_A^{♂}(1+\alpha_\Delta^{♂})\right]^{-1} \frac{w_a^{♀}}{w_A^{♀}} \frac{\left[w_A^{♂}(1+\alpha_\Delta^{♂})w_{Aa}^{♀}(1-\alpha_\Delta^{♀}) + w_a^{♂}(1-\alpha_\Delta^{♂})w_{aa}^{♀}\right]}{2w_{AA}^{♀}} \tag{S3.1b}$$

$$\chi_{W'A}^{(XY)} = \frac{1}{2}\left[w_A^{♂}(1+\alpha_\Delta^{♂})\right]^{-1} \frac{w_A^{♀}}{w_A^{♀}} \frac{\left[w_a^{♂}(1-\alpha_\Delta^{♂})w_{Aa}^{♀}(1+\alpha_\Delta^{♀})\right]}{w_{AA}^{♀}} \frac{R}{2} \tag{S3.1c}$$

$$\chi_{W'a}^{(XY)} = \frac{1}{2}\left[w_A^{♂}(1+\alpha_\Delta^{♂})\right]^{-1} \frac{w_a^{♀}}{w_A^{♀}} \frac{\left[w_A^{♂}(1+\alpha_\Delta^{♂})w_{Aa}^{♀}(1-\alpha_\Delta^{♀})\right]}{w_{AA}^{♀}} \frac{R}{2}. \tag{S3.1d}$$

Haploid selection impacts the spread of neo-W haplotypes in three ways (also seen in Table 2). Firstly, the zygotic sex ratio becomes male biased, $\zeta > 1/2$, when the a allele (which is fixed on the Y) is favoured during competition among male gametes or by meiotic drive in males. Specifically, at equilibrium ($\boldsymbol{B}$), female zygote frequency is $1-\zeta = w_A^{♂}(1+\alpha_\Delta^{♂})/(2\bar{w}_H^{♂})$ where $2\bar{w}_H^{♂} = \left[w_a^{♂}(1-\alpha_\Delta^{♂}) + w_A^{♂}(1+\alpha_\Delta^{♂})\right]$ has been canceled out in equations (S3.1) to leave the term $\left[w_A^{♂}(1+\alpha_\Delta^{♂})\right]^{-1}$. Male biased sex ratios facilitate the spread of a neo-W because neo-W alleles cause the zygotes that carry them to develop as the rarer, female, sex.

Secondly, haploid selection in females selects on neo-W haplotypes directly. At equilibrium ($\boldsymbol{B}$), the fitness of female gametes under the ancestral sex-determining system is $w_A^{♀}$ such that the relative fitnesses of neo-W-A and neo-W-a haplotypes during female gametic competition are $w_A^{♀}/w_A^{♀}$ and $w_a^{♀}/w_A^{♀}$ (see terms in equation S3.1). Meiotic drive in females will also change the proportion of gametes that carry the A versus a alleles, which will be produced by heterozygous females in proportions $(1+\alpha_\Delta^{♀})/2$ and $(1-\alpha_\Delta^{♀})/2$, respectively. These terms are only associated with heterozygous females, i.e., they are found alongside $w_{Aa}^{♀}$.

Thirdly, haploid selection in males affects the diploid genotypes of females by altering the allele frequencies in the male gametes with which female gametes pair. At equlibrium ($\boldsymbol{B}$), neo-W female gametes will mate with X-A male gametes with probability $w_A^{♂}(1+\alpha_\Delta^{♂})/(2\bar{w}_H^{♂})$ and Y-a male gametes with probability $w_a^{♂}(1-\alpha_\Delta^{♂})/(2\bar{w}_H^{♂})$, where the $2\bar{w}_H^{♂}$ terms have been canceled in Eq (S3.1) (as mentioned above). Thus, neo-W-A haplotypes are found in AA female diploids with probability $w_A^{♂}(1+\alpha_\Delta^{♂})/(2\bar{w}_H^{♂})$ (e.g., first term in square brackets in the numerator of equation S3.1a) and in Aa female diploids with probability $w_a^{♂}(1-\alpha_\Delta^{♂})/(2\bar{w}_H^{♂})$ (e.g., the second term in square brackets in the numerator of equation S3.1a).

The other terms in equations (S3.1) are more easily interpreted if we assume that there is no haploid selection in either sex, in which case $\Lambda_{W'A}^{(XY)} = (w_{AA}^{♀} + w_{Aa}^{♀})/2w_{AA}^{♀}$ and $\Lambda_{W'a}^{(XY)} = (w_{aa}^{♀} + w_{Aa}^{♀})/2w_{AA}^{♀}$. Neither haplotype can spread under purely sexually-antagonistic selection at equilibrium ($\boldsymbol{B}$), with directional selection in each sex. Essentially, the X is then already as specialized as possible for the female beneficial allele (A is fixed on the X background), and the neo-W often makes daughters with the Y-a haplotype, increasing the flow of a alleles into females, which reduces the fitness of those females.

If selection doesn't uniformly favour A in females, however, neo-W-A haplotypes and/or neo-W-a haplotypes can spread ($\Lambda_{W'A}^{(XY)} > 1$ and/or $\Lambda_{W'a}^{(XY)} > 1$). A neo-W-A haplotype can spread ($\Lambda_{W'A}^{(XY)} > 1$) when $w_{Aa}^{♀} > w_{AA}^{♀}$, despite the fact that a neo-W brings Y-a haplotypes into females. In this case the a allele is favoured by selection in females despite A being fixed on the X background. For this equilibrium to be stable (i.e., to keep A fixed on the X), X-a cannot be overly favoured in females and X-A must be sufficiently favoured in males (for example, by overdominance in males). Specifically, from the stability conditions for equilibrium (B), we must have $w_{Aa}^{♀} < 2w_{AA}^{♀}$ and $w_{Aa}^{♂}/\left[(w_{aa}^{♂} + w_{Aa}^{♂})/2\right] > w_{Aa}^{♀}/w_{AA}^{♀}$.

Still considering $w^{♀}_{Aa} > w^{♀}_{AA}$, the neo-W can also spread alongside the a allele ($\Lambda^{(XY)}_{W'a} > 1$) if $w^{♀}_{aa}$ is large enough such that $(w^{♀}_{Aa} + w^{♀}_{aa})/2 > w^{♀}_{AA}$. This can occur with overdominance or directional selection for a in females (Fig 2B,C). In this case, a is favoured on the ancestral Y background in males and on the ancestral X background in females (comparing Aa to AA genotypes in females) but A is fixed on the X background due to selection in males. The neo-W-a haplotype can spread because it produces females with higher fitness Aa and aa genotypes.

Similar equations can be derived for equilibrium (A) by substituting the equilibrium frequencies into Table 2

$$\Lambda^{(XY)}_{W'A} = \frac{a}{b}\left[w^{♀}_{AA} w^{♂}_{Aa} w^{♂}_{A}(1+\alpha^{♂}_{\Delta})\phi + w^{♀}_{Aa}(1+\alpha^{♂}_{\Delta}) w^{♂}_{a} c\right]/(2w^{♀}_{a}) \tag{S3.2a}$$
$$\Lambda^{(XY)}_{W'a} = \frac{a}{b}\left[w^{♀}_{Aa}(1-\alpha^{♀}_{\Delta}) w^{♂}_{Aa} w^{♂}_{A}(1+\alpha^{♂}_{\Delta})\phi + w^{♀}_{aa} w^{♂}_{a} c\right]/(2w^{♀}_{A}) \tag{S3.2b}$$
$$\chi^{(XY)}_{W'A} = \frac{a}{b}\frac{R}{2}\left[w^{♀}_{Aa}(1+\alpha^{♀}_{\Delta}) w^{♂}_{a} c\right]/w^{♀}_{a} \tag{S3.2c}$$
$$\chi^{(XY)}_{W'a} = \frac{a}{b}\frac{R}{2}\left[w^{♀}_{Aa}(1-\alpha^{♀}_{\Delta}) w^{♂}_{Aa} w^{♂}_{A}(1+\alpha^{♂}_{\Delta})\phi\right]/w^{♀}_{A}, \tag{S3.2d}$$

where

$$a = w^{♀}_{a}\phi + w^{♀}_{A}\psi \tag{S3.3a}$$
$$b = w^{♀}_{AA}\left[w^{♂}_{Aa} w^{♂}_{A}(1+\alpha^{♂}_{\Delta})\right]\phi^2 + w^{♀}_{Aa}\left[w^{♂}_{Aa} w^{♂}_{A}(1+\alpha^{♂}_{\Delta}) + w^{♂}_{aa} w^{♂}_{a}\right]\psi\phi + w^{♀}_{aa}\left(w^{♂}_{aa} w^{♂}_{a}\right)\psi^2 \tag{S3.3b}$$
$$c = w^{♂}_{Aa}(1-\alpha^{♂}_{\Delta})\phi + 2w^{♂}_{aa}\psi. \tag{S3.3c}$$

As with equilibrium (B), haploid selection again modifies invasion fitnesses by altering the ancestral sex ratio, ζ (see Table 2), and directly selecting upon female gametes, through $w^{♀}_{i}$. The only difference is that resident XX females are no longer always homozygote AA and males are no longer always heterozygote Aa. Thus the effect of haploid selection in males is reduced, as is the difference in fitness between neo-W haplotypes and resident X haplotypes, as both can be on any diploid or haploid background.

The other terms are easier to interpret in the absence of haploid selection. For instance, without haploid selection, the neo-W-A haplotype spreads ($\Lambda^{(XY)}_{W'A} > 1$) if and only if

$$2(w^{♀}_{Aa} - w^{♀}_{aa}) w^{♂}_{aa}\psi^2 > (w^{♀}_{AA} - w^{♀}_{Aa}) w^{♂}_{Aa}\phi(\phi - \psi), \tag{S3.4}$$

where $\phi - \psi = w^{♀}_{AA} w^{♂}_{Aa} - w^{♀}_{aa} w^{♂}_{aa}$ and both ϕ and ψ are positive when equilibrium (A) is stable. In contrast to equilibrium (B), a neo-W haplotype can spread under purely sexually-antagonistic selection ($w^{♀}_{aa} < w^{♀}_{Aa} < w^{♀}_{AA}$ and $w^{♂}_{AA} < w^{♂}_{Aa} < w^{♂}_{aa}$). The neo-W-$A$ can spread as long as it becomes associated with females that bear more A alleles than observed at equilibrium (A), effectively specializing on female fitness.

Without haploid selection, the neo-W-a haplotype spreads ($\Lambda^{(XY)}_{W'a} > 1$) if and only if

$$(w^{♀}_{aa} + w^{♀}_{Aa} - 2w^{♀}_{AA}) w^{♂}_{Aa}\phi^2 + (w^{♀}_{aa} - w^{♀}_{Aa})(w^{♂}_{Aa} + 2w^{♂}_{aa})\phi\psi > 0. \tag{S3.5}$$

This condition cannot be met with purely sexually antagonistic selection (as both terms on the left-hand side would then be negative), but it can be met under other circumstances. For example, with overdominance in males there is selection for increased A frequencies on the X background in males, which are always paired with Y-a haplotypes. Directional selection for a in females can then maintain a polymorphism at the **A** locus on the X background. This scenario selects for a modifier that increases recombination between the sex chromosomes (e.g., blue

region of Fig 2d in [1]) and facilitates the spread of neo-W-a haplotypes, which create females bearing more a alleles than the ancestral X haplotype does.

In absence of haploid selection, the fact that a less closely linked neo-W ($R > 0$) can invade an XY system with tight sex-linkage can also be reached from Equation 7 in [2]; for example, with no polymorphism on the Y ($V_Y = 0$) and an allelic substitution favoured in females ($\alpha^f, \alpha^f_X > 0$) a loosely linked neo-W can invade given the allelic substitution is sufficiently disfavoured on the X in males ($\alpha^m_X < -2\alpha^f_X$), although it is unclear from their implicit equation if and when such an equilibrium is stable.

Role of haploid selection with tight linkage between X and A loci

Haploid selection generally expands the conditions under which neo-W alleles can spread within ancestral systems that have evolved tight linkage between the sex-determining locus and a selected locus ($r \approx 0$). First, haploid selection can allow a polymorphism to be maintained when it would not under diploid selection alone (e.g., with directional selection in diploids). In cases of ploidally-antagonistic selection, where there is a balance between alleles favored in the haploid stage and the diploid stage, neo-W alleles - even if unlinked to the selected locus - can spread (Figure). Second, even when diploid selection could itself maintain a polymorphism, haploid selection can increase the conditions under which transitions among sex-determining systems are possible. Of particularly importance, when selection is sexually-antagonistic in diploids ($s^{♀} s^{♂} < 0$ and $0 < h^{\circ} < 1$), an unlinked neo-W ($R = 1/2$) cannot invade unless there is also haploid selection (see proof in S1 File; Fig 3 and Figure). More generally, haploid selection alters the conditions under which neo-W alleles can spread (compare Figure -Figure to Fig 2).

Male haploid selection in favour of the a allele ($\alpha^{♂}_{\Delta} < 0$, $w^{♂}_A < w^{♂}_a$) generates male-biased sex ratios at equilibria (A) and (B), where Y-a is fixed ($\hat{p}^{♂}_Y = 0$). Male-biased sex ratios facilitate the spread of neo-W-A and neo-W-a haplotypes (increasing $\Lambda^{(XY)}_{W'A}$ and $\Lambda^{(XY)}_{W'a}$). Panels A-C in Figure and show that neo-W haplotypes tend to spread for a wider range of parameters when sex ratios are male biased, compared to Fig 2 without haploid selection. By contrast, male haploid selection in favour of the A allele generates female-biased sex ratios and reduces $\Lambda^{(XY)}_{W'A}$ and $\Lambda^{(XY)}_{W'a}$, as demonstrated by panels D-F in Figure and Figure .

Female haploid selection generates direct selection on the neo-W-A and neo-W-a haplotypes as they spread in females. Thus, female haploid selection in favour of the a allele tends to increase $\Lambda^{(XY)}_{W'a}$ and decrease $\Lambda^{(XY)}_{W'A}$, as shown by panels A-C in Figure and Figure . Conversely, female haploid selection in favour of the A allele increases $\Lambda^{(XY)}_{W'A}$ and decreases $\Lambda^{(XY)}_{W'a}$, see panels D-F in Figure and Figure .

Thus, the impact of haploid selection on transitions between sex-determining systems must be considered as two sides of a coin: it can generate sex ratio biases that promote transitions that equalize the sex ratio, but it can also direct select for transitions that cause sex ratios to become biased.

References

1. Otto SP. Selective maintenance of recombination between the sex chromosomes. Journal of Evolutionary Biology. 2014;27:1431–1442.

2. van Doorn GS, Kirkpatrick M. Transitions between male and female heterogamety caused by sex-antagonistic selection. Genetics. 2010;186(2):629–645.
