# Supplementary figures and images for: Haploid selection, sex ratio bias, and transitions between sex-determining systems
Source: PLoS Biol. 2018 Jun 25;16(6):e2005609. doi: 10.1371/journal.pbio.2005609 (PMC6042799; doi:10.1371/journal.pbio.2005609)

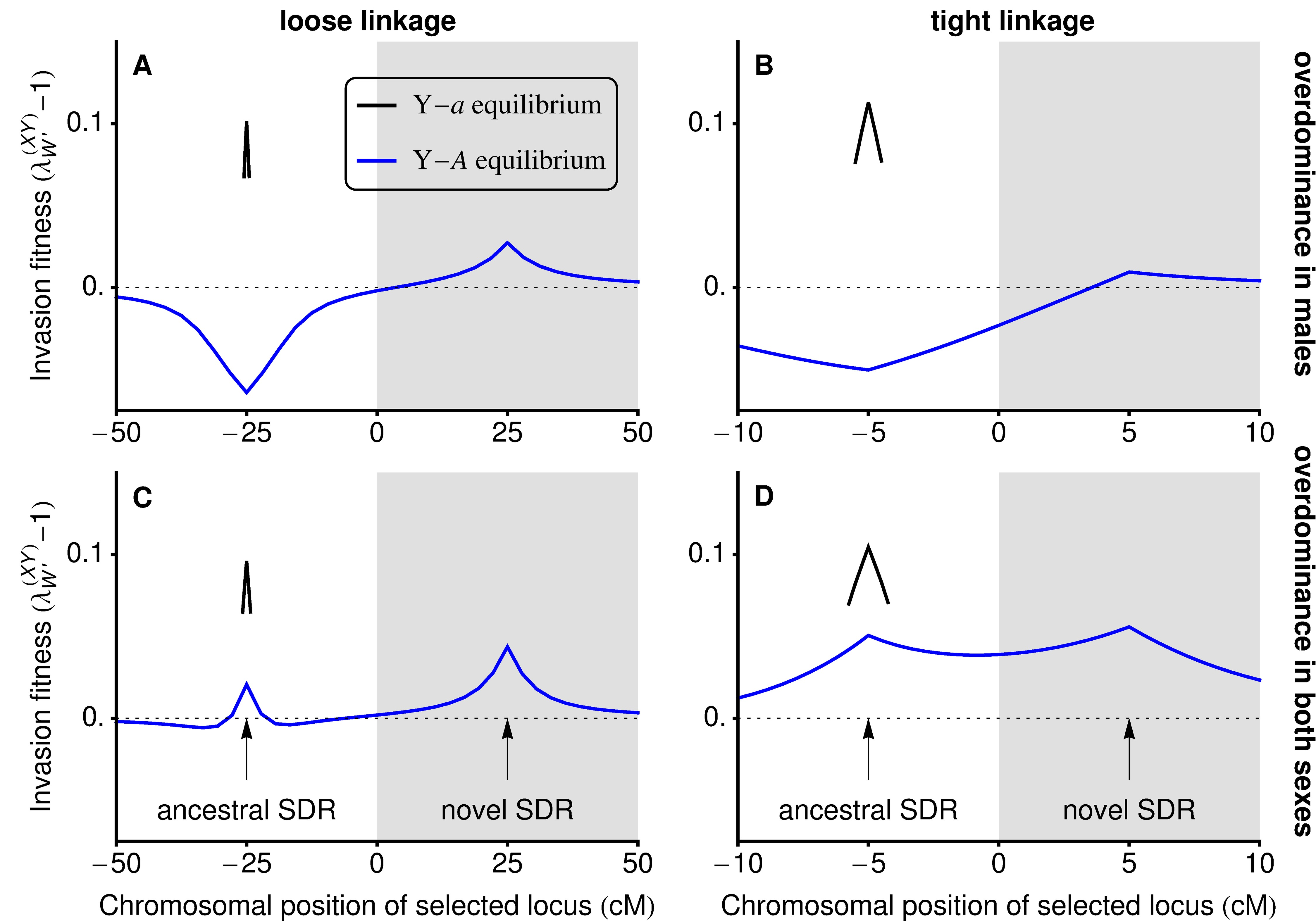

Supplement: S1 Fig — In panels A and B, the a allele is favoured in females (waa♀=1.05, , wAA♀=0.85), and selection in males is overdominant (waa♂=wAA♂=0.75). In panels C and D, selection in males and females is overdominant (waa♀=wAA♀=0.6, waa♂=0.5, wAA♂=0.7, ). There is no haploid selection . These parameters are marked by daggers in Fig 2B and 2C, which show that neo-W invasion is expected for any R (ΛW′A(XY), ΛW′a(XY)>1) if the a allele is nearly fixed on the Y (black lines in this figure; not stable for r ≫ 0). Equilibria in which the A allele is more common among Y-bearing male gametes can also be stable and allow neo-W invasion for these parameters (blue lines). (TIF) [file pbio.2005609.s009.tif]

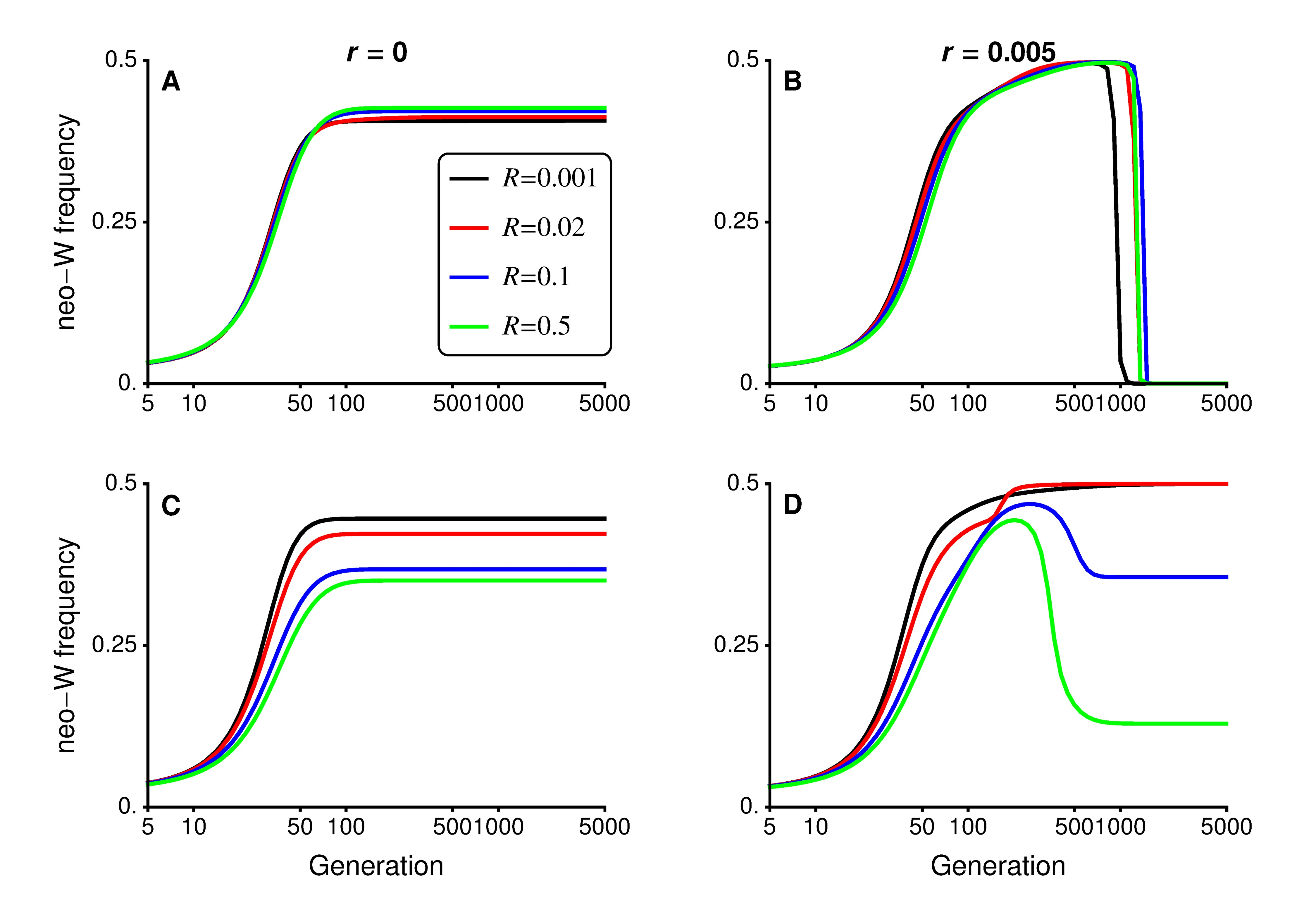

Supplement: S2 Fig — Here, we plot the frequency of the neo-W allele among female gametes. Panels A, C, and D show cases in which a steady state is reached with the neo-W at a frequency below 0.5, in which case ancestral X and Y alleles also both segregate. In all cases, we assume that the a allele is initially more common than the A allele on the Y background (Y-a is fixed when r = 0). When r > 0 (panels B and D), Y-A haplotypes created by recombination can become more common than Y-a haplotypes as the neo-W spreads. In B, this leads to loss of the neo-W, and the system goes to an equilibrium with X-a and Y-A haplotypes fixed (equilibrium A′), such that all females have the high fitness genotype aa, and all males are Aa. For the parameters in B, neo-W alleles have negative invasion fitness when the Y-A haplotype is ancestrally more common than Y-a (compare blue to black curves in S1A Fig and S2B Fig near the ancestral sex-determining locus). In contrast, the neo-W is not lost in panel D, as it is favoured regardless of whether Y-A or Y-a haplotypes predominate (again, compare blue to black curves in S1C and S1D Fig). (TIF) [file pbio.2005609.s010.tif]

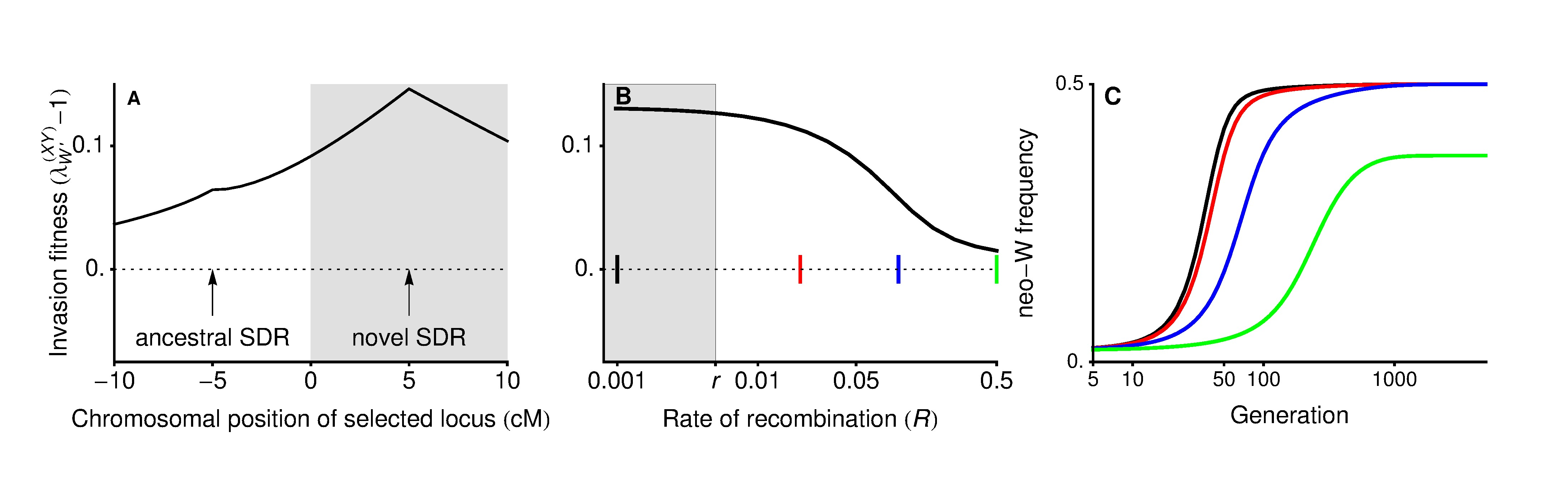

Supplement: S3 Fig — Panel A shows that the invasion fitness of a neo-W is positive, even when r < R (unshaded region). In panel B, we vary the recombination rate between the neo-W and the selected locus (R) for a fixed recombination rate between the ancestral sex-determining locus and the selected locus (r = 0.005). Coloured markers show recombination rates for which the temporal dynamics of neo-W invasion are plotted in panel C (black R = 0.001, red R = 0.02, blue R = 0.1, green R = 0.5). The diploid selection parameters used in this plot are the same as in Fig 3. There is also meiotic drive in males favouring a (αΔ♂=−0.08); this full set of parameters is marked by an asterisk in S4A Fig. When R = 0.5 (green curve), the neo-W does not reach fixation, and X, Y, Z, and W alleles are all maintained in the population; see S9C Fig. (TIF) [file pbio.2005609.s011.tif]

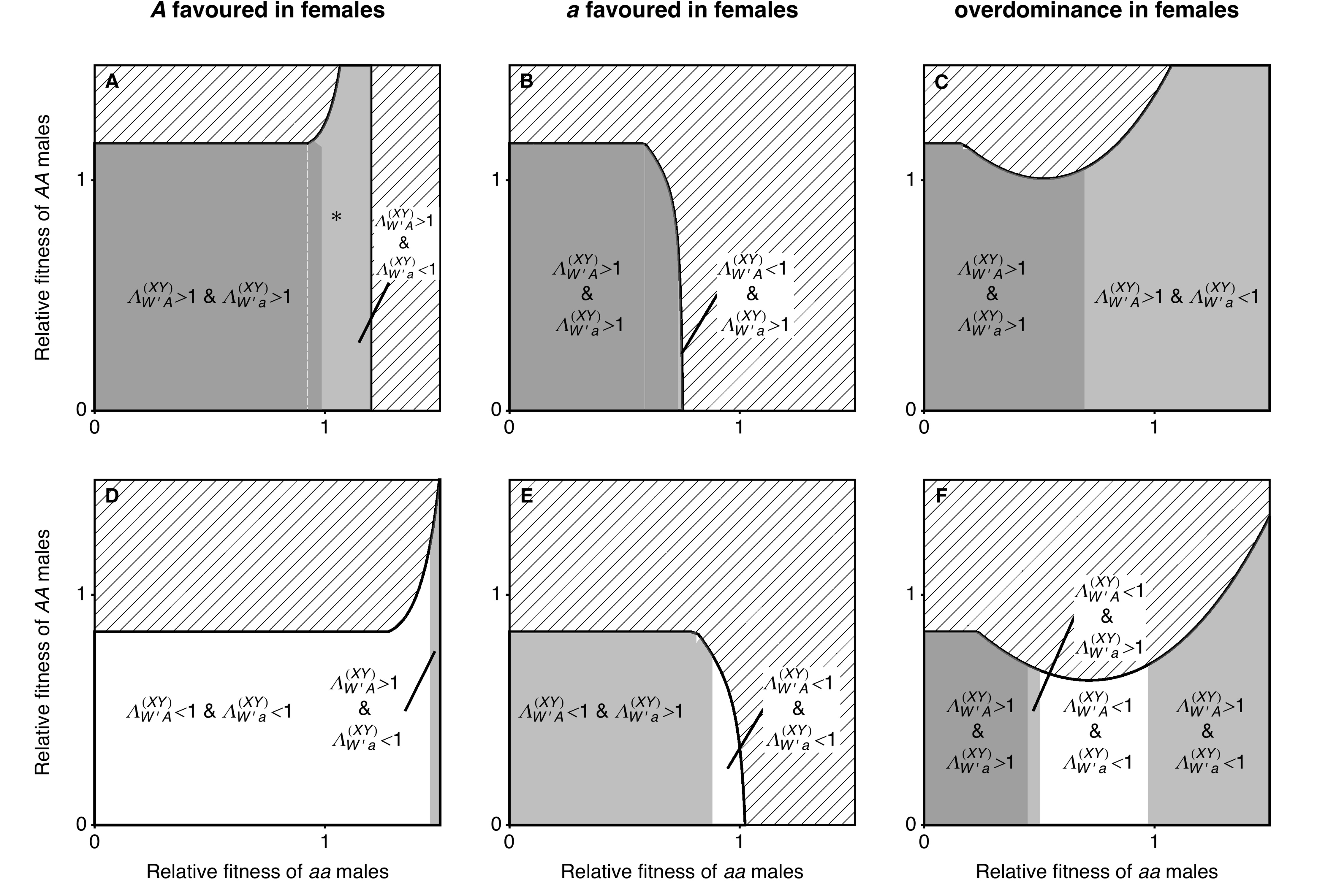

Supplement: S4 Fig — This figure is equivalent to Fig 2 but with meiotic drive in males. In panels A–C, meiotic drive in males favours the a allele (αΔ♂=−0.16), creating male-biased sex ratios and generally increasing ΛW′A(XY) and ΛW′a(XY). By contrast, ΛW′A(XY) and ΛW′a(XY) tend to be reduced when meiotic drive in males favours the A allele (αΔ♂=0.16), panels D–F. (TIF) [file pbio.2005609.s012.tif]

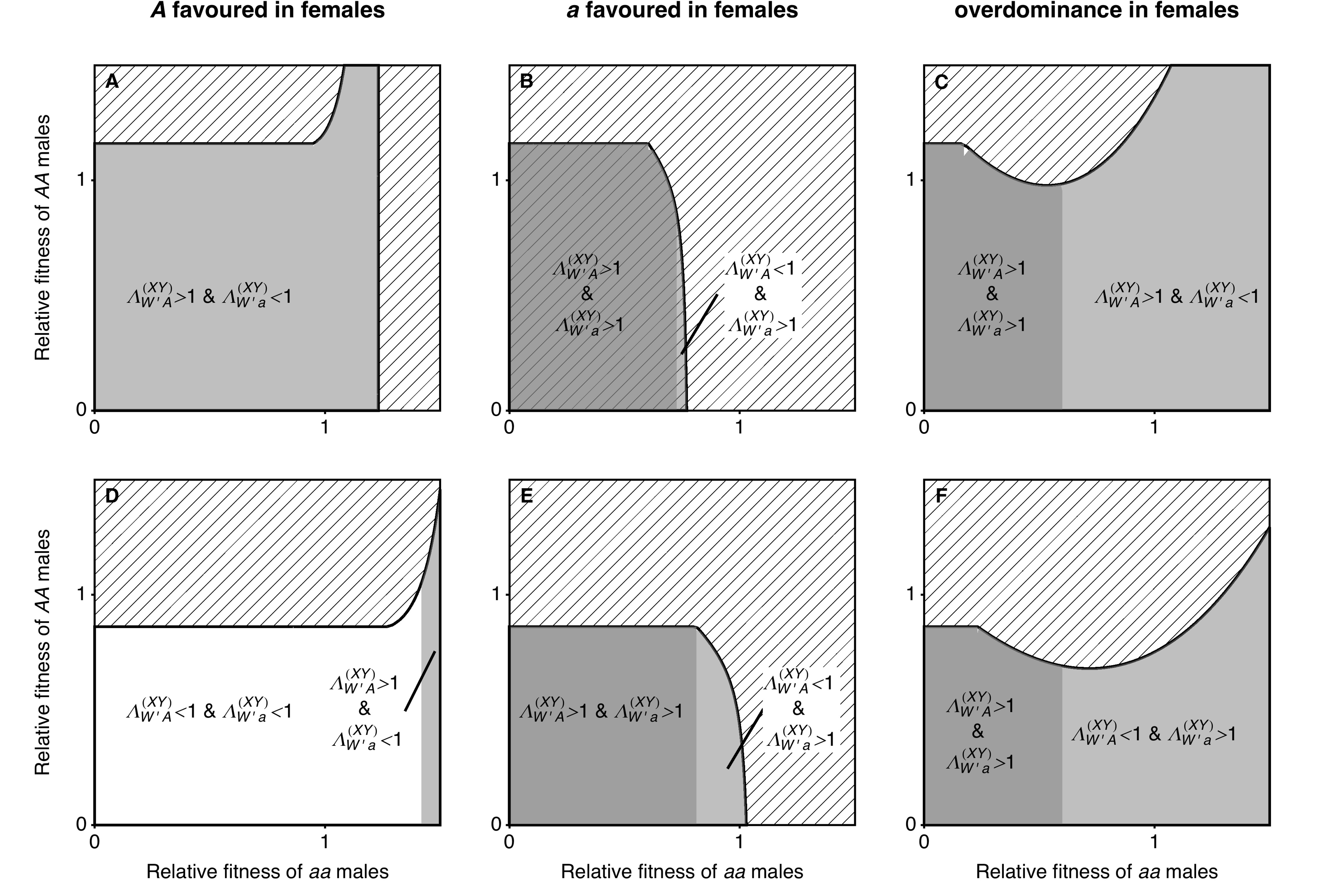

Supplement: S5 Fig — This figure is equivalent to Fig 2 but with gametic competition in males. The a allele is favoured during male gametic competition in panels A–C (wa♂=1.16, wA♂=1), which creates male-biased sex ratios and increases ΛW′A(XY) and ΛW′a(XY). By contrast, ΛW′A(XY) and ΛW′a(XY) tend to be reduced when the A allele is favoured during male gametic competition, panels D–F. Compared to the meiotic drive parameters in S4 Fig, the effect of these male gametic competition parameters on the sex ratio is smaller. For example, in S4A–S4C Fig, the ancestral sex ratio is α♂ = 0.58 at equilibrium (B), and in panels A–C of this plot, the ancestral sex ratio is wa♂/(wA♂+wa♂)=0.537 at equilibrium (B). (TIF) [file pbio.2005609.s013.tif]

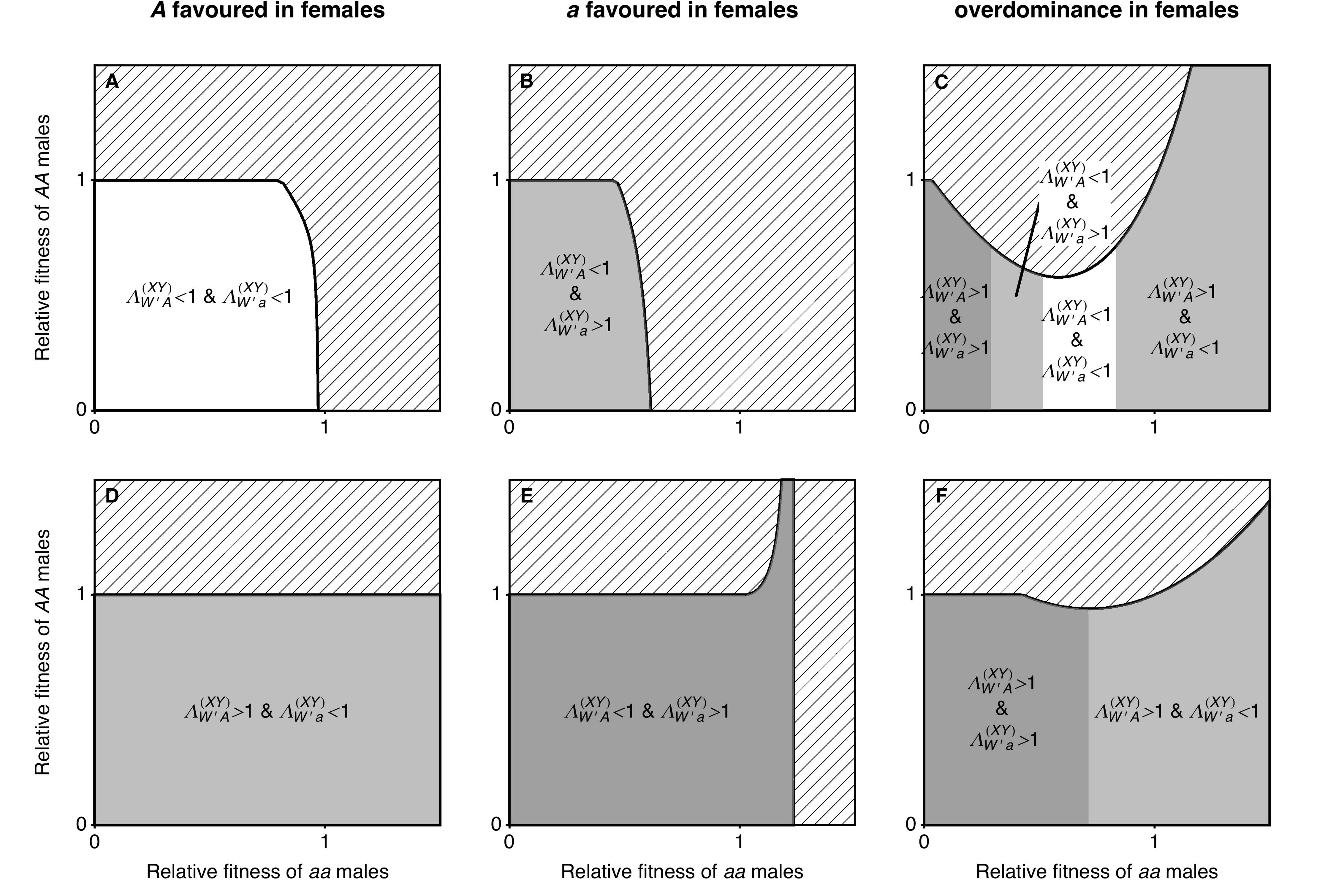

Supplement: S6 Fig — This figure is equivalent to Fig 2 but with meiotic drive in females. The a allele is favoured by meiotic drive in females in panels A–C (αΔ♀=−0.16), which increases ΛW′a(XY) and decreases ΛW′A(XY). Female meiotic drive in favour of the A allele (panels D–F, αΔ♂=−0.16) has the opposite effect. (TIF) [file pbio.2005609.s014.tif]

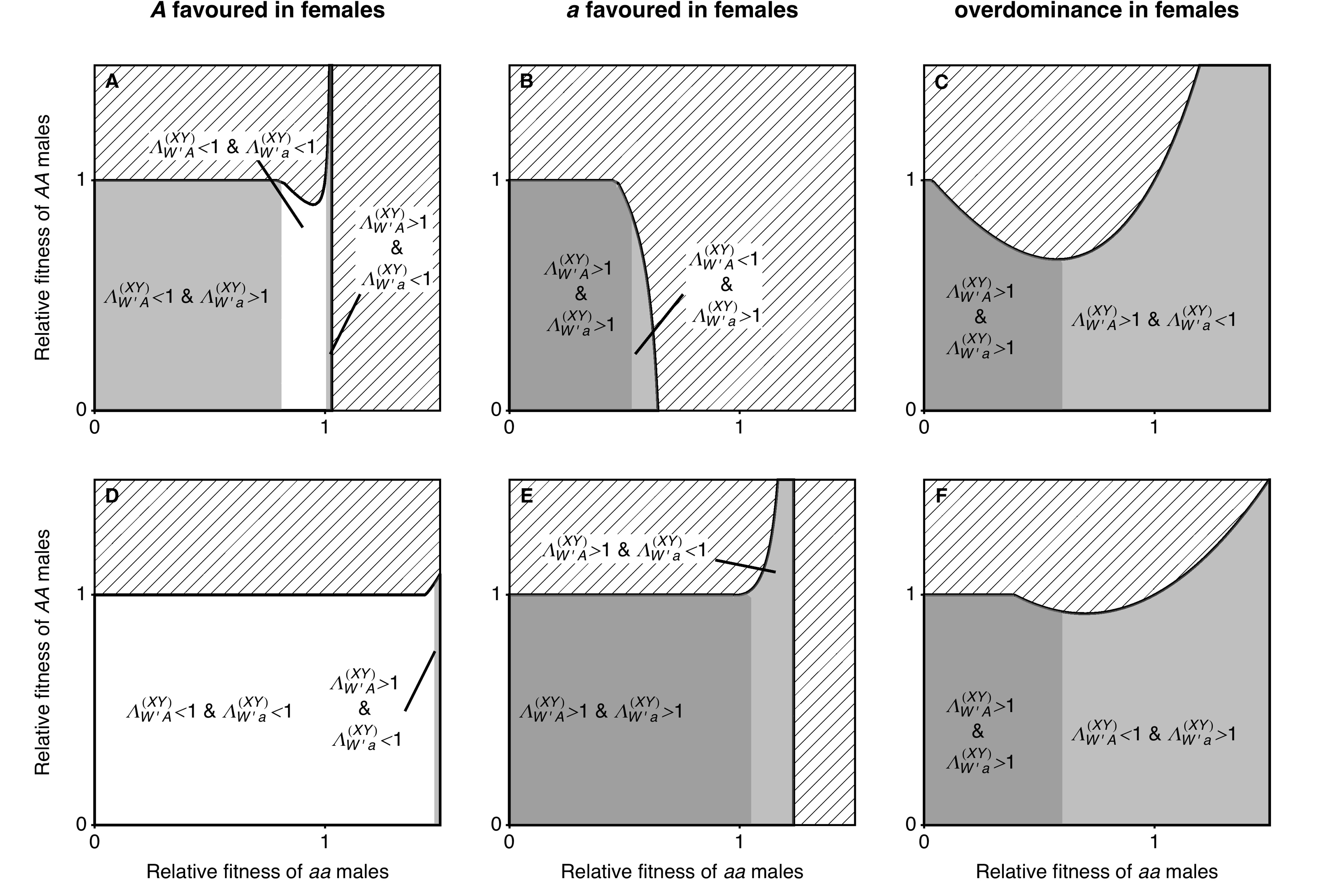

Supplement: S7 Fig — This figure is equivalent to Fig 2 but with gametic competition in females. The a allele is favoured during female gametic competition in females in panels A–C (wa♀=1.16, wA♀=1), which increases ΛW′a(XY) and decreases ΛW′A(XY). The A allele is favoured during gametic competition in panels D–F (wa♀=1, wA♀=1.16), giving the opposite effect on ΛW′a(XY) and ΛW′A(XY). (TIF) [file pbio.2005609.s015.tif]

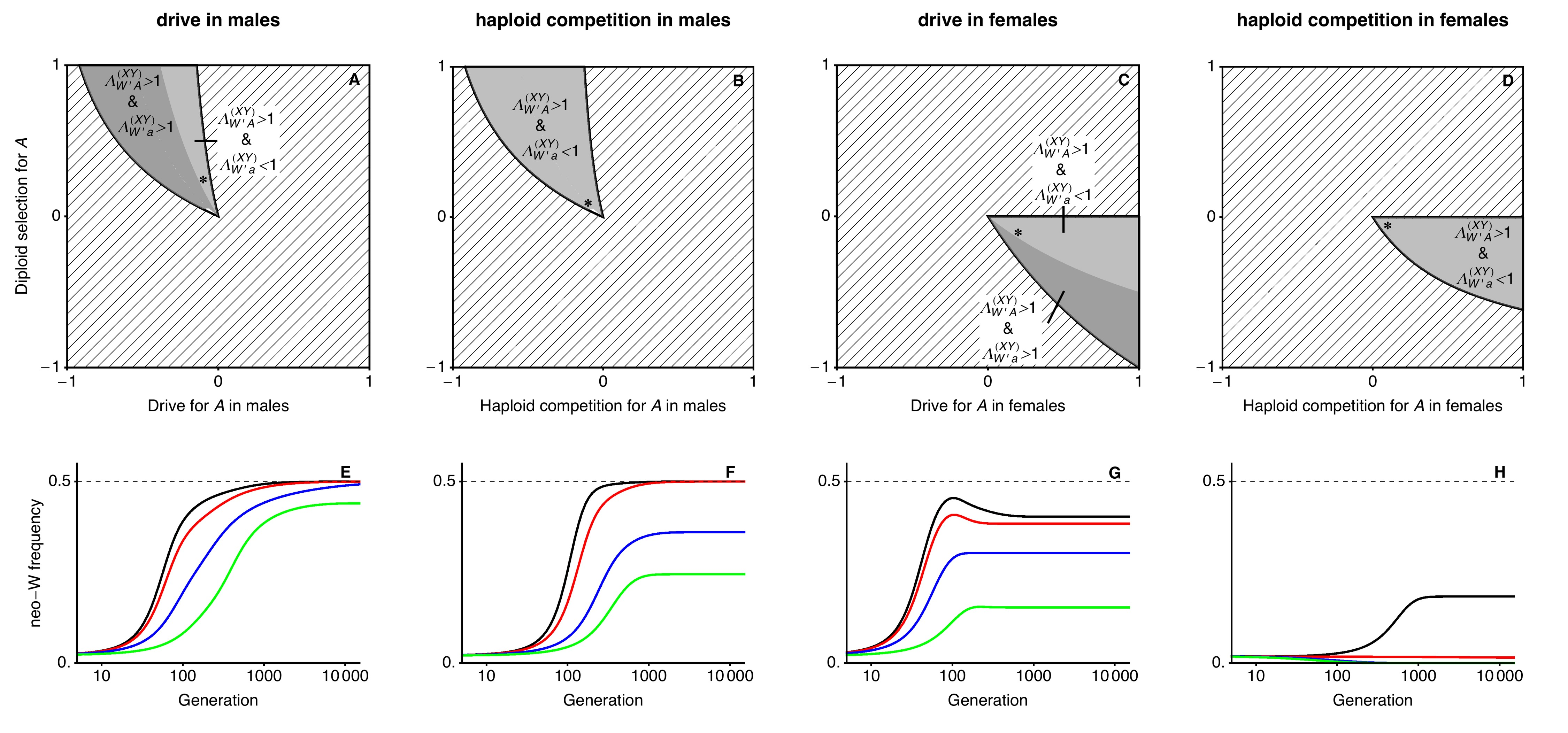

Supplement: S8 Fig — A–D show when each of the neo-W haplotypes invades an internally stable equilibrium with a fixed on the Y (found by setting r = 0). The y-axis shows directional selection in diploids of both sexes, s♀ = s♂, and the x-axes show sex-limited drive, , or haploid competition, . The top-left and bottom-right quadrants therefore imply ploidally antagonistic selection (and these are the only places where neo-W haplotypes can invade). Dominance is equal in both sexes, h♀ = h♂ = 3/4. E–F show the temporal dynamics of neo-W frequency in females, with parameters given by the asterisks in the corresponding A–D plot, with r = 1/200, for four different R. Black R = 1/1000, Red R = 2/100, Blue R = 1/10, Green R = 1/2. (TIF) [file pbio.2005609.s016.tif]

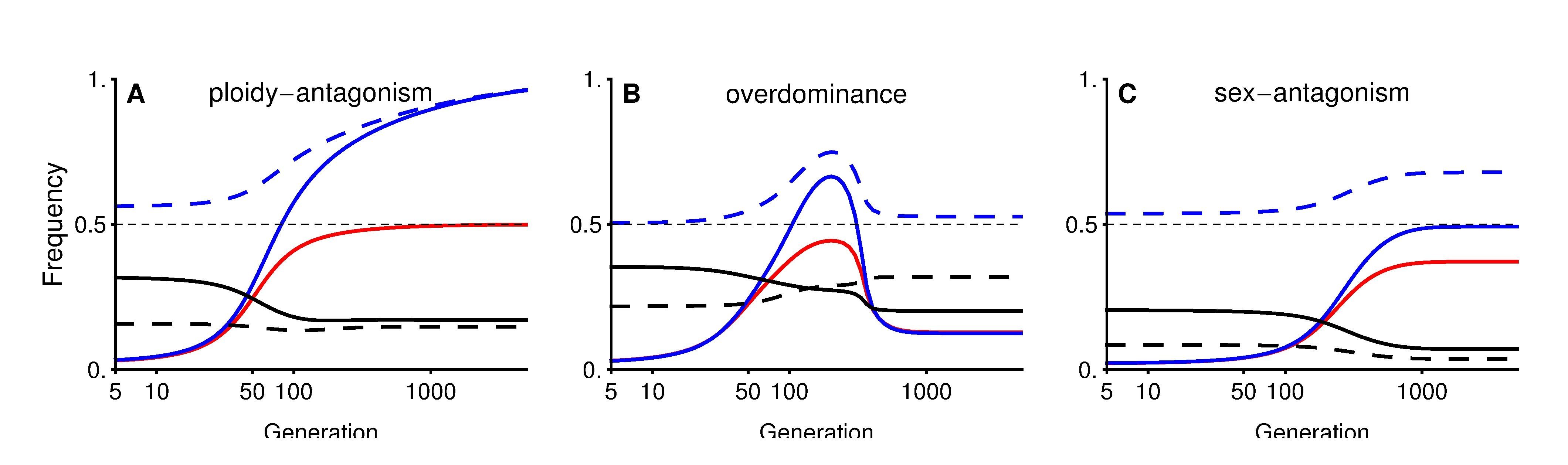

Supplement: S9 Fig — The curves show the frequencies of the neo-W (red), ancestral Y (blue), and A allele (black) among female gametes (solid curves) and among male gametes (dashed curves). In panel A, there is a complete transition from XY sex determination (XX-ZZ females and XY-ZZ males, labeling allele M as Z and the new allele m as W) to ZW sex determination (YY-ZW females and YY-ZZ males). In panels B and C, a polymorphism is maintained at both the ancestral XY locus and the new ZW locus, such that there are males with genotypes XY-ZZ and YY-ZZ and females with genotypes XX-ZZ, XX-ZW, XY-ZW, and YY-ZW. In panel A, selection is ploidally antagonistic with drive in males (parameters as in the green curve in Fig 5B). In panel B, there is overdominance in both sexes and no haploid selection (parameters as in the green curve in S2C Fig). In panel C, there is sexually antagonistic selection in diploids with drive in males (parameters as in the green curve in S4C Fig). In all cases, the initial equilibrium frequency has a near fixation on the Y. (TIF) [file pbio.2005609.s017.tif]
